# West Nile virus vaccine candidates attenuated by dinucleotide enrichment are immunogenic and protective against lethal infection

**DOI:** 10.1371/journal.ppat.1013560

**Published:** 2025-10-03

**Authors:** Nguyen Phuong Khanh Le, Prince Pal Singh, Ivan Trus, Uladzimir Karniychuk

**Affiliations:** 1 Department of Veterinary Biosciences, College of Veterinary Medicine, The Ohio State University, Columbus, Ohio, United States of America; 2 School of Public Health, Vaccinology and Immunotherapeutics Program, University of Saskatchewan, Saskatoon, Canada; 3 Dioscuri Centre for RNA-Protein Interactions in Human Health and Disease, International Institute of Molecular and Cell Biology, Warsaw, Poland; 4 Veterinary Microbiology Department, Wester College of Veterinary Medicine, University of Saskatchewan, Saskatoon, Canada; 5 Center for RNA Biology, The Ohio State University, Columbus, Ohio, United States of America; 6 Infectious Disease Institute, The Ohio State University, Columbus, Ohio, United States of America; National Institute of Allergy and Infectious Diseases Laboratory of Viral Diseases, UNITED STATES OF AMERICA

## Abstract

West Nile virus (WNV) poses a global public health threat. This study demonstrates that the WNV RNA tolerates CpG and UpA dinucleotide enrichment in different genomic regions resulting in attenuation of CpG- and CpG/UpA-enriched variants. Attenuation was zinc finger antiviral protein 1 (ZAP)-dependent, and ZAP knockout (ZAP-KO) cells were used to generate high-titer stocks. Ten enriched variants, with permuted control and wild-type (WT) viruses, were screened in immunocompetent mice upon intraperitoneal injection. In contrast to lethal WNV-WT and permuted viruses, the E-MAX variant, with the RNA region encoding envelope (E) protein enriched both with CpG and UpA, caused no mortality. E-MAX was immunogenic and protective against lethal challenge. Stability of enriched dinucleotides was confirmed upon serial passaging in ZAP-WT and ZAP-KO cells, with only minor (17–21%) reversion at a single site in ZAP-WT condition. E-MAX upregulated interferon (IFN) signaling genes in human cells, suggesting that the combination of CpG/UpA-mediated attenuation, and concurrent activation of IFN responses potentially driven by CpG/UpA enrichment, may contribute to E-MAX immunogenicity. Evaluation using footpad injection in mice showed E-MAX had a promising safety and immunogenicity profile, although brain infection was occasionally detected. Then, we developed the E-MAX+ FR variant by combining CpG/UpA enrichment with two amino acid substitutions in functional domains of the E protein. This strategy eliminated neuroinvasion while maintaining immunogenicity and protection. Altogether, CpG/UpA dinucleotide enrichment in the genomic E region in combination with amino acid substitutions in the E protein yields a promising platform for vaccine development against WNV and potentially other flaviviruses.

## Author summary

West Nile virus (WNV) poses a global public health threat. In humans, WNV damages the central nervous system, leading to meningitis, encephalitis, acute flaccid paralysis, and long-term impairments including memory loss and chronic fatigue. Despite decades of research, no antivirals or human WNV vaccines have been approved. Here, we used cytosine-phosphate-guanine (CpG) and uracil-phosphate-adenine (UpA) dinucleotide enrichment in WNV RNA to attenuate an aggressive virus strain while inducing immunogenicity and single-dose protection against lethal infection. We also assessed a combined vaccine strategy incorporating dinucleotide enrichment with two stable amino acid mutations in functional domains of the WNV E protein to introduce two safety layers.

## Introduction

West Nile virus (WNV; *Orthoflavivirus*, *Flaviviridae*) is a significant global threat. West Nile virus is endemic on all continents except Antarctica. The first European epidemic occurred in Romania in 1996, with subsequent annual outbreaks [1]. In the US, it is the most common and expanding insect-borne zoonotic virus. Since its emergence in the US in 1999, WNV has caused millions of human infections, with thousands of neuroinvasive cases, long-term neuropathology, and annual deaths [2]. In humans, WNV causes central nervous system injury, including meningitis, encephalitis, and acute flaccid paralysis. Survivors may suffer from long-term physical and cognitive impairments. Recovery from severe WNV is often long and requires extensive healthcare resources. WNV has recently been proposed as a neglected tropical disease in North America due to its endemic nature, continued morbidity and mortality, lack of antivirals and approved vaccines, declining research attention and funding [3]. The eradication of WNV is impossible due to its establishment in zoonotic reservoirs [4–6]. This highlights the need for research to develop effective WNV vaccines.

Despite decades of research none of the WNV vaccines for humans have received approval. Two inactivated WNV vaccines, one DNA vaccine, and one recombinant vaccine candidate have entered human clinical trials. However, all of them elicited suboptimal humoral and T cell immune responses, even after 2–3 immunization doses [7]. ChimeriVax, a live-attenuated yellow fever virus (YFV) 17D vector expressing only WNV structural prM/E proteins, is a more promising vaccine candidate [8]. However, flavivirus vaccines based on the heterologous YFV 17D vector have failed to produce optimal T cell responses in humans [9]. The absence of homologous non-structural proteins may limit durability of cellular immune responses and protection [10], given that non-structural flavivirus proteins (i.e., from Zika virus, dengue virus, and WNV) contain essential immunodominant T cell epitopes [9–17].

Live-attenuated vaccines (LAVs) capitalize on single-dose immunization, robust immune responses, and durable protection. The most successful LAVs are represented by vaccines against flaviviruses, YFV 17D and Japanese encephalitis virus (JEV) SA14-14-2, are safe and protective [18,19]. However, introducing mutations that lead to attenuated phenotypes in the 17D and SA14-14-2 failed to deliver broad attenuation platforms. Enrichment of CpG dinucleotides in viral RNA, without altering protein sequences, is an emerging live vaccine approach [20–27]. CpG-enriched vaccines are also live, but in contrast to classical LAVs, CpG enrichment is based on the cumulative effect of many nucleotide mutations. Mechanistically, Zinc-finger CCCH-type antiviral protein 1 (ZAP) targets CpG-rich or CpG-enriched viral RNA for degradation [28]. In addition to CpG enrichment, the enrichment of UpA dinucleotides can also attenuate virus infection [29–31]. The mechanism behind UpA underrepresentation is hypothesized to be caused by RNA-degrading enzymes [32]. Enteroviruses enriched for UpA dinucleotides have increased sensitivity to ZAP and attenuation, though to a lesser extent than CG-enriched variants [29–31].

In this study, we used a rational approach to evaluate the tolerance of the WNV RNA to CpG, UpA, and CpG/UpA dinucleotide enrichment in different genomic regions, and to assess the effects *in vitro*. We also tested the utility of ZAP knockout cells to produce high-titer stocks of dinucleotide-enriched WNV variants, which are otherwise difficult or not possible to generate in wild-type cells. A panel of ten WNV variants was initially evaluated for infection phenotypes in immunocompetent mice susceptible to lethal WNV infection. After identifying the most promising CpG/UpA-enriched WNV variant, we assessed its genetic stability using whole-genome NGS, *in vitro* CpG- and UpA-associated innate immune responses using RNA-seq and Western blot, and neuroinvasion, immunogenicity, and protection in mice. Finally, we tested a combined strategy which included dinucleotide enrichment with two amino acid (aa) mutations in functional domains of the WNV envelope (E) protein to introduce two safety layers.

## Results

### Enriched WNV variants show ZAP-dependent *in vitro* infection phenotypes

To enrich dinucleotides in WNV RNA, we used established recoding principles and the SSE software [33,34]. During the enrichment process, we avoided cis-acting replication RNA elements. SSE employs algorithms that enable dinucleotide enrichment without altering the protein coding sequence and with minimal impact on codon usage parameters (S1 Table). The reference sequence used for CpG/UpA enrichment was the WNV NY99 strain [GenBank DQ211652.1], representing the 1999 introduction of WNV lineage one. Lineage one remains the predominant lineage in the US and globally [35,36], supporting the selection of NY99 for sequence enrichment.

**Fig 1:**
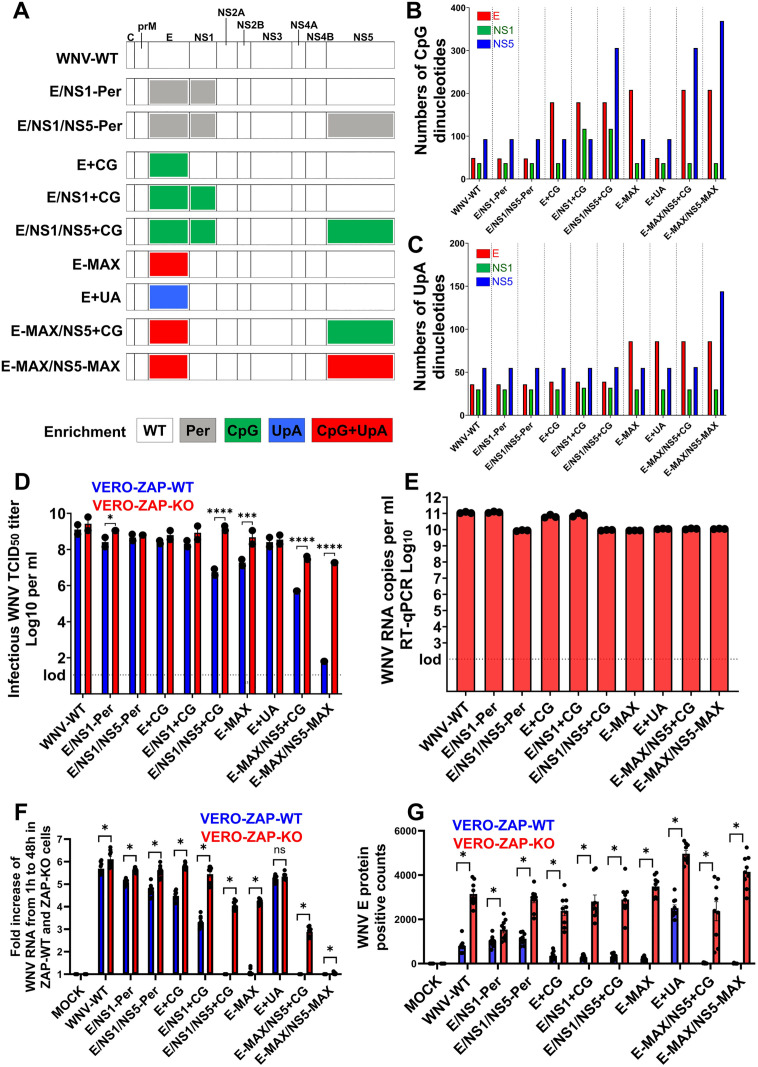
ZAP affects infection phenotypes of WNV variants. (A) Single WNV ORF representing wild-type (WT), permuted (Per), CpG-, UpA-, or CpG/UpA-enriched RNA regions. (B) The number of CpG dinucleotides in E, NS1, and NS5 genomic regions of all WNV variants. Data are also provided in S1 Table. (C) The number of UpA dinucleotides in E, NS1, and NS5 genomic regions of all WNV variants. Data are also in S1 Table. (D) Infectious titers of WNV variant stocks produced in ZAP-KO Vero cells and titrated in ZAP-WT or ZAP-KO Vero cells. lod: limit of detection. Two-way ANOVA was performed on log_10_-transformed infectious WNV TCID_50_ titers (*p ≤ 0.05, **p ≤ 0.01, ***p ≤ 0.001; ****p ≤ 0.001). (E) RNA loads in WNV variant stocks produced in ZAP-KO Vero cells. lod: limit of detection. (F) The fold increase of WNV variant RNA loads in supernatants collected at 1 h vs. 48 h after inoculation. ZAP-WT and ZAP-KO cells were inoculated with MOI 0.01. Virus inoculums were removed and replaced with media. Supernatants were collected for RNA extraction at 1 h post-inoculation (to normalize for leftover virus inoculum RNA) and at 48 h. *Unpaired t-test: *p* < 0.0001 for all variants except WNV-WT *p* = 0.0137, E+ UA *p* = 0.0947 (ns), and E-MAX/NS5-MAX *p* = 0.0053. The experiment was done in 3 biological and 2 technical replicates. (G) The digital quantification (counts per microscopic field; 3 random fields from 3 well replicates) of WNV E protein positive staining. Representative images of cells positive for WNV E are in Fig 3C and 3F. *Unpaired t-test: *p* < 0.0001 for all variants, except E/NS1-Per *p* = 0.0137.

We generated wild-type WNV (WNV-WT) and two permuted controls (E/NS1-Per and E/NS1/NS5-Per) (S1 Table and Fig 1A and S1 File). These permuted controls were designed to ensure that dinucleotide enrichment does not disrupt unknown cis-acting elements. The permutation strategy incorporates the maximum number of synonymous mutations within the target regions while preserving the wild-type mono- and dinucleotide frequencies and protein coding, with minimal possible effects on overall dinucleotide content and codon usage [29,33,34,37]. We also generated seven dinucleotide-enriched variants: E+ CG, E/NS1+ CG, E/NS1/NS5+ CG, E-MAX, E+ UA, E-MAX/NS5+ CG, and E-MAX/NS5-MAX (S1 Table and Fig 1A–C and S1 File). In E+ CG, E/NS1+ CG, and E/NS1/NS5+ CG, the UpA content was maintained at wild-type levels (S1 Table and Fig 1C and S1 File), which limited the number of CpGs that could be introduced without altering protein sequences. In contrast, for the E-MAX variant we maximized CpG content without normalizing for UpA levels, allowing both CpG and UpA frequencies to increase (S1 Table and Fig 1B and 1C and S1 File). For comparison, we also generated the E+ UA variant, in which CpG levels were maintained at wild-type levels, but UpA levels were increased to match those in E-MAX (S1 Table and Fig 1A–C and S1 File). Finally, the E-MAX/NS5+ CG and E-MAX/NS5-MAX variants contained the same CpG/UpA enrichment in the E region as E-MAX, along with either CpG-only or combined CpG/UpA enrichment in the NS5 region (S1 Table and Fig 1A–C and S1 File).

**Fig 2:**
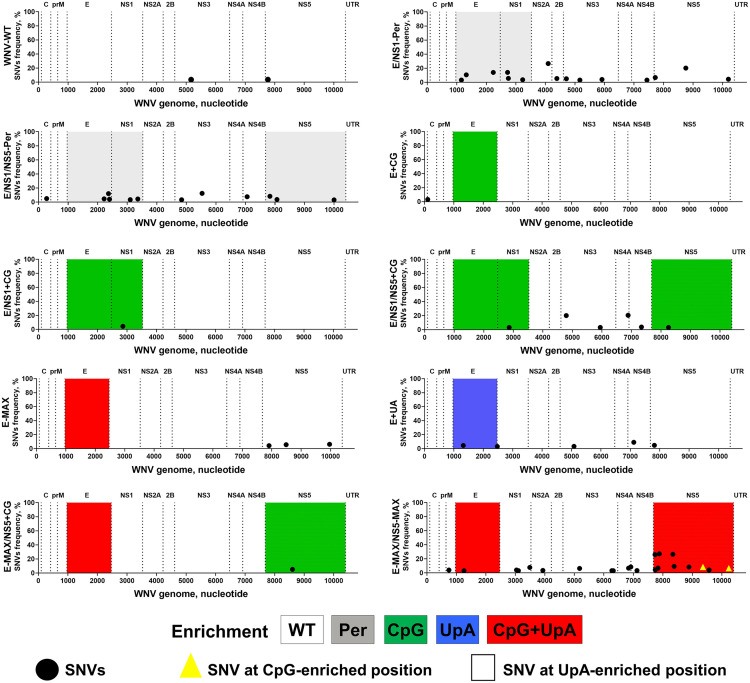
Stability of CpG/UpA enriched and endogenous dinucleotides in working WNV variant stocks. C: WNV genomic region encoding capsid protein; prM: precursor membrane protein; E: envelope protein; NS1: nonstructural protein 1; NS2A: nonstructural protein 2A; NS2B: nonstructural protein 2B; NS3: nonstructural protein 3; NS4A: nonstructural protein 4A; NS4B: nonstructural protein 4B; NS5: nonstructural protein 5. UTR: untranslated region. Highlighted E, NS1, and NS5 regions contain permuted or dinucleotide-enriched sequences (Fig 1A and S1 Table and S1 File). SNVs are also shown in S2 Table. Shannon entropy is shown in S6 File and S2 and S3 Tables. Raw FASTQ NGS files are deposited in BioProject: PRJNA1277320.

To rescue all WNV variants, we used the Infectious Subgenomic Amplicons (ISA) method [24,25,38–40] and ZAP-KO Vero cells [25,39,40]. Transfection of overlapping ISA DNA fragments for each WNV variant (S1 Table and S2 File) consistently rescued infectious viruses in all five well replicates. All variants were passaged and grown in ZAP-KO cells to generate working stocks. The consensus sequences of all WNV working stocks were verified by next-generation sequencing (NGS) (Fig 2 and S2 Table). We identified single nucleotide variants (SNVs) by aligning the NGS sequences of the viral stocks to the reference sequences used to generate the ISA fragments (S1 File). WNV-WT had only two SNVs, while substantially higher numbers of SNVs were found in the two permuted controls (Fig 2). Between one and seven low-frequency SNVs were identified in the other CpG/UpA-enriched variants. Shannon entropy was comparable between all variants (S6 File and S2 and S3 Tables). The only exception was the E-MAX/NS5-MAX variant, which contained 24 SNVs, including two affecting CpG-enriched positions (Fig 2). Shannon entropy also increased in the 5′-end–enriched NS5 region of the E-MAX/NS5-MAX variant (S6 File and S2 and S3 Tables). All other WNV variants retained the enriched and native CpG/UpA dinucleotides without changes.

Stock TCID_50_ titers were quantified by endpoint dilution assay in both ZAP-KO and ZAP-WT cells (Fig 1D). The two permuted control WNV stocks showed infectious titers comparable to WNV-WT in both cell lines, confirming that dinucleotide permutation in the selected genomic regions did not disrupt essential RNA structures or sequences. In contrast, variants with the highest number of enriched dinucleotides—E/NS1/NS5+ CG, E-MAX, E-MAX/NS5+ CG, and E-MAX/NS5-MAX (S1 Table)—exhibited substantially lower titers than WNV-WT when titrated in ZAP-WT cells (Fig 1D). These differences were less pronounced in ZAP-KO cells. Accordingly, the viral RNA copy-to-infectious titer ratios of all WNV variant stocks were lower when infectious titers were determined in ZAP-KO cells compared to ZAP-WT cells (S3 File). All WNV variants rescued in ZAP-KO cells also showed high viral RNA loads (Fig 1E), ranging from 10^9.9^ to 10^11^ viral RNA copies per ml.

A comparison of infectious titers and RNA loads in WNV stocks (Fig 1D and 1E) was performed after four days of growth in ZAP-KO cells. To assess how CpG/UpA enrichment affects early infection phenotypes, we inoculated ZAP-WT and ZAP-KO cells with WNV variants at multiplicity of infection (MOI) of 0.01 and measured RNA fold change between 1 h and 48 h post-inoculation (Fig 1F). We also compared WNV E protein expression across variants under the same experimental conditions (Fig 1G). All WNV variants, except E+ UA, showed statistically significant suppression of RNA loads in ZAP-WT cells compared to ZAP-KO cells (Fig 1F). The ZAP-dependent phenotype was particularly pronounced in E/NS1/NS5+ CG, E-MAX, and E-MAX/NS5+ CG (Fig 1F). An even more prominent ZAP-dependent pattern was observed when quantifying WNV E protein-positive staining (Fig 1G). All WNV variants exhibited significantly reduced E-positive cell counts in ZAP-WT cells relative to ZAP-KO cells. This effect was especially strong for E+ CG, E/NS1+ CG, E/NS1/NS5+ CG, E-MAX, E-MAX/NS5+ CG, and E-MAX/NS5-MAX (Fig 1G).

Zinc finger CCCH-type antiviral protein 1 is a host protein with broad antiviral activity [40–45]. ZAP binds CpG-rich or CpG-enriched viral RNA and targets it for exosome-mediated degradation [41]. This motivated us to initially use ZAP knockout cell lines for ISA rescue of all WNV variants (S1 Table). Here, we conducted comparative ISA studies to rescue E-MAX and E-MAX/NS5+ CG variants in both ZAP-WT and ZAP-KO cell lines, to determine how ZAP influences ISA efficiency for CpG/UpA-enriched WNV variants. These two variants were selected based on their pronounced reduction of infection in ZAP-WT cells (Fig 1D, 1F, and 1G). Infectious-subgenomic amplicons for each variant were prepared in parallel, and DNA amplicons used for transfection into ZAP-WT or ZAP-KO cells originated from the same PCR reactions.

**Fig 3:**
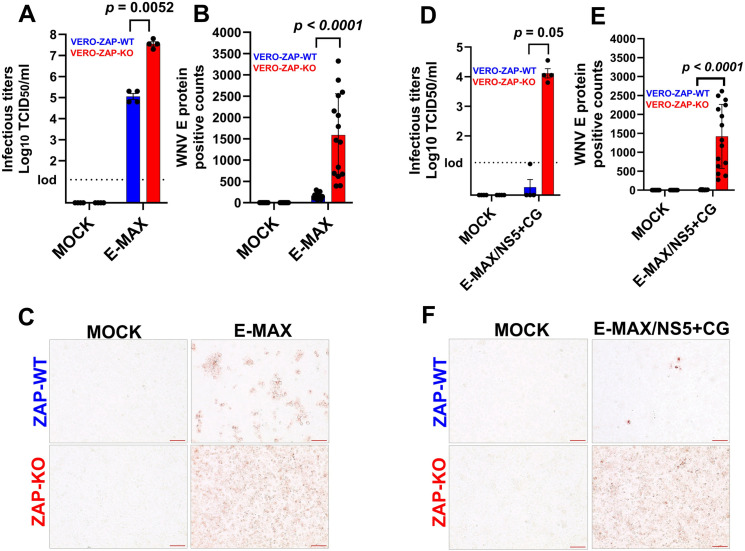
Comparative ISA of CpG/UpA-enriched WNV variants in ZAP-WT and ZAP-KO cells. Infectious TCID_50_ titers of the E-MAX (**A**) and E-MAX/NS5+ CG (**D**) in supernatants from ISA-transfected ZAP-WT and ZAP-KO VERO cells, collected at day 2 post-transfection during passage 1 of comparative ISA. The supernatants from each day were titrated in ZAP-WT cells. lod: limit of detection. Data from 4-well replicates. *p*: unpaired t-test. The digital quantification (counts per microscopic field) of E-MAX (**B**) and E-MAX/NS5+ CG **(E)** WNV E protein-positive counts in 5-well replicates (3 random fields in each well) at day 2 during passage 1 of comparative ISA. For technical replicates, three random images were obtained from each well. Representative images of E-MAX (**C**) and E-MAX/NS5+ CG **(F)** E protein IHC. Magnification x200. Red bar: 100 µm.

Infectious titers of E-MAX transfected and rescued in ZAP-WT cells (on average 10^5.05^ TCID_50_/ml) were significantly lower than those rescued in ZAP-KO cells (10^7.55^ TCID_50_/ml) under identical experimental conditions (Fig 3A). Correspondingly, E protein expression was considerably reduced in transfected ZAP-WT cells compared to ZAP-KO cells (Fig 3B and 3C). Infectious titers of E-MAX/NS5+ CG transfected and rescued in ZAP-WT cells were below the detection limit in three replicates, with only one replicate yielding very low titers at the detection threshold (Fig 3D). In contrast, infectious titers of E-MAX/NS5+ CG rescued in ZAP-KO cells consistently reached 10^3.8^ to 10^4.0^ TCID_50_/ml titers across all four replicates (Fig 3D). Similarly, E protein expression was considerably lower in ZAP-WT cells compared to ZAP-KO cells (Fig 3E and 3F). All E-MAX/NS5+ CG replicates in ZAP-WT cells displayed a small number of dispersed cells positive for WNV E protein (Fig 3F), including replicates that did not exhibit detectable infectious titers in supernatants (Fig 3D).

Altogether, using rational CpG and UpA dinucleotide enrichment, ISA reverse genetics, and transfection in ZAP-KO Vero cells, we generated WNV variants capable of producing high-titer stocks for *in vitro* and *in vivo* studies. We also demonstrated that CpG- and CpG/UpA-enriched WNV variants—but not UpA-enriched WNV—exhibit prominent ZAP-dependent reduction of infection in ZAP-WT Vero cells.

### Dinucleotide-enriched WNV variants are attenuated and protective in mice

**Fig 4:**
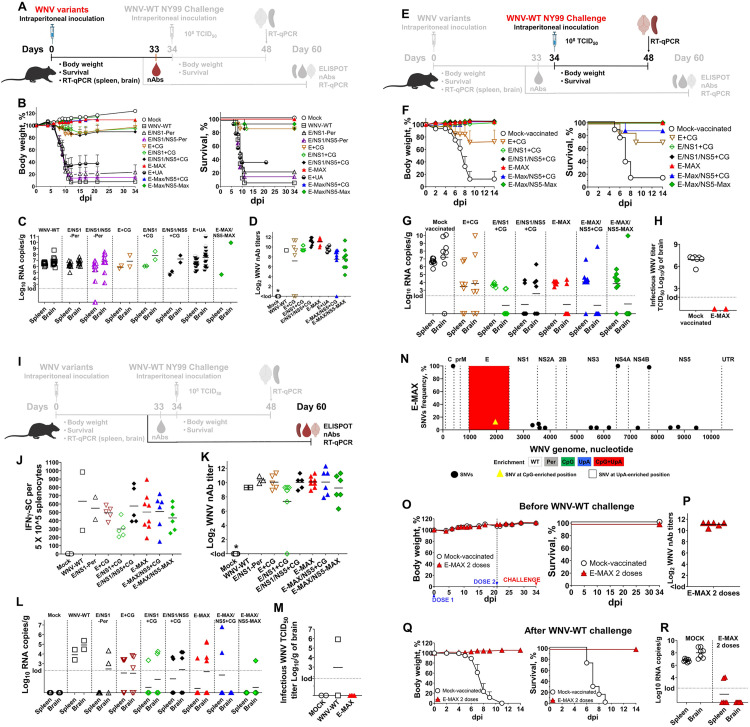
Infection phenotypes of WNV variants in mice. Dim and bold lines/text in panels **A**, **E**, and **I** outline the overall experimental design of the Fig 4 IP study. The bold lines and text in these panels highlight the specific experimental setup corresponding to the data presented in the associated four graph panels shown below each **A**, **E**, and **I**. **(A)** Animal IP trial timeline for infection phenotypes of WNV variants. On day 0, mice were inoculated IP with WNV-WT, permuted control, or dinucleotide-enriched variants (with doses normalized to 10^7^ RNA copies/mouse; 10^7^ RNA copies/mouse of WNV-WT corresponds to 10^5^ TCID_50_/mouse, see S3 File). 14 mice/group; 7 males and 7 females. (B) Body weight and survival in mice; a standard error of the mean (SEM) is shown. (C) WNV RNA loads in tissues of diseased or euthanized moribund mice in WNV-WT and Permuted control groups, determined by RT-qPCR. Brains and spleen from diseased mice in E+ CG (2 out of 14 mice), E/NS1+ CG (2/14), E/NS1/NS5+ CG (2/14), and E-MAX/NS5-MAX (1/14) groups, were also tested in WNV-specific RT-qPCR. In the E+ UA group, tissues from all diseased (9/14) and survived mice were tested in RT-qPCR. lod: limit of detection. **(D)** Neutralizing Ab (nAb) responses in mice at 33 days after IP injection with WNV variants. < lod: below the limit of detection. **(E-H)** Protection evoked by dinucleotide-enriched WNV variants after single IP injection. **(E)** Animal IP trial timeline to assess the protection evoked by enriched variants (6-8 immunized mice from **A**). On day 34 after IP injection with dinucleotide-enriched WNV variants, mice were IP challenged with the high (10^8^ TCID_50_/mouse) infectious doses of WNV-WT. **(F)** Body weight and survival in mice within 14 days after IP injection. In body weight, SEM is shown. **(G)** WNV RNA loads in tissues of mice diseased after challenge, or in tissues of survived mice euthanized on day 14 after challenge. lod: limit of detection. **(H)** Brains positive for WNV RNA by RT-qPCR (in **G**) from Mock-immunized and E-MAX-immunized (we focused on E-MAX because it is the most promising vaccine candidate) and challenged mice were tested for live WNV using the endpoint dilution assay in ZAP-KO Vero cells. lod: limit of detection. **(I-M)** Long-term (60 days) immunogenicity of WNV variants in mice after single-dose IP injection. **(I)** Animal IP trial timeline for long-term immunogenicity (5-8 immunized mice from **A**). **(J)** The number of IFN*γ*-secreting splenocytes was determined by the ELISpot assay. **(K)** nAb responses in mice on day 60 after IP injection with WNV variants. < lod: below the limit of detection. **(L)** WNV RNA loads in tissues of mice on day 60 after IP injection. lod: limit of detection. **(M)** Brains positive for WNV RNA by RT-qPCR (in **L**) from E-MAX-immunized mice (we focused on E-MAX because it is the most promising vaccine candidate) and from survived mice injected with WMV-WT were tested for infectious WNV using the endpoint dilution assay in ZAP-KO Vero cells. lod: limit of detection. **(N)** RNA samples from mouse brains positive for E-MAX were used for targeted PrimalSeq NGS. Brain samples from two mice (W.EU.F42.2 and **W.**EU.M43.1, S2 and S3 Tables) injected IP with E-MAX that showed no clinical signs for 60 days post-injection but tested positive by RT-PCR (Fig 4L; two E-MAX mice with the highest PCR values were tested). WNV SNVs in mouse **W.**EU.F42.2 brain are shown. SNVs for **W.**EU.M43.1 mouse which did not have affected CpGs/UpAs are areshown in S2 and S3 Tables. C: WNV genomic region encoding capsid protein; prM: precursor membrane protein; E: envelope protein; NS1: nonstructural protein 1; NS2A: nonstructural protein 2A; NS2B: nonstructural protein 2B; NS3: nonstructural protein 3; NS4A: nonstructural protein 4A; NS4B: nonstructural protein 4B; NS5: nonstructural protein 5. UTR: untranslated region. Highlighted E region contains dinucleotide-enriched sequences (Fig 1A and S1 Table and S1 File). SNVs are also shown in S2 Table. Shannon entroy data for this dataset are in S2 and S3 Tables and S6 File. Raw FASTQ NGS files are deposited in BioProject: PRJNA1310643. **(O)** Protection against lethal challenge after two injections with E-MAX. Body weight after 1^st^ and 2^nd^ IP injections with media (Mock-vaccination) or E-MAX (with doses normalized to 10^7^ RNA copies/mouse; 10^7^ RNA copies/mouse of WNV-WT is equal to 10^5^ TCID_50_/mouse, see S3 File). 6-7 mice/group. No clinical signs and 100% survival in mice after IP injections. **(P)** nAb titers at 33 days after the first E-MAX injection, corresponding to 11 days after the second E-MAX injection. < lod: below the limit of detection. **(Q)** Body weight and survival of mice after IP challenge with the 10^8^ TCID_50_/mouse of WNV-WT. The challenge was conducted 34 days after the first injection (corresponding to 12 days after the second injection). In body weight, SEM is shown. **(R)** WNV RNA loads determined by RT-qPCR in tissues from mice sampled at 14 days after challenge. lod: limit of detection. Portions of the figure are created in BioRender; Karniychuk, **V.** (2025) https://BioRender.com/7v7x5rj.

WNV neuroinvasion was assessed by monitoring neurological signs and mortality in wild-type C57BL/6J mice, a well-established model for neuroinvasive WNV disease [8,46–48]. C57BL/6J mice (14 mice per group) were injected intraperitoneally (IP) with a normalized dose of each WNV variant (S3 File) or media (Mock) (Fig 4A).

The Mock group exhibited no clinical signs (Fig 4B). In contrast, WNV-WT, E/NS1-Per and E/NS1/NS5-Per permuted control viruses caused severe clinical signs and high mortality—93%, 79%, and 86%, respectively—within 6–21 days post-injection (Fig 4B). Clinical signs included ataxia, depression, paralysis, weight loss exceeding 20%, and death. Despite substantial reduction of *in vitro* infection (Fig 1F and 1G), the E+ CG, E/NS1+ CG, and E/NS1/NS5+ CG variants resulted in 14% mortality (2 of 14 mice per group; Fig 4B), while the E-MAX/NS5-MAX variant caused 7% mortality (1 of 14 mice). These mice either developed paralysis (one E+ CG mouse), were found dead (one mouse each in E+ CG, E/NS1+ CG, and E/NS1/NS5+ CG groups), or reached the 20% body weight loss endpoint. Remarkably, the E-MAX and E-MAX/NS5+ CG variants did not cause clinical signs or mortality (Fig 4A and 4B). The E+ UA variant, which has the same UpA enrichment as E-MAX, resulted in 64% mortality (Fig 4A and 4B).

Tissues from deceased mice in the WNV-WT, E/NS1-Per, and E/NS1/NS5-Per groups, as well as from deceased and surviving mice in the E+ UA group, were analyzed using WNV-specific RT-qPCR. Brains from these mice had high WNV loads (Fig 4C). Additionally, one or two deceased mice from the E+ CG, E/NS1+ CG, E/NS1/NS5+ CG, and E-MAX/NS5-MAX groups also showed detectable viral loads in the brain (Fig 4C).

Next, we quantified neutralizing antibody (nAb) titers in a subset of mice (6–8 per group, Fig 4D). These mice were injected with variants causing either low (E+ CG, E/NS1+ CG, E/NS1/NS5+ CG, E-MAX/NS5-MAX; Fig 4B) or no mortality (E-MAX and E-MAX/NS5+ CG; Fig 4B). On day 33 after a single IP injection, mice showed high levels of WNV nAbs (Fig 4D), except for two mice in the E+ CG group and one mouse in the E-MAX/NS5+ CG group, which had titers below the detection limit (Fig 4D). Interestingly, mice in the E-MAX group, characterized by a highly attenuated infection phenotype with no mortality (Fig 4B), developed nAb responses with average titers higher than all other groups (except E/NS1/NS5+ CG), including the WNV-WT (available sample from one surviving mouse) and E+ UA groups. The same mice were challenged IP with a high infectious dose of WNV-WT (10^8^ TCID_50_/mouse; Fig 4E). Injection with CpG-enriched WNV vaccine candidates partially or fully protected mice from disease following IP challenge with a high infectious dose (10^8^ TCID_50_) of WNV-WT. Mock-vaccinated mice exhibited severe clinical signs and 88% mortality (Fig 4F). The E+ CG candidate protected 71% of mice; the two mice that succumbed to infection (Fig 4F) had nAb titers below the detection limit prior to WNV challenge (Fig 4D). The E-MAX/NS5+ CG candidate protected 88% of mice, with only one mouse succumbing to infection (Fig 4F). E-MAX, E/NS1+ CG, E/NS1/NS5+ CG, and E-MAX/NS5-MAX provided 100% protection against clinical disease and death following a single IP injection (Fig 4F). Using RT-qPCR assay, we detected WNV RNA in the spleen and brain tissues of mice in all groups 14 days after WNV-WT challenge (Fig 4G). We selected brains positive for WNV RNA from Mock-immunized and E-MAX-immunized mice (focusing on E-MAX as the most promising vaccine candidate) for virus isolation and titration in ZAP-KO Vero cells. High infectious WNV titers were detected in the brains of all Mock-immunized mice; in contrast, no infectious virus was detected from the PCR-positive brains of the two E-MAX-immunized mice (Fig 4H).

Another cohort of mice immunized with E+ CG, E/NS1+ CG, E/NS1/NS5+ CG, E-MAX, E-MAX/NS5+ CG, and E-MAX/NS5-MAX (5–8 mice per group; single IP injection), along with two surviving WNV-WT and E/NS1-Per mice from the initial IP study (Fig 4A and 4B), entered a 60-day immunogenicity study (Fig 4I). Mice immunized with dinucleotide-enriched WNV variants showed no apparent clinical signs during the 60-day period. All groups exhibited high numbers of IFNγ-secreting splenocytes as measured by ELISpot assay (Fig 4J). Additionally, all groups developed high nAb titers, except for one mouse in the E/NS1+ CG group (Fig 4K). RT-qPCR assay detected WNV RNA in spleens and brains of mice from all groups, including the E-MAX group (Fig 4L). Interestingly, sensitive PrimalSeq NGS, which provides high sequencing depth (10^5^-10^6^; S5 File), revealed that only one of the 159 enriched CpGs in E-MAX (S1 Table) was partially affected (Fig 4N) in a brain of asymptomatic mouse that remained positive for E-MAX RNA 60 days after IP injection (Fig 4L). The SNV affecting this CpG, however, was present at only 12.8% frequency (Fig 4N and S2 Table). All 50 enriched UpAs in E-MAX remained stable and were not affected by SNVs (Fig 4N and S2 and S3 Tables and S6 File). In a second mouse brain from the same group (Fig 4L), PrimalSeq NGS confirmed complete stability of all introduced CpGs and UpAs (S2 and S3 Tables and S6 File). Both mice also carried two to three synonymous or nonsynonymous high-frequency (98–100%) mutations and 12 low-frequency (3-12.8%) mutations each; none of these SNVs affected enriched or endogenous CpGs or UpAs (Fig 4N and S2 and S3 Tables). These SNVs did not affect E-MAX virulence, as two mice remained asymptomatic for 60 days. Brains from E-MAX-immunized mice (as E-MAX was the most promising vaccine candidate) and surviving mice injected with WNV-WT (n = 2) were further assessed for infectious virus in ZAP-KO Vero cells. Endpoint dilution assays showed high infectious WNV titers in the brain of one WNV-WT mouse. However, all brains from E-MAX-immunized mice (n = 4) were negative for infectious virus at 60 days after injection (Fig 4M).

The IP studies (Fig 4A, 4E, and 4I) indicated E-MAX as the most promising vaccine candidate, demonstrating no morbidity or mortality (Fig 4B), robust nAb (Fig 4D and 4K) and cellular (Fig 4J) responses, and protection against high-dose WNV-WT challenge (Fig 4F). However, despite lacking clinical signs, E-MAX-immunized mice challenged with WNV-WT (n = 7) had detectable WNV RNA in spleen (all mice) and brains (two mice; Fig 4G). Infectious WNV was not isolated from the brains of these two E-MAX-injected mice (Fig 4H), but the presence of viral RNA potentially indicated WNV-WT neuroinvasion post-challenge. Thus, to evaluate whether two E-MAX injections would fully prevent WNV neuroinvasion, we injected C57BL/6J mice (6–7 per group) twice with either media (Mock) or E-MAX, followed by an IP challenge with a high dose (10^8^ TCID_50_/mouse) of WNV-WT (Fig 4O). Two E-MAX injections did not cause clinical signs and mortality (Fig 4O), while elicited strong nAb responses in all six mice (Fig 4P). Similar to the single-dose study (Fig 4F), the two injections fully protected mice from clinical signs and mortality (Fig 4Q). Furthermore, RT-qPCR assay did not detect WNV RNA in the brains of these mice after the high-dose WNV-WT challenge (Fig 4R).

Altogether, all dinucleotide-enriched WNV variants exhibited attenuated infection phenotypes compared to WNV-WT and permuted controls following IP injection. The IP studies highlighted E-MAX as the most promising preclinical vaccine candidate, demonstrating attenuated infection, strong immune responses, and protection against lethal challenge. Thus, E-MAX was selected to further assess the stability of enriched CpG/UpA dinucleotides, examine effects on cellular innate transcriptional responses, and evaluate footpad (intradermal/subcutaneous) immunization.

### Enriched CpG/UpA dinucleotides are stable in the E-MAX genome after serial passaging in VERO-ZAP-KO cells

**Fig 5:**
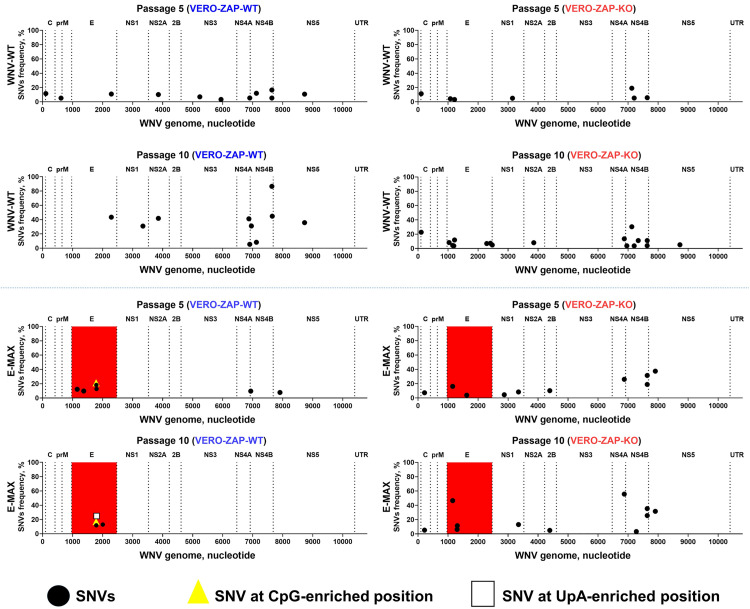
Stability of CpG/UpA enriched and endogenous dinucleotides in the E-MAX variant after serial passaging in VERO-ZAP-WT and VERO-ZAP-KO cells. C: WNV genomic region encoding capsid protein; prM: precursor membrane protein; E: envelope protein; NS1: nonstructural protein 1; NS2A: nonstructural protein 2A; NS2B: nonstructural protein 2B; NS3: nonstructural protein 3; NS4A: nonstructural protein 4A; NS4B: nonstructural protein 4B; NS5: nonstructural protein 5. UTR: untranslated region. Highlighted in red E region contains CpG+UpA dinucleotide-enriched sequences (Fig 1A and S1 Table and S1 File). SNVs are also shown in S2 Table. Shannon entropy data for this dataset are in S2 and S3 Tables and S6 File. Raw FASTQ NGS files are deposited in BioProject: PRJNA1277320.

To determine the stability of enriched CpG/UpA content in the context of the E-MAX genome, we passaged E-MAX variant ten times in VERO-ZAP-WT and VERO-ZAP-KO cells. For comparison, we also passaged WNV-WT under identical conditions. Initially, both WNV-WT and E-MAX variants had 2–3 low-frequency SNVs (Fig 2 and S2 Table). After serial passaging in ZAP-WT cells, E-MAX exhibited fewer SNVs than WNV-WT (4–8 SNVs vs. 10–11 SNVs, respectively; Fig 5). However, the number of SNVs, specifically in the genomic E region, was higher for E-MAX than for WNV-WT (4–6 SNVs vs. 1 SNV; Fig 5). E-MAX SNVs affected one CpG-enriched position (one out of the 159 enriched CpGs in E-MAX; no endogenous CpGs were affected) at passage 5, and one CpG- and one UpA-enriched position (one out of the 50 enriched UpAs in E-MAX; no endogenous UpAs were affected) at passage 10 (Fig 5). Shannon entropy also increased in the same RNA region of the E-MAX variant (Fig 5 and S6 File and S2 and S3 Tables). However, these SNVs reached frequencies of 17–21% and, therefore, did not completely replace the enriched dinucleotides (Fig 5). Furthermore, the frequency of the mutation C1782G, which affected enriched CpG, decreased from 21% at passage 5–17% at passage 10 (Fig 5 and S2 Table), indicating that the CpG enrichment at that site remains relatively stable in ZAP-WT cells, and the mutation did not confer a strong replication advantage.

Both total and E-region-specific SNVs were comparable for WNV-WT and E-MAX variants after passaging in ZAP-KO cells (Fig 5). WNV-WT had 8–19 total SNVs compared to 11–12 for E-MAX. In the genomic E region, WNV-WT and E-MAX variants had 2–5 and 2–3 SNVs, respectively. Unlike in ZAP-WT cells, serial passages in ZAP-KO cells did not affect the enriched or endogenous CpG and UpA dinucleotides in the E-MAX genome (Fig 5 and S2 Table).

### Attenuated E-MAX induces innate immune responses in a human cell line

**Fig 6:**
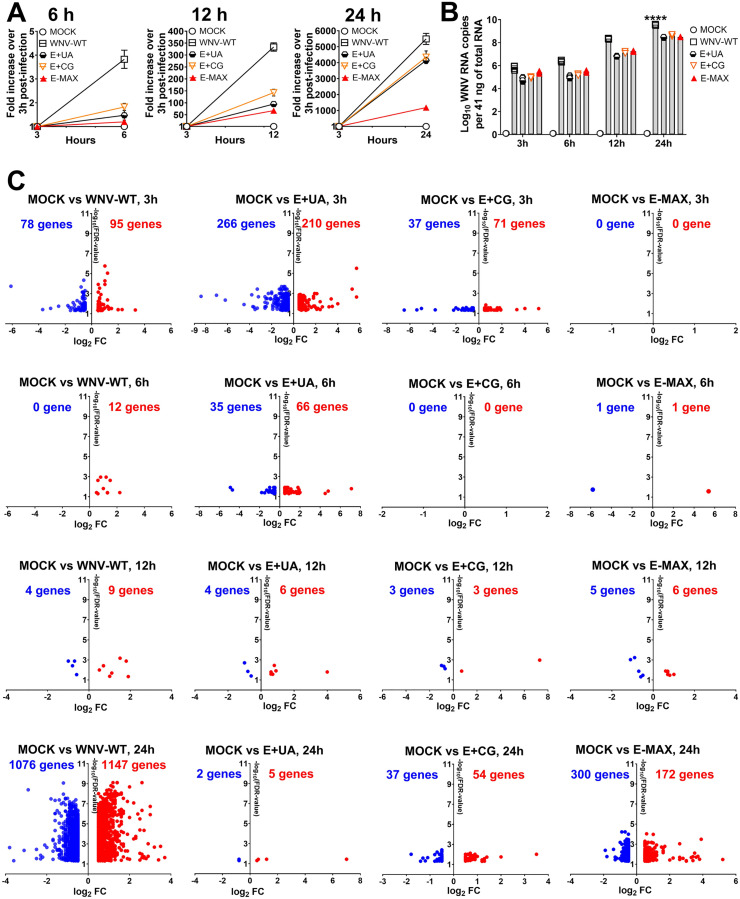
Viral RNA kinetics and significantly affected genes during infection caused by WNV variants in human Huh7 cells. Intracellular WNV RNA fold change over 3 h post-infection **(A)** and log_10_ RNA copies per 41 ng of total RNA **(B)** after synchronized infection with viruses at a concentration of 1,000 RNA genome copies per cell. WNV RNA copies were calculated using RT-qPCR targeting UTR that was unmodified in all variants. Results are the mean and standard error of four biological replicates. ****p < 0.0001; WNV-WT had a significantly higher number of absolute viral RNA copies than E+ UA, E+ CG, and E-MAX at 24 h after inoculation; one-way ANOVA. The total RNA from the same Huh7 cell replicates were used for RNA-seq **(C)**. Upregulated (red) and downregulated (blue) genes. Direction of analysis: WNV variant/MOCK. Genes with FDR < 0.05 and log_2_ fold change (FC) ≥ 0.5 (1.42-fold) are shown. FDR: false discovery rate. Raw RNA-seq data are in S4 Table. Raw FASTQ NGS files are deposited in BioProject: PRJNA1281688.

To assess whether E-MAX induces transcriptional responses despite its reduced infection and attenuated phenotype (Figs 1F, 1G, 3A–C, 4A, 4B, 4O, and 9), human Huh7 cells were synchronously inoculated with 1,000 viral RNA genome copies per cell, as determined by RT-qPCR targeting the UTR that was unmodified in all WNV variants. We aimed to examine the early innate responses elicited by dinucleotide-enriched RNA during infection; thus, inoculums were normalized by viral RNA copies. E-MAX has both CpG and UpA enrichment in the genomic E region; for comparison, cells were also inoculated with WNV-WT, E+ CG, and E+ UA variants, which have wild-type dinucleotide content or single CpG or UpA enrichment, respectively, in the same genomic region (Fig 1A and S1 Table and Fig 6).

**Fig 7:**
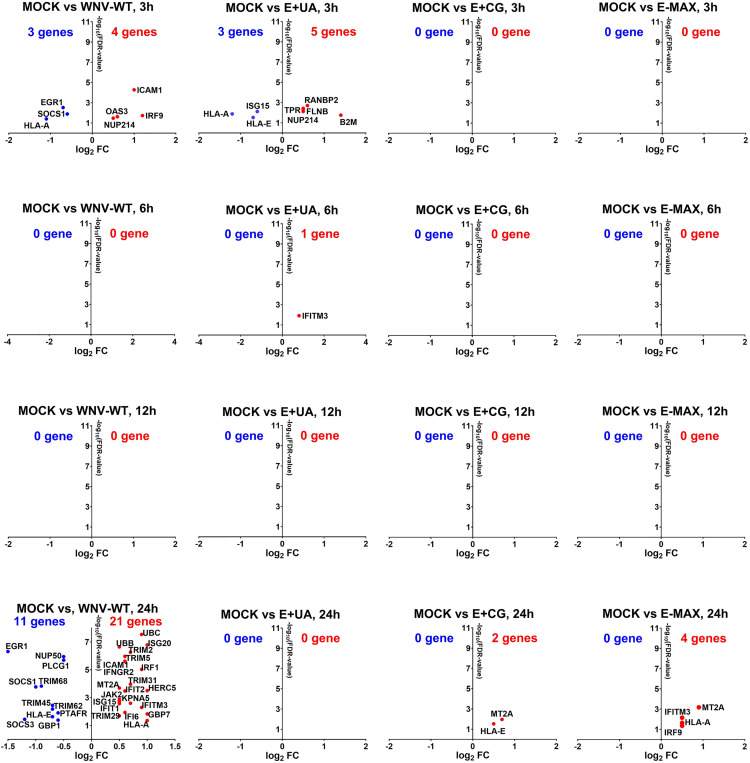
Significantly affected interferon signaling genes during infection caused by WNV variants in Huh7 cells. The total RNA from Huh7 cells infected with different WNV variants and sampled at 3, 6, 12, and 24 h (from Fig 6A and 6B) were used for RNA-seq. Upregulated (red) and downregulated (blue) genes are shown. Direction of analysis: WNV variant/MOCK. Genes with FDR < 0.05 and log_2_ fold change (FC) ≥ 0.5 (1.42-fold) are shown. FDR: false discovery rate. Raw FASTQ NGS files are deposited in BioProject: PRJNA1281688.

**Fig 8:**
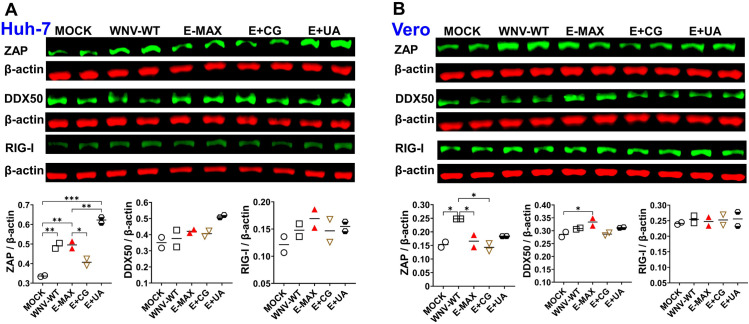
The expression of ZAP, DDX50, RIG-I during infection caused by wild-type and dinucleotide-enriched WNV variants. Human Huh-7 and monkey VERO-ZAP-WT cells were synchronously inoculated with 1,000 viral RNA genome copies/cell of WNV-WT, E-MAX, E+ CG, E+ UA, or mock. Cells were washed and lysed at 6 h post-inoculation for Western blot. Green bands indicate proteins of interest: ZAP (100 kDa), DDX50 (83 kDa), and RIG-I (107 kDa). Red bands represent β-actin loading control (42 kDa). Western blot was performed in biological duplicate for each experimental condition. Protein expression levels were quantified using ChemiDoc MP Imaging system and Image Lab 6.1 Software. Protein loading was standardized (25 µg total protein per sample for RIG-I; 50 µg for ZAP and DDX50). Quantitative data are shown as densitometric quantification relative to *β*-actin loading control. Data are presented as scatter dot plots with means. Statistical analyses were performed by one-way ANOVA using GraphPad Prism software version 10.3.1 (*p ≤ 0.05, **p ≤ 0.01, ***p ≤ 0.001). Raw Western blot images are in S4 File.

At 3, 6, 12, and 24 h post-inoculation, intracellular viral genomes were quantified using RT-qPCR targeting the unmodified UTR present in all WNV variants. As anticipated, the WNV-WT variant accumulated intracellular RNA more rapidly within 24 h compared to the dinucleotide-enriched variants (Fig 6A). Consequently, WNV-WT RNA copies were significantly higher than those of all three enriched variants at 24 h; at earlier time points (3, 6, and 12 h), differences were not statistically significant, but WNV-WT RNA levels were still 10^0.4^-10^1.5^ higher (Fig 6B). Differences between E+ UA, E+ CG, and E-MAX RNA levels were not statistically significant (Fig 6B).

Global host mRNA expression was evaluated by RNA-seq at 3, 6, 12, and 24 h post-inoculation relative to mock-infected cells at corresponding time points (S4 Table). The WNV-WT and E+ UA variants, both displaying aggressive infection phenotypes *in vivo* (Fig 4B), triggered similar transcriptional activation patterns between 3–12 h post-inoculation. At 3 h, both variants altered the expression of numerous genes (Fig 6C). Notably, the E+ UA variant impacted 2–3 times more genes than the WNV-WT variant (210–266 vs. 78–95 genes), potentially reflecting host responses to elevated UpA content. Gene expression changes sharply declined at 6 and 12 h for both variants. However, at 24 h, WNV-WT infection significantly altered expression levels for thousands of genes, whereas only 2–5 genes were differentially expressed in E+ UA-infected cells (Fig 6C).

The partially attenuated E+ CG variant (Figs 1F, 1G and 4B) impacted gene expression at 3h (37–71 affected genes), though less extensively than WNV-WT (78–95 genes) and E+ UA (266–210 genes; Fig 6C). Similar to other variants, E+ CG-infected cells showed considerable or complete reduction of differentially expressed genes at 6–12 h compared to the 3 h time point. At 24 h, E+ CG-infected cells showed moderate gene expression changes, with 37–54 affected genes—fewer than WNV-WT-infected cells but more than E+ UA-infected cells (Fig 6C).

The promising vaccine candidate E-MAX displayed a unique gene expression profile. Very few genes (0–11) were affected at early time points (3–12 h), while at 24 h post-inoculation, a substantial number of genes (300 downregulated and 172 upregulated) showed differential expression (Fig 6C).

Significantly affected genes from each WNV variant and timepoint were further analyzed by comparing these differentially expressed genes (DEGs; Fig 6 and S4 Table) to a set of 194 genes involved in the interferon signaling pathway (S4 Table). DEGs significantly affected in each condition and related to interferon signaling were summarized in S4 Table and visualized in Fig 7 for pairwise comparison.

Consistent with global gene expression patterns (Fig 6C), WNV-WT and E+ UA variants induced similar numbers of affected genes related to IFN signaling (3–5 genes) at 3 h post-inoculation (Fig 7). At 6 h and 12 h, cells infected with either WNV-WT or E+ UA showed either no DEGs or only a single DEG related to interferon signaling. At 24 h, E+ UA infection resulted in no DEGs related to interferon signaling, whereas WNV-WT induced significant under- and overexpression of 11 and 21 genes, respectively (Fig 7).

Although Huh7 cells infected with all three dinucleotide-enriched WNV variants had similar viral RNA copy numbers at 24 h (Fig 6B), in contrast to E+ UA, both E+ CG and E-MAX variants induced the overexpression of 2 and 4 interferon signaling genes, respectively, at 24 h (Fig 7). Interestingly, unlike WNV-WT, which downregulated significantly 11 DEGs related to IFN signaling, neither E+ CG nor E-MAX significantly downregulated the expression of interferon signaling genes.

To further evaluate innate immune responses induced by WNV variants with varying CpG and UpA content, we quantified protein levels of ZAP, DDX50, and RIG-I during early infection in human Huh7 and wild-type Vero cells (Fig 8). These innate markers were selected because ZAP binds CpG-rich or CpG-enriched viral RNA, targets it for exosome-mediated degradation, and attenuates infection phenotypes [27,28,41]. Additionally, ZAP is an interferon (IFN)-stimulated gene, and potentially enhancing type I IFN antiviral responses [41,44,49–54]. DDX50, a recently identified viral restriction factor, inhibits dengue virus replication [55,56], and our recent study suggests it may act as co-factor for ZAP [40]. RIG-I is crucial for innate immunity against flaviviruses, including WNV [57], and synergistic antiviral activity between ZAP and RIG-I has been proposed [51].

Infection with WNV-WT, E-MAX, E+ CG, and E+ UA significantly increased ZAP expression in Huh7 cells (Fig 8A), whereas in Vero cells, only WNV-WT induced a significant increase (Fig 8B). No statistically significant changes in DDX50 expression were observed in Huh7 or Vero cells infected by variants, except for a significant increase by E-MAX in Vero cells (Fig 8B). Similarly, no significant changes in RIG-I expression were observed in either cell line across all WNV variants, although Huh7 cells infected with enriched variants, particularly E+ UA and E-MAX, showed a trend toward increased RIG-I expression (Fig 8A).

### E-MAX one-dose footpad immunization evokes protection against lethal challenge in mice and has a dose-dependent neuroinvasion phenotype

After evaluating infection and immune phenotypes using IP injection, we selected E-MAX as the most promising vaccine candidate and conducted a series of safety, immunogenicity, and protection studies using footpad injection. Footpad injection delivers the inoculum subcutaneously/intradermally representing a potential vaccine administration route.

**Fig 9:**
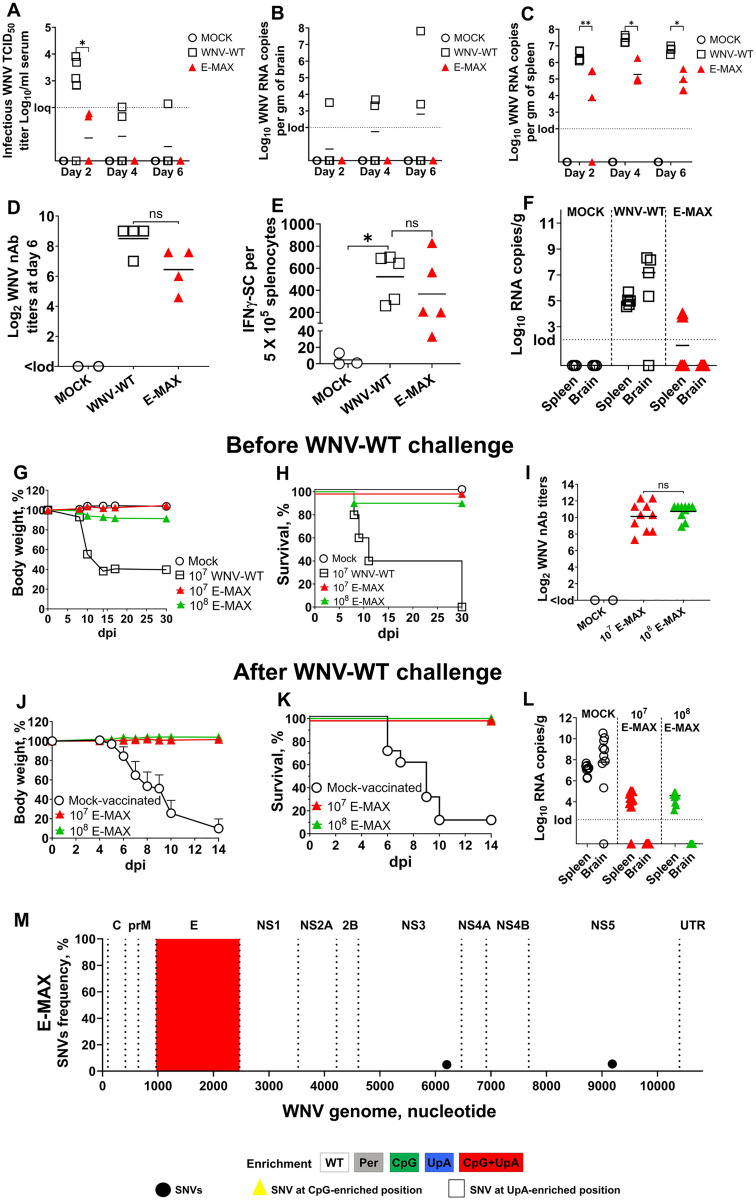
Neuroinvasion, early immune responses, and protection against lethal challenge after footpad immunization with E-MAX. **(A-D)** C57BL/6J mice were injected (one dose) into the footpad with MOCK (media) or E-MAX (with doses normalized to 10^7^ RNA copies/mouse; 10^7^ RNA copies/mouse of WNV-WT is equivalent to 10^5^ TCID_50_/mouse, see S3 File). 4-5 mice per group at each time point. **(A)** Viremia quantified by the end point dilution assay. WNV RNA loads were determined by RT-qPCR in the brain (**B**) and spleen **(C)**. **(D)** Neutralizing antibody titers on day 6 after E-MAX immunization. **(E, F)** C57BL/6J mice were injected into the footpad with MOCK (media) or E-MAX (with doses normalized to 10^5^ RNA copies/mouse; 10^5^ RNA copies/mouse of WNV-WT is equivalent to 10^3^ TCID_50_/mouse dose, see S3 File). On day 10 after injection, we quantified the number of IFNγ-secreting splenocytes by the ELISpot assay **(E)** and WNV RNA loads by RT-qPCR in the brain and spleen **(F)**. Body weight **(G)** and survival **(H)** after footpad immunization with media (MOCK), WNV-WT (10^7^ RNA WNV copies/mouse), or E-MAX (10^7^ or 10^8^ RNA WNV copies/mouse; see S3 File for infectious dose equivalents). 10 mice per group. **(I)** Neutralizing antibody titers on day 28 after E-MAX immunization. **(J)** Body weight and **(K)** survival of E-MAX-immunized mice after IP challenge with the 10^8^ TCID_50_/mouse of WNV-WT. The challenge was conducted on day 30 after single-dose footpad immunization. In body weight, a standard error of the mean (SEM) is shown. **(L)** WNV RNA loads determined by RT-qPCR in tissues from mice immunized, challenged, and sampled 14 days after the challenge. lod: limit of detection. loq: limit of quantification. *p ≤ 0.05, **p ≤ 0.01, ns: not significant by two-way ANOVA in A, B, C and one-way ANOVA in D, E, and **I. (M)** WNV SNVs in mouse E.m.M77.4 brain tissue. An RNA sample from an E.m.M77.4 mouse (Fig 9H and S2 and S3 Tables) brain positive for E-MAX after foodpad injection was used for PrimalSeq NGS. C: WNV genomic region encoding capsid protein; prM: precursor membrane protein; E: envelope protein; NS1: nonstructural protein 1; NS2A: nonstructural protein 2A; NS2B: nonstructural protein 2B; NS3: nonstructural protein 3; NS4A: nonstructural protein 4A; NS4B: nonstructural protein 4B; NS5: nonstructural protein 5. UTR: untranslated region. Highlighted E region contains dinucleotide-enriched sequences (Fig 1A and S1 Table and S1 File). SNVs are also shown in S2 Table. Shannon entropy is shown in S6 File and S2 and S3 Tables. Raw FASTQ NGS files are deposited in BioProject: PRJNA1310643.

First, we injected mice with MOCK, WNV-WT, and E-MAX to compare viremia, neuroinvasion, viral loads in the spleen, and early nAb responses. Mice were sampled on day 2, 4, and 6 after footpad injection. Unlike WNV-WT, which caused viremia from days 2–6, E-MAX resulted in only transient low-level viremia on day 2 (Fig 9A). In contrast to WNV-WT, which showed increasing WNV loads in the brain over time, E-MAX did not cause neuroinvasion and was undetectable in the brain by the RT-qPCR assay (Fig 9B). In the spleen, E-MAX viral loads were significantly lower than those of WNV-WT (Fig 9C). Despite its attenuated phenotype, E-MAX induced high nAb titers as early as 6 days post-injection (dpi), which were not significantly different from those induced by WNV-WT (Fig 9D).

Second, we injected mice with MOCK, WNV-WT, and E-MAX to compare early IFNγ responses in splenocytes. At day 10 post-immunization, both WNV-WT and E-MAX induced comparable numbers of IFNγ-secreting splenocytes (Fig 9E). Unlike WNV-WT, which showed high viral RNA loads in the spleen (all five mice) and brain (four out of five mice), E-MAX-injected mice exhibited lower RNA loads in the spleens of only two out of five mice, and all mice showed no viral RNA in brains at day 10 after footpad injection (Fig 9F).

Next, to further compare infection phenotypes after footpad injection and assess protection, we injected mice with MOCK, WNV-WT, and E-MAX (Fig 9G–L). For the WNV-WT infectious control, we used 10^7^ RNA copies/mouse (this corresponds to 10^5^ TCID_50_/mouse, titrated on VERO-ZAP-WT cells; 10^5.4^ TCID_50_/mouse, titrated on VERO-ZAP-KO cells; S3 File). For E-MAX, two different doses were tested—10^7^ RNA copies/mouse (10^4.4^ TCID_50_/mouse on VERO-ZAP-WT; 10^5.8^ TCID_50_/mouse on VERO-ZAP-KO; S3 File) and 10^8^ RNA copies/mouse (10^5.4^ TCID_50_/mouse on VERO-ZAP-WT; 10^6.8^ TCID_50_/mouse on VERO-ZAP-KO; S3 File). All mice in the WNV-WT group lost weight and reached the termination point within 11 days (two mice survived to day 11 but were found dead on day 30) (Fig 9G and 9H). In contrast, mice in the E-MAX groups did not exhibit clinical signs (Fig 9G and 9H) and showed high WNV nAb titers (Fig 9I). Unexpectedly, one mouse injected with 10^8^ RNA copies of E-MAX reached the termination point (score 4: weight loss, near-moribund but still somewhat responsive; see Materials and Methods for scoring) and was euthanized on day 8 (Fig 9G and 9H). The brain of this mouse contained high WNV RNA loads (10^10.29^ RNA copies/gram). Interestingly, PrimalSeq NGS detected only two low-frequency SNVs (4.9-5.4%) that did not affect any of the 159 enriched CpGs and 50 enriched UpAs or endogenous CpGs/UpAs in the E-MAX genome (Fig 9M and S2 and S3 Tables and S6 File).

MOCK- and E-MAX-vaccinated mice were challenged intraperitoneally with 10^8^ TCID_50_/mouse of WNV-WT on day 30 after immunization. A single footpad immunization with either 10^7^ or 10^8^ RNA copies/mouse of E-MAX provided full protection from clinical signs and mortality (Fig 9J and 9K). Furthermore, WNV RNA was undetectable in the brains of vaccinated mice, as assessed by RT-qPCR on day 14 after challenge (Fig 9L).

Altogether, the E-MAX variant demonstrated a promising safety profile in footpad injection studies at a dose of 10^7^ RNA copies/mouse (Fig 9A, 9B, 9G, and 9H). Immunization with 10^8^ RNA copies/mouse (10^5.4^ TCID_50_/mouse on VERO-ZAP-WT; 10^6.8^ TCID_50_/mouse on VERO-ZAP-KO; S3 File)—a dose higher than that used for WNV-WT (10^5^ TCID_50_/mouse on VERO-ZAP-WT; 10^5.4^ TCID_50_/mouse on VERO-ZAP-KO)—resulted in death of one mouse (10% mortality vs. 100% mortality in the WNV-WT group; Fig 9G and 9H). E-MAX immunization at both doses elicited robust humoral and cellular immune responses (Fig 9D, 9E, and 9I) and conferred complete protection against disease and neuroinvasion following lethal WNV-WT challenge (Fig 9J–L).

### A combination of CpG/UpA enrichment and L107F and K440R amino acid substitutions in the genomic region encoding the E protein attenuates aggressive WNV infection enabling protection against lethal challenge

**Fig 10:**
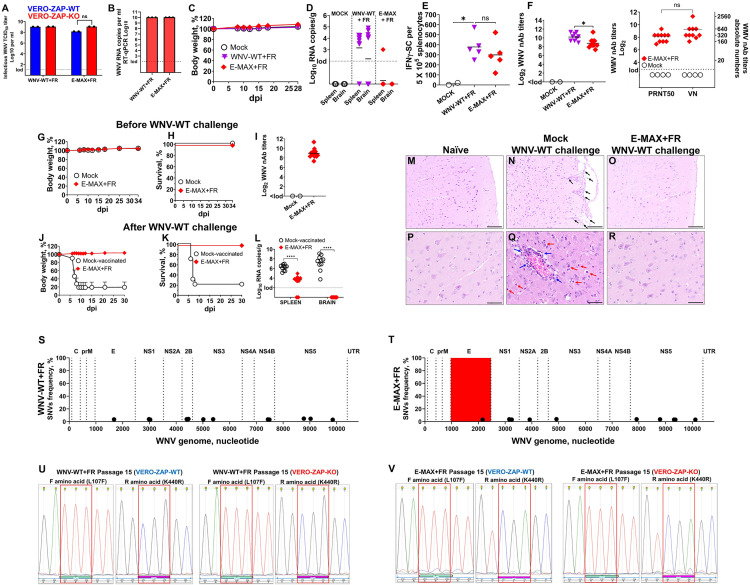
*In vitro* and *in vivo* infection phenotypes, immunogenicity and protection against lethal challenge after footpad immunization with the CG/UA-enriched and FR-modified variants. **(A)** Infectious titers of WNV variant stocks produced in ZAP-KO Vero cells and titrated in ZAP-WT or ZAP-KO Vero cells. lod: limit of detection. ns: Unpaired t-test, *p* = 0.33. **(B)** RNA loads in WNV variant stocks produced in ZAP-KO Vero cells. lod: limit of detection. **(C-F)** C57BL/6J mice were footpad inoculated with 10^8^ RNA copies/mouse of WNV-WT+ FR (10^7^ TCID_50_/mouse, titration on VERO-ZAP-WT cells; 10^7^ TCID_50_/mouse, titration on VERO-ZAP-KO cells; S3 File) or 10^8^ RNA copies/mouse E-MAX+ FR variant (10^6^ TCID_50_/mouse, titration on VERO-ZAP-WT cells; 10^7^ TCID_50_/mouse, titration on VERO-ZAP-KO cells; S3 File). 10 mice per group. All mice remained asymptomatic **(C)** and were euthanized on day 28 post-immunization. lod: the limit of detection. **(D)** WNV RNA loads were determined by RT-qPCR in the spleen and brain. lod: limit of detection. **(E)** On day 28 after injection, we quantified the number of IFNγ-secreting splenocytes by the ELISpot assay. **(F)** The first panel represents nAb titers on day 28 after WNV-WT+ FR and E-MAX+ FR immunization, determined by virus neutralization (VN) assay. The second panel represents comparative nAb titers determined by the VN and 50% plaque reduction neutralization test (PRNT_50_) assays for samples from the same ten E-MAX+ FR mice. Both serum dilution log_2_ and absolute numbers are shown for each serum. Horizontal solid lines are mean. ns: Paired t-test, p = 0.17. Body weight **(G)** and survival **(H)** after footpad immunization with media (Mock) or E-MAX+ FR (10^8^ RNA copies/mouse). 10 mice per MOCK group. 15 mice per E-MAX+ FR group. **(I)** Neutralizing antibody titers on day 30 after E-MAX+ FR immunization. **(J)** Body weight and **(K)** survival of immunized mice after IP challenge with the 10^8^ TCID_50_/mouse of WNV-WT. The challenge was conducted on day 34 after single-dose footpad immunization. In body weight, a standard error of the mean (SEM) is shown. **(L)** WNV RNA loads determined by RT-qPCR in tissues from mice immunized, challenged, and sampled at 30 days after WNV-WT challenge. lod: the limit of detection. *p ≤ 0.05 **p ≤ 0.01; ***p ≤ 0.001; ****p ≤ 0.001 by two-way ANOVA. **(M-R)** Brain sections (n = 3 per group) were stained with hematoxylin and eosin and examined under light microscopy at x200 (**M**, **N**, **O**, scale bar 100µm) and x400 (**P**, **Q**, **R**, scale bar 50µm) magnification. Mock-vaccinated mice challenged with WNV-WT (**N**, **Q**) exhibited inflammatory cell infiltration in the meninges (**N**, black arrows), perivascular inflammation with infiltrating leukocytes (**Q**, blue arrow), and degenerating neurons (**Q**, red arrows). **(S, T)** SNV frequences determined by NGS in working stocks of WNV-WT+ FR and E-MAX+ FR variants. Highlighted in red E region contains CpG+UpA dinucleotide-enriched sequences and modified aa. Raw FASTQ NGS files are deposited in BioProject: PRJNA1277320. (**U**, **V**) Sanger sequencing. Introduced L107F and K440R substitutions showed stability in both WNV-WT+ FR and E-MAX+ FR variants after 15 passages in VERO-ZAP-WT and VERO-ZAP-KO cells. Nucleotide picks highlighted in red squares encode introduced L107F and K440R substitutions. Reference sequences are in S1 File.

In the IP experiment, the most promising vaccine candidate, E-MAX, showed asymptomatic persistence in the brain of four out of eight mice (Fig 4L), and in the footpad experiments, neuroinvasion/lethality in one out of ten mice (Fig 9G and 9H). To further enhance safety, we introduced attenuating aa substitutions—L107**F**, A316**V**, and K440**R**—into the structural E protein domains II and III, generating WNV-WT+ **FVR** and E-MAX+ **FVR** variants (S1 File). These mutations were selected based on previous studies demonstrating their roles in attenuating Japanese encephalitis virus, tick-borne encephalitis virus, and chimeric WNV constructs [8,58,59]. Both WNV-WT+ FVR and E-MAX+ FVR stocks were rescued and propagated in ZAP-KO cells using the ISA method. However, Sanger sequencing revealed that the A316V mutation was unstable and reverted to the wild-type A variant in the E-MAX+ FVR stock (S1 Fig). We, therefore, generated variants containing only the stable L107**F** and K440**R** mutations—WNV-WT+ **FR** and E-MAX+ **FR** (**S1 File**). Correct consensus sequences of WNV-WT+ FR and E-MAX+ FR working stocks were confirmed by NGS (S1 File). All CpG/UpA-enriched dinucleotides and L107F/K440R substitutions remained stable through three passages in VERO-ZAP-KO cells (S2 and S3 Tables and S6 File and Fig 10S and 10T). To further assess the stability of these mutations, we serially passaged WNV-WT+ FR and E-MAX+ FR 15 times in both VERO-ZAP-WT and ZAP-KO cells. The L107F and K440R substitutions remained stable (Fig 10U and 10V).

TCID_50_ titers of WNV-WT+ FR and E-MAX+ FR stocks were quantified via endpoint dilution assay in both ZAP-KO and ZAP-WT cells (Fig 10A). The L107F and K440R substitutions did not affect WNV-WT+ FR titers between ZAP-WT and ZAP-KO cells. In contrast, E-MAX+ FR titers were lower in ZAP-WT cells compared to ZAP-KO cells (Fig 10A), while without a statistically significant difference (p = 0.33, t-test). For the initial ISA transfection, we used a combination of BHK-ZAP-KO and VERO-ZAP-KO cells. BHK cells are highly permissive to DNA transfection, facilitating efficient initiation of viral replication [60–62]. In addition, we successfully ISA-rescued E-MAX+ FR, using only VERO-ZAP-KO cells (S2 Fig), which provided comparable RNA and infectious titers (Fig 10A and S2 Fig). This may be relevant for future vaccine development efforts since Vero cells are more commonly used for commercial live vaccine production.

While footpad injection of 10^7^ RNA copies/mouse of WNV-WT (10^5^ TCID_50_/mouse, titrated on VERO-ZAP-WT cells; 10^5.4^ TCID_50_/mouse, titrated on VERO-ZAP-KO cells; S3 File and Fig 9G and 9H) was lethal in ten-week-old mice, administration of 10^8^ RNA copies/mouse of either WNV-WT+ FR (10^7^ TCID_50_/mouse, as titrated in VERO-ZAP-WT or VERO-ZAP-KO cells) or E-MAX+ FR (10^6^ TCID_50_/mouse as titrated in VERO-ZAP-WT or 10^7^ TCID_50_/mouse as titrated in VERO-ZAP-KO cells) caused no clinical signs (S3 File and Fig 10C; 10 mice per group). However, WNV-WT+ FR remained neuroinvasive, resulting in high brain viral loads (Fig 10D). In contrast, E-MAX+ FR did not show neuroinvasion (Fig 10D). Notably, despite its attenuated phenotype, E-MAX+ FR induced high IFNγ-secreting splenocyte counts (Fig 10E) and nAb titers (Fig 10F and 10I) comparable to WNV-WT+ FR (Fig 10E and 10F).

To assess protection, an independent cohort of mice was mock-immunized (10 mice per group) or immunized with 10^8^ RNA copies/mouse of E-MAX+ FR (15 mice per group) (Fig 10G–I). On day 34 post-immunization, mice were challenged intraperitoneally with 10^8^ TCID_50_/mouse of WNV-WT. E-MAX+ FR-vaccinated mice were fully protected from clinical signs and mortality (Fig 10J and 10K). Importantly, no WNV RNA was detected in the brains of vaccinated mice using RT-qPCR (Fig 10L). Consistent with RT-qPCR data, the histopathological analysis showed that E-MAX+ FR-vaccinated and afterward WNV-WT-challenged mice (Fig 10O and 10R) had preserved brain architecture and normal cerebrum cellular organization, like naïve controls (Fig 10M and 10P). In contrast, mock-vaccinated and WNV-WT-challenged mice displayed severe meningitis (Fig 10N), diffuse neuronal degeneration (Fig 10Q), and extensive perivascular inflammatory cell infiltration (Fig 10Q), consistent with established WNV neuropathology [63–67].

Altogether, introducing L107F and K440R substitutions into the structural E protein of the CpG/UpA-enriched E-MAX variant abrogated neuroinvasion while preserving immunogenicity and single-dose protection.

## Discussion

Live-attenuated vaccines, including flavivirus YFV 17D and JEV SA14-14-2 vaccines, offer the advantages of robust immune responses and long-lasting protection [18,19]. However, classical LAV development relies on prolonged serial passaging in cell cultures or animals, which leads to the uncontrolled accumulation of mutations responsible for (i) attenuation, (ii) tissue-specific adaptation, and (iii) random nonsense mutations due to low fidelity of viral polymerases. Thus, faster and more controlled techniques for LAV development would be advantageous. Flaviviruses, like many other RNA viruses, have evolved to mimic the low CpG content of vertebrate genomes to evade recognition by host ZAP, which targets CpG-rich or CpG-enriched non-self RNA for degradation via the exosome pathway [28]. Flaviviruses also exhibit low UpA dinucleotide content [29,30], which may help them evade RNA-degrading enzymes [32] and possibly ZAP-mediated effects [29–31]. Using the ISA method, a currently leading bacteria-free and rapid technique for rescuing flavivirus genomes [21,23–25,38,68–80], and Vero ZAP-KO cells, which enable the production of high-titer stocks of otherwise highly attenuated CpG/UpA-enriched viruses, we generated a panel of enriched WNV variants. Furthermore, serial passaging of WNV variants demonstrated the stability of the enriched dinucleotides and aa substitutions, particularly under ZAP-KO conditions. In contrast to the time-consuming legacy attenuation process involving many of passages, CpG and UpA dinucleotide enrichment—enabled by *de novo* gene synthesis, ISA reverse genetics, and ZAP-KO cells—may offer a faster and more controlled promising platform for live virus vaccine development against flaviviruses.

The location of dinucleotide enrichment within the flavivirus genome, as well as the combined effects of CpG and UpA dinucleotides, appear to be critical determinants of the attenuated infection phenotype. The flavivirus genome contains key RNA structures in the 5′ and 3′ untranslated regions (UTRs) involved in genome cyclization, as well as a conserved heptanucleotide motif and pseudoknot in the NS2A region required for ribosomal frameshifting and NS1 production [81–83]. Other genomic regions are potentially suitable for introducing synonymous mutations [81,84]. In our previous studies, the E and NS1 regions were used to enrich and attenuate Zika virus and JEV [21,23,25,69,70]. Previous attempts to attenuate flaviviruses, i.e., Powassan virus (POWV), which like WNV, causes high mortality in immunocompetent C57BL/6 mice, by enriching CpG or UpA dinucleotides within NS1-NS3 or NS3 regions were unsuccessful [85]. Therefore, we selected the WNV E, NS1, and NS5 RNA regions for enrichment (Fig 1A). Also, during the preparation of this manuscript, another group released a bioRxiv preprint demonstrating that CpG enrichment in a WNV genomic region spanning part of NS3, the entire NS4A-NS4B, and part of NS5 resulted in partial attenuation in C57BL/6 mice with delayed weight loss compared to wild-type WNV, though infection still resulted in 100% mortality [81]. In contrast, our CpG enrichment approach targeting the **E**, **E**/NS1, and **E**/NS1/NS5 genomic regions of WNV led to significantly reduced mortality—only 14% in C57BL/6 mice (Fig 4B). Given that both studies used comparable experimental conditions (mouse strain, animal age, injection dose, volume, and intraperitoneal injection) [81], the contrasting outcomes likely reflect differences in the enrichment strategy. Interestingly, the WNV variant enriched only for UpA dinucleotides in the same portion of NS3, the entire NS4A-NS4B, and the portion of NS5 region was less virulent, causing 25% mortality [81]. In our study, the E+ UA variant caused substantially lower mortality (64%) compared to the E/NS1-Per, E/NS1/NS5-Per, and WNV-WT variants (79–93%), and E-MAX—the variant enriched with both CpG and UpA in the E region—was the only one that caused no mortality following IP injection and provided protection against lethal challenge (Fig 4B). Notably, further UpA enrichment beyond the E region (i.e., NS5 in the E-MAX/NS5-MAX variant) did not further contribute to attenuated phenotype (Fig 4B). These findings, along with our earlier CpG enrichment studies in Zika virus and JEV [21,23–25,69,70], support that CpG and UpA enrichment specifically within the E region represents a promising strategy for preclinical flavivirus vaccine development. However, while the RNA E region may serve as a universal target, an enrichment approach which provides the universal number of additional CpG and UpA dinucleotides needed to be introduced in different flaviviruses is unlikely to develop. Each flavivirus will likely require virus-specific fine-tuning of CpG and UpA dinucleotide content in the E region to achieve optimal attenuation and immunogenicity. In support, although the combined CpG/UpA enrichment was most effective for WNV E-MAX, our previous studies showed that CpG enrichment alone was sufficient to attenuate and induce immune responses for Zika virus and JEV [21,23–25,69,70].

Analysis of archived gene expression data suggested that central nervous system tissues express low levels of ZAP, which may impair CpG-dependent attenuation of viral infection [30]. Consistent with this, we previously found that CpG-enriched Zika virus variants can replicate in the mouse brain following intracerebral injection; however, unlike the WT virus, the enriched variant did not cause histological lesions [23]. In the present study, we detected E-MAX RNA in some asymptomatic mice at day 60 after IP injection (Fig 4L), and one mouse succumbed at 8 days following high-dose footpad injection with E-MAX (Fig 9G and 9H). While NGS confirmed the stability of the enriched (159 additional CpGs and 50 additional UpAs) and endogenous CpG/UpA dinucleotides in E-MAX-positive brains (Figs 4N and 9M and S2 and S3 Tables and S6 File), it was important to develop an approach that would fully eliminate asymptomatic or rare lethal (10%, one mouse, **Fig 9H**) neuroinvasion events caused by E-MAX. To address this, we applied a novel dual-safety layer attenuation strategy and demonstrated that potential safety concerns with neurotropic flavivirus vaccines can be mitigated through a combination of rational CpG/UpA enrichment and targeted aa substitutions. Indeed, our experiments with E-MAX+ FR—which incorporates two aa substitutions in functional E protein domains—demonstrated a favorable safety profile, immunogenicity, and single-dose protection, supporting the feasibility of this dual-safety layer attenuation strategy. Additionally, our WNV experiments were conducted in C57BL/6 mice, a strain susceptible to lethal WNV infection, which may exaggerate clinical outcomes compared to natural hosts such as horses and humans. Despite the promise of the dinucleotide enrichment vaccine approach, all current studies have been limited to *in vitro* systems or mouse models [21,26,27,86]. Understanding the phenotypes of enriched viruses in natural hosts is essential, as the attenuation and immunogenicity of enriched vaccines depend on species-specific ZAP activity. While ZAPs from humans, mice, and bats all exhibit antiviral activity against HIV-1 *in vitro* [87], and CpG-enriched Zika virus [21] and WNV variants (Fig 6A and 6B) show expected attenuation in human cell lines, *in vivo* studies in natural hosts are critical for advancing the enrichment platform for vaccines. Given that WNV causes natural infections in horses with a pathogenesis similar to that in humans [88], we are actively pursuing funding to evaluate the dual-safety layer enrichment platform in the natural equine host, using E-MAX+ FR as a prototype.

A major advantage of dinucleotide-enriched vaccines is that they encode all structural and non-structural proteins. This is particularly important for flavivirus vaccines, as non-structural proteins from Zika virus, dengue virus, and WNV contain critical immunodominant T cell epitopes [9–17]. Neutralizing Abs are also essential in protection against WNV infection in mice [89–91]. The most promising vaccine candidate E-MAX+ FR consistently induced high nAb titers, ranging from 7.32 to 9.32 log_2_ (1:160–1:640) in two independent experiments (Fig 10F and 10I), with one mouse outlier 11.32 log_2_ (1:2,560) in each experiment, at 28 days after footpad injection. Comparison of the immunogenicity induced by the most promising enriched one-dose candidate E-MAX+ FR in this study with previous inactivated or DNA vaccines is challenging, as those historical vaccines typically required 2–3 doses and the use of adjuvants [7]. A more relevant benchmark is ChimeriVax, a live-attenuated YFV 17D vector vaccine expressing WNV prM/E structural proteins [8]. While accurate comparison requires that serum samples from subjects immunized with different vaccines be tested in parallel using the same assay by the same personnel, we enabled a preliminary comparison by testing serum samples from mice immunized with the most promising vaccine candidate, E-MAX+ FR (Fig 10F), using both virus neutralization (VN) and the 50% plaque reduction neutralization test (PRNT_50_) assays (because along with VN assay many groups use PRNT_50_ assay). Consistent with previous studies [92], we found no substantial differences (p = 0.17, paired t-test) between VN and PRNT_50_ titers (Fig 10F). In mice and rhesus macaques immunized once with ChimeriVax, nAb titers at four weeks post-immunization ranged from 1:20–1:200 (4.32 to 7.64 log_2_) and 1:320–1:640 (8.32 to 9.32 log_2_) PRNT_50_, respectively [8]. In comparison, E-MAX-FR induced nAb titers were ranging from 1:120–1:640 PRNT_50_ (6.9 to 9.32 log_2_) (Fig 10F) at the same point following single dose immunization (Fig 10F). Another recent WNV live vaccine candidate, based on a large replacement of the 3′UTR internal poly(A) region, also induced comparable nAb titers (1:40–1:640 PRNT_50_) [46]. Both live WNV vaccine types also induced robust comparable IFNγ responses in splenocytes, as measured by ELISpot (Fig 10E) [46]. In this study, E-MAX+ FR protected against a lethal challenge with a remarkably high dose of WNV-WT. For the challenge, we used the highest dose attainable from our stock for IP challenge—10^8^ TCID_50_ in 100µl per mouse. By comparison, previous studies evaluating chimeric WNV vaccines in mice used a lower challenge dose of approximately 10^3^ TCID_50_/mouse [8].

Despite their attenuated phenotypes, E-MAX and E-MAX+ FR elicited nAb titers and IFNγ-producing splenocyte responses comparable or higher than those induced by the more aggressive WNV-WT, E/NS1-Per, E/NS1/NS5-Per, and other enriched WNV variants (Figs 4B, 4D, 4J, 4K, 9D, 9E, 9I, 10E, 10F, and 10I). RNA-seq analysis in Huh7 cells infected with E-MAX (Figs 6 and 7) suggests that combination of attenuation and concurrent innate immune activation by the CpG/UpA-enriched content may contribute to these comparable *in vivo* immune responses. This hypothesis is also based on previous findings which showed increased ZAP binding to CpG-enriched HIV-1 RNA [27], as well as our recent discovery of the ZAP-dependent interactome [40]. In Zika virus-infected ZAP-WT and ZAP-KO cells, we identified over 200 cellular proteins whose interaction with wild-type flavivirus RNA was determined by ZAP. Among the top ZAP-dependent interactors were RNA helicases and other proteins involved in innate immune signaling pathways [40]. Altogether, these findings suggest that increased ZAP binding to the CpG-enriched RNA of attenuated vaccine strains may facilitate the recruitment of other host proteins involved in IFN responses. This mechanism may provide sufficient immune stimulus to induce protection, even during attenuated infection. It will be interesting and important to experimentally test this hypothesis and further investigate how early CpG/UpA-dependent interactions between enriched viral RNA and host proteins influence the quality of innate and adaptive antiviral responses, and whether this can be leveraged to fine-tune vaccine immunogenicity.

A limitation of the present study is that we do not know how dinucleotide enrichment affects viral RNA structure and whether potential changes in RNA structure contribute to the attenuated infection phenotypes observed *in vitro* and *in vivo*. Also, relatively modest changes in codon pair bias (CPB) were introduced during WNV enrichment (S1 Table). Several studies have shown that codon-pair deoptimization does not attenuate viruses; rather, attenuation by codon-pair deoptimization is an artifact of increased CpG and UpA dinucleotide frequencies [29,93]. We have also demonstrated that CpG-enriched Zika virus and JEV variants, which contain negligible CPB changes, are attenuated both *in vitro* and *in vivo* [21,25]. However, in the present study, our methodology does not allow to conclusively determine whether CPB changes in enriched WNV variants contribute to the attenuated phenotype.

In conclusion, this study demonstrates that CpG/UpA dinucleotide enrichment in the RNA region encoding E protein of an aggressive WNV strain in combination with point aa substitutions yields a promising platform for further preclinical vaccine development. Also, an exciting research direction supported by our findings is the investigation of interactions between CpG-, UpA-, and CpG/UpA-enriched viral RNA and cellular proteins, and whether these interactions during early infection affect downstream innate and adaptive immune responses. Understanding these mechanisms may aid the development of vaccines that selectively activate desirable immune pathways.

## Materials and methods

### Ethics statement

The animal studies were approved by The Ohio State University Institutional Biosafety Committee (e-Protocol #2022R00000111) and the Institutional Animal Care and Use Committee (e-Protocol #2023A00000095).

### *In silico* CpG and UpA enrichment, permutation, and mutagenesis in the WNV genome

For CpG and UpA dinucleotide enrichment in the WNV genome, we used the SSE software package [33,34]. Enrichment does not alter the protein code and has no significant impact on codon usage metrics. Scrambled or permuted controls were constructed using CDLR randomizing in the SSE [29,33,34]. Permuted controls incorporate the maximum number of synonymous changes within the target regions while preserving the wild-type mono- and dinucleotide frequencies and encoded proteins. During randomization or enrichment, we sought to avoid areas of the genome containing RNA elements required for the replication or translation of the virus genome, such as cis-replicating elements, gene start or gene-end signals; we also avoided regions with prominent secondary structures. The genomic parameters and sequences of the wild-type, permuted, and dinucleotide-enriched WNV variants are in S1 Table and S1 File.

West Nile virus is neurotropic [94]. Therefore, after *in vitro* and *in vivo* testing of all CpG/UpA-enriched WNV variants (S1 Table), we introduced three, and later two (one proved unstable), aa substitutions into the E protein of the most promising vaccine candidate prototype, E-MAX. These aa substitutions were introduced into E-MAX or WNV-WT (for comparative purposes) to generate WNV-WT+ FVR, WNV-WT+ FR, E-MAX+ FVR, and E-MAX+ FR and further enhance the attenuation of E-MAX beyond attenuation achieved through dinucleotide enrichment. The selection of mutation sites in the WNV genomic region encoding the E protein was informed by earlier research that identified mutations correlated with attenuation and reduce flavivirus neuroinvasion and neurovirulence in related flaviviruses, such as JEV and tick-borne encephalitis virus [8,58,59,95–97]. Specifically, we introduced L107F, A316V, and K440R mutations in the E protein domains II and III, respectively (S1 File).

### Mouse experiments

We used the well-characterized C57BL/6J mouse model for WNV infection [46–48]. Eight- to ten-week-old C57BL/6J mice (strain #: 000664) were ordered from The Jackson Laboratory. After one week of acclimatization, mice were injected (intraperitoneally (IP) or in footpad) with the virus-free media (MOCK) or WNV variants (S1 File and S1 Table). In the initial IP studies (Fig 4), we used equal numbers of female and male mice. As no apparent differences in outcomes were observed between sexes, subsequent studies were conducted using male mice.

In mouse experiments comparing the infection phenotypes of WNV-WT and various modified WNV variants after IP and footpad injections, the injection doses were normalized based on viral RNA copy numbers in the corresponding stocks quantified by RT-qPCR targeting the untranslated region conserved across all WNV variants (see RT-qPCR assay below). We used normalized injection doses of 10^5^-10^8^ WNV variant RNA copies per mouse. The 10^5^-10^8^ RNA copies/mouse of the WNV-WT variant corresponds to 10^3^-10^6^ TCID_50_/mouse, as quantified in VERO-ZAP-WT cells (S3 File); this injection dose range was used in previous WNV mouse studies [8,46–48]. This viral RNA-based normalization approach was adopted for several reasons:

(i) CpG-enriched viruses exhibit variable infection phenotypes across different cell lines, as evidenced in our prior studies on Zika virus employing three cell lines [21]. Currently, there are no standardized protocols for evaluating titers of CpG-enriched vaccine candidates, and even commonly used cell lines may exhibit variability across laboratories. Therefore, to facilitate more accurate comparability across studies, and particularly across studies from different laboratories, we propose standardizing vaccine dosing by quantifying viral RNA copies using RT-qPCR targeting the unmodified UTR.
(ii) Dinucleotide enrichment-driven attenuation is determined by viral RNA composition during the early stages of replication, making it essential to deliver equivalent RNA amounts across WNV variants for accurate attenuated phenotype comparisons. RNA-based dose normalization or titration in cells lacking ZAP expression (C6/36 insect cells) has been successfully applied in other comparative studies of dinucleotide-enriched viruses [81,86]. In support, as shown in Figs 6 and 7, dinucleotide enrichment may also influence early (3h after inoculation) RNA-mediated immune recognition of vaccine candidates and associated transcriptional responses, reinforcing the need for RNA dose equivalency in immunogenicity studies.
(iii) Highly attenuated variants, such as E-MAX/NS5-MAX, showed very low infectious titers in VERO-ZAP-WT cells (and infectious titers below the detection/quantification limits in the injection volume for mouse inoculation, S3 File) despite comparable infectivity to WNV-WT in ZAP-KO cells (Fig 1D) and immunogenicity in mice (Fig 4J and 4K). Therefore, VERO-ZAP-WT infectious titer-based normalization is not feasible for such variants. For example, if in this study we normalized all injection doses in the IP mouse experiment (Fig 4) to infectious titer of E-MAX/NS5-MAX, which had the lowest infectious titer among all 10 variants on ZAP-WT cells (10^1.8^ TCID_50_/ml; Fig 1D), then the IP dose (100 μl/mouse) for all 10 WNV variants would be 10^0.18^ TCID_50_ per mouse, an unreliable dose for mouse experiments with attenuated WNV vaccine candidates.
(iv) Following RNA-based normalization, the infectious titers of different WNV variants (per mouse) measured in VERO-ZAP-WT (excluding E-MAX/NS5+ CG and E-MAX/NS5-MAX) and VERO-ZAP-KO cells (excluding E-MAX/NS5+ CG and E-MAX/NS5-MAX), were comparable (10^0.0^-10^1.4^ difference) in each experimental comparison (S3 File). These relatively minor differences were unlikely to have biased the results in mice. Supporting this, in comparative experiments between WNV-WT and E-MAX (the prototype for the most promising vaccine candidate), E-MAX was equally or more immunogenic than WNV-WT (Fig 4D, 4J, and 4K) despite a lower immunization dose (10^4.4^ versus 10^5.0^ TCID_50_/mouse) based on infectious titers determined in VERO-ZAP-WT cells (S3 File). In another experiment (Fig 9G, 9H and S3 File), E-MAX caused considerably less mortality than WNV-WT (10% vs. 100%; only one death out of 10 mice for E-MAX) despite being administered at a higher dose (10^5.0^ versus 10^5.4^ TCID_50_/mouse based on infectious titers determined in VERO-ZAP-WT cells; 10^5.4^ versus 10^6.8^ TCID_50_/mouse based on infectious titers determined in VERO-ZAP-KO cells, footpad injection) (S3 File). Following RNA-based normalization, E-MAX+ FR was also administered to mice (Fig 10) at higher infectious doses (10^5.4^ versus 10^6.0^ TCID_50_/mouse based on infectious titers determined in VERO-ZAP-WT cells; 10^6.8^ versus 10^7.0^ TCID_50_/mouse based on infectious titers determined in VERO-ZAP-KO cells, footpad injection; S3 File) than E-MAX (Fig 9 and S3 File) without causing neuroinvasion or lethality, further supporting the combined attenuating effects of CpG/UpA enrichment and FR amino acid substitutions.

In mouse studies evaluating the infection phenotypes, safety and immunogenicity of the most promising WNV vaccine prototype—E-MAX+ FR (Fig 10 and S3 File)—we used footpad immunization doses of 10^8^ WNV RNA copies per mouse. This corresponds to 10^6^ TCID_50_/mouse as quantified in VERO-ZAP-WT cells (S3 File). This dose is close to the 10^5^ PFU/mouse (determined in wild-type Vero cells) used in previous studies with several live WNV vaccine candidates in the same C57BL/6J mouse model [8,46] as it is known that for Spearman-Kärber calculations 0.56 PFU = 1 TCID_50_ [98].

In all studies (Figs 4, 9, and 10) evaluating the protection evoked by vaccine candidates, mice were challenged with 10^10^ WNV-WT RNA copies per mouse (equivalent to 10^8^ TCID_50_/mouse in VERO-ZAP-WT cells and 10^8.4^ TCID_50_/mouse in VERO-ZAP-KO cells) (S3 File). This represents the highest dose of WNV-WT that available stock permits to deliver IP in a 100 µL volume.

Mice were monitored for clinical signs and body weight changes using a previously described clinical scoring system [21]: 0—no visible abnormalities; 1—mild ataxia or tremors; 2—obvious ataxia or tremors; 3—depression, hunching, reluctance to walk, and falling to the side when walking; 4—close to moribund but still somewhat responsive; 5—paralysis; 6—loss of greater than 20% baseline body weight; 7—found dead. Scores of 4, 5, or 6 were used as the endpoint and for mouse euthanasia.

Blood, spleen, and brain samples were collected to assess WNV-neutralizing antibody (Ab) titers, WNV RNA loads, infectious titers, and ELISpot responses. The left hemisphere of the brain was fixed in 10% buffered formalin for hematoxylin and eosin (H&E) staining for histopathological examinations.

Details of cells, viruses, ISA and virus stock generation, NGS and Sanger sequencing, comparative infection phenotypes of WNV variants in wild-type and ZAP-KO cells, comparative ISA, RNA-seq, Western blot, infectious virus titration, RT-qPCR, virus neutralization and ELISpot assays, histopathology and statistics are in S1 Supplemental Materials and Methods.

## Supporting information

S1 Supplemental Materials and Methods (PDF)

S1 Fig Sanger sequencing of WNV FVR variants. Sanger sequencing revealed that the A316V mutation was not stable and reverted to wild-type A in the E-MAX+ FVR stock.
(PDF)

S2 Fig E-MAX-FR ISA-rescued in only Vero cells. For ISA transfections, we initially used a combination of ZAP-KO BHK-21 and Vero cells, a standard approach to enhance transfection efficiency. Subsequently, we repeated the rescue of E-MAX+ FR, the most promising vaccine candidate in this study, using only VERO-ZAP-KO cells. Representative images of cytopathic effect (CPE) in VERO-ZAP-KO cells transfected with ISA DNA fragments representing E-MAX+ FR or mock (A-D). Magnification ×100. Scale bar: 100 µm. ISA transfection was performed as described in S1 Supplemental Materials and Methods, but with DNA fragments mixed in equimolar concentrations to obtain a total of 3 µg of DNA per transfection well. Transfections were carried out in 6-well plates with five replicates and one mock-transfected control well (D). CPE and infectious E-MAX+ FR were observed in three (A-C) out of five wells. (E) Infectious titers of the E-MAX+ FR stock produced in only VERO-ZAP-KO cells and titrated in ZAP-WT or ZAP-KO Vero cells. lod: limit of detection. ns: Unpaired t-test: *p* = 0.3333. (F) RNA loads of the E-MAX+ FR stock produced in VERO-ZAP-KO cells. lod: limit of detection. (G) Sanger sequencing confirmed the presence of the introduced L107F and K440R substitutions in the E-MAX+ FR variant rescued in only VERO-ZAP-KO cells. Nucleotide peaks encoding these substitutions are highlighted in red squares. Reference sequences are provided in S1 File.
(PDF)

S1 File WNV reference sequences. (PDF)

S2 File ISA fragments. (PDF)

S3 File WNV RNA copy numbers, infectious titers, and viral RNA to infectious titer ratio. (PDF)

S4 File Raw Western blot images. Raw Western blot images of ZAP, DDX50, RIG-I in Huh7 and Vero cells following infection with wild-type and dinucleotide-enriched WNV variants. Human HuH-7 and monkey Vero cells were inoculated with 1,000 RNA genome copies/cell of WNV-WT, E-MAX, E+ CG, E+ UA, or MOCK. Cells were washed and lysed at 6 h post-inoculation for Western blot. Green bands indicate target proteins of interest: ZAP (100 kDa; multiple bands may represent the four isoforms described for human ZAP, which are still not experimentally characterized in monkeys), DDX50 (83 kDa), and RIG-I (107 kDa). Red bands represent β-actin internal loading control (42 kDa). Western blot was performed in biological duplicate for each experimental condition.
(PDF)

S5 File West Nile virus NGS coverage and depth. (PDF)

S6 File Shannon entropy. (PDF)

S1 Table CpG and UpA composition in WNV variants. (PDF)

S2 Table WNV SNVs identified by NGS. (XLSX)

S3 Table WNV SNVs identified by NGS after downsampling. (XLSX)

S4 Table RNA-seq data. (XLSB)

S5 Table ISA fragments and primers. (PDF)

S6 Table Primers for PrimalSeq NGS and Sanger sequencing. (PDF)
