# Supplementary material for: West Nile virus vaccine candidates attenuated by dinucleotide enrichment are immunogenic and protective against lethal infection
Source: PLoS Pathog. 2025 Oct 3;21(10):e1013560. doi: 10.1371/journal.ppat.1013560 (PMC12513643; doi:10.1371/journal.ppat.1013560)
Supplement: S1 Supplemental Materials and Methods — (PDF) [file ppat.1013560.s001.pdf]

Supplemental materials and methods

Supplemental materials and methods

Cells

Wild-type African green monkey kidney epithelial cells Vero E6 (VERO-ZAP-WT), human hepatoma cell line Huh-7 and baby hamster kidney BHK-21 (BHK-ZAP-WT) cells, along with CCCH-type zinc finger antiviral protein (ZAP) knockout (ZAP-KO) derivatives (VERO-ZAP-KO and BHK-ZAP-KO) were cultured in Dulbecco's Modified Eagle's Medium (DMEM, Fisher, MA, USA; #11-965-118) supplemented with 10% heat-inactivated fetal bovine serum (FBS, Fisher, MA, USA; #A5256801), 1x Penicillin-Streptomycin (Fisher, MA, USA; #15140122), and 2.67 mM sodium bicarbonate (Fisher, MA, USA; # 25080094) at +37°C in a 5% CO_2 humidified incubator. The generation and validation of VERO-ZAP-KO and BHK-ZAP-KO cell lines were previously described in detail (1-3).

Infectious Subgenomic Amplicons and virus stock generation

The Infectious Subgenomic Amplicons (ISA) reverse genetics method was used as previously described (3, 4). As the reference sequence for enrichment, we used the WNV NY99 strain [GenBank DQ211652.1]. To rescue WNV variants, we used overlapping (70-80 nt overlap) ISA DNA fragments covering the entire viral genome (**S2 File**). The first and last fragments for each virus are flanked with pCMV promoter and HDR/SV40pA sequences. Sequences, primers, and origin of all ISA fragments for each WNV variant are described in **S2 File** and **S5 Table.**

Overlapping clonal genes or synthetic DNA ISA fragments were amplified with Invitrogen Platinum PCR SuperMix High Fidelity (Fisher, MA, USA; #12-532-016) (primers are listed in

S5 Table), mixed in equimolar concentration to obtain the final 1µg of DNA for transfection, and transfected into BHK-ZAP-KO cell monolayers (6-well plates, five transfection replicates, one mock-transfection control well) with Lipofectamine 3000 (Fisher, MA, USA; #L3000015) in 640 µl of OptiMEM (Fisher, MA, USA; #11-058-021) for 12 h at +37°C, 5% CO_2. Afterward, OptiMEM media was removed and replaced with 3 ml of DMEM with 10% of FBS containing $4x10^5$ VERO-ZAP-KO cells on top of BHK-ZAP-KO cells, and plates were incubated for an additional 6-7 days (passage 0). For passage 1, 400 µl of medium from each well of passage 0 was transferred to wells with VERO-ZAP-KO cells and incubated for 4 days. During passages 0 and 1, cells were monitored for cytopathic effect (CPE). Generated flaviviruses were passaged 2 times on VERO-ZAP-KO cells to generate working virus stocks. Cell culture media from the final working stocks were centrifuged (12,000 x g, 20 min, +4°C), aliquoted, and frozen (-80°C). For ISA transfections, we initially used a combination of ZAP-KO BHK-21 and Vero cells, as this is a standard approach to enhance transfection efficiency (5-7). Afterward, we also repeated the rescue of E-MAX+FR, a most promising vaccine candidate in this study, using only VERO-ZAP-KO cells (**S2 Fig**). Viral titers in stocks were quantified in duplicate in VERO-ZAP-WT or VERO-ZAP-KO cells with an endpoint dilution assay described below. The absence of mycoplasma contamination in all virus stocks and cell cultures was confirmed using PCR Detection Kit (Millipore Sigma, MA, USA; #MP0035). Genomic sequences in all working stocks and throughout serial passaging were confirmed using next-generation sequencing (NGS) and Sanger sequencing, as described below.

Supplemental materials and methods

Next-generation sequencing (NGS) and Sanger sequencing in viral stocks and during serial passage

Working viral stocks of WNV variants—WNV-WT, E/NS1-Per, E/NS1/NS5-Per, E+CG, E/NS1+CG, E/NS1/NS5+CG, E-MAX, E+UA, E-MAX/NS5+CG, E-MAX/NS5-MAX (**S1 Table**), WNV-WT+FR, and E-MAX+FR—were used for RNA extraction and NGS sequencing.

To define the stability of enriched CpG/UpA dinucleotides and introduced amino acid substitutions, we passaged E-MAX, E-MAX+FVR, and E-MAX+FR, along with their corresponding wild-type variants, WNV-WT, WNV-WT+FVR, and WNV-WT+FR, in VERO-ZAP-WT or VERO-ZAP-KO cells 10-15 times. For each subsequent passage, cells were seeded in 6-well plates 24 h before virus inoculation and inoculated with 1:1,000 diluted (wild-type prototypes) or undiluted (dinucleotide-enriched variants and variants with amino acid substitutions). After 1 h (WNV-WT, WNV-WT+FVR, and WNV-WT+FR) or 5 h (attenuated E-MAX, E-MAX+FVR, and E-MAX+FR) at +37°C, the inoculum was discarded, and the cells were covered with fresh culture medium. They were then incubated for 4 days at +37°C and 5% CO_2. Afterward, the supernatants were collected to inoculate corresponding cells for the next passage. Serial passages for all variants were conducted in two biological replicates.

All 12 working WNV variant stocks (**S1 Table**), as well as WNV-WT and E-MAX at passages 5th and 10th were sequenced using classical non-targeted NGS. The virus stocks obtained from the cell culture contain high virus loads, which permit the use of the NGS protocol for library preparation without PCR pre-amplification. Briefly, RNA was extracted from the viral stocks (140 µl) using the QIAamp Viral RNA Kit (Qiagen) and concentrated via vacuum centrifugation. After vacuum centrifugation, RNA was reverse-transcribed into cDNA using the Thermo Scientific Maxima H Minus First Strand cDNA Synthesis Kit (Fisher, MA, USA; #1652). The cDNA was further used

for the preparation of double-stranded DNA (dsDNA) using *E. coli* DNA Polymerase I (New England Biolabs), *E. coli* DNA Ligase (New England Biolabs), RNase H (New England Biolabs), Invitrogen dNTP Mix (10 mM; Thermo Scientific™), and NEBNext Second Strand Synthesis (dNTP-free) Reaction Buffer (New England Biolabs). After dsDNA preparation, the samples were purified using NEBNext sample purification beads (New England Biolabs; 2:1 ratio of beads). DNA concentrations were measured with the Qubit dsDNA HR Assay Kit and Qubit 4.0 Fluorometer (Thermo Fisher Scientific). The purified DNA was used for library construction with the NEBNext® Ultra™ II FS DNA Library Prep with Sample Purification Beads (New England Biolabs) and NEBNext® Multiplex Oligos for Illumina® (96 Index Primers; New England Biolabs) with (USER enzyme and ADAPTOR; New England Biolabs) according to the manufacturer's instructions. Individual sample libraries were quantified with the Qubit dsDNA HR Assay Kit and Qubit 4.0 Fluorometer (Thermo Fisher Scientific). The peak DNA fragment size was identified using the TapeStation HS-DNA 1000 Bioanalysis kit on an Agilent 4150 TapeStation (Agilent). In total, 2 ng of each barcoded library was pooled, quantified (Qubit 4.0), quality-checked (Agilent TapeStation), and converted to moles: Molecular concentration [nM] = Library concentration [ng/µL]/((Average library size x 650)/1,000,000). The pooled library was used to generate paired-end 150PE reads with the 6000 S1 1.5 Reagent Kit on the NovaSeq 6000 System (Illumina).

To analyze data from non-targeted NGS, the output FASTQ files were quality-trimmed using Trimmomatic 0.39 (8), and the trimmed reads were aligned with BWA to their reference sequences (**S1 File**). The sorted and merged BAM files, representing the aligned and assembled NGS reads, were used to calculate NGS coverage and depth using an R script (9-11) (**S5 File**). Then, we identified the consensus sequences for all working WNV variant stocks using Unipro UGENE 46.0 (64-bit) using the default algorithm (12). Classical non-targeted NGS provided sufficient

average sequencing depth, and nearly the entire WNV genome coverage (on average, 25-127 nt at both UTRs were not covered in WNV variants) (**S5 File)**. For further NGS data processing and calling single-nucleotide variants (SNVs), we used BAM files as input for VarScan 2.4.4 (13). We identified SNVs by aligning NGS sequences with reference sequences used to generate ISA fragments (**S1 File**). We also compared CpG and UpA dinucleotide content in reference sequences (**S1 File**), working WNV variant stock sequences, and sequences obtained after serial passaging. For the validated best practice (9-11, 14), we considered SNVs with a frequency of at least 3% detected in two biological replicates (replicates were used in serial passage experiments); insertions and deletions were not analyzed (**S2 Table**).

To quantify and compare Shannon entropy between all 12 WNV variant working stocks, we downsampled NGS depth to the WNV-WT variant with the lowest NGS depth (2,395×; **S5 File**) and generated normalized SNV data (**S3 Table**). For passaged viruses, downsampling was done to the lowest NGS depth of 265× (excluding samples representing highly attenuated E-MAX variant passaged in VERO-ZAP-WT cells). Reads were randomly downsampled using *samtools view -s*. Per-base Shannon entropy was calculated from the downsampled BAM alignments using *Rsamtools* and *data.table* packages in R, counting A, C, G, and T bases after filtering for mapping quality ≥ 20 and base quality ≥ 20, and excluding sites with total depth < 10. Shannon entropy at each site was calculated as the negative sum of each base's frequency multiplied by the base-2 logarithm of that frequency. Results were plotted with rolling mean windows of 50 nt.

RNA samples from mouse brains positive for E-MAX variant were used for targeted PrimalSeq NGS which provides higher sequencing depth (~10^5-10^6, **S5 File**), coverage, and sensitivity than classical non-targeted NGS (14-16). We selected the brain from the single mouse that succumbed

to E-MAX infection (**Fig 9H**); and brain samples from two mice injected IP with E-MAX that showed no clinical signs for 60 days post-injection but tested positive by RT-PCR (**Fig 4L**; two mice with the highest PCR values were tested). The targeted PrimalSeq protocol was applied as we previously described (9-11), but using NEBNext® Ultra™ II FS DNA Library Prep with Sample Purification Beads, and Illumina NextSeq2000 2x300bp Sequencing. The PrimalSeq protocol implies the nearly entire virus genome amplified in ~446-469 bp overlapping fragments with multiplexed PCR reactions implying 28 primer pairs (**S5 Table**) (14-16). WNV cDNA (2 μL) was amplified in two multiplex WNV specific PCR reactions with Primal Scheme primers (**S5 Table**). These two multiplex primer schemes were designed with a web-based tool Primal Scheme (14) using the E-MAX strain reference sequence (**S1 File**) For NGS data processing and variant calling, we used an open-source software package iVar (14) as we described (9-11). As the reference, we used corresponding E-MAX reference sequence, which was used for gene synthesis and ISA rescue (**S1 File**). For the validated best iVar practices (14), we analyzed virus genomic regions with a sequencing depth of at least 400×; SNVs with a frequency of at least 0.03 (3%) were considered for analysis; insertions and deletions were not analyzed. In addition, genomic regions amplified with primers that contained nucleotide mismatches within the binding sites were omitted. The detailed computational protocol used in this study for iVar was previously published by our and other labs (9-11, 14). To quantify Shannon entropy in the NGS E-MAX data from mouse brains, we downsampled reads from each sample to match the lowest average depth (285,257× for mouse WEUF42.2; **S5 File** and **S2 Table**). SNV detection was then re-performed on the normalized datasets (**S3 Table**). Downsampling and entropy calculations (**S6 File**) followed the procedures described above. All raw NGS data have been deposited in the Sequence Read Archive [BioProject: PRJNA1277320; PRJNA1310643].

Supplemental materials and methods

To evaluate the stability of the introduced amino acid substitutions (L107F, A316V, and K440R) in WNV-WT+FVR, E-MAX+FVR, WNV-WT+FR, and E-MAX+FR across different passages, viral RNA was extracted using the QIAamp Viral RNA Kit. PCR was performed using the Invitrogen SuperScript IV One-Step RT-PCR System (Fisher, MA, USA; #12-594-025) according to the manufacturer's instructions, with primers targeting sequences encoding the E protein bearing L107F, A316V, and K440R mutations (**S6 Table**). PCR products were run on a 1% agarose gel, and DNA was purified and concentrated using DNA Clean & Concentrator-5 (Zymo Research, CA, USA; #D4013) as per the manufacturer's instructions. DNA was sequenced using Sanger sequencing, and the sequences were aligned with corresponding reference sequences (**S1 File**) and visualized using Unipro UGENE software package.

Comparative infection phenotypes of WNV variants in wild-type and ZAP-KO cells

We tested alongside WNV-WT, E/NS1-Per, E/NS1/NS5-Per, E+CG, E/NS1+CG, E/NS1/NS5+CG, E-MAX, E-UA, E-MAX/NS5+CG, and E-MAX/NS5-MAX (**S1 Table**) in VERO-ZAP-WT and VERO-ZAP-KO cells. Fifty thousand cells per well were seeded into 96-well plates. The next day, the media were removed, and monolayers were inoculated with MOIs of 0.01 (normalized based on titers in VERO-ZAP-KO cells) of each variant in 50 µl for 2 h at 37°C and 5% CO_2. Afterward, the inoculum was removed and replaced with 150 µl DMEM supplemented with 2% FBS, 1x Penicillin-Streptomycin, and 2.67 mM sodium bicarbonate. At 1 h and 48 h, supernatants from 3 biological and 2 technical replicates were collected for subsequent RNA extraction and virus-specific RT-qPCR (2). The difference between the ZAP-WT and ZAP-KO conditions was represented by the fold change in viral RNA loads in supernatants collected at

1 h and 48 h after inoculation. Samples collected at 1 h after inoculation were used to normalize for residual inoculum RNA.

Cells in six wells, representing three biological and three technical replicates, were fixed and stained for flavivirus E protein, as previously (3, 9, 17-21). For immunohistochemistry staining, we used 4G2 (ATCC, MA, VA, USA; #HB-112) antibodies (Ab) against WNV E protein (19, 21-23). To quantify and compare immunohistochemistry staining between ZAP-WT and ZAP-KO conditions, images were randomly acquired at 200x magnification in all replicates with the same microscopic settings. Afterward, each image was processed in ImageJ (NIH, USA) using Automatic Particle Counting (https://imagej.net/imaging/particle-analysis) as previously described (3).

Comparative ISA

To compare the efficiency of ISA in rescuing WNV variants on VERO-ZAP-WT and VERO-ZAP-KO cells, we selected the E-MAX and E-MAX/NS5+CG variants. Overlapping DNA ISA fragments were amplified as described above, mixed in equimolar concentration to obtain the final quantities of 1 µg of DNA per transfection, and transfected into a plate with either ZAP-WT or ZAP-KO BHK cell monolayers (five transfection replicates and one mock-transfected control well per 6-well plate) with Lipofectamine 3000 in 640 µl of OptiMEM for 12 h at +37°C, 5% CO_2. Afterward, OptiMEM media was removed and replaced with 3 ml of DMEM with 10% of FBS containing 4×10^5 VERO-ZAP-WT cells added on top of BHK-ZAP-WT cells, and 4×10^5 VERO-ZAP-KO cells on top of BHK-ZAP-KO cells, and incubated for additional 4 days (passage 0). These experiments were conducted simultaneously; also, the DNA amplicons for transfection in

Supplemental materials and methods

ZAP-WT or ZAP-KO cells were obtained from the same PCR reactions. For passage 1, 400 µl of media from each well of passage 0 was transferred to wells with corresponding VERO-ZAP-WT and VERO-ZAP-KO cells and incubated for 2 days. During passage 1, supernatants were collected on day 2 from four well replicates, and infectious virus titers were quantified using the end-point dilution assay with VERO-ZAP-WT cells, as described below. The quantitative difference between ZAP-WT and ZAP-KO transfection conditions was represented by 50% tissue culture infectious dose ($TCID_{50}$) infectious titers in cell culture supernatants. Additionally, cellular monolayers from each transfected and mock-transfected well were fixed and stained for viral E protein via immunohistochemistry (IHC) as described in the infectious virus titration below. To quantify and compare IHC staining between ZAP-WT and ZAP-KO conditions, three images (magnification 200×) were acquired in all five well replicates at random regions. Afterward, each image was processed in ImageJ (NIH) (https://imagej.net/imaging/particle-analysis) using Automatic Particle Counting, as described previously (3).

Infection phenotypes in Huh7 cells and RNA-seq

We used Huh7 cells, a human cell line that is competent in interferon (IFN) responses and is commonly used to study virus-cellular protein interactions, including those of flaviviruses (24-29). Cells were seeded in 6-well plates at a density of 1.1×10^6 cells/well in DMEM supplemented with 10% FBS. Four plates were prepared for each experimental group (Mock, WNV-WT, E-MAX, E+CG, and E+UA) across four time points (3, 6, 12, and 24 h post-inoculation). Cells were synchronously inoculated with 1,000 viral RNA genome copies per cell, as determined by RT-qPCR targeting the UTR, which was unmodified in all WNV variants. This viral RNA copies-normalized inoculation facilitated specific evaluation of innate immune responses elicited by

dinucleotide-enriched RNA during early infection, consistent with established methodologies for assessing gene expression differences between wild-type and enriched enteroviruses (30). For mock inoculation, we used virus-negative cell culture supernatants obtained under the same conditions as virus stocks. Following a 2-hour incubation at 37°C, the inoculum was removed, cells were washed with 1× PBS, and fresh DMEM with 2% FBS was added. Cells were incubated at 37°C for 3, 6, 12, and 24 h. At each time point, the media were synchronously removed, and the cells were washed with commercial 1× PBS before being homogenized in 1 ml TRI Reagent Solution (Fisher, #AM9738). RNA extraction was then performed using the PureLink RNA Mini Kit (Fisher, #12-183-018A) according to the manufacturer's protocol. Concentrations of total cellular RNA and RNA quality (RNA Integrity Numbers, RINs) were measured using the High Sensitivity RNA ScreenTape kit (Agilent Technologies, #5067-5579) on a TapeStation 4150 instrument with TapeStation Analysis Software - Prokaryotic RNA Analysis 5.1 (Agilent Technologies, CA., USA). All tested RNA samples contained 40.92±0.33 ng/µl of total RNA with RINs ≥ 8-9.

For RT-qPCR quantification of WNV infection, 4 µl of extracted RNA (40.92±0.33 ng/µl) from each sample was analyzed using a probe-based one-step RT-qPCR assay (31, 32) targeting the UTR which was unmodified in all WNV variants, as described below. Upon logarithmic transformation, PCR values were expressed as virus RNA genome copies per 1 µl of total RNA extract (equivalent to 41 ng of total RNA). Fold changes of WNV RNA copies for all WNV variants, and for all four replicates at 6, 12, and 24 h post-inoculation were calculated relative to corresponding WNV RNA copies at 3 h post-inoculation.

Supplemental materials and methods

The same RNA samples tested in WNV-specific RT-qPCR assay were analyzed with RNA-seq. Residual DNA was eliminated using the TURBO DNA-free Kit (#AM1907, Fisher). Poly(A)-tailed mRNA was enriched using NEBNext Poly(A) mRNA Magnetic Isolation Module (#E7490L, NEB). RNA libraries were constructed using the NEBNext Ultra II Directional RNA Library Prep Kit (#E7760L, NEB). Sequencing was performed on the Illumina NovaSeq platform (paired-end reads, S1 1.5 kit, 300 cycles).

Adaptor sequences were trimmed and low-quality reads were filtered out from FASTQ files using *Trimmomatic*. RNA-seq analysis was conducted as previously described with some modifications (17, 19, 20, 33). Briefly, paired-end reads were processed using the *kallisto* (34) and were quantified using the human reference genome assembly (ENSEMBL GRCh38.113, NCBI RefSeq assembly: GCA_000001405.29). The count table for RNA-seq data was assembled using the *tximport::tximport* function in R. After importing the data into the R environment, we removed data for genes with low expression using the *edgeR::filterByExpr* function. Normalization was performed using the *edgeR::calcNormFactors* function, and the *limma::voom* function was used to convert the data into a normal distribution. We calculated differential expression using the *limma::lmFit* function with empirical-Bayes moderation via the *limma::eBayes* function (**S4 Table**). Raw sequencing data were deposited in BioProject: PRJNA1281688.

Two complementary analyses were applied to compare Differentially Expressed Genes (DEGs; **S4 Table**). First, we compared gene expression directly between mock-infected cells and cells infected with different WNV variants at 3, 6, 12, and 24 h post-inoculation (direction of comparison: WNV variant / MOCK). Significantly affected genes (FDR (false discovery rate) <

0.05 and $\log_2$ FC (fold change) ≥ 0.5 (1.42×)) were summarized in **S4 Table**. Second, significant DEGs from each experimental condition were compared with a curated set of 194 interferon-signaling genes (**S4 Table**) obtained from Harmonizome 3.0 (https://maayanlab.cloud/Harmonizome/gene_set/Interferon+Signaling/Reactome+Pathways+2014), yielding variant- and time-specific interferon-response profiles. The DEGs significantly affected in each condition that are part of the interferon signaling pathway were summarized in **S4 Table** and visualized in **Fig 7** with pairwise comparisons.

Western blot

Huh7 cells were synchronously inoculated with 1,000 RNA genome copies per cell of WNV variants (or mock-inoculated), as determined by RT-qPCR targeting the UTR, which was unmodified in all WNV variants. We normalized inoculum based on viral RNA copies to specifically investigate the effects of dinucleotide-enriched RNA on innate immune responses during early infection. Following a 2-hour incubation at 37°C, the inoculum was removed, the cells were washed with 1× PBS, and fresh DMEM with 2% FBS was added. After inoculation cells were then incubated at 37°C for 6 h. Cell lysis and Western blot were performed according to our previous protocol (1, 2). Cells were washed, pelleted, and lysed in 150 µL of RIPA buffer (Fisher, MA, USA; # PI89900) supplemented with 1× Halt protease inhibitor cocktail (Fisher, MA, USA; #PI87786) and 1× EDTA (Fisher, MA, USA; #BP2482100). After incubation on ice and centrifugation (12,000 g, 10 min, 4°C), protein concentrations were quantified using the Pierce BCA Protein Assay Kit (Fisher, #PI23225). Protein samples (25 µg for RIG I; 50 µg for ZAP and DDX50) were denatured, resolved by 10% SDS-PAGE (Mini-PROTEAN TGX gels, Bio-Rad, #4561036), and transferred onto methanol-activated 0.45 µm LF PVDF membranes (Bio-Rad,

#1620260). Membranes were blocked with Rapidblock Blocking Solution (VWR, #97064-124), incubated overnight at 4°C with primary antibodies (Rabbit anti-ZAP IgG Ab (1:1,000 dilution; ANTI-ZC3HAV1 #HPA059096-100UL, Millipore Sigma, MA, USA), Rabbit anti-DDX50 IgG Ab (1:1,000 dilution; #10358-1-AP; ProteinTech, IL, USA), Rabbit anti-RIG-I/DDX58 IgG Ab (Abcam, ab180675)) and subsequently with hFAB™ Rhodamine Anti-Actin Primary Abs (1:3,000; #12004164; Bio-Rad, CA, USA) and IRDye® 680RD Goat anti-Rabbit IgG secondary Abs (1:10,000; #926-68071; LI-COR, NE, USA).

Fluorescence imaging for ZAP (100 kDa), DDX50 (83 kDa), RIG-I (107 kDa), and β-actin (42 kDa) and densitometric analyses were performed using the ChemiDoc MP Imaging system (Bio-Rad, CA., USA) and Image Lab Software 6.1 (Bio-Rad, CA., USA), with intensities normalized to β-actin controls according to the Bio-Rad user guide instructions and our previous brief description (1, 2). Briefly, the "Lanes and Bands" option was used to select the specific lanes and bands corresponding to the protein of interest. The "Lane Profile" option was used to subtract the background. In the "Analysis Table," the background-adjusted (“Adj. Volume”) intensities of each ZAP, DDX50, or RIG-I band were normalized by the background-adjusted β-actin intensities. Subsequently, the ZAP, DDX50, or RIG-I to β-actin ratios were used to analyze the data for each experimental condition.

Infectious virus titration

Infectious viral loads for WNV stocks were quantified using an endpoint dilution assay to determine the $TCID_{50}$ performed in two biological replicates as previously described (9, 17-21). Viral stocks were serially diluted in four technical replicates in DMEM supplemented with 1%

FBS. Then, 50 µl of each dilution were inoculated into confluent VERO-ZAP-WT or VERO-ZAP-KO cells cultured in 96-well plates. After 2h of incubation at 37°C, 150 µl of media was added per well. The cells were incubated for 5 days at 37°C in a humidified atmosphere in 5% CO_2 incubator. Afterward, cells were washed, dried, formalin-fixed, and stained with specific primary anti-pan flavivirus envelope (E) protein monoclonal Ab (mAb) D1-4G2-4-15 (ATCC, VA, USA; #HB-112) followed by Goat anti-Mouse IgG HRP Ab (Abcam, MA, USA; #ab97023). (9, 17-21). We observed that introducing two amino acid substitutions in the E protein of the WNV-WT+FR and E-MAX-FR variants reduced the staining signal of the D1-4G2-4-15 mAbs, which specifically binds to the E protein. Thus, for the WNV-WT+FR and E-MAX-FR stock titration, we used mouse monoclonal anti-West Nile Virus nonstructural protein 1 Ab, Clone 22-NS1 (BEI Resources, # NR-10145). Infectious titers were calculated using the Spearman-Kärber formula and expressed as $\log_{10}$ $TCID_{50}$ per ml. Cells mock-inoculated with media served as negative controls.

RNA extraction and virus-specific reverse transcriptase quantitative polymerase chain reaction assays (RT-qPCR)

The RNA from cell culture supernatants was extracted with QIAamp Viral RNA Mini Kit (QIAGEN) according to the manufacturer's instructions. The RNA from mouse tissues or from cell homogenates in TRI Reagent Solution was extracted using the PureLink RNA Mini Kit (Fisher, #12-183-018A) according to the manufacturer's instructions.

All RT-qPCR reactions were conducted on the QuantStudio 3 real-time PCR system (Applied Biosystems) and analyzed using QuantStudio Design & Analysis Software 1.5.2. We used the previously validated probe-based one-step RT-qPCR assays with specific WNV primers (Forward: 5′-AGTAGTTCGCCTGTGTGAGC-3′; Reverse: 5′-GCCCTCCTGGTTTCTTAGA-3′; Probe:

FAM-AATCCTCACAAACACTACTAAGTTTGTCA-TAMRA) following validated Probe-based one-step RT-qPCR assay (31). The Luna Universal Probe One-Step RT-qPCR Kit (NEB) reaction mixture (20 μL) consisted of 10 μL Luna Universal Probe One-Step Reaction Mix, 1 μL Luna WarmStart RT Enzyme Mix, 1 μL of 10 μM dilution of forward and reverse primers, 0.5 μL of the probe, 2.5 μL nuclease-free water and 4 μL of RNA template. The reverse transcription (10 minutes at 55°C) and enzyme activation steps (1 minute at 95°C) were followed by 40 amplification cycles (10 s at 95°C and 30 s at 60°C). A standard curve was used to quantify viral RNA loads as we previously described (17).

PCR values were normalized to fluid volume or tissue weight, $\log_{10}$-transformed, and reported as virus RNA genome copies per ml or g. Strict precautions were taken to prevent PCR contamination. All master mix preparations were done in the dedicated PCR cabinet. Aerosol-resistant filter pipette tips and disposable gloves were used. Kit reagent controls were included in every RNA extraction and PCR run.

Virus neutralization (VN) assay and 50% plaque reduction neutralization test ($PRNT_{50}$)

WNV-neutralizing Abs in mouse serum were quantified using a serum neutralization assay (35). This assay was previously validated against the traditional plaque reduction neutralization test for quantifying WNV antibodies by comparing approximately 1,400 serum samples, which showed no statistically significant differences (35). Also, we previously used this VN assay in flavivirus studies (21). Briefly, serum samples were heat-inactivated at 56°C for 30 minutes. Afterward, sera were diluted in media through 2-fold serial dilutions, starting from either 1:10 or 1:20, with each dilution performed in duplicate. Following dilution, 50 μl of the WNV-WT NY99 strain (100

$TCID_{50}$/well; ISA-derived) were added to the samples, mixed, and incubated at +37 °C for 1h. Afterward, 10^5 wild-type Vero cell suspended in 100 µl of DMEM supplemented with 10% FBS, 1x Penicillin-Streptomycin, and 2.67 mM Sodium Bicarbonate were added into each well. After five days, cells were fixed and stained with flavivirus-specific D1-4G2-4-15 (ATCC) Abs as described above. The neutralizing Ab titers were expressed as the highest serum dilution capable of inhibiting WNV infection in 50% of the inoculated wells.

Sera from mice immunized with the E-MAX-FR variant (**Fig 10F**) or mock-immunized controls were used to quantify nAb titers by the 50% plaque reduction neutralization test ($PRNT_{50}$) (35-37). Equal volumes (150 µl) of WNV-WT NY99 suspension (10^3 $TCID_{50}$/well; ISA-derived) and serial twofold dilutions of heat-inactivated serum were incubated for 1 h at 37 °C. The serum–virus mixtures were then inoculated onto wild-type Vero cell monolayers (two replicates) in 12-well plates. After 1 h of adsorption at 37 °C, the inoculum was removed and replaced with an overlay containing 2% Invitrogen UltraPure Low Melting Point Agarose (Fisher) mixed with DMEM supplemented with 10% FBS, yielding final concentrations of 1.3% agarose and 3.3% FBS. Cultures were incubated for four days at 37 °C before fixation with 10% formalin and staining with crystal violet for plaque counting. Neutralization titers were defined as the reciprocal of the highest serum dilution that reduced virus infectivity by 50% compared with the average plaque counts from virus-only control wells (two replicates). Controls confirmed that sera from mock-immunized mice did not reduce plaque counts compared with virus-only control wells.

Supplemental materials and methods

ELISpot assay

The ELISpot assay was performed as previously described (21). Freshly isolated mouse splenocytes (5×10^5 cells/well) were seeded in RPMI medium supplemented with 10% FBS, 1× MEM Non-Essential Amino Acids (Gibco, #11140-050), 1× Penicillin-Streptomycin, 50 µg/ml Gentamycin, 10 mM HEPES (MP Biomedicals, #1688449), 1 mM Sodium Pyruvate (Gibco, #11360-070), and 50 µM 2-mercaptoethanol. Cells were stimulated for 20 h at 37°C with 5% CO_2 using a 67-peptide pool (1.5 µg/ml per peptide; BEI Resources, #NR-435) spanning the WNV E protein from the NY99-flamingo382-99 strain [GenBank: AF196835]. Interferon-gamma-secreting cells (IFNγ-SC) were quantified from duplicate wells using a murine IFNγ ELISpot kit (Diaclone, #862.031.001S) and analyzed with the C.T.L scanner and ImmunoSpot 7.0 Pro DC Analyzer software (Cellular Technology Limited). Unstimulated splenocytes treated with DMSO (the peptide solvent) were used as a control for the background signal.

Histopathology

Mouse brain tissues (the left hemisphere) were fixed in 10% formalin, processed, and embedded in paraffin. The brain sections were stained with hematoxylin and eosin (H&E) for histopathological examination. Histopathological evaluation was adapted from previous WNV studies (38-42).

Statistics

Statistical analyses were performed using GraphPad Prism 10.3.1. For different datasets, we used the unpaired t-test, one-way ANOVA, or two-way ANOVA. Specific statistical tests applied are

indicated within each figure legend. A *p*-value ≤ 0.05 was interpreted as statistically significant. All results are presented as mean ± standard error of the mean (SEM).

Supplemental references

1. Le NPK, Singh PP, Sabir AJ, Trus I, Karniychuk U. 2024. Endogenous ZAP is associated with altered Zika virus infection phenotype. Virol J 21:285.
2. Sabir AJ, Le NPK, Singh PP, Karniychuk U. 2024. Endogenous ZAP affects Zika virus RNA interactome. RNA Biol 21:1-10.
3. Singh PP, Le NPK, Karniychuk U. 2025. Infectious Subgenomic Amplicon Strategies for Japanese Encephalitis and West Nile Viruses. J Med Virol 97:e70205.
4. Aubry F, Nougairède A, de Fabritus L, Querat G, Gould EA, de Lamballerie X. 2014. Single-stranded positive-sense RNA viruses generated in days using infectious subgenomic amplicons. The Journal of general virology 95:2462-7.
5. Tran Thi Nhu Thao FL, Nadine Ebert, Philip V'kovski, Hanspeter Stalder, Jasmine Portmann, Jenna Kelly, Silvio Steiner, Melle Holwerda, Annika Kratzel, Mitra Gultom, Kimberly Schmied, Laura Laloli, Linda Hüsser, Manon Wider, Stephanie Pfaen, Volker Thiel. 2020. Rapid reconstruction of SARS-CoV-2 using a synthetic genomics platform. Nature 582:561-565.
6. Park CJ, Kim T, Yoo SM, Lee MS, Cho NH, Park C. 2025. Efficiency of reverse genetics methods for rescuing severe acute respiratory syndrome coronavirus 2. J Microbiol 63:e2411023.
7. Yun SM, Lee TY, Lim HY, Ryou J, Lee JY, Kim YE. 2021. Development and Characterization of a Reverse Genetics System for a Human-Derived Severe Fever With Thrombocytopenia Syndrome Virus Isolate From South Korea. Front Microbiol 12:772802.
8. Bolger AM, Lohse M, Usadel B. 2014. Trimmomatic: a flexible trimmer for Illumina sequence data. Bioinformatics 30:2114-20.
9. Sabir AJ, Singh PP, Trus I, Le NPK, Karniychuk U. 2023. Asian Zika virus can acquire generic African-lineage mutations during in utero infection. Emerg Microbes Infect 12:2263592.
10. Udenze D, Trus I, Berube N, Karniychuk U. 2022. CpG content in the Zika virus genome affects infection phenotypes in the adult brain and fetal lymph nodes. Front Immunol 13:943481.
11. Udenze D, Trus I, Munyanduki H, Berube N, Karniychuk U. 2021. The isolated in utero environment is conducive to the emergence of rna and dna virus variants. Viruses 13:1827.
12. Okonechnikov K, Golosova O, Fursov M, team U. 2012. Unipro UGENE: a unified bioinformatics toolkit. Bioinformatics 28:1166-7.
13. Koboldt DC, Zhang Q, Larson DE, Shen D, McLellan MD, Lin L, Miller CA, Mardis ER, Ding L, Wilson RK. 2012. VarScan 2: somatic mutation and copy number alteration discovery in cancer by exome sequencing. Genome research 22:568-76.
14. Grubaugh ND, Gangavarapu K, Quick J, Matteson NL, De Jesus JG, Main BJ, Tan AL, Paul LM, Brackney DE, Grewal S, Gurfield N, Van Rompay KKA, Isern S, Michael SF, Coffey LL, Loman NJ, Andersen KG. 2019. An amplicon-based sequencing framework for accurately measuring intrahost virus diversity using PrimalSeq and iVar. Genome Biol 20:8.
15. Metsky HC, Matranga CB, Wohl S, Schaffner SF, Freije CA, Winnicki SM, West K, Qu J, Baniecki ML, Gladden-Young A, Lin AE, Tomkins-Tinch CH, Ye SH, Park DJ, Luo CY, Barnes KG, Shah RR, Chak B, Barbosa-Lima G, Delatorre E, Vieira YR, Paul LM, Tan AL, Barcellona CM, Porcelli MC, Vasquez C, Cannons AC, Cone MR, Hogan KN, Kopp EW, Anzinger JJ, Garcia KF, Parham LA, Ramirez RMG, Montoya MCM, Rojas DP, Brown CM, Hennigan S, Sabina B, Scotland S,

Gangavarapu K, Grubaugh ND, Oliveira G, Robles-Sikisaka R, Rambaut A, Gehrke L, Smole S, Halloran ME, Villar L, Mattar S, et al. 2017. Zika virus evolution and spread in the Americas. Nature 546:411-415.

16. Grubaugh ND, Ladner JT, Kraemer MUG, Dudas G, Tan AL, Gangavarapu K, Wiley MR, White S, Theze J, Magnani DM, Prieto K, Reyes D, Bingham AM, Paul LM, Robles-Sikisaka R, Oliveira G, Pronty D, Barcellona CM, Metsky HC, Baniecki ML, Barnes KG, Chak B, Freije CA, Gladden-Young A, Gnirke A, Luo C, MacInnis B, Matranga CB, Park DJ, Qu J, Schaffner SF, Tomkins-Tinch C, West KL, Winnicki SM, Wohl S, Yozwiak NL, Quick J, Fauver JR, Khan K, Brent SE, Reiner RC, Jr., Lichtenberger PN, Ricciardi MJ, Bailey VK, Watkins DI, Cone MR, Kopp EWt, Hogan KN, Cannons AC, Jean R, et al. 2017. Genomic epidemiology reveals multiple introductions of Zika virus into the United States. Nature 546:401-405.

17. Chapagain S, Pal Singh P, Le K, Safronetz D, Wood H, Karniychuk U. 2022. Japanese encephalitis virus persists in the human reproductive epithelium and porcine reproductive tissues. PLoS Negl Trop Dis 16:e0010656.

18. Darbellay J, Lai K, Babiuk S, Berhane Y, Ambagala A, Wheler C, Wilson D, Walker S, Potter A, Gilmour M, Safronetz D, Gerdts V, Karniychuk U. 2017. Neonatal pigs are susceptible to experimental Zika virus infection. Emerg Microbes Infect 6:e6.

19. Darbellay J, Cox B, Lai K, Delgado-Ortega M, Wheler C, Wilson D, Walker S, Starrak G, Hockley D, Huang Y, Mutwiri G, Potter A, Gilmour M, Safronetz D, Gerdts V, Karniychuk U. 2017. Zika Virus Causes Persistent Infection in Porcine Conceptuses and may Impair Health in Offspring doi:10.1016/j.ebiom.2017.09.021.

20. Trus I, Udenze D, Cox B, Berube N, Nordquist RE, van der Staay FJ, Huang Y, Kobinger G, Safronetz D, Gerdts V, Karniychuk U. 2019. Subclinical in utero Zika virus infection is associated with interferon alpha sequelae and sex-specific molecular brain pathology in asymptomatic porcine offspring, vol 15, p e1008038.

21. Trus I, Udenze D, Berube N, Wheler C, Martel MJ, Gerdts V, Karniychuk U. 2019. CpG-Recoding in Zika Virus Genome Causes Host-Age-Dependent Attenuation of Infection With Protection Against Lethal Heterologous Challenge in Mice. Front Immunol 10:3077.

22. Trus I, Berube N, Jiang P, Rak J, Gerdts V, Karniychuk U. 2020. Zika Virus with Increased CpG Dinucleotide Frequencies Shows Oncolytic Activity in Glioblastoma Stem Cells, vol 12, p 579. Multidisciplinary Digital Publishing Institute.

23. Udenze D, Trus I, Berube N, Gerdts V, Karniychuk U. 2019. The African strain of Zika virus causes more severe in utero infection than Asian strain in a porcine fetal transmission model, vol 8.

24. Saito K, Fukasawa M, Shirasago Y, Suzuki R, Osada N, Yamaji T, Wakita T, Konishi E, Hanada K. 2020. Comparative characterization of flavivirus production in two cell lines: Human hepatoma-derived Huh7.5.1-8 and African green monkey kidney-derived Vero. PLoS One 15:e0232274.

25. Schwerk J, Soveg FW, Ryan AP, Thomas KR, Hatfield LD, Ozarkar S, Forero A, Kell AM, Roby JA, So L, Hyde JL, Gale M, Jr., Daugherty MD, Savan R. 2019. RNA-binding protein isoforms ZAP-S and ZAP-L have distinct antiviral and immune resolution functions. Nat Immunol 20:1610-1620.

26. Chen X, Saccon E, Appelberg KS, Mikaeloff F, Rodriguez JE, Vinhas BS, Frisan T, Vegvari A, Mirazimi A, Neogi U, Gupta S. 2021. Type-I interferon signatures in SARS-CoV-2 infected Huh7 cells. Cell Death Discov 7:114.

27. Saito T, Hirai R, Loo YM, Owen D, Johnson CL, Sinha SC, Akira S, Fujita T, Gale M, Jr. 2007. Regulation of innate antiviral defenses through a shared repressor domain in RIG-I and LGP2. Proc Natl Acad Sci U S A 104:582-7.

28. Jia L, Chen Z, Zhang Y, Ma L, Wang L, Hu X, Liu H, Chen J, Liu D, Guan W. 2021. Suppression and Activation of Intracellular Immune Response in Initial Severe Acute Respiratory Syndrome Coronavirus 2 Infection. Front Microbiol 12:768740.

29. Geerling E, Pinski AN, Stone TE, DiPaolo RJ, Zulu MZ, Maroney KJ, Brien JD, Messaoudi I, Pinto AK. 2022. Roles of antiviral sensing and type I interferon signaling in the restriction of SARS-CoV-2 replication. iScience 25:103553.
30. Atkinson NJ, Witteveldt J, Evans DJ, Simmonds P. 2014. The influence of CpG and UpA dinucleotide frequencies on RNA virus replication and characterization of the innate cellular pathways underlying virus attenuation and enhanced replication. Nucleic acids research 42:4527-45.
31. Eiden M, Vina-Rodriguez A, Hoffmann B, Ziegler U, Groschup MH. 2010. Two new real-time quantitative reverse transcription polymerase chain reaction assays with unique target sites for the specific and sensitive detection of lineages 1 and 2 West Nile virus strains. J Vet Diagn Invest 22:748-53.
32. Shao N, Li F, Nie K, Fu SH, Zhang WJ, He Y, Lei WW, Wang QY, Liang GD, Cao YX, Wang HY. 2018. TaqMan Real-time RT-PCR Assay for Detecting and Differentiating Japanese Encephalitis Virus, vol 31, p 208-214. Biomed Environ Sci.
33. Udenze D, Trus I, Lipsit S, Napper S, Karniychuk U. 2022. Offspring affected with in utero Zika virus infection retain molecular footprints in the bone marrow and blood cells doi:10.1080/22221751.2022.2147021, p 1-43. Emerg Microbes Infect.
34. Bray NL, Pimentel H, Melsted P, Pachter L. 2016. Near-optimal probabilistic RNA-seq quantification. Nature Biotechnology 34:525-527.
35. Di Gennaro A, Lorusso A, Casaccia C, Conte A, Monaco F, Savini G. 2014. Serum neutralization assay can efficiently replace plaque reduction neutralization test for detection and quantitation of West Nile virus antibodies in human and animal serum samples. Clin Vaccine Immunol 21:1460-2.
36. Roehrig JT, Hombach J, Barrett AD. 2008. Guidelines for Plaque-Reduction Neutralization Testing of Human Antibodies to Dengue Viruses. Viral Immunol 21:123-32.
37. Arroyo J, Miller C, Catalan J, Myers GA, Ratterree MS, Trent DW, Monath TP. 2004. ChimeriVax-West Nile virus live-attenuated vaccine: preclinical evaluation of safety, immunogenicity, and efficacy. J Virol 78:12497-507.
38. Roe K, Kumar M, Lum S, Orillo B, Nerurkar VR, Verma S. 2012. West Nile virus-induced disruption of the blood-brain barrier in mice is characterized by the degradation of the junctional complex proteins and increase in multiple matrix metalloproteinases. J Gen Virol 93:1193-1203.
39. Shrestha B, Gottlieb D, Diamond MS. 2003. Infection and injury of neurons by West Nile encephalitis virus. J Virol 77:13203-13.
40. Garcia-Tapia D, Hassett DE, Mitchell WJ, Jr., Johnson GC, Kleiboeker SB. 2007. West Nile virus encephalitis: sequential histopathological and immunological events in a murine model of infection. J Neurovirol 13:130-8.
41. Chambers TJ, Diamond MS. 2003. Pathogenesis of flavivirus encephalitis. Adv Virus Res 60:273-342.
42. Cho H, Diamond MS. 2012. Immune responses to West Nile virus infection in the central nervous system. Viruses 4:3812-30.
