# Supplementary figures and images for: West Nile virus vaccine candidates attenuated by dinucleotide enrichment are immunogenic and protective against lethal infection
Source: PLoS Pathog. 2025 Oct 3;21(10):e1013560. doi: 10.1371/journal.ppat.1013560 (PMC12513643; doi:10.1371/journal.ppat.1013560)

Fig S1

WNV-WT+FVR (working stock)

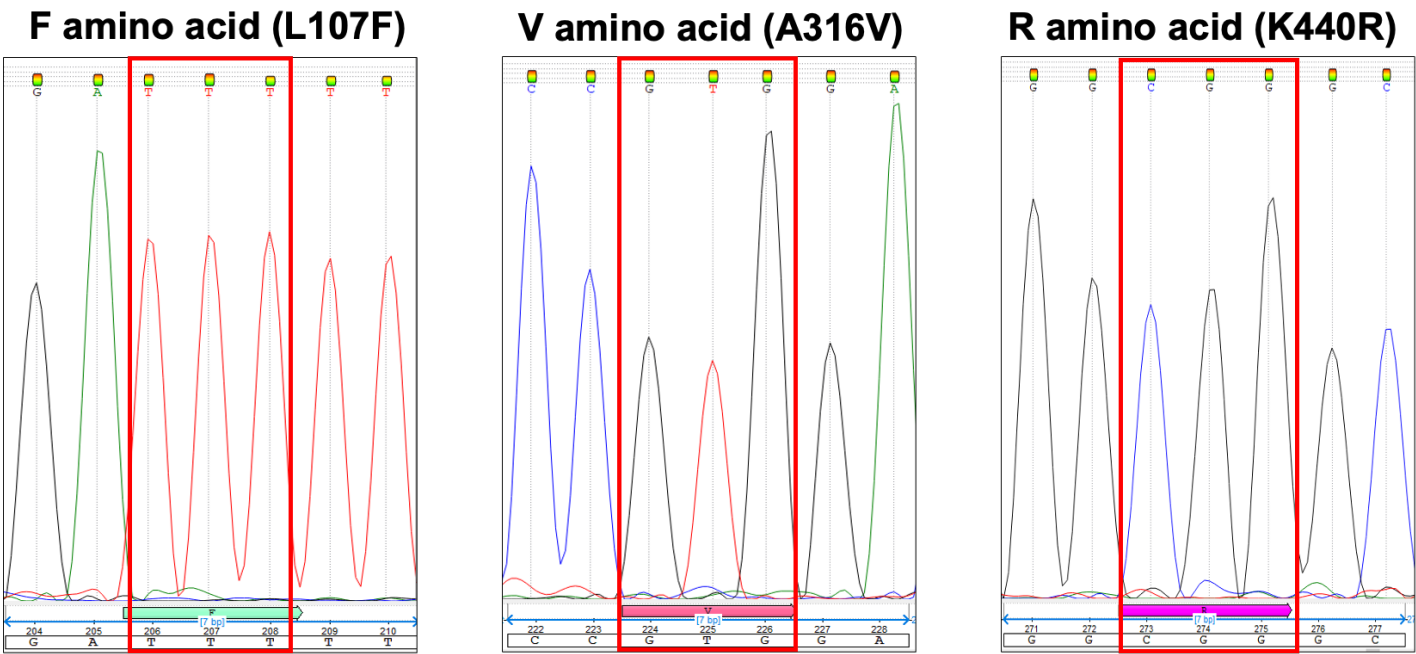

E-MAX+FVR (working stock)

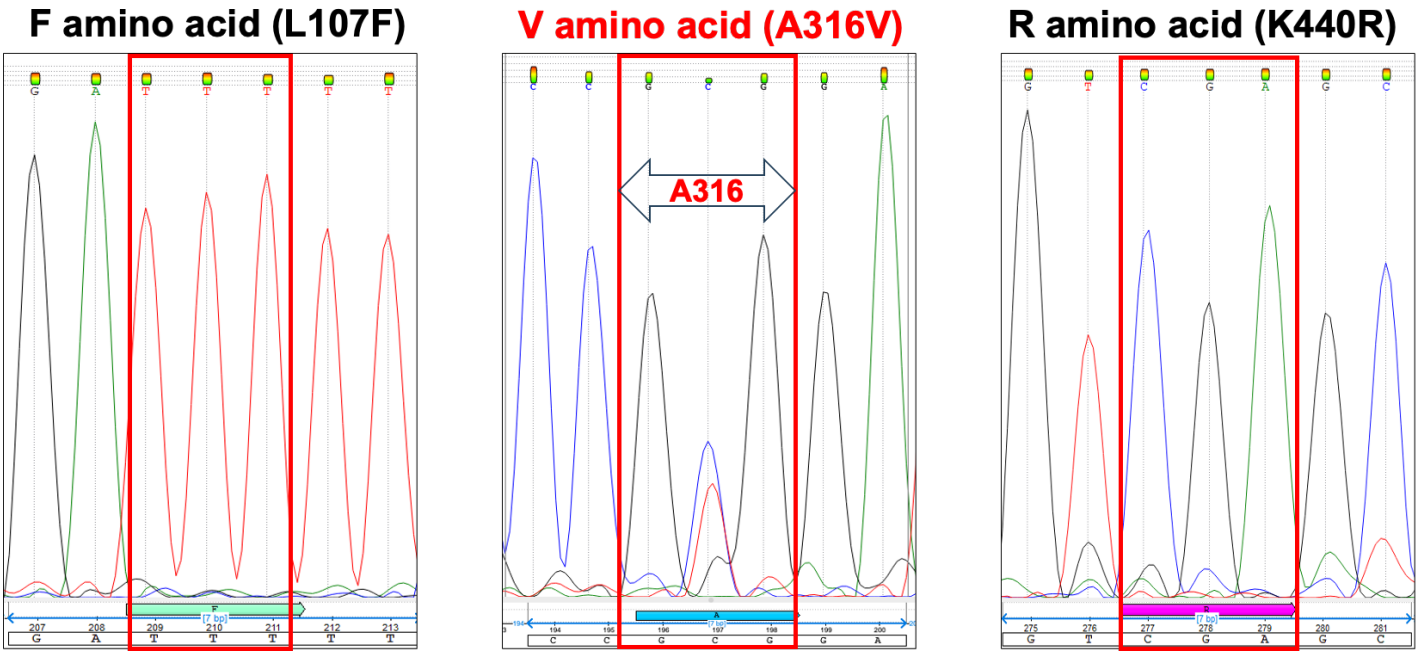

Supplement: S1 Fig — Sanger sequencing revealed that the A316V mutation was not stable and reverted to wild-type A in the E-MAX+ FVR stock. (PDF) [file ppat.1013560.s002.pdf]

Fig S2

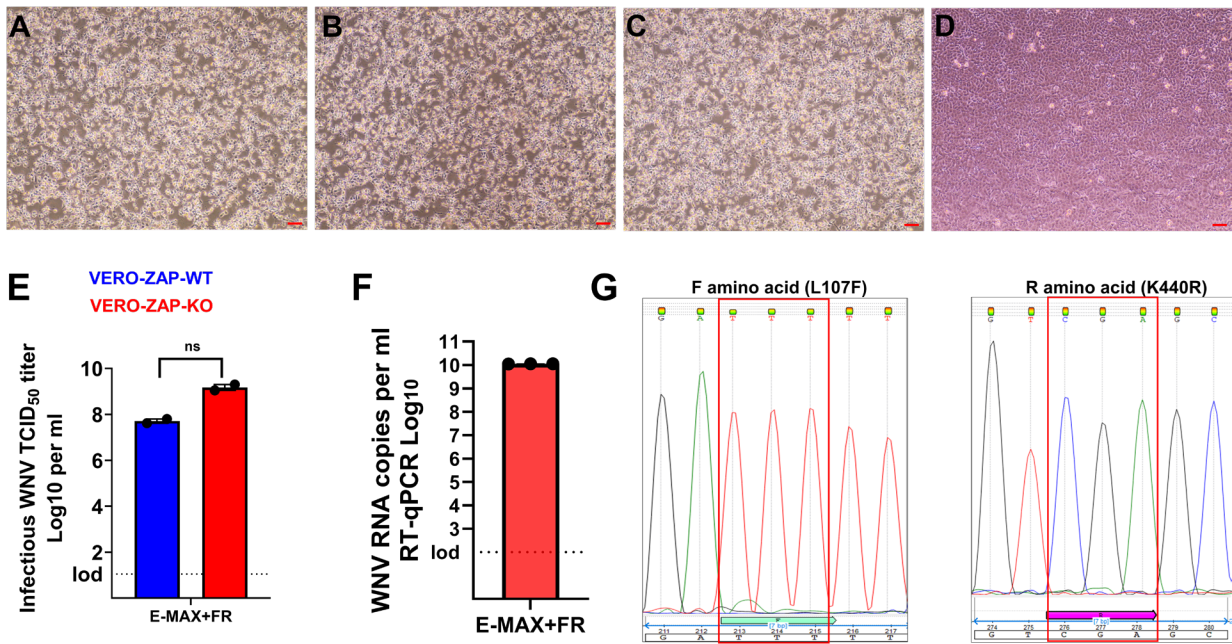

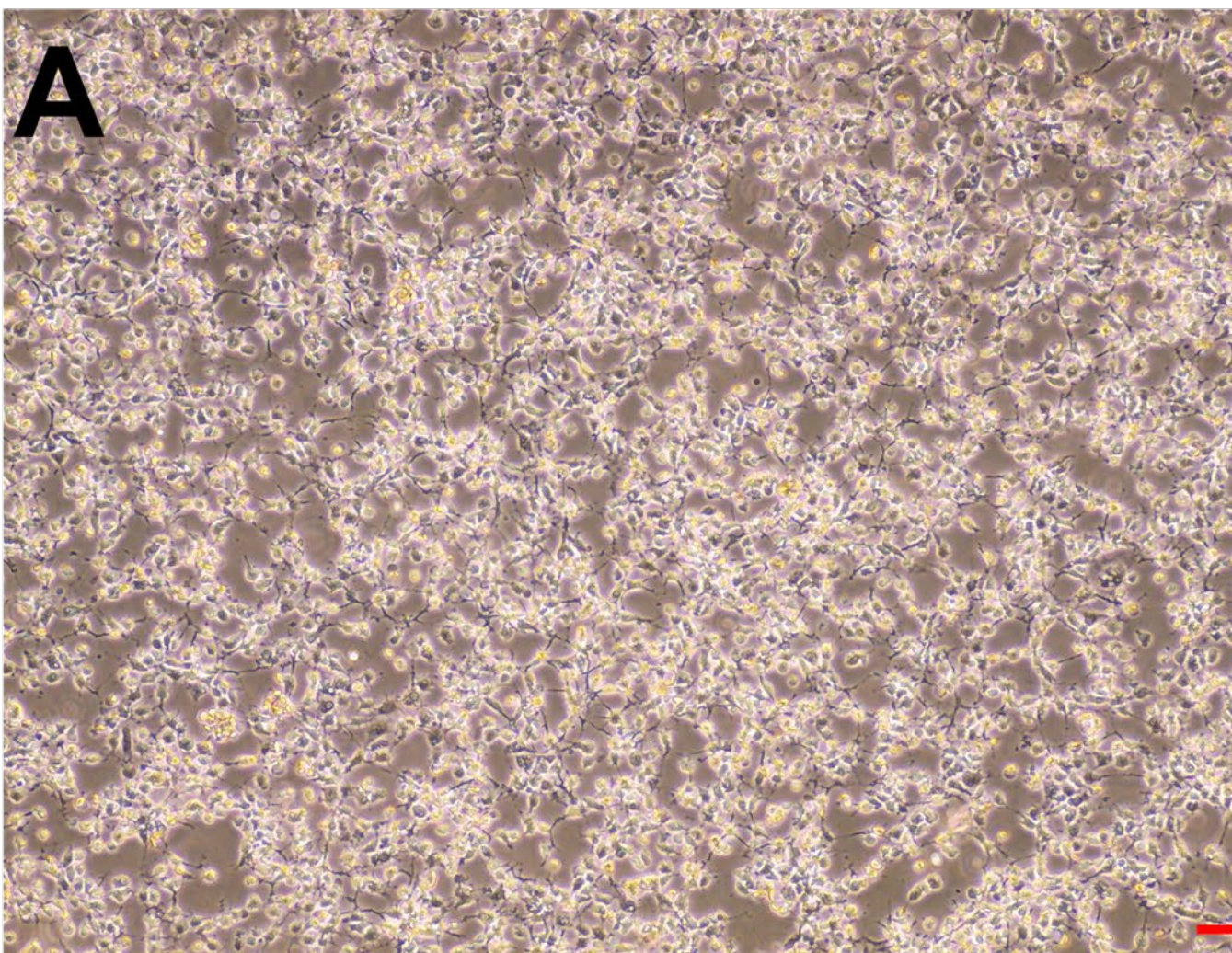

**E**

**VERO-ZAP-WT**

**VERO-ZAP-KO**

**OD<sub>50</sub> titer**

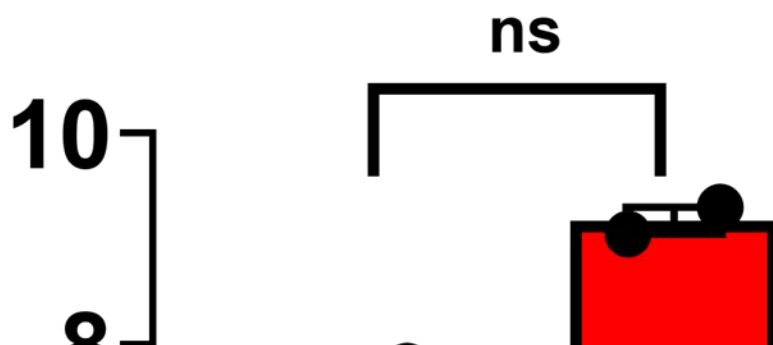

Supplement: S2 Fig — For ISA transfections, we initially used a combination of ZAP-KO BHK-21 and Vero cells, a standard approach to enhance transfection efficiency. Subsequently, we repeated the rescue of E-MAX+ FR, the most promising vaccine candidate in this study, using only VERO-ZAP-KO cells. Representative images of cytopathic effect (CPE) in VERO-ZAP-KO cells transfected with ISA DNA fragments representing E-MAX+ FR or mock (A-D). Magnification ×100. Scale bar: 100 µm. ISA transfection was performed as described in S1 Supplemental Materials and Methods, but with DNA fragments mixed in equimolar concentrations to obtain a total of 3 µg of DNA per transfection well. Transfections were carried out in 6-well plates with five replicates and one mock-transfected control well (D). CPE and infectious E-MAX+ FR were observed in three (A-C) out of five wells. (E) Infectious titers of the E-MAX+ FR stock produced in only VERO-ZAP-KO cells and titrated in ZAP-WT or ZAP-KO Vero cells. lod: limit of detection. ns: Unpaired t-test: p = 0.3333. (F) RNA loads of the E-MAX+ FR stock produced in VERO-ZAP-KO cells. lod: limit of detection. (G) Sanger sequencing confirmed the presence of the introduced L107F and K440R substitutions in the E-MAX+ FR variant rescued in only VERO-ZAP-KO cells. Nucleotide peaks encoding these substitutions are highlighted in red squares. Reference sequences are provided in S1 File. (PDF) [file ppat.1013560.s003.pdf]
