# Supplementary material for: West Nile virus vaccine candidates attenuated by dinucleotide enrichment are immunogenic and protective against lethal infection
Source: PLoS Pathog. 2025 Oct 3;21(10):e1013560. doi: 10.1371/journal.ppat.1013560 (PMC12513643; doi:10.1371/journal.ppat.1013560)
Supplement: S1 File — (PDF) [file ppat.1013560.s004.pdf]

File S1 WNV reference sequences

> WNV-WT

DQ211652.1 West Nile virus strain NY99, complete genome

AGTAGTTCGCCTGTGTGAGCTGACAAACTTAGTAGTGTTTGTGAGGATTAACAACAATTAAC
ACAGTGCGAGCTGTTTCTTAGCACGAAGATCTCGATGTCTAAGAAACCAGGAGGGCCCGGC
AAGAGCCGGGCTGTCAATATGCTAAAACGCGGAATGCCCCGCGTGTTGTCCTTGATTGGAC
TGAAGAGGGCTATGTTGAGCCTGATCGACGGCAAGGGGCCAATACGATTTGTGTTGGCTCT
CTTGGCGTTCTTCAGGTTCACAGCAATTGCTCCGACCCGAGCAGTGCTGGATCGATGGAGA
GGTGTGAACAAACAAACAGCGATGAAACACCTTCTGAGTTTTAAGAAGGAACTAGGGACCT
TGACCAGTGCTATCAATCGGCGGAGCTCAAAACAAAAGAAAAGAGGAGGAAAGACCGGAAT
TGCAGTCATGATTGGCCTGATCGCCAGCGTAGGAGCAGTTACCCTCTCTAACTTCCAAGGG
AAGGTGATGATGACGGTAAATGCTACTGACGTCACAGATGTCATCACGATTCCAACAGCTGC
TGGAAAGAACCTATGCATTGTCAGAGCAATGGATGTGGGATACATGTGCGATGATACTATCA
CTTATGAATGCCCAGTACTGTCGGCTGGTAATGATCCAGAAGACATCGACTGTTGGTGCACA
AAGTCAGCAGTCTACGTCAGGTATGGAAGATGCACCAAGACACGCCACTCAAGACGCAGTC
GGAGGTCACTGACAGTGCAGACACACGGAGAAAGCACTCTAGCGAACAAGAAGGGGGCTT
GGATGGACAGCACCAAGGCCACAAGGTATTTGGTAAAAACAGAATCATGGATCTTGAGGAA
CCCTGGATATGCCCTGGTGGCAGCCGTCATTGGTTGGATGCTTGGGAGCAACACCATGCA
GAGAGTTGTGTTTGTCGTGCTATTGCTTTTGGTGGCCCCAGCTTACAGCTTCAACTGCCTTG
GAATGAGCAACAGAGACTTCTTGGAAGGAGTGTCTGGAGCAACATGGGTGGATTTGGTTCT
CGAAGGCGACAGCTGCGTGACTATCATGTCTAAGGACAAGCCTACCATCGATGTGAAGATG
ATGAATATGGAGGCGGCCAACCTGGCAGAGGTCCGCAGTTATTGCTATTTGGCTACCGTCA
GCGATCTCTCCACCAAAGCTGCGTGCCCGACCATGGGAGAAGCTCACAATGACAAACGTG
CTGACCCAGCTTTTGTGTGCAGACAAGGAGTGGTGGACAGGGGCTGGGGCAACGGCTGC
GGACTATTTGGCAAAGGAAGCATTGACACATGCGCCAAATTTGCCTGCTCTACCAAGGCAAT
AGGAAGAACCATCTTGAAAGAGAATATCAAGTACGAAGTGGCCATTTTTGTCCATGGACCAA
CTACTGTGGAGTCGCACGGAAACTACTCCACACAGGTTGGAGCCACTCAGGCAGGGAGAC
TCAGCATCACTCCTGCGGCGCCTTCATACACACTAAAGCTTGGAGAATATGGAGAGGTGAC
AGTGGACTGTGAACCACGGTCAGGGATTGACACCAATGCATACTACGTGATGACTGTTGGA
ACAAAGACGTTCTTGGTCCATCGTGAGTGGTTCATGGACCTCAACCTCCCTTGGAGCAGTG
CTGGAAGTACTGTGTGGAGGAACAGAGAGACGTTAATGGAGTTTGAGGAACCACACGCCA
CGAAGCAGTCTGTGATAGCATTGGGCTCACAAGAGGGAGCTCTGCATCAAGCTTTGGCTGG
AGCCATTCCTGTGGAATTTTCAAGCAACACTGTCAAGTTGACGTCGGGTCATTTGAAGTGTA
GAGTGAAGATGGAAAAATTGCAGTTGAAGGGAACAACCTATGGCGTCTGTTCAAAGGCTTT
CAAGTTTCTTGGGACTCCCGCAGACACAGGTCACGGCACTGTGGTGTTGGAATTGCAGTAC
ACTGGCACGGATGGACCTTGCAAAGTTCCTATCTCGTCAGTGGCTTCATTGAACGACCTAA
CGCCAGTGGGCAGATTGGTCACTGTCAACCCTTTTGTTTCAGTGGCCACGGCCAACGCTAA
GGTCCTGATTGAATTGGAACCACCCTTTGGAGACTCATACATAGTGGTGGGCAGAGGAGAA
CAACAGATCAATCACCATTGGCACAAGTCTGGAAGCAGCATTGGCAAAGCCTTTACAACCA
CCCTCAAAGGAGCGCAGAGACTAGCCGCTCTAGGAGACACAGCTTGGGACTTTGGATCAG
TTGGAGGGGTGTTCACCTCAGTTGGGAAGGCTGTCCATCAAGTGTTCGGAGGAGCATTCC
GCTCACTGTTCGGAGGCATGTCCTGGATAACGCAAGGATTGCTGGGGGCTCTCCTGTTGTG
GATGGGCATCAATGCTCGTGATAGGTCCATAGCTCTCACGTTTCTCGCAGTTGGAGGAGTT
CTGCTCTTCCTCTCCGTGAACGTGCACGCTGACACTGGGTGTGCCATAGACATCAGCCGG
CAAGAGCTGAGATGTGGAAGTGGAGTGTTCATACACAATGATGTGGAGGCTTGGATGGACC
GGTACAAGTATTACCCTGAAACGCCACAAGGCCTAGCCAAGATCATTCAGAAAGCTCATAAG
GAAGGAGTGTGCGGTCTACGATCAGTTTCCAGACTGGAGCATCAAATGTGGGAAGCAGTGA

File S1 WNV reference sequences

AGGACGAGCTGAACACTCTTTTGAAGGAGAATGGTGTGGACCTTAGTGTCGTGGTTGAGAA
ACAGGAGGGAATGTACAAGTCAGCACCTAAACGCCTCACCGCCACCACGGAAAAATTGGAA
ATTGGCTGGAAGGCCTGGGGAAAGAGTATTTTATTTGCACCAGAACTCGCCAACAACACCT
TTGTGGTTGATGGTCCGGAGACCAAGGAATGTCCGACTCAGAATCGCGCTTGGAATAGCTT
AGAAGTGGAGGATTTTGGATTTGGTCTCACCAGCACTCGGATGTTCCTGAAGGTCAGAGAG
AGCAACACAACTGAATGTGACTCGAAGATCATTGGAACGGCTGTCAAGAACAACTTGGCGA
TCCACAGTGACCTGTCCTATTGGATTGAAAGCAGGCTCAATGATACGTGGAAGCTTGAAAG
GGCAGTTCTGGGTGAAGTCAAATCATGTACGTGGCCTGAGACGCATACCTTGTGGGGCGAT
GGAATCCTTGAGAGTGACTTGATAATACCAGTCACACTGGCGGGACCACGAAGCAATCACA
ATCGGAGACCTGGGTACAAGACACAAAACCAGGGCCCATGGGACGAAGGCCGGGTAGAGA
TTGACTTCGATTACTGCCCAGGAACTACGGTCACCCTGAGTGAGAGCTGCGGACACCGTG
GACCTGCCACTCGCACCACCACAGAGAGCGGAAAGTTGATAACAGATTGGTGCTGCAGGA
GCTGCACCTTACCACCACTGCGCTACCAAACTGACAGCGGCTGTTGGTATGGTATGGAGAT
CAGACCACAGAGACATGATGAAAAGACCCTCGTGCAGTCACAAGTGAATGCTTATAATGCTG
ATATGATTGACCCTTTTCAGTTGGGCCTTCTGGTCGTGTTCTTGGCCACCCAGGAGGTCCTT
CGCAAGAGGTGGACAGCCAAGATCAGCATGCCAGCTATACTGATTGCTCTGCTAGTCCTGG
TGTTTGGGGGCATTACTTACACTGATGTGTTACGCTATGTCATCTTGGTGGGGGCAGCTTTC
GCAGAATCTAATTCGGGAGGAGACGTGGTACACTTGGCGCTCATGGCGACCTTCAAGATAC
AACCAGTGTTTATGGTGGCATCGTTTCTCAAAGCGAGATGGACCAACCAGGAGAACATTTTG
TTGATGTTGGCGGCTGTTTTCTTTCAAATGGCTTATCACGATGCCCGCCAAATTCTGCTCTG
GGAGATCCCTGATGTGTTGAATTCACTGGCGGTAGCTTGGATGATACTGAGAGCCATAACAT
TCACAACGACATCAAACGTGGTTGTTCCGCTGCTAGCCCTGCTAACACCCGGGCTGAGATG
CTTGAATCTGGATGTGTACAGGATACTGCTGTTGATGGTCGGAATAGGCAGCTTGATCAGGG
AGAAGAGGAGTGCAGCCGCAAAAAAGAAAGGAGCAAGTCTGCTATGCTTGGCTCTAGCCT
CAACAGGACTTTTCAACCCCATGATCCTTGCTGCTGGACTGATTGCATGTGATCCCAACCGT
AAACGCGGATGGCCCGCAACTGAAGTGATGACAGCTGTCGGCCTAATGTTTGCCATCGTCG
GAGGGCTGGCAGAGCTTGACATTGACTCCATGGCCATTCCAATGACTATCGCGGGGCTCAT
GTTTGCTGCTTTCGTGATTTCTGGGAAATCAACAGATATGTGGATTGAGAGAACGGCGGACA
TTTCCTGGGAAAGTGATGCAGAAATTACAGGCTCGAGCGAAAGAGTTGATGTGCGGCTTGA
TGATGATGGAAACTTCCAGCTCATGAATGATCCAGGAGCACCTTGGAAGATATGGATGCTCA
GAATGGTCTGTCTCGCGATTAGTGCGTACACCCCCTGGGCAATCTTGCCCTCAGTAGTTGG
ATTTTGGATAACTCTCCAATACACAAAGAGAGGAGGCGTGTTGTGGGACACTCCCTCACCA
AAGGAGTACAAAAAGGGGGACACGACCACCGGCGTCTACAGGATCATGACTCGTGGGCTG
CTCGGCAGTTATCAAGCAGGAGCGGGCGTGATGGTTGAAGGTGTTTTCCACACCCTTTGG
CATACAACAAAAGGAGCCGCTTTGATGAGCGGAGAGGGCCGCCTGGACCCATACTGGGGC
AGTGTCAAGGAGGATCGACTTTGTTACGGAGGACCCTGGAAATTGCAGCACAAGTGGAAC
GGGCAGGATGAGGTGCAGATGATTGTGGTGGAACCTGGCAAGAACGTTAAGAACGTCCAG
ACGAAACCAGGGGTGTTCAAAACACCTGAAGGAGAAATCGGGGCCGTGACTTTGGACTTC
CCCACTGGAACATCAGGCTCACCAATAGTGGACAAAAACGGTGATGTGATTGGGCTTTATG
GCAATGGAGTCATAATGCCCAACGGCTCATACATAAGCGCGATAGTGCAGGGTGAAAGGAT
GGATGAGCCAATCCCAGCCGGATTCGAACCTGAGATGCTGAGGAAAAAACAGATCACTGTA
CTGGATCTCCATCCCGGCGCCGGTAAAACAAGGAGGATTCTGCCACAGATCATCAAAGAGG
CCATAAACAGAAGACTGAGAACAGCCGTGCTAGCGCCAACCAGGGTTGTGGCTGCTGAGA
TGGCTGAAGCACTGAGAGGACTGCCCATCCGGTACCAGACATCCGCAGTGCCCAGAGAAC
ATAATGGAAATGAGATTGTTGATGTCATGTGTCATGCTACCCTCACCCACAGGCTGATGTCTC
CTCACAGGGTGCCGAACTACAACCTGTTCGTGATGGATGAGGCTCATTTCACCGACCCAGC
TAGCATTGCAGCAAGAGGTTACATTTCCACAAAGGTCGAGCTAGGGGAGGCGGCGGCAATA

File S1 WNV reference sequences

TTCATGACAGCCACCCCACCAGGCACTTCAGATCCATTCCCAGAGTCCAATTCACCAATTTC
CGACTTACAGACTGAGATCCCGGATCGAGCTTGGAACTCTGGATACGAATGGATCACAGAA
TACACCGGGAAGACGGTTTGGTTTGTGCCTAGTGTCAAGATGGGGAATGAGATTGCCCTTT
GCCTACAACGTGCTGGAAAGAAAGTAGTCCAATTGAACAGAAAGTCGTACGAGACGGAGTA
CCCAAAATGTAAGAACGATGATTGGGACTTTGTTATCACAACAGACATATCTGAAATGGGGG
CTAACTTCAAGGCGAGCAGGGTGATTGACAGCCGGAAGAGTGTGAAACCAACCATCATAAC
AGAAGGAGAAGGGAGAGTGATCCTGGGAGAACCATCTGCAGTGACAGCAGCTAGTGCCGC
CCAGAGACGTGGACGTATCGGTAGAAATCCGTCGCAAGTTGGTGATGAGTACTGTTATGGG
GGGCACACGAATGAAGACGACTCGAACTTCGCCCATTGGACTGAGGCACGAATCATGCTG
GACAACATCAACATGCCAAACGGACTGATCGCTCAATTCTACCAACCAGAGCGTGAGAAGG
TATATACCATGGATGGGGAATACCGGCTCAGAGGAGAAGAGAGAAAAAACTTTCTGGAACTG
TTGAGGACTGCAGATCTGCCAGTTTGGCTGGCTTACAAGGTTGCAGCGGCTGGAGTGTCAT
ACCACGACCGGAGGTGGTGCTTTGATGGTCCTAGGACAAACACAATTTTAGAAGACAACAA
CGAAGTGGAAGTCATCACGAAGCTTGGTGAAAGGAAGATTCTGAGGCCGCGCTGGATTGAT
GCCAGGGTGTACTCGGATCACCAGGCACTAAAGGCGTTCAAGGACTTCGCCTCGGGAAAA
CGTTCTCAGATAGGGCTCATTGAGGTTCTGGGAAAGATGCCTGAGCACTTCATGGGGAAGA
CATGGGAAGCACTTGACACCATGTACGTTGTGGCCACTGCAGAGAAAGGAGGAAGAGCTC
ACAGAATGGCCCTGGAGGAACTGCCAGATGCTCTTCAGACAATTGCCTTGATTGCCTTATTG
AGTGTGATGACCATGGGAGTATTCTTCCTCCTCATGCAGCGGAAGGGCATTGGAAAGATAG
GTTTGGGAGGCGCTGTCTTGGGAGTCGCGACCTTTTTCTGTTGGATGGCTGAAGTTCCAG
GAACGAAGATCGCCGGAATGTTGCTGCTCTCCCTTCTCTTGATGATTGTGCTAATTCCTGAG
CCAGAGAAGCAACGTTCGCAGACAGACAACCAGCTAGCCGTGTTCCTGATTTGTGTCATGA
CCCTTGTGAGCGCAGTGGCAGCCAACGAGATGGGTTGGCTAGATAAGACCAAGAGTGACA
TAAGCAGTTTGTTTGGGCAAAGAATTGAGGTCAAGGAGAATTTCAGCATGGGAGAGTTTCTT
CTGGACTTGAGGCCGGCAACAGCCTGGTCACTGTACGCTGTGACAACAGCGGTCCTCACT
CCACTGCTAAAGCATTTGATCACGTCAGATTACATCAACACCTCATTGACCTCAATAAACGTT
CAGGCAAGTGCACTATTCACACTCGCGCGAGGCTTCCCCTTCGTCGATGTTGGAGTGTCG
GCTCTCCTGCTAGCAGCCGGATGCTGGGGACAAGTCACCCTCACCGTTACGGTAACAGCG
GCAACACTCCTTTTTTGCCACTATGCCTACATGGTTCCCGGTTGGCAAGCTGAGGCAATGC
GCTCAGCCCAGCGGCGGACAGCGGCCGGAATCATGAAGAACGCTGTAGTGGATGGCATCG
TGGCCACGGACGTCCCAGAATTAGAGCGCACCACACCCATCATGCAGAAGAAAGTTGGAC
AGATCATGCTGATCTTGGTGTCTCTAGCTGCAGTAGTAGTGAACCCGTCTGTGAAGACAGTA
CGAGAAGCCGGAATTTTGATCACGGCCGCAGCGGTGACGCTTTGGGAGAATGGAGCAAGC
TCTGTTTGGAACGCAACAACTGCCATCGGACTCTGCCACATCATGCGTGGGGGTTGGTTGT
CATGTCTATCCATAACATGGACACTCATAAAGAACATGGAAAAACCAGGACTAAAAAGAGGT
GGGGCAAAAGGACGCACCTTGGGAGAGGTTTGGAAAGAAAGACTCAACCAGATGACAAAA
GAAGAGTTCACTAGGTACCGCAAAGAGGCCATCATCGAAGTCGATCGCTCAGCGGCAAAAC
ACGCCAGGAAAGAAGGCAATGTCACTGGAGGGCATCCAGTCTCTAGGGGCACAGCAAAAC
TGAGATGGCTGGTCGAACGGAGGTTTCTCGAACCGGTCGGAAAAGTGATTGACCTTGGAT
GTGGAAGAGGCGGTTGGTGTTACTATATGGCAACCCAAAAAAGAGTCCAAGAAGTCAGAGG
GTACACAAAGGGCGGTCCCGGACATGAAGAGCCCCAACTAGTGCAAAGTTATGGATGGAAC
ATTGTCACCATGAAGAGTGGAGTGGATGTGTTCTACAGACCTTCTGAGTGTTGTGACACCCT
CCTTTGTGACATCGGAGAGTCCTCGTCAAGTGCTGAGGTTGAAGAGCATAGGACGATTCGG
GTCCTTGAAATGGTTGAGGACTGGCTGCACCGAGGGCCAAGGGAATTTTGCGTGAAGGTG
CTCTGTCCCTACATGCCGAAAGTCATAGAGAAGATGGAGCTGCTCCAACGCCGGTATGGGG
GGGGACTGGTCAGAAACCCACTCTCACGGAATTCCACGCACGAGATGTATTGGGTGAGTC
GAGCTTCAGGCAATGTGGTACATTCAGTGAATATGACCAGCCAGGTGCTCCTAGGAAGAAT

File S1 WNV reference sequences

GGAAAAAAGGACCTGGAAGGGACCCCAATACGAGGAAGATGTAAACTTGGGAAGTGGAAC
CAGGGCGGTGGGAAAACCCCTGCTCAACTCAGACACCAGTAAAATCAAGAACAGGATTGAA
CGACTCAGGCGTGAGTACAGTTCGACGTGGCACCACGATGAGAACCACCCATATAGAACCT
GGAACTATCACGGCAGTTATGATGTGAAGCCCACAGGCTCCGCCAGTTCGCTGGTCAATGG
AGTGGTCAGGCTCCTCTCAAAACCATGGGACACCATCACGAATGTTACCACCATGGCCATG
ACTGACACTACTCCCTTCGGGCAGCAGCGAGTGTTCAAAGAGAAGGTGGACACGAAAGCT
CCTGAACCGCCAGAAGGAGTGAAGTACGTGCTCAACGAGACCACCAACTGGTTGTGGGCG
TTTTTGGCCAGAGAAAAACGTCCCAGAATGTGCTCTCGAGAGGAATTCATAAGAAAGGTCAA
CAGCAATGCAGCTTTGGGTGCCATGTTTGAAGAGCAGAATCAATGGAGGAGCGCCAGAGA
AGCAGTTGAAGATCCAAAATTTTGGGAGATGGTGGATGAGGAGCGCGAGGCACATCTGCG
GGGGGAATGTCACACTTGCATTTACAACATGATGGGAAAGAGAGAGAAAAAACCCGGAGAG
TTCGGAAAGGCCAAGGGAAGCAGAGCCATTTGGTTCATGTGGCTCGGAGCTCGCTTTCTG
GAGTTCGAGGCTCTGGGTTTTCTCAATGAAGACCACTGGCTTGGAAGAAAGAACTCAGGAG
GAGGTGTCGAGGGCTTGGGCCTCCAAAAACTGGGTTACATCCTGCGTGAAGTTGGCACCC
GGCCTGGGGGCAAGATCTATGCTGATGACACAGCTGGCTGGGACACCCGCATCACGAGAG
CTGACTTGGAAAATGAAGCTAAGGTGCTTGAGCTGCTTGATGGGGAACATCGGCGTCTTGC
CAGGGCCATCATTGAGCTCACCTATCGTCACAAAGTTGTGAAAGTGATGCGCCCGGCTGCT
GATGGAAGAACCGTCATGGATGTTATCTCCAGAGAAGATCAGAGGGGGAGTGGACAAGTTG
TCACCTACGCCCTAAACACTTTCACCAACCTGGCCGTCCAGCTGGTGAGGATGATGGAAGG
GGAAGGAGTGATTGGCCCAGATGATGTGGAGAAACTCACAAAAGGGAAAGGACCCAAAGT
CAGGACCTGGCTGTTTGAGAATGGGGAAGAAAGACTCAGCCGCATGGCTGTCAGTGGAGA
TGACTGTGTGGTAAAGCCCCTGGACGATCGCTTTGCCACCTCGCTCCACTTCCTCAATGCT
ATGTCAAAGGTTCGCAAAGACATCCAAGAGTGGAAACCGTCAACTGGATGGTATGATTGGC
AGCAGGTTCCATTTTGCTCAAACCATTTCACTGAATTGATCATGAAAGATGGAAGAACACTG
GTGGTTCCATGCCGAGGACAGGATGAATTGGTAGGCAGAGCTCGCATATCTCCAGGGGCC
GGATGGAACGTCCGCGACACTGCTTGTCTGGCTAAGTCTTATGCCCAGATGTGGCTGCTTC
TGTACTTCCACAGAAGAGACCTGCGGCTCATGGCCAACGCCATTTGCTCCGCTGTCCCTGT
GAATTGGGTCCCTACCGGAAGAACCACGTGGTCCATCCATGCAGGAGGAGAGTGGATGAC
AACAGAGGACATGTTGGAGGTCTGGAACCGTGTTTGGATAGAGGAGAATGAATGGATGGAA
GACAAAACCCCAGTGGAGAAATGGAGTGACGTCCCATATTCAGGAAAACGAGAGGACATCT
GGTGTGGCAGCCTGATTGGCACAAGAGCCCGAGCCACGTGGGCAGAAAACATCCAGGTG
GCTATCAACCAAGTCAGAGCAATCATCGGAGATGAGAAGTATGTGGACTACATGAGTTCACT
AAAGAGATATGAAGACACAACTTTGGTTGAGGACACAGTACTGTAGATATTTAATCAATTGTA
AATAGACAATATAAGTATGCATAAAAGTGTAGTTTTATAGTAGTATTTAGTGGTGTTAGTGTAAA
TAGTTAAGAAAATTTTGAGGAGAAAGTCAGGCCGGGAAGTTCCCGCCACCGGAAGTTGAGT
AGACGGTGCTGCCTGCGACTCAACCCCAGGAGGACTGGGTGAACAAAGCCGCGAAGTGA
TCCATGTAAGCCCTCAGAACCGTCTCGGAAGGAGGACCCCACATGTTGTAACTTCAAAGCC
CAATGTCAGACCACGCTACGGCGTGCTACTCTGCGGAGAGTGCAGTCTGCGATAGTGCCC
CAGGAGGACTGGGTTAACAAAGGCAAACCAACGCCCCACGCGGCCCTAGCCCCGGTAATG
GTGTTAACCAGGGCGAAAGGACTAGAGGTTAGAGGAGACCCCGCGGTTTAAAGTGCACGG
CCCAGCCTGGCTGAAGCTGTAGGTCAGGGGAAGGACTAGAGGTTAGTGGAGACCCCGTG
CCACAAAACACCACAACAAAACAGCATATTGACACCTGGGATAGACTAGGAGATCTTCTGCT
CTGCACAACCAGCCACACGGCACAGTGCGCCGACAATGGTGGCTGGTGGTGCGAGAACA
CAGGATCT

File S1 WNV reference sequences

> E/NS1-Per
E sequences are in green.
NS1 sequences are in red.

AGTAGTTCGCCTGTGTGAGCTGACAAACTTAGTAGTGTTTGTGAGGATTAACAACAATTAAC
ACAGTGCGAGCTGTTTCTTAGCACGAAGATCTCGATGTCTAAGAAACCAGGAGGGCCCGGC
AAGAGCCGGGCTGTCAATATGCTAAAACGCGGAATGCCCCGCGTGTTGTCCTTGATTGGAC
TGAAGAGGGCTATGTTGAGCCTGATCGACGGCAAGGGGCCAATACGATTTGTGTTGGCTCT
CTTGGCGTTCTTCAGGTTCACAGCAATTGCTCCGACCCGAGCAGTGCTGGATCGATGGAGA
GGTGTGAACAAACAAACAGCGATGAAACACCTTCTGAGTTTTAAGAAGGAACTAGGGACCT
TGACCAGTGCTATCAATCGGCGGAGCTCAAAACAAAAGAAAAGAGGAGGAAAGACCGGAAT
TGCAGTCATGATTGGCCTGATCGCCAGCGTAGGAGCAGTTACCCTCTCTAACTTCCAAGGG
AAGGTGATGATGACGGTAAATGCTACTGACGTCACAGATGTCATCACGATTCCAACAGCTGC
TGGAAAGAACCTATGCATTGTCAGAGCAATGGATGTGGGATACATGTGCGATGATACTATCA
CTTATGAATGCCCAGTACTGTCGGCTGGTAATGATCCAGAAGACATCGACTGTTGGTGCACA
AAGTCAGCAGTCTACGTCAGGTATGGAAGATGCACCAAGACACGCCACTCAAGACGCAGTC
GGAGGTCACTGACAGTGCAGACACACGGAGAAAGCACTCTAGCGAACAAGAAGGGGGCTT
GGATGGACAGCACCAAGGCCACAAGGTATTTGGTAAAAACAGAATCATGGATCTTGAGGAA
CCCTGGATATGCCCTGGTGGCAGCCGTCATTGGTTGGATGCTTGGGAGCAACACCATGCA
GAGAGTTGTGTTTGTCGTGCTATTGCTTTTGGTGGCCCCAGCTTACAGC**TTCAACTGTCTG**
GGCATGAGCAATAGAGACTTTTTGGAAGGAGTCTCCGGAGCAACCTGGGTGGATCTGGT
CCTCGAAGGCGACAGCTGCGTGACTATCATGTCCAAGGATAAGCCCACCATCGATGTGAA
GATGATGAACATGGAGGCTGCCAATCTGGCGGAGGTTCGAAGCTATTGTTATTTGGCAAC
CGTCAGTGATCTCTCCACTAAAGCTGCCTGCCCGACCATGGGAGAAGCGCACAACGACA
AACGTGCTGACCCTGCATTTGTGTGCAGACAAGGAGTGGTGGACAGGGGCTGGGGCAA
CGGATGCGGCCTTTTTGGGAAAGGCAGCATTGACACATGCGCCAAATTTGCTTGTTCCAC
TAAAGCAATAGGGAGAACCATTTTGAAAGAGAACATCAAGTACGAAGTGGCTATCTTTGT
CCATGGACCAACTACAGTGGAGTCTCACGGCAACTACTCCACCCAGGTTGGAGCCACAC
AGGCAGGAAGACTCAGCATCACACCAGCAGCTCCATCATACACACTAAAACTTGGAGAAT
ATGGCGAGGTCACAGTTGACTGTGAACCTCGGTCAGGAATTGACACCAATGCCTACTACG
TGATGACTGTGGGAACTAAGACGTTCTTGGTTCACCGTGAGTGGTTCATGGACCTGAACC
TGCCTTGGAGCAGCGCGGGAAGCACAGTGTGGAGGAACCGAGAGACTTTGATGGAGTT
CGAGGAGCCGCACGCCACTAAGCAGTCTGTGATAGCCTTGGGCTCTCAGGAGGGAGCG
CTGCACCAAGCATTGGCTGGAGCCATCCCAGTTGAATTCTCCAGTAACACAGTCAAGTTG
ACGTCGGGTCATTTAAAATGTAGAGTGAAGATGGAAAAATTGCAGTTGAAAGGAACAACT
TATGGAGTATGCTCAAAAGCGTTCAAGTTCCTTGGGACACCCGCTGACACTGGACACGGC
ACGGTGGTCTTGGAATTGCAGTACACTGGCACGGATGGACCTTGCAAGGTTCCGATCTCG
TCTGTGGCCTCGTTAAACGATTTGACGCCTGTGGGCAGATTGGTGACTGTCAATCCTTTTG
TGTCAGTGGCCACTGCCAATGCTAAGGTCCTCATTGAGTTGGAACCTCCATTTGGAGACT
CATACATAGTGGTGGGCAGAGGAGAACAACAGATCAACCATCATTGGCACAAGTCTGGC
AGCAGCATTGGCAAAGCTTTCACAACCACTCTCAAAGGAGCACAGAGACTAGCCGCTCT
AGGAGACACAGCCTGGGACTTTGGCTCAGTTGGAGGGGTCTTTACATCAGTTGGGAAAG
CTGTCCACCAGGTGTTTGGAGGAGCTTTCAGATCACTGTTCGGAGGAATGTCTTGGATAA
CTCAGGGCTTGCTGGGGGCTCTTCTGTTGTGGATGGGAATCAATGCGCGTGATAGGTCAA
TAGCACTCACGTTCCTCGCAGTTGGAGGAGTTCTTCTGTTCCTGTCTGTGAACGTGCACG
CTGACACGGGGTGTGCCATTGATATCAGCCGCCAAGAGCTGAGATGTGGAAGTGGGGTG
TTCATACACAACGATGTGGAGGCTTGGATGGATCGGTACAAGTATTACCCAGAAACGCCA

File S1 WNV reference sequences

**CAAGGACTAGCCAAGATCATACAGAAGGCACATAAAGAAGGCGTGTGCGGTCTGCGATC
TGTTTCCAGGCTGGAACACCAAATGTGGGAGGCTGTGAAGGATGAACTCAATACACTTTT
GAAGGAAAACGGTGTGGATCTCAGTGTCGTGGTTGAGAAGCAAGAGGGAATGTACAAGT
CCGCTCCCAAGCGCCTGACGGCCACGACAGAAAAATTGGAAATTGGCTGGAAGGCCTG
GGGTAAGAGCATTTTATTTGCACCGGAACTAGCCAACAACACCTTTGTTGTGGATGGTCCT
GAGACCAAAGAATGTCCGACTCAGAATCGTGCTTGGAACAGTTTGGAAGTAGAGGATTTT
GGATTCGGACTCACCAGTACTCGGATGTTCCTCAAGGTCAGAGAGAGCAACACAACAGA
ATGTGACTCTAAGATCATTGGAACTGCTGTGAAGAACAACTTGGCCATCCACAGTGACCT
GTCATATTGGATTGAGAGCAGGCTAAATGACACCTGGAAGCTGGAGAGGGCAGTACTTGG
TGAGGTTAAATCCTGTACGTGGCCTGAGACCCATACATTGTGGGGCGATGGAATCCTCGA
GAGCGACTTGATAATTCCAGTCACACTGGCTGGACCTCGAAGCAACCACAATCGGAGAC
CGGGGTACAAGACACAAAACCAGGGTCCCTGGGATGAAGGCCGAGTAGAAATTGACTTT
GATTATTGCCCAGGAACCACAGTCACGCTGAGCGAAAGCTGTGGACACCGAGGCCCGGC
CACTCGCACTACGACAGAGAGTGGGAAGTTGATCACAGACTGGTGCTGCAGAAGCTGCA
CGTTACCACCACTTCGCTACCAAACTGACAGCGGCTGCTGGTATGGAATGGAGATCAGAC
CACAGAGACATGATGAGAAGACCCTCGTGCAGTCACAAGTGAATGCT**TATAATGCTGATAT
GATTGACCCTTTTCAGTTGGGCCTTCTGGTCGTGTTCTTGGCCACCCAGGAGGTCCTTCGC
AAGAGGTGGACAGCCAAGATCAGCATGCCAGCTATACTGATTGCTCTGCTAGTCCTGGTGT
TTGGGGGCATTACTTACACTGATGTGTTACGCTATGTCATCTTGGTGGGGGCAGCTTTCGCA
GAATCTAATTCGGGAGGAGACGTGGTACACTTGGCGCTCATGGCGACCTTCAAGATACAAC
CAGTGTTTATGGTGGCATCGTTTCTCAAAGCGAGATGGACCAACCAGGAGAACATTTTGTTG
ATGTTGGCGGCTGTTTTCTTTCAAATGGCTTATCACGATGCCCGCCAAATTCTGCTCTGGGA
GATCCCTGATGTGTTGAATTCACTGGCGGTAGCTTGGATGATACTGAGAGCCATAACATTCA
CAACGACATCAAACGTGGTTGTTCCGCTGCTAGCCCTGCTAACACCCGGGCTGAGATGCTT
GAATCTGGATGTGTACAGGATACTGCTGTTGATGGTCGGAATAGGCAGCTTGATCAGGGAG
AAGAGGAGTGCAGCCGCAAAAAAGAAAGGAGCAAGTCTGCTATGCTTGGCTCTAGCCTCAA
CAGGACTTTTCAACCCCATGATCCTTGCTGCTGGACTGATTGCATGTGATCCCAACCGTAAA
CGCGGATGGCCCGCAACTGAAGTGATGACAGCTGTCGGCCTAATGTTTGCCATCGTCGGA
GGGCTGGCAGAGCTTGACATTGACTCCATGGCCATTCCAATGACTATCGCGGGGCTCATGT
TTGCTGCTTTCGTGATTTCTGGGAAATCAACAGATATGTGGATTGAGAGAACGGCGGACATT
TCCTGGGAAAGTGATGCAGAAATTACAGGCTCGAGCGAAAGAGTTGATGTGCGGCTTGATG
ATGATGGAAACTTCCAGCTCATGAATGATCCAGGAGCACCTTGGAAGATATGGATGCTCAGA
ATGGTCTGTCTCGCGATTAGTGCGTACACCCCCTGGGCAATCTTGCCCTCAGTAGTTGGATT
TTGGATAACTCTCCAATACACAAAGAGAGGAGGCGTGTTGTGGGACACTCCCTCACCAAAG
GAGTACAAAAAGGGGGACACGACCACCGGCGTCTACAGGATCATGACTCGTGGGCTGCTC
GGCAGTTATCAAGCAGGAGCGGGCGTGATGGTTGAAGGTGTTTTCCACACCCTTTGGCATA
CAACAAAAGGAGCCGCTTTGATGAGCGGAGAGGGCCGCCTGGACCCATACTGGGGCAGT
GTCAAGGAGGATCGACTTTGTTACGGAGGACCCTGGAAATTGCAGCACAAGTGGAACGGG
CAGGATGAGGTGCAGATGATTGTGGTGGAACCTGGCAAGAACGTTAAGAACGTCCAGACG
AAACCAGGGGTGTTCAAAACACCTGAAGGAGAAATCGGGGCCGTGACTTTGGACTTCCCC
ACTGGAACATCAGGCTCACCAATAGTGGACAAAAACGGTGATGTGATTGGGCTTTATGGCAA
TGGAGTCATAATGCCCAACGGCTCATACATAAGCGCGATAGTGCAGGGTGAAAGGATGGAT
GAGCCAATCCCAGCCGGATTCGAACCTGAGATGCTGAGGAAAAAACAGATCACTGTACTGG
ATCTCCATCCCGGCGCCGGTAAAACAAGGAGGATTCTGCCACAGATCATCAAAGAGGCCAT
AAACAGAAGACTGAGAACAGCCGTGCTAGCGCCAACCAGGGTTGTGGCTGCTGAGATGGC
TGAAGCACTGAGAGGACTGCCCATCCGGTACCAGACATCCGCAGTGCCCAGAGAACATAAT
GGAAATGAGATTGTTGATGTCATGTGTCATGCTACCCTCACCCACAGGCTGATGTCTCCTCA

File S1 WNV reference sequences

CAGGGTGCCGAACTACAACCTGTTCGTGATGGATGAGGCTCATTTCACCGACCCAGCTAGC
ATTGCAGCAAGAGGTTACATTTCCACAAAGGTCGAGCTAGGGGAGGCGGCGGCAATATTCA
TGACAGCCACCCCACCAGGCACTTCAGATCCATTCCCAGAGTCCAATTCACCAATTTCCGA
CTTACAGACTGAGATCCCGGATCGAGCTTGGAACTCTGGATACGAATGGATCACAGAATACA
CCGGGAAGACGGTTTGGTTTGTGCCTAGTGTCAAGATGGGGAATGAGATTGCCCTTTGCCT
ACAACGTGCTGGAAAGAAAGTAGTCCAATTGAACAGAAAGTCGTACGAGACGGAGTACCCA
AAATGTAAGAACGATGATTGGGACTTTGTTATCACAACAGACATATCTGAAATGGGGGCTAAC
TTCAAGGCGAGCAGGGTGATTGACAGCCGGAAGAGTGTGAAACCAACCATCATAACAGAAG
GAGAAGGGAGAGTGATCCTGGGAGAACCATCTGCAGTGACAGCAGCTAGTGCCGCCCAGA
GACGTGGACGTATCGGTAGAAATCCGTCGCAAGTTGGTGATGAGTACTGTTATGGGGGGCA
CACGAATGAAGACGACTCGAACTTCGCCCATTGGACTGAGGCACGAATCATGCTGGACAAC
ATCAACATGCCAAACGGACTGATCGCTCAATTCTACCAACCAGAGCGTGAGAAGGTATATAC
CATGGATGGGGAATACCGGCTCAGAGGAGAAGAGAGAAAAAACTTTCTGGAACTGTTGAGG
ACTGCAGATCTGCCAGTTTGGCTGGCTTACAAGGTTGCAGCGGCTGGAGTGTCATACCACG
ACCGGAGGTGGTGCTTTGATGGTCCTAGGACAAACACAATTTTAGAAGACAACAACGAAGT
GGAAGTCATCACGAAGCTTGGTGAAAGGAAGATTCTGAGGCCGCGCTGGATTGATGCCAG
GGTGTACTCGGATCACCAGGCACTAAAGGCGTTCAAGGACTTCGCCTCGGGAAAACGTTCT
CAGATAGGGCTCATTGAGGTTCTGGGAAAGATGCCTGAGCACTTCATGGGGAAGACATGGG
AAGCACTTGACACCATGTACGTTGTGGCCACTGCAGAGAAAGGAGGAAGAGCTCACAGAAT
GGCCCTGGAGGAACTGCCAGATGCTCTTCAGACAATTGCCTTGATTGCCTTATTGAGTGTG
ATGACCATGGGAGTATTCTTCCTCCTCATGCAGCGGAAGGGCATTGGAAAGATAGGTTTGG
GAGGCGCTGTCTTGGGAGTCGCGACCTTTTTCTGTTGGATGGCTGAAGTTCCAGGAACGA
AGATCGCCGGAATGTTGCTGCTCTCCCTTCTCTTGATGATTGTGCTAATTCCTGAGCCAGAG
AAGCAACGTTCGCAGACAGACAACCAGCTAGCCGTGTTCCTGATTTGTGTCATGACCCTTG
TGAGCGCAGTGGCAGCCAACGAGATGGGTTGGCTAGATAAGACCAAGAGTGACATAAGCA
GTTTGTTTGGGCAAAGAATTGAGGTCAAGGAGAATTTCAGCATGGGAGAGTTTCTTCTGGA
CTTGAGGCCGGCAACAGCCTGGTCACTGTACGCTGTGACAACAGCGGTCCTCACTCCACT
GCTAAAGCATTTGATCACGTCAGATTACATCAACACCTCATTGACCTCAATAAACGTTCAGGC
AAGTGCACTATTCACACTCGCGCGAGGCTTCCCCTTCGTCGATGTTGGAGTGTCGGCTCTC
CTGCTAGCAGCCGGATGCTGGGGACAAGTCACCCTCACCGTTACGGTAACAGCGGCAACA
CTCCTTTTTTGCCACTATGCCTACATGGTTCCCGGTTGGCAAGCTGAGGCAATGCGCTCAG
CCCAGCGGCGGACAGCGGCCGGAATCATGAAGAACGCTGTAGTGGATGGCATCGTGGCCA
CGGACGTCCCAGAATTAGAGCGCACCACACCCATCATGCAGAAGAAAGTTGGACAGATCAT
GCTGATCTTGGTGTCTCTAGCTGCAGTAGTAGTGAACCCGTCTGTGAAGACAGTACGAGAA
GCCGGAATTTTGATCACGGCCGCAGCGGTGACGCTTTGGGAGAATGGAGCAAGCTCTGTT
TGGAACGCAACAACTGCCATCGGACTCTGCCACATCATGCGTGGGGGTTGGTTGTCATGTC
TATCCATAACATGGACACTCATAAAGAACATGGAAAAACCAGGACTAAAAAGAGGTGGGGCA
AAAGGACGCACCTTGGGAGAGGTTTGGAAAGAAAGACTCAACCAGATGACAAAAGAAGAG
TTCACTAGGTACCGCAAAGAGGCCATCATCGAAGTCGATCGCTCAGCGGCAAAACACGCCA
GGAAAGAAGGCAATGTCACTGGAGGGCATCCAGTCTCTAGGGGCACAGCAAAACTGAGAT
GGCTGGTCGAACGGAGGTTTCTCGAACCGGTCGGAAAAGTGATTGACCTTGGATGTGGAA
GAGGCGGTTGGTGTTACTATATGGCAACCCAAAAAAGAGTCCAAGAAGTCAGAGGGTACAC
AAAGGGCGGTCCCGGACATGAAGAGCCCCAACTAGTGCAAAGTTATGGATGGAACATTGTC
ACCATGAAGAGTGGAGTGGATGTGTTCTACAGACCTTCTGAGTGTTGTGACACCCTCCTTT
GTGACATCGGAGAGTCCTCGTCAAGTGCTGAGGTTGAAGAGCATAGGACGATTCGGGTCC
TTGAAATGGTTGAGGACTGGCTGCACCGAGGGCCAAGGGAATTTTGCGTGAAGGTGCTCT
GTCCCTACATGCCGAAAGTCATAGAGAAGATGGAGCTGCTCCAACGCCGGTATGGGGGG

File S1 WNV reference sequences

GACTGGTCAGAAACCCACTCTCACGGAATTCCACGCACGAGATGTATTGGGTGAGTCGAGC
TTCAGGCAATGTGGTACATTCAGTGAATATGACCAGCCAGGTGCTCCTAGGAAGAATGGAAA
AAAGGACCTGGAAGGGACCCCAATACGAGGAAGATGTAAACTTGGGAAGTGGAACCAGGG
CGGTGGGAAAACCCCTGCTCAACTCAGACACCAGTAAAATCAAGAACAGGATTGAACGACT
CAGGCGTGAGTACAGTTCGACGTGGCACCACGATGAGAACCACCCATATAGAACCTGGAAC
TATCACGGCAGTTATGATGTGAAGCCCACAGGCTCCGCCAGTTCGCTGGTCAATGGAGTGG
TCAGGCTCCTCTCAAAACCATGGGACACCATCACGAATGTTACCACCATGGCCATGACTGA
CACTACTCCCTTCGGGCAGCAGCGAGTGTTCAAAGAGAAGGTGGACACGAAAGCTCCTGA
ACCGCCAGAAGGAGTGAAGTACGTGCTCAACGAGACCACCAACTGGTTGTGGGCGTTTTT
GGCCAGAGAAAAACGTCCCAGAATGTGCTCTCGAGAGGAATTCATAAGAAAGGTCAACAGC
AATGCAGCTTTGGGTGCCATGTTTGAAGAGCAGAATCAATGGAGGAGCGCCAGAGAAGCA
GTTGAAGATCCAAAATTTTGGGAGATGGTGGATGAGGAGCGCGAGGCACATCTGCGGGGG
GAATGTCACACTTGCATTTACAACATGATGGGAAAGAGAGAGAAAAAACCCGGAGAGTTCG
GAAAGGCCAAGGGAAGCAGAGCCATTTGGTTCATGTGGCTCGGAGCTCGCTTTCTGGAGT
TCGAGGCTCTGGGTTTTCTCAATGAAGACCACTGGCTTGGAAGAAAGAACTCAGGAGGAG
GTGTCGAGGGCTTGGGCCTCCAAAAACTGGGTTACATCCTGCGTGAAGTTGGCACCCGGC
CTGGGGGCAAGATCTATGCTGATGACACAGCTGGCTGGGACACCCGCATCACGAGAGCTG
ACTTGGAAAATGAAGCTAAGGTGCTTGAGCTGCTTGATGGGGAACATCGGCGTCTTGCCAG
GGCCATCATTGAGCTCACCTATCGTCACAAAGTTGTGAAAGTGATGCGCCCGGCTGCTGAT
GGAAGAACCGTCATGGATGTTATCTCCAGAGAAGATCAGAGGGGGAGTGGACAAGTTGTCA
CCTACGCCCTAAACACTTTCACCAACCTGGCCGTCCAGCTGGTGAGGATGATGGAAGGGG
AAGGAGTGATTGGCCCAGATGATGTGGAGAAACTCACAAAAGGGAAAGGACCCAAAGTCA
GGACCTGGCTGTTTGAGAATGGGGAAGAAAGACTCAGCCGCATGGCTGTCAGTGGAGATG
ACTGTGTGGTAAAGCCCCTGGACGATCGCTTTGCCACCTCGCTCCACTTCCTCAATGCTAT
GTCAAAGGTTCGCAAAGACATCCAAGAGTGGAAACCGTCAACTGGATGGTATGATTGGCAG
CAGGTTCCATTTTGCTCAAACCATTTCACTGAATTGATCATGAAAGATGGAAGAACACTGGT
GGTTCCATGCCGAGGACAGGATGAATTGGTAGGCAGAGCTCGCATATCTCCAGGGGCCGG
ATGGAACGTCCGCGACACTGCTTGTCTGGCTAAGTCTTATGCCCAGATGTGGCTGCTTCTG
TACTTCCACAGAAGAGACCTGCGGCTCATGGCCAACGCCATTTGCTCCGCTGTCCCTGTGA
ATTGGGTCCCTACCGGAAGAACCACGTGGTCCATCCATGCAGGAGGAGAGTGGATGACAA
CAGAGGACATGTTGGAGGTCTGGAACCGTGTTTGGATAGAGGAGAATGAATGGATGGAAGA
CAAAACCCCAGTGGAGAAATGGAGTGACGTCCCATATTCAGGAAAACGAGAGGACATCTGG
TGTGGCAGCCTGATTGGCACAAGAGCCCGAGCCACGTGGGCAGAAAACATCCAGGTGGCT
ATCAACCAAGTCAGAGCAATCATCGGAGATGAGAAGTATGTGGACTACATGAGTTCACTAAA
GAGATATGAAGACACAACTTTGGTTGAGGACACAGTACTGTAGATATTTAATCAATTGTAAATA
GACAATATAAGTATGCATAAAAGTGTAGTTTTATAGTAGTATTTAGTGGTGTTAGTGTAAATAGT
TAAGAAAATTTTGAGGAGAAAGTCAGGCCGGGAAGTTCCCGCCACCGGAAGTTGAGTAGA
CGGTGCTGCCTGCGACTCAACCCCAGGAGGACTGGGTGAACAAAGCCGCGAAGTGATCC
ATGTAAGCCCTCAGAACCGTCTCGGAAGGAGGACCCCACATGTTGTAACTTCAAAGCCCAA
TGTCAGACCACGCTACGGCGTGCTACTCTGCGGAGAGTGCAGTCTGCGATAGTGCCCCAG
GAGGACTGGGTTAACAAAGGCAAACCAACGCCCCACGCGGCCCTAGCCCCGGTAATGGTG
TTAACCAGGGCGAAAGGACTAGAGGTTAGAGGAGACCCCGCGGTTTAAAGTGCACGGCCC
AGCCTGGCTGAAGCTGTAGGTCAGGGGAAGGACTAGAGGTTAGTGGAGACCCCGTGCCAC
AAAACACCACAACAAAACAGCATATTGACACCTGGGATAGACTAGGAGATCTTCTGCTCTGC
ACAACCAGCCACACGGCACAGTGCGCCGACAATGGTGGCTGGTGGTGCGAGAACACAGG
ATCT

File S1 WNV reference sequences

> E/NS1/NS5-Per

E sequences are in green.

NS1 sequences are in red.

NS5 sequences are in blue.

AGTAGTTCGCCTGTGTGAGCTGACAAACTTAGTAGTGTTTGTGAGGATTAACAACAATTAAC
ACAGTGCGAGCTGTTTCTTAGCACGAAGATCTCGATGTCTAAGAAACCAGGAGGGCCCGGC
AAGAGCCGGGCTGTCAATATGCTAAAACGCGGAATGCCCCGCGTGTTGTCCTTGATTGGAC
TGAAGAGGGCTATGTTGAGCCTGATCGACGGCAAGGGGCCAATACGATTTGTGTTGGCTCT
CTTGGCGTTCTTCAGGTTCACAGCAATTGCTCCGACCCGAGCAGTGCTGGATCGATGGAGA
GGTGTGAACAAACAAACAGCGATGAAACACCTTCTGAGTTTTAAGAAGGAACTAGGGACCT
TGACCAGTGCTATCAATCGGCGGAGCTCAAAACAAAAGAAAAGAGGAGGAAAGACCGGAAT
TGCAGTCATGATTGGCCTGATCGCCAGCGTAGGAGCAGTTACCCTCTCTAACTTCCAAGGG
AAGGTGATGATGACGGTAAATGCTACTGACGTCACAGATGTCATCACGATTCCAACAGCTGC
TGGAAAGAACCTATGCATTGTCAGAGCAATGGATGTGGGATACATGTGCGATGATACTATCA
CTTATGAATGCCCAGTACTGTCGGCTGGTAATGATCCAGAAGACATCGACTGTTGGTGCACA
AAGTCAGCAGTCTACGTCAGGTATGGAAGATGCACCAAGACACGCCACTCAAGACGCAGTC
GGAGGTCACTGACAGTGCAGACACACGGAGAAAGCACTCTAGCGAACAAGAAGGGGGCTT
GGATGGACAGCACCAAGGCCACAAGGTATTTGGTAAAAACAGAATCATGGATCTTGAGGAA
CCCTGGATATGCCCTGGTGGCAGCCGTCATTGGTTGGATGCTTGGGAGCAACACCATGCA
GAGAGTTGTGTTTGTCGTGCTATTGCTTTTGGTGGCCCCAGCTTACAGC**TTCAACTGTCTG**
GGCATGAGCAATAGAGACTTTTTGGAAGGAGTCTCCGGAGCAACCTGGGTGGATCTGGT
CCTCGAAGGCGACAGCTGCGTGACTATCATGTCCAAGGATAAGCCCACCATCGATGTGAA
GATGATGAACATGGAGGCTGCCAATCTGGCGGAGGTTCGAAGCTATTGTTATTTGGCAAC
CGTCAGTGATCTCTCCACTAAAGCTGCCTGCCCGACCATGGGAGAAGCGCACAACGACA
AACGTGCTGACCCTGCATTTGTGTGCAGACAAGGAGTGGTGGACAGGGGCTGGGGCAA
CGGATGCGGCCTTTTTGGGAAAGGCAGCATTGACACATGCGCCAAATTTGCTTGTTCCAC
TAAAGCAATAGGGAGAACCATTTTGAAAGAGAACATCAAGTACGAAGTGGCTATCTTTGT
CCATGGACCAACTACAGTGGAGTCTCACGGCAACTACTCCACCCAGGTTGGAGCCACAC
AGGCAGGAAGACTCAGCATCACACCAGCAGCTCCATCATACACACTAAAACTTGGAGAAT
ATGGCGAGGTCACAGTTGACTGTGAACCTCGGTCAGGAATTGACACCAATGCCTACTACG
TGATGACTGTGGGAACTAAGACGTTCTTGGTTCACCGTGAGTGGTTCATGGACCTGAACC
TGCCTTGGAGCAGCGCGGGAAGCACAGTGTGGAGGAACCGAGAGACTTTGATGGAGTT
CGAGGAGCCGCACGCCACTAAGCAGTCTGTGATAGCCTTGGGCTCTCAGGAGGGAGCG
CTGCACCAAGCATTGGCTGGAGCCATCCCAGTTGAATTCTCCAGTAACACAGTCAAGTTG
ACGTCGGGTCATTTAAAATGTAGAGTGAAGATGGAAAAATTGCAGTTGAAAGGAACAACT
TATGGAGTATGCTCAAAAGCGTTCAAGTTCCTTGGGACACCCGCTGACACTGGACACGGC
ACGGTGGTCTTGGAATTGCAGTACACTGGCACGGATGGACCTTGCAAGGTTCCGATCTCG
TCTGTGGCCTCGTTAAACGATTTGACGCCTGTGGGCAGATTGGTGACTGTCAATCCTTTTG
TGTCAGTGGCCACTGCCAATGCTAAGGTCCTCATTGAGTTGGAACCTCCATTTGGAGACT
CATACATAGTGGTGGGCAGAGGAGAACAACAGATCAACCATCATTGGCACAAGTCTGGC
AGCAGCATTGGCAAAGCTTTCACAACCACTCTCAAAGGAGCACAGAGACTAGCCGCTCT
AGGAGACACAGCCTGGGACTTTGGCTCAGTTGGAGGGGTCTTTACATCAGTTGGGAAAG
CTGTCCACCAGGTGTTTGGAGGAGCTTTCAGATCACTGTTCGGAGGAATGTCTTGGATAA
CTCAGGGCTTGCTGGGGGCTCTTCTGTTGTGGATGGGAATCAATGCGCGTGATAGGTCAA
TAGCACTCACGTTCCTCGCAGTTGGAGGAGTTCTTCTGTTCCTGTCTGTGAACGTGCACG
CTGACACGGGGTGTGCCATTGATATCAGCCGCCAAGAGCTGAGATGTGGAAGTGGGGTG
TTCATACACAACGATGTGGAGGCTTGGATGGATCGGTACAAGTATTACCCAGAAACGCCA

File S1 WNV reference sequences

CAAGGACTAGCCAAGATCATACAGAAGGCACATAAAGAAGGCGTGTGCGGTCTGCGATC
TGTTTCCAGGCTGGAACACCAAATGTGGGAGGCTGTGAAGGATGAACTCAATACACTTTT
GAAGGAAAACGGTGTGGATCTCAGTGTCGTGGTTGAGAAGCAAGAGGGAATGTACAAGT
CCGCTCCCAAGCGCCTGACGGCCACGACAGAAAAATTGGAAATTGGCTGGAAGGCCTG
GGGTAAGAGCATTTTATTTGCACCGGAACTAGCCAACAACACCTTTGTTGTGGATGGTCCT
GAGACCAAAGAATGTCCGACTCAGAATCGTGCTTGGAACAGTTTGGAAGTAGAGGATTTT
GGATTCGGACTCACCAGTACTCGGATGTTCCTCAAGGTCAGAGAGAGCAACACAACAGA
ATGTGACTCTAAGATCATTGGAACTGCTGTGAAGAACAACTTGGCCATCCACAGTGACCT
GTCATATTGGATTGAGAGCAGGCTAAATGACACCTGGAAGCTGGAGAGGGCAGTACTTGG
TGAGGTTAAATCCTGTACGTGGCCTGAGACCCATACATTGTGGGGCGATGGAATCCTCGA
GAGCGACTTGATAATTCCAGTCACACTGGCTGGACCTCGAAGCAACCACAATCGGAGAC
CGGGGTACAAGACACAAAACCAGGGTCCCTGGGATGAAGGCCGAGTAGAAATTGACTTT
GATTATTGCCCAGGAACCACAGTCACGCTGAGCGAAAGCTGTGGACACCGAGGCCCGGC
CACTCGCACTACGACAGAGAGTGGGAAGTTGATCACAGACTGGTGCTGCAGAAGCTGCA
CGTTACCACCACTTCGCTACCAAACTGACAGCGGCTGCTGGTATGGAATGGAGATCAGAC
CACAGAGACATGATGAGAAGACCCTCGTGCAGTCACAAGTGAATGCTTATAATGCTGATAT
GATTGACCCTTTTCAGTTGGGCCTTCTGGTCGTGTTCTTGGCCACCCAGGAGGTCCTTCGC
AAGAGGTGGACAGCCAAGATCAGCATGCCAGCTATACTGATTGCTCTGCTAGTCCTGGTGT
TTGGGGGCATTACTTACACTGATGTGTTACGCTATGTCATCTTGGTGGGGGCAGCTTTCGCA
GAATCTAATTCGGGAGGAGACGTGGTACACTTGGCGCTCATGGCGACCTTCAAGATACAAC
CAGTGTTTATGGTGGCATCGTTTCTCAAAGCGAGATGGACCAACCAGGAGAACATTTTGTTG
ATGTTGGCGGCTGTTTTCTTTCAAATGGCTTATCACGATGCCCGCCAAATTCTGCTCTGGGA
GATCCCTGATGTGTTGAATTCACTGGCGGTAGCTTGGATGATACTGAGAGCCATAACATTCA
CAACGACATCAAACGTGGTTGTTCCGCTGCTAGCCCTGCTAACACCCGGGCTGAGATGCTT
GAATCTGGATGTGTACAGGATACTGCTGTTGATGGTCGGAATAGGCAGCTTGATCAGGGAG
AAGAGGAGTGCAGCCGCAAAAAAGAAAGGAGCAAGTCTGCTATGCTTGGCTCTAGCCTCAA
CAGGACTTTTCAACCCCATGATCCTTGCTGCTGGACTGATTGCATGTGATCCCAACCGTAAA
CGCGGATGGCCCGCAACTGAAGTGATGACAGCTGTCGGCCTAATGTTTGCCATCGTCGGA
GGGCTGGCAGAGCTTGACATTGACTCCATGGCCATTCCAATGACTATCGCGGGGCTCATGT
TTGCTGCTTTCGTGATTTCTGGGAAATCAACAGATATGTGGATTGAGAGAACGGCGGACATT
TCCTGGGAAAGTGATGCAGAAATTACAGGCTCGAGCGAAAGAGTTGATGTGCGGCTTGATG
ATGATGGAAACTTCCAGCTCATGAATGATCCAGGAGCACCTTGGAAGATATGGATGCTCAGA
ATGGTCTGTCTCGCGATTAGTGCGTACACCCCCTGGGCAATCTTGCCCTCAGTAGTTGGATT
TTGGATAACTCTCCAATACACAAAGAGAGGAGGCGTGTTGTGGGACACTCCCTCACCAAAG
GAGTACAAAAAGGGGGACACGACCACCGGCGTCTACAGGATCATGACTCGTGGGCTGCTC
GGCAGTTATCAAGCAGGAGCGGGCGTGATGGTTGAAGGTGTTTTCCACACCCTTTGGCATA
CAACAAAAGGAGCCGCTTTGATGAGCGGAGAGGGCCGCCTGGACCCATACTGGGGCAGT
GTCAAGGAGGATCGACTTTGTTACGGAGGACCCTGGAAATTGCAGCACAAGTGGAACGGG
CAGGATGAGGTGCAGATGATTGTGGTGGAACCTGGCAAGAACGTTAAGAACGTCCAGACG
AAACCAGGGGTGTTCAAAACACCTGAAGGAGAAATCGGGGCCGTGACTTTGGACTTCCCC
ACTGGAACATCAGGCTCACCAATAGTGGACAAAAACGGTGATGTGATTGGGCTTTATGGCAA
TGGAGTCATAATGCCCAACGGCTCATACATAAGCGCGATAGTGCAGGGTGAAAGGATGGAT
GAGCCAATCCCAGCCGGATTCGAACCTGAGATGCTGAGGAAAAAACAGATCACTGTACTGG
ATCTCCATCCCGGCGCCGGTAAAACAAGGAGGATTCTGCCACAGATCATCAAAGAGGCCAT
AAACAGAAGACTGAGAACAGCCGTGCTAGCGCCAACCAGGGTTGTGGCTGCTGAGATGGC
TGAAGCACTGAGAGGACTGCCCATCCGGTACCAGACATCCGCAGTGCCCAGAGAACATAAT
GGAAATGAGATTGTTGATGTCATGTGTCATGCTACCCTCACCCACAGGCTGATGTCTCCTCA

File S1 WNV reference sequences

CAGGGTGCCGAACTACAACCTGTTCGTGATGGATGAGGCTCATTTCACCGACCCAGCTAGC
ATTGCAGCAAGAGGTTACATTTCCACAAAGGTCGAGCTAGGGGAGGCGGCGGCAATATTCA
TGACAGCCACCCCACCAGGCACTTCAGATCCATTCCCAGAGTCCAATTCACCAATTTCCGA
CTTACAGACTGAGATCCCGGATCGAGCTTGGAACTCTGGATACGAATGGATCACAGAATACA
CCGGGAAGACGGTTTGGTTTGTGCCTAGTGTCAAGATGGGGAATGAGATTGCCCTTTGCCT
ACAACGTGCTGGAAAGAAAGTAGTCCAATTGAACAGAAAGTCGTACGAGACGGAGTACCCA
AAATGTAAGAACGATGATTGGGACTTTGTTATCACAACAGACATATCTGAAATGGGGGCTAAC
TTCAAGGCGAGCAGGGTGATTGACAGCCGGAAGAGTGTGAAACCAACCATCATAACAGAAG
GAGAAGGGAGAGTGATCCTGGGAGAACCATCTGCAGTGACAGCAGCTAGTGCCGCCCAGA
GACGTGGACGTATCGGTAGAAATCCGTCGCAAGTTGGTGATGAGTACTGTTATGGGGGGCA
CACGAATGAAGACGACTCGAACTTCGCCCATTGGACTGAGGCACGAATCATGCTGGACAAC
ATCAACATGCCAAACGGACTGATCGCTCAATTCTACCAACCAGAGCGTGAGAAGGTATATAC
CATGGATGGGGAATACCGGCTCAGAGGAGAAGAGAGAAAAAACTTTCTGGAACTGTTGAGG
ACTGCAGATCTGCCAGTTTGGCTGGCTTACAAGGTTGCAGCGGCTGGAGTGTCATACCACG
ACCGGAGGTGGTGCTTTGATGGTCCTAGGACAAACACAATTTTAGAAGACAACAACGAAGT
GGAAGTCATCACGAAGCTTGGTGAAAGGAAGATTCTGAGGCCGCGCTGGATTGATGCCAG
GGTGTACTCGGATCACCAGGCACTAAAGGCGTTCAAGGACTTCGCCTCGGGAAAACGTTCT
CAGATAGGGCTCATTGAGGTTCTGGGAAAGATGCCTGAGCACTTCATGGGGAAGACATGGG
AAGCACTTGACACCATGTACGTTGTGGCCACTGCAGAGAAAGGAGGAAGAGCTCACAGAAT
GGCCCTGGAGGAACTGCCAGATGCTCTTCAGACAATTGCCTTGATTGCCTTATTGAGTGTG
ATGACCATGGGAGTATTCTTCCTCCTCATGCAGCGGAAGGGCATTGGAAAGATAGGTTTGG
GAGGCGCTGTCTTGGGAGTCGCGACCTTTTTCTGTTGGATGGCTGAAGTTCCAGGAACGA
AGATCGCCGGAATGTTGCTGCTCTCCCTTCTCTTGATGATTGTGCTAATTCCTGAGCCAGAG
AAGCAACGTTCGCAGACAGACAACCAGCTAGCCGTGTTCCTGATTTGTGTCATGACCCTTG
TGAGCGCAGTGGCAGCCAACGAGATGGGTTGGCTAGATAAGACCAAGAGTGACATAAGCA
GTTTGTTTGGGCAAAGAATTGAGGTCAAGGAGAATTTCAGCATGGGAGAGTTTCTTCTGGA
CTTGAGGCCGGCAACAGCCTGGTCACTGTACGCTGTGACAACAGCGGTCCTCACTCCACT
GCTAAAGCATTTGATCACGTCAGATTACATCAACACCTCATTGACCTCAATAAACGTTCAGGC
AAGTGCACTATTCACACTCGCGCGAGGCTTCCCCTTCGTCGATGTTGGAGTGTCGGCTCTC
CTGCTAGCAGCCGGATGCTGGGGACAAGTCACCCTCACCGTTACGGTAACAGCGGCAACA
CTCCTTTTTTGCCACTATGCCTACATGGTTCCCGGTTGGCAAGCTGAGGCAATGCGCTCAG
CCCAGCGGCGGACAGCGGCCGGAATCATGAAGAACGCTGTAGTGGATGGCATCGTGGCCA
CGGACGTCCCAGAATTAGAGCGCACCACACCCATCATGCAGAAGAAAGTTGGACAGATCAT
GCTGATCTTGGTGTCTCTAGCTGCAGTAGTAGTGAACCCGTCTGTGAAGACAGTACGAGAA
GCCGGAATTTTGATCACGGCCGCAGCGGTGACGCTTTGGGAGAATGGAGCAAGCTCTGTT
TGGAACGCAACAACTGCCATCGGACTCTGCCACATCATGCGTGGGGGTTGGTTGTCATGTC
TATCCATAACATGGACACTCATAAAGAACATGGAAAAACCAGGACTAAAAAGA**GGTGGGGCA**
AAAGGACGCACCTTGGGAGAGGTTTGGAAAGAAAGACTCAACCAGATGACAAAAGAAG
AGTTCACTAGGTACCGAAAGGAGGCAATCATCGAAGTCGATCGATCTGCAGCCAAACATG
CCAGGAAAGAGGGAAACGTCACTGGAGGGCATCCAGTCTCCAGGGGCACTGCAAAGCT
CAGATGGCTTGTCGAACGGAGGTTTCTCGAACCGGTGGGAAAAGTGATTGACCTAGGGT
GTGGAAGAGGTGGCTGGTGTTATTACATGGCGACCCAAAAAAGAGTCCAAGAAGTCAGA
GGCTACACTAAGGGCGGGCCCGGGCATGAAGAGCCCCAACTCGTCCAAAGTTATGGTTG
GAACATTGTCACAATGAAGAGTGGAGTTGATGTTTTTTATAGACCATCAGAATGCTGTGAC
ACCCTACTCTGTGACATAGGAGAGTCTTCGTCCAGTGCTGAGGTGGAAGAGCACAGAAC
CATCCGGGTCCTGGAAATGGTCGAGGATTGGCTCCACCGGGGACCCAGGGAGTTCTGCG
TGAAGGTGCTGTGTCCCTACATGCCGAAAGTAATTGAGAAGATGGAACTGCTGCAACGGC

File S1 WNV reference sequences

**GATATGGTGGTGGACTGGTCAGAAATCCCCTCTCCCGAAATTCGACTCACGAGATGTACT
GGGTGAGTCGGGCCTCGGGCAATGTGGTGCATTCAGTGAACATGACCAGCCAGGTCCTG
CTAGGAAGAATGGAAAAGAGAACTTGGAAGGGACCACAATACGAGGAAGATGTAAACTT
GGGAAGTGGCACCAGGGCAGTGGGCAAGCCCCTCCTCAACTCAGATACTAGTAAAATCA
AGAACAGGATTGAACGTCTCAGGCGGGAGTACAGTTCCACTTGGCATCACGATGAGAAC
CACCCATATAGAACGTGGAACTACCACGGCAGCTATGATGTCAAGCCCACAGGCTCAGCC
AGCTCGCTGGTCAATGGGGTGGTGAGGCTCCTGTCAAAACCCTGGGACACGATCACGAA
TGTCACAACCATGGCCATGACAGACACTACGCCATTTGGACAGCAACGGGTCTTCAAAGA
AAAGGTGGACACCAAAGCACCTGAGCCTCCAGAAGGAGTCAAATACGTGCTGAACGAAA
CCACAAACTGGTTGTGGGCATTTTTGGCCAGAGAAAAACGTCCCAGAATGTGCAGCCGA
GAGGAATTCATCAGGAAAGTAAACTCCAATGCTGCCTTGGGTGCCATGTTTGAAGAACAG
AATCAATGGAGGAGCGCCAGAGAAGCAGTCGAGGATCCAAAGTTCTGGGAGATGGTGGA
CGAGGAGCGCGAAGCCCACCTCCGGGGAGAATGTCACACGTGCATCTACAACATGATGG
GCAAAAGAGAGAAAAAACCAGGAGAGTTCGGGAAAGCAAAGGGCAGCAGAGCCATTTG
GTTCATGTGGCTTGGAGCTCGCTTTCTGGAGTTCGAGGCACTGGGCTTTCTTAATGAAGAT
CACTGGCTTGGGAGGAAGAACTCTGGAGGAGGGGTGGAGGGATTGGGCCTGCAAAAAC
TGGGGTACATCCTGCGTGAGGTTGGCACTCGGCCTGGGGGAAAGATCTATGCTGATGAC
ACTGCCGGTTGGGACACCCGCATCACCAGAGCAGACTTGGAAAACGAAGCCAAAGTGCT
CGAGCTGCTGGATGGGGAACATCGACGTCTTGCCAGAGCTATCATTGAGCTCACCTACCG
TCATAAAGTCGTGAAAGTGATGCGCCCTGCGGCCGACGGCAGAACAGTCATGGATGTTAT
TTCGAGAGAAGACCAGAGAGGAAGTGGCCAGGTAGTCACTTACGCCCTCAACACCTTCA
CAAATCTGGCCGTCCAGCTGGTGAGGATGATGGAAGGCGAAGGAGTCATTGGCCCAGAT
GATGTTGAGAAACTCACCAAAGGAAAAGGACCAAAAGTGAGGACGTGGCTGTTTGAGAA
TGGGGAAGAAAGACTGAGCCGAATGGCAGTCAGTGGAGATGACTGTGTGGTAAAACCGC
TTGACGACCGTTTTGCTACGTCTCTCCATTTCCTCAATGCCATGTCAAAGGTCCGCAAGGA
CATCCAAGAGTGGAAACCTTCCACCGGTTGGTATGACTGGCAGCAAGTTCCCTTTTGTTC
AAACCATTTCACAGAATTGATCATGAAAGATGGAAGAACCCTAGTTGTCCCTTGCCGAGG
GCAGGATGAATTGGTGGGAAGAGCGCGCATATCTCCTGGAGCCGGATGGAATGTTCGCG
ACACTGCCTGTCTGGCCAAGTCCTATGCACAGATGTGGCTCCTGCTTTATTTTCACAGAAG
AGATCTGCGACTCATGGCCAATGCAATTTGTTCCGCGGTCCCTGTCAACTGGGTGCCTAC
CGGGAGAACCACGTGGTCCATTCATGCTGGAGGAGAGTGGATGACAACAGAAGACATGT
TGGAGGTGTGGAACCGAGTCTGGATTGAGGAGAATGAATGGATGGAAGACAAGACTCCT
GTGGAGAAATGGAGTGATGTTCCATACTCTGGAAAGCGAGAGGACATTTGGTGTGGCAG
CCTGATTGGCACCAGAGCCCGGGCCACTTGGGCAGAGAACATCCAGGTGGCTATCAACC
AAGTGAGAGCAATCATCGGAGATGAGAAGTATGTGGACTACATGAGTTCACTAAAGAGAT
ATGAAGACACAACTTTGGTTGAGGACACAGTACTG**TAGATATTTAATCAATTGTAAATAGAC
AATATAAGTATGCATAAAAGTGTAGTTTTATAGTAGTATTTAGTGGTGTTAGTGTAAATAGTTAA
GAAAATTTTGAGGAGAAAGTCAGGCCGGGAAGTTCCCGCCACCGGAAGTTGAGTAGACGG
TGCTGCCTGCGACTCAACCCCAGGAGGACTGGGTGAACAAAGCCGCGAAGTGATCCATGT
AAGCCCTCAGAACCGTCTCGGAAGGAGGACCCCACATGTTGTAACTTCAAAGCCCAATGTC
AGACCACGCTACGGCGTGCTACTCTGCGGAGAGTGCAGTCTGCGATAGTGCCCCAGGAGG
ACTGGGTTAACAAAGGCAAACCAACGCCCCACGCGGCCCTAGCCCCGGTAATGGTGTTAA
CCAGGGCGAAAGGACTAGAGGTTAGAGGAGACCCCGCGGTTTAAAGTGCACGGCCCAGC
CTGGCTGAAGCTGTAGGTCAGGGGAAGGACTAGAGGTTAGTGGAGACCCCGTGCCACAAA
ACACCACAACAAAACAGCATATTGACACCTGGGATAGACTAGGAGATCTTCTGCTCTGCACA
ACCAGCCACACGGCACAGTGCGCCGACAATGGTGGCTGGTGGTGCGAGAACACAGGATC
T

File S1 WNV reference sequences

> E+CG

E sequences are in green.

AGTAGTTCGCCTGTGTGAGCTGACAAACTTAGTAGTGTTTGTGAGGATTAACAACAATTAAC
ACAGTGCGAGCTGTTTCTTAGCACGAAGATCTCGATGTCTAAGAAACCAGGAGGGCCCGGC
AAGAGCCGGGCTGTCAATATGCTAAAACGCGGAATGCCCCGCGTGTTGTCCTTGATTGGAC
TGAAGAGGGCTATGTTGAGCCTGATCGACGGCAAGGGGCCAATACGATTTGTGTTGGCTCT
CTTGGCGTTCTTCAGGTTCACAGCAATTGCTCCGACCCGAGCAGTGCTGGATCGATGGAGA
GGTGTGAACAAACAAACAGCGATGAAACACCTTCTGAGTTTTAAGAAGGAACTAGGGACCT
TGACCAGTGCTATCAATCGGCGGAGCTCAAAACAAAAGAAAAGAGGAGGAAAGACCGGAAT
TGCAGTCATGATTGGCCTGATCGCCAGCGTAGGAGCAGTTACCCTCTCTAACTTCCAAGGG
AAGGTGATGATGACGGTAAATGCTACTGACGTCACAGATGTCATCACGATTCCAACAGCTGC
TGGAAAGAACCTATGCATTGTCAGAGCAATGGATGTGGGATACATGTGCGATGATACTATCA
CTTATGAATGCCCAGTACTGTCGGCTGGTAATGATCCAGAAGACATCGACTGTTGGTGCACA
AAGTCAGCAGTCTACGTCAGGTATGGAAGATGCACCAAGACACGCCACTCAAGACGCAGTC
GGAGGTCACTGACAGTGCAGACACACGGAGAAAGCACTCTAGCGAACAAGAAGGGGGCTT
GGATGGACAGCACCAAGGCCACAAGGTATTTGGTAAAAACAGAATCATGGATCTTGAGGAA
CCCTGGATATGCCCTGGTGGCAGCCGTCATTGGTTGGATGCTTGGGAGCAACACCATGCA
GAGAGTTGTGTTTGTCGTGCTATTGCTTTTGGTGGCCCCAGCTTACAGC**TTCAACTGCCTT**
GGAATGAGCAACAGAGACTTTTTGGAAGGCGTGTCCGGAGCAACGTGGGTCGATTTGGT
TCTCGAAGGCGACAGTTGTGTAACGATAATGTCGAAAGACAAACCAACGATCGACGTCAA
GATGATGAACATGGAAGCGGCGAACCTCGCCGAAGTACGAAGTTATTGTTATTTGGCGAC
CGTAAGTGATCTCTCAACCAAAGCGGCGTGTCCGACAATGGGCGAAGCGCACAACGACA
AACGTGCGGATCCCGCATTTGTGTGCCGACAAGGCGTCGTCGACCGCGGATGGGGAAAC
GGATGTGGACTGTTCGGAAAAGGAAGCATCGACACGTGCGCAAAATTTGCATGCTCGAC
GAAAGCGATCGGACGAACGATTTTGAAAGAAAACATTAAATACGAAGTTGCGATTTTTGTT
CACGGACCGACAACAGTAGAATCTCACGGAAATTATTCGACGCAAGTCGGCGCGACACA
AGCCGGTCGACTGAGCATCACGCCCGCGGCGCCGTCGTATACGCTAAAACTCGGCGAAT
ACGGCGAAGTGACGGTTGATTGCGAACCTCGTTCCGGCATCGACACGAACGCGTACTAC
GTTATGACGGTCGGAACGAAGACTTTTTTAGTTCATCGCGAATGGTTCATGGATCTTAATCT
ACCTTGGAGCAGCGCGGGAAGCACGGTTTGGCGTAATCGCGAAACGTTGATGGAATTTG
AAGAACCTCACGCGACAAAACAGTCCGTTATCGCGTTGGGTTCACAAGAAGGCGCGCTA
CATCAAGCGTTGGCCGGCGCGATTCCTGTCGAATTTTCGAGCAATACAGTTAAGTTGACG
TCCGGTCATTTGAAATGTCGCGTGAAAATGGAAAAATTGCAATTGAAAGGAACGACATAC
GGTGTGTGTTCGAAAGCGTTCAAATTTCTCGGAACGCCCGCGGATACCGGTCACGGAAC
TGTTGTTTTAGAATTGCAATACACTGGAACGGACGGACCTTGTAAAGTTCCGATCTCATCG
GTCGCGTCATTGAACGATCTGACACCGGTCGGACGATTAGTGACTGTGAACCCGTTTGTT
TCGGTCGCGACGGCAAATGCAAAAGTTCTGATCGAATTGGAACCGCCGTTTGGCGACTC
GTACATCGTTGTTGGACGCGGCGAACAACAAATTAATCATCATTGGCACAAATCGGGAAG
CAGCATCGGAAAAGCGTTCACGACGACGCTCAAAGGCGCTCAGCGACTTGCCGCGCTC
GGTGATACGGCGTGGGATTTTGGATCCGTCGGCGGGGTTTTCACGTCGGTAGGGAAGGC
TGTTCATCAAGTATTCGGCGGCGCGTTTCGTTCGCTTTTTGGCGGAATGTCGTGGATTACT
CAAGGATTACTCGGCGCGCTTCTGTTGTGGATGGGAATTAACGCACGAGATCGATCGATC
GCGCTCACATTTCTCGCTGTCGGAGGAGTTCTGCTCTTCCTCTCCGTGAACGTGCACGCT
GACACTGGGTGTGCCATAGACATCAGCCGGCAAGAGCTGAGATGTGGAAGTGGAGTGTTC
ATACACAATGATGTGGAGGCTTGGATGGACCGGTACAAGTATTACCCTGAAACGCCACAAG
GCCTAGCCAAGATCATTCAGAAAGCTCATAAGGAAGGAGTGTGCGGTCTACGATCAGTTTC

File S1 WNV reference sequences

CAGACTGGAGCATCAAATGTGGGAAGCAGTGAAGGACGAGCTGAACACTCTTTTGAAGGA
GAATGGTGTGGACCTTAGTGTCGTGGTTGAGAAACAGGAGGGAATGTACAAGTCAGCACCT
AAACGCCTCACCGCCACCACGGAAAAATTGGAAATTGGCTGGAAGGCCTGGGGAAAGAGT
ATTTTATTTGCACCAGAACTCGCCAACAACACCTTTGTGGTTGATGGTCCGGAGACCAAGGA
ATGTCCGACTCAGAATCGCGCTTGGAATAGCTTAGAAGTGGAGGATTTTGGATTTGGTCTCA
CCAGCACTCGGATGTTCCTGAAGGTCAGAGAGAGCAACACAACTGAATGTGACTCGAAGAT
CATTGGAACGGCTGTCAAGAACAACTTGGCGATCCACAGTGACCTGTCCTATTGGATTGAA
AGCAGGCTCAATGATACGTGGAAGCTTGAAAGGGCAGTTCTGGGTGAAGTCAAATCATGTA
CGTGGCCTGAGACGCATACCTTGTGGGGCGATGGAATCCTTGAGAGTGACTTGATAATACC
AGTCACACTGGCGGGACCACGAAGCAATCACAATCGGAGACCTGGGTACAAGACACAAAA
CCAGGGCCCATGGGACGAAGGCCGGGTAGAGATTGACTTCGATTACTGCCCAGGAACTAC
GGTCACCCTGAGTGAGAGCTGCGGACACCGTGGACCTGCCACTCGCACCACCACAGAGA
GCGGAAAGTTGATAACAGATTGGTGCTGCAGGAGCTGCACCTTACCACCACTGCGCTACCA
AACTGACAGCGGCTGTTGGTATGGTATGGAGATCAGACCACAGAGACATGATGAAAAGACC
CTCGTGCAGTCACAAGTGAATGCTTATAATGCTGATATGATTGACCCTTTTCAGTTGGGCCTT
CTGGTCGTGTTCTTGGCCACCCAGGAGGTCCTTCGCAAGAGGTGGACAGCCAAGATCAGC
ATGCCAGCTATACTGATTGCTCTGCTAGTCCTGGTGTTTGGGGGCATTACTTACACTGATGT
GTTACGCTATGTCATCTTGGTGGGGGCAGCTTTCGCAGAATCTAATTCGGGAGGAGACGTG
GTACACTTGGCGCTCATGGCGACCTTCAAGATACAACCAGTGTTTATGGTGGCATCGTTTCT
CAAAGCGAGATGGACCAACCAGGAGAACATTTTGTTGATGTTGGCGGCTGTTTTCTTTCAAA
TGGCTTATCACGATGCCCGCCAAATTCTGCTCTGGGAGATCCCTGATGTGTTGAATTCACTG
GCGGTAGCTTGGATGATACTGAGAGCCATAACATTCACAACGACATCAAACGTGGTTGTTCC
GCTGCTAGCCCTGCTAACACCCGGGCTGAGATGCTTGAATCTGGATGTGTACAGGATACTG
CTGTTGATGGTCGGAATAGGCAGCTTGATCAGGGAGAAGAGGAGTGCAGCCGCAAAAAAG
AAAGGAGCAAGTCTGCTATGCTTGGCTCTAGCCTCAACAGGACTTTTCAACCCCATGATCCT
TGCTGCTGGACTGATTGCATGTGATCCCAACCGTAAACGCGGATGGCCCGCAACTGAAGTG
ATGACAGCTGTCGGCCTAATGTTTGCCATCGTCGGAGGGCTGGCAGAGCTTGACATTGACT
CCATGGCCATTCCAATGACTATCGCGGGGCTCATGTTTGCTGCTTTCGTGATTTCTGGGAAA
TCAACAGATATGTGGATTGAGAGAACGGCGGACATTTCCTGGGAAAGTGATGCAGAAATTAC
AGGCTCGAGCGAAAGAGTTGATGTGCGGCTTGATGATGATGGAAACTTCCAGCTCATGAAT
GATCCAGGAGCACCTTGGAAGATATGGATGCTCAGAATGGTCTGTCTCGCGATTAGTGCGT
ACACCCCCTGGGCAATCTTGCCCTCAGTAGTTGGATTTTGGATAACTCTCCAATACACAAAG
AGAGGAGGCGTGTTGTGGGACACTCCCTCACCAAAGGAGTACAAAAAGGGGGACACGACC
ACCGGCGTCTACAGGATCATGACTCGTGGGCTGCTCGGCAGTTATCAAGCAGGAGCGGGC
GTGATGGTTGAAGGTGTTTTCCACACCCTTTGGCATACAACAAAAGGAGCCGCTTTGATGA
GCGGAGAGGGCCGCCTGGACCCATACTGGGGCAGTGTCAAGGAGGATCGACTTTGTTACG
GAGGACCCTGGAAATTGCAGCACAAGTGGAACGGGCAGGATGAGGTGCAGATGATTGTGG
TGGAACCTGGCAAGAACGTTAAGAACGTCCAGACGAAACCAGGGGTGTTCAAAACACCTG
AAGGAGAAATCGGGGCCGTGACTTTGGACTTCCCCACTGGAACATCAGGCTCACCAATAGT
GGACAAAAACGGTGATGTGATTGGGCTTTATGGCAATGGAGTCATAATGCCCAACGGCTCAT
ACATAAGCGCGATAGTGCAGGGTGAAAGGATGGATGAGCCAATCCCAGCCGGATTCGAACC
TGAGATGCTGAGGAAAAAACAGATCACTGTACTGGATCTCCATCCCGGCGCCGGTAAAACA
AGGAGGATTCTGCCACAGATCATCAAAGAGGCCATAAACAGAAGACTGAGAACAGCCGTGC
TAGCGCCAACCAGGGTTGTGGCTGCTGAGATGGCTGAAGCACTGAGAGGACTGCCCATCC
GGTACCAGACATCCGCAGTGCCCAGAGAACATAATGGAAATGAGATTGTTGATGTCATGTGT
CATGCTACCCTCACCCACAGGCTGATGTCTCCTCACAGGGTGCCGAACTACAACCTGTTCG
TGATGGATGAGGCTCATTTCACCGACCCAGCTAGCATTGCAGCAAGAGGTTACATTTCCACA

File S1 WNV reference sequences

AAGGTCGAGCTAGGGGAGGCGGCGGCAATATTCATGACAGCCACCCCACCAGGCACTTCA
GATCCATTCCCAGAGTCCAATTCACCAATTTCCGACTTACAGACTGAGATCCCGGATCGAGC
TTGGAACTCTGGATACGAATGGATCACAGAATACACCGGGAAGACGGTTTGGTTTGTGCCTA
GTGTCAAGATGGGGAATGAGATTGCCCTTTGCCTACAACGTGCTGGAAAGAAAGTAGTCCA
ATTGAACAGAAAGTCGTACGAGACGGAGTACCCAAAATGTAAGAACGATGATTGGGACTTTG
TTATCACAACAGACATATCTGAAATGGGGGCTAACTTCAAGGCGAGCAGGGTGATTGACAG
CCGGAAGAGTGTGAAACCAACCATCATAACAGAAGGAGAAGGGAGAGTGATCCTGGGAGA
ACCATCTGCAGTGACAGCAGCTAGTGCCGCCCAGAGACGTGGACGTATCGGTAGAAATCC
GTCGCAAGTTGGTGATGAGTACTGTTATGGGGGCACACGAATGAAGACGACTCGAACTTC
GCCCATTGGACTGAGGCACGAATCATGCTGGACAACATCAACATGCCAAACGGACTGATCG
CTCAATTCTACCAACCAGAGCGTGAGAAGGTATATACCATGGATGGGGAATACCGGCTCAGA
GGAGAAGAGAGAAAAAACTTTCTGGAACTGTTGAGGACTGCAGATCTGCCAGTTTGGCTGG
CTTACAAGGTTGCAGCGGCTGGAGTGTCATACCACGACCGGAGGTGGTGCTTTGATGGTC
CTAGGACAAACACAATTTTAGAAGACAACAACGAAGTGGAAGTCATCACGAAGCTTGGTGA
AAGGAAGATTCTGAGGCCGCGCTGGATTGATGCCAGGGTGTACTCGGATCACCAGGCACT
AAAGGCGTTCAAGGACTTCGCCTCGGGAAAACGTTCTCAGATAGGGCTCATTGAGGTTCTG
GGAAAGATGCCTGAGCACTTCATGGGGAAGACATGGGAAGCACTTGACACCATGTACGTTG
TGGCCACTGCAGAGAAAGGAGGAAGAGCTCACAGAATGGCCCTGGAGGAACTGCCAGATG
CTCTTCAGACAATTGCCTTGATTGCCTTATTGAGTGTGATGACCATGGGAGTATTCTTCCTCC
TCATGCAGCGGAAGGGCATTGGAAAGATAGGTTTGGGAGGCGCTGTCTTGGGAGTCGCGA
CCTTTTTCTGTTGGATGGCTGAAGTTCCAGGAACGAAGATCGCCGGAATGTTGCTGCTCTC
CCTTCTCTTGATGATTGTGCTAATTCCTGAGCCAGAGAAGCAACGTTCGCAGACAGACAAC
CAGCTAGCCGTGTTCCTGATTTGTGTCATGACCCTTGTGAGCGCAGTGGCAGCCAACGAGA
TGGGTTGGCTAGATAAGACCAAGAGTGACATAAGCAGTTTGTTTGGGCAAAGAATTGAGGT
CAAGGAGAATTTCAGCATGGGAGAGTTTCTTCTGGACTTGAGGCCGGCAACAGCCTGGTC
ACTGTACGCTGTGACAACAGCGGTCCTCACTCCACTGCTAAAGCATTTGATCACGTCAGATT
ACATCAACACCTCATTGACCTCAATAAACGTTCAGGCAAGTGCACTATTCACACTCGCGCGA
GGCTTCCCCTTCGTCGATGTTGGAGTGTCGGCTCTCCTGCTAGCAGCCGGATGCTGGGGA
CAAGTCACCCTCACCGTTACGGTAACAGCGGCAACACTCCTTTTTTGCCACTATGCCTACAT
GGTTCCCGGTTGGCAAGCTGAGGCAATGCGCTCAGCCCAGCGGCGGACAGCGGCCGGAA
TCATGAAGAACGCTGTAGTGGATGGCATCGTGGCCACGGACGTCCCAGAATTAGAGCGCA
CCACACCCATCATGCAGAAGAAAGTTGGACAGATCATGCTGATCTTGGTGTCTCTAGCTGCA
GTAGTAGTGAACCCGTCTGTGAAGACAGTACGAGAAGCCGGAATTTTGATCACGGCCGCAG
CGGTGACGCTTTGGGAGAATGGAGCAAGCTCTGTTTGGAACGCAACAACTGCCATCGGAC
TCTGCCACATCATGCGTGGGGGTTGGTTGTCATGTCTATCCATAACATGGACACTCATAAAG
AACATGGAAAAACCAGGACTAAAAAGAGGTGGGGCAAAAGGACGCACCTTGGGAGAGGTT
TGGAAAGAAAGACTCAACCAGATGACAAAAGAAGAGTTCACTAGGTACCGCAAAGAGGCCA
TCATCGAAGTCGATCGCTCAGCGGCAAAACACGCCAGGAAAGAAGGCAATGTCACTGGAG
GGCATCCAGTCTCTAGGGGCACAGCAAAACTGAGATGGCTGGTCGAACGGAGGTTTCTCG
AACCGGTCGGAAAAGTGATTGACCTTGGATGTGGAAGAGGCGGTTGGTGTTACTATATGGC
AACCCAAAAAAGAGTCCAAGAAGTCAGAGGGTACACAAAGGGCGGTCCCGGACATGAAGA
GCCCCAACTAGTGCAAAGTTATGGATGGAACATTGTCACCATGAAGAGTGGAGTGGATGTG
TTCTACAGACCTTCTGAGTGTTGTGACACCCTCCTTTGTGACATCGGAGAGTCCTCGTCAA
GTGCTGAGGTTGAAGAGCATAGGACGATTCGGGTCCTTGAAATGGTTGAGGACTGGCTGC
ACCGAGGGCCAAGGGAATTTTGCGTGAAGGTGCTCTGTCCCTACATGCCGAAAGTCATAGA
GAAGATGGAGCTGCTCCAACGCCGGTATGGGGGGGGACTGGTCAGAAACCCACTCTCACG
GAATTCCACGCACGAGATGTATTGGGTGAGTCGAGCTTCAGGCAATGTGGTACATTCAGTG

File S1 WNV reference sequences

AATATGACCAGCCAGGTGCTCCTAGGAAGAATGGAAAAAAGGACCTGGAAGGGACCCCAAT
ACGAGGAAGATGTAAACTTGGGAAGTGGAACCAGGGCGGTGGGAAAACCCCTGCTCAACT
CAGACACCAGTAAAATCAAGAACAGGATTGAACGACTCAGGCGTGAGTACAGTTCGACGTG
GCACCACGATGAGAACCACCCATATAGAACCTGGAACTATCACGGCAGTTATGATGTGAAGC
CCACAGGCTCCGCCAGTTCGCTGGTCAATGGAGTGGTCAGGCTCCTCTCAAAACCATGGG
ACACCATCACGAATGTTACCACCATGGCCATGACTGACACTACTCCCTTCGGGCAGCAGCG
AGTGTTCAAAGAGAAGGTGGACACGAAAGCTCCTGAACCGCCAGAAGGAGTGAAGTACGT
GCTCAACGAGACCACCAACTGGTTGTGGGCGTTTTTGGCCAGAGAAAAACGTCCCAGAAT
GTGCTCTCGAGAGGAATTCATAAGAAAGGTCAACAGCAATGCAGCTTTGGGTGCCATGTTT
GAAGAGCAGAATCAATGGAGGAGCGCCAGAGAAGCAGTTGAAGATCCAAAATTTTGGGAGA
TGGTGGATGAGGAGCGCGAGGCACATCTGCGGGGGGAATGTCACACTTGCATTTACAACAT
GATGGGAAAGAGAGAGAAAAAACCCGGAGAGTTCGGAAAGGCCAAGGGAAGCAGAGCCA
TTTGGTTCATGTGGCTCGGAGCTCGCTTTCTGGAGTTCGAGGCTCTGGGTTTTCTCAATGA
AGACCACTGGCTTGGAAGAAAGAACTCAGGAGGAGGTGTCGAGGGCTTGGGCCTCCAAAA
ACTGGGTTACATCCTGCGTGAAGTTGGCACCCGGCCTGGGGGCAAGATCTATGCTGATGAC
ACAGCTGGCTGGGACACCCGCATCACGAGAGCTGACTTGGAAAATGAAGCTAAGGTGCTT
GAGCTGCTTGATGGGGAACATCGGCGTCTTGCCAGGGCCATCATTGAGCTCACCTATCGTC
ACAAAGTTGTGAAAGTGATGCGCCCGGCTGCTGATGGAAGAACCGTCATGGATGTTATCTC
CAGAGAAGATCAGAGGGGGAGTGGACAAGTTGTCACCTACGCCCTAAACACTTTCACCAAC
CTGGCCGTCCAGCTGGTGAGGATGATGGAAGGGGAAGGAGTGATTGGCCCAGATGATGTG
GAGAAACTCACAAAAGGGAAAGGACCCAAAGTCAGGACCTGGCTGTTTGAGAATGGGGAA
GAAAGACTCAGCCGCATGGCTGTCAGTGGAGATGACTGTGTGGTAAAGCCCCTGGACGAT
CGCTTTGCCACCTCGCTCCACTTCCTCAATGCTATGTCAAAGGTTCGCAAAGACATCCAAGA
GTGGAAACCGTCAACTGGATGGTATGATTGGCAGCAGGTTCCATTTTGCTCAAACCATTTCA
CTGAATTGATCATGAAAGATGGAAGAACACTGGTGGTTCCATGCCGAGGACAGGATGAATT
GGTAGGCAGAGCTCGCATATCTCCAGGGGCCGGATGGAACGTCCGCGACACTGCTTGTCT
GGCTAAGTCTTATGCCCAGATGTGGCTGCTTCTGTACTTCCACAGAAGAGACCTGCGGCTC
ATGGCCAACGCCATTTGCTCCGCTGTCCCTGTGAATTGGGTCCCTACCGGAAGAACCACGT
GGTCCATCCATGCAGGAGGAGAGTGGATGACAACAGAGGACATGTTGGAGGTCTGGAACC
GTGTTTGGATAGAGGAGAATGAATGGATGGAAGACAAAACCCCAGTGGAGAAATGGAGTGA
CGTCCCATATTCAGGAAAACGAGAGGACATCTGGTGTGGCAGCCTGATTGGCACAAGAGCC
CGAGCCACGTGGGCAGAAAACATCCAGGTGGCTATCAACCAAGTCAGAGCAATCATCGGA
GATGAGAAGTATGTGGACTACATGAGTTCACTAAAGAGATATGAAGACACAACTTTGGTTGA
GGACACAGTACTGTAGATATTTAATCAATTGTAAATAGACAATATAAGTATGCATAAAAGTGTAG
TTTTATAGTAGTATTTAGTGGTGTTAGTGTAAATAGTTAAGAAAATTTTGAGGAGAAAGTCAGG
CCGGGAAGTTCCCGCCACCGGAAGTTGAGTAGACGGTGCTGCCTGCGACTCAACCCCAG
GAGGACTGGGTGAACAAAGCCGCGAAGTGATCCATGTAAGCCCTCAGAACCGTCTCGGAA
GGAGGACCCCACATGTTGTAACTTCAAAGCCCAATGTCAGACCACGCTACGGCGTGCTACT
CTGCGGAGAGTGCAGTCTGCGATAGTGCCCCAGGAGGACTGGGTTAACAAAGGCAAACCA
ACGCCCCACGCGGCCCTAGCCCCGGTAATGGTGTTAACCAGGGCGAAAGGACTAGAGGTT
AGAGGAGACCCCGCGGTTTAAAGTGCACGGCCCAGCCTGGCTGAAGCTGTAGGTCAGGG
GAAGGACTAGAGGTTAGTGGAGACCCCGTGCCACAAAACACCACAACAAAACAGCATATTG
ACACCTGGGATAGACTAGGAGATCTTCTGCTCTGCACAACCAGCCACACGGCACAGTGCG
CCGACAATGGTGGCTGGTGGTGCGAGAACACAGGATCT

File S1 WNV reference sequences

> E/NS1+CG

E sequences are in green.
NS1 sequences are in red.

AGTAGTTCGCCTGTGTGAGCTGACAAACTTAGTAGTGTTTGTGAGGATTAACAACAATTAAC
ACAGTGCGAGCTGTTTCTTAGCACGAAGATCTCGATGTCTAAGAAACCAGGAGGGCCCGGC
AAGAGCCGGGCTGTCAATATGCTAAAACGCGGAATGCCCCGCGTGTTGTCCTTGATTGGAC
TGAAGAGGGCTATGTTGAGCCTGATCGACGGCAAGGGGCCAATACGATTTGTGTTGGCTCT
CTTGGCGTTCTTCAGGTTCACAGCAATTGCTCCGACCCGAGCAGTGCTGGATCGATGGAGA
GGTGTGAACAAACAAACAGCGATGAAACACCTTCTGAGTTTTAAGAAGGAACTAGGGACCT
TGACCAGTGCTATCAATCGGCGGAGCTCAAAACAAAAGAAAAGAGGAGGAAAGACCGGAAT
TGCAGTCATGATTGGCCTGATCGCCAGCGTAGGAGCAGTTACCCTCTCTAACTTCCAAGGG
AAGGTGATGATGACGGTAAATGCTACTGACGTCACAGATGTCATCACGATTCCAACAGCTGC
TGGAAAGAACCTATGCATTGTCAGAGCAATGGATGTGGGATACATGTGCGATGATACTATCA
CTTATGAATGCCCAGTACTGTCGGCTGGTAATGATCCAGAAGACATCGACTGTTGGTGCACA
AAGTCAGCAGTCTACGTCAGGTATGGAAGATGCACCAAGACACGCCACTCAAGACGCAGTC
GGAGGTCACTGACAGTGCAGACACACGGAGAAAGCACTCTAGCGAACAAGAAGGGGGCTT
GGATGGACAGCACCAAGGCCACAAGGTATTTGGTAAAAACAGAATCATGGATCTTGAGGAA
CCCTGGATATGCCCTGGTGGCAGCCGTCATTGGTTGGATGCTTGGGAGCAACACCATGCA
GAGAGTTGTGTTTGTCGTGCTATTGCTTTTGGTGGCCCCAGCTTACAGC**TTCAACTGCCTT**
GGAATGAGCAACAGAGACTTTTTGGAAGGCGTGTCCGGAGCAACGTGGGTCGATTTGGT
TCTCGAAGGCGACAGTTGTGTAACGATAATGTCGAAAGACAAACCAACGATCGACGTCAA
GATGATGAACATGGAAGCGGCGAACCTCGCCGAAGTACGAAGTTATTGTTATTTGGCGAC
CGTAAGTGATCTCTCAACCAAAGCGGCGTGTCCGACAATGGGCGAAGCGCACAACGACA
AACGTGCGGATCCCGCATTTGTGTGCCGACAAGGCGTCGTCGACCGCGGATGGGGAAAC
GGATGTGGACTGTTCGGAAAAGGAAGCATCGACACGTGCGCAAAATTTGCATGCTCGAC
GAAAGCGATCGGACGAACGATTTTGAAAGAAAACATTAAATACGAAGTTGCGATTTTTGTT
CACGGACCGACAACAGTAGAATCTCACGGAAATTATTCGACGCAAGTCGGCGCGACACA
AGCCGGTCGACTGAGCATCACGCCCGCGGCGCCGTCGTATACGCTAAAACTCGGCGAAT
ACGGCGAAGTGACGGTTGATTGCGAACCTCGTTCCGGCATCGACACGAACGCGTACTAC
GTTATGACGGTCGGAACGAAGACTTTTTTAGTTCATCGCGAATGGTTCATGGATCTTAATCT
ACCTTGGAGCAGCGCGGGAAGCACGGTTTGGCGTAATCGCGAAACGTTGATGGAATTTG
AAGAACCTCACGCGACAAAACAGTCCGTTATCGCGTTGGGTTCACAAGAAGGCGCGCTA
CATCAAGCGTTGGCCGGCGCGATTCCTGTCGAATTTTCGAGCAATACAGTTAAGTTGACG
TCCGGTCATTTGAAATGTCGCGTGAAAATGGAAAAATTGCAATTGAAAGGAACGACATAC
GGTGTGTGTTCGAAAGCGTTCAAATTTCTCGGAACGCCCGCGGATACCGGTCACGGAAC
TGTTGTTTTAGAATTGCAATACACTGGAACGGACGGACCTTGTAAAGTTCCGATCTCATCG
GTCGCGTCATTGAACGATCTGACACCGGTCGGACGATTAGTGACTGTGAACCCGTTTGTT
TCGGTCGCGACGGCAAATGCAAAAGTTCTGATCGAATTGGAACCGCCGTTTGGCGACTC
GTACATCGTTGTTGGACGCGGCGAACAACAAATTAATCATCATTGGCACAAATCGGGAAG
CAGCATCGGAAAAGCGTTCACGACGACGCTCAAAGGCGCTCAGCGACTTGCCGCGCTC
GGTGATACGGCGTGGGATTTTGGATCCGTCGGCGGGGTTTTCACGTCGGTAGGGAAGGC
TGTTCATCAAGTATTCGGCGGCGCGTTTCGTTCGCTTTTTGGCGGAATGTCGTGGATTACT
CAAGGATTACTCGGCGCGCTTCTGTTGTGGATGGGAATTAACGCACGAGATCGATCGATC
GCGCTCACATTTCTCGCTGTCGGAGGAGTTCTGCTCTTCCTCTCCGTGAACGTGCACGCT
GACACTGGGTGTGCCATAGACATCAGCCGGCAAGAGCTACGATGCGGAAGTGGCGTGTT
TATTCACAACGACGTCGAAGCGTGGATGGATCGGTACAAGTATTATCCGGAAACGCCGCA

File S1 WNV reference sequences

AGGTCTTGCGAAAATTATACAGAAAGCGCACAAAGAAGGCGTTTGCGGTCTACGATCGGT
TTCTCGACTCGAACATCAAATGTGGGAAGCAGTGAAGGACGAGCTAAACACGCTTTTAAA
AGAAAACGGTGTCGATCTCAGCGTCGTCGTTGAAAAACAAGAGGGAATGTACAAGTCGG
CGCCGAAACGACTCACCGCAACGACGGAAAAATTGGAAATTGGATGGAAGGCGTGGGG
AAAAAGCATTTTATTCGCGCCTGAACTCGCGAACAATACGTTCGTCGTCGACGGTCCTGA
AACGAAAGAATGTCCAACGCAAAATCGCGCGTGGAACAGTTTAGAAGTCGAGGATTTCG
GATTCGGTCTCACGAGCACGCGGATGTTTCTTAAGGTCCGAGAGAGCAACACGACGGAA
TGCGACTCGAAAATAATTGGAACGGCGGTTAAAAACAATTTGGCGATACACAGCGATCTT
TCTTATTGGATCGAAAGTCGGCTGAACGATACTTGGAAACTCGAACGCGCCGTTCTCGGC
GAAGTAAAATCTTGCACGTGGCCGGAGACGCATACGTTGTGGGGTGACGGAATTCTCGA
AAGCGATTTGATAATTCCCGTGACCCTCGCGGGACCGCGAAGCAATCACAATCGTCGACC
GGGGTATAAAACGCAAAATCAGGGTCCATGGGACGAAGGACGCGTCGAGATTGATTTCG
ATTATTGTCCCGGAACAACGGTGACACTGAGTGAGAGTTGTGGACATCGCGGACCCGCG
ACGCGAACGACGACGGAGAGTGGAAAGTTGATTACGGATTGGTGCTGTCGTAGTTGCAC
CTTGCCTCCGCTTCGTTACCAAACCGACAGCGGATGTTGGTACGGGATGGAAATACGACC
GCAACGACACGATGAAAAGACCCTCGTGCAGTCACAAGTGAATGCTTATAATGCTGATATG
ATTGACCCTTTTCAGTTGGGCCTTCTGGTCGTGTTCTTGGCCACCCAGGAGGTCCTTCGCA
AGAGGTGGACAGCCAAGATCAGCATGCCAGCTATACTGATTGCTCTGCTAGTCCTGGTGTTT
GGGGGCATTACTTACACTGATGTGTTACGCTATGTCATCTTGGTGGGGGCAGCTTTCGCAG
AATCTAATTCGGGAGGAGACGTGGTACACTTGGCGCTCATGGCGACCTTCAAGATACAACC
AGTGTTTATGGTGGCATCGTTTCTCAAAGCGAGATGGACCAACCAGGAGAACATTTTGTTGA
TGTTGGCGGCTGTTTTCTTTCAAATGGCTTATCACGATGCCCGCCAAATTCTGCTCTGGGAG
ATCCCTGATGTGTTGAATTCACTGGCGGTAGCTTGGATGATACTGAGAGCCATAACATTCAC
AACGACATCAAACGTGGTTGTTCCGCTGCTAGCCCTGCTAACACCCGGGCTGAGATGCTTG
AATCTGGATGTGTACAGGATACTGCTGTTGATGGTCGGAATAGGCAGCTTGATCAGGGAGA
AGAGGAGTGCAGCCGCAAAAAAGAAAGGAGCAAGTCTGCTATGCTTGGCTCTAGCCTCAA
CAGGACTTTTCAACCCCATGATCCTTGCTGCTGGACTGATTGCATGTGATCCCAACCGTAAA
CGCGGATGGCCCGCAACTGAAGTGATGACAGCTGTCGGCCTAATGTTTGCCATCGTCGGA
GGGCTGGCAGAGCTTGACATTGACTCCATGGCCATTCCAATGACTATCGCGGGGCTCATGT
TTGCTGCTTTCGTGATTTCTGGGAAATCAACAGATATGTGGATTGAGAGAACGGCGGACATT
TCCTGGGAAAGTGATGCAGAAATTACAGGCTCGAGCGAAAGAGTTGATGTGCGGCTTGATG
ATGATGGAAACTTCCAGCTCATGAATGATCCAGGAGCACCTTGGAAGATATGGATGCTCAGA
ATGGTCTGTCTCGCGATTAGTGCGTACACCCCCTGGGCAATCTTGCCCTCAGTAGTTGGATT
TTGGATAACTCTCCAATACACAAAGAGAGGAGGCGTGTTGTGGGACACTCCCTCACCAAAG
GAGTACAAAAAGGGGGACACGACCACCGGCGTCTACAGGATCATGACTCGTGGGCTGCTC
GGCAGTTATCAAGCAGGAGCGGGCGTGATGGTTGAAGGTGTTTTCCACACCCTTTGGCATA
CAACAAAAGGAGCCGCTTTGATGAGCGGAGAGGGCCGCCTGGACCCATACTGGGGCAGT
GTCAAGGAGGATCGACTTTGTTACGGAGGACCCTGGAAATTGCAGCACAAGTGGAACGGG
CAGGATGAGGTGCAGATGATTGTGGTGGAACCTGGCAAGAACGTTAAGAACGTCCAGACG
AAACCAGGGGTGTTCAAAACACCTGAAGGAGAAATCGGGGCCGTGACTTTGGACTTCCCC
ACTGGAACATCAGGCTCACCAATAGTGGACAAAAACGGTGATGTGATTGGGCTTTATGGCAA
TGGAGTCATAATGCCCAACGGCTCATACATAAGCGCGATAGTGCAGGGTGAAAGGATGGAT
GAGCCAATCCCAGCCGGATTCGAACCTGAGATGCTGAGGAAAAAACAGATCACTGTACTGG
ATCTCCATCCCGGCGCCGGTAAAACAAGGAGGATTCTGCCACAGATCATCAAAGAGGCCAT
AAACAGAAGACTGAGAACAGCCGTGCTAGCGCCAACCAGGGTTGTGGCTGCTGAGATGGC
TGAAGCACTGAGAGGACTGCCCATCCGGTACCAGACATCCGCAGTGCCCAGAGAACATAAT
GGAAATGAGATTGTTGATGTCATGTGTCATGCTACCCTCACCCACAGGCTGATGTCTCCTCA

File S1 WNV reference sequences

CAGGGTGCCGAACTACAACCTGTTCGTGATGGATGAGGCTCATTTCACCGACCCAGCTAGC
ATTGCAGCAAGAGGTTACATTTCCACAAAGGTCGAGCTAGGGGAGGCGGCGGCAATATTCA
TGACAGCCACCCCACCAGGCACTTCAGATCCATTCCCAGAGTCCAATTCACCAATTTCCGA
CTTACAGACTGAGATCCCGGATCGAGCTTGGAACTCTGGATACGAATGGATCACAGAATACA
CCGGGAAGACGGTTTGGTTTGTGCCTAGTGTCAAGATGGGGAATGAGATTGCCCTTTGCCT
ACAACGTGCTGGAAAGAAAGTAGTCCAATTGAACAGAAAGTCGTACGAGACGGAGTACCCA
AAATGTAAGAACGATGATTGGGACTTTGTTATCACAACAGACATATCTGAAATGGGGGCTAAC
TTCAAGGCGAGCAGGGTGATTGACAGCCGGAAGAGTGTGAAACCAACCATCATAACAGAAG
GAGAAGGGAGAGTGATCCTGGGAGAACCATCTGCAGTGACAGCAGCTAGTGCCGCCCAGA
GACGTGGACGTATCGGTAGAAATCCGTCGCAAGTTGGTGATGAGTACTGTTATGGGGGGCA
CACGAATGAAGACGACTCGAACTTCGCCCATTGGACTGAGGCACGAATCATGCTGGACAAC
ATCAACATGCCAAACGGACTGATCGCTCAATTCTACCAACCAGAGCGTGAGAAGGTATATAC
CATGGATGGGGAATACCGGCTCAGAGGAGAAGAGAGAAAAAACTTTCTGGAACTGTTGAGG
ACTGCAGATCTGCCAGTTTGGCTGGCTTACAAGGTTGCAGCGGCTGGAGTGTCATACCACG
ACCGGAGGTGGTGCTTTGATGGTCCTAGGACAAACACAATTTTAGAAGACAACAACGAAGT
GGAAGTCATCACGAAGCTTGGTGAAAGGAAGATTCTGAGGCCGCGCTGGATTGATGCCAG
GGTGTACTCGGATCACCAGGCACTAAAGGCGTTCAAGGACTTCGCCTCGGGAAAACGTTCT
CAGATAGGGCTCATTGAGGTTCTGGGAAAGATGCCTGAGCACTTCATGGGGAAGACATGGG
AAGCACTTGACACCATGTACGTTGTGGCCACTGCAGAGAAAGGAGGAAGAGCTCACAGAAT
GGCCCTGGAGGAACTGCCAGATGCTCTTCAGACAATTGCCTTGATTGCCTTATTGAGTGTG
ATGACCATGGGAGTATTCTTCCTCCTCATGCAGCGGAAGGGCATTGGAAAGATAGGTTTGG
GAGGCGCTGTCTTGGGAGTCGCGACCTTTTTCTGTTGGATGGCTGAAGTTCCAGGAACGA
AGATCGCCGGAATGTTGCTGCTCTCCCTTCTCTTGATGATTGTGCTAATTCCTGAGCCAGAG
AAGCAACGTTCGCAGACAGACAACCAGCTAGCCGTGTTCCTGATTTGTGTCATGACCCTTG
TGAGCGCAGTGGCAGCCAACGAGATGGGTTGGCTAGATAAGACCAAGAGTGACATAAGCA
GTTTGTTTGGGCAAAGAATTGAGGTCAAGGAGAATTTCAGCATGGGAGAGTTTCTTCTGGA
CTTGAGGCCGGCAACAGCCTGGTCACTGTACGCTGTGACAACAGCGGTCCTCACTCCACT
GCTAAAGCATTTGATCACGTCAGATTACATCAACACCTCATTGACCTCAATAAACGTTCAGGC
AAGTGCACTATTCACACTCGCGCGAGGCTTCCCCTTCGTCGATGTTGGAGTGTCGGCTCTC
CTGCTAGCAGCCGGATGCTGGGGACAAGTCACCCTCACCGTTACGGTAACAGCGGCAACA
CTCCTTTTTTGCCACTATGCCTACATGGTTCCCGGTTGGCAAGCTGAGGCAATGCGCTCAG
CCCAGCGGCGGACAGCGGCCGGAATCATGAAGAACGCTGTAGTGGATGGCATCGTGGCCA
CGGACGTCCCAGAATTAGAGCGCACCACACCCATCATGCAGAAGAAAGTTGGACAGATCAT
GCTGATCTTGGTGTCTCTAGCTGCAGTAGTAGTGAACCCGTCTGTGAAGACAGTACGAGAA
GCCGGAATTTTGATCACGGCCGCAGCGGTGACGCTTTGGGAGAATGGAGCAAGCTCTGTT
TGGAACGCAACAACTGCCATCGGACTCTGCCACATCATGCGTGGGGGTTGGTTGTCATGTC
TATCCATAACATGGACACTCATAAAGAACATGGAAAAACCAGGACTAAAAAGAGGTGGGGCA
AAAGGACGCACCTTGGGAGAGGTTTGGAAAGAAAGACTCAACCAGATGACAAAAGAAGAG
TTCACTAGGTACCGCAAAGAGGCCATCATCGAAGTCGATCGCTCAGCGGCAAAACACGCCA
GGAAAGAAGGCAATGTCACTGGAGGGCATCCAGTCTCTAGGGGCACAGCAAAACTGAGAT
GGCTGGTCGAACGGAGGTTTCTCGAACCGGTCGGAAAAGTGATTGACCTTGGATGTGGAA
GAGGCGGTTGGTGTTACTATATGGCAACCCAAAAAAGAGTCCAAGAAGTCAGAGGGTACAC
AAAGGGCGGTCCCGGACATGAAGAGCCCCAACTAGTGCAAAGTTATGGATGGAACATTGTC
ACCATGAAGAGTGGAGTGGATGTGTTCTACAGACCTTCTGAGTGTTGTGACACCCTCCTTT
GTGACATCGGAGAGTCCTCGTCAAGTGCTGAGGTTGAAGAGCATAGGACGATTCGGGTCC
TTGAAATGGTTGAGGACTGGCTGCACCGAGGGCCAAGGGAATTTTGCGTGAAGGTGCTCT
GTCCCTACATGCCGAAAGTCATAGAGAAGATGGAGCTGCTCCAACGCCGGTATGGGGGG

File S1 WNV reference sequences

GACTGGTCAGAAACCCACTCTCACGGAATTCCACGCACGAGATGTATTGGGTGAGTCGAGC
TTCAGGCAATGTGGTACATTCAGTGAATATGACCAGCCAGGTGCTCCTAGGAAGAATGGAAA
AAAGGACCTGGAAGGGACCCCAATACGAGGAAGATGTAAACTTGGGAAGTGGAACCAGGG
CGGTGGGAAAACCCCTGCTCAACTCAGACACCAGTAAAATCAAGAACAGGATTGAACGACT
CAGGCGTGAGTACAGTTCGACGTGGCACCACGATGAGAACCACCCATATAGAACCTGGAAC
TATCACGGCAGTTATGATGTGAAGCCCACAGGCTCCGCCAGTTCGCTGGTCAATGGAGTGG
TCAGGCTCCTCTCAAAACCATGGGACACCATCACGAATGTTACCACCATGGCCATGACTGA
CACTACTCCCTTCGGGCAGCAGCGAGTGTTCAAAGAGAAGGTGGACACGAAAGCTCCTGA
ACCGCCAGAAGGAGTGAAGTACGTGCTCAACGAGACCACCAACTGGTTGTGGGCGTTTTT
GGCCAGAGAAAAACGTCCCAGAATGTGCTCTCGAGAGGAATTCATAAGAAAGGTCAACAGC
AATGCAGCTTTGGGTGCCATGTTTGAAGAGCAGAATCAATGGAGGAGCGCCAGAGAAGCA
GTTGAAGATCCAAAATTTTGGGAGATGGTGGATGAGGAGCGCGAGGCACATCTGCGGGGG
GAATGTCACACTTGCATTTACAACATGATGGGAAAGAGAGAGAAAAAACCCGGAGAGTTCG
GAAAGGCCAAGGGAAGCAGAGCCATTTGGTTCATGTGGCTCGGAGCTCGCTTTCTGGAGT
TCGAGGCTCTGGGTTTTCTCAATGAAGACCACTGGCTTGGAAGAAAGAACTCAGGAGGAG
GTGTCGAGGGCTTGGGCCTCCAAAAACTGGGTTACATCCTGCGTGAAGTTGGCACCCGGC
CTGGGGGCAAGATCTATGCTGATGACACAGCTGGCTGGGACACCCGCATCACGAGAGCTG
ACTTGGAAAATGAAGCTAAGGTGCTTGAGCTGCTTGATGGGGAACATCGGCGTCTTGCCAG
GGCCATCATTGAGCTCACCTATCGTCACAAAGTTGTGAAAGTGATGCGCCCGGCTGCTGAT
GGAAGAACCGTCATGGATGTTATCTCCAGAGAAGATCAGAGGGGGAGTGGACAAGTTGTCA
CCTACGCCCTAAACACTTTCACCAACCTGGCCGTCCAGCTGGTGAGGATGATGGAAGGGG
AAGGAGTGATTGGCCCAGATGATGTGGAGAAACTCACAAAAGGGAAAGGACCCAAAGTCA
GGACCTGGCTGTTTGAGAATGGGGAAGAAAGACTCAGCCGCATGGCTGTCAGTGGAGATG
ACTGTGTGGTAAAGCCCCTGGACGATCGCTTTGCCACCTCGCTCCACTTCCTCAATGCTAT
GTCAAAGGTTCGCAAAGACATCCAAGAGTGGAAACCGTCAACTGGATGGTATGATTGGCAG
CAGGTTCCATTTTGCTCAAACCATTTCACTGAATTGATCATGAAAGATGGAAGAACACTGGT
GGTTCCATGCCGAGGACAGGATGAATTGGTAGGCAGAGCTCGCATATCTCCAGGGGCCGG
ATGGAACGTCCGCGACACTGCTTGTCTGGCTAAGTCTTATGCCCAGATGTGGCTGCTTCTG
TACTTCCACAGAAGAGACCTGCGGCTCATGGCCAACGCCATTTGCTCCGCTGTCCCTGTGA
ATTGGGTCCCTACCGGAAGAACCACGTGGTCCATCCATGCAGGAGGAGAGTGGATGACAA
CAGAGGACATGTTGGAGGTCTGGAACCGTGTTTGGATAGAGGAGAATGAATGGATGGAAGA
CAAAACCCCAGTGGAGAAATGGAGTGACGTCCCATATTCAGGAAAACGAGAGGACATCTGG
TGTGGCAGCCTGATTGGCACAAGAGCCCGAGCCACGTGGGCAGAAAACATCCAGGTGGCT
ATCAACCAAGTCAGAGCAATCATCGGAGATGAGAAGTATGTGGACTACATGAGTTCACTAAA
GAGATATGAAGACACAACTTTGGTTGAGGACACAGTACTGTAGATATTTAATCAATTGTAAATA
GACAATATAAGTATGCATAAAAGTGTAGTTTTATAGTAGTATTTAGTGGTGTTAGTGTAAATAGT
TAAGAAAATTTTGAGGAGAAAGTCAGGCCGGGAAGTTCCCGCCACCGGAAGTTGAGTAGA
CGGTGCTGCCTGCGACTCAACCCCAGGAGGACTGGGTGAACAAAGCCGCGAAGTGATCC
ATGTAAGCCCTCAGAACCGTCTCGGAAGGAGGACCCCACATGTTGTAACTTCAAAGCCCAA
TGTCAGACCACGCTACGGCGTGCTACTCTGCGGAGAGTGCAGTCTGCGATAGTGCCCCAG
GAGGACTGGGTTAACAAAGGCAAACCAACGCCCCACGCGGCCCTAGCCCCGGTAATGGTG
TTAACCAGGGCGAAAGGACTAGAGGTTAGAGGAGACCCCGCGGTTTAAAGTGCACGGCCC
AGCCTGGCTGAAGCTGTAGGTCAGGGGAAGGACTAGAGGTTAGTGGAGACCCCGTGCCAC
AAAACACCACAACAAAACAGCATATTGACACCTGGGATAGACTAGGAGATCTTCTGCTCTGC
ACAACCAGCCACACGGCACAGTGCGCCGACAATGGTGGCTGGTGGTGCGAGAACACAGG
ATCT

File S1 WNV reference sequences

> E/NS1/NS5+CG

E sequences are in green.
NS1 sequences are in red.
NS5 sequences are in blue.

AGTAGTTCGCCTGTGTGAGCTGACAAACTTAGTAGTGTTTGTGAGGATTAACAACAATTAAC
ACAGTGCGAGCTGTTTCTTAGCACGAAGATCTCGATGTCTAAGAAACCAGGAGGGCCCGGC
AAGAGCCGGGCTGTCAATATGCTAAAACGCGGAATGCCCCGCGTGTTGTCCTTGATTGGAC
TGAAGAGGGCTATGTTGAGCCTGATCGACGGCAAGGGGCCAATACGATTTGTGTTGGCTCT
CTTGGCGTTCTTCAGGTTCACAGCAATTGCTCCGACCCGAGCAGTGCTGGATCGATGGAGA
GGTGTGAACAAACAAACAGCGATGAAACACCTTCTGAGTTTTAAGAAGGAACTAGGGACCT
TGACCAGTGCTATCAATCGGCGGAGCTCAAAACAAAAGAAAAGAGGAGGAAAGACCGGAAT
TGCAGTCATGATTGGCCTGATCGCCAGCGTAGGAGCAGTTACCCTCTCTAACTTCCAAGGG
AAGGTGATGATGACGGTAAATGCTACTGACGTCACAGATGTCATCACGATTCCAACAGCTGC
TGGAAAGAACCTATGCATTGTCAGAGCAATGGATGTGGGATACATGTGCGATGATACTATCA
CTTATGAATGCCCAGTACTGTCGGCTGGTAATGATCCAGAAGACATCGACTGTTGGTGCACA
AAGTCAGCAGTCTACGTCAGGTATGGAAGATGCACCAAGACACGCCACTCAAGACGCAGTC
GGAGGTCACTGACAGTGCAGACACACGGAGAAAGCACTCTAGCGAACAAGAAGGGGGCTT
GGATGGACAGCACCAAGGCCACAAGGTATTTGGTAAAAACAGAATCATGGATCTTGAGGAA
CCCTGGATATGCCCTGGTGGCAGCCGTCATTGGTTGGATGCTTGGGAGCAACACCATGCA
GAGAGTTGTGTTTGTCGTGCTATTGCTTTTGGTGGCCCCAGCTTACAGC**TTCAACTGCCTT**
GGAATGAGCAACAGAGACTTTTTGGAAGGCGTGTCCGGAGCAACGTGGGTCGATTTGGT
TCTCGAAGGCGACAGTTGTGTAACGATAATGTCGAAAGACAAACCAACGATCGACGTCAA
GATGATGAACATGGAAGCGGCGAACCTCGCCGAAGTACGAAGTTATTGTTATTTGGCGAC
CGTAAGTGATCTCTCAACCAAAGCGGCGTGTCCGACAATGGGCGAAGCGCACAACGACA
AACGTGCGGATCCCGCATTTGTGTGCCGACAAGGCGTCGTCGACCGCGGATGGGGAAAC
GGATGTGGACTGTTCGGAAAAGGAAGCATCGACACGTGCGCAAAATTTGCATGCTCGAC
GAAAGCGATCGGACGAACGATTTTGAAAGAAAACATTAAATACGAAGTTGCGATTTTTGTT
CACGGACCGACAACAGTAGAATCTCACGGAAATTATTCGACGCAAGTCGGCGCGACACA
AGCCGGTCGACTGAGCATCACGCCCGCGGCGCCGTCGTATACGCTAAAACTCGGCGAAT
ACGGCGAAGTGACGGTTGATTGCGAACCTCGTTCCGGCATCGACACGAACGCGTACTAC
GTTATGACGGTCGGAACGAAGACTTTTTTAGTTCATCGCGAATGGTTCATGGATCTTAATCT
ACCTTGGAGCAGCGCGGGAAGCACGGTTTGGCGTAATCGCGAAACGTTGATGGAATTTG
AAGAACCTCACGCGACAAAACAGTCCGTTATCGCGTTGGGTTCACAAGAAGGCGCGCTA
CATCAAGCGTTGGCCGGCGCGATTCCTGTCGAATTTTCGAGCAATACAGTTAAGTTGACG
TCCGGTCATTTGAAATGTCGCGTGAAAATGGAAAAATTGCAATTGAAAGGAACGACATAC
GGTGTGTGTTCGAAAGCGTTCAAATTTCTCGGAACGCCCGCGGATACCGGTCACGGAAC
TGTTGTTTTAGAATTGCAATACACTGGAACGGACGGACCTTGTAAAGTTCCGATCTCATCG
GTCGCGTCATTGAACGATCTGACACCGGTCGGACGATTAGTGACTGTGAACCCGTTTGTT
TCGGTCGCGACGGCAAATGCAAAAGTTCTGATCGAATTGGAACCGCCGTTTGGCGACTC
GTACATCGTTGTTGGACGCGGCGAACAACAAATTAATCATCATTGGCACAAATCGGGAAG
CAGCATCGGAAAAGCGTTCACGACGACGCTCAAAGGCGCTCAGCGACTTGCCGCGCTC
GGTGATACGGCGTGGGATTTTGGATCCGTCGGCGGGGTTTTCACGTCGGTAGGGAAGGC
TGTTCATCAAGTATTCGGCGGCGCGTTTCGTTCGCTTTTTGGCGGAATGTCGTGGATTACT
CAAGGATTACTCGGCGCGCTTCTGTTGTGGATGGGAATTAACGCACGAGATCGATCGATC
GCGCTCACATTTCTCGCTGTCGGAGGAGTTCTGCTCTTCCTCTCCGTGAACGTGCACGCT
GACACTGGGTGTGCCATAGACATCAGCCGGCAAGAGCTACGATGCGGAAGTGGCGTGTT
TATTCACAACGACGTCGAAGCGTGGATGGATCGGTACAAGTATTATCCGGAAACGCCGCA

File S1 WNV reference sequences

AGGTCTTGCGAAAATTATACAGAAAGCGCACAAAGAAGGCGTTTGCGGTCTACGATCGGT
TTCTCGACTCGAACATCAAATGTGGGAAGCAGTGAAGGACGAGCTAAACACGCTTTTAAA
AGAAAACGGTGTCGATCTCAGCGTCGTCGTTGAAAAACAAGAGGGAATGTACAAGTCGG
CGCCGAAACGACTCACCGCAACGACGGAAAAATTGGAAATTGGATGGAAGGCGTGGGG
AAAAAGCATTTTATTCGCGCCTGAACTCGCGAACAATACGTTCGTCGTCGACGGTCCTGA
AACGAAAGAATGTCCAACGCAAAATCGCGCGTGGAACAGTTTAGAAGTCGAGGATTTCG
GATTCGGTCTCACGAGCACGCGGATGTTTCTTAAGGTCCGAGAGAGCAACACGACGGAA
TGCGACTCGAAAATAATTGGAACGGCGGTTAAAAACAATTTGGCGATACACAGCGATCTT
TCTTATTGGATCGAAAGTCGGCTGAACGATACTTGGAAACTCGAACGCGCCGTTCTCGGC
GAAGTAAAATCTTGCACGTGGCCGGAGACGCATACGTTGTGGGGTGACGGAATTCTCGA
AAGCGATTTGATAATTCCCGTGACCCTCGCGGGACCGCGAAGCAATCACAATCGTCGACC
GGGGTATAAAACGCAAAATCAGGGTCCATGGGACGAAGGACGCGTCGAGATTGATTTCG
ATTATTGTCCCGGAACAACGGTGACACTGAGTGAGAGTTGTGGACATCGCGGACCCGCG
ACGCGAACGACGACGGAGAGTGGAAAGTTGATTACGGATTGGTGCTGTCGTAGTTGCAC
CTTGCCTCCGCTTCGTTACCAAACCGACAGCGGATGTTGGTACGGGATGGAAATACGACC
GCAACGACACGATGAAAAGACCCTCGTGCAGTCACAAGTGAATGCTTATAATGCTGATATG
ATTGACCCTTTTCAGTTGGGCCTTCTGGTCGTGTTCTTGGCCACCCAGGAGGTCCTTCGCA
AGAGGTGGACAGCCAAGATCAGCATGCCAGCTATACTGATTGCTCTGCTAGTCCTGGTGTTT
GGGGGCATTACTTACACTGATGTGTTACGCTATGTCATCTTGGTGGGGGCAGCTTTCGCAG
AATCTAATTCGGGAGGAGACGTGGTACACTTGGCGCTCATGGCGACCTTCAAGATACAACC
AGTGTTTATGGTGGCATCGTTTCTCAAAGCGAGATGGACCAACCAGGAGAACATTTTGTTGA
TGTTGGCGGCTGTTTTCTTTCAAATGGCTTATCACGATGCCCGCCAAATTCTGCTCTGGGAG
ATCCCTGATGTGTTGAATTCACTGGCGGTAGCTTGGATGATACTGAGAGCCATAACATTCAC
AACGACATCAAACGTGGTTGTTCCGCTGCTAGCCCTGCTAACACCCGGGCTGAGATGCTTG
AATCTGGATGTGTACAGGATACTGCTGTTGATGGTCGGAATAGGCAGCTTGATCAGGGAGA
AGAGGAGTGCAGCCGCAAAAAAGAAAGGAGCAAGTCTGCTATGCTTGGCTCTAGCCTCAA
CAGGACTTTTCAACCCCATGATCCTTGCTGCTGGACTGATTGCATGTGATCCCAACCGTAAA
CGCGGATGGCCCGCAACTGAAGTGATGACAGCTGTCGGCCTAATGTTTGCCATCGTCGGA
GGGCTGGCAGAGCTTGACATTGACTCCATGGCCATTCCAATGACTATCGCGGGGCTCATGT
TTGCTGCTTTCGTGATTTCTGGGAAATCAACAGATATGTGGATTGAGAGAACGGCGGACATT
TCCTGGGAAAGTGATGCAGAAATTACAGGCTCGAGCGAAAGAGTTGATGTGCGGCTTGATG
ATGATGGAAACTTCCAGCTCATGAATGATCCAGGAGCACCTTGGAAGATATGGATGCTCAGA
ATGGTCTGTCTCGCGATTAGTGCGTACACCCCCTGGGCAATCTTGCCCTCAGTAGTTGGATT
TTGGATAACTCTCCAATACACAAAGAGAGGAGGCGTGTTGTGGGACACTCCCTCACCAAAG
GAGTACAAAAAGGGGGACACGACCACCGGCGTCTACAGGATCATGACTCGTGGGCTGCTC
GGCAGTTATCAAGCAGGAGCGGGCGTGATGGTTGAAGGTGTTTTCCACACCCTTTGGCATA
CAACAAAAGGAGCCGCTTTGATGAGCGGAGAGGGCCGCCTGGACCCATACTGGGGCAGT
GTCAAGGAGGATCGACTTTGTTACGGAGGACCCTGGAAATTGCAGCACAAGTGGAACGGG
CAGGATGAGGTGCAGATGATTGTGGTGGAACCTGGCAAGAACGTTAAGAACGTCCAGACG
AAACCAGGGGTGTTCAAAACACCTGAAGGAGAAATCGGGGCCGTGACTTTGGACTTCCCC
ACTGGAACATCAGGCTCACCAATAGTGGACAAAAACGGTGATGTGATTGGGCTTTATGGCAA
TGGAGTCATAATGCCCAACGGCTCATACATAAGCGCGATAGTGCAGGGTGAAAGGATGGAT
GAGCCAATCCCAGCCGGATTCGAACCTGAGATGCTGAGGAAAAAACAGATCACTGTACTGG
ATCTCCATCCCGGCGCCGGTAAAACAAGGAGGATTCTGCCACAGATCATCAAAGAGGCCAT
AAACAGAAGACTGAGAACAGCCGTGCTAGCGCCAACCAGGGTTGTGGCTGCTGAGATGGC
TGAAGCACTGAGAGGACTGCCCATCCGGTACCAGACATCCGCAGTGCCCAGAGAACATAAT
GGAAATGAGATTGTTGATGTCATGTGTCATGCTACCCTCACCCACAGGCTGATGTCTCCTCA

File S1 WNV reference sequences

CAGGGTGCCGAACTACAACCTGTTCGTGATGGATGAGGCTCATTTCACCGACCCAGCTAGC
ATTGCAGCAAGAGGTTACATTTCCACAAAGGTCGAGCTAGGGGAGGCGGCGGCAATATTCA
TGACAGCCACCCCACCAGGCACTTCAGATCCATTCCCAGAGTCCAATTCACCAATTTCCGA
CTTACAGACTGAGATCCCGGATCGAGCTTGGAACTCTGGATACGAATGGATCACAGAATACA
CCGGGAAGACGGTTTGGTTTGTGCCTAGTGTCAAGATGGGGAATGAGATTGCCCTTTGCCT
ACAACGTGCTGGAAAGAAAGTAGTCCAATTGAACAGAAAGTCGTACGAGACGGAGTACCCA
AAATGTAAGAACGATGATTGGGACTTTGTTATCACAACAGACATATCTGAAATGGGGGCTAAC
TTCAAGGCGAGCAGGGTGATTGACAGCCGGAAGAGTGTGAAACCAACCATCATAACAGAAG
GAGAAGGGAGAGTGATCCTGGGAGAACCATCTGCAGTGACAGCAGCTAGTGCCGCCCAGA
GACGTGGACGTATCGGTAGAAATCCGTCGCAAGTTGGTGATGAGTACTGTTATGGGGGGCA
CACGAATGAAGACGACTCGAACTTCGCCCATTGGACTGAGGCACGAATCATGCTGGACAAC
ATCAACATGCCAAACGGACTGATCGCTCAATTCTACCAACCAGAGCGTGAGAAGGTATATAC
CATGGATGGGGAATACCGGCTCAGAGGAGAAGAGAGAAAAAACTTTCTGGAACTGTTGAGG
ACTGCAGATCTGCCAGTTTGGCTGGCTTACAAGGTTGCAGCGGCTGGAGTGTCATACCACG
ACCGGAGGTGGTGCTTTGATGGTCCTAGGACAAACACAATTTTAGAAGACAACAACGAAGT
GGAAGTCATCACGAAGCTTGGTGAAAGGAAGATTCTGAGGCCGCGCTGGATTGATGCCAG
GGTGTACTCGGATCACCAGGCACTAAAGGCGTTCAAGGACTTCGCCTCGGGAAAACGTTCT
CAGATAGGGCTCATTGAGGTTCTGGGAAAGATGCCTGAGCACTTCATGGGGAAGACATGGG
AAGCACTTGACACCATGTACGTTGTGGCCACTGCAGAGAAAGGAGGAAGAGCTCACAGAAT
GGCCCTGGAGGAACTGCCAGATGCTCTTCAGACAATTGCCTTGATTGCCTTATTGAGTGTG
ATGACCATGGGAGTATTCTTCCTCCTCATGCAGCGGAAGGGCATTGGAAAGATAGGTTTGG
GAGGCGCTGTCTTGGGAGTCGCGACCTTTTTCTGTTGGATGGCTGAAGTTCCAGGAACGA
AGATCGCCGGAATGTTGCTGCTCTCCCTTCTCTTGATGATTGTGCTAATTCCTGAGCCAGAG
AAGCAACGTTCGCAGACAGACAACCAGCTAGCCGTGTTCCTGATTTGTGTCATGACCCTTG
TGAGCGCAGTGGCAGCCAACGAGATGGGTTGGCTAGATAAGACCAAGAGTGACATAAGCA
GTTTGTTTGGGCAAAGAATTGAGGTCAAGGAGAATTTCAGCATGGGAGAGTTTCTTCTGGA
CTTGAGGCCGGCAACAGCCTGGTCACTGTACGCTGTGACAACAGCGGTCCTCACTCCACT
GCTAAAGCATTTGATCACGTCAGATTACATCAACACCTCATTGACCTCAATAAACGTTCAGGC
AAGTGCACTATTCACACTCGCGCGAGGCTTCCCCTTCGTCGATGTTGGAGTGTCGGCTCTC
CTGCTAGCAGCCGGATGCTGGGGACAAGTCACCCTCACCGTTACGGTAACAGCGGCAACA
CTCCTTTTTTGCCACTATGCCTACATGGTTCCCGGTTGGCAAGCTGAGGCAATGCGCTCAG
CCCAGCGGCGGACAGCGGCCGGAATCATGAAGAACGCTGTAGTGGATGGCATCGTGGCCA
CGGACGTCCCAGAATTAGAGCGCACCACACCCATCATGCAGAAGAAAGTTGGACAGATCAT
GCTGATCTTGGTGTCTCTAGCTGCAGTAGTAGTGAACCCGTCTGTGAAGACAGTACGAGAA
GCCGGAATTTTGATCACGGCCGCAGCGGTGACGCTTTGGGAGAATGGAGCAAGCTCTGTT
TGGAACGCAACAACTGCCATCGGACTCTGCCACATCATGCGTGGGGGTTGGTTGTCATGTC
TATCCATAACATGGACACTCATAAAGAACATGGAAAAACCAGGACTAAAAAGA**GGTGGGGCA**
AAAGGACGCACCTTGGGAGAGGTTTGGAAAGAAAGACTCAACCAGATGACAAAAGAAG
AGTTCACTAGGTACCGCAAAGAAGCGATCATAGAAGTCGATCGATCGGCAGCGAAACAC
GCGCGAAAAGAAGGAAACGTAACGGGCGGTCATCCCGTTTCACGCGGGACGGCGAAAC
TTCGATGGCTTGTAGAACGGCGGTTTCTCGAACCGGTCGGAAAAGTGATAGATCTAGGAT
GCGGACGCGGAGGTTGGTGTTATTACATGGCGACACAAAAACGCGTTCAAGAAGTCCGT
GGGTACACGAAAGGAGGTCCGGGACACGAAGAACCGCAACTCGTTCAAAGTTACGGAT
GGAACATCGTCACCATGAAGAGCGGCGTCGACGTTTTTTATCGACCTTCGGAATGTTGCG
ACACACTTCTATGTGACATTGGCGAGTCTTCATCAAGTGCGGAAGTCGAAGAACATCGCA
CAATTCGCGTTCTAGAAATGGTCGAAGACTGGCTACATCGCGGACCGCGCGAATTTTGTG
TGAAAGTGCTCTGTCCGTACATGCCGAAAGTTATCGAAAAGATGGAACTGCTTCAACGTC

File S1 WNV reference sequences

**GTTATGGCGGAGGACTCGTGCGAAATCCGCTGTCTCGGAATTCGACGCACGAAATGTATT
GGGTGAGTCGAGCGTCGGGTAATGTCGTCCATTCGGTAAACATGACGAGCCAAGTTCTCC
TCGGACGAATGGAAAAACGAACGTGGAAGGGACCGCAATACGAAGAAGATGTCAATTTG
GGAAGCGGAACCCGAGCGGTCGGAAAACCGCTTCTGAATTCCGACACGAGCAAAATCA
AAAACCGAATCGAACGACTCCGACGCGAATACAGTTCGACATGGCATCACGATGAGAATC
ATCCTTATCGAACCTGGAATTATCACGGGAGTTACGACGTGAAGCCGACGGGATCGGCCA
GTTCGCTCGTGAACGGAGTGGTTCGACTTCTGTCAAAACCGTGGGACACGATAACGAAC
GTAACGACAATGGCCATGACGGACACGACTCCGTTCGGTCAGCAACGCGTTTTCAAAGA
GAAAGTTGACACGAAAGCGCCGGAACCGCCGGAAGGCGTGAAGTACGTGCTTAACGAA
ACGACCAATTGGTTGTGGGCGTTTTTGGCACGCGAAAAACGACCTCGAATGTGTTCGCGA
GAAGAATTCATTCGAAAAGTCAACAGCAACGCGGCATTGGGCGCGATGTTCGAAGAGCA
AAATCAATGGCGGAGCGCGCGAGAAGCGGTTGAAGATCCCAAATTTTGGGAAATGGTGG
ACGAGGAGCGAGAAGCGCATCTGCGCGGCGAATGTCACACGTGCATTTACAACATGATG
GGAAAGCGCGAGAAAAAACCAGGCGAATTTGGAAAAGCAAAAGGAAGCCGCGCGATTT
GGTTCATGTGGCTCGGCGCTCGATTTCTCGAATTTGAGGCGCTCGGTTTTCTCAACGAAG
ATCATTGGCTAGGACGAAAAAATTCCGGAGGCGGCGTCGAAGGATTGGGTCTGCAAAAA
CTCGGTTACATTCTTCGTGAAGTTGGAACGCGGCCAGGCGGAAAGATCTACGCGGACGA
CACGGCAGGATGGGACACGCGAATTACGCGAGCGGATTTGGAAAACGAAGCGAAAGTA
CTCGAGCTGCTTGATGGCGAACATCGTCGTCTCGCGCGAGCCATCATCGAGCTGACGTAT
CGTCACAAAGTCGTCAAAGTGATGCGGCCGGCGGCGGACGGACGAACGGTGATGGACG
TCATCTCGCGAGAAGATCAGCGTGGGAGTGGACAAGTCGTTACATACGCGCTCAACACG
TTCACCAATCTCGCCGTGCAACTAGTTCGAATGATGGAAGGAGAAGGCGTGATCGGACC
GGATGACGTCGAAAAACTTACAAAAGGCAAAGGACCGAAAGTACGGACGTGGCTGTTCG
AAAATGGAGAAGAACGACTGAGTCGAATGGCGGTTAGTGGCGACGATTGCGTCGTTAAA
CCGCTCGACGATCGATTCGCGACCTCGCTTCATTTCCTTAACGCGATGTCGAAGGTTCGA
AAAGACATTCAAGAATGGAAACCCTCGACGGGATGGTATGATTGGCAACAAGTTCCGTTT
TGTTCGAATCATTTCACCGAATTGATAATGAAAGACGGACGAACGCTTGTTGTTCCTTGTC
GAGGACAAGATGAATTGGTCGGGCGCGCACGAATTTCGCCGGGCGCAGGATGGAATGTT
CGCGACACGGCGTGTCTCGCAAAATCGTACGCGCAGATGTGGCTGCTTCTGTATTTTCAT
CGACGCGATCTTCGACTCATGGCGAACGCGATTTGTTCGGCAGTTCCGGTGAATTGGGTT
CCAACGGGACGAACAACGTGGTCAATCCACGCGGGCGGTGAGTGGATGACAACGGAAG
ACATGTTGGAAGTCTGGAACCGCGTTTGGATAGAAGAGAATGAATGGATGGAAGACAAA
ACGCCGGTCGAGAAATGGAGCGATGTTCCGTATTCGGGAAAACGCGAGGACATTTGGTG
CGGGAGTCTGATCGGAACGCGAGCGCGCGCCACGTGGGCGGAAAACATTCAAGTCGCA
ATCAATCAAGTCCGTGCGATCATCGGAGATGAGAAGTATGTGGACTACATGAGTTCACTAA
AGAGATATGAAGACACAACTTTGGTTGAGGACACAGTACTG**TAGATATTTAATCAATTGTAA
ATAGACAATATAAGTATGCATAAAAGTGTAGTTTTATAGTAGTATTTAGTGGTGTTAGTGTAAAT
AGTTAAGAAAATTTTGAGGAGAAAGTCAGGCCGGGAAGTTCCCGCCACCGGAAGTTGAGTA
GACGGTGCTGCCTGCGACTCAACCCCAGGAGGACTGGGTGAACAAAGCCGCGAAGTGAT
CCATGTAAGCCCTCAGAACCGTCTCGGAAGGAGGACCCCACATGTTGTAACTTCAAAGCCC
AATGTCAGACCACGCTACGGCGTGCTACTCTGCGGAGAGTGCAGTCTGCGATAGTGCCCC
AGGAGGACTGGGTTAACAAAGGCAAACCAACGCCCCACGCGGCCCTAGCCCCGGTAATGG
TGTTAACCAGGGCGAAAGGACTAGAGGTTAGAGGAGACCCCGCGGTTTAAAGTGCACGGC
CCAGCCTGGCTGAAGCTGTAGGTCAGGGGAAGGACTAGAGGTTAGTGGAGACCCCGTGC
CACAAAACACCACAACAAAACAGCATATTGACACCTGGGATAGACTAGGAGATCTTCTGCTC
TGCACAACCAGCCACACGGCACAGTGCGCCGACAATGGTGGCTGGTGGTGCGAGAACAC
AGGATCT

File S1 WNV reference sequences

> E-MAX

E sequences are in green.

AGTAGTTCGCCTGTGTGAGCTGACAAACTTAGTAGTGTTTGTGAGGATTAACAACAATTAAC
ACAGTGCGAGCTGTTTCTTAGCACGAAGATCTCGATGTCTAAGAAACCAGGAGGGCCCGGC
AAGAGCCGGGCTGTCAATATGCTAAAACGCGGAATGCCCCGCGTGTTGTCCTTGATTGGAC
TGAAGAGGGCTATGTTGAGCCTGATCGACGGCAAGGGGCCAATACGATTTGTGTTGGCTCT
CTTGGCGTTCTTCAGGTTCACAGCAATTGCTCCGACCCGAGCAGTGCTGGATCGATGGAGA
GGTGTGAACAAACAAACAGCGATGAAACACCTTCTGAGTTTTAAGAAGGAACTAGGGACCT
TGACCAGTGCTATCAATCGGCGGAGCTCAAAACAAAAGAAAAGAGGAGGAAAGACCGGAAT
TGCAGTCATGATTGGCCTGATCGCCAGCGTAGGAGCAGTTACCCTCTCTAACTTCCAAGGG
AAGGTGATGATGACGGTAAATGCTACTGACGTCACAGATGTCATCACGATTCCAACAGCTGC
TGGAAAGAACCTATGCATTGTCAGAGCAATGGATGTGGGATACATGTGCGATGATACTATCA
CTTATGAATGCCCAGTACTGTCGGCTGGTAATGATCCAGAAGACATCGACTGTTGGTGCACA
AAGTCAGCAGTCTACGTCAGGTATGGAAGATGCACCAAGACACGCCACTCAAGACGCAGTC
GGAGGTCACTGACAGTGCAGACACACGGAGAAAGCACTCTAGCGAACAAGAAGGGGGCTT
GGATGGACAGCACCAAGGCCACAAGGTATTTGGTAAAAACAGAATCATGGATCTTGAGGAA
CCCTGGATATGCCCTGGTGGCAGCCGTCATTGGTTGGATGCTTGGGAGCAACACCATGCA
GAGAGTTGTGTTTGTCGTGCTATTGCTTTTGGTGGCCCCAGCTTACAGC**TTCAACTGCCTT**
GGAATGAGCAACAGAGACTTTTTGGAAGGCGTTTCCGGCGCGACGTGGGTCGATTTAGTT
CTCGAAGGGGATAGTTGCGTTACGATAATGTCGAAAGACAAACCGACGATCGACGTTAAA
ATGATGAATATGGAGGCCGCGAATCTAGCGGAAGTTCGCAGTTATTGTTATTTAGCAACCG
TAAGCGATCTTTCGACGAAAGCCGCGTGTCCGACGATGGGCGAAGCGCACAACGATAAA
CGGGCGGATCCCGCGTTTGTTTGTCGACAAGGCGTCGTCGATCGCGGTTGGGGAAATGG
TTGTGGACTATTCGGCAAAGGAAGTATCGATACGTGCGCGAAATTTGCGTGTTCTACTAAA
GCGATAGGACGAACGATATTAAAAGAAAATATCAAATACGAAGTCGCGATTTTTGTCCACG
GACCGACGACGGTCGAATCGCACGGAAATTATTCGACGCAAGTCGGCGCGACGCAGGC
CGGTCGACTAAGTATAACGCCCGCGGCGCCGTCGTACACGCTAAAACTGGGCGAATATG
GCGAGGTTACGGTCGACTGCGAACCGCGTTCGGGTATCGATACGAACGCGTATTATGTTAT
GACGGTCGGAACGAAAACGTTTTTAGTACATCGGGAATGGTTCATGGATCTAAACCTACC
GTGGAGCAGTGCCGGAAGTACCGTTTGGCGTAACCGGGAAACGTTAATGGAATTCGAAG
AACCGCACGCGACTAAACAGTCCGTAATCGCGTTAGGATCGCAAGAAGGCGCGCTTCAT
CAAGCGTTAGCCGGCGCGATTCCGGTCGAATTTTCGAGTAATACCGTCAAATTAACATCCG
GTCATTTGAAATGTCGGGTCAAAATGGAAAAATTACAATTAAAAGGAACGACTTACGGCG
TATGTTCGAAAGCGTTCAAGTTTCTCGGTACACCCGCCGATACCGGTCACGGAACCGTCG
TTTTGGAATTACAATATACCGGAACCGATGGACCGTGTAAAGTTCCGATATCGTCCGTGGC
GTCGTTGAACGATCTAACACCCGTCGGCCGATTAGTCACCGTTAATCCGTTCGTTTCCGTC
GCGACGGCGAACGCGAAAGTACTTATAGAATTGGAACCGCCGTTTGGCGATTCGTATATC
GTCGTCGGCCGCGGGGAACAACAAATAAATCACCATTGGCATAAGTCCGGAAGTAGTATC
GGAAAAGCGTTTACGACGACGCTAAAAGGCGCGCAACGACTAGCCGCGCTCGGCGATAC
CGCGTGGGATTTCGGATCCGTCGGGGGCGTTTTTACGTCCGTCGGTAAAGCCGTACATCA
AGTATTCGGCGGGGCGTTTCGCTCGCTTTTCGGCGGAATGTCGTGGATAACGCAAGGATT
ACTGGGCGCGCTACTTTTATGGATGGGAATAAACGCGCGGGATCGTTCGATCGCGCTAAC
GTTTCTCGCCGTCGGCGGAGTTCTGCTCTTCCTCTCCGTGAACGTGCACGCTGACACTGG
GTGTGCCATAGACATCAGCCGGCAAGAGCTGAGATGTGGAAGTGGAGTGTTCATACACAAT
GATGTGGAGGCTTGGATGGACCGGTACAAGTATTACCCTGAAACGCCACAAGGCCTAGCCA
AGATCATTCAGAAAGCTCATAAGGAAGGAGTGTGCGGTCTACGATCAGTTTCCAGACTGGA

File S1 WNV reference sequences

GCATCAAATGTGGGAAGCAGTGAAGGACGAGCTGAACACTCTTTTGAAGGAGAATGGTGTG
GACCTTAGTGTCGTGGTTGAGAAACAGGAGGGAATGTACAAGTCAGCACCTAAACGCCTCA
CCGCCACCACGGAAAAATTGGAAATTGGCTGGAAGGCCTGGGGAAAGAGTATTTTATTTGC
ACCAGAACTCGCCAACAACACCTTTGTGGTTGATGGTCCGGAGACCAAGGAATGTCCGACT
CAGAATCGCGCTTGGAATAGCTTAGAAGTGGAGGATTTTGGATTTGGTCTCACCAGCACTC
GGATGTTCCTGAAGGTCAGAGAGAGCAACACAACTGAATGTGACTCGAAGATCATTGGAAC
GGCTGTCAAGAACAACTTGGCGATCCACAGTGACCTGTCCTATTGGATTGAAAGCAGGCTC
AATGATACGTGGAAGCTTGAAAGGGCAGTTCTGGGTGAAGTCAAATCATGTACGTGGCCTG
AGACGCATACCTTGTGGGGCGATGGAATCCTTGAGAGTGACTTGATAATACCAGTCACACTG
GCGGGACCACGAAGCAATCACAATCGGAGACCTGGGTACAAGACACAAAACCAGGGCCCA
TGGGACGAAGGCCGGGTAGAGATTGACTTCGATTACTGCCCAGGAACTACGGTCACCCTG
AGTGAGAGCTGCGGACACCGTGGACCTGCCACTCGCACCACCACAGAGAGCGGAAAGTT
GATAACAGATTGGTGCTGCAGGAGCTGCACCTTACCACCACTGCGCTACCAAACTGACAGC
GGCTGTTGGTATGGTATGGAGATCAGACCACAGAGACATGATGAAAAGACCCTCGTGCAGT
CACAAGTGAATGCTTATAATGCTGATATGATTGACCCTTTTCAGTTGGGCCTTCTGGTCGTGT
TCTTGGCCACCCAGGAGGTCCTTCGCAAGAGGTGGACAGCCAAGATCAGCATGCCAGCTA
TACTGATTGCTCTGCTAGTCCTGGTGTTTGGGGGCATTACTTACACTGATGTGTTACGCTATG
TCATCTTGGTGGGGGCAGCTTTCGCAGAATCTAATTCGGGAGGAGACGTGGTACACTTGGC
GCTCATGGCGACCTTCAAGATACAACCAGTGTTTATGGTGGCATCGTTTCTCAAAGCGAGAT
GGACCAACCAGGAGAACATTTTGTTGATGTTGGCGGCTGTTTTCTTTCAAATGGCTTATCAC
GATGCCCGCCAAATTCTGCTCTGGGAGATCCCTGATGTGTTGAATTCACTGGCGGTAGCTT
GGATGATACTGAGAGCCATAACATTCACAACGACATCAAACGTGGTTGTTCCGCTGCTAGCC
CTGCTAACACCCGGGCTGAGATGCTTGAATCTGGATGTGTACAGGATACTGCTGTTGATGGT
CGGAATAGGCAGCTTGATCAGGGAGAAGAGGAGTGCAGCCGCAAAAAAGAAAGGAGCAAG
TCTGCTATGCTTGGCTCTAGCCTCAACAGGACTTTTCAACCCCATGATCCTTGCTGCTGGAC
TGATTGCATGTGATCCCAACCGTAAACGCGGATGGCCCGCAACTGAAGTGATGACAGCTGT
CGGCCTAATGTTTGCCATCGTCGGAGGGCTGGCAGAGCTTGACATTGACTCCATGGCCATT
CCAATGACTATCGCGGGGCTCATGTTTGCTGCTTTCGTGATTTCTGGGAAATCAACAGATAT
GTGGATTGAGAGAACGGCGGACATTTCCTGGGAAAGTGATGCAGAAATTACAGGCTCGAGC
GAAAGAGTTGATGTGCGGCTTGATGATGATGGAAACTTCCAGCTCATGAATGATCCAGGAG
CACCTTGGAAGATATGGATGCTCAGAATGGTCTGTCTCGCGATTAGTGCGTACACCCCCTG
GGCAATCTTGCCCTCAGTAGTTGGATTTTGGATAACTCTCCAATACACAAAGAGAGGAGGCG
TGTTGTGGGACACTCCCTCACCAAAGGAGTACAAAAAGGGGGACACGACCACCGGCGTCT
ACAGGATCATGACTCGTGGGCTGCTCGGCAGTTATCAAGCAGGAGCGGGCGTGATGGTTG
AAGGTGTTTTCCACACCCTTTGGCATACAACAAAAGGAGCCGCTTTGATGAGCGGAGAGGG
CCGCCTGGACCCATACTGGGGCAGTGTCAAGGAGGATCGACTTTGTTACGGAGGACCCTG
GAAATTGCAGCACAAGTGGAACGGGCAGGATGAGGTGCAGATGATTGTGGTGGAACCTGG
CAAGAACGTTAAGAACGTCCAGACGAAACCAGGGGTGTTCAAAACACCTGAAGGAGAAATC
GGGGCCGTGACTTTGGACTTCCCCACTGGAACATCAGGCTCACCAATAGTGGACAAAAAC
GGTGATGTGATTGGGCTTTATGGCAATGGAGTCATAATGCCCAACGGCTCATACATAAGCGC
GATAGTGCAGGGTGAAAGGATGGATGAGCCAATCCCAGCCGGATTCGAACCTGAGATGCTG
AGGAAAAAACAGATCACTGTACTGGATCTCCATCCCGGCGCCGGTAAAACAAGGAGGATTC
TGCCACAGATCATCAAAGAGGCCATAAACAGAAGACTGAGAACAGCCGTGCTAGCGCCAAC
CAGGGTTGTGGCTGCTGAGATGGCTGAAGCACTGAGAGGACTGCCCATCCGGTACCAGAC
ATCCGCAGTGCCCAGAGAACATAATGGAAATGAGATTGTTGATGTCATGTGTCATGCTACCC
TCACCCACAGGCTGATGTCTCCTCACAGGGTGCCGAACTACAACCTGTTCGTGATGGATGA
GGCTCATTTCACCGACCCAGCTAGCATTGCAGCAAGAGGTTACATTTCCACAAAGGTCGAG

File S1 WNV reference sequences

CTAGGGGAGGCGGCGGCAATATTCATGACAGCCACCCCACCAGGCACTTCAGATCCATTCC
CAGAGTCCAATTCACCAATTTCCGACTTACAGACTGAGATCCCGGATCGAGCTTGGAACTCT
GGATACGAATGGATCACAGAATACACCGGGAAGACGGTTTGGTTTGTGCCTAGTGTCAAGA
TGGGGAATGAGATTGCCCTTTGCCTACAACGTGCTGGAAAGAAAGTAGTCCAATTGAACAG
AAAGTCGTACGAGACGGAGTACCCAAAATGTAAGAACGATGATTGGGACTTTGTTATCACAA
CAGACATATCTGAAATGGGGGCTAACTTCAAGGCGAGCAGGGTGATTGACAGCCGGAAGA
GTGTGAAACCAACCATCATAACAGAAGGAGAAGGGAGAGTGATCCTGGGAGAACCATCTGC
AGTGACAGCAGCTAGTGCCGCCCAGAGACGTGGACGTATCGGTAGAAATCCGTCGCAAGT
TGGTGATGAGTACTGTTATGGGGGGCACACGAATGAAGACGACTCGAACTTCGCCCATTGG
ACTGAGGCACGAATCATGCTGGACAACATCAACATGCCAAACGGACTGATCGCTCAATTCTA
CCAACCAGAGCGTGAGAAGGTATATACCATGGATGGGGAATACCGGCTCAGAGGAGAAGAG
AGAAAAAACTTTCTGGAACTGTTGAGGACTGCAGATCTGCCAGTTTGGCTGGCTTACAAGG
TTGCAGCGGCTGGAGTGTCATACCACGACCGGAGGTGGTGCTTTGATGGTCCTAGGACAA
ACACAATTTTAGAAGACAACAACGAAGTGGAAGTCATCACGAAGCTTGGTGAAAGGAAGATT
CTGAGGCCGCGCTGGATTGATGCCAGGGTGTACTCGGATCACCAGGCACTAAAGGCGTTC
AAGGACTTCGCCTCGGGAAAACGTTCTCAGATAGGGCTCATTGAGGTTCTGGGAAAGATGC
CTGAGCACTTCATGGGGAAGACATGGGAAGCACTTGACACCATGTACGTTGTGGCCACTGC
AGAGAAAGGAGGAAGAGCTCACAGAATGGCCCTGGAGGAACTGCCAGATGCTCTTCAGAC
AATTGCCTTGATTGCCTTATTGAGTGTGATGACCATGGGAGTATTCTTCCTCCTCATGCAGC
GGAAGGGCATTGGAAAGATAGGTTTGGGAGGCGCTGTCTTGGGAGTCGCGACCTTTTTCT
GTTGGATGGCTGAAGTTCCAGGAACGAAGATCGCCGGAATGTTGCTGCTCTCCCTTCTCTT
GATGATTGTGCTAATTCCTGAGCCAGAGAAGCAACGTTCGCAGACAGACAACCAGCTAGCC
GTGTTCCTGATTTGTGTCATGACCCTTGTGAGCGCAGTGGCAGCCAACGAGATGGGTTGG
CTAGATAAGACCAAGAGTGACATAAGCAGTTTGTTTGGGCAAAGAATTGAGGTCAAGGAGA
ATTTCAGCATGGGAGAGTTTCTTCTGGACTTGAGGCCGGCAACAGCCTGGTCACTGTACGC
TGTGACAACAGCGGTCCTCACTCCACTGCTAAAGCATTTGATCACGTCAGATTACATCAACA
CCTCATTGACCTCAATAAACGTTCAGGCAAGTGCACTATTCACACTCGCGCGAGGCTTCCC
CTTCGTCGATGTTGGAGTGTCGGCTCTCCTGCTAGCAGCCGGATGCTGGGGACAAGTCAC
CCTCACCGTTACGGTAACAGCGGCAACACTCCTTTTTTGCCACTATGCCTACATGGTTCCCG
GTTGGCAAGCTGAGGCAATGCGCTCAGCCCAGCGGCGGACAGCGGCCGGAATCATGAAG
AACGCTGTAGTGGATGGCATCGTGGCCACGGACGTCCCAGAATTAGAGCGCACCACACCC
ATCATGCAGAAGAAAGTTGGACAGATCATGCTGATCTTGGTGTCTCTAGCTGCAGTAGTAGT
GAACCCGTCTGTGAAGACAGTACGAGAAGCCGGAATTTTGATCACGGCCGCAGCGGTGAC
GCTTTGGGAGAATGGAGCAAGCTCTGTTTGGAACGCAACAACTGCCATCGGACTCTGCCA
CATCATGCGTGGGGGTTGGTTGTCATGTCTATCCATAACATGGACACTCATAAAGAACATGG
AAAAACCAGGACTAAAAAGAGGTGGGGCAAAAGGACGCACCTTGGGAGAGGTTTGGAAAG
AAAGACTCAACCAGATGACAAAAGAAGAGTTCACTAGGTACCGCAAAGAGGCCATCATCGA
AGTCGATCGCTCAGCGGCAAAACACGCCAGGAAAGAAGGCAATGTCACTGGAGGGCATCC
AGTCTCTAGGGGCACAGCAAAACTGAGATGGCTGGTCGAACGGAGGTTTCTCGAACCGGT
CGGAAAAGTGATTGACCTTGGATGTGGAAGAGGCGGTTGGTGTTACTATATGGCAACCCAA
AAAAGAGTCCAAGAAGTCAGAGGGTACACAAAGGGCGGTCCCGGACATGAAGAGCCCCAA
CTAGTGCAAAGTTATGGATGGAACATTGTCACCATGAAGAGTGGAGTGGATGTGTTCTACAG
ACCTTCTGAGTGTTGTGACACCCTCCTTTGTGACATCGGAGAGTCCTCGTCAAGTGCTGAG
GTTGAAGAGCATAGGACGATTCGGGTCCTTGAAATGGTTGAGGACTGGCTGCACCGAGGG
CCAAGGGAATTTTGCGTGAAGGTGCTCTGTCCCTACATGCCGAAAGTCATAGAGAAGATGG
AGCTGCTCCAACGCCGGTATGGGGGGGGACTGGTCAGAAACCCACTCTCACGGAATTCCA
CGCACGAGATGTATTGGGTGAGTCGAGCTTCAGGCAATGTGGTACATTCAGTGAATATGACC

File S1 WNV reference sequences

AGCCAGGTGCTCCTAGGAAGAATGGAAAAAAGGACCTGGAAGGGACCCCAATACGAGGAA
GATGTAAACTTGGGAAGTGGAACCAGGGCGGTGGGAAAACCCCTGCTCAACTCAGACACC
AGTAAAATCAAGAACAGGATTGAACGACTCAGGCGTGAGTACAGTTCGACGTGGCACCACG
ATGAGAACCACCCATATAGAACCTGGAACTATCACGGCAGTTATGATGTGAAGCCCACAGGC
TCCGCCAGTTCGCTGGTCAATGGAGTGGTCAGGCTCCTCTCAAAACCATGGGACACCATCA
CGAATGTTACCACCATGGCCATGACTGACACTACTCCCTTCGGGCAGCAGCGAGTGTTCAA
AGAGAAGGTGGACACGAAAGCTCCTGAACCGCCAGAAGGAGTGAAGTACGTGCTCAACGA
GACCACCAACTGGTTGTGGGCGTTTTTGGCCAGAGAAAAACGTCCCAGAATGTGCTCTCGA
GAGGAATTCATAAGAAAGGTCAACAGCAATGCAGCTTTGGGTGCCATGTTTGAAGAGCAGA
ATCAATGGAGGAGCGCCAGAGAAGCAGTTGAAGATCCAAAATTTTGGGAGATGGTGGATGA
GGAGCGCGAGGCACATCTGCGGGGGGAATGTCACACTTGCATTTACAACATGATGGGAAA
GAGAGAGAAAAAACCCGGAGAGTTCGGAAAGGCCAAGGGAAGCAGAGCCATTTGGTTCAT
GTGGCTCGGAGCTCGCTTTCTGGAGTTCGAGGCTCTGGGTTTTCTCAATGAAGACCACTG
GCTTGGAAGAAAGAACTCAGGAGGAGGTGTCGAGGGCTTGGGCCTCCAAAAACTGGGTTA
CATCCTGCGTGAAGTTGGCACCCGGCCTGGGGGCAAGATCTATGCTGATGACACAGCTGG
CTGGGACACCCGCATCACGAGAGCTGACTTGGAAAATGAAGCTAAGGTGCTTGAGCTGCTT
GATGGGGAACATCGGCGTCTTGCCAGGGCCATCATTGAGCTCACCTATCGTCACAAAGTTG
TGAAAGTGATGCGCCCGGCTGCTGATGGAAGAACCGTCATGGATGTTATCTCCAGAGAAGA
TCAGAGGGGGAGTGGACAAGTTGTCACCTACGCCCTAAACACTTTCACCAACCTGGCCGT
CCAGCTGGTGAGGATGATGGAAGGGGAAGGAGTGATTGGCCCAGATGATGTGGAGAAACT
CACAAAAGGGAAAGGACCCAAAGTCAGGACCTGGCTGTTTGAGAATGGGGAAGAAAGACT
CAGCCGCATGGCTGTCAGTGGAGATGACTGTGTGGTAAAGCCCCTGGACGATCGCTTTGC
CACCTCGCTCCACTTCCTCAATGCTATGTCAAAGGTTCGCAAAGACATCCAAGAGTGGAAA
CCGTCAACTGGATGGTATGATTGGCAGCAGGTTCCATTTTGCTCAAACCATTTCACTGAATT
GATCATGAAAGATGGAAGAACACTGGTGGTTCCATGCCGAGGACAGGATGAATTGGTAGGC
AGAGCTCGCATATCTCCAGGGGCCGGATGGAACGTCCGCGACACTGCTTGTCTGGCTAAG
TCTTATGCCCAGATGTGGCTGCTTCTGTACTTCCACAGAAGAGACCTGCGGCTCATGGCCA
ACGCCATTTGCTCCGCTGTCCCTGTGAATTGGGTCCCTACCGGAAGAACCACGTGGTCCAT
CCATGCAGGAGGAGAGTGGATGACAACAGAGGACATGTTGGAGGTCTGGAACCGTGTTTG
GATAGAGGAGAATGAATGGATGGAAGACAAAACCCCAGTGGAGAAATGGAGTGACGTCCCA
TATTCAGGAAAACGAGAGGACATCTGGTGTGGCAGCCTGATTGGCACAAGAGCCCGAGCC
ACGTGGGCAGAAAACATCCAGGTGGCTATCAACCAAGTCAGAGCAATCATCGGAGATGAGA
AGTATGTGGACTACATGAGTTCACTAAAGAGATATGAAGACACAACTTTGGTTGAGGACACA
GTACTGTAGATATTTAATCAATTGTAAATAGACAATATAAGTATGCATAAAAGTGTAGTTTTATAG
TAGTATTTAGTGGTGTTAGTGTAAATAGTTAAGAAAATTTTGAGGAGAAAGTCAGGCCGGGAA
GTTCCCGCCACCGGAAGTTGAGTAGACGGTGCTGCCTGCGACTCAACCCCAGGAGGACTG
GGTGAACAAAGCCGCGAAGTGATCCATGTAAGCCCTCAGAACCGTCTCGGAAGGAGGACC
CCACATGTTGTAACTTCAAAGCCCAATGTCAGACCACGCTACGGCGTGCTACTCTGCGGAG
AGTGCAGTCTGCGATAGTGCCCCAGGAGGACTGGGTTAACAAAGGCAAACCAACGCCCCA
CGCGGCCCTAGCCCCGGTAATGGTGTTAACCAGGGCGAAAGGACTAGAGGTTAGAGGAGA
CCCCGCGGTTTAAAGTGCACGGCCCAGCCTGGCTGAAGCTGTAGGTCAGGGGAAGGACTA
GAGGTTAGTGGAGACCCCGTGCCACAAAACACCACAACAAAACAGCATATTGACACCTGGG
ATAGACTAGGAGATCTTCTGCTCTGCACAACCAGCCACACGGCACAGTGCGCCGACAATGG
TGGCTGGTGGTGCGAGAACACAGGATCT

File S1 WNV reference sequences

> E+UA

E sequences are in green.

AGTAGTTCGCCTGTGTGAGCTGACAAACTTAGTAGTGTTTGTGAGGATTAACAACAATTAAC
ACAGTGCGAGCTGTTTCTTAGCACGAAGATCTCGATGTCTAAGAAACCAGGAGGGCCCGGC
AAGAGCCGGGCTGTCAATATGCTAAAACGCGGAATGCCCCGCGTGTTGTCCTTGATTGGAC
TGAAGAGGGCTATGTTGAGCCTGATCGACGGCAAGGGGCCAATACGATTTGTGTTGGCTCT
CTTGGCGTTCTTCAGGTTCACAGCAATTGCTCCGACCCGAGCAGTGCTGGATCGATGGAGA
GGTGTGAACAAACAAACAGCGATGAAACACCTTCTGAGTTTTAAGAAGGAACTAGGGACCT
TGACCAGTGCTATCAATCGGCGGAGCTCAAAACAAAAGAAAAGAGGAGGAAAGACCGGAAT
TGCAGTCATGATTGGCCTGATCGCCAGCGTAGGAGCAGTTACCCTCTCTAACTTCCAAGGG
AAGGTGATGATGACGGTAAATGCTACTGACGTCACAGATGTCATCACGATTCCAACAGCTGC
TGGAAAGAACCTATGCATTGTCAGAGCAATGGATGTGGGATACATGTGCGATGATACTATCA
CTTATGAATGCCCAGTACTGTCGGCTGGTAATGATCCAGAAGACATCGACTGTTGGTGCACA
AAGTCAGCAGTCTACGTCAGGTATGGAAGATGCACCAAGACACGCCACTCAAGACGCAGTC
GGAGGTCACTGACAGTGCAGACACACGGAGAAAGCACTCTAGCGAACAAGAAGGGGGCTT
GGATGGACAGCACCAAGGCCACAAGGTATTTGGTAAAAACAGAATCATGGATCTTGAGGAA
CCCTGGATATGCCCTGGTGGCAGCCGTCATTGGTTGGATGCTTGGGAGCAACACCATGCA
GAGAGTTGTGTTTGTCGTGCTATTGCTTTTGGTGGCCCCAGCTTACAGC**TTCAACTGCCTT**
GGAATGAGCAACAGAGACTTCTTGGAGGGAGTATCTGGAGCAACGTGGGTAGACTTAGT
GCTAGAAGGCGACAGCTGTGTGACTATCATGTCTAAGGACAAGCCTACCATCGATGTTAA
GATGATGAACATGGAGGCGGCCAACCTGGCAGAGGTCCGTAGCTATTGCTACTTGGCGA
CCGTCAGTGATCTCTCCACCAAAGCCGCGTGCCCGACTATGGGAGAAGCGCACAATGAT
AAACGTGCGGACCCAGCTTTCGTATGCAGACAGGGAGTAGTAGACAGGGGCTGGGGCAA
CGGCTGCGGGCTATTCGGCAAAGGAAGCATAGATACGTGTGCCAAGTTTGCCTGCTCTAC
CAAGGCAATAGGAAGAACCATATTGAAAGAGAACATCAAGTATGAGGTGGCTATTTTTGTA
CATGGGCCTACTACTGTAGAGTCTCACGGCAACTACTCCACCCAGGTAGGAGCTACTCAG
GCAGGTAGACTCAGTATCACTCCTGCGGCTCCCTCATATACCCTAAAGCTTGGGGAATATG
GAGAGGTTACGGTGGACTGTGAGCCCCGGTCAGGGATAGACACTAATGCATACTACGTGA
TGACTGTTGGTACAAAGACGTTCTTGGTCCATAGGGAGTGGTTCATGGACCTCAACCTAC
CCTGGAGCAGTGCGGGAAGTACTGTGTGGAGGAATAGAGAGACTTTAATGGAGTTTGAG
GAGCCACACGCCACTAAGCAGTCTGTTATAGCATTAGGCTCACAAGAGGGCGCCCTGCAT
CAAGCTTTAGCGGGGGCCATTCCTGTGGAATTCTCCAGCAACACTGTCAAGTTGACGTCC
GGTCATTTGAAGTGTAGAGTAAAGATGGAAAAATTGCAGTTGAAGGGCACCACCTATGGA
GTCTGTTCAAAGGCTTTTAAGTTTCTTGGGACTCCCGCGGACACAGGTCACGGCACTGTG
GTGTTAGAATTACAGTACACTGGCACGGATGGACCTTGCAAAGTTCCTATCTCTTCGGTGG
CTTCATTGAATGACCTAACACCGGTAGGTAGATTGGTAACTGTAAACCCCTTTGTCTCCGT
GGCCACGGCCAACGCTAAGGTACTCATTGAATTGGAGCCACCCTTCGGGGACTCCTACAT
AGTAGTAGGCAGAGGGGAACAACAGATCAATCACCATTGGCACAAGTCGGGTAGCAGCA
TAGGCAAAGCCTTTACCACCACCCTAAAAGGAGCGCAGAGACTAGCAGCTCTAGGAGAC
ACAGCTTGGGACTTTGGGTCGGTTGGAGGGGTATTTACGTCAGTAGGGAAGGCTGTCCAC
CAAGTATTTGGAGGAGCCTTCAGATCACTGTTCGGAGGCATGTCCTGGATAACCCAAGGC
TTGCTGGGGGCTCTCCTCTTGTGGATGGGCATAAATGCTCGGGATAGGTCCATAGCCCTC
ACTTTCCTGGCAGTTGGAGGAGTTCTGCTCTTCCTCTCCGTGAACGTGCACGCTGACACT
GGGTGTGCCATAGACATCAGCCGGCAAGAGCTGAGATGTGGAAGTGGAGTGTTCATACACA
ATGATGTGGAGGCTTGGATGGACCGGTACAAGTATTACCCTGAAACGCCACAAGGCCTAGC
CAAGATCATTCAGAAAGCTCATAAGGAAGGAGTGTGCGGTCTACGATCAGTTTCCAGACTG

File S1 WNV reference sequences

GAGCATCAAATGTGGGAAGCAGTGAAGGACGAGCTGAACACTCTTTTGAAGGAGAATGGT
GTGGACCTTAGTGTCGTGGTTGAGAAACAGGAGGGAATGTACAAGTCAGCACCTAAACGCC
TCACCGCCACCACGGAAAAATTGGAAATTGGCTGGAAGGCCTGGGGAAAGAGTATTTTATT
TGCACCAGAACTCGCCAACAACACCTTTGTGGTTGATGGTCCGGAGACCAAGGAATGTCC
GACTCAGAATCGCGCTTGGAATAGCTTAGAAGTGGAGGATTTTGGATTTGGTCTCACCAGC
ACTCGGATGTTCCTGAAGGTCAGAGAGAGCAACACAACTGAATGTGACTCGAAGATCATTG
GAACGGCTGTCAAGAACAACTTGGCGATCCACAGTGACCTGTCCTATTGGATTGAAAGCAG
GCTCAATGATACGTGGAAGCTTGAAAGGGCAGTTCTGGGTGAAGTCAAATCATGTACGTGG
CCTGAGACGCATACCTTGTGGGGCGATGGAATCCTTGAGAGTGACTTGATAATACCAGTCA
CACTGGCGGGACCACGAAGCAATCACAATCGGAGACCTGGGTACAAGACACAAAACCAGG
GCCCATGGGACGAAGGCCGGGTAGAGATTGACTTCGATTACTGCCCAGGAACTACGGTCA
CCCTGAGTGAGAGCTGCGGACACCGTGGACCTGCCACTCGCACCACCACAGAGAGCGGA
AAGTTGATAACAGATTGGTGCTGCAGGAGCTGCACCTTACCACCACTGCGCTACCAAACTG
ACAGCGGCTGTTGGTATGGTATGGAGATCAGACCACAGAGACATGATGAAAAGACCCTCGT
GCAGTCACAAGTGAATGCTTATAATGCTGATATGATTGACCCTTTTCAGTTGGGCCTTCTGGT
CGTGTTCTTGGCCACCCAGGAGGTCCTTCGCAAGAGGTGGACAGCCAAGATCAGCATGCC
AGCTATACTGATTGCTCTGCTAGTCCTGGTGTTTGGGGGCATTACTTACACTGATGTGTTACG
CTATGTCATCTTGGTGGGGGCAGCTTTCGCAGAATCTAATTCGGGAGGAGACGTGGTACAC
TTGGCGCTCATGGCGACCTTCAAGATACAACCAGTGTTTATGGTGGCATCGTTTCTCAAAGC
GAGATGGACCAACCAGGAGAACATTTTGTTGATGTTGGCGGCTGTTTTCTTTCAAATGGCTT
ATCACGATGCCCGCCAAATTCTGCTCTGGGAGATCCCTGATGTGTTGAATTCACTGGCGGTA
GCTTGGATGATACTGAGAGCCATAACATTCACAACGACATCAAACGTGGTTGTTCCGCTGCT
AGCCCTGCTAACACCCGGGCTGAGATGCTTGAATCTGGATGTGTACAGGATACTGCTGTTG
ATGGTCGGAATAGGCAGCTTGATCAGGGAGAAGAGGAGTGCAGCCGCAAAAAAGAAAGGA
GCAAGTCTGCTATGCTTGGCTCTAGCCTCAACAGGACTTTTCAACCCCATGATCCTTGCTGC
TGGACTGATTGCATGTGATCCCAACCGTAAACGCGGATGGCCCGCAACTGAAGTGATGACA
GCTGTCGGCCTAATGTTTGCCATCGTCGGAGGGCTGGCAGAGCTTGACATTGACTCCATGG
CCATTCCAATGACTATCGCGGGGCTCATGTTTGCTGCTTTCGTGATTTCTGGGAAATCAACA
GATATGTGGATTGAGAGAACGGCGGACATTTCCTGGGAAAGTGATGCAGAAATTACAGGCT
CGAGCGAAAGAGTTGATGTGCGGCTTGATGATGATGGAAACTTCCAGCTCATGAATGATCC
AGGAGCACCTTGGAAGATATGGATGCTCAGAATGGTCTGTCTCGCGATTAGTGCGTACACC
CCCTGGGCAATCTTGCCCTCAGTAGTTGGATTTTGGATAACTCTCCAATACACAAAGAGAGG
AGGCGTGTTGTGGGACACTCCCTCACCAAAGGAGTACAAAAAGGGGGACACGACCACCGG
CGTCTACAGGATCATGACTCGTGGGCTGCTCGGCAGTTATCAAGCAGGAGCGGGCGTGAT
GGTTGAAGGTGTTTTCCACACCCTTTGGCATACAACAAAAGGAGCCGCTTTGATGAGCGGA
GAGGGCCGCCTGGACCCATACTGGGGCAGTGTCAAGGAGGATCGACTTTGTTACGGAGGA
CCCTGGAAATTGCAGCACAAGTGGAACGGGCAGGATGAGGTGCAGATGATTGTGGTGGAA
CCTGGCAAGAACGTTAAGAACGTCCAGACGAAACCAGGGGTGTTCAAAACACCTGAAGGA
GAAATCGGGGCCGTGACTTTGGACTTCCCCACTGGAACATCAGGCTCACCAATAGTGGACA
AAAACGGTGATGTGATTGGGCTTTATGGCAATGGAGTCATAATGCCCAACGGCTCATACATA
AGCGCGATAGTGCAGGGTGAAAGGATGGATGAGCCAATCCCAGCCGGATTCGAACCTGAG
ATGCTGAGGAAAAAACAGATCACTGTACTGGATCTCCATCCCGGCGCCGGTAAAACAAGGA
GGATTCTGCCACAGATCATCAAAGAGGCCATAAACAGAAGACTGAGAACAGCCGTGCTAGC
GCCAACCAGGGTTGTGGCTGCTGAGATGGCTGAAGCACTGAGAGGACTGCCCATCCGGTA
CCAGACATCCGCAGTGCCCAGAGAACATAATGGAAATGAGATTGTTGATGTCATGTGTCATG
CTACCCTCACCCACAGGCTGATGTCTCCTCACAGGGTGCCGAACTACAACCTGTTCGTGAT
GGATGAGGCTCATTTCACCGACCCAGCTAGCATTGCAGCAAGAGGTTACATTTCCACAAAG

File S1 WNV reference sequences

GTCGAGCTAGGGGAGGCGGCGGCAATATTCATGACAGCCACCCCACCAGGCACTTCAGAT
CCATTCCCAGAGTCCAATTCACCAATTTCCGACTTACAGACTGAGATCCCGGATCGAGCTTG
GAACTCTGGATACGAATGGATCACAGAATACACCGGGAAGACGGTTTGGTTTGTGCCTAGT
GTCAAGATGGGGAATGAGATTGCCCTTTGCCTACAACGTGCTGGAAAGAAAGTAGTCCAAT
TGAACAGAAAGTCGTACGAGACGGAGTACCCAAAATGTAAGAACGATGATTGGGACTTTGTT
ATCACAACAGACATATCTGAAATGGGGGCTAACTTCAAGGCGAGCAGGGTGATTGACAGCC
GGAAGAGTGTGAAACCAACCATCATAACAGAAGGAGAAGGGAGAGTGATCCTGGGAGAAC
CATCTGCAGTGACAGCAGCTAGTGCCGCCCAGAGACGTGGACGTATCGGTAGAAATCCGT
CGCAAGTTGGTGATGAGTACTGTTATGGGGGGCACACGAATGAAGACGACTCGAACTTCGC
CCATTGGACTGAGGCACGAATCATGCTGGACAACATCAACATGCCAAACGGACTGATCGCT
CAATTCTACCAACCAGAGCGTGAGAAGGTATATACCATGGATGGGGAATACCGGCTCAGAG
GAGAAGAGAGAAAAAACTTTCTGGAACTGTTGAGGACTGCAGATCTGCCAGTTTGGCTGGC
TTACAAGGTTGCAGCGGCTGGAGTGTCATACCACGACCGGAGGTGGTGCTTTGATGGTCCT
AGGACAAACACAATTTTAGAAGACAACAACGAAGTGGAAGTCATCACGAAGCTTGGTGAAA
GGAAGATTCTGAGGCCGCGCTGGATTGATGCCAGGGTGTACTCGGATCACCAGGCACTAA
AGGCGTTCAAGGACTTCGCCTCGGGAAAACGTTCTCAGATAGGGCTCATTGAGGTTCTGG
GAAAGATGCCTGAGCACTTCATGGGGAAGACATGGGAAGCACTTGACACCATGTACGTTGT
GGCCACTGCAGAGAAAGGAGGAAGAGCTCACAGAATGGCCCTGGAGGAACTGCCAGATG
CTCTTCAGACAATTGCCTTGATTGCCTTATTGAGTGTGATGACCATGGGAGTATTCTTCCTCC
TCATGCAGCGGAAGGGCATTGGAAAGATAGGTTTGGGAGGCGCTGTCTTGGGAGTCGCGA
CCTTTTTCTGTTGGATGGCTGAAGTTCCAGGAACGAAGATCGCCGGAATGTTGCTGCTCTC
CCTTCTCTTGATGATTGTGCTAATTCCTGAGCCAGAGAAGCAACGTTCGCAGACAGACAAC
CAGCTAGCCGTGTTCCTGATTTGTGTCATGACCCTTGTGAGCGCAGTGGCAGCCAACGAGA
TGGGTTGGCTAGATAAGACCAAGAGTGACATAAGCAGTTTGTTTGGGCAAAGAATTGAGGT
CAAGGAGAATTTCAGCATGGGAGAGTTTCTTCTGGACTTGAGGCCGGCAACAGCCTGGTC
ACTGTACGCTGTGACAACAGCGGTCCTCACTCCACTGCTAAAGCATTTGATCACGTCAGATT
ACATCAACACCTCATTGACCTCAATAAACGTTCAGGCAAGTGCACTATTCACACTCGCGCGA
GGCTTCCCCTTCGTCGATGTTGGAGTGTCGGCTCTCCTGCTAGCAGCCGGATGCTGGGGA
CAAGTCACCCTCACCGTTACGGTAACAGCGGCAACACTCCTTTTTTGCCACTATGCCTACAT
GGTTCCCGGTTGGCAAGCTGAGGCAATGCGCTCAGCCCAGCGGCGGACAGCGGCCGGAA
TCATGAAGAACGCTGTAGTGGATGGCATCGTGGCCACGGACGTCCCAGAATTAGAGCGCA
CCACACCCATCATGCAGAAGAAAGTTGGACAGATCATGCTGATCTTGGTGTCTCTAGCTGCA
GTAGTAGTGAACCCGTCTGTGAAGACAGTACGAGAAGCCGGAATTTTGATCACGGCCGCAG
CGGTGACGCTTTGGGAGAATGGAGCAAGCTCTGTTTGGAACGCAACAACTGCCATCGGAC
TCTGCCACATCATGCGTGGGGGTTGGTTGTCATGTCTATCCATAACATGGACACTCATAAAG
AACATGGAAAAACCAGGACTAAAAAGAGGTGGGGCAAAAGGACGCACCTTGGGAGAGGTT
TGGAAAGAAAGACTCAACCAGATGACAAAAGAAGAGTTCACTAGGTACCGCAAAGAGGCCA
TCATCGAAGTCGATCGCTCAGCGGCAAAACACGCCAGGAAAGAAGGCAATGTCACTGGAG
GGCATCCAGTCTCTAGGGGCACAGCAAAACTGAGATGGCTGGTCGAACGGAGGTTTCTCG
AACCGGTCGGAAAAGTGATTGACCTTGGATGTGGAAGAGGCGGTTGGTGTTACTATATGGC
AACCCAAAAAAGAGTCCAAGAAGTCAGAGGGTACACAAAGGGCGGTCCCGGACATGAAGA
GCCCCAACTAGTGCAAAGTTATGGATGGAACATTGTCACCATGAAGAGTGGAGTGGATGTG
TTCTACAGACCTTCTGAGTGTTGTGACACCCTCCTTTGTGACATCGGAGAGTCCTCGTCAA
GTGCTGAGGTTGAAGAGCATAGGACGATTCGGGTCCTTGAAATGGTTGAGGACTGGCTGC
ACCGAGGGCCAAGGGAATTTTGCGTGAAGGTGCTCTGTCCCTACATGCCGAAAGTCATAGA
GAAGATGGAGCTGCTCCAACGCCGGTATGGGGGGGGACTGGTCAGAAACCCACTCTCACG
GAATTCCACGCACGAGATGTATTGGGTGAGTCGAGCTTCAGGCAATGTGGTACATTCAGTG

File S1 WNV reference sequences

AATATGACCAGCCAGGTGCTCCTAGGAAGAATGGAAAAAAGGACCTGGAAGGGACCCCAAT
ACGAGGAAGATGTAAACTTGGGAAGTGGAACCAGGGCGGTGGGAAAACCCCTGCTCAACT
CAGACACCAGTAAAATCAAGAACAGGATTGAACGACTCAGGCGTGAGTACAGTTCGACGTG
GCACCACGATGAGAACCACCCATATAGAACCTGGAACTATCACGGCAGTTATGATGTGAAGC
CCACAGGCTCCGCCAGTTCGCTGGTCAATGGAGTGGTCAGGCTCCTCTCAAAACCATGGG
ACACCATCACGAATGTTACCACCATGGCCATGACTGACACTACTCCCTTCGGGCAGCAGCG
AGTGTTCAAAGAGAAGGTGGACACGAAAGCTCCTGAACCGCCAGAAGGAGTGAAGTACGT
GCTCAACGAGACCACCAACTGGTTGTGGGCGTTTTTGGCCAGAGAAAAACGTCCCAGAAT
GTGCTCTCGAGAGGAATTCATAAGAAAGGTCAACAGCAATGCAGCTTTGGGTGCCATGTTT
GAAGAGCAGAATCAATGGAGGAGCGCCAGAGAAGCAGTTGAAGATCCAAAATTTTGGGAGA
TGGTGGATGAGGAGCGCGAGGCACATCTGCGGGGGGAATGTCACACTTGCATTTACAACAT
GATGGGAAAGAGAGAGAAAAAACCCGGAGAGTTCGGAAAGGCCAAGGGAAGCAGAGCCA
TTTGGTTCATGTGGCTCGGAGCTCGCTTTCTGGAGTTCGAGGCTCTGGGTTTTCTCAATGA
AGACCACTGGCTTGGAAGAAAGAACTCAGGAGGAGGTGTCGAGGGCTTGGGCCTCCAAAA
ACTGGGTTACATCCTGCGTGAAGTTGGCACCCGGCCTGGGGGCAAGATCTATGCTGATGAC
ACAGCTGGCTGGGACACCCGCATCACGAGAGCTGACTTGGAAAATGAAGCTAAGGTGCTT
GAGCTGCTTGATGGGGAACATCGGCGTCTTGCCAGGGCCATCATTGAGCTCACCTATCGTC
ACAAAGTTGTGAAAGTGATGCGCCCGGCTGCTGATGGAAGAACCGTCATGGATGTTATCTC
CAGAGAAGATCAGAGGGGGAGTGGACAAGTTGTCACCTACGCCCTAAACACTTTCACCAAC
CTGGCCGTCCAGCTGGTGAGGATGATGGAAGGGGAAGGAGTGATTGGCCCAGATGATGTG
GAGAAACTCACAAAAGGGAAAGGACCCAAAGTCAGGACCTGGCTGTTTGAGAATGGGGAA
GAAAGACTCAGCCGCATGGCTGTCAGTGGAGATGACTGTGTGGTAAAGCCCCTGGACGAT
CGCTTTGCCACCTCGCTCCACTTCCTCAATGCTATGTCAAAGGTTCGCAAAGACATCCAAGA
GTGGAAACCGTCAACTGGATGGTATGATTGGCAGCAGGTTCCATTTTGCTCAAACCATTTCA
CTGAATTGATCATGAAAGATGGAAGAACACTGGTGGTTCCATGCCGAGGACAGGATGAATT
GGTAGGCAGAGCTCGCATATCTCCAGGGGCCGGATGGAACGTCCGCGACACTGCTTGTCT
GGCTAAGTCTTATGCCCAGATGTGGCTGCTTCTGTACTTCCACAGAAGAGACCTGCGGCTC
ATGGCCAACGCCATTTGCTCCGCTGTCCCTGTGAATTGGGTCCCTACCGGAAGAACCACGT
GGTCCATCCATGCAGGAGGAGAGTGGATGACAACAGAGGACATGTTGGAGGTCTGGAACC
GTGTTTGGATAGAGGAGAATGAATGGATGGAAGACAAAACCCCAGTGGAGAAATGGAGTGA
CGTCCCATATTCAGGAAAACGAGAGGACATCTGGTGTGGCAGCCTGATTGGCACAAGAGCC
CGAGCCACGTGGGCAGAAAACATCCAGGTGGCTATCAACCAAGTCAGAGCAATCATCGGA
GATGAGAAGTATGTGGACTACATGAGTTCACTAAAGAGATATGAAGACACAACTTTGGTTGA
GGACACAGTACTGTAGATATTTAATCAATTGTAAATAGACAATATAAGTATGCATAAAAGTGTAG
TTTTATAGTAGTATTTAGTGGTGTTAGTGTAAATAGTTAAGAAAATTTTGAGGAGAAAGTCAGG
CCGGGAAGTTCCCGCCACCGGAAGTTGAGTAGACGGTGCTGCCTGCGACTCAACCCCAG
GAGGACTGGGTGAACAAAGCCGCGAAGTGATCCATGTAAGCCCTCAGAACCGTCTCGGAA
GGAGGACCCCACATGTTGTAACTTCAAAGCCCAATGTCAGACCACGCTACGGCGTGCTACT
CTGCGGAGAGTGCAGTCTGCGATAGTGCCCCAGGAGGACTGGGTTAACAAAGGCAAACCA
ACGCCCCACGCGGCCCTAGCCCCGGTAATGGTGTTAACCAGGGCGAAAGGACTAGAGGTT
AGAGGAGACCCCGCGGTTTAAAGTGCACGGCCCAGCCTGGCTGAAGCTGTAGGTCAGGG
GAAGGACTAGAGGTTAGTGGAGACCCCGTGCCACAAAACACCACAACAAAACAGCATATTG
ACACCTGGGATAGACTAGGAGATCTTCTGCTCTGCACAACCAGCCACACGGCACAGTGCG
CCGACAATGGTGGCTGGTGGTGCGAGAACACAGGATCT

File S1 WNV reference sequences

> E-MAX/NS5+CG

E sequences are in green.
NS5 sequences are in blue.

AGTAGTTCGCCTGTGTGAGCTGACAAACTTAGTAGTGTTTGTGAGGATTAACAACAATTAAC
ACAGTGCGAGCTGTTTCTTAGCACGAAGATCTCGATGTCTAAGAAACCAGGAGGGCCCGGC
AAGAGCCGGGCTGTCAATATGCTAAAACGCGGAATGCCCCGCGTGTTGTCCTTGATTGGAC
TGAAGAGGGCTATGTTGAGCCTGATCGACGGCAAGGGGCCAATACGATTTGTGTTGGCTCT
CTTGGCGTTCTTCAGGTTCACAGCAATTGCTCCGACCCGAGCAGTGCTGGATCGATGGAGA
GGTGTGAACAAACAAACAGCGATGAAACACCTTCTGAGTTTTAAGAAGGAACTAGGGACCT
TGACCAGTGCTATCAATCGGCGGAGCTCAAAACAAAAGAAAAGAGGAGGAAAGACCGGAAT
TGCAGTCATGATTGGCCTGATCGCCAGCGTAGGAGCAGTTACCCTCTCTAACTTCCAAGGG
AAGGTGATGATGACGGTAAATGCTACTGACGTCACAGATGTCATCACGATTCCAACAGCTGC
TGGAAAGAACCTATGCATTGTCAGAGCAATGGATGTGGGATACATGTGCGATGATACTATCA
CTTATGAATGCCCAGTACTGTCGGCTGGTAATGATCCAGAAGACATCGACTGTTGGTGCACA
AAGTCAGCAGTCTACGTCAGGTATGGAAGATGCACCAAGACACGCCACTCAAGACGCAGTC
GGAGGTCACTGACAGTGCAGACACACGGAGAAAGCACTCTAGCGAACAAGAAGGGGGCTT
GGATGGACAGCACCAAGGCCACAAGGTATTTGGTAAAAACAGAATCATGGATCTTGAGGAA
CCCTGGATATGCCCTGGTGGCAGCCGTCATTGGTTGGATGCTTGGGAGCAACACCATGCA
GAGAGTTGTGTTTGTCGTGCTATTGCTTTTGGTGGCCCCAGCTTACAGC**TTCAACTGCCTT**
GGAATGAGCAACAGAGACTTTTTGGAAGGCGTTTCCGGCGCGACGTGGGTCGATTTAGTT
CTCGAAGGGGATAGTTGCGTTACGATAATGTCGAAAGACAAACCGACGATCGACGTTAAA
ATGATGAATATGGAGGCCGCGAATCTAGCGGAAGTTCGCAGTTATTGTTATTTAGCAACCG
TAAGCGATCTTTCGACGAAAGCCGCGTGTCCGACGATGGGCGAAGCGCACAACGATAAA
CGGGCGGATCCCGCGTTTGTTTGTCGACAAGGCGTCGTCGATCGCGGTTGGGGAAATGG
TTGTGGACTATTCGGCAAAGGAAGTATCGATACGTGCGCGAAATTTGCGTGTTCTACTAAA
GCGATAGGACGAACGATATTAAAAGAAAATATCAAATACGAAGTCGCGATTTTTGTCCACG
GACCGACGACGGTCGAATCGCACGGAAATTATTCGACGCAAGTCGGCGCGACGCAGGC
CGGTCGACTAAGTATAACGCCCGCGGCGCCGTCGTACACGCTAAAACTGGGCGAATATG
GCGAGGTTACGGTCGACTGCGAACCGCGTTCGGGTATCGATACGAACGCGTATTATGTTAT
GACGGTCGGAACGAAAACGTTTTTAGTACATCGGGAATGGTTCATGGATCTAAACCTACC
GTGGAGCAGTGCCGGAAGTACCGTTTGGCGTAACCGGGAAACGTTAATGGAATTCGAAG
AACCGCACGCGACTAAACAGTCCGTAATCGCGTTAGGATCGCAAGAAGGCGCGCTTCAT
CAAGCGTTAGCCGGCGCGATTCCGGTCGAATTTTCGAGTAATACCGTCAAATTAACATCCG
GTCATTTGAAATGTCGGGTCAAAATGGAAAAATTACAATTAAAAGGAACGACTTACGGCG
TATGTTCGAAAGCGTTCAAGTTTCTCGGTACACCCGCCGATACCGGTCACGGAACCGTCG
TTTTGGAATTACAATATACCGGAACCGATGGACCGTGTAAAGTTCCGATATCGTCCGTGGC
GTCGTTGAACGATCTAACACCCGTCGGCCGATTAGTCACCGTTAATCCGTTCGTTTCCGTC
GCGACGGCGAACGCGAAAGTACTTATAGAATTGGAACCGCCGTTTGGCGATTCGTATATC
GTCGTCGGCCGCGGGGAACAACAAATAAATCACCATTGGCATAAGTCCGGAAGTAGTATC
GGAAAAGCGTTTACGACGACGCTAAAAGGCGCGCAACGACTAGCCGCGCTCGGCGATAC
CGCGTGGGATTTCGGATCCGTCGGGGGCGTTTTTACGTCCGTCGGTAAAGCCGTACATCA
AGTATTCGGCGGGGCGTTTCGCTCGCTTTTCGGCGGAATGTCGTGGATAACGCAAGGATT
ACTGGGCGCGCTACTTTTATGGATGGGAATAAACGCGCGGGATCGTTCGATCGCGCTAAC
GTTTCTCGCCGTCGGCGGAGTTCTGCTCTTCCTCTCCGTGAACGTGCACGCTGACACTGG
GTGTGCCATAGACATCAGCCGGCAAGAGCTGAGATGTGGAAGTGGAGTGTTCATACACAAT
GATGTGGAGGCTTGGATGGACCGGTACAAGTATTACCCTGAAACGCCACAAGGCCTAGCCA

File S1 WNV reference sequences

AGATCATTCAGAAAGCTCATAAGGAAGGAGTGTGCGGTCTACGATCAGTTTCCAGACTGGA
GCATCAAATGTGGGAAGCAGTGAAGGACGAGCTGAACACTCTTTTGAAGGAGAATGGTGTG
GACCTTAGTGTCGTGGTTGAGAAACAGGAGGGAATGTACAAGTCAGCACCTAAACGCCTCA
CCGCCACCACGGAAAAATTGGAAATTGGCTGGAAGGCCTGGGGAAAGAGTATTTTATTTGC
ACCAGAACTCGCCAACAACACCTTTGTGGTTGATGGTCCGGAGACCAAGGAATGTCCGACT
CAGAATCGCGCTTGGAATAGCTTAGAAGTGGAGGATTTTGGATTTGGTCTCACCAGCACTC
GGATGTTCCTGAAGGTCAGAGAGAGCAACACAACTGAATGTGACTCGAAGATCATTGGAAC
GGCTGTCAAGAACAACTTGGCGATCCACAGTGACCTGTCCTATTGGATTGAAAGCAGGCTC
AATGATACGTGGAAGCTTGAAAGGGCAGTTCTGGGTGAAGTCAAATCATGTACGTGGCCTG
AGACGCATACCTTGTGGGGCGATGGAATCCTTGAGAGTGACTTGATAATACCAGTCACACTG
GCGGGACCACGAAGCAATCACAATCGGAGACCTGGGTACAAGACACAAAACCAGGGCCCA
TGGGACGAAGGCCGGGTAGAGATTGACTTCGATTACTGCCCAGGAACTACGGTCACCCTG
AGTGAGAGCTGCGGACACCGTGGACCTGCCACTCGCACCACCACAGAGAGCGGAAAGTT
GATAACAGATTGGTGCTGCAGGAGCTGCACCTTACCACCACTGCGCTACCAAACTGACAGC
GGCTGTTGGTATGGTATGGAGATCAGACCACAGAGACATGATGAAAAGACCCTCGTGCAGT
CACAAGTGAATGCTTATAATGCTGATATGATTGACCCTTTTCAGTTGGGCCTTCTGGTCGTGT
TCTTGGCCACCCAGGAGGTCCTTCGCAAGAGGTGGACAGCCAAGATCAGCATGCCAGCTA
TACTGATTGCTCTGCTAGTCCTGGTGTTTGGGGGCATTACTTACACTGATGTGTTACGCTATG
TCATCTTGGTGGGGGCAGCTTTCGCAGAATCTAATTCGGGAGGAGACGTGGTACACTTGGC
GCTCATGGCGACCTTCAAGATACAACCAGTGTTTATGGTGGCATCGTTTCTCAAAGCGAGAT
GGACCAACCAGGAGAACATTTTGTTGATGTTGGCGGCTGTTTTCTTTCAAATGGCTTATCAC
GATGCCCGCCAAATTCTGCTCTGGGAGATCCCTGATGTGTTGAATTCACTGGCGGTAGCTT
GGATGATACTGAGAGCCATAACATTCACAACGACATCAAACGTGGTTGTTCCGCTGCTAGCC
CTGCTAACACCCGGGCTGAGATGCTTGAATCTGGATGTGTACAGGATACTGCTGTTGATGGT
CGGAATAGGCAGCTTGATCAGGGAGAAGAGGAGTGCAGCCGCAAAAAAGAAAGGAGCAAG
TCTGCTATGCTTGGCTCTAGCCTCAACAGGACTTTTCAACCCCATGATCCTTGCTGCTGGAC
TGATTGCATGTGATCCCAACCGTAAACGCGGATGGCCCGCAACTGAAGTGATGACAGCTGT
CGGCCTAATGTTTGCCATCGTCGGAGGGCTGGCAGAGCTTGACATTGACTCCATGGCCATT
CCAATGACTATCGCGGGGCTCATGTTTGCTGCTTTCGTGATTTCTGGGAAATCAACAGATAT
GTGGATTGAGAGAACGGCGGACATTTCCTGGGAAAGTGATGCAGAAATTACAGGCTCGAGC
GAAAGAGTTGATGTGCGGCTTGATGATGATGGAAACTTCCAGCTCATGAATGATCCAGGAG
CACCTTGGAAGATATGGATGCTCAGAATGGTCTGTCTCGCGATTAGTGCGTACACCCCCTG
GGCAATCTTGCCCTCAGTAGTTGGATTTTGGATAACTCTCCAATACACAAAGAGAGGAGGCG
TGTTGTGGGACACTCCCTCACCAAAGGAGTACAAAAAGGGGGACACGACCACCGGCGTCT
ACAGGATCATGACTCGTGGGCTGCTCGGCAGTTATCAAGCAGGAGCGGGCGTGATGGTTG
AAGGTGTTTTCCACACCCTTTGGCATACAACAAAAGGAGCCGCTTTGATGAGCGGAGAGGG
CCGCCTGGACCCATACTGGGGCAGTGTCAAGGAGGATCGACTTTGTTACGGAGGACCCTG
GAAATTGCAGCACAAGTGGAACGGGCAGGATGAGGTGCAGATGATTGTGGTGGAACCTGG
CAAGAACGTTAAGAACGTCCAGACGAAACCAGGGGTGTTCAAAACACCTGAAGGAGAAATC
GGGGCCGTGACTTTGGACTTCCCCACTGGAACATCAGGCTCACCAATAGTGGACAAAAAC
GGTGATGTGATTGGGCTTTATGGCAATGGAGTCATAATGCCCAACGGCTCATACATAAGCGC
GATAGTGCAGGGTGAAAGGATGGATGAGCCAATCCCAGCCGGATTCGAACCTGAGATGCTG
AGGAAAAAACAGATCACTGTACTGGATCTCCATCCCGGCGCCGGTAAAACAAGGAGGATTC
TGCCACAGATCATCAAAGAGGCCATAAACAGAAGACTGAGAACAGCCGTGCTAGCGCCAAC
CAGGGTTGTGGCTGCTGAGATGGCTGAAGCACTGAGAGGACTGCCCATCCGGTACCAGAC
ATCCGCAGTGCCCAGAGAACATAATGGAAATGAGATTGTTGATGTCATGTGTCATGCTACCC
TCACCCACAGGCTGATGTCTCCTCACAGGGTGCCGAACTACAACCTGTTCGTGATGGATGA

File S1 WNV reference sequences

GGCTCATTTCACCGACCCAGCTAGCATTGCAGCAAGAGGTTACATTTCCACAAAGGTCGAG
CTAGGGGAGGCGGCGGCAATATTCATGACAGCCACCCCACCAGGCACTTCAGATCCATTCC
CAGAGTCCAATTCACCAATTTCCGACTTACAGACTGAGATCCCGGATCGAGCTTGGAACTCT
GGATACGAATGGATCACAGAATACACCGGGAAGACGGTTTGGTTTGTGCCTAGTGTCAAGA
TGGGGAATGAGATTGCCCTTTGCCTACAACGTGCTGGAAAGAAAGTAGTCCAATTGAACAG
AAAGTCGTACGAGACGGAGTACCCAAAATGTAAGAACGATGATTGGGACTTTGTTATCACAA
CAGACATATCTGAAATGGGGGCTAACTTCAAGGCGAGCAGGGTGATTGACAGCCGGAAGA
GTGTGAAACCAACCATCATAACAGAAGGAGAAGGGAGAGTGATCCTGGGAGAACCATCTGC
AGTGACAGCAGCTAGTGCCGCCCAGAGACGTGGACGTATCGGTAGAAATCCGTCGCAAGT
TGGTGATGAGTACTGTTATGGGGGGCACACGAATGAAGACGACTCGAACTTCGCCCATTGG
ACTGAGGCACGAATCATGCTGGACAACATCAACATGCCAAACGGACTGATCGCTCAATTCTA
CCAACCAGAGCGTGAGAAGGTATATACCATGGATGGGGAATACCGGCTCAGAGGAGAAGAG
AGAAAAAACTTTCTGGAACTGTTGAGGACTGCAGATCTGCCAGTTTGGCTGGCTTACAAGG
TTGCAGCGGCTGGAGTGTCATACCACGACCGGAGGTGGTGCTTTGATGGTCCTAGGACAA
ACACAATTTTAGAAGACAACAACGAAGTGGAAGTCATCACGAAGCTTGGTGAAAGGAAGATT
CTGAGGCCGCGCTGGATTGATGCCAGGGTGTACTCGGATCACCAGGCACTAAAGGCGTTC
AAGGACTTCGCCTCGGGAAAACGTTCTCAGATAGGGCTCATTGAGGTTCTGGGAAAGATGC
CTGAGCACTTCATGGGGAAGACATGGGAAGCACTTGACACCATGTACGTTGTGGCCACTGC
AGAGAAAGGAGGAAGAGCTCACAGAATGGCCCTGGAGGAACTGCCAGATGCTCTTCAGAC
AATTGCCTTGATTGCCTTATTGAGTGTGATGACCATGGGAGTATTCTTCCTCCTCATGCAGC
GGAAGGGCATTGGAAAGATAGGTTTGGGAGGCGCTGTCTTGGGAGTCGCGACCTTTTTCT
GTTGGATGGCTGAAGTTCCAGGAACGAAGATCGCCGGAATGTTGCTGCTCTCCCTTCTCTT
GATGATTGTGCTAATTCCTGAGCCAGAGAAGCAACGTTCGCAGACAGACAACCAGCTAGCC
GTGTTCCTGATTTGTGTCATGACCCTTGTGAGCGCAGTGGCAGCCAACGAGATGGGTTGG
CTAGATAAGACCAAGAGTGACATAAGCAGTTTGTTTGGGCAAAGAATTGAGGTCAAGGAGA
ATTTCAGCATGGGAGAGTTTCTTCTGGACTTGAGGCCGGCAACAGCCTGGTCACTGTACGC
TGTGACAACAGCGGTCCTCACTCCACTGCTAAAGCATTTGATCACGTCAGATTACATCAACA
CCTCATTGACCTCAATAAACGTTCAGGCAAGTGCACTATTCACACTCGCGCGAGGCTTCCC
CTTCGTCGATGTTGGAGTGTCGGCTCTCCTGCTAGCAGCCGGATGCTGGGGACAAGTCAC
CCTCACCGTTACGGTAACAGCGGCAACACTCCTTTTTTGCCACTATGCCTACATGGTTCCCG
GTTGGCAAGCTGAGGCAATGCGCTCAGCCCAGCGGCGGACAGCGGCCGGAATCATGAAG
AACGCTGTAGTGGATGGCATCGTGGCCACGGACGTCCCAGAATTAGAGCGCACCACACCC
ATCATGCAGAAGAAAGTTGGACAGATCATGCTGATCTTGGTGTCTCTAGCTGCAGTAGTAGT
GAACCCGTCTGTGAAGACAGTACGAGAAGCCGGAATTTTGATCACGGCCGCAGCGGTGAC
GCTTTGGGAGAATGGAGCAAGCTCTGTTTGGAACGCAACAACTGCCATCGGACTCTGCCA
CATCATGCGTGGGGGTTGGTTGTCATGTCTATCCATAACATGGACACTCATAAAGAACATGG
AAAAACCAGGACTAAAAAGA**GGTGGGGCAAAAGGACGCACCTTGGGAGAGGTTTGGAAA**
GAAAGACTCAACCAGATGACAAAAGAAGAGTTCACTAGGTACCGCAAAGAAGCGATCAT
AGAAGTCGATCGATCGGCAGCGAAACACGCGCGAAAAGAAGGAAACGTAACGGGCGGT
CATCCCGTTTCACGCGGGACGGCGAAACTTCGATGGCTTGTAGAACGGCGGTTTCTCGA
ACCGGTCGGAAAAGTGATAGATCTAGGATGCGGACGCGGAGGTTGGTGTTATTACATGGC
GACACAAAAACGCGTTCAAGAAGTCCGTGGGTACACGAAAGGAGGTCCGGGACACGAA
GAACCGCAACTCGTTCAAAGTTACGGATGGAACATCGTCACCATGAAGAGCGGCGTCGA
CGTTTTTATCGACCTTCGGAATGTTGCGACACACTTCTATGTGACATTGGCGAGTCTTCAT
CAAGTGCGGAAGTCGAAGAACATCGCACAATTCGCGTTCTAGAAATGGTCGAAGACTGG
CTACATCGCGGACCGCGCGAATTTTGTGTGAAAGTGCTCTGTCCGTACATGCCGAAAGTT
ATCGAAAAGATGGAACTGCTTCAACGTCGTTATGGCGGAGGACTCGTGCGAAATCCGCTG

File S1 WNV reference sequences

TCTCGGAATTCGACGCACGAAATGTATTGGGTGAGTCGAGCGTCGGGTAATGTCGTCCAT
TCGGTAAACATGACGAGCCAAGTTCTCCTCGGACGAATGGAAAAACGAACGTGGAAGGG
ACCGCAATACGAAGAAGATGTCAATTTGGGAAGCGGAACCCGAGCGGTCGGAAAACCGC
TTCTGAATTCCGACACGAGCAAAATCAAAAACCGAATCGAACGACTCCGACGCGAATACA
GTTCGACATGGCATCACGATGAGAATCATCCTTATCGAACCTGGAATTATCACGGGAGTTA
CGACGTGAAGCCGACGGGATCGGCCAGTTCGCTCGTGAACGGAGTGGTTCGACTTCTGT
CAAAACCGTGGGACACGATAACGAACGTAACGACAATGGCCATGACGGACACGACTCCG
TTCGGTCAGCAACGCGTTTTCAAAGAGAAAGTTGACACGAAAGCGCCGGAACCGCCGG
AAGGCGTGAAGTACGTGCTTAACGAAACGACCAATTGGTTGTGGCGTTTTTGGCACGC
GAAAAACGACCTCGAATGTGTTCGCGAGAAGAATTCATTCGAAAAGTCAACAGCAACGC
GGCATTGGGCGCGATGTTCGAAGAGCAAAATCAATGGCGGAGCGCGCGAGAAGCGGTT
GAAGATCCCAAATTTTGGGAAATGGTGGACGAGGAGCGAGAAGCGCATCTGCGCGGCGA
ATGTCACACGTGCATTTACAACATGATGGGAAAGCGCGAGAAAAAACCAGGCGAATTTG
GAAAAGCAAAAGGAAGCCGCGCGATTTGGTTCATGTGGCTCGGCGCTCGATTTCTCGAAT
TTGAGGCGCTCGGTTTTCTCAACGAAGATCATTGGCTAGGACGAAAAAATTCCGGAGGCG
GCGTCGAAGGATTGGGTCTGCAAAAACTCGGTTACATTCTTCGTGAAGTTGGAACGCGGC
CAGGCGGAAAGATCTACGCGGACGACACGGCAGGATGGGACACGCGAATTACGCGAGC
GGATTTGGAAAACGAAGCGAAAGTACTCGAGCTGCTTGATGGCGAACATCGTCGTCTCG
CGCGAGCCATCATCGAGCTGACGTATCGTCACAAAGTCGTCAAAGTGATGCGGCCGGCG
GCGGACGGACGAACGGTGATGGACGTCATCTCGCGAGAAGATCAGCGTGGGAGTGGAC
AAGTCGTTACATACGCGCTCAACACGTTCACCAATCTCGCCGTGCAACTAGTTCGAATGAT
GGAAGGAGAAGGCGTGATCGGACCGGATGACGTCGAAAAACTTACAAAAGGCAAAGGA
CCGAAAGTACGGACGTGGCTGTTCGAAAATGGAGAAGAACGACTGAGTCGAATGGCGGT
TAGTGGCGACGATTGCGTCGTTAAACCGCTCGACGATCGATTCGCGACCTCGCTTCATTT
CCTTAACGCGATGTCGAAGGTTCGAAAAGACATTCAAGAATGGAAACCCTCGACGGGAT
GGTATGATTGGCAACAAGTTCCGTTTTGTTCGAATCATTTCACCGAATTGATAATGAAAGA
CGGACGAACGCTTGTTGTTCCTTGTCGAGGACAAGATGAATTGGTCGGGCGCGCACGAA
TTTCGCCGGGCGCAGGATGGAATGTTCGCGACACGGCGTGTCTCGCAAAATCGTACGCG
CAGATGTGGCTGCTTCTGTATTTTCATCGACGCGATCTTCGACTCATGGCGAACGCGATTT
GTTCGGCAGTTCCGGTGAATTGGGTTCCAACGGGACGAACAACGTGGTCAATCCACGCG
GGCGGTGAGTGGATGACAACGGAAGACATGTTGGAAGTCTGGAACCGCGTTTGGATAGA
AGAGAATGAATGGATGGAAGACAAAACGCCGGTCGAGAAATGGAGCGATGTTCCGTATT
CGGGAAAACGCGAGGACATTTGGTGCGGGAGTCTGATCGGAACGCGAGCGCGCGCCAC
GTGGGCGGAAAACATTCAAGTCGCAATCAATCAAGTCCGTGCGATCATCGGAGATGAGA
AGTATGTGGACTACATGAGTTCACTAAAGAGATATGAAGACACAACTTTGGTTGAGGACA
CAGTACTGTAGATATTTAATCAATTGTAAATAGACAATATAAGTATGCATAAAAGTGTAGTTTTAT
AGTAGTATTTAGTGGTGTTAGTGTAAATAGTTAAGAAAATTTTGAGGAGAAAGTCAGGCCGG
GAAGTTCCCGCCACCGGAAGTTGAGTAGACGGTGCTGCCTGCGACTCAACCCCAGGAGGA
CTGGGTGAACAAAGCCGCGAAGTGATCCATGTAAGCCCTCAGAACCGTCTCGGAAGGAGG
ACCCCACATGTTGTAACTTCAAAGCCCAATGTCAGACCACGCTACGGCGTGCTACTCTGCG
GAGAGTGCAGTCTGCGATAGTGCCCCAGGAGGACTGGGTTAACAAAGGCAAACCAACGCC
CCACGCGGCCCTAGCCCCGGTAATGGTGTTAACCAGGGCGAAAGGACTAGAGGTTAGAGG
AGACCCCGCGGTTTAAAGTGCACGGCCCAGCCTGGCTGAAGCTGTAGGTCAGGGGAAGG
ACTAGAGGTTAGTGGAGACCCCGTGCCACAAAACACCACAACAAAACAGCATATTGACACC
TGGGATAGACTAGGAGATCTTCTGCTCTGCACAACCAGCCACACGGCACAGTGCGCCGAC
AATGGTGGCTGGTGGTGCGAGAACACAGGATCT

File S1 WNV reference sequences

> E-MAX/NS5-MAX

E sequences are in green.
NS5 sequences are in blue.

AGTAGTTCGCCTGTGTGAGCTGACAAACTTAGTAGTGTTTGTGAGGATTAACAACAATTAAC
ACAGTGCGAGCTGTTTCTTAGCACGAAGATCTCGATGTCTAAGAAACCAGGAGGGCCCGGC
AAGAGCCGGGCTGTCAATATGCTAAAACGCGGAATGCCCCGCGTGTTGTCCTTGATTGGAC
TGAAGAGGGCTATGTTGAGCCTGATCGACGGCAAGGGGCCAATACGATTTGTGTTGGCTCT
CTTGGCGTTCTTCAGGTTCACAGCAATTGCTCCGACCCGAGCAGTGCTGGATCGATGGAGA
GGTGTGAACAAACAAACAGCGATGAAACACCTTCTGAGTTTTAAGAAGGAACTAGGGACCT
TGACCAGTGCTATCAATCGGCGGAGCTCAAAACAAAAGAAAAGAGGAGGAAAGACCGGAAT
TGCAGTCATGATTGGCCTGATCGCCAGCGTAGGAGCAGTTACCCTCTCTAACTTCCAAGGG
AAGGTGATGATGACGGTAAATGCTACTGACGTCACAGATGTCATCACGATTCCAACAGCTGC
TGGAAAGAACCTATGCATTGTCAGAGCAATGGATGTGGGATACATGTGCGATGATACTATCA
CTTATGAATGCCCAGTACTGTCGGCTGGTAATGATCCAGAAGACATCGACTGTTGGTGCACA
AAGTCAGCAGTCTACGTCAGGTATGGAAGATGCACCAAGACACGCCACTCAAGACGCAGTC
GGAGGTCACTGACAGTGCAGACACACGGAGAAAGCACTCTAGCGAACAAGAAGGGGGCTT
GGATGGACAGCACCAAGGCCACAAGGTATTTGGTAAAAACAGAATCATGGATCTTGAGGAA
CCCTGGATATGCCCTGGTGGCAGCCGTCATTGGTTGGATGCTTGGGAGCAACACCATGCA
GAGAGTTGTGTTTGTCGTGCTATTGCTTTTGGTGGCCCCAGCTTACAGC**TTCAACTGCCTT**
GGAATGAGCAACAGAGACTTTTTGGAAGGCGTTTCCGGCGCGACGTGGGTCGATTTAGTT
CTCGAAGGGGATAGTTGCGTTACGATAATGTCGAAAGACAAACCGACGATCGACGTTAAA
ATGATGAATATGGAGGCCGCGAATCTAGCGGAAGTTCGCAGTTATTGTTATTTAGCAACCG
TAAGCGATCTTTCGACGAAAGCCGCGTGTCCGACGATGGGCGAAGCGCACAACGATAAA
CGGGCGGATCCCGCGTTTGTTTGTCGACAAGGCGTCGTCGATCGCGGTTGGGGAAATGG
TTGTGGACTATTCGGCAAAGGAAGTATCGATACGTGCGCGAAATTTGCGTGTTCTACTAAA
GCGATAGGACGAACGATATTAAAAGAAAATATCAAATACGAAGTCGCGATTTTTGTCCACG
GACCGACGACGGTCGAATCGCACGGAAATTATTCGACGCAAGTCGGCGCGACGCAGGC
CGGTCGACTAAGTATAACGCCCGCGGCGCCGTCGTACACGCTAAAACTGGGCGAATATG
GCGAGGTTACGGTCGACTGCGAACCGCGTTCGGGTATCGATACGAACGCGTATTATGTTAT
GACGGTCGGAACGAAAACGTTTTTAGTACATCGGGAATGGTTCATGGATCTAAACCTACC
GTGGAGCAGTGCCGGAAGTACCGTTTGGCGTAACCGGGAAACGTTAATGGAATTCGAAG
AACCGCACGCGACTAAACAGTCCGTAATCGCGTTAGGATCGCAAGAAGGCGCGCTTCAT
CAAGCGTTAGCCGGCGCGATTCCGGTCGAATTTTCGAGTAATACCGTCAAATTAACATCCG
GTCATTTGAAATGTCGGGTCAAAATGGAAAAATTACAATTAAAAGGAACGACTTACGGCG
TATGTTCGAAAGCGTTCAAGTTTCTCGGTACACCCGCCGATACCGGTCACGGAACCGTCG
TTTTGGAATTACAATATACCGGAACCGATGGACCGTGTAAAGTTCCGATATCGTCCGTGGC
GTCGTTGAACGATCTAACACCCGTCGGCCGATTAGTCACCGTTAATCCGTTCGTTTCCGTC
GCGACGGCGAACGCGAAAGTACTTATAGAATTGGAACCGCCGTTTGGCGATTCGTATATC
GTCGTCGGCCGCGGGGAACAACAAATAAATCACCATTGGCATAAGTCCGGAAGTAGTATC
GGAAAAGCGTTTACGACGACGCTAAAAGGCGCGCAACGACTAGCCGCGCTCGGCGATAC
CGCGTGGGATTTCGGATCCGTCGGGGGCGTTTTTACGTCCGTCGGTAAAGCCGTACATCA
AGTATTCGGCGGGGCGTTTCGCTCGCTTTTCGGCGGAATGTCGTGGATAACGCAAGGATT
ACTGGGCGCGCTACTTTTATGGATGGGAATAAACGCGCGGGATCGTTCGATCGCGCTAAC
GTTTCTCGCCGTCGGCGGAGTTCTGCTCTTCCTCTCCGTGAACGTGCACGCTGACACTGG
GTGTGCCATAGACATCAGCCGGCAAGAGCTGAGATGTGGAAGTGGAGTGTTCATACACAAT

File S1 WNV reference sequences

GATGTGGAGGCTTGGATGGACCGGTACAAGTATTACCCTGAAACGCCACAAGGCCTAGCCA
AGATCATTCAGAAAGCTCATAAGGAAGGAGTGTGCGGTCTACGATCAGTTTCCAGACTGGA
GCATCAAATGTGGGAAGCAGTGAAGGACGAGCTGAACACTCTTTTGAAGGAGAATGGTGTG
GACCTTAGTGTCGTGGTTGAGAAACAGGAGGGAATGTACAAGTCAGCACCTAAACGCCTCA
CCGCCACCACGGAAAAATTGGAAATTGGCTGGAAGGCCTGGGGAAAGAGTATTTTATTTGC
ACCAGAACTCGCCAACAACACCTTTGTGGTTGATGGTCCGGAGACCAAGGAATGTCCGACT
CAGAATCGCGCTTGGAATAGCTTAGAAGTGGAGGATTTTGGATTTGGTCTCACCAGCACTC
GGATGTTCCTGAAGGTCAGAGAGAGCAACACAACTGAATGTGACTCGAAGATCATTGGAAC
GGCTGTCAAGAACAACTTGGCGATCCACAGTGACCTGTCCTATTGGATTGAAAGCAGGCTC
AATGATACGTGGAAGCTTGAAAGGGCAGTTCTGGGTGAAGTCAAATCATGTACGTGGCCTG
AGACGCATACCTTGTGGGGCGATGGAATCCTTGAGAGTGACTTGATAATACCAGTCACACTG
GCGGGACCACGAAGCAATCACAATCGGAGACCTGGGTACAAGACACAAAACCAGGGCCCA
TGGGACGAAGGCCGGGTAGAGATTGACTTCGATTACTGCCCAGGAACTACGGTCACCCTG
AGTGAGAGCTGCGGACACCGTGGACCTGCCACTCGCACCACCACAGAGAGCGGAAAGTT
GATAACAGATTGGTGCTGCAGGAGCTGCACCTTACCACCACTGCGCTACCAAACTGACAGC
GGCTGTTGGTATGGTATGGAGATCAGACCACAGAGACATGATGAAAAGACCCTCGTGCAGT
CACAAGTGAATGCTTATAATGCTGATATGATTGACCCTTTTCAGTTGGGCCTTCTGGTCGTGT
TCTTGGCCACCCAGGAGGTCCTTCGCAAGAGGTGGACAGCCAAGATCAGCATGCCAGCTA
TACTGATTGCTCTGCTAGTCCTGGTGTTTGGGGGCATTACTTACACTGATGTGTTACGCTATG
TCATCTTGGTGGGGGCAGCTTTCGCAGAATCTAATTCGGGAGGAGACGTGGTACACTTGGC
GCTCATGGCGACCTTCAAGATACAACCAGTGTTTATGGTGGCATCGTTTCTCAAAGCGAGAT
GGACCAACCAGGAGAACATTTTGTTGATGTTGGCGGCTGTTTTCTTTCAAATGGCTTATCAC
GATGCCCGCCAAATTCTGCTCTGGGAGATCCCTGATGTGTTGAATTCACTGGCGGTAGCTT
GGATGATACTGAGAGCCATAACATTCACAACGACATCAAACGTGGTTGTTCCGCTGCTAGCC
CTGCTAACACCCGGGCTGAGATGCTTGAATCTGGATGTGTACAGGATACTGCTGTTGATGGT
CGGAATAGGCAGCTTGATCAGGGAGAAGAGGAGTGCAGCCGCAAAAAAGAAAGGAGCAAG
TCTGCTATGCTTGGCTCTAGCCTCAACAGGACTTTTCAACCCCATGATCCTTGCTGCTGGAC
TGATTGCATGTGATCCCAACCGTAAACGCGGATGGCCCGCAACTGAAGTGATGACAGCTGT
CGGCCTAATGTTTGCCATCGTCGGAGGGCTGGCAGAGCTTGACATTGACTCCATGGCCATT
CCAATGACTATCGCGGGGCTCATGTTTGCTGCTTTCGTGATTTCTGGGAAATCAACAGATAT
GTGGATTGAGAGAACGGCGGACATTTCCTGGGAAAGTGATGCAGAAATTACAGGCTCGAGC
GAAAGAGTTGATGTGCGGCTTGATGATGATGGAAACTTCCAGCTCATGAATGATCCAGGAG
CACCTTGGAAGATATGGATGCTCAGAATGGTCTGTCTCGCGATTAGTGCGTACACCCCCTG
GGCAATCTTGCCCTCAGTAGTTGGATTTTGGATAACTCTCCAATACACAAAGAGAGGAGGCG
TGTTGTGGGACACTCCCTCACCAAAGGAGTACAAAAAGGGGGACACGACCACCGGCGTCT
ACAGGATCATGACTCGTGGGCTGCTCGGCAGTTATCAAGCAGGAGCGGGCGTGATGGTTG
AAGGTGTTTTCCACACCCTTTGGCATACAACAAAAGGAGCCGCTTTGATGAGCGGAGAGGG
CCGCCTGGACCCATACTGGGGCAGTGTCAAGGAGGATCGACTTTGTTACGGAGGACCCTG
GAAATTGCAGCACAAGTGGAACGGGCAGGATGAGGTGCAGATGATTGTGGTGGAACCTGG
CAAGAACGTTAAGAACGTCCAGACGAAACCAGGGGTGTTCAAAACACCTGAAGGAGAAATC
GGGGCCGTGACTTTGGACTTCCCCACTGGAACATCAGGCTCACCAATAGTGGACAAAAAC
GGTGATGTGATTGGGCTTTATGGCAATGGAGTCATAATGCCCAACGGCTCATACATAAGCGC
GATAGTGCAGGGTGAAAGGATGGATGAGCCAATCCCAGCCGGATTCGAACCTGAGATGCTG
AGGAAAAAACAGATCACTGTACTGGATCTCCATCCCGGCGCCGGTAAAACAAGGAGGATTC
TGCCACAGATCATCAAAGAGGCCATAAACAGAAGACTGAGAACAGCCGTGCTAGCGCCAAC
CAGGGTTGTGGCTGCTGAGATGGCTGAAGCACTGAGAGGACTGCCCATCCGGTACCAGAC
ATCCGCAGTGCCCAGAGAACATAATGGAAATGAGATTGTTGATGTCATGTGTCATGCTACCC

File S1 WNV reference sequences

TCACCCACAGGCTGATGTCTCCTCACAGGGTGCCGAACTACAACCTGTTCGTGATGGATGA
GGCTCATTTCACCGACCCAGCTAGCATTGCAGCAAGAGGTTACATTTCCACAAAGGTCGAG
CTAGGGGAGGCGGCGGCAATATTCATGACAGCCACCCCACCAGGCACTTCAGATCCATTCC
CAGAGTCCAATTCACCAATTTCCGACTTACAGACTGAGATCCCGGATCGAGCTTGGAACTCT
GGATACGAATGGATCACAGAATACACCGGGAAGACGGTTTGGTTTGTGCCTAGTGTCAAGA
TGGGGAATGAGATTGCCCTTTGCCTACAACGTGCTGGAAAGAAAGTAGTCCAATTGAACAG
AAAGTCGTACGAGACGGAGTACCCAAAATGTAAGAACGATGATTGGGACTTTGTTATCACAA
CAGACATATCTGAAATGGGGGCTAACTTCAAGGCGAGCAGGGTGATTGACAGCCGGAAGA
GTGTGAAACCAACCATCATAACAGAAGGAGAAGGGAGAGTGATCCTGGGAGAACCATCTGC
AGTGACAGCAGCTAGTGCCGCCCAGAGACGTGGACGTATCGGTAGAAATCCGTCGCAAGT
TGGTGATGAGTACTGTTATGGGGGGCACACGAATGAAGACGACTCGAACTTCGCCCATTGG
ACTGAGGCACGAATCATGCTGGACAACATCAACATGCCAAACGGACTGATCGCTCAATTCTA
CCAACCAGAGCGTGAGAAGGTATATACCATGGATGGGGAATACCGGCTCAGAGGAGAAGAG
AGAAAAAACTTTCTGGAACTGTTGAGGACTGCAGATCTGCCAGTTTGGCTGGCTTACAAGG
TTGCAGCGGCTGGAGTGTCATACCACGACCGGAGGTGGTGCTTTGATGGTCCTAGGACAA
ACACAATTTTAGAAGACAACAACGAAGTGGAAGTCATCACGAAGCTTGGTGAAAGGAAGATT
CTGAGGCCGCGCTGGATTGATGCCAGGGTGTACTCGGATCACCAGGCACTAAAGGCGTTC
AAGGACTTCGCCTCGGGAAAACGTTCTCAGATAGGGCTCATTGAGGTTCTGGGAAAGATGC
CTGAGCACTTCATGGGGAAGACATGGGAAGCACTTGACACCATGTACGTTGTGGCCACTGC
AGAGAAAGGAGGAAGAGCTCACAGAATGGCCCTGGAGGAACTGCCAGATGCTCTTCAGAC
AATTGCCTTGATTGCCTTATTGAGTGTGATGACCATGGGAGTATTCTTCCTCCTCATGCAGC
GGAAGGGCATTGGAAAGATAGGTTTGGGAGGCGCTGTCTTGGGAGTCGCGACCTTTTTCT
GTTGGATGGCTGAAGTTCCAGGAACGAAGATCGCCGGAATGTTGCTGCTCTCCCTTCTCTT
GATGATTGTGCTAATTCCTGAGCCAGAGAAGCAACGTTCGCAGACAGACAACCAGCTAGCC
GTGTTCCTGATTTGTGTCATGACCCTTGTGAGCGCAGTGGCAGCCAACGAGATGGGTTGG
CTAGATAAGACCAAGAGTGACATAAGCAGTTTGTTTGGGCAAAGAATTGAGGTCAAGGAGA
ATTTCAGCATGGGAGAGTTTCTTCTGGACTTGAGGCCGGCAACAGCCTGGTCACTGTACGC
TGTGACAACAGCGGTCCTCACTCCACTGCTAAAGCATTTGATCACGTCAGATTACATCAACA
CCTCATTGACCTCAATAAACGTTCAGGCAAGTGCACTATTCACACTCGCGCGAGGCTTCCC
CTTCGTCGATGTTGGAGTGTCGGCTCTCCTGCTAGCAGCCGGATGCTGGGGACAAGTCAC
CCTCACCGTTACGGTAACAGCGGCAACACTCCTTTTTTGCCACTATGCCTACATGGTTCCCG
GTTGGCAAGCTGAGGCAATGCGCTCAGCCCAGCGGCGGACAGCGGCCGGAATCATGAAG
AACGCTGTAGTGGATGGCATCGTGGCCACGGACGTCCCAGAATTAGAGCGCACCACACCC
ATCATGCAGAAGAAAGTTGGACAGATCATGCTGATCTTGGTGTCTCTAGCTGCAGTAGTAGT
GAACCCGTCTGTGAAGACAGTACGAGAAGCCGGAATTTTGATCACGGCCGCAGCGGTGAC
GCTTTGGGAGAATGGAGCAAGCTCTGTTTGGAACGCAACAACTGCCATCGGACTCTGCCA
CATCATGCGTGGGGGTTGGTTGTCATGTCTATCCATAACATGGACACTCATAAAGAACATGG
AAAAACCAGGACTAAAAAGA**GGTGGGGCAAAAGGACGCACCTTGGGAGAGGTTTGGAAA**
GAAAGACTCAACCAGATGACAAAAGAAGAGTTCACTAGGTACCGTAAAGAAGCGATAATC
GAAGTCGATCGATCGGCGGCGAAACACGCGCGAAAAGAAGGTAACGTTACGGGCGGTC
ATCCCGTATCGCGCGGTACGGCGAAACTTCGATGGCTCGTCGAACGGCGGTTTCTCGAAC
CGGTCGGAAAAGTGATCGATCTCGGATGCGGACGCGGCGGTTGGTGTTATTATATGGCGA
CACAAAAACGCGTACAAGAAGTACGTGGGTATACTAAAGGCGGTCCGGGACACGAAGAA
CCGCAACTCGTACAAAGTTACGGATGGAATATCGTTACTATGAAGAGCGGCGTCGACGTT
TTTTATCGACCCTCGGAATGTTGCGATACGCTACTTTGCGATATCGGCGAGTCGTCCTCAA
GCGCGGAAGTCGAAGAACATCGTACGATTCGCGTACTCGAAATGGTCGAAGACTGGCTG
CATCGCGGTCCGCGCGAATTTTGCGTGAAAGTGCTATGTCCGTACATGCCGAAAGTAATC

File S1 WNV reference sequences

**GAAAAGATGGAACTGCTACAACGTCGTTACGGCGGCGGACTAGTACGAAATCCGCTATCC
CGGAATTCGACGCACGAGATGTATTGGGTGAGTCGCGCGTCGGGTAACGTCGTACATTCG
GTTAATATGACGAGCCAAGTTCTACTCGGACGAATGGAAAAACGTACGTGGAAGGGACC
GCAATACGAAGAAGACGTAAATTTAGGAAGCGGAACCCGCGCGGTCGGAAAACCGCTTC
TAAATTCCGACACGAGTAAAATAAAAAACCGAATCGAACGACTCCGACGCGAATATAGTT
CGACCTGGCATCACGACGAGAATCATCCGTATCGAACCTGGAATTATCACGGTAGTTACG
ACGTGAAGCCGACGGGATCGGCCAGTTCGCTCGTAAACGGCGTAGTTCGGCTTCTATCTA
AACCGTGGGACACGATTACGAACGTTACGACGATGGCCATGACGGACACGACTCCGTTC
GGTCAGCAACGCGTTTTTAAAGAGAAAGTCGATACGAAAGCGCCGGAACCGCCGGAAG
GCGTGAAGTACGTGCTAAACGAAACGACCAATTGGTTATGGGCGTTTTTAGCACGCGAAA
AACGTCCTCGAATGTGTTCGCGCGAAGAATTTATACGAAAAGTAAATAGTAACGCGGCGT
TAGGCGCGATGTTCGAAGAGCAAAATCAATGGCGGAGCGCGCGCGAAGCGGTCGAAGA
TCCCAAATTTTGGGAGATGGTAGACGAGGAGCGCGAAGCGCATCTGCGCGGCGAATGTC
ATACGTGCATTTATAATATGATGGGAAAGCGCGAGAAAAAACCCGGCGAATTCGGAAAAG
CAAAAGGAAGCCGCGCGATTTGGTTTATGTGGCTCGGCGCTCGATTTCTCGAATTCGAGG
CGCTCGGTTTTCTAAACGAAGATCATTGGCTCGGACGAAAAAATTCCGGCGGCGGCGTC
GAGGGATTGGGTCTACAAAAACTCGGTTATATTCTTCGCGAAGTCGGAACGCGGCCGGG
CGGAAAGATATACGCGGACGACACGGCGGGATGGGATACGCGAATAACGCGCGCGGATT
TGGAAAACGAAGCGAAAGTGCTCGAGCTGCTTGACGGCGAACATCGTCGTCTCGCGCGA
GCCATAATCGAGCTAACGTATCGTCATAAAGTCGTTAAAGTGATGCGGCCGGCGGCGGAC
GGACGAACGGTAATGGACGTTATATCGCGAGAAGATCAGCGTGGTAGCGGACAAGTCGT
AACATACGCGCTAAATACGTTTACCAATCTAGCCGTACAACTCGTTCGAATGATGGAAGGC
GAAGGCGTTATCGGTCCGGACGACGTCGAGAAACTAACGAAAGGTAAAGGACCGAAAGT
CCGGACGTGGCTGTTCGAGAATGGCGAAGAACGACTAAGTCGAATGGCGGTAAGCGGC
GACGATTGCGTCGTAAAACCGCTCGACGATCGATTCGCGACCTCGCTTCATTTCCTAAAC
GCGATGTCGAAGGTTCGAAAAGATATACAAGAATGGAAACCCTCGACGGGATGGTACGAT
TGGCAACAGGTTCCGTTTTGCTCGAATCATTTTACCGAATTAATTATGAAAGACGGACGAA
CGCTAGTTGTTCCTTGTCGCGGACAAGACGAATTAGTCGGGCGCGCGCGAATATCGCCG
GGCGCCGGATGGAATGTACGCGACACGGCGTGTCTCGCGAAATCGTACGCGCAGATGTG
GCTGCTTCTATATTTTCATCGACGCGATCTTCGACTAATGGCGAACGCGATTTGTTCGGCG
GTACCGGTAAATTGGGTACCAACGGGACGAACGACGTGGTCGATACACGCGGGCGGCGA
GTGGATGACAACGGAAGATATGTTGGAAGTATGGAACCGCGTTTGGATCGAAGAGAACG
AATGGATGGAAGATAAAACGCCGGTCGAGAAATGGAGCGATGTTCCGTATTCGGGAAAA
CGCGAGGATATATGGTGCGGTAGTCTGATCGGAACGCGCGCGCGCCACGTGGGCGGA
AAATATACAAGTCGCGATAAACCAAGTACGCGCGATAATCGGAGATGAGAAGTATGTGGA
CTACATGAGTTCACTAAAGAGATATGAAGACACAACTTTGGTTGAGGACACAGTACTG**TAG
ATATTTAATCAATTGTAAATAGACAATATAAGTATGCATAAAAGTGTAGTTTTATAGTAGTATTTA
GTGGTGTTAGTGTAAATAGTTAAGAAAATTTTGAGGAGAAAGTCAGGCCGGGAAGTTCCCG
CCACCGGAAGTTGAGTAGACGGTGCTGCCTGCGACTCAACCCCAGGAGGACTGGGTGAA
CAAAGCCGCGAAGTGATCCATGTAAGCCCTCAGAACCGTCTCGGAAGGAGGACCCCACAT
GTTGTAACTTCAAAGCCCAATGTCAGACCACGCTACGGCGTGCTACTCTGCGGAGAGTGCA
GTCTGCGATAGTGCCCCAGGAGGACTGGGTTAACAAAGGCAAACCAACGCCCCACGCGGC
CCTAGCCCCGGTAATGGTGTTAACCAGGGCGAAAGGACTAGAGGTTAGAGGAGACCCCGC
GGTTTAAAGTGCACGGCCCAGCCTGGCTGAAGCTGTAGGTCAGGGGAAGGACTAGAGGTT
AGTGGAGACCCCGTGCCACAAAACACCACAACAAAACAGCATATTGACACCTGGGATAGAC
TAGGAGATCTTCTGCTCTGCACAACCAGCCACACGGCACAGTGCGCCGACAATGGTGGCT
GGTGGTGCGAGAACACAGGATCT

File S1 WNV reference sequences

> WNV-WT+FVR

DQ211652.1 West Nile virus strain NY99. Nucleotide substitutions to introduce L107F, A316V, and K440R mutations are highlighted.

AGTAGTTCGCCTGTGTGAGCTGACAAACTTAGTAGTGTTTGTGAGGATTAACAACAATTAAC
ACAGTGCGAGCTGTTTCTTAGCACGAAGATCTCGATGTCTAAGAAACCAGGAGGGCCCGGC
AAGAGCCGGGCTGTCAATATGCTAAAACGCGGAATGCCCCGCGTGTTGTCCTTGATTGGAC
TGAAGAGGGCTATGTTGAGCCTGATCGACGGCAAGGGGCCAATACGATTTGTGTTGGCTCT
CTTGGCGTTCTTCAGGTTCACAGCAATTGCTCCGACCCGAGCAGTGCTGGATCGATGGAGA
GGTGTGAACAAACAAACAGCGATGAAACACCTTCTGAGTTTTAAGAAGGAACTAGGGACCT
TGACCAGTGCTATCAATCGGCGGAGCTCAAAACAAAAGAAAAGAGGAGGAAAGACCGGAAT
TGCAGTCATGATTGGCCTGATCGCCAGCGTAGGAGCAGTTACCCTCTCTAACTTCCAAGGG
AAGGTGATGATGACGGTAAATGCTACTGACGTCACAGATGTCATCACGATTCCAACAGCTGC
TGGAAAGAACCTATGCATTGTCAGAGCAATGGATGTGGGATACATGTGCGATGATACTATCA
CTTATGAATGCCCAGTACTGTCGGCTGGTAATGATCCAGAAGACATCGACTGTTGGTGCACA
AAGTCAGCAGTCTACGTCAGGTATGGAAGATGCACCAAGACACGCCACTCAAGACGCAGTC
GGAGGTCACTGACAGTGCAGACACACGGAGAAAGCACTCTAGCGAACAAGAAGGGGGCTT
GGATGGACAGCACCAAGGCCACAAGGTATTTGGTAAAAACAGAATCATGGATCTTGAGGAA
CCCTGGATATGCCCTGGTGGCAGCCGTCATTGGTTGGATGCTTGGGAGCAACACCATGCA
GAGAGTTGTGTTTGTCGTGCTATTGCTTTTGGTGGCCCCAGCTTACAGCTTCAACTGCCTTG
GAATGAGCAACAGAGACTTCTTGGAAGGAGTGTCTGGAGCAACATGGGTGGATTTGGTTCT
CGAAGGCGACAGCTGCGTGACTATCATGTCTAAGGACAAGCCTACCATCGATGTGAAGATG
ATGAATATGGAGGCGGCCAACCTGGCAGAGGTCCGCAGTTATTGCTATTTGGCTACCGTCA
GCGATCTCTCCACCAAAGCTGCGTGCCCGACCATGGGAGAAGCTCACAATGACAAACGTG
CTGACCCAGCTTTTGTGTGCAGACAAGGAGTGGTGGACAGGGGCTGGGGCAACGGCTGC
GGA**T**T**T**TTTGGCAAAGGAAGCATTGACACATGCGCCAAATTTGCCTGCTCTACCAAGGCAAT
AGGAAGAACCATCTTGAAAGAGAATATCAAGTACGAAGTGGCCATTTTTGTCCATGGACCAA
CTACTGTGGAGTCGCACGGAAACTACTCCACACAGGTTGGAGCCACTCAGGCAGGGAGAC
TCAGCATCACTCCTGCGGCGCCTTCATACACACTAAAGCTTGGAGAATATGGAGAGGTGAC
AGTGGACTGTGAACCACGGTCAGGGATTGACACCAATGCATACTACGTGATGACTGTTGGA
ACAAAGACGTTCTTGGTCCATCGTGAGTGGTTCATGGACCTCAACCTCCCTTGGAGCAGTG
CTGGAAGTACTGTGTGGAGGAACAGAGAGACGTTAATGGAGTTTGAGGAACCACACGCCA
CGAAGCAGTCTGTGATAGCATTGGGCTCACAAGAGGGAGCTCTGCATCAAGCTTTGGCTGG
AGCCATTCCTGTGGAATTTTCAAGCAACACTGTCAAGTTGACGTCGGGTCATTTGAAGTGTA
GAGTGAAGATGGAAAAATTGCAGTTGAAGGGAACAACCTATGGCGTCTGTTCAAAGGCTTT
CAAGTTTCTTGGGACTCCCGTGGACACAGGTCACGGCACTGTGGTGTTGGAATTGCAGTAC
ACTGGCACGGATGGACCTTGCAAAGTTCCTATCTCGTCAGTGGCTTCATTGAACGACCTAA
CGCCAGTGGGCAGATTGGTCACTGTCAACCCTTTTGTTTCAGTGGCCACGGCCAACGCTAA
GGTCCTGATTGAATTGGAACCACCCTTTGGAGACTCATACATAGTGGTGGGCAGAGGAGAA
CAACAGATCAATCACCATTGGCACAAGTCTGGAAGCAGCATTGGCAAAGCCTTTACAACCA
CCCTCAAAGGAGCGCAGAGACTAGCCGCTCTAGGAGACACAGCTTGGGACTTTGGATCAG
TTGGAGGGGTGTTCACCTCAGTTGGGCGGGCTGTCCATCAAGTGTTCGGAGGAGCATTCC
GCTCACTGTTCGGAGGCATGTCCTGGATAACGCAAGGATTGCTGGGGGCTCTCCTGTTGTG
GATGGGCATCAATGCTCGTGATAGGTCCATAGCTCTCACGTTTCTCGCAGTTGGAGGAGTT
CTGCTCTTCCTCTCCGTGAACGTGCACGCTGACACTGGGTGTGCCATAGACATCAGCCGG
CAAGAGCTGAGATGTGGAAGTGGAGTGTTCATACACAATGATGTGGAGGCTTGGATGGACC
GGTACAAGTATTACCCTGAAACGCCACAAGGCCTAGCCAAGATCATTCAGAAAGCTCATAAG

File S1 WNV reference sequences

GAAGGAGTGTGCGGTCTACGATCAGTTTCCAGACTGGAGCATCAAATGTGGGAAGCAGTGA
AGGACGAGCTGAACACTCTTTTGAAGGAGAATGGTGTGGACCTTAGTGTCGTGGTTGAGAA
ACAGGAGGGAATGTACAAGTCAGCACCTAAACGCCTCACCGCCACCACGGAAAAATTGGAA
ATTGGCTGGAAGGCCTGGGGAAAGAGTATTTTATTTGCACCAGAACTCGCCAACAACACCT
TTGTGGTTGATGGTCCGGAGACCAAGGAATGTCCGACTCAGAATCGCGCTTGGAATAGCTT
AGAAGTGGAGGATTTTGGATTTGGTCTCACCAGCACTCGGATGTTCCTGAAGGTCAGAGAG
AGCAACACAACTGAATGTGACTCGAAGATCATTGGAACGGCTGTCAAGAACAACTTGGCGA
TCCACAGTGACCTGTCCTATTGGATTGAAAGCAGGCTCAATGATACGTGGAAGCTTGAAAG
GGCAGTTCTGGGTGAAGTCAAATCATGTACGTGGCCTGAGACGCATACCTTGTGGGGCGAT
GGAATCCTTGAGAGTGACTTGATAATACCAGTCACACTGGCGGGACCACGAAGCAATCACA
ATCGGAGACCTGGGTACAAGACACAAAACCAGGGCCCATGGGACGAAGGCCGGGTAGAGA
TTGACTTCGATTACTGCCCAGGAACTACGGTCACCCTGAGTGAGAGCTGCGGACACCGTG
GACCTGCCACTCGCACCACCACAGAGAGCGGAAAGTTGATAACAGATTGGTGCTGCAGGA
GCTGCACCTTACCACCACTGCGCTACCAAACTGACAGCGGCTGTTGGTATGGTATGGAGAT
CAGACCACAGAGACATGATGAAAAGACCCTCGTGCAGTCACAAGTGAATGCTTATAATGCTG
ATATGATTGACCCTTTTCAGTTGGGCCTTCTGGTCGTGTTCTTGGCCACCCAGGAGGTCCTT
CGCAAGAGGTGGACAGCCAAGATCAGCATGCCAGCTATACTGATTGCTCTGCTAGTCCTGG
TGTTTGGGGGCATTACTTACACTGATGTGTTACGCTATGTCATCTTGGTGGGGGCAGCTTTC
GCAGAATCTAATTCGGGAGGAGACGTGGTACACTTGGCGCTCATGGCGACCTTCAAGATAC
AACCAGTGTTTATGGTGGCATCGTTTCTCAAAGCGAGATGGACCAACCAGGAGAACATTTTG
TTGATGTTGGCGGCTGTTTTCTTTCAAATGGCTTATCACGATGCCCGCCAAATTCTGCTCTG
GGAGATCCCTGATGTGTTGAATTCACTGGCGGTAGCTTGGATGATACTGAGAGCCATAACAT
TCACAACGACATCAAACGTGGTTGTTCCGCTGCTAGCCCTGCTAACACCCGGGCTGAGATG
CTTGAATCTGGATGTGTACAGGATACTGCTGTTGATGGTCGGAATAGGCAGCTTGATCAGGG
AGAAGAGGAGTGCAGCCGCAAAAAAGAAAGGAGCAAGTCTGCTATGCTTGGCTCTAGCCT
CAACAGGACTTTTCAACCCCATGATCCTTGCTGCTGGACTGATTGCATGTGATCCCAACCGT
AAACGCGGATGGCCCGCAACTGAAGTGATGACAGCTGTCGGCCTAATGTTTGCCATCGTCG
GAGGGCTGGCAGAGCTTGACATTGACTCCATGGCCATTCCAATGACTATCGCGGGGCTCAT
GTTTGCTGCTTTCGTGATTTCTGGGAAATCAACAGATATGTGGATTGAGAGAACGGCGGACA
TTTCCTGGGAAAGTGATGCAGAAATTACAGGCTCGAGCGAAAGAGTTGATGTGCGGCTTGA
TGATGATGGAAACTTCCAGCTCATGAATGATCCAGGAGCACCTTGGAAGATATGGATGCTCA
GAATGGTCTGTCTCGCGATTAGTGCGTACACCCCCTGGGCAATCTTGCCCTCAGTAGTTGG
ATTTTGGATAACTCTCCAATACACAAAGAGAGGAGGCGTGTTGTGGGACACTCCCTCACCA
AAGGAGTACAAAAAGGGGGACACGACCACCGGCGTCTACAGGATCATGACTCGTGGGCTG
CTCGGCAGTTATCAAGCAGGAGCGGGCGTGATGGTTGAAGGTGTTTTCCACACCCTTTGG
CATACAACAAAAGGAGCCGCTTTGATGAGCGGAGAGGGCCGCCTGGACCCATACTGGGGC
AGTGTCAAGGAGGATCGACTTTGTTACGGAGGACCCTGGAAATTGCAGCACAAGTGGAAC
GGGCAGGATGAGGTGCAGATGATTGTGGTGGAACCTGGCAAGAACGTTAAGAACGTCCAG
ACGAAACCAGGGGTGTTCAAAACACCTGAAGGAGAAATCGGGGCCGTGACTTTGGACTTC
CCCACTGGAACATCAGGCTCACCAATAGTGGACAAAAACGGTGATGTGATTGGGCTTTATG
GCAATGGAGTCATAATGCCCAACGGCTCATACATAAGCGCGATAGTGCAGGGTGAAAGGAT
GGATGAGCCAATCCCAGCCGGATTCGAACCTGAGATGCTGAGGAAAAAACAGATCACTGTA
CTGGATCTCCATCCCGGCGCCGGTAAAACAAGGAGGATTCTGCCACAGATCATCAAAGAGG
CCATAAACAGAAGACTGAGAACAGCCGTGCTAGCGCCAACCAGGGTTGTGGCTGCTGAGA
TGGCTGAAGCACTGAGAGGACTGCCCATCCGGTACCAGACATCCGCAGTGCCCAGAGAAC
ATAATGGAAATGAGATTGTTGATGTCATGTGTCATGCTACCCTCACCCACAGGCTGATGTCTC
CTCACAGGGTGCCGAACTACAACCTGTTCGTGATGGATGAGGCTCATTTCACCGACCCAGC

File S1 WNV reference sequences

TAGCATTGCAGCAAGAGGTTACATTTCCACAAAGGTCGAGCTAGGGGAGGCGGCGGCAATA
TTCATGACAGCCACCCCACCAGGCACTTCAGATCCATTCCCAGAGTCCAATTCACCAATTTC
CGACTTACAGACTGAGATCCCGGATCGAGCTTGGAACTCTGGATACGAATGGATCACAGAA
TACACCGGGAAGACGGTTTGGTTTGTGCCTAGTGTCAAGATGGGGAATGAGATTGCCCTTT
GCCTACAACGTGCTGGAAAGAAAGTAGTCCAATTGAACAGAAAGTCGTACGAGACGGAGTA
CCCAAAATGTAAGAACGATGATTGGGACTTTGTTATCACAACAGACATATCTGAAATGGGGG
CTAACTTCAAGGCGAGCAGGGTGATTGACAGCCGGAAGAGTGTGAAACCAACCATCATAAC
AGAAGGAGAAGGGAGAGTGATCCTGGGAGAACCATCTGCAGTGACAGCAGCTAGTGCCGC
CCAGAGACGTGGACGTATCGGTAGAAATCCGTCGCAAGTTGGTGATGAGTACTGTTATGGG
GGGCACACGAATGAAGACGACTCGAACTTCGCCCATTGGACTGAGGCACGAATCATGCTG
GACAACATCAACATGCCAAACGGACTGATCGCTCAATTCTACCAACCAGAGCGTGAGAAGG
TATATACCATGGATGGGGAATACCGGCTCAGAGGAGAAGAGAGAAAAAACTTTCTGGAACTG
TTGAGGACTGCAGATCTGCCAGTTTGGCTGGCTTACAAGGTTGCAGCGGCTGGAGTGTCAT
ACCACGACCGGAGGTGGTGCTTTGATGGTCCTAGGACAAACACAATTTTAGAAGACAACAA
CGAAGTGGAAGTCATCACGAAGCTTGGTGAAAGGAAGATTCTGAGGCCGCGCTGGATTGAT
GCCAGGGTGTACTCGGATCACCAGGCACTAAAGGCGTTCAAGGACTTCGCCTCGGGAAAA
CGTTCTCAGATAGGGCTCATTGAGGTTCTGGGAAAGATGCCTGAGCACTTCATGGGGAAGA
CATGGGAAGCACTTGACACCATGTACGTTGTGGCCACTGCAGAGAAAGGAGGAAGAGCTC
ACAGAATGGCCCTGGAGGAACTGCCAGATGCTCTTCAGACAATTGCCTTGATTGCCTTATTG
AGTGTGATGACCATGGGAGTATTCTTCCTCCTCATGCAGCGGAAGGGCATTGGAAAGATAG
GTTTGGGAGGCGCTGTCTTGGGAGTCGCGACCTTTTTCTGTTGGATGGCTGAAGTTCCAG
GAACGAAGATCGCCGGAATGTTGCTGCTCTCCCTTCTCTTGATGATTGTGCTAATTCCTGAG
CCAGAGAAGCAACGTTCGCAGACAGACAACCAGCTAGCCGTGTTCCTGATTTGTGTCATGA
CCCTTGTGAGCGCAGTGGCAGCCAACGAGATGGGTTGGCTAGATAAGACCAAGAGTGACA
TAAGCAGTTTGTTTGGGCAAAGAATTGAGGTCAAGGAGAATTTCAGCATGGGAGAGTTTCTT
CTGGACTTGAGGCCGGCAACAGCCTGGTCACTGTACGCTGTGACAACAGCGGTCCTCACT
CCACTGCTAAAGCATTTGATCACGTCAGATTACATCAACACCTCATTGACCTCAATAAACGTT
CAGGCAAGTGCACTATTCACACTCGCGCGAGGCTTCCCCTTCGTCGATGTTGGAGTGTCG
GCTCTCCTGCTAGCAGCCGGATGCTGGGGACAAGTCACCCTCACCGTTACGGTAACAGCG
GCAACACTCCTTTTTTGCCACTATGCCTACATGGTTCCCGGTTGGCAAGCTGAGGCAATGC
GCTCAGCCCAGCGGCGGACAGCGGCCGGAATCATGAAGAACGCTGTAGTGGATGGCATCG
TGGCCACGGACGTCCCAGAATTAGAGCGCACCACACCCATCATGCAGAAGAAAGTTGGAC
AGATCATGCTGATCTTGGTGTCTCTAGCTGCAGTAGTAGTGAACCCGTCTGTGAAGACAGTA
CGAGAAGCCGGAATTTTGATCACGGCCGCAGCGGTGACGCTTTGGGAGAATGGAGCAAGC
TCTGTTTGGAACGCAACAACTGCCATCGGACTCTGCCACATCATGCGTGGGGGTTGGTTGT
CATGTCTATCCATAACATGGACACTCATAAAGAACATGGAAAAACCAGGACTAAAAAGAGGT
GGGGCAAAAGGACGCACCTTGGGAGAGGTTTGGAAAGAAAGACTCAACCAGATGACAAAA
GAAGAGTTCACTAGGTACCGCAAAGAGGCCATCATCGAAGTCGATCGCTCAGCGGCAAAAC
ACGCCAGGAAAGAAGGCAATGTCACTGGAGGGCATCCAGTCTCTAGGGGCACAGCAAAAC
TGAGATGGCTGGTCGAACGGAGGTTTCTCGAACCGGTCGGAAAAGTGATTGACCTTGGAT
GTGGAAGAGGCGGTTGGTGTTACTATATGGCAACCCAAAAAAGAGTCCAAGAAGTCAGAGG
GTACACAAAGGGCGGTCCCGGACATGAAGAGCCCCAACTAGTGCAAAGTTATGGATGGAAC
ATTGTCACCATGAAGAGTGGAGTGGATGTGTTCTACAGACCTTCTGAGTGTTGTGACACCCT
CCTTTGTGACATCGGAGAGTCCTCGTCAAGTGCTGAGGTTGAAGAGCATAGGACGATTCGG
GTCCTTGAAATGGTTGAGGACTGGCTGCACCGAGGGCCAAGGGAATTTTGCGTGAAGGTG
CTCTGTCCCTACATGCCGAAAGTCATAGAGAAGATGGAGCTGCTCCAACGCCGGTATGGGG
GGGGACTGGTCAGAAACCCACTCTCACGGAATTCCACGCACGAGATGTATTGGGTGAGTC

File S1 WNV reference sequences

GAGCTTCAGGCAATGTGGTACATTCAGTGAATATGACCAGCCAGGTGCTCCTAGGAAGAAT
GGAAAAAAGGACCTGGAAGGGACCCCAATACGAGGAAGATGTAAACTTGGGAAGTGGAAC
CAGGGCGGTGGGAAAACCCCTGCTCAACTCAGACACCAGTAAAATCAAGAACAGGATTGAA
CGACTCAGGCGTGAGTACAGTTCGACGTGGCACCACGATGAGAACCACCCATATAGAACCT
GGAACTATCACGGCAGTTATGATGTGAAGCCCACAGGCTCCGCCAGTTCGCTGGTCAATGG
AGTGGTCAGGCTCCTCTCAAAACCATGGGACACCATCACGAATGTTACCACCATGGCCATG
ACTGACACTACTCCCTTCGGGCAGCAGCGAGTGTTCAAAGAGAAGGTGGACACGAAAGCT
CCTGAACCGCCAGAAGGAGTGAAGTACGTGCTCAACGAGACCACCAACTGGTTGTGGGCG
TTTTTGGCCAGAGAAAAACGTCCCAGAATGTGCTCTCGAGAGGAATTCATAAGAAAGGTCAA
CAGCAATGCAGCTTTGGGTGCCATGTTTGAAGAGCAGAATCAATGGAGGAGCGCCAGAGA
AGCAGTTGAAGATCCAAAATTTTGGGAGATGGTGGATGAGGAGCGCGAGGCACATCTGCG
GGGGGAATGTCACACTTGCATTTACAACATGATGGGAAAGAGAGAGAAAAAACCCGGAGAG
TTCGGAAAGGCCAAGGGAAGCAGAGCCATTTGGTTCATGTGGCTCGGAGCTCGCTTTCTG
GAGTTCGAGGCTCTGGGTTTTCTCAATGAAGACCACTGGCTTGGAAGAAAGAACTCAGGAG
GAGGTGTCGAGGGCTTGGGCCTCCAAAAACTGGGTTACATCCTGCGTGAAGTTGGCACCC
GGCCTGGGGGCAAGATCTATGCTGATGACACAGCTGGCTGGGACACCCGCATCACGAGAG
CTGACTTGGAAAATGAAGCTAAGGTGCTTGAGCTGCTTGATGGGGAACATCGGCGTCTTGC
CAGGGCCATCATTGAGCTCACCTATCGTCACAAAGTTGTGAAAGTGATGCGCCCGGCTGCT
GATGGAAGAACCGTCATGGATGTTATCTCCAGAGAAGATCAGAGGGGGAGTGGACAAGTTG
TCACCTACGCCCTAAACACTTTCACCAACCTGGCCGTCCAGCTGGTGAGGATGATGGAAGG
GGAAGGAGTGATTGGCCCAGATGATGTGGAGAAACTCACAAAAGGGAAAGGACCCAAAGT
CAGGACCTGGCTGTTTGAGAATGGGGAAGAAAGACTCAGCCGCATGGCTGTCAGTGGAGA
TGACTGTGTGGTAAAGCCCCTGGACGATCGCTTTGCCACCTCGCTCCACTTCCTCAATGCT
ATGTCAAAGGTTCGCAAAGACATCCAAGAGTGGAAACCGTCAACTGGATGGTATGATTGGC
AGCAGGTTCCATTTTGCTCAAACCATTTCACTGAATTGATCATGAAAGATGGAAGAACACTG
GTGGTTCCATGCCGAGGACAGGATGAATTGGTAGGCAGAGCTCGCATATCTCCAGGGGCC
GGATGGAACGTCCGCGACACTGCTTGTCTGGCTAAGTCTTATGCCCAGATGTGGCTGCTTC
TGTACTTCCACAGAAGAGACCTGCGGCTCATGGCCAACGCCATTTGCTCCGCTGTCCCTGT
GAATTGGGTCCCTACCGGAAGAACCACGTGGTCCATCCATGCAGGAGGAGAGTGGATGAC
AACAGAGGACATGTTGGAGGTCTGGAACCGTGTTTGGATAGAGGAGAATGAATGGATGGAA
GACAAAACCCCAGTGGAGAAATGGAGTGACGTCCCATATTCAGGAAAACGAGAGGACATCT
GGTGTGGCAGCCTGATTGGCACAAGAGCCCGAGCCACGTGGGCAGAAAACATCCAGGTG
GCTATCAACCAAGTCAGAGCAATCATCGGAGATGAGAAGTATGTGGACTACATGAGTTCACT
AAAGAGATATGAAGACACAACTTTGGTTGAGGACACAGTACTGTAGATATTTAATCAATTGTA
AATAGACAATATAAGTATGCATAAAAGTGTAGTTTTATAGTAGTATTTAGTGGTGTTAGTGTAAA
TAGTTAAGAAAATTTTGAGGAGAAAGTCAGGCCGGGAAGTTCCCGCCACCGGAAGTTGAGT
AGACGGTGCTGCCTGCGACTCAACCCCAGGAGGACTGGGTGAACAAAGCCGCGAAGTGA
TCCATGTAAGCCCTCAGAACCGTCTCGGAAGGAGGACCCCACATGTTGTAACTTCAAAGCC
CAATGTCAGACCACGCTACGGCGTGCTACTCTGCGGAGAGTGCAGTCTGCGATAGTGCCC
CAGGAGGACTGGGTTAACAAAGGCAAACCAACGCCCCACGCGGCCCTAGCCCCGGTAATG
GTGTTAACCAGGGCGAAAGGACTAGAGGTTAGAGGAGACCCCGCGGTTTAAAGTGCACGG
CCCAGCCTGGCTGAAGCTGTAGGTCAGGGGAAGGACTAGAGGTTAGTGGAGACCCCGTG
CCACAAAACACCACAACAAAACAGCATATTGACACCTGGGATAGACTAGGAGATCTTCTGCT
CTGCACAACCAGCCACACGGCACAGTGCGCCGACAATGGTGGCTGGTGGTGCGAGAACA
CAGGATCT

File S1 WNV reference sequences

> WNV-WT+FR

DQ211652.1 West Nile virus strain NY99. Nucleotide substitutions to introduce L107F and K440R mutations are highlighted.

AGTAGTTCGCCTGTGTGAGCTGACAAACTTAGTAGTGTTTGTGAGGATTAACAACAATTAAC
ACAGTGCGAGCTGTTTCTTAGCACGAAGATCTCGATGTCTAAGAAACCAGGAGGGCCCGGC
AAGAGCCGGGCTGTCAATATGCTAAAACGCGGAATGCCCCGCGTGTTGTCCTTGATTGGAC
TGAAGAGGGCTATGTTGAGCCTGATCGACGGCAAGGGGCCAATACGATTTGTGTTGGCTCT
CTTGGCGTTCTTCAGGTTCACAGCAATTGCTCCGACCCGAGCAGTGCTGGATCGATGGAGA
GGTGTGAACAAACAAACAGCGATGAAACACCTTCTGAGTTTTAAGAAGGAACTAGGGACCT
TGACCAGTGCTATCAATCGGCGGAGCTCAAAACAAAAGAAAAGAGGAGGAAAGACCGGAAT
TGCAGTCATGATTGGCCTGATCGCCAGCGTAGGAGCAGTTACCCTCTCTAACTTCCAAGGG
AAGGTGATGATGACGGTAAATGCTACTGACGTCACAGATGTCATCACGATTCCAACAGCTGC
TGGAAAGAACCTATGCATTGTCAGAGCAATGGATGTGGGATACATGTGCGATGATACTATCA
CTTATGAATGCCCAGTACTGTCGGCTGGTAATGATCCAGAAGACATCGACTGTTGGTGCACA
AAGTCAGCAGTCTACGTCAGGTATGGAAGATGCACCAAGACACGCCACTCAAGACGCAGTC
GGAGGTCACTGACAGTGCAGACACACGGAGAAAGCACTCTAGCGAACAAGAAGGGGGCTT
GGATGGACAGCACCAAGGCCACAAGGTATTTGGTAAAAACAGAATCATGGATCTTGAGGAA
CCCTGGATATGCCCTGGTGGCAGCCGTCATTGGTTGGATGCTTGGGAGCAACACCATGCA
GAGAGTTGTGTTTGTCGTGCTATTGCTTTTGGTGGCCCCAGCTTACAGCTTCAACTGCCTTG
GAATGAGCAACAGAGACTTCTTGGAAGGAGTGTCTGGAGCAACATGGGTGGATTTGGTTCT
CGAAGGCGACAGCTGCGTGACTATCATGTCTAAGGACAAGCCTACCATCGATGTGAAGATG
ATGAATATGGAGGCGGCCAACCTGGCAGAGGTCCGCAGTTATTGCTATTTGGCTACCGTCA
GCGATCTCTCCACCAAAGCTGCGTGCCCGACCATGGGAGAAGCTCACAATGACAAACGTG
CTGACCCAGCTTTTGTGTGCAGACAAGGAGTGGTGGACAGGGGCTGGGGCAACGGCTGC
GGATTTTTTGGCAAAGGAAGCATTGACACATGCGCCAAATTTGCCTGCTCTACCAAGGCAAT
AGGAAGAACCATCTTGAAAGAGAATATCAAGTACGAAGTGGCCATTTTTGTCCATGGACCAA
CTACTGTGGAGTCGCACGGAAACTACTCCACACAGGTTGGAGCCACTCAGGCAGGGAGAC
TCAGCATCACTCCTGCGGCGCCTTCATACACACTAAAGCTTGGAGAATATGGAGAGGTGAC
AGTGGACTGTGAACCACGGTCAGGGATTGACACCAATGCATACTACGTGATGACTGTTGGA
ACAAAGACGTTCTTGGTCCATCGTGAGTGGTTCATGGACCTCAACCTCCCTTGGAGCAGTG
CTGGAAGTACTGTGTGGAGGAACAGAGAGACGTTAATGGAGTTTGAGGAACCACACGCCA
CGAAGCAGTCTGTGATAGCATTGGGCTCACAAGAGGGAGCTCTGCATCAAGCTTTGGCTGG
AGCCATTCCTGTGGAATTTTCAAGCAACACTGTCAAGTTGACGTCGGGTCATTTGAAGTGTA
GAGTGAAGATGGAAAAATTGCAGTTGAAGGGAACAACCTATGGCGTCTGTTCAAAGGCTTT
CAAGTTTCTTGGGACTCCCGCAGACACAGGTCACGGCACTGTGGTGTTGGAATTGCAGTAC
ACTGGCACGGATGGACCTTGCAAAGTTCCTATCTCGTCAGTGGCTTCATTGAACGACCTAA
CGCCAGTGGGCAGATTGGTCACTGTCAACCCTTTTGTTTCAGTGGCCACGGCCAACGCTAA
GGTCCTGATTGAATTGGAACCACCCTTTGGAGACTCATACATAGTGGTGGGCAGAGGAGAA
CAACAGATCAATCACCATTGGCACAAGTCTGGAAGCAGCATTGGCAAAGCCTTTACAACCA
CCCTCAAAGGAGCGCAGAGACTAGCCGCTCTAGGAGACACAGCTTGGGACTTTGGATCAG
TTGGAGGGGTGTTCACCTCAGTTGGGCGGGCTGTCCATCAAGTGTTCGGAGGAGCATTCC
GCTCACTGTTCGGAGGCATGTCCTGGATAACGCAAGGATTGCTGGGGGCTCTCCTGTTGTG
GATGGGCATCAATGCTCGTGATAGGTCCATAGCTCTCACGTTTCTCGCAGTTGGAGGAGTT
CTGCTCTTCCTCTCCGTGAACGTGCACGCTGACACTGGGTGTGCCATAGACATCAGCCGG
CAAGAGCTGAGATGTGGAAGTGGAGTGTTCATACACAATGATGTGGAGGCTTGGATGGACC
GGTACAAGTATTACCCTGAAACGCCACAAGGCCTAGCCAAGATCATTCAGAAAGCTCATAAG

File S1 WNV reference sequences

GAAGGAGTGTGCGGTCTACGATCAGTTTCCAGACTGGAGCATCAAATGTGGGAAGCAGTGA
AGGACGAGCTGAACACTCTTTTGAAGGAGAATGGTGTGGACCTTAGTGTCGTGGTTGAGAA
ACAGGAGGGAATGTACAAGTCAGCACCTAAACGCCTCACCGCCACCACGGAAAAATTGGAA
ATTGGCTGGAAGGCCTGGGGAAAGAGTATTTTATTTGCACCAGAACTCGCCAACAACACCT
TTGTGGTTGATGGTCCGGAGACCAAGGAATGTCCGACTCAGAATCGCGCTTGGAATAGCTT
AGAAGTGGAGGATTTTGGATTTGGTCTCACCAGCACTCGGATGTTCCTGAAGGTCAGAGAG
AGCAACACAACTGAATGTGACTCGAAGATCATTGGAACGGCTGTCAAGAACAACTTGGCGA
TCCACAGTGACCTGTCCTATTGGATTGAAAGCAGGCTCAATGATACGTGGAAGCTTGAAAG
GGCAGTTCTGGGTGAAGTCAAATCATGTACGTGGCCTGAGACGCATACCTTGTGGGGCGAT
GGAATCCTTGAGAGTGACTTGATAATACCAGTCACACTGGCGGGACCACGAAGCAATCACA
ATCGGAGACCTGGGTACAAGACACAAAACCAGGGCCCATGGGACGAAGGCCGGGTAGAGA
TTGACTTCGATTACTGCCCAGGAACTACGGTCACCCTGAGTGAGAGCTGCGGACACCGTG
GACCTGCCACTCGCACCACCACAGAGAGCGGAAAGTTGATAACAGATTGGTGCTGCAGGA
GCTGCACCTTACCACCACTGCGCTACCAAACTGACAGCGGCTGTTGGTATGGTATGGAGAT
CAGACCACAGAGACATGATGAAAAGACCCTCGTGCAGTCACAAGTGAATGCTTATAATGCTG
ATATGATTGACCCTTTTCAGTTGGGCCTTCTGGTCGTGTTCTTGGCCACCCAGGAGGTCCTT
CGCAAGAGGTGGACAGCCAAGATCAGCATGCCAGCTATACTGATTGCTCTGCTAGTCCTGG
TGTTTGGGGGCATTACTTACACTGATGTGTTACGCTATGTCATCTTGGTGGGGGCAGCTTTC
GCAGAATCTAATTCGGGAGGAGACGTGGTACACTTGGCGCTCATGGCGACCTTCAAGATAC
AACCAGTGTTTATGGTGGCATCGTTTCTCAAAGCGAGATGGACCAACCAGGAGAACATTTTG
TTGATGTTGGCGGCTGTTTTCTTTCAAATGGCTTATCACGATGCCCGCCAAATTCTGCTCTG
GGAGATCCCTGATGTGTTGAATTCACTGGCGGTAGCTTGGATGATACTGAGAGCCATAACAT
TCACAACGACATCAAACGTGGTTGTTCCGCTGCTAGCCCTGCTAACACCCGGGCTGAGATG
CTTGAATCTGGATGTGTACAGGATACTGCTGTTGATGGTCGGAATAGGCAGCTTGATCAGGG
AGAAGAGGAGTGCAGCCGCAAAAAAGAAAGGAGCAAGTCTGCTATGCTTGGCTCTAGCCT
CAACAGGACTTTTCAACCCCATGATCCTTGCTGCTGGACTGATTGCATGTGATCCCAACCGT
AAACGCGGATGGCCCGCAACTGAAGTGATGACAGCTGTCGGCCTAATGTTTGCCATCGTCG
GAGGGCTGGCAGAGCTTGACATTGACTCCATGGCCATTCCAATGACTATCGCGGGGCTCAT
GTTTGCTGCTTTCGTGATTTCTGGGAAATCAACAGATATGTGGATTGAGAGAACGGCGGACA
TTTCCTGGGAAAGTGATGCAGAAATTACAGGCTCGAGCGAAAGAGTTGATGTGCGGCTTGA
TGATGATGGAAACTTCCAGCTCATGAATGATCCAGGAGCACCTTGGAAGATATGGATGCTCA
GAATGGTCTGTCTCGCGATTAGTGCGTACACCCCCTGGGCAATCTTGCCCTCAGTAGTTGG
ATTTTGGATAACTCTCCAATACACAAAGAGAGGAGGCGTGTTGTGGGACACTCCCTCACCA
AAGGAGTACAAAAAGGGGGACACGACCACCGGCGTCTACAGGATCATGACTCGTGGGCTG
CTCGGCAGTTATCAAGCAGGAGCGGGCGTGATGGTTGAAGGTGTTTTCCACACCCTTTGG
CATACAACAAAAGGAGCCGCTTTGATGAGCGGAGAGGGCCGCCTGGACCCATACTGGGGC
AGTGTCAAGGAGGATCGACTTTGTTACGGAGGACCCTGGAAATTGCAGCACAAGTGGAAC
GGGCAGGATGAGGTGCAGATGATTGTGGTGGAACCTGGCAAGAACGTTAAGAACGTCCAG
ACGAAACCAGGGGTGTTCAAAACACCTGAAGGAGAAATCGGGGCCGTGACTTTGGACTTC
CCCACTGGAACATCAGGCTCACCAATAGTGGACAAAAACGGTGATGTGATTGGGCTTTATG
GCAATGGAGTCATAATGCCCAACGGCTCATACATAAGCGCGATAGTGCAGGGTGAAAGGAT
GGATGAGCCAATCCCAGCCGGATTCGAACCTGAGATGCTGAGGAAAAAACAGATCACTGTA
CTGGATCTCCATCCCGGCGCCGGTAAAACAAGGAGGATTCTGCCACAGATCATCAAAGAGG
CCATAAACAGAAGACTGAGAACAGCCGTGCTAGCGCCAACCAGGGTTGTGGCTGCTGAGA
TGGCTGAAGCACTGAGAGGACTGCCCATCCGGTACCAGACATCCGCAGTGCCCAGAGAAC
ATAATGGAAATGAGATTGTTGATGTCATGTGTCATGCTACCCTCACCCACAGGCTGATGTCTC
CTCACAGGGTGCCGAACTACAACCTGTTCGTGATGGATGAGGCTCATTTCACCGACCCAGC

File S1 WNV reference sequences

TAGCATTGCAGCAAGAGGTTACATTTCCACAAAGGTCGAGCTAGGGGAGGCGGCGGCAATA
TTCATGACAGCCACCCCACCAGGCACTTCAGATCCATTCCCAGAGTCCAATTCACCAATTTC
CGACTTACAGACTGAGATCCCGGATCGAGCTTGGAACTCTGGATACGAATGGATCACAGAA
TACACCGGGAAGACGGTTTGGTTTGTGCCTAGTGTCAAGATGGGGAATGAGATTGCCCTTT
GCCTACAACGTGCTGGAAAGAAAGTAGTCCAATTGAACAGAAAGTCGTACGAGACGGAGTA
CCCAAAATGTAAGAACGATGATTGGGACTTTGTTATCACAACAGACATATCTGAAATGGGGG
CTAACTTCAAGGCGAGCAGGGTGATTGACAGCCGGAAGAGTGTGAAACCAACCATCATAAC
AGAAGGAGAAGGGAGAGTGATCCTGGGAGAACCATCTGCAGTGACAGCAGCTAGTGCCGC
CCAGAGACGTGGACGTATCGGTAGAAATCCGTCGCAAGTTGGTGATGAGTACTGTTATGGG
GGGCACACGAATGAAGACGACTCGAACTTCGCCCATTGGACTGAGGCACGAATCATGCTG
GACAACATCAACATGCCAAACGGACTGATCGCTCAATTCTACCAACCAGAGCGTGAGAAGG
TATATACCATGGATGGGGAATACCGGCTCAGAGGAGAAGAGAGAAAAAACTTTCTGGAACTG
TTGAGGACTGCAGATCTGCCAGTTTGGCTGGCTTACAAGGTTGCAGCGGCTGGAGTGTCAT
ACCACGACCGGAGGTGGTGCTTTGATGGTCCTAGGACAAACACAATTTTAGAAGACAACAA
CGAAGTGGAAGTCATCACGAAGCTTGGTGAAAGGAAGATTCTGAGGCCGCGCTGGATTGAT
GCCAGGGTGTACTCGGATCACCAGGCACTAAAGGCGTTCAAGGACTTCGCCTCGGGAAAA
CGTTCTCAGATAGGGCTCATTGAGGTTCTGGGAAAGATGCCTGAGCACTTCATGGGGAAGA
CATGGGAAGCACTTGACACCATGTACGTTGTGGCCACTGCAGAGAAAGGAGGAAGAGCTC
ACAGAATGGCCCTGGAGGAACTGCCAGATGCTCTTCAGACAATTGCCTTGATTGCCTTATTG
AGTGTGATGACCATGGGAGTATTCTTCCTCCTCATGCAGCGGAAGGGCATTGGAAAGATAG
GTTTGGGAGGCGCTGTCTTGGGAGTCGCGACCTTTTTCTGTTGGATGGCTGAAGTTCCAG
GAACGAAGATCGCCGGAATGTTGCTGCTCTCCCTTCTCTTGATGATTGTGCTAATTCCTGAG
CCAGAGAAGCAACGTTCGCAGACAGACAACCAGCTAGCCGTGTTCCTGATTTGTGTCATGA
CCCTTGTGAGCGCAGTGGCAGCCAACGAGATGGGTTGGCTAGATAAGACCAAGAGTGACA
TAAGCAGTTTGTTTGGGCAAAGAATTGAGGTCAAGGAGAATTTCAGCATGGGAGAGTTTCTT
CTGGACTTGAGGCCGGCAACAGCCTGGTCACTGTACGCTGTGACAACAGCGGTCCTCACT
CCACTGCTAAAGCATTTGATCACGTCAGATTACATCAACACCTCATTGACCTCAATAAACGTT
CAGGCAAGTGCACTATTCACACTCGCGCGAGGCTTCCCCTTCGTCGATGTTGGAGTGTCG
GCTCTCCTGCTAGCAGCCGGATGCTGGGGACAAGTCACCCTCACCGTTACGGTAACAGCG
GCAACACTCCTTTTTTGCCACTATGCCTACATGGTTCCCGGTTGGCAAGCTGAGGCAATGC
GCTCAGCCCAGCGGCGGACAGCGGCCGGAATCATGAAGAACGCTGTAGTGGATGGCATCG
TGGCCACGGACGTCCCAGAATTAGAGCGCACCACACCCATCATGCAGAAGAAAGTTGGAC
AGATCATGCTGATCTTGGTGTCTCTAGCTGCAGTAGTAGTGAACCCGTCTGTGAAGACAGTA
CGAGAAGCCGGAATTTTGATCACGGCCGCAGCGGTGACGCTTTGGGAGAATGGAGCAAGC
TCTGTTTGGAACGCAACAACTGCCATCGGACTCTGCCACATCATGCGTGGGGGTTGGTTGT
CATGTCTATCCATAACATGGACACTCATAAAGAACATGGAAAAACCAGGACTAAAAAGAGGT
GGGGCAAAAGGACGCACCTTGGGAGAGGTTTGGAAAGAAAGACTCAACCAGATGACAAAA
GAAGAGTTCACTAGGTACCGCAAAGAGGCCATCATCGAAGTCGATCGCTCAGCGGCAAAAC
ACGCCAGGAAAGAAGGCAATGTCACTGGAGGGCATCCAGTCTCTAGGGGCACAGCAAAAC
TGAGATGGCTGGTCGAACGGAGGTTTCTCGAACCGGTCGGAAAAGTGATTGACCTTGGAT
GTGGAAGAGGCGGTTGGTGTTACTATATGGCAACCCAAAAAAGAGTCCAAGAAGTCAGAGG
GTACACAAAGGGCGGTCCCGGACATGAAGAGCCCCAACTAGTGCAAAGTTATGGATGGAAC
ATTGTCACCATGAAGAGTGGAGTGGATGTGTTCTACAGACCTTCTGAGTGTTGTGACACCCT
CCTTTGTGACATCGGAGAGTCCTCGTCAAGTGCTGAGGTTGAAGAGCATAGGACGATTCGG
GTCCTTGAAATGGTTGAGGACTGGCTGCACCGAGGGCCAAGGGAATTTTGCGTGAAGGTG
CTCTGTCCCTACATGCCGAAAGTCATAGAGAAGATGGAGCTGCTCCAACGCCGGTATGGGG
GGGGACTGGTCAGAAACCCACTCTCACGGAATTCCACGCACGAGATGTATTGGGTGAGTC

File S1 WNV reference sequences

GAGCTTCAGGCAATGTGGTACATTCAGTGAATATGACCAGCCAGGTGCTCCTAGGAAGAAT
GGAAAAAAGGACCTGGAAGGGACCCCAATACGAGGAAGATGTAAACTTGGGAAGTGGAAC
CAGGGCGGTGGGAAAACCCCTGCTCAACTCAGACACCAGTAAAATCAAGAACAGGATTGAA
CGACTCAGGCGTGAGTACAGTTCGACGTGGCACCACGATGAGAACCACCCATATAGAACCT
GGAACTATCACGGCAGTTATGATGTGAAGCCCACAGGCTCCGCCAGTTCGCTGGTCAATGG
AGTGGTCAGGCTCCTCTCAAAACCATGGGACACCATCACGAATGTTACCACCATGGCCATG
ACTGACACTACTCCCTTCGGGCAGCAGCGAGTGTTCAAAGAGAAGGTGGACACGAAAGCT
CCTGAACCGCCAGAAGGAGTGAAGTACGTGCTCAACGAGACCACCAACTGGTTGTGGGCG
TTTTTGGCCAGAGAAAAACGTCCCAGAATGTGCTCTCGAGAGGAATTCATAAGAAAGGTCAA
CAGCAATGCAGCTTTGGGTGCCATGTTTGAAGAGCAGAATCAATGGAGGAGCGCCAGAGA
AGCAGTTGAAGATCCAAAATTTTGGGAGATGGTGGATGAGGAGCGCGAGGCACATCTGCG
GGGGGAATGTCACACTTGCATTTACAACATGATGGGAAAGAGAGAGAAAAAACCCGGAGAG
TTCGGAAAGGCCAAGGGAAGCAGAGCCATTTGGTTCATGTGGCTCGGAGCTCGCTTTCTG
GAGTTCGAGGCTCTGGGTTTTCTCAATGAAGACCACTGGCTTGGAAGAAAGAACTCAGGAG
GAGGTGTCGAGGGCTTGGGCCTCCAAAAACTGGGTTACATCCTGCGTGAAGTTGGCACCC
GGCCTGGGGGCAAGATCTATGCTGATGACACAGCTGGCTGGGACACCCGCATCACGAGAG
CTGACTTGGAAAATGAAGCTAAGGTGCTTGAGCTGCTTGATGGGGAACATCGGCGTCTTGC
CAGGGCCATCATTGAGCTCACCTATCGTCACAAAGTTGTGAAAGTGATGCGCCCGGCTGCT
GATGGAAGAACCGTCATGGATGTTATCTCCAGAGAAGATCAGAGGGGGAGTGGACAAGTTG
TCACCTACGCCCTAAACACTTTCACCAACCTGGCCGTCCAGCTGGTGAGGATGATGGAAGG
GGAAGGAGTGATTGGCCCAGATGATGTGGAGAAACTCACAAAAGGGAAAGGACCCAAAGT
CAGGACCTGGCTGTTTGAGAATGGGGAAGAAAGACTCAGCCGCATGGCTGTCAGTGGAGA
TGACTGTGTGGTAAAGCCCCTGGACGATCGCTTTGCCACCTCGCTCCACTTCCTCAATGCT
ATGTCAAAGGTTCGCAAAGACATCCAAGAGTGGAAACCGTCAACTGGATGGTATGATTGGC
AGCAGGTTCCATTTTGCTCAAACCATTTCACTGAATTGATCATGAAAGATGGAAGAACACTG
GTGGTTCCATGCCGAGGACAGGATGAATTGGTAGGCAGAGCTCGCATATCTCCAGGGGCC
GGATGGAACGTCCGCGACACTGCTTGTCTGGCTAAGTCTTATGCCCAGATGTGGCTGCTTC
TGTACTTCCACAGAAGAGACCTGCGGCTCATGGCCAACGCCATTTGCTCCGCTGTCCCTGT
GAATTGGGTCCCTACCGGAAGAACCACGTGGTCCATCCATGCAGGAGGAGAGTGGATGAC
AACAGAGGACATGTTGGAGGTCTGGAACCGTGTTTGGATAGAGGAGAATGAATGGATGGAA
GACAAAACCCCAGTGGAGAAATGGAGTGACGTCCCATATTCAGGAAAACGAGAGGACATCT
GGTGTGGCAGCCTGATTGGCACAAGAGCCCGAGCCACGTGGGCAGAAAACATCCAGGTG
GCTATCAACCAAGTCAGAGCAATCATCGGAGATGAGAAGTATGTGGACTACATGAGTTCACT
AAAGAGATATGAAGACACAACTTTGGTTGAGGACACAGTACTGTAGATATTTAATCAATTGTA
AATAGACAATATAAGTATGCATAAAAGTGTAGTTTTATAGTAGTATTTAGTGGTGTTAGTGTAAA
TAGTTAAGAAAATTTTGAGGAGAAAGTCAGGCCGGGAAGTTCCCGCCACCGGAAGTTGAGT
AGACGGTGCTGCCTGCGACTCAACCCCAGGAGGACTGGGTGAACAAAGCCGCGAAGTGA
TCCATGTAAGCCCTCAGAACCGTCTCGGAAGGAGGACCCCACATGTTGTAACTTCAAAGCC
CAATGTCAGACCACGCTACGGCGTGCTACTCTGCGGAGAGTGCAGTCTGCGATAGTGCCC
CAGGAGGACTGGGTTAACAAAGGCAAACCAACGCCCCACGCGGCCCTAGCCCCGGTAATG
GTGTTAACCAGGGCGAAAGGACTAGAGGTTAGAGGAGACCCCGCGGTTTAAAGTGCACGG
CCCAGCCTGGCTGAAGCTGTAGGTCAGGGGAAGGACTAGAGGTTAGTGGAGACCCCGTG
CCACAAAACACCACAACAAAACAGCATATTGACACCTGGGATAGACTAGGAGATCTTCTGCT
CTGCACAACCAGCCACACGGCACAGTGCGCCGACAATGGTGGCTGGTGGTGCGAGAACA
CAGGATCT

File S1 WNV reference sequences

> E-MAX+FVR

E sequences are in green. Nucleotide substitutions to introduce L107F, A316V, and K440R mutations are highlighted.

AGTAGTTCGCCTGTGTGAGCTGACAAACTTAGTAGTGTTTGTGAGGATTAACAACAATTAAC
ACAGTGCGAGCTGTTTCTTAGCACGAAGATCTCGATGTCTAAGAAACCAGGAGGGCCCGGC
AAGAGCCGGGCTGTCAATATGCTAAAACGCGGAATGCCCCGCGTGTTGTCCTTGATTGGAC
TGAAGAGGGCTATGTTGAGCCTGATCGACGGCAAGGGGCCAATACGATTTGTGTTGGCTCT
CTTGGCGTTCTTCAGGTTCACAGCAATTGCTCCGACCCGAGCAGTGCTGGATCGATGGAGA
GGTGTGAACAAACAAACAGCGATGAAACACCTTCTGAGTTTTAAGAAGGAACTAGGGACCT
TGACCAGTGCTATCAATCGGCGGAGCTCAAAACAAAAGAAAAGAGGAGGAAAGACCGGAAT
TGCAGTCATGATTGGCCTGATCGCCAGCGTAGGAGCAGTTACCCTCTCTAACTTCCAAGGG
AAGGTGATGATGACGGTAAATGCTACTGACGTCACAGATGTCATCACGATTCCAACAGCTGC
TGGAAAGAACCTATGCATTGTCAGAGCAATGGATGTGGGATACATGTGCGATGATACTATCA
CTTATGAATGCCCAGTACTGTCGGCTGGTAATGATCCAGAAGACATCGACTGTTGGTGCACA
AAGTCAGCAGTCTACGTCAGGTATGGAAGATGCACCAAGACACGCCACTCAAGACGCAGTC
GGAGGTCACTGACAGTGCAGACACACGGAGAAAGCACTCTAGCGAACAAGAAGGGGGCTT
GGATGGACAGCACCAAGGCCACAAGGTATTTGGTAAAAACAGAATCATGGATCTTGAGGAA
CCCTGGATATGCCCTGGTGGCAGCCGTCATTGGTTGGATGCTTGGGAGCAACACCATGCA
GAGAGTTGTGTTTGTCGTGCTATTGCTTTTGGTGGCCCCAGCTTACAGCTTCAACTGCCTT
GGAATGAGCAACAGAGACTTTTTGGAAGGCGTTTCCGGCGCGACGTGGGTCGATTTAGTT
CTCGAAGGGGATAGTTGCGTTACGATAATGTCGAAAGACAAACCGACGATCGACGTTAAA
ATGATGAATATGGAGGCCGCGAATCTAGCGGAAGTTCGCAGTTATTGTTATTTAGCAACCG
TAAGCGATCTTTCGACGAAAGCCGCGTGTCCGACGATGGGCGAAGCGCACAACGATAAA
CGGGCGGATCCCGCGTTTGTTTGTCGACAAGGCGTCGTCGATCGCGGTTGGGGAAATGG
TTGTGGATTTTTCGGCAAAGGAAGTATCGATACGTGCGCGAAATTTGCGTGTTCTACTAAA
GCGATAGGACGAACGATATTAAAAGAAAATATCAAATACGAAGTCGCGATTTTTGTCCACG
GACCGACGACGGTCGAATCGCACGGAAATTATTCGACGCAAGTCGGCGCGACGCAGGC
CGGTCGACTAAGTATAACGCCCGCGGCGCCGTCGTACACGCTAAAACTGGGCGAATATG
GCGAGGTTACGGTCGACTGCGAACCGCGTTCGGGTATCGATACGAACGCGTATTATGTTAT
GACGGTCGGAACGAAAACGTTTTTAGTACATCGGGAATGGTTCATGGATCTAAACCTACC
GTGGAGCAGTGCCGGAAGTACCGTTTGGCGTAACCGGGAAACGTTAATGGAATTCGAAG
AACCGCACGCGACTAAACAGTCCGTAATCGCGTTAGGATCGCAAGAAGGCGCGCTTCAT
CAAGCGTTAGCCGGCGCGATTCCGGTCGAATTTTCGAGTAATACCGTCAAATTAACATCCG
GTCATTTGAAATGTCGGGTCAAAATGGAAAAATTACAATTAAAAGGAACGACTTACGGCG
TATGTTCGAAAGCGTTCAAGTTTCTCGGTACACCCGTGGATACCGGTCACGGAACCGTCG
TTTTGGAATTACAATATACCGGAACCGATGGACCGTGTAAAGTTCCGATATCGTCCGTGGC
GTCGTTGAACGATCTAACACCCGTCGGCCGATTAGTCACCGTTAATCCGTTCGTTTCCGTC
GCGACGGCGAACGCGAAAGTACTTATAGAATTGGAACCGCCGTTTGGCGATTCGTATATC
GTCGTCGGCCGCGGGGAACAACAAATAAATCACCATTGGCATAAGTCCGGAAGTAGTATC
GGAAAAGCGTTTACGACGACGCTAAAAGGCGCGCAACGACTAGCCGCGCTCGGCGATAC
CGCGTGGGATTTCGGATCCGTCGGGGGCGTTTTTACGTCCGTCGGTCGAGCCGTACATCA
AGTATTCGGCGGGGCGTTTCGCTCGCTTTTCGGCGGAATGTCGTGGATAACGCAAGGATT
ACTGGGCGCGCTACTTTTATGGATGGGAATAAACGCGCGGGATCGTTCGATCGCGCTAAC
GTTTCTCGCCGTCGGCGGAGTTCTGCTCTTCCTCTCCGTGAACGTGCACGCTGACACTGG
GTGTGCCATAGACATCAGCCGGCAAGAGCTGAGATGTGGAAGTGGAGTGTTCATACACAAT
GATGTGGAGGCTTGGATGGACCGGTACAAGTATTACCCTGAAACGCCACAAGGCCTAGCCA

File S1 WNV reference sequences

AGATCATTCAGAAAGCTCATAAGGAAGGAGTGTGCGGTCTACGATCAGTTTCCAGACTGGA
GCATCAAATGTGGGAAGCAGTGAAGGACGAGCTGAACACTCTTTTGAAGGAGAATGGTGTG
GACCTTAGTGTCGTGGTTGAGAAACAGGAGGGAATGTACAAGTCAGCACCTAAACGCCTCA
CCGCCACCACGGAAAAATTGGAAATTGGCTGGAAGGCCTGGGGAAAGAGTATTTTATTTGC
ACCAGAACTCGCCAACAACACCTTTGTGGTTGATGGTCCGGAGACCAAGGAATGTCCGACT
CAGAATCGCGCTTGGAATAGCTTAGAAGTGGAGGATTTTGGATTTGGTCTCACCAGCACTC
GGATGTTCCTGAAGGTCAGAGAGAGCAACACAACTGAATGTGACTCGAAGATCATTGGAAC
GGCTGTCAAGAACAACTTGGCGATCCACAGTGACCTGTCCTATTGGATTGAAAGCAGGCTC
AATGATACGTGGAAGCTTGAAAGGGCAGTTCTGGGTGAAGTCAAATCATGTACGTGGCCTG
AGACGCATACCTTGTGGGGCGATGGAATCCTTGAGAGTGACTTGATAATACCAGTCACACTG
GCGGGACCACGAAGCAATCACAATCGGAGACCTGGGTACAAGACACAAAACCAGGGCCCA
TGGGACGAAGGCCGGGTAGAGATTGACTTCGATTACTGCCCAGGAACTACGGTCACCCTG
AGTGAGAGCTGCGGACACCGTGGACCTGCCACTCGCACCACCACAGAGAGCGGAAAGTT
GATAACAGATTGGTGCTGCAGGAGCTGCACCTTACCACCACTGCGCTACCAAACTGACAGC
GGCTGTTGGTATGGTATGGAGATCAGACCACAGAGACATGATGAAAAGACCCTCGTGCAGT
CACAAGTGAATGCTTATAATGCTGATATGATTGACCCTTTTCAGTTGGGCCTTCTGGTCGTGT
TCTTGGCCACCCAGGAGGTCCTTCGCAAGAGGTGGACAGCCAAGATCAGCATGCCAGCTA
TACTGATTGCTCTGCTAGTCCTGGTGTTTGGGGGCATTACTTACACTGATGTGTTACGCTATG
TCATCTTGGTGGGGGCAGCTTTCGCAGAATCTAATTCGGGAGGAGACGTGGTACACTTGGC
GCTCATGGCGACCTTCAAGATACAACCAGTGTTTATGGTGGCATCGTTTCTCAAAGCGAGAT
GGACCAACCAGGAGAACATTTTGTTGATGTTGGCGGCTGTTTTCTTTCAAATGGCTTATCAC
GATGCCCGCCAAATTCTGCTCTGGGAGATCCCTGATGTGTTGAATTCACTGGCGGTAGCTT
GGATGATACTGAGAGCCATAACATTCACAACGACATCAAACGTGGTTGTTCCGCTGCTAGCC
CTGCTAACACCCGGGCTGAGATGCTTGAATCTGGATGTGTACAGGATACTGCTGTTGATGGT
CGGAATAGGCAGCTTGATCAGGGAGAAGAGGAGTGCAGCCGCAAAAAAGAAAGGAGCAAG
TCTGCTATGCTTGGCTCTAGCCTCAACAGGACTTTTCAACCCCATGATCCTTGCTGCTGGAC
TGATTGCATGTGATCCCAACCGTAAACGCGGATGGCCCGCAACTGAAGTGATGACAGCTGT
CGGCCTAATGTTTGCCATCGTCGGAGGGCTGGCAGAGCTTGACATTGACTCCATGGCCATT
CCAATGACTATCGCGGGGCTCATGTTTGCTGCTTTCGTGATTTCTGGGAAATCAACAGATAT
GTGGATTGAGAGAACGGCGGACATTTCCTGGGAAAGTGATGCAGAAATTACAGGCTCGAGC
GAAAGAGTTGATGTGCGGCTTGATGATGATGGAAACTTCCAGCTCATGAATGATCCAGGAG
CACCTTGGAAGATATGGATGCTCAGAATGGTCTGTCTCGCGATTAGTGCGTACACCCCCTG
GGCAATCTTGCCCTCAGTAGTTGGATTTTGGATAACTCTCCAATACACAAAGAGAGGAGGCG
TGTTGTGGGACACTCCCTCACCAAAGGAGTACAAAAAGGGGGACACGACCACCGGCGTCT
ACAGGATCATGACTCGTGGGCTGCTCGGCAGTTATCAAGCAGGAGCGGGCGTGATGGTTG
AAGGTGTTTTCCACACCCTTTGGCATACAACAAAAGGAGCCGCTTTGATGAGCGGAGAGGG
CCGCCTGGACCCATACTGGGGCAGTGTCAAGGAGGATCGACTTTGTTACGGAGGACCCTG
GAAATTGCAGCACAAGTGGAACGGGCAGGATGAGGTGCAGATGATTGTGGTGGAACCTGG
CAAGAACGTTAAGAACGTCCAGACGAAACCAGGGGTGTTCAAAACACCTGAAGGAGAAATC
GGGGCCGTGACTTTGGACTTCCCCACTGGAACATCAGGCTCACCAATAGTGGACAAAAAC
GGTGATGTGATTGGGCTTTATGGCAATGGAGTCATAATGCCCAACGGCTCATACATAAGCGC
GATAGTGCAGGGTGAAAGGATGGATGAGCCAATCCCAGCCGGATTCGAACCTGAGATGCTG
AGGAAAAAACAGATCACTGTACTGGATCTCCATCCCGGCGCCGGTAAAACAAGGAGGATTC
TGCCACAGATCATCAAAGAGGCCATAAACAGAAGACTGAGAACAGCCGTGCTAGCGCCAAC
CAGGGTTGTGGCTGCTGAGATGGCTGAAGCACTGAGAGGACTGCCCATCCGGTACCAGAC
ATCCGCAGTGCCCAGAGAACATAATGGAAATGAGATTGTTGATGTCATGTGTCATGCTACCC
TCACCCACAGGCTGATGTCTCCTCACAGGGTGCCGAACTACAACCTGTTCGTGATGGATGA

File S1 WNV reference sequences

GGCTCATTTCACCGACCCAGCTAGCATTGCAGCAAGAGGTTACATTTCCACAAAGGTCGAG
CTAGGGGAGGCGGCGGCAATATTCATGACAGCCACCCCACCAGGCACTTCAGATCCATTCC
CAGAGTCCAATTCACCAATTTCCGACTTACAGACTGAGATCCCGGATCGAGCTTGGAACTCT
GGATACGAATGGATCACAGAATACACCGGGAAGACGGTTTGGTTTGTGCCTAGTGTCAAGA
TGGGGAATGAGATTGCCCTTTGCCTACAACGTGCTGGAAAGAAAGTAGTCCAATTGAACAG
AAAGTCGTACGAGACGGAGTACCCAAAATGTAAGAACGATGATTGGGACTTTGTTATCACAA
CAGACATATCTGAAATGGGGGCTAACTTCAAGGCGAGCAGGGTGATTGACAGCCGGAAGA
GTGTGAAACCAACCATCATAACAGAAGGAGAAGGGAGAGTGATCCTGGGAGAACCATCTGC
AGTGACAGCAGCTAGTGCCGCCCAGAGACGTGGACGTATCGGTAGAAATCCGTCGCAAGT
TGGTGATGAGTACTGTTATGGGGGCACACGAATGAAGACGACTCGAACTTCGCCCATTGG
ACTGAGGCACGAATCATGCTGGACAACATCAACATGCCAAACGGACTGATCGCTCAATTCTA
CCAACCAGAGCGTGAGAAGGTATATACCATGGATGGGGAATACCGGCTCAGAGGAGAAGAG
AGAAAAAACTTTCTGGAACTGTTGAGGACTGCAGATCTGCCAGTTTGGCTGGCTTACAAGG
TTGCAGCGGCTGGAGTGTCATACCACGACCGGAGGTGGTGCTTTGATGGTCCTAGGACAA
ACACAATTTTAGAAGACAACAACGAAGTGGAAGTCATCACGAAGCTTGGTGAAAGGAAGATT
CTGAGGCCGCGCTGGATTGATGCCAGGGTGTACTCGGATCACCAGGCACTAAAGGCGTTC
AAGGACTTCGCCTCGGGAAAACGTTCTCAGATAGGGCTCATTGAGGTTCTGGGAAAGATGC
CTGAGCACTTCATGGGGAAGACATGGGAAGCACTTGACACCATGTACGTTGTGGCCACTGC
AGAGAAAGGAGGAAGAGCTCACAGAATGGCCCTGGAGGAACTGCCAGATGCTCTTCAGAC
AATTGCCTTGATTGCCTTATTGAGTGTGATGACCATGGGAGTATTCTTCCTCCTCATGCAGC
GGAAGGGCATTGGAAAGATAGGTTTGGGAGGCGCTGTCTTGGGAGTCGCGACCTTTTTCT
GTTGGATGGCTGAAGTTCCAGGAACGAAGATCGCCGGAATGTTGCTGCTCTCCCTTCTCTT
GATGATTGTGCTAATTCCTGAGCCAGAGAAGCAACGTTCGCAGACAGACAACCAGCTAGCC
GTGTTCCTGATTTGTGTCATGACCCTTGTGAGCGCAGTGGCAGCCAACGAGATGGGTTGG
CTAGATAAGACCAAGAGTGACATAAGCAGTTTGTTTGGGCAAAGAATTGAGGTCAAGGAGA
ATTTCAGCATGGGAGAGTTTCTTCTGGACTTGAGGCCGGCAACAGCCTGGTCACTGTACGC
TGTGACAACAGCGGTCCTCACTCCACTGCTAAAGCATTTGATCACGTCAGATTACATCAACA
CCTCATTGACCTCAATAAACGTTCAGGCAAGTGCACTATTCACACTCGCGCGAGGCTTCCC
CTTCGTCGATGTTGGAGTGTCGGCTCTCCTGCTAGCAGCCGGATGCTGGGGACAAGTCAC
CCTCACCGTTACGGTAACAGCGGCAACACTCCTTTTTTGCCACTATGCCTACATGGTTCCCG
GTTGGCAAGCTGAGGCAATGCGCTCAGCCCAGCGGCGGACAGCGGCCGGAATCATGAAG
AACGCTGTAGTGGATGGCATCGTGGCCACGGACGTCCCAGAATTAGAGCGCACCACACCC
ATCATGCAGAAGAAAGTTGGACAGATCATGCTGATCTTGGTGTCTCTAGCTGCAGTAGTAGT
GAACCCGTCTGTGAAGACAGTACGAGAAGCCGGAATTTTGATCACGGCCGCAGCGGTGAC
GCTTTGGGAGAATGGAGCAAGCTCTGTTTGGAACGCAACAACTGCCATCGGACTCTGCCA
CATCATGCGTGGGGGTTGGTTGTCATGTCTATCCATAACATGGACACTCATAAAGAACATGG
AAAAACCAGGACTAAAAAGAGGTGGGGCAAAAGGACGCACCTTGGGAGAGGTTTGGAAAG
AAAGACTCAACCAGATGACAAAAGAAGAGTTCACTAGGTACCGCAAAGAGGCCATCATCGA
AGTCGATCGCTCAGCGGCAAAACACGCCAGGAAAGAAGGCAATGTCACTGGAGGGCATCC
AGTCTCTAGGGGCACAGCAAAACTGAGATGGCTGGTCGAACGGAGGTTTCTCGAACCGGT
CGGAAAAGTGATTGACCTTGGATGTGGAAGAGGCGGTTGGTGTTACTATATGGCAACCCAA
AAAAGAGTCCAAGAAGTCAGAGGGTACACAAAGGGCGGTCCCGGACATGAAGAGCCCCAA
CTAGTGCAAAGTTATGGATGGAACATTGTCACCATGAAGAGTGGAGTGGATGTGTTCTACAG
ACCTTCTGAGTGTTGTGACACCCTCCTTTGTGACATCGGAGAGTCCTCGTCAAGTGCTGAG
GTTGAAGAGCATAGGACGATTCGGGTCCTTGAAATGGTTGAGGACTGGCTGCACCGAGGG
CCAAGGGAATTTTGCGTGAAGGTGCTCTGTCCCTACATGCCGAAAGTCATAGAGAAGATGG
AGCTGCTCCAACGCCGGTATGGGGGGGGACTGGTCAGAAACCCACTCTCACGGAATTCCA

File S1 WNV reference sequences

CGCACGAGATGTATTGGGTGAGTCGAGCTTCAGGCAATGTGGTACATTCAGTGAATATGACC
AGCCAGGTGCTCCTAGGAAGAATGGAAAAAAGGACCTGGAAGGGACCCCAATACGAGGAA
GATGTAAACTTGGGAAGTGGAACCAGGGCGGTGGGAAAACCCCTGCTCAACTCAGACACC
AGTAAAATCAAGAACAGGATTGAACGACTCAGGCGTGAGTACAGTTCGACGTGGCACCACG
ATGAGAACCACCCATATAGAACCTGGAACTATCACGGCAGTTATGATGTGAAGCCCACAGGC
TCCGCCAGTTCGCTGGTCAATGGAGTGGTCAGGCTCCTCTCAAAACCATGGGACACCATCA
CGAATGTTACCACCATGGCCATGACTGACACTACTCCCTTCGGGCAGCAGCGAGTGTTCAA
AGAGAAGGTGGACACGAAAGCTCCTGAACCGCCAGAAGGAGTGAAGTACGTGCTCAACGA
GACCACCAACTGGTTGTGGGCGTTTTTGGCCAGAGAAAAACGTCCCAGAATGTGCTCTCGA
GAGGAATTCATAAGAAAGGTCAACAGCAATGCAGCTTTGGGTGCCATGTTTGAAGAGCAGA
ATCAATGGAGGAGCGCCAGAGAAGCAGTTGAAGATCCAAAATTTTGGGAGATGGTGGATGA
GGAGCGCGAGGCACATCTGCGGGGGGAATGTCACACTTGCATTTACAACATGATGGGAAA
GAGAGAGAAAAAACCCGGAGAGTTCGGAAAGGCCAAGGGAAGCAGAGCCATTTGGTTCAT
GTGGCTCGGAGCTCGCTTTCTGGAGTTCGAGGCTCTGGGTTTTCTCAATGAAGACCACTG
GCTTGGAAGAAAGAACTCAGGAGGAGGTGTCGAGGGCTTGGGCCTCCAAAAACTGGGTTA
CATCCTGCGTGAAGTTGGCACCCGGCCTGGGGGCAAGATCTATGCTGATGACACAGCTGG
CTGGGACACCCGCATCACGAGAGCTGACTTGGAAAATGAAGCTAAGGTGCTTGAGCTGCTT
GATGGGGAACATCGGCGTCTTGCCAGGGCCATCATTGAGCTCACCTATCGTCACAAAGTTG
TGAAAGTGATGCGCCCGGCTGCTGATGGAAGAACCGTCATGGATGTTATCTCCAGAGAAGA
TCAGAGGGGGAGTGGACAAGTTGTCACCTACGCCCTAAACACTTTCACCAACCTGGCCGT
CCAGCTGGTGAGGATGATGGAAGGGGAAGGAGTGATTGGCCCAGATGATGTGGAGAAACT
CACAAAAGGGAAAGGACCCAAAGTCAGGACCTGGCTGTTTGAGAATGGGGAAGAAAGACT
CAGCCGCATGGCTGTCAGTGGAGATGACTGTGTGGTAAAGCCCCTGGACGATCGCTTTGC
CACCTCGCTCCACTTCCTCAATGCTATGTCAAAGGTTCGCAAAGACATCCAAGAGTGGAAA
CCGTCAACTGGATGGTATGATTGGCAGCAGGTTCCATTTTGCTCAAACCATTTCACTGAATT
GATCATGAAAGATGGAAGAACACTGGTGGTTCCATGCCGAGGACAGGATGAATTGGTAGGC
AGAGCTCGCATATCTCCAGGGGCCGGATGGAACGTCCGCGACACTGCTTGTCTGGCTAAG
TCTTATGCCCAGATGTGGCTGCTTCTGTACTTCCACAGAAGAGACCTGCGGCTCATGGCCA
ACGCCATTTGCTCCGCTGTCCCTGTGAATTGGGTCCCTACCGGAAGAACCACGTGGTCCAT
CCATGCAGGAGGAGAGTGGATGACAACAGAGGACATGTTGGAGGTCTGGAACCGTGTTTG
GATAGAGGAGAATGAATGGATGGAAGACAAAACCCCAGTGGAGAAATGGAGTGACGTCCCA
TATTCAGGAAAACGAGAGGACATCTGGTGTGGCAGCCTGATTGGCACAAGAGCCCGAGCC
ACGTGGGCAGAAAACATCCAGGTGGCTATCAACCAAGTCAGAGCAATCATCGGAGATGAGA
AGTATGTGGACTACATGAGTTCACTAAAGAGATATGAAGACACAACTTTGGTTGAGGACACA
GTACTGTAGATATTTAATCAATTGTAAATAGACAATATAAGTATGCATAAAAGTGTAGTTTTATAG
TAGTATTTAGTGGTGTTAGTGTAAATAGTTAAGAAAATTTTGAGGAGAAAGTCAGGCCGGGAA
GTTCCCGCCACCGGAAGTTGAGTAGACGGTGCTGCCTGCGACTCAACCCCAGGAGGACTG
GGTGAACAAAGCCGCGAAGTGATCCATGTAAGCCCTCAGAACCGTCTCGGAAGGAGGACC
CCACATGTTGTAACTTCAAAGCCCAATGTCAGACCACGCTACGGCGTGCTACTCTGCGGAG
AGTGCAGTCTGCGATAGTGCCCCAGGAGGACTGGGTTAACAAAGGCAAACCAACGCCCCA
CGCGGCCCTAGCCCCGGTAATGGTGTTAACCAGGGCGAAAGGACTAGAGGTTAGAGGAGA
CCCCGCGGTTTAAAGTGCACGGCCCAGCCTGGCTGAAGCTGTAGGTCAGGGGAAGGACTA
GAGGTTAGTGGAGACCCCGTGCCACAAAACACCACAACAAAACAGCATATTGACACCTGGG
ATAGACTAGGAGATCTTCTGCTCTGCACAACCAGCCACACGGCACAGTGCGCCGACAATGG
TGGCTGGTGGTGCGAGAACACAGGATCT

File S1 WNV reference sequences

> E-MAX+FR

E sequences are in green. Nucleotide substitutions to introduce L107F and K440R mutations are highlighted.

AGTAGTTCGCCTGTGTGAGCTGACAAACTTAGTAGTGTTTGTGAGGATTAACAACAATTAAC
ACAGTGCGAGCTGTTTCTTAGCACGAAGATCTCGATGTCTAAGAAACCAGGAGGGCCCGGC
AAGAGCCGGGCTGTCAATATGCTAAAACGCGGAATGCCCCGCGTGTTGTCCTTGATTGGAC
TGAAGAGGGCTATGTTGAGCCTGATCGACGGCAAGGGGCCAATACGATTTGTGTTGGCTCT
CTTGGCGTTCTTCAGGTTCACAGCAATTGCTCCGACCCGAGCAGTGCTGGATCGATGGAGA
GGTGTGAACAAACAAACAGCGATGAAACACCTTCTGAGTTTTAAGAAGGAACTAGGGACCT
TGACCAGTGCTATCAATCGGCGGAGCTCAAAACAAAAGAAAAGAGGAGGAAAGACCGGAAT
TGCAGTCATGATTGGCCTGATCGCCAGCGTAGGAGCAGTTACCCTCTCTAACTTCCAAGGG
AAGGTGATGATGACGGTAAATGCTACTGACGTCACAGATGTCATCACGATTCCAACAGCTGC
TGGAAAGAACCTATGCATTGTCAGAGCAATGGATGTGGGATACATGTGCGATGATACTATCA
CTTATGAATGCCCAGTACTGTCGGCTGGTAATGATCCAGAAGACATCGACTGTTGGTGCACA
AAGTCAGCAGTCTACGTCAGGTATGGAAGATGCACCAAGACACGCCACTCAAGACGCAGTC
GGAGGTCACTGACAGTGCAGACACACGGAGAAAGCACTCTAGCGAACAAGAAGGGGGCTT
GGATGGACAGCACCAAGGCCACAAGGTATTTGGTAAAAACAGAATCATGGATCTTGAGGAA
CCCTGGATATGCCCTGGTGGCAGCCGTCATTGGTTGGATGCTTGGGAGCAACACCATGCA
GAGAGTTGTGTTTGTCGTGCTATTGCTTTTGGTGGCCCCAGCTTACAGC**TTCAACTGCCTT**
GGAATGAGCAACAGAGACTTTTTGGAAGGCGTTTCCGGCGCGACGTGGGTCGATTTAGTT
CTCGAAGGGGATAGTTGCGTTACGATAATGTCGAAAGACAAACCGACGATCGACGTTAAA
ATGATGAATATGGAGGCCGCGAATCTAGCGGAAGTTCGCAGTTATTGTTATTTAGCAACCG
TAAGCGATCTTTCGACGAAAGCCGCGTGTCCGACGATGGGCGAAGCGCACAACGATAAA
CGGGCGGATCCCGCGTTTGTTTGTCGACAAGGCGTCGTCGATCGCGGTTGGGGAAATGG
TTGTGGATTTTTCGGCAAAGGAAGTATCGATACGTGCGCGAAATTTGCGTGTTCTACTAAA
GCGATAGGACGAACGATATTAAAAGAAAATATCAAATACGAAGTCGCGATTTTTGTCCACG
GACCGACGACGGTCGAATCGCACGGAAATTATTCGACGCAAGTCGGCGCGACGCAGGC
CGGTCGACTAAGTATAACGCCCGCGGCGCCGTCGTACACGCTAAAACTGGGCGAATATG
GCGAGGTTACGGTCGACTGCGAACCGCGTTCGGGTATCGATACGAACGCGTATTATGTTAT
GACGGTCGGAACGAAAACGTTTTTAGTACATCGGGAATGGTTCATGGATCTAAACCTACC
GTGGAGCAGTGCCGGAAGTACCGTTTGGCGTAACCGGGAAACGTTAATGGAATTCGAAG
AACCGCACGCGACTAAACAGTCCGTAATCGCGTTAGGATCGCAAGAAGGCGCGCTTCAT
CAAGCGTTAGCCGGCGCGATTCCGGTCGAATTTTCGAGTAATACCGTCAAATTAACATCCG
GTCATTTGAAATGTCGGGTCAAAATGGAAAAATTACAATTAAAAGGAACGACTTACGGCG
TATGTTCGAAAGCGTTCAAGTTTCTCGGTACACCCGCCGATACCGGTCACGGAACCGTCG
TTTTGGAATTACAATATACCGGAACCGATGGACCGTGTAAAGTTCCGATATCGTCCGTGGC
GTCGTTGAACGATCTAACACCCGTCGGCCGATTAGTCACCGTTAATCCGTTCGTTTCCGTC
GCGACGGCGAACGCGAAAGTACTTATAGAATTGGAACCGCCGTTTGGCGATTCGTATATC
GTCGTCGGCCGCGGGGAACAACAAATAAATCACCATTGGCATAAGTCCGGAAGTAGTATC
GGAAAAGCGTTTACGACGACGCTAAAAGGCGCGCAACGACTAGCCGCGCTCGGCGATAC
CGCGTGGGATTTCGGATCCGTCGGGGGCGTTTTTACGTCCGTCGGTCGAGCCGTACATCA
AGTATTCGGCGGGGCGTTTCGCTCGCTTTTCGGCGGAATGTCGTGGATAACGCAAGGATT
ACTGGGCGCGCTACTTTTATGGATGGGAATAAACGCGCGGATCGTTCGATCGCGCTAAC
GTTTCTCGCCGTCGGCGGAGTTCTGCTCTTCCTCTCCGTGAACGTGCACGCTGACACTGG
GTGTGCCATAGACATCAGCCGGCAAGAGCTGAGATGTGGAAGTGGAGTGTTCATACACAAT
GATGTGGAGGCTTGGATGGACCGGTACAAGTATTACCCTGAAACGCCACAAGGCCTAGCCA

File S1 WNV reference sequences

AGATCATTCAGAAAGCTCATAAGGAAGGAGTGTGCGGTCTACGATCAGTTTCCAGACTGGA
GCATCAAATGTGGGAAGCAGTGAAGGACGAGCTGAACACTCTTTTGAAGGAGAATGGTGTG
GACCTTAGTGTCGTGGTTGAGAAACAGGAGGGAATGTACAAGTCAGCACCTAAACGCCTCA
CCGCCACCACGGAAAAATTGGAAATTGGCTGGAAGGCCTGGGGAAAGAGTATTTTATTTGC
ACCAGAACTCGCCAACAACACCTTTGTGGTTGATGGTCCGGAGACCAAGGAATGTCCGACT
CAGAATCGCGCTTGGAATAGCTTAGAAGTGGAGGATTTTGGATTTGGTCTCACCAGCACTC
GGATGTTCCTGAAGGTCAGAGAGAGCAACACAACTGAATGTGACTCGAAGATCATTGGAAC
GGCTGTCAAGAACAACTTGGCGATCCACAGTGACCTGTCCTATTGGATTGAAAGCAGGCTC
AATGATACGTGGAAGCTTGAAAGGGCAGTTCTGGGTGAAGTCAAATCATGTACGTGGCCTG
AGACGCATACCTTGTGGGGCGATGGAATCCTTGAGAGTGACTTGATAATACCAGTCACACTG
GCGGGACCACGAAGCAATCACAATCGGAGACCTGGGTACAAGACACAAAACCAGGGCCCA
TGGGACGAAGGCCGGGTAGAGATTGACTTCGATTACTGCCCAGGAACTACGGTCACCCTG
AGTGAGAGCTGCGGACACCGTGGACCTGCCACTCGCACCACCACAGAGAGCGGAAAGTT
GATAACAGATTGGTGCTGCAGGAGCTGCACCTTACCACCACTGCGCTACCAAACTGACAGC
GGCTGTTGGTATGGTATGGAGATCAGACCACAGAGACATGATGAAAAGACCCTCGTGCAGT
CACAAGTGAATGCTTATAATGCTGATATGATTGACCCTTTTCAGTTGGGCCTTCTGGTCGTGT
TCTTGGCCACCCAGGAGGTCCTTCGCAAGAGGTGGACAGCCAAGATCAGCATGCCAGCTA
TACTGATTGCTCTGCTAGTCCTGGTGTTTGGGGGCATTACTTACACTGATGTGTTACGCTATG
TCATCTTGGTGGGGGCAGCTTTCGCAGAATCTAATTCGGGAGGAGACGTGGTACACTTGGC
GCTCATGGCGACCTTCAAGATACAACCAGTGTTTATGGTGGCATCGTTTCTCAAAGCGAGAT
GGACCAACCAGGAGAACATTTTGTTGATGTTGGCGGCTGTTTTCTTTCAAATGGCTTATCAC
GATGCCCGCCAAATTCTGCTCTGGGAGATCCCTGATGTGTTGAATTCACTGGCGGTAGCTT
GGATGATACTGAGAGCCATAACATTCACAACGACATCAAACGTGGTTGTTCCGCTGCTAGCC
CTGCTAACACCCGGGCTGAGATGCTTGAATCTGGATGTGTACAGGATACTGCTGTTGATGGT
CGGAATAGGCAGCTTGATCAGGGAGAAGAGGAGTGCAGCCGCAAAAAAGAAAGGAGCAAG
TCTGCTATGCTTGGCTCTAGCCTCAACAGGACTTTTCAACCCCATGATCCTTGCTGCTGGAC
TGATTGCATGTGATCCCAACCGTAAACGCGGATGGCCCGCAACTGAAGTGATGACAGCTGT
CGGCCTAATGTTTGCCATCGTCGGAGGGCTGGCAGAGCTTGACATTGACTCCATGGCCATT
CCAATGACTATCGCGGGGCTCATGTTTGCTGCTTTCGTGATTTCTGGGAAATCAACAGATAT
GTGGATTGAGAGAACGGCGGACATTTCCTGGGAAAGTGATGCAGAAATTACAGGCTCGAGC
GAAAGAGTTGATGTGCGGCTTGATGATGATGGAAACTTCCAGCTCATGAATGATCCAGGAG
CACCTTGGAAGATATGGATGCTCAGAATGGTCTGTCTCGCGATTAGTGCGTACACCCCCTG
GGCAATCTTGCCCTCAGTAGTTGGATTTTGGATAACTCTCCAATACACAAAGAGAGGAGGCG
TGTTGTGGGACACTCCCTCACCAAAGGAGTACAAAAAGGGGGACACGACCACCGGCGTCT
ACAGGATCATGACTCGTGGGCTGCTCGGCAGTTATCAAGCAGGAGCGGGCGTGATGGTTG
AAGGTGTTTTCCACACCCTTTGGCATACAACAAAAGGAGCCGCTTTGATGAGCGGAGAGGG
CCGCCTGGACCCATACTGGGGCAGTGTCAAGGAGGATCGACTTTGTTACGGAGGACCCTG
GAAATTGCAGCACAAGTGGAACGGGCAGGATGAGGTGCAGATGATTGTGGTGGAACCTGG
CAAGAACGTTAAGAACGTCCAGACGAAACCAGGGGTGTTCAAAACACCTGAAGGAGAAATC
GGGGCCGTGACTTTGGACTTCCCCACTGGAACATCAGGCTCACCAATAGTGGACAAAAAC
GGTGATGTGATTGGGCTTTATGGCAATGGAGTCATAATGCCCAACGGCTCATACATAAGCGC
GATAGTGCAGGGTGAAAGGATGGATGAGCCAATCCCAGCCGGATTCGAACCTGAGATGCTG
AGGAAAAAACAGATCACTGTACTGGATCTCCATCCCGGCGCCGGTAAAACAAGGAGGATTC
TGCCACAGATCATCAAAGAGGCCATAAACAGAAGACTGAGAACAGCCGTGCTAGCGCCAAC
CAGGGTTGTGGCTGCTGAGATGGCTGAAGCACTGAGAGGACTGCCCATCCGGTACCAGAC
ATCCGCAGTGCCCAGAGAACATAATGGAAATGAGATTGTTGATGTCATGTGTCATGCTACCC
TCACCCACAGGCTGATGTCTCCTCACAGGGTGCCGAACTACAACCTGTTCGTGATGGATGA

File S1 WNV reference sequences

GGCTCATTTCACCGACCCAGCTAGCATTGCAGCAAGAGGTTACATTTCCACAAAGGTCGAG
CTAGGGGAGGCGGCGGCAATATTCATGACAGCCACCCCACCAGGCACTTCAGATCCATTCC
CAGAGTCCAATTCACCAATTTCCGACTTACAGACTGAGATCCCGGATCGAGCTTGGAACTCT
GGATACGAATGGATCACAGAATACACCGGGAAGACGGTTTGGTTTGTGCCTAGTGTCAAGA
TGGGGAATGAGATTGCCCTTTGCCTACAACGTGCTGGAAAGAAAGTAGTCCAATTGAACAG
AAAGTCGTACGAGACGGAGTACCCAAAATGTAAGAACGATGATTGGGACTTTGTTATCACAA
CAGACATATCTGAAATGGGGGCTAACTTCAAGGCGAGCAGGGTGATTGACAGCCGGAAGA
GTGTGAAACCAACCATCATAACAGAAGGAGAAGGGAGAGTGATCCTGGGAGAACCATCTGC
AGTGACAGCAGCTAGTGCCGCCCAGAGACGTGGACGTATCGGTAGAAATCCGTCGCAAGT
TGGTGATGAGTACTGTTATGGGGGGCACACGAATGAAGACGACTCGAACTTCGCCCATTGG
ACTGAGGCACGAATCATGCTGGACAACATCAACATGCCAAACGGACTGATCGCTCAATTCTA
CCAACCAGAGCGTGAGAAGGTATATACCATGGATGGGGAATACCGGCTCAGAGGAGAAGAG
AGAAAAAACTTTCTGGAACTGTTGAGGACTGCAGATCTGCCAGTTTGGCTGGCTTACAAGG
TTGCAGCGGCTGGAGTGTCATACCACGACCGGAGGTGGTGCTTTGATGGTCCTAGGACAA
ACACAATTTTAGAAGACAACAACGAAGTGGAAGTCATCACGAAGCTTGGTGAAAGGAAGATT
CTGAGGCCGCGCTGGATTGATGCCAGGGTGTACTCGGATCACCAGGCACTAAAGGCGTTC
AAGGACTTCGCCTCGGGAAAACGTTCTCAGATAGGGCTCATTGAGGTTCTGGGAAAGATGC
CTGAGCACTTCATGGGGAAGACATGGGAAGCACTTGACACCATGTACGTTGTGGCCACTGC
AGAGAAAGGAGGAAGAGCTCACAGAATGGCCCTGGAGGAACTGCCAGATGCTCTTCAGAC
AATTGCCTTGATTGCCTTATTGAGTGTGATGACCATGGGAGTATTCTTCCTCCTCATGCAGC
GGAAGGGCATTGGAAAGATAGGTTTGGGAGGCGCTGTCTTGGGAGTCGCGACCTTTTTCT
GTTGGATGGCTGAAGTTCCAGGAACGAAGATCGCCGGAATGTTGCTGCTCTCCCTTCTCTT
GATGATTGTGCTAATTCCTGAGCCAGAGAAGCAACGTTCGCAGACAGACAACCAGCTAGCC
GTGTTCCTGATTTGTGTCATGACCCTTGTGAGCGCAGTGGCAGCCAACGAGATGGGTTGG
CTAGATAAGACCAAGAGTGACATAAGCAGTTTGTTTGGGCAAAGAATTGAGGTCAAGGAGA
ATTTCAGCATGGGAGAGTTTCTTCTGGACTTGAGGCCGGCAACAGCCTGGTCACTGTACGC
TGTGACAACAGCGGTCCTCACTCCACTGCTAAAGCATTTGATCACGTCAGATTACATCAACA
CCTCATTGACCTCAATAAACGTTCAGGCAAGTGCACTATTCACACTCGCGCGAGGCTTCCC
CTTCGTCGATGTTGGAGTGTCGGCTCTCCTGCTAGCAGCCGGATGCTGGGGACAAGTCAC
CCTCACCGTTACGGTAACAGCGGCAACACTCCTTTTTTGCCACTATGCCTACATGGTTCCCG
GTTGGCAAGCTGAGGCAATGCGCTCAGCCCAGCGGCGGACAGCGGCCGGAATCATGAAG
AACGCTGTAGTGGATGGCATCGTGGCCACGGACGTCCCAGAATTAGAGCGCACCACACCC
ATCATGCAGAAGAAAGTTGGACAGATCATGCTGATCTTGGTGTCTCTAGCTGCAGTAGTAGT
GAACCCGTCTGTGAAGACAGTACGAGAAGCCGGAATTTTGATCACGGCCGCAGCGGTGAC
GCTTTGGGAGAATGGAGCAAGCTCTGTTTGGAACGCAACAACTGCCATCGGACTCTGCCA
CATCATGCGTGGGGGTTGGTTGTCATGTCTATCCATAACATGGACACTCATAAAGAACATGG
AAAAACCAGGACTAAAAAGAGGTGGGGCAAAAGGACGCACCTTGGGAGAGGTTTGGAAAG
AAAGACTCAACCAGATGACAAAAGAAGAGTTCACTAGGTACCGCAAAGAGGCCATCATCGA
AGTCGATCGCTCAGCGGCAAAACACGCCAGGAAAGAAGGCAATGTCACTGGAGGGCATCC
AGTCTCTAGGGGCACAGCAAAACTGAGATGGCTGGTCGAACGGAGGTTTCTCGAACCGGT
CGGAAAAGTGATTGACCTTGGATGTGGAAGAGGCGGTTGGTGTTACTATATGGCAACCCAA
AAAAGAGTCCAAGAAGTCAGAGGGTACACAAAGGGCGGTCCCGGACATGAAGAGCCCCAA
CTAGTGCAAAGTTATGGATGGAACATTGTCACCATGAAGAGTGGAGTGGATGTGTTCTACAG
ACCTTCTGAGTGTTGTGACACCCTCCTTTGTGACATCGGAGAGTCCTCGTCAAGTGCTGAG
GTTGAAGAGCATAGGACGATTCGGGTCCTTGAAATGGTTGAGGACTGGCTGCACCGAGGG
CCAAGGGAATTTTGCGTGAAGGTGCTCTGTCCCTACATGCCGAAAGTCATAGAGAAGATGG
AGCTGCTCCAACGCCGGTATGGGGGGGGACTGGTCAGAAACCCACTCTCACGGAATTCCA

File S1 WNV reference sequences

CGCACGAGATGTATTGGGTGAGTCGAGCTTCAGGCAATGTGGTACATTCAGTGAATATGACC
AGCCAGGTGCTCCTAGGAAGAATGGAAAAAAGGACCTGGAAGGGACCCCAATACGAGGAA
GATGTAAACTTGGGAAGTGGAACCAGGGCGGTGGGAAAACCCCTGCTCAACTCAGACACC
AGTAAAATCAAGAACAGGATTGAACGACTCAGGCGTGAGTACAGTTCGACGTGGCACCACG
ATGAGAACCACCCATATAGAACCTGGAACTATCACGGCAGTTATGATGTGAAGCCCACAGGC
TCCGCCAGTTCGCTGGTCAATGGAGTGGTCAGGCTCCTCTCAAAACCATGGGACACCATCA
CGAATGTTACCACCATGGCCATGACTGACACTACTCCCTTCGGGCAGCAGCGAGTGTTCAA
AGAGAAGGTGGACACGAAAGCTCCTGAACCGCCAGAAGGAGTGAAGTACGTGCTCAACGA
GACCACCAACTGGTTGTGGGCGTTTTTGGCCAGAGAAAAACGTCCCAGAATGTGCTCTCGA
GAGGAATTCATAAGAAAGGTCAACAGCAATGCAGCTTTGGGTGCCATGTTTGAAGAGCAGA
ATCAATGGAGGAGCGCCAGAGAAGCAGTTGAAGATCCAAAATTTTGGGAGATGGTGGATGA
GGAGCGCGAGGCACATCTGCGGGGGGAATGTCACACTTGCATTTACAACATGATGGGAAA
GAGAGAGAAAAAACCCGGAGAGTTCGGAAAGGCCAAGGGAAGCAGAGCCATTTGGTTCAT
GTGGCTCGGAGCTCGCTTTCTGGAGTTCGAGGCTCTGGGTTTTCTCAATGAAGACCACTG
GCTTGGAAGAAAGAACTCAGGAGGAGGTGTCGAGGGCTTGGGCCTCCAAAAACTGGGTTA
CATCCTGCGTGAAGTTGGCACCCGGCCTGGGGGCAAGATCTATGCTGATGACACAGCTGG
CTGGGACACCCGCATCACGAGAGCTGACTTGGAAAATGAAGCTAAGGTGCTTGAGCTGCTT
GATGGGGAACATCGGCGTCTTGCCAGGGCCATCATTGAGCTCACCTATCGTCACAAAGTTG
TGAAAGTGATGCGCCCGGCTGCTGATGGAAGAACCGTCATGGATGTTATCTCCAGAGAAGA
TCAGAGGGGGAGTGGACAAGTTGTCACCTACGCCCTAAACACTTTCACCAACCTGGCCGT
CCAGCTGGTGAGGATGATGGAAGGGGAAGGAGTGATTGGCCCAGATGATGTGGAGAAACT
CACAAAAGGGAAAGGACCCAAAGTCAGGACCTGGCTGTTTGAGAATGGGGAAGAAAGACT
CAGCCGCATGGCTGTCAGTGGAGATGACTGTGTGGTAAAGCCCCTGGACGATCGCTTTGC
CACCTCGCTCCACTTCCTCAATGCTATGTCAAAGGTTCGCAAAGACATCCAAGAGTGGAAA
CCGTCAACTGGATGGTATGATTGGCAGCAGGTTCCATTTTGCTCAAACCATTTCACTGAATT
GATCATGAAAGATGGAAGAACACTGGTGGTTCCATGCCGAGGACAGGATGAATTGGTAGGC
AGAGCTCGCATATCTCCAGGGGCCGGATGGAACGTCCGCGACACTGCTTGTCTGGCTAAG
TCTTATGCCCAGATGTGGCTGCTTCTGTACTTCCACAGAAGAGACCTGCGGCTCATGGCCA
ACGCCATTTGCTCCGCTGTCCCTGTGAATTGGGTCCCTACCGGAAGAACCACGTGGTCCAT
CCATGCAGGAGGAGAGTGGATGACAACAGAGGACATGTTGGAGGTCTGGAACCGTGTTTG
GATAGAGGAGAATGAATGGATGGAAGACAAAACCCCAGTGGAGAAATGGAGTGACGTCCCA
TATTCAGGAAAACGAGAGGACATCTGGTGTGGCAGCCTGATTGGCACAAGAGCCCGAGCC
ACGTGGGCAGAAAACATCCAGGTGGCTATCAACCAAGTCAGAGCAATCATCGGAGATGAGA
AGTATGTGGACTACATGAGTTCACTAAAGAGATATGAAGACACAACTTTGGTTGAGGACACA
GTACTGTAGATATTTAATCAATTGTAAATAGACAATATAAGTATGCATAAAAGTGTAGTTTTATAG
TAGTATTTAGTGGTGTTAGTGTAAATAGTTAAGAAAATTTTGAGGAGAAAGTCAGGCCGGGAA
GTTCCCGCCACCGGAAGTTGAGTAGACGGTGCTGCCTGCGACTCAACCCCAGGAGGACTG
GGTGAACAAAGCCGCGAAGTGATCCATGTAAGCCCTCAGAACCGTCTCGGAAGGAGGACC
CCACATGTTGTAACTTCAAAGCCCAATGTCAGACCACGCTACGGCGTGCTACTCTGCGGAG
AGTGCAGTCTGCGATAGTGCCCCAGGAGGACTGGGTTAACAAAGGCAAACCAACGCCCCA
CGCGGCCCTAGCCCCGGTAATGGTGTTAACCAGGGCGAAAGGACTAGAGGTTAGAGGAGA
CCCCGCGGTTTAAAGTGCACGGCCCAGCCTGGCTGAAGCTGTAGGTCAGGGGAAGGACTA
GAGGTTAGTGGAGACCCCGTGCCACAAAACACCACAACAAAACAGCATATTGACACCTGGG
ATAGACTAGGAGATCTTCTGCTCTGCACAACCAGCCACACGGCACAGTGCGCCGACAATGG
TGGCTGGTGGTGCGAGAACACAGGATCT
