# Supplementary material for: West Nile virus vaccine candidates attenuated by dinucleotide enrichment are immunogenic and protective against lethal infection
Source: PLoS Pathog. 2025 Oct 3;21(10):e1013560. doi: 10.1371/journal.ppat.1013560 (PMC12513643; doi:10.1371/journal.ppat.1013560)
Supplement: S2 File — (PDF) [file ppat.1013560.s005.pdf]

File S2 ISA fragments

WNV-WT

WNV NY99 sequences [GenBank: DQ211652.1].
Highlighted in red – pCMV promoter sequence.
Highlighted in green – HDR/SV40pA sequence.

>wt-NY99_**Fragment-I**
CACCCAACTGATCTTCAGCATCTTCAATATTGGCCATTAGCCATATTATTCATTGGTTATA
TAGCATAAATCAATATTGGCTATTGGCCATTGCATACGTTGTATCTATATCATAATATGTAC
ATTTATATTGGCTCATGTCCAATATGACCGCCATGTTGGCATTGATTATTGACTAGTTATT
AATAGTAATCAATTACGGGGTCATTAGTTCATAGCCCATATATGGAGTTCCGCGTTACATA
ACTTACGGTAAATGGCCCGCCTGGCTGACCGCCCAACGACCCCCGCCCATTGACGTCAA
TAATGACGTATGTTCCCATAGTAACGCCAATAGGGACTTTCCATTGACGTCAATGGGTGG
AGTATTTACGGTAAACTGCCCACTTGGCAGTACATCAAGTGTATCATATGCCAAGTCCGC
CCCCTATTGACGTCAATGACGGTAAATGGCCCGCCTGGCATTATGCCCAGTACATGACCT
TACGGGACTTTCCTACTTGGCAGTACATCTACGTATTAGTCATCGCTATTACCATGGTGAT
GCGGTTTTGGCAGTACACCAATGGGCGTGGATAGCGGTTTGACTCACGGGGATTTCCAA
GTCTCCACCCCATTGACGTCAATGGGAGTTTGTTTTGGCACCAAAATCAACGGGACTTT
CCAAAATGTCGTAATAACCCCGCCCCGTTGACGCAAATGGGCGGTAGGCGTGTACGGTG
GGAGGTCTATATAAGCAGAGCTCGTTTAGTGAACCGAGTAGTTCGCCTGTGTGAGCTGAC
AAACTTAGTAGTGTTTGTGAGGATTAACAACAATTAACACAGTGCGAGCTGTTTCTTAGCACG
AAGATCTCGATGTCTAAGAAACCAGGAGGGCCCGGCAAGAGCCGGGCTGTCAATATGCTAAA
ACGCGGAATGCCCCGCGTGTTGTCCTTGATTGGACTGAAGAGGGCTATGTTGAGCCTGATCGA
CGGCAAGGGGCCAATACGATTTGTGTTGGCTCTCTTGGCGTTCTTCAGGTTCACAGCAATTGC
TCCGACCCGAGCAGTGCTGGATCGATGGAGAGGTGTGAACAAACAAACAGCGATGAAACAC
CTTCTGAGTTTTAAGAAGGAACTAGGGACCTTGACCAGTGCTATCAATCGGCGGAGCTCAAA
ACAAAAGAAAAGAGGAGGAAAGACCGGAATTGCAGTCATGATTGGCCTGATCGCCAGCGTA
GGAGCAGTTACCCTCTCTAACTTCCAAGGGAAGGTGATGATGACGGTAAATGCTACTGACGTC
ACAGATGTCATCACGATTCCAACAGCTGCTGGAAAGAACCTATGCATTGTCAGAGCAATGGAT
GTGGGATACATGTGCGATGATACTATCACTTATGAATGCCCAGTACTGTCGGCTGGTAATGATC
CAGAAGACATCGACTGTTGGTGCACAAAGTCAGCAGTCTACGTCAGGTATGGAAGATGCACC
AAGACACGCCACTCAAGACGCAGTCGGAGGTCACTGACAGTGCAGACACACGGAGAAAGC
ACTCTAGCGAACAAGAAGGGGGCTTGGATGGACAGCACCAAGGCCACAAGGTATTTGGTAAA
AACAGAATCATGGATCTTGAGGAACCCTGGATATGCCCTGGTGGCAGCCGTCATTGGTTGGAT
GCTTGGGAGCAACACCATGCAGAGAGTTGTGTTTGTCGTGCTATTGCTTTTGGTGGCCCCAGC
TTACAGCTTCAACTGCCTTGGAATGAGCAACAGAGACTTCTTGGAAGGAGTGTCTGGAGCAA
CATGGGTGGATTTGGTTCTCGAAGGCGACAGCTGCGTGACTATCATGTCTAAGGACAAGCCTA
CCATCGATGTGAAGATGATGAATATGGAGGCGGCCAACCTGGCAGAGGTCCGCAGTTATTGCT
ATTTGGCTACCGTCAGCGATCTCTCCACCAAAGCTGCGTGCCCGACCATGGGAGAAGCTCAC
AATGACAAACGTGCTGACCCAGCTTTTGTGTGCAGACAAGGAGTGGTGGACAGGGGCTGGG
GCAACGGCTGCGGACTATTTGGCAAAGGAAGCATTGACACATGCGCCAAATTTGCCTGCTCTA
CCAAGGCAATAGGAAGAACCATCTTGAAAGAGAATATCAAGTACGAAGTGGCCATTTTTGTCC
ATGGACCAACTACTGTGGAGTCGCACGGAAACTACTCCACACAGGTTGGAGCCACTCAGGCA
GGGAGACTCAGCATCACTCCTGCGGCGCCTTCATACACACTAAAGCTTGGAGAATATGGAGA
GGTGACAGTGGACTGTGAACCACGGTCAGGGATTGACACCAATGCATACTACGTGATGACTG
TTGGAACAAAGACGTTCTTGGTCCATCGTGAGTGGTTCATGGACCTCAACCTCCCTTGGAGCA
GTGCTGGAAGTACTGTGTGGAGGAACAGAGAGACGTTAATGGAGTTTGAGGAACCACACGC
CACGAAGCAGTCTGTGATAGCATTGGGCTCACAAGAGGGAGCTCTGCATCAAGCTTTGGCTG

File S2 ISA fragments

GAGCCATTCCTGTGGAATTTTCAAGCAACACTGTCAAGTTGACGTCGGGTCATTTGAAGTGTA
GAGTGAAGATGGAAAAATTGCAGTTGAAGGGAACAACCTATGGCGTCTGTTCAAAGGCTTTC
AAGTTTCTTGGGACTCCCGCAGACACAGGTCACGGCACTGTGGTGTTGGAATTGCAGTACAC
TGGCACGGATGGACCTTGCAAAGTTCCTATCTCGTCAGTGGCTTCATTGAACGACCTAACGCC
AGTGGGCAGATTGGTCACTGTCAACCCTTTTGTTTCAGTGGCCACGGCCAACGCTAAGGTCCT
GATTGAATTGGAACCACCCTTTGGAGACTCATACATAGTGGTGGGCAGAGGAGAACAACAGA
TCAATCACCATTGGCACAAGTCTGGAAGCAGCATTGGCAAAGCCTTTACAACCACCCTCAAA
GGAGCGCAGAGACTAGCCGCTCTAGGAGACACAGCTTGGGACTTTGGATCAGTTGGAGGGGT
GTTCACCTCAGTTGGGAAGGCTGTCCATCAAGTGTTCGGAGGAGCATTCCGCTCACTGTTCGG
AGGCATGTCCTGGATAACGCAAGGATTGCTGGGGGCTCTCCTGTTGTGGATGGGCATCAATGC
TCGTGATAGGTCCATAGCTCTCACGTTTCTCGCAGTTGGAGGAGTTCTGCTCTTCCTCTCCGTG
AACGTGCACGCTGACACTGGGTGTGCCATAGACATCAGCCGGCAAGAGCTGAGATGTGGAAG
TGGAGTGTTCATACACAATGATGTGGAGGCTTGGATGGACCGGTACAAGTATTACCCTGAAAC
GCCACAAGGCCTAGCCAAGATCATTCAGAAAGCTCATAAGGAAGGAGTGTGCGGTCTACGAT
CAGTTTCCAGACTGGAGCATCAAATGTGGGAAGCAGTGAAGGACGAGCTGAACACTCTTTTG
AAGGAGAATGGTGTGGACCTTAGTGTCGTGGTTGAGAAACAGGAGGGAATGTACAAGTCAG
CACCTAAACGCCTCACCGCCACCACGGAAAAATTGGAAATTGGCTGGAAGGCCTGGGGAAA
GAGTATTTTATTTGCACCAGAACTCGCCAACAACACCTTTGTGGTTGATGGTCCGGAGACCAA
GGAATGTCCGACTCAGAATCGCGCTTGGAATAGCTTAGAAGTGGAGGATTTTGGATTTGGTCT
CACCAGCACTCGGATGTTCCTGAAGGTCAGAGAGAGCAACACAACTGAATGTGACTCGAAG
ATCATTGGAACGGCTGTCAAGAACAACTTGGCGATCCACAGTGACCTGTCCTATTGGATTGAA
AGCAGGCTCAATGATACGTGGAAGCTTGAAAGGGCAGTTCTGGGTGAAGTCAAATCATGTAC
GTGGCCTGAGACGCATACCTTGTGGGGCGATGGAATCCTTGAGAGTGACTTGATAATACCAGT
CACACTGGCGGGACCACGAAGCAATCACAATCGGAGACCTGGGTACAAGACACAAAACCAG
GGCCCATGGGACGAAGGCCGGGTAGAGATTGACTTCGATTACTGCCCAGGAACTACGGTCAC
CCTGAGTGAGAGCTGCGGACACCGTGGACCTGCCACTCGCACCACCACAGAGAGCGGAAAG
TTGATAACAGATTGGTGCTGCAGGAGCTGCACCTTACCACCACTGCGCTACCAAACTGACAGC
GGCTGTTGGTATGGTATGGAGATCAGACCACAGAGACATGATGAAAAGACCCTCGTGCAGTC
ACAAGTGAATGCTTATAATGCTGATATGATTGACCCTTTTCAGTTGGGCCTTCTGGTCGTGTTC
TTGGCCACCCAGGAGGTCCTTCGC

>wt-NY99_**Fragment-II**
TATAATGCTGATATGATTGACCCTTTTCAGTTGGGCCTTCTGGTCGTGTTCTTGGCCACCCAGG
AGGTCCTTCGCAAGAGGTGGACAGCCAAGATCAGCATGCCAGCTATACTGATTGCTCTGCTAG
TCCTGGTGTTTGGGGGCATTACTTACACTGATGTGTTACGCTATGTCATCTTGGTGGGGGCAGC
TTTCGCAGAATCTAATTCGGGAGGAGACGTGGTACACTTGGCGCTCATGGCGACCTTCAAGAT
ACAACCAGTGTTTATGGTGGCATCGTTTCTCAAAGCGAGATGGACCAACCAGGAGAACATTTT
GTTGATGTTGGCGGCTGTTTTCTTTCAAATGGCTTATCACGATGCCCGCCAAATTCTGCTCTGG
GAGATCCCTGATGTGTTGAATTCACTGGCGGTAGCTTGGATGATACTGAGAGCCATAACATTCA
CAACGACATCAAACGTGGTTGTTCCGCTGCTAGCCCTGCTAACACCCGGGCTGAGATGCTTGA
ATCTGGATGTGTACAGGATACTGCTGTTGATGGTCGGAATAGGCAGCTTGATCAGGGAGAAGA
GGAGTGCAGCCGCAAAAAAGAAAGGAGCAAGTCTGCTATGCTTGGCTCTAGCCTCAACAGG
ACTTTTCAACCCCATGATCCTTGCTGCTGGACTGATTGCATGTGATCCCAACCGTAAACGCGG
ATGGCCCGCAACTGAAGTGATGACAGCTGTCGGCCTAATGTTTGCCATCGTCGGAGGGCTGGC
AGAGCTTGACATTGACTCCATGGCCATTCCAATGACTATCGCGGGGCTCATGTTTGCTGCTTTC
GTGATTTCTGGGAAATCAACAGATATGTGGATTGAGAGAACGGCGGACATTTCCTGGGAAAGT
GATGCAGAAATTACAGGCTCGAGCGAAAGAGTTGATGTGCGGCTTGATGATGATGGAAACTT

File S2 ISA fragments

CCAGCTCATGAATGATCCAGGAGCACCTTGGAAGATATGGATGCTCAGAATGGTCTGTCTCGC
GATTAGTGCGTACACCCCCTGGGCAATCTTGCCCTCAGTAGTTGGATTTTGGATAACTCTCCAA
TACACAAAGAGAGGAGGCGTGTTGTGGGACACTCCCTCACCAAAGGAGTACAAAAAGGGGG
ACACGACCACCGGCGTCTACAGGATCATGACTCGTGGGCTGCTCGGCAGTTATCAAGCAGGA
GCGGGCGTGATGGTTGAAGGTGTTTTCCACACCCTTTGGCATACAACAAAAGGAGCCGCTTT
GATGAGCGGAGAGGGCCGCCTGGACCCATACTGGGGCAGTGTCAAGGAGGATCGACTTTGTT
ACGGAGGACCCTGGAAATTGCAGCACAAGTGGAACGGGCAGGATGAGGTGCAGATGATTGT
GGTGGAACCTGGCAAGAACGTTAAGAACGTCCAGACGAAACCAGGGGTGTTCAAAACACCT
GAAGGAGAAATCGGGGCCGTGACTTTGGACTTCCCCACTGGAACATCAGGCTCACCAATAGT
GGACAAAAACGGTGATGTGATTGGGCTTTATGGCAATGGAGTCATAATGCCCAACGGCTCATA
CATAAGCGCGATAGTGCAGGGTGAAAGGATGGATGAGCCAATCCCAGCCGGATTCGAACCTG
AGATGCTGAGGAAAAAACAGATCACTGTACTGGATCTCCATCCCGGCGCCGGTAAAACAAGG
AGGATTCTGCCACAGATCATCAAAGAGGCCATAAACAGAAGACTGAGAACAGCCGTGCTAGC
GCCAACCAGGGTTGTGGCTGCTGAGATGGCTGAAGCACTGAGAGGACTGCCCATCCGGTACC
AGACATCCGCAGTGCCCAGAGAACATAATGGAAATGAGATTGTTGATGTCATGTGTCATGCTA
CCCTCACCCACAGGCTGATGTCTCCTCACAGGGTGCCGAACTACAACCTGTTCGTGATGGATG
AGGCTCATTTCACCGACCCAGCTAGCATTGCAGCAAGAGGTTACATTTCCACAAAGGTCGAG
CTAGGGGAGGCGGCGGCAATATTCATGACAGCCACCCCACCAGGCACTTCAGATCCATTCCCA
GAGTCCAATTCACCAATTTCCGACTTACAGACTGAGATCCCGGATCGAGCTTGGAACTCTGGA
TACGAATGGATCACAGAATACACCGGGAAGACGGTTTGGTTTGTGCCTAGTGTCAAGATGGG
GAATGAGATTGCCCTTTGCCTACAACGTGCTGGAAAGAAAGTAGTCCAATTGAACAGAAAGT
CGTACGAGACGGAGTACCCAAAATGTAAGAACGATGATTGGGACTTTGTTATCACAACAGAC
ATATCTGAAATGGGGGCTAACTTCAAGGCGAGCAGGGTGATTGACAGCCGGAAGAGTGTGAA
ACCAACCATCATAACAGAAGGAGAAGGGAGAGTGATCCTGGGAGAACCATCTGCAGTGACA
GCAGCTAGTGCCGCCCAGAGACGTGGACGTATCGGTAGAAATCCGTCGCAAGTTGGTGATGA
GTACTGTTATGGGGGGCACACGAATGAAGACGACTCGAACTTCGCCCATTGGACTGAGGCAC
GAATCATGCTGGACAACATCAACATGCCAAACGGACTGATCGCTCAATTCTACCAACCAGAGC
GTGAGAAGGTATATACCATGGATGGGGAATACCGGCTCAGAGGAGAAGAGAGAAAAAACTTT
CTGGAACTGTTGAGGACTGCAGATCTGCCAGTTTGGCTGGCTTACAAGGTTGCAGCGGCTGG
AGTGTCATACCACGACCGGAGGTGGTGCTTTGATGGTCCTAGGACAAACACAATTTTAGAAG
ACAACAACGAAGTGGAAGTCATCACGAAGCTTGGTGAAAGGAAGATTCTGAGGCCGCGCTG
GATTGATGCCAGGGTGTACTCGGATCACCAGGCACTAAAGGCGTTCAAGGACTTCGCCTCGG
GAAAACGTTCTCAGATAGGGCTCATTGAGGTTCTGGGAAAGATGCCTGAGCACTTCATGGGG
AAGACATGGGAAGCACTTGACACCATGTACGTTGTGGCCACTGCAGAGAAAGGAGGAAGAG
CTCACAGAATGGCCCTGGAGGAACTGCCAGATGCTCTTCAGACAATTGCCTTGATTGCCTTAT
TGAGTGTGATGACCATGGGAGTATTCTTCCTCCTCATGCAGCGGAAGGGCATTGGAAAGATAG
GTTTGGGAGGCGCTGTCTTGGGAGTCGCGACCTTTTTCTGTTGGATGGCTGAAGTTCCAGGA
ACGAAGATCGCCGGAATGTTGCTGCTCTCCCTTCTCTTGATGATTGTGCTAATTCCTGAGCCAG
AGAAGCAACGTTCGCAGACAGACAACCAGCTAGCCGTGTTCCTGATTTGTGTCATGACCCTT
GTGAGCGCAGTGGCAGCCAACGAGATGGGTTGGCTAGATAAGACCAAGAGTGACATAAGCA
GTTTGTTTGGGCAAAGAATTGAGGTCAAGGAGAATTTCAGCATGGGAGAGTTTCTTCTGGAC
TTGAGGCCGGCAACAGCCTGGTCACTGTACGCTGTGACAACAGCGGTCCTCACTCCACTGCT
AAAGCATTTGATCACGTCAGATTACATCAACACCTCATTGACCTCAATAAACGTTCAGGCAAG
TGCACTATTCACACTCGCGCGAGGCTTCCCCTTCGTCGATGTTGGAGTGTCGGCTCTCCTGCT
AGCAGCCGGATGCTGGGGACAAGTCACCCTCACCGTTACGGTAACAGCGGCAACACTCCTTT
TTTGCCACTATGCCTACATGGTTCCCGGTTGGCAAGCTGAGGCAATGCGCTCAGCCCAGCGGC
GGACAGCGGCCGGAATCATGAAGAACGCTGTAGTGGATGGCATCGTGGCCACGGACGTCCCA

File S2 ISA fragments

GAATTAGAGCGCACCACACCCATCATGCAGAAGAAAGTTGGACAGATCATGCTGATCTTGGTG
TCTCTAGCTGCAGTAGTAGTGAACCCGTCTGTGAAGACAGTACGAGAAGCCGGAATTTTGATC
ACGGCCGCAGCGGTGACGCTTTGGGAGAATGGAGCAAGCTCTGTTTGGAACGCAACAACTG
CCATCGGACTCTGCCACATCATGCGTGGGGGTTGGTTGTCATGTCTATCCATAACATGGACACT
CATAAAGAACATGGAAAAACCAGGACTAAAAAGAGGTGGGGCAAAAGGACGCACCTTGGGA
GAGGTTTGGAAAGAAAGACTCAACCAGATGACAAAAGAAGAGTTCACT

>wt-NY99_**Fragment-III**
GGTGGGGCAAAAGGACGCACCTTGGGAGAGGTTTGGAAAGAAAGACTCAACCAGATGACAA
AAGAAGAGTTCACTAGGTACCGCAAAGAGGCCATCATCGAAGTCGATCGCTCAGCGGCAAAA
CACGCCAGGAAAGAAGGCAATGTCACTGGAGGGCATCCAGTCTCTAGGGGCACAGCAAAAC
TGAGATGGCTGGTCGAACGGAGGTTTCTCGAACCGGTCGGAAAAGTGATTGACCTTGGATGT
GGAAGAGGCGGTTGGTGTTACTATATGGCAACCCAAAAAAGAGTCCAAGAAGTCAGAGGGTA
CACAAAGGGCGGTCCCGGACATGAAGAGCCCCAACTAGTGCAAAGTTATGGATGGAACATTG
TCACCATGAAGAGTGGAGTGGATGTGTTCTACAGACCTTCTGAGTGTTGTGACACCCTCCTTT
GTGACATCGGAGAGTCCTCGTCAAGTGCTGAGGTTGAAGAGCATAGGACGATTCGGGTCCTT
GAAATGGTTGAGGACTGGCTGCACCGAGGGCCAAGGGAATTTTGCGTGAAGGTGCTCTGTCC
CTACATGCCGAAAGTCATAGAGAAGATGGAGCTGCTCCAACGCCGGTATGGGGGGGGACTGG
TCAGAAACCCACTCTCACGGAATTCCACGCACGAGATGTATTGGGTGAGTCGAGCTTCAGGC
AATGTGGTACATTCAGTGAATATGACCAGCCAGGTGCTCCTAGGAAGAATGGAAAAAAGGAC
CTGGAAGGGACCCCAATACGAGGAAGATGTAAACTTGGGAAGTGGAACCAGGGCGGTGGGA
AAACCCCTGCTCAACTCAGACACCAGTAAAATCAAGAACAGGATTGAACGACTCAGGCGTGA
GTACAGTTCGACGTGGCACCACGATGAGAACCACCCATATAGAACCTGGAACTATCACGGCA
GTTATGATGTGAAGCCCACAGGCTCCGCCAGTTCGCTGGTCAATGGAGTGGTCAGGCTCCTCT
CAAAACCATGGGACACCATCACGAATGTTACCACCATGGCCATGACTGACACTACTCCCTTCG
GGCAGCAGCGAGTGTTCAAAGAGAAGGTGGACACGAAAGCTCCTGAACCGCCAGAAGGAG
TGAAGTACGTGCTCAACGAGACCACCAACTGGTTGTGGGCGTTTTTGGCCAGAGAAAAACGT
CCCAGAATGTGCTCTCGAGAGGAATTCATAAGAAAGGTCAACAGCAATGCAGCTTTGGGTGC
CATGTTTGAAGAGCAGAATCAATGGAGGAGCGCCAGAGAAGCAGTTGAAGATCCAAAATTTT
GGGAGATGGTGGATGAGGAGCGCGAGGCACATCTGCGGGGGGAATGTCACACTTGCATTTAC
AACATGATGGGAAAGAGAGAGAAAAAACCCGGAGAGTTCGGAAAGGCCAAGGGAAGCAGA
GCCATTTGGTTCATGTGGCTCGGAGCTCGCTTTCTGGAGTTCGAGGCTCTGGGTTTTCTCAAT
GAAGACCACTGGCTTGGAAGAAAGAACTCAGGAGGAGGTGTCGAGGGCTTGGGCCTCCAAA
AACTGGGTTACATCCTGCGTGAAGTTGGCACCCGGCCTGGGGGCAAGATCTATGCTGATGACA
CAGCTGGCTGGGACACCCGCATCACGAGAGCTGACTTGGAAAATGAAGCTAAGGTGCTTGAG
CTGCTTGATGGGGAACATCGGCGTCTTGCCAGGGCCATCATTGAGCTCACCTATCGTCACAAA
GTTGTGAAAGTGATGCGCCCGGCTGCTGATGGAAGAACCGTCATGGATGTTATCTCCAGAGAA
GATCAGAGGGGGAGTGGACAAGTTGTCACCTACGCCCTAAACACTTTCACCAACCTGGCCGT
CCAGCTGGTGAGGATGATGGAAGGGGAAGGAGTGATTGGCCCAGATGATGTGGAGAAACTC
ACAAAAGGGAAAGGACCCAAAGTCAGGACCTGGCTGTTTGAGAATGGGGAAGAAAGACTCA
GCCGCATGGCTGTCAGTGGAGATGACTGTGTGGTAAAGCCCCTGGACGATCGCTTTGCCACCT
CGCTCCACTTCCTCAATGCTATGTCAAAGGTTCGCAAAGACATCCAAGAGTGGAAACCGTCA
ACTGGATGGTATGATTGGCAGCAGGTTCCATTTTGCTCAAACCATTTCACTGAATTGATCATGA
AAGATGGAAGAACACTGGTGGTTCCATGCCGAGGACAGGATGAATTGGTAGGCAGAGCTCGC
ATATCTCCAGGGGCCGGATGGAACGTCCGCGACACTGCTTGTCTGGCTAAGTCTTATGCCCAG
ATGTGGCTGCTTCTGTACTTCCACAGAAGAGACCTGCGGCTCATGGCCAACGCCATTTGCTCC
GCTGTCCCTGTGAATTGGGTCCCTACCGGAAGAACCACGTGGTCCATCCATGCAGGAGGAGA

File S2 ISA fragments

GTGGATGACAACAGAGGACATGTTGGAGGTCTGGAACCGTGTTTGGATAGAGGAGAATGAAT
GGATGGAAGACAAAACCCCAGTGGAGAAATGGAGTGACGTCCCATATTCAGGAAAACGAGA
GGACATCTGGTGTGGCAGCCTGATTGGCACAAGAGCCCGAGCCACGTGGGCAGAAAACATCC
AGGTGGCTATCAACCAAGTCAGAGCAATCATCGGAGATGAGAAGTATGTGGACTACATGAGTT
CACTAAAGAGATATGAAGACACAACTTTGGTTGAGGACACAGTACTGTAGATATTTAATCAAT
TGTAAATAGACAATATAAGTATGCATAAAAGTGTAGTTTTATAGTAGTATTTAGTGGTGTTAGTG
TAAATAGTTAAGAAAATTTTGAGGAGAAAGTCAGGCCGGGAAGTTCCCGCCACCGGAAGTTG
AGTAGACGGTGCTGCCTGCGACTCAACCCCAGGAGGACTGGGTGAACAAAGCCGCGAAGTG
ATCCATGTAAGCCCTCAGAACCGTCTCGGAAGGAGGACCCCACATGTTGTAACTTCAAAGCC
CAATGTCAGACCACGCTACGGCGTGCTACTCTGCGGAGAGTGCAGTCTGCGATAGTGCCCCA
GGAGGACTGGGTTAACAAAGGCAAACCAACGCCCCACGCGGCCCTAGCCCCGGTAATGGTGT
TAACCAGGGCGAAAGGACTAGAGGTTAGAGGAGACCCCGCGGTTTAAAGTGCACGGCCCAG
CCTGGCTGAAGCTGTAGGTCAGGGGAAGGACTAGAGGTTAGTGGAGACCCCGTGCCACAAA
ACACCACAACAAAACAGCATATTGACACCTGGGATAGACTAGGAGATCTTCTGCTCTGCACAA
CCAGCCACACGGCACAGTGCGCCGACAATGGTGGCTGGTGGTGCGAGAACACAGGATCT**GG
CCGGCATGGTCCCAGCCTCCTCGCTGGCGCCGGCTGGGCAACATTCCGAGGGGACCGT
CCCCTCGGTAATGGCGAATGGGACTCGCGACAGACATGATAAGATACATTGATGAGTTT
GGACAAACCACAACTAGAATGCAGTGAAAAAAATGCTTTATTTGTGAAATTAAGCGCTG
GCATTGACCCTGAG**

File S2 ISA fragments

E/NS1-Per

Highlighted in red – pCMV promoter sequence.
Highlighted in green – HDR/SV40pA sequence.

In brown are 22 nt adapters applied during synthesis of DNA without using bacterial plasmids and bacteria.

>NY99-Pr-E-NS1_**Fragment-I-A**
caatccgccctcactacaaccg**CACCCAACTGATCTTCAGCATCTTCAATATTGGCCATTAGCCATATT
ATTCATTGGTTATATAGCATAAATCAATATTGGCTATTGGCCATTGCATACGTTGTATCTAT
ATCATAATATGTACATTTATATTGGCTCATGTCCAATATGACCGCCATGTTGGCATTGATT
ATTGACTAGTTATTAATAGTAATCAATTACGGGGTCATTAGTTCATAGCCCATATATGGAG
TTCCGCGTTACATAACTTACGGTAAATGGCCCGCCTGGCTGACCGCCCAACGACCCCCG
CCCATTGACGTCAATAATGACGTATGTTCCCATAGTAACGCCAATAGGGACTTTCCATTG
ACGTCAATGGGTGGAGTATTTACGGTAAACTGCCCACTTGGCAGTACATCAAGTGTATC
ATATGCCAAGTCCGCCCCCTATTGACGTCAATGACGGTAAATGGCCCGCCTGGCATTATG
CCCAGTACATGACCTTACGGGACTTTCCTACTTGGCAGTACATCTACGTATTAGTCATCG
CTATTACCATGGTGATGCGGTTTTGGCAGTACACCAATGGGCGTGGATAGCGGTTTGAC
TCACGGGGATTTCCAAGTCTCCACCCCATTGACGTCAATGGGAGTTTGTTTTGGCACCA
AAATCAACGGGACTTTCCAAAATGTCGTAATAACCCCGCCCCGTTGACGCAAATGGGCG
GTAGGCGTGTACGGTGGGAGGTCTATATAAGCAGAGCTCGTTTAGTGAACCG**AGTAGTT
CGCCTGTGTGAGCTGACAAACTTAGTAGTGTTTGTGAGGATTAACAACAATTAACACAGTGCG
AGCTGTTTCTTAGCACGAAGATCTCGATGTCTAAGAAACCAGGAGGGCCCGGCAAGAGCCGG
GCTGTCAATATGCTAAAACGCGGAATGCCCCGCGTGTTGTCCTTGATTGGACTGAAGAGGGCT
ATGTTGAGCCTGATCGACGGCAAGGGGCCAATACGATTTGTGTTGGCTCTCTTGGCGTTCTTC
AGGTTCACAGCAATTGCTCCGACCCGAGCAGTGCTGGATCGATGGAGAGGTGTGAACAAACA
AACAGCGATGAAACACCTTCTGAGTTTTAAGAAGGAACTAGGGACCTTGACCAGTGCTATCA
ATCGGCGGAGCTCAAAACAAAAGAAAAGAGGAGGAAAGACCGGAATTGCAGTCATGAT***ctact
ctggcgtcgatgaggga***

>NY99-Pr-E-NS1_**Fragment-I-B**
caatccgccctcactacaaccgTGACCAGTGCTATCAATCGGCGGAGCTCAAAACAAAAGAAAAGAGGA
GGAAAGACCGGAATTGCAGTCATGATTGGCCTGATCGCCAGCGTAGGAGCAGTTACCCTCTCT
AACTTCCAAGGGAAGGTGATGATGACGGTAAATGCTACTGACGTCACAGATGTCATCACGATT
CCAACAGCTGCTGGAAAGAACCTATGCATTGTCAGAGCAATGGATGTGGGATACATGTGCGAT
GATACTATCACTTATGAATGCCCAGTACTGTCGGCTGGTAATGATCCAGAAGACATCGACTGTT
GGTGCACAAAGTCAGCAGTCTACGTCAGGTATGGAAGATGCACCAAGACACGCCACTCAAGA
CGCAGTCGGAGGTCACTGACAGTGCAGACACACGGAGAAAGCACTCTAGCGAACAAGAAGG
GGGCTTGGATGGACAGCACCAAGGCCACAAGGTATTTGGTAAAAACAGAATCATGGATCTTG
AGGAACCCTGGATATGCCCTGGTGGCAGCCGTCATTGGTTGGATGCTTGGGAGCAACACCATG
CAGAGAGTTGTGTTTGTCGTGCTATTGCTTTTGGTGGCCCCAGCTTACAGCTTCAACTGTCTG
GGCATGAGCAATAGAGACTTTTTGGAAGGAGTCTCCGGAGCAACCTGGGTGGATCTGGTCCT
CGAAGGCGACAGCTGCGTGACTATCATGTCCAAGGATAAGCCCACCATCGATGTGAAGATGAT
GAACATGGAGGCTGCCAATCTGGCGGAGGTTCGAAGCTATTGTTATTTGGCAACCGTCAGTGA
TCTCTCCACTAAAGCTGCCTGCCCGACCATGGGAGAAGCGCACAACGACAAACGTGCTGACC

CTGCATTTGTGTGCAGACAAGGAGTGGTGGACAGGGGCTGGGGCAACGGATGCGGCCTTTTT
GGGAAAGGCAGCATTGACACATGCGCCAAATTTGCTTGTTCCACTAAAGCAATAGGGAGAAC
CATTTTGAAAGAGAACATCAAGTACGAAGTGGCTATCTTTGTCCATGGACCAACTACAGTGGA
GTCTCACGGCAACTACTCCACCCAGGTTGGAGCCACACAGGCAGGAAGACTCAGCATCACAC
CAGCAGCTCCATCATA***ctactctggcgtcgatgaggga***

> NY99-Pr-E-NS1_**Fragment-I-C**
caatccgccctcactacaaccgCGGCAACTACTCCACCCAGGTTGGAGCCACACAGGCAGGAAGACTCA
GCATCACACCAGCAGCTCCATCATACACACTAAAACTTGGAGAATATGGCGAGGTCACAGTTG
ACTGTGAACCTCGGTCAGGAATTGACACCAATGCCTACTACGTGATGACTGTGGGAACTAAG
ACGTTCTTGGTTCACCGTGAGTGGTTCATGGACCTGAACCTGCCTTGGAGCAGCGCGGGAAG
CACAGTGTGGAGGAACCGAGAGACTTTGATGGAGTTCGAGGAGCCGCACGCCACTAAGCAG
TCTGTGATAGCCTTGGGCTCTCAGGAGGGAGCGCTGCACCAAGCATTGGCTGGAGCCATCCC
AGTTGAATTCTCCAGTAACACAGTCAAGTTGACGTCGGGTCATTTAAAATGTAGAGTGAAGAT
GGAAAAATTGCAGTTGAAAGGAACAACTTATGGAGTATGCTCAAAAGCGTTCAAGTTCCTTG
GGACACCCGCTGACACTGGACACGGCACGGTGGTCTTGGAATTGCAGTACACTGGCACGGAT
GGACCTTGCAAGGTTCCGATCTCGTCTGTGGCCTCGTTAAACGATTTGACGCCTGTGGGCAGA
TTGGTGACTGTCAATCCTTTTGTGTCAGTGGCCACTGCCAATGCTAAGGTCCTCATTGAGTTG
GAACCTCCATTTGGAGACTCATACATAGTGGTGGGCAGAGGAGAACAACAGATCAACCATCA
TTGGCACAAGTCTGGCAGCAGCATTGGCAAAGCTTTCACAACCACTCTCAAAGGAGCACAGA
GACTAGCCGCTCTAGGAGACACAGCCTGGGACTTTGGCTCAGTTGGAGGGGTCTTTACATCA
GTTGGGAAAGCTGTCCACCAGGTGTTTGGAGGAGCTTTCAGATCACTGTTCGGAGGAATGTC
TTGGATAACTCAGGGCTTGCTGGGGGCTCTTCTGTTGTGGATGGGAATCAATGCGCGTGATAG
GTCAATAGCACTCACGTTCCTCGCAGTTGGAGGAGTTCTTCTGTTCCTGTCTGTGAACGTGCA
CGCTGACACGGGGTGTGCCATTGATATCAGCCGCCAAGAGCTGAGATGTGGAAGTGGGGTGT
TCATACACAACGATGTGGAGGCTTGGATGGATCGGTACAAGTATTACCCAGAAACG***ctactctggcgt
cgatgaggga***

> NY99-Pr-E-NS1_**Fragment-I-D**
caatccgccctcactacaaccgTGTGGAAGTGGGGTGTTCATACACAACGATGTGGAGGCTTGGATGGAT
CGGTACAAGTATTACCCAGAAACGCCACAAGGACTAGCCAAGATCATACAGAAGGCACATAA
AGAAGGCGTGTGCGGTCTGCGATCTGTTTCCAGGCTGGAACACCAAATGTGGGAGGCTGTGA
AGGATGAACTCAATACACTTTTGAAGGAAAACGGTGTGGATCTCAGTGTCGTGGTTGAGAAG
CAAGAGGGAATGTACAAGTCCGCTCCCAAGCGCCTGACGGCCACGACAGAAAAATTGGAAA
TTGGCTGGAAGGCCTGGGGTAAGAGCATTTTATTTGCACCGGAACTAGCCAACAACACCTTTG
TTGTGGATGGTCCTGAGACCAAAGAATGTCCGACTCAGAATCGTGCTTGGAACAGTTTGGAA
GTAGAGGATTTTGGATTCGGACTCACCAGTACTCGGATGTTCCTCAAGGTCAGAGAGAGCAA
CACAACAGAATGTGACTCTAAGATCATTGGAACTGCTGTGAAGAACAACTTGGCCATCCACA
GTGACCTGTCATATTGGATTGAGAGCAGGCTAAATGACACCTGGAAGCTGGAGAGGGCAGTA
CTTGGTGAGGTTAAATCCTGTACGTGGCCTGAGACCCATACATTGTGGGGCGATGGAATCCTC
GAGAGCGACTTGATAATTCCAGTCACACTGGCTGGACCTCGAAGCAACCACAATCGGAGACC
GGGGTACAAGACACAAAACCAGGGTCCCTGGGATGAAGGCCGAGTAGAAATTGACTTTGATT
ATTGCCCAGGAACCACAGTCACGCTGAGCGAAAGCTGTGGACACCGAGGCCCGGCCACTCG
CACTACGACAGAGAGTGGGAAGTTGATCACAGACTGGTGCTGCAGAAGCTGCACGTTACCAC
CACTTCGCTACCAAACTGACAGCGGCTGCTGGTATGGAATGGAGATCAGACCACAGAGACAT

File S2 ISA fragments

GATGAGAAGACCCTCGTGCAGTCACAAGTGAATGCTTATAATGCTGATATGATTGACCCTTTTC
AGTTGGGCCTTCTGGTCGTGTTCTTGGCCACCCAGGAGGTCCTTCGC***ctactctggcgtcgatgaggga***

>wt-NY99_**Fragment-II**
TATAATGCTGATATGATTGACCCTTTTCAGTTGGGCCTTCTGGTCGTGTTCTTGGCCACCCAGG
AGGTCCTTCGCAAGAGGTGGACAGCCAAGATCAGCATGCCAGCTATACTGATTGCTCTGCTAG
TCCTGGTGTTTGGGGGCATTACTTACACTGATGTGTTACGCTATGTCATCTTGGTGGGGGCAGC
TTTCGCAGAATCTAATTCGGGAGGAGACGTGGTACACTTGGCGCTCATGGCGACCTTCAAGAT
ACAACCAGTGTTTATGGTGGCATCGTTTCTCAAAGCGAGATGGACCAACCAGGAGAACATTTT
GTTGATGTTGGCGGCTGTTTTCTTTCAAATGGCTTATCACGATGCCCGCCAAATTCTGCTCTGG
GAGATCCCTGATGTGTTGAATTCACTGGCGGTAGCTTGGATGATACTGAGAGCCATAACATTCA
CAACGACATCAAACGTGGTTGTTCCGCTGCTAGCCCTGCTAACACCCGGGCTGAGATGCTTGA
ATCTGGATGTGTACAGGATACTGCTGTTGATGGTCGGAATAGGCAGCTTGATCAGGGAGAAGA
GGAGTGCAGCCGCAAAAAAGAAAGGAGCAAGTCTGCTATGCTTGGCTCTAGCCTCAACAGG
ACTTTTCAACCCCATGATCCTTGCTGCTGGACTGATTGCATGTGATCCCAACCGTAAACGCGG
ATGGCCCGCAACTGAAGTGATGACAGCTGTCGGCCTAATGTTTGCCATCGTCGGAGGGCTGGC
AGAGCTTGACATTGACTCCATGGCCATTCCAATGACTATCGCGGGGCTCATGTTTGCTGCTTTC
GTGATTTCTGGGAAATCAACAGATATGTGGATTGAGAGAACGGCGGACATTTCCTGGGAAAGT
GATGCAGAAATTACAGGCTCGAGCGAAAGAGTTGATGTGCGGCTTGATGATGATGGAAACTT
CCAGCTCATGAATGATCCAGGAGCACCTTGGAAGATATGGATGCTCAGAATGGTCTGTCTCGC
GATTAGTGCGTACACCCCCTGGGCAATCTTGCCCTCAGTAGTTGGATTTTGGATAACTCTCCAA
TACACAAAGAGAGGAGGCGTGTTGTGGGACACTCCCTCACCAAAGGAGTACAAAAAGGGGG
ACACGACCACCGGCGTCTACAGGATCATGACTCGTGGGCTGCTCGGCAGTTATCAAGCAGGA
GCGGGCGTGATGGTTGAAGGTGTTTTCCACACCCTTTGGCATACAACAAAAGGAGCCGCTTT
GATGAGCGGAGAGGGCCGCCTGGACCCATACTGGGGCAGTGTCAAGGAGGATCGACTTTGTT
ACGGAGGACCCTGGAAATTGCAGCACAAGTGGAACGGGCAGGATGAGGTGCAGATGATTGT
GGTGGAACCTGGCAAGAACGTTAAGAACGTCCAGACGAAACCAGGGGTGTTCAAAACACCT
GAAGGAGAAATCGGGGCCGTGACTTTGGACTTCCCCACTGGAACATCAGGCTCACCAATAGT
GGACAAAAACGGTGATGTGATTGGGCTTTATGGCAATGGAGTCATAATGCCCAACGGCTCATA
CATAAGCGCGATAGTGCAGGGTGAAAGGATGGATGAGCCAATCCCAGCCGGATTCGAACCTG
AGATGCTGAGGAAAAAACAGATCACTGTACTGGATCTCCATCCCGGCGCCGGTAAAACAAGG
AGGATTCTGCCACAGATCATCAAAGAGGCCATAAACAGAAGACTGAGAACAGCCGTGCTAGC
GCCAACCAGGGTTGTGGCTGCTGAGATGGCTGAAGCACTGAGAGGACTGCCCATCCGGTACC
AGACATCCGCAGTGCCCAGAGAACATAATGGAAATGAGATTGTTGATGTCATGTGTCATGCTA
CCCTCACCCACAGGCTGATGTCTCCTCACAGGGTGCCGAACTACAACCTGTTCGTGATGGATG
AGGCTCATTTCACCGACCCAGCTAGCATTGCAGCAAGAGGTTACATTTCCACAAAGGTCGAG
CTAGGGGAGGCGGCGGCAATATTCATGACAGCCACCCCACCAGGCACTTCAGATCCATTCCCA
GAGTCCAATTCACCAATTTCCGACTTACAGACTGAGATCCCGGATCGAGCTTGGAACTCTGGA
TACGAATGGATCACAGAATACACCGGGAAGACGGTTTGGTTTGTGCCTAGTGTCAAGATGGG
GAATGAGATTGCCCTTTGCCTACAACGTGCTGGAAAGAAAGTAGTCCAATTGAACAGAAAGT
CGTACGAGACGGAGTACCCAAAATGTAAGAACGATGATTGGGACTTTGTTATCACAACAGAC
ATATCTGAAATGGGGGCTAACTTCAAGGCGAGCAGGGTGATTGACAGCCGGAAGAGTGTGAA
ACCAACCATCATAACAGAAGGAGAAGGGAGAGTGATCCTGGGAGAACCATCTGCAGTGACA
GCAGCTAGTGCCGCCCAGAGACGTGGACGTATCGGTAGAAATCCGTCGCAAGTTGGTGATGA
GTACTGTTATGGGGGGCACACGAATGAAGACGACTCGAACTTCGCCCATTGGACTGAGGCAC
GAATCATGCTGGACAACATCAACATGCCAAACGGACTGATCGCTCAATTCTACCAACCAGAGC
GTGAGAAGGTATATACCATGGATGGGGAATACCGGCTCAGAGGAGAAGAGAGAAAAAACTTT

CTGGAACTGTTGAGGACTGCAGATCTGCCAGTTTGGCTGGCTTACAAGGTTGCAGCGGCTGG
AGTGTCATACCACGACCGGAGGTGGTGCTTTGATGGTCCTAGGACAAACACAATTTTAGAAG
ACAACAACGAAGTGGAAGTCATCACGAAGCTTGGTGAAAGGAAGATTCTGAGGCCGCGCTG
GATTGATGCCAGGGTGTACTCGGATCACCAGGCACTAAAGGCGTTCAAGGACTTCGCCTCGG
GAAAACGTTCTCAGATAGGGCTCATTGAGGTTCTGGGAAAGATGCCTGAGCACTTCATGGGG
AAGACATGGGAAGCACTTGACACCATGTACGTTGTGGCCACTGCAGAGAAAGGAGGAAGAG
CTCACAGAATGGCCCTGGAGGAACTGCCAGATGCTCTTCAGACAATTGCCTTGATTGCCTTAT
TGAGTGTGATGACCATGGGAGTATTCTTCCTCCTCATGCAGCGGAAGGGCATTGGAAAGATAG
GTTTGGGAGGCGCTGTCTTGGGAGTCGCGACCTTTTTCTGTTGGATGGCTGAAGTTCCAGGA
ACGAAGATCGCCGGAATGTTGCTGCTCTCCCTTCTCTTGATGATTGTGCTAATTCCTGAGCCAG
AGAAGCAACGTTCGCAGACAGACAACCAGCTAGCCGTGTTCCTGATTTGTGTCATGACCCTT
GTGAGCGCAGTGGCAGCCAACGAGATGGGTTGGCTAGATAAGACCAAGAGTGACATAAGCA
GTTTGTTTGGGCAAAGAATTGAGGTCAAGGAGAATTTCAGCATGGGAGAGTTTCTTCTGGAC
TTGAGGCCGGCAACAGCCTGGTCACTGTACGCTGTGACAACAGCGGTCCTCACTCCACTGCT
AAAGCATTTGATCACGTCAGATTACATCAACACCTCATTGACCTCAATAAACGTTCAGGCAAG
TGCACTATTCACACTCGCGCGAGGCTTCCCCTTCGTCGATGTTGGAGTGTCGGCTCTCCTGCT
AGCAGCCGGATGCTGGGGACAAGTCACCCTCACCGTTACGGTAACAGCGGCAACACTCCTTT
TTTGCCACTATGCCTACATGGTTCCCGGTTGGCAAGCTGAGGCAATGCGCTCAGCCCAGCGGC
GGACAGCGGCCGGAATCATGAAGAACGCTGTAGTGGATGGCATCGTGGCCACGGACGTCCCA
GAATTAGAGCGCACCACACCCATCATGCAGAAGAAAGTTGGACAGATCATGCTGATCTTGGTG
TCTCTAGCTGCAGTAGTAGTGAACCCGTCTGTGAAGACAGTACGAGAAGCCGGAATTTTGATC
ACGGCCGCAGCGGTGACGCTTTGGGAGAATGGAGCAAGCTCTGTTTGGAACGCAACAACTG
CCATCGGACTCTGCCACATCATGCGTGGGGGTTGGTTGTCATGTCTATCCATAACATGGACACT
CATAAAGAACATGGAAAAACCAGGACTAAAAAGAGGTGGGGCAAAAGGACGCACCTTGGGA
GAGGTTTGGAAAGAAAGACTCAACCAGATGACAAAAGAAGAGTTCACT

>wt-NY99_**Fragment-III**
GGTGGGGCAAAAGGACGCACCTTGGGAGAGGTTTGGAAAGAAAGACTCAACCAGATGACAA
AAGAAGAGTTCACTAGGTACCGCAAAGAGGCCATCATCGAAGTCGATCGCTCAGCGGCAAAA
CACGCCAGGAAAGAAGGCAATGTCACTGGAGGGCATCCAGTCTCTAGGGGCACAGCAAAAC
TGAGATGGCTGGTCGAACGGAGGTTTCTCGAACCGGTCGGAAAAGTGATTGACCTTGGATGT
GGAAGAGGCGGTTGGTGTTACTATATGGCAACCCAAAAAAGAGTCCAAGAAGTCAGAGGGTA
CACAAAGGGCGGTCCCGGACATGAAGAGCCCCAACTAGTGCAAAGTTATGGATGGAACATTG
TCACCATGAAGAGTGGAGTGGATGTGTTCTACAGACCTTCTGAGTGTTGTGACACCCTCCTTT
GTGACATCGGAGAGTCCTCGTCAAGTGCTGAGGTTGAAGAGCATAGGACGATTCGGGTCCTT
GAAATGGTTGAGGACTGGCTGCACCGAGGGCCAAGGGAATTTTGCGTGAAGGTGCTCTGTCC
CTACATGCCGAAAGTCATAGAGAAGATGGAGCTGCTCCAACGCCGGTATGGGGGGGGACTGG
TCAGAAACCCACTCTCACGGAATTCCACGCACGAGATGTATTGGGTGAGTCGAGCTTCAGGC
AATGTGGTACATTCAGTGAATATGACCAGCCAGGTGCTCCTAGGAAGAATGGAAAAAAGGAC
CTGGAAGGGACCCCAATACGAGGAAGATGTAAACTTGGGAAGTGGAACCAGGGCGGTGGGA
AAACCCCTGCTCAACTCAGACACCAGTAAAATCAAGAACAGGATTGAACGACTCAGGCGTGA
GTACAGTTCGACGTGGCACCACGATGAGAACCACCCATATAGAACCTGGAACTATCACGGCA
GTTATGATGTGAAGCCCACAGGCTCCGCCAGTTCGCTGGTCAATGGAGTGGTCAGGCTCCTCT
CAAAACCATGGGACACCATCACGAATGTTACCACCATGGCCATGACTGACACTACTCCCTTCG
GGCAGCAGCGAGTGTTCAAAGAGAAGGTGGACACGAAAGCTCCTGAACCGCCAGAAGGAG
TGAAGTACGTGCTCAACGAGACCACCAACTGGTTGTGGGCGTTTTTGGCCAGAGAAAAACGT
CCCAGAATGTGCTCTCGAGAGGAATTCATAAGAAAGGTCAACAGCAATGCAGCTTTGGGTGC

File S2 ISA fragments

CATGTTTGAAGAGCAGAATCAATGGAGGAGCGCCAGAGAAGCAGTTGAAGATCCAAAATTTT
GGGAGATGGTGGATGAGGAGCGCGAGGCACATCTGCGGGGGGAATGTCACACTTGCATTTAC
AACATGATGGGAAAGAGAGAGAAAAAACCCGGAGAGTTCGGAAAGGCCAAGGGAAGCAGA
GCCATTTGGTTCATGTGGCTCGGAGCTCGCTTTCTGGAGTTCGAGGCTCTGGGTTTTCTCAAT
GAAGACCACTGGCTTGGAAGAAAGAACTCAGGAGGAGGTGTCGAGGGCTTGGGCCTCCAAA
AACTGGGTTACATCCTGCGTGAAGTTGGCACCCGGCCTGGGGGCAAGATCTATGCTGATGACA
CAGCTGGCTGGGACACCCGCATCACGAGAGCTGACTTGGAAAATGAAGCTAAGGTGCTTGAG
CTGCTTGATGGGGAACATCGGCGTCTTGCCAGGGCCATCATTGAGCTCACCTATCGTCACAAA
GTTGTGAAAGTGATGCGCCCGGCTGCTGATGGAAGAACCGTCATGGATGTTATCTCCAGAGAA
GATCAGAGGGGGAGTGGACAAGTTGTCACCTACGCCCTAAACACTTTCACCAACCTGGCCGT
CCAGCTGGTGAGGATGATGGAAGGGGAAGGAGTGATTGGCCCAGATGATGTGGAGAAACTC
ACAAAAGGGAAAGGACCCAAAGTCAGGACCTGGCTGTTTGAGAATGGGGAAGAAAGACTCA
GCCGCATGGCTGTCAGTGGAGATGACTGTGTGGTAAAGCCCCTGGACGATCGCTTTGCCACCT
CGCTCCACTTCCTCAATGCTATGTCAAAGGTTCGCAAAGACATCCAAGAGTGGAAACCGTCA
ACTGGATGGTATGATTGGCAGCAGGTTCCATTTTGCTCAAACCATTTCACTGAATTGATCATGA
AAGATGGAAGAACACTGGTGGTTCCATGCCGAGGACAGGATGAATTGGTAGGCAGAGCTCGC
ATATCTCCAGGGGCCGGATGGAACGTCCGCGACACTGCTTGTCTGGCTAAGTCTTATGCCCAG
ATGTGGCTGCTTCTGTACTTCCACAGAAGAGACCTGCGGCTCATGGCCAACGCCATTTGCTCC
GCTGTCCCTGTGAATTGGGTCCCTACCGGAAGAACCACGTGGTCCATCCATGCAGGAGGAGA
GTGGATGACAACAGAGGACATGTTGGAGGTCTGGAACCGTGTTTGGATAGAGGAGAATGAAT
GGATGGAAGACAAAACCCCAGTGGAGAAATGGAGTGACGTCCCATATTCAGGAAAACGAGA
GGACATCTGGTGTGGCAGCCTGATTGGCACAAGAGCCCGAGCCACGTGGGCAGAAAACATCC
AGGTGGCTATCAACCAAGTCAGAGCAATCATCGGAGATGAGAAGTATGTGGACTACATGAGTT
CACTAAAGAGATATGAAGACACAACTTTGGTTGAGGACACAGTACTGTAGATATTTAATCAAT
TGTAAATAGACAATATAAGTATGCATAAAAGTGTAGTTTTATAGTAGTATTTAGTGGTGTTAGTG
TAAATAGTTAAGAAAATTTTGAGGAGAAAGTCAGGCCGGGAAGTTCCCGCCACCGGAAGTTG
AGTAGACGGTGCTGCCTGCGACTCAACCCCAGGAGGACTGGGTGAACAAAGCCGCGAAGTG
ATCCATGTAAGCCCTCAGAACCGTCTCGGAAGGAGGACCCCACATGTTGTAACTTCAAAGCC
CAATGTCAGACCACGCTACGGCGTGCTACTCTGCGGAGAGTGCAGTCTGCGATAGTGCCCCA
GGAGGACTGGGTTAACAAAGGCAAACCAACGCCCCACGCGGCCCTAGCCCCGGTAATGGTGT
TAACCAGGGCGAAAGGACTAGAGGTTAGAGGAGACCCCGCGGTTTAAAGTGCACGGCCCAG
CCTGGCTGAAGCTGTAGGTCAGGGGAAGGACTAGAGGTTAGTGGAGACCCCGTGCCACAAA
ACACCACAACAAAACAGCATATTGACACCTGGGATAGACTAGGAGATCTTCTGCTCTGCACAA
CCAGCCACACGGCACAGTGCGCCGACAATGGTGGCTGGTGGTGCGAGAACACAGGATCT**GG
CCGGCATGGTCCCAGCCTCCTCGCTGGCGCCGGCTGGGCAACATTCCGAGGGGACCGT
CCCCTCGGTAATGGCGAATGGGACTCGCGACAGACATGATAAGATACATTGATGAGTTT
GGACAAACCACAACTAGAATGCAGTGAAAAAAATGCTTTATTTGTGAAATTAAGCGCTG
GCATTGACCCTGAG**

File S2 ISA fragments

E/NS1/NS5-Per

Highlighted in red – pCMV promoter sequence.
Highlighted in green – HDR/SV40pA sequence.

In brown are 22 nt adapters applied during synthesis of DNA without using bacterial plasmids and bacteria.

>NY99-Pr-E-NS1_**Fragment-I-A**
*caatccgccctcactacaaccg***CACCCAACTGATCTTCAGCATCTTCAATATTGGCCATTAGCCATATT ATTCATTGGTTATATAGCATAAATCAATATTGGCTATTGGCCATTGCATACGTTGTATCTAT ATCATAATATGTACATTTATATTGGCTCATGTCCAATATGACCGCCATGTTGGCATTGATT ATTGACTAGTTATTAATAGTAATCAATTACGGGGTCATTAGTTCATAGCCCATATATGGAG TTCCGCGTTACATAACTTACGGTAAATGGCCCGCCTGGCTGACCGCCCAACGACCCCCG CCCATTGACGTCAATAATGACGTATGTTCCCATAGTAACGCCAATAGGGACTTTCCATTG ACGTCAATGGGTGGAGTATTTACGGTAAACTGCCCACTTGGCAGTACATCAAGTGTATC ATATGCCAAGTCCGCCCCCTATTGACGTCAATGACGGTAAATGGCCCGCCTGGCATTATG CCCAGTACATGACCTTACGGGACTTTCCTACTTGGCAGTACATCTACGTATTAGTCATCG CTATTACCATGGTGATGCGGTTTTGGCAGTACACCAATGGGCGTGGATAGCGGTTTGAC TCACGGGGATTTCCAAGTCTCCACCCCATTGACGTCAATGGGAGTTTGTTTTGGCACCA AAATCAACGGGACTTTCCAAAATGTCGTAATAACCCCGCCCCGTTGACGCAAATGGGCG GTAGGCGTGTACGGTGGGAGGTCTATATAAGCAGAGCTCGTTTAGTGAACCG**AGTAGTT CGCCTGTGTGAGCTGACAAACTTAGTAGTGTTTGTGAGGATTAACAACAATTAACACAGTGCG AGCTGTTTCTTAGCACGAAGATCTCGATGTCTAAGAAACCAGGAGGGCCCGGCAAGAGCCGG GCTGTCAATATGCTAAAACGCGGAATGCCCCGCGTGTTGTCCTTGATTGGACTGAAGAGGGCT ATGTTGAGCCTGATCGACGGCAAGGGGCCAATACGATTTGTGTTGGCTCTCTTGGCGTTCTTC AGGTTCACAGCAATTGCTCCGACCCGAGCAGTGCTGGATCGATGGAGAGGTGTGAACAAACA AACAGCGATGAAACACCTTCTGAGTTTTAAGAAGGAACTAGGGACCTTGACCAGTGCTATCA ATCGGCGGAGCTCAAAACAAAAGAAAAGAGGAGGAAAGACCGGAATTGCAGTCATGAT*ctact ctggcgtcgatgaggga*

>NY99-Pr-E-NS1_**Fragment-I-B**
*caatccgccctcactacaaccg*TGACCAGTGCTATCAATCGGCGGAGCTCAAAACAAAAGAAAAGAGGA GGAAAGACCGGAATTGCAGTCATGATTGGCCTGATCGCCAGCGTAGGAGCAGTTACCCTCTCT AACTTCCAAGGGAAGGTGATGATGACGGTAAATGCTACTGACGTCACAGATGTCATCACGATT CCAACAGCTGCTGGAAAGAACCTATGCATTGTCAGAGCAATGGATGTGGGATACATGTGCGAT GATACTATCACTTATGAATGCCCAGTACTGTCGGCTGGTAATGATCCAGAAGACATCGACTGTT GGTGCACAAAGTCAGCAGTCTACGTCAGGTATGGAAGATGCACCAAGACACGCCACTCAAGA CGCAGTCGGAGGTCACTGACAGTGCAGACACACGGAGAAAGCACTCTAGCGAACAAGAAGG GGGCTTGGATGGACAGCACCAAGGCCACAAGGTATTTGGTAAAAACAGAATCATGGATCTTG AGGAACCCTGGATATGCCCTGGTGGCAGCCGTCATTGGTTGGATGCTTGGGAGCAACACCATG CAGAGAGTTGTGTTTGTCGTGCTATTGCTTTTGGTGGCCCCAGCTTACAGCTTCAACTGTCTG GGCATGAGCAATAGAGACTTTTTGGAAGGAGTCTCCGGAGCAACCTGGGTGGATCTGGTCCT CGAAGGCGACAGCTGCGTGACTATCATGTCCAAGGATAAGCCCACCATCGATGTGAAGATGAT GAACATGGAGGCTGCCAATCTGGCGGAGGTTCGAAGCTATTGTTATTTGGCAACCGTCAGTGA TCTCTCCACTAAAGCTGCCTGCCCGACCATGGGAGAAGCGCACAACGACAAACGTGCTGACC

File S2 ISA fragments

CTGCATTTGTGTGCAGACAAGGAGTGGTGGACAGGGGCTGGGGCAACGGATGCGGCCTTTTT
GGGAAAGGCAGCATTGACACATGCGCCAAATTTGCTTGTTCCACTAAAGCAATAGGGAGAAC
CATTTTGAAAGAGAACATCAAGTACGAAGTGGCTATCTTTGTCCATGGACCAACTACAGTGGA
GTCTCACGGCAACTACTCCACCCAGGTTGGAGCCACACAGGCAGGAAGACTCAGCATCACAC
CAGCAGCTCCATCATA*ctactctggcgtcgatgaggga*

> NY99-Pr-E-NS1_**Fragment-I-C**
*caatccgccctcactacaaccg*CGGCAACTACTCCACCCAGGTTGGAGCCACACAGGCAGGAAGACTCA
GCATCACACCAGCAGCTCCATCATACACACTAAAACTTGGAGAATATGGCGAGGTCACAGTTG
ACTGTGAACCTCGGTCAGGAATTGACACCAATGCCTACTACGTGATGACTGTGGGAACTAAG
ACGTTCTTGGTTCACCGTGAGTGGTTCATGGACCTGAACCTGCCTTGGAGCAGCGCGGGAAG
CACAGTGTGGAGGAACCGAGAGACTTTGATGGAGTTCGAGGAGCCGCACGCCACTAAGCAG
TCTGTGATAGCCTTGGGCTCTCAGGAGGGAGCGCTGCACCAAGCATTGGCTGGAGCCATCCC
AGTTGAATTCTCCAGTAACACAGTCAAGTTGACGTCGGGTCATTTAAAATGTAGAGTGAAGAT
GGAAAAATTGCAGTTGAAAGGAACAACTTATGGAGTATGCTCAAAAGCGTTCAAGTTCCTTG
GGACACCCGCTGACACTGGACACGGCACGGTGGTCTTGGAATTGCAGTACACTGGCACGGAT
GGACCTTGCAAGGTTCCGATCTCGTCTGTGGCCTCGTTAAACGATTTGACGCCTGTGGGCAGA
TTGGTGACTGTCAATCCTTTTGTGTCAGTGGCCACTGCCAATGCTAAGGTCCTCATTGAGTTG
GAACCTCCATTTGGAGACTCATACATAGTGGTGGGCAGAGGAGAACAACAGATCAACCATCA
TTGGCACAAGTCTGGCAGCAGCATTGGCAAAGCTTTCACAACCACTCTCAAAGGAGCACAGA
GACTAGCCGCTCTAGGAGACACAGCCTGGGACTTTGGCTCAGTTGGAGGGGTCTTTACATCA
GTTGGGAAAGCTGTCCACCAGGTGTTTGGAGGAGCTTTCAGATCACTGTTCGGAGGAATGTC
TTGGATAACTCAGGGCTTGCTGGGGGCTCTTCTGTTGTGGATGGGAATCAATGCGCGTGATAG
GTCAATAGCACTCACGTTCCTCGCAGTTGGAGGAGTTCTTCTGTTCCTGTCTGTGAACGTGCA
CGCTGACACGGGGTGTGCCATTGATATCAGCCGCCAAGAGCTGAGATGTGGAAGTGGGGTGT
TCATACACAACGATGTGGAGGCTTGGATGGATCGGTACAAGTATTACCCAGAAACG*ctactctggcgt
cgatgaggga*

> NY99-Pr-E-NS1_**Fragment-I-D**
*caatccgccctcactacaaccg*TGTGGAAGTGGGGTGTTCATACACAACGATGTGGAGGCTTGGATGGAT
CGGTACAAGTATTACCCAGAAACGCCACAAGGACTAGCCAAGATCATACAGAAGGCACATAA
AGAAGGCGTGTGCGGTCTGCGATCTGTTTCCAGGCTGGAACACCAAATGTGGGAGGCTGTGA
AGGATGAACTCAATACACTTTTGAAGGAAAACGGTGTGGATCTCAGTGTCGTGGTTGAGAAG
CAAGAGGGAATGTACAAGTCCGCTCCCAAGCGCCTGACGGCCACGACAGAAAAATTGGAAA
TTGGCTGGAAGGCCTGGGGTAAGAGCATTTTATTTGCACCGGAACTAGCCAACAACACCTTTG
TTGTGGATGGTCCTGAGACCAAAGAATGTCCGACTCAGAATCGTGCTTGGAACAGTTTGGAA
GTAGAGGATTTTGGATTCGGACTCACCAGTACTCGGATGTTCCTCAAGGTCAGAGAGAGCAA
CACAACAGAATGTGACTCTAAGATCATTGGAACTGCTGTGAAGAACAACTTGGCCATCCACA
GTGACCTGTCATATTGGATTGAGAGCAGGCTAAATGACACCTGGAAGCTGGAGAGGGCAGTA
CTTGGTGAGGTTAAATCCTGTACGTGGCCTGAGACCCATACATTGTGGGGCGATGGAATCCTC
GAGAGCGACTTGATAATTCCAGTCACACTGGCTGGACCTCGAAGCAACCACAATCGGAGACC
GGGGTACAAGACACAAAACCAGGGTCCCTGGGATGAAGGCCGAGTAGAAATTGACTTTGATT
ATTGCCCAGGAACCACAGTCACGCTGAGCGAAAGCTGTGGACACCGAGGCCCGGCCACTCG
CACTACGACAGAGAGTGGGAAGTTGATCACAGACTGGTGCTGCAGAAGCTGCACGTTACCAC
CACTTCGCTACCAAACTGACAGCGGCTGCTGGTATGGAATGGAGATCAGACCACAGAGACAT

File S2 ISA fragments

GATGAGAAGACCCTCGTGCAGTCACAAGTGAATGCTTATAATGCTGATATGATTGACCCTTTTC
AGTTGGGCCTTCTGGTCGTGTTCTTGGCCACCCAGGAGGTCCTTCGC***ctactctggcgtcgatgaggga***

>wt-NY99_**Fragment-II**
TATAATGCTGATATGATTGACCCTTTTCAGTTGGGCCTTCTGGTCGTGTTCTTGGCCACCCAGG
AGGTCCTTCGCAAGAGGTGGACAGCCAAGATCAGCATGCCAGCTATACTGATTGCTCTGCTAG
TCCTGGTGTTTGGGGGCATTACTTACACTGATGTGTTACGCTATGTCATCTTGGTGGGGGCAGC
TTTCGCAGAATCTAATTCGGGAGGAGACGTGGTACACTTGGCGCTCATGGCGACCTTCAAGAT
ACAACCAGTGTTTATGGTGGCATCGTTTCTCAAAGCGAGATGGACCAACCAGGAGAACATTTT
GTTGATGTTGGCGGCTGTTTTCTTTCAAATGGCTTATCACGATGCCCGCCAAATTCTGCTCTGG
GAGATCCCTGATGTGTTGAATTCACTGGCGGTAGCTTGGATGATACTGAGAGCCATAACATTCA
CAACGACATCAAACGTGGTTGTTCCGCTGCTAGCCCTGCTAACACCCGGGCTGAGATGCTTGA
ATCTGGATGTGTACAGGATACTGCTGTTGATGGTCGGAATAGGCAGCTTGATCAGGGAGAAGA
GGAGTGCAGCCGCAAAAAAGAAAGGAGCAAGTCTGCTATGCTTGGCTCTAGCCTCAACAGG
ACTTTTCAACCCCATGATCCTTGCTGCTGGACTGATTGCATGTGATCCCAACCGTAAACGCGG
ATGGCCCGCAACTGAAGTGATGACAGCTGTCGGCCTAATGTTTGCCATCGTCGGAGGGCTGGC
AGAGCTTGACATTGACTCCATGGCCATTCCAATGACTATCGCGGGGCTCATGTTTGCTGCTTTC
GTGATTTCTGGGAAATCAACAGATATGTGGATTGAGAGAACGGCGGACATTTCCTGGGAAAGT
GATGCAGAAATTACAGGCTCGAGCGAAAGAGTTGATGTGCGGCTTGATGATGATGGAAACTT
CCAGCTCATGAATGATCCAGGAGCACCTTGGAAGATATGGATGCTCAGAATGGTCTGTCTCGC
GATTAGTGCGTACACCCCCTGGGCAATCTTGCCCTCAGTAGTTGGATTTTGGATAACTCTCCAA
TACACAAAGAGAGGAGGCGTGTTGTGGGACACTCCCTCACCAAAGGAGTACAAAAAGGGGG
ACACGACCACCGGCGTCTACAGGATCATGACTCGTGGGCTGCTCGGCAGTTATCAAGCAGGA
GCGGGCGTGATGGTTGAAGGTGTTTTCCACACCCTTTGGCATACAACAAAAGGAGCCGCTTT
GATGAGCGGAGAGGGCCGCCTGGACCCATACTGGGGCAGTGTCAAGGAGGATCGACTTTGTT
ACGGAGGACCCTGGAAATTGCAGCACAAGTGGAACGGGCAGGATGAGGTGCAGATGATTGT
GGTGGAACCTGGCAAGAACGTTAAGAACGTCCAGACGAAACCAGGGGTGTTCAAAACACCT
GAAGGAGAAATCGGGGCCGTGACTTTGGACTTCCCCACTGGAACATCAGGCTCACCAATAGT
GGACAAAAACGGTGATGTGATTGGGCTTTATGGCAATGGAGTCATAATGCCCAACGGCTCATA
CATAAGCGCGATAGTGCAGGGTGAAAGGATGGATGAGCCAATCCCAGCCGGATTCGAACCTG
AGATGCTGAGGAAAAAACAGATCACTGTACTGGATCTCCATCCCGGCGCCGGTAAAACAAGG
AGGATTCTGCCACAGATCATCAAAGAGGCCATAAACAGAAGACTGAGAACAGCCGTGCTAGC
GCCAACCAGGGTTGTGGCTGCTGAGATGGCTGAAGCACTGAGAGGACTGCCCATCCGGTACC
AGACATCCGCAGTGCCCAGAGAACATAATGGAAATGAGATTGTTGATGTCATGTGTCATGCTA
CCCTCACCCACAGGCTGATGTCTCCTCACAGGGTGCCGAACTACAACCTGTTCGTGATGGATG
AGGCTCATTTCACCGACCCAGCTAGCATTGCAGCAAGAGGTTACATTTCCACAAAGGTCGAG
CTAGGGGAGGCGGCGGCAATATTCATGACAGCCACCCCACCAGGCACTTCAGATCCATTCCCA
GAGTCCAATTCACCAATTTCCGACTTACAGACTGAGATCCCGGATCGAGCTTGGAACTCTGGA
TACGAATGGATCACAGAATACACCGGGAAGACGGTTTGGTTTGTGCCTAGTGTCAAGATGGG
GAATGAGATTGCCCTTTGCCTACAACGTGCTGGAAAGAAAGTAGTCCAATTGAACAGAAAGT
CGTACGAGACGGAGTACCCAAAATGTAAGAACGATGATTGGGACTTTGTTATCACAACAGAC
ATATCTGAAATGGGGGCTAACTTCAAGGCGAGCAGGGTGATTGACAGCCGGAAGAGTGTGAA
ACCAACCATCATAACAGAAGGAGAAGGGAGAGTGATCCTGGGAGAACCATCTGCAGTGACA
GCAGCTAGTGCCGCCCAGAGACGTGGACGTATCGGTAGAAATCCGTCGCAAGTTGGTGATGA
GTACTGTTATGGGGGGCACACGAATGAAGACGACTCGAACTTCGCCCATTGGACTGAGGCAC
GAATCATGCTGGACAACATCAACATGCCAAACGGACTGATCGCTCAATTCTACCAACCAGAGC
GTGAGAAGGTATATACCATGGATGGGGAATACCGGCTCAGAGGAGAAGAGAGAAAAAACTTT

File S2 ISA fragments

CTGGAACTGTTGAGGACTGCAGATCTGCCAGTTTGGCTGGCTTACAAGGTTGCAGCGGCTGG
AGTGTCATACCACGACCGGAGGTGGTGCTTTGATGGTCCTAGGACAAACACAATTTTAGAAG
ACAACAACGAAGTGGAAGTCATCACGAAGCTTGGTGAAAGGAAGATTCTGAGGCCGCGCTG
GATTGATGCCAGGGTGTACTCGGATCACCAGGCACTAAAGGCGTTCAAGGACTTCGCCTCGG
GAAAACGTTCTCAGATAGGGCTCATTGAGGTTCTGGGAAAGATGCCTGAGCACTTCATGGGG
AAGACATGGGAAGCACTTGACACCATGTACGTTGTGGCCACTGCAGAGAAAGGAGGAAGAG
CTCACAGAATGGCCCTGGAGGAACTGCCAGATGCTCTTCAGACAATTGCCTTGATTGCCTTAT
TGAGTGTGATGACCATGGGAGTATTCTTCCTCCTCATGCAGCGGAAGGGCATTGGAAAGATAG
GTTTGGGAGGCGCTGTCTTGGGAGTCGCGACCTTTTTCTGTTGGATGGCTGAAGTTCCAGGA
ACGAAGATCGCCGGAATGTTGCTGCTCTCCCTTCTCTTGATGATTGTGCTAATTCCTGAGCCAG
AGAAGCAACGTTCGCAGACAGACAACCAGCTAGCCGTGTTCCTGATTTGTGTCATGACCCTT
GTGAGCGCAGTGGCAGCCAACGAGATGGGTTGGCTAGATAAGACCAAGAGTGACATAAGCA
GTTTGTTTGGGCAAAGAATTGAGGTCAAGGAGAATTTCAGCATGGGAGAGTTTCTTCTGGAC
TTGAGGCCGGCAACAGCCTGGTCACTGTACGCTGTGACAACAGCGGTCCTCACTCCACTGCT
AAAGCATTTGATCACGTCAGATTACATCAACACCTCATTGACCTCAATAAACGTTCAGGCAAG
TGCACTATTCACACTCGCGCGAGGCTTCCCCTTCGTCGATGTTGGAGTGTCGGCTCTCCTGCT
AGCAGCCGGATGCTGGGGACAAGTCACCCTCACCGTTACGGTAACAGCGGCAACACTCCTTT
TTTGCCACTATGCCTACATGGTTCCCGGTTGGCAAGCTGAGGCAATGCGCTCAGCCCAGCGGC
GGACAGCGGCCGGAATCATGAAGAACGCTGTAGTGGATGGCATCGTGGCCACGGACGTCCCA
GAATTAGAGCGCACCACACCCATCATGCAGAAGAAAGTTGGACAGATCATGCTGATCTTGGTG
TCTCTAGCTGCAGTAGTAGTGAACCCGTCTGTGAAGACAGTACGAGAAGCCGGAATTTTGATC
ACGGCCGCAGCGGTGACGCTTTGGGAGAATGGAGCAAGCTCTGTTTGGAACGCAACAACTG
CCATCGGACTCTGCCACATCATGCGTGGGGGTTGGTTGTCATGTCTATCCATAACATGGACACT
CATAAAGAACATGGAAAAACCAGGACTAAAAAGAGGTGGGGCAAAAGGACGCACCTTGGGA
GAGGTTTGGAAAGAAAGACTCAACCAGATGACAAAAGAAGAGTTCACT

>NY99-Permuted-NS5_**Fragment-III**
GGTGGGGCAAAAGGACGCACCTTGGGAGAGGTTTGGAAAGAAAGACTCAACCAGATGACAA
AAGAAGAGTTCACTAGGTACCGAAAGGAGGCAATCATCGAAGTCGATCGATCTGCAGCCAAA
CATGCCAGGAAAGAGGGAAACGTCACTGGAGGGCATCCAGTCTCCAGGGGCACTGCAAAGC
TCAGATGGCTTGTCGAACGGAGGTTTCTCGAACCGGTGGGAAAAGTGATTGACCTAGGGTGT
GGAAGAGGTGGCTGGTGTTATTACATGGCGACCCAAAAAAGAGTCCAAGAAGTCAGAGGCTA
CACTAAGGGCGGGCCCGGGCATGAAGAGCCCCAACTCGTCCAAAGTTATGGTTGGAACATTG
TCACAATGAAGAGTGGAGTTGATGTTTTTTATAGACCATCAGAATGCTGTGACACCCTACTCTG
TGACATAGGAGAGTCTTCGTCCAGTGCTGAGGTGGAAGAGCACAGAACCATCCGGGTCCTGG
AAATGGTCGAGGATTGGCTCCACCGGGGACCCAGGGAGTTCTGCGTGAAGGTGCTGTGTCCC
TACATGCCGAAAGTAATTGAGAAGATGGAACTGCTGCAACGGCGATATGGTGGTGGACTGGT
CAGAAATCCCCTCTCCCGAAATTCGACTCACGAGATGTACTGGGTGAGTCGGGCCTCGGGCA
ATGTGGTGCATTCAGTGAACATGACCAGCCAGGTCCTGCTAGGAAGAATGGAAAAGAGAACT
TGGAAGGGACCACAATACGAGGAAGATGTAAACTTGGGAAGTGGCACCAGGGCAGTGGGCA
AGCCCCTCCTCAACTCAGATACTAGTAAAATCAAGAACAGGATTGAACGTCTCAGGCGGGAG
TACAGTTCCACTTGGCATCACGATGAGAACCACCCATATAGAACGTGGAACTACCACGGCAGC
TATGATGTCAAGCCCACAGGCTCAGCCAGCTCGCTGGTCAATGGGGTGGTGAGGCTCCTGTC
AAAACCCTGGGACACGATCACGAATGTCACAACCATGGCCATGACAGACACTACGCCATTTG
GACAGCAACGGGTCTTCAAAGAAAAGGTGGACACCAAAGCACCTGAGCCTCCAGAAGGAGT
CAAATACGTGCTGAACGAAACCACAAACTGGTTGTGGGCATTTTTGGCCAGAGAAAAACGTC
CCAGAATGTGCAGCCGAGAGGAATTCATCAGGAAAGTAAACTCCAATGCTGCCTTGGGTGCC

File S2 ISA fragments

ATGTTTGAAGAACAGAATCAATGGAGGAGCGCCAGAGAAGCAGTCGAGGATCCAAAGTTCT
GGGAGATGGTGGACGAGGAGCGCGAAGCCCACCTCCGGGGAGAATGTCACACGTGCATCTA
CAACATGATGGGCAAAAGAGAGAAAAAACCAGGAGAGTTCGGGAAAGCAAAGGGCAGCAG
AGCCATTTGGTTCATGTGGCTTGGAGCTCGCTTTCTGGAGTTCGAGGCACTGGGCTTTCTTAAT
GAAGATCACTGGCTTGGGAGGAAGAACTCTGGAGGAGGGGTGGAGGGATTGGGCCTGCAAA
AACTGGGGTACATCCTGCGTGAGGTTGGCACTCGGCCTGGGGGAAAGATCTATGCTGATGAC
ACTGCCGGTTGGGACACCCGCATCACCAGAGCAGACTTGGAAAACGAAGCCAAAGTGCTCG
AGCTGCTGGATGGGGAACATCGACGTCTTGCCAGAGCTATCATTGAGCTCACCTACCGTCATA
AAGTCGTGAAAGTGATGCGCCCTGCGGCCGACGGCAGAACAGTCATGGATGTTATTTCGAGA
GAAGACCAGAGAGGAAGTGGCCAGGTAGTCACTTACGCCCTCAACACCTTCACAAATCTGGC
CGTCCAGCTGGTGAGGATGATGGAAGGCGAAGGAGTCATTGGCCCAGATGATGTTGAGAAAC
TCACCAAAGGAAAAGGACCAAAAGTGAGGACGTGGCTGTTTGAGAATGGGGAAGAAAGACT
GAGCCGAATGGCAGTCAGTGGAGATGACTGTGTGGTAAAACCGCTTGACGACCGTTTTGCTA
CGTCTCTCCATTTCCTCAATGCCATGTCAAAGGTCCGCAAGGACATCCAAGAGTGGAAACCTT
CCACCGGTTGGTATGACTGGCAGCAAGTTCCCTTTTGTTCAAACCATTTCACAGAATTGATCAT
GAAAGATGGAAGAACCCTAGTTGTCCCTTGCCGAGGGCAGGATGAATTGGTGGGAAGAGCGC
GCATATCTCCTGGAGCCGGATGGAATGTTCGCGACACTGCCTGTCTGGCCAAGTCCTATGCAC
AGATGTGGCTCCTGCTTTATTTTCACAGAAGAGATCTGCGACTCATGGCCAATGCAATTTGTTC
CGCGGTCCCTGTCAACTGGGTGCCTACCGGGAGAACCACGTGGTCCATTCATGCTGGAGGAG
AGTGGATGACAACAGAAGACATGTTGGAGGTGTGGAACCGAGTCTGGATTGAGGAGAATGA
ATGGATGGAAGACAAGACTCCTGTGGAGAAATGGAGTGATGTTCCATACTCTGGAAAGCGAG
AGGACATTTGGTGTGGCAGCCTGATTGGCACCAGAGCCCGGGCCACTTGGGCAGAGAACATC
CAGGTGGCTATCAACCAAGTGAGAGCAATCATCGGAGATGAGAAGTATGTGGACTACATGAG
TTCACTAAAGAGATATGAAGACACAACTTTGGTTGAGGACACAGTACTGTAGATATTTAATCA
ATTGTAAATAGACAATATAAGTATGCATAAAAGTGTAGTTTTATAGTAGTATTTAGTGGTGTTAG
TGTAAATAGTTAAGAAAATTTTGAGGAGAAAGTCAGGCCGGGAAGTTCCCGCCACCGGAAGT
TGAGTAGACGGTGCTGCCTGCGACTCAACCCCAGGAGGACTGGGTGAACAAAGCCGCGAAG
TGATCCATGTAAGCCCTCAGAACCGTCTCGGAAGGAGGACCCCACATGTTGTAACTTCAAAG
CCCAATGTCAGACCACGCTACGGCGTGCTACTCTGCGGAGAGTGCAGTCTGCGATAGTGCCCC
AGGAGGACTGGGTTAACAAAGGCAAACCAACGCCCCACGCGGCCCTAGCCCCGGTAATGGT
GTTAACCAGGGCGAAAGGACTAGAGGTTAGAGGAGACCCCGCGGTTTAAAGTGCACGGCCC
AGCCTGGCTGAAGCTGTAGGTCAGGGGAAGGACTAGAGGTTAGTGGAGACCCCGTGCCACA
AAACACCACAACAAAACAGCATATTGACACCTGGGATAGACTAGGAGATCTTCTGCTCTGCAC
AACCAGCCACACGGCACAGTGCGCCGACAATGGTGGCTGGTGGTGCGAGAACACAGGATCT
GGCCGGCATGGTCCCAGCCTCCTCGCTGGCGCCGGCTGGGCAACATTCCGAGGGGACCGTCC
CCTCGGTAATGGCGAATGGGACTCGCGACAGACATGATAAGATACATTGATGAGTTTGGACAA
ACCACAACTAGAATGCAGTGAAAAAAATGCTTTATTTGTGAAATTAAGCGCTGGCATTGACCC
TGAG

File S2 ISA fragments

E+CG

Highlighted in red – pCMV promoter sequence.
Highlighted in green – HDR/SV40pA sequence.

>NY99-CpG-E_**Fragment-I**
CACCCAACTGATCTTCAGCATCTTCAATATTGGCCATTAGCCATATTATTCATTGGTTATATAGCA
TAAATCAATATTGGCTATTGGCCATTGCATACGTTGTATCTATATCATAATATGTACATTTATATTG
GCTCATGTCCAATATGACCGCCATGTTGGCATTGATTATTGACTAGTTATTAATAGTAATCAATTA
CGGGGTCATTAGTTCATAGCCCATATATGGAGTTCCGCGTTACATAACTTACGGTAAATGGCCC
GCCTGGCTGACCGCCCAACGACCCCCGCCCATTGACGTCAATAATGACGTATGTTCCCATAGT
AACGCCAATAGGGACTTTCCATTGACGTCAATGGGTGGAGTATTTACGGTAAACTGCCCACTT
GGCAGTACATCAAGTGTATCATATGCCAAGTCCGCCCCCTATTGACGTCAATGACGGTAAATGG
CCCGCCTGGCATTATGCCCAGTACATGACCTTACGGGACTTTCCTACTTGGCAGTACATCTACG
TATTAGTCATCGCTATTACCATGGTGATGCGGTTTTGGCAGTACACCAATGGGCGTGGATAGCG
GTTTGACTCACGGGGATTTCCAAGTCTCCACCCCATTGACGTCAATGGGAGTTTGTTTTGGCA
CCAAAATCAACGGGACTTTCCAAAATGTCGTAATAACCCCGCCCCGTTGACGCAAATGGGCG
GTAGGCGTGTACGGTGGGAGGTCTATATAAGCAGAGCTCGTTTAGTGAACCGAGTAGTTCGCC
TGTGTGAGCTGACAAACTTAGTAGTGTTTGTGAGGATTAACAACAATTAACACAGTGCGAGCT
GTTTCTTAGCACGAAGATCTCGATGTCTAAGAAACCAGGAGGGCCCGGCAAGAGCCGGGCTG
TCAATATGCTAAAACGCGGAATGCCCCGCGTGTTGTCCTTGATTGGACTGAAGAGGGCTATGT
TGAGCCTGATCGACGGCAAGGGGCCAATACGATTTGTGTTGGCTCTCTTGGCGTTCTTCAGGT
TCACAGCAATTGCTCCGACCCGAGCAGTGCTGGATCGATGGAGAGGTGTGAACAAACAAACA
GCGATGAAACACCTTCTGAGTTTTAAGAAGGAACTAGGGACCTTGACCAGTGCTATCAATCGG
CGGAGCTCAAAACAAAAGAAAAGAGGAGGAAAGACCGGAATTGCAGTCATGATTGGCCTGA
TCGCCAGCGTAGGAGCAGTTACCCTCTCTAACTTCCAAGGGAAGGTGATGATGACGGTAAATG
CTACTGACGTCACAGATGTCATCACGATTCCAACAGCTGCTGGAAAGAACCTATGCATTGTCA
GAGCAATGGATGTGGGATACATGTGCGATGATACTATCACTTATGAATGCCCAGTACTGTCGGC
TGGTAATGATCCAGAAGACATCGACTGTTGGTGCACAAAGTCAGCAGTCTACGTCAGGTATGG
AAGATGCACCAAGACACGCCACTCAAGACGCAGTCGGAGGTCACTGACAGTGCAGACACAC
GGAGAAAGCACTCTAGCGAACAAGAAGGGGGCTTGGATGGACAGCACCAAGGCCACAAGGT
ATTTGGTAAAAACAGAATCATGGATCTTGAGGAACCCTGGATATGCCCTGGTGGCAGCCGTCA
TTGGTTGGATGCTTGGGAGCAACACCATGCAGAGAGTTGTGTTTGTCGTGCTATTGCTTTTGG
TGGCCCCAGCTTACAGCTTCAACTGCCTTGGAATGAGCAACAGAGACTTTTTGGAAGGCGTG
TCCGGAGCAACGTGGGTCGATTTGGTTCTCGAAGGCGACAGTTGTGTAACGATAATGTCGAA
AGACAAACCAACGATCGACGTCAAGATGATGAACATGGAAGCGGCGAACCTCGCCGAAGTA
CGAAGTTATTGTTATTTGGCGACCGTAAGTGATCTCTCAACCAAAGCGGCGTGTCCGACAATG
GGCGAAGCGCACAACGACAAACGTGCGGATCCCGCATTTGTGTGCCGACAAGGCGTCGTCG
ACCGCGGATGGGGAAACGGATGTGGACTGTTCGGAAAAGGAAGCATCGACACGTGCGCAAA
ATTTGCATGCTCGACGAAAGCGATCGGACGAACGATTTTGAAAGAAAACATTAAATACGAAG
TTGCGATTTTTGTTCACGGACCGACAACAGTAGAATCTCACGGAAATTATTCGACGCAAGTCG
GCGCGACACAAGCCGGTCGACTGAGCATCACGCCCGCGGCGCCGTCGTATACGCTAAAACTC
GGCGAATACGGCGAAGTGACGGTTGATTGCGAACCTCGTTCCGGCATCGACACGAACGCGTA
CTACGTTATGACGGTCGGAACGAAGACTTTTTTAGTTCATCGCGAATGGTTCATGGATCTTAAT
CTACCTTGGAGCAGCGCGGGAAGCACGGTTTGGCGTAATCGCGAAACGTTGATGGAATTTGA
AGAACCTCACGCGACAAAACAGTCCGTTATCGCGTTGGGTTCACAAGAAGGCGCGCTACATC
AAGCGTTGGCCGGCGCGATTCCTGTCGAATTTTCGAGCAATACAGTTAAGTTGACGTCCGGTC

File S2 ISA fragments

ATTTGAAATGTCGCGTGAAAATGGAAAAATTGCAATTGAAAGGAACGACATACGGTGTGTGTT
CGAAAGCGTTCAAATTTCTCGGAACGCCCGCGGATACCGGTCACGGAACTGTTGTTTTAGAAT
TGCAATACACTGGAACGGACGGACCTTGTAAAGTTCCGATCTCATCGGTCGCGTCATTGAACG
ATCTGACACCGGTCGGACGATTAGTGACTGTGAACCCGTTTGTTTCGGTCGCGACGGCAAATG
CAAAAGTTCTGATCGAATTGGAACCGCCGTTTGGCGACTCGTACATCGTTGTTGGACGCGGCG
AACAACAAATTAATCATCATTGGCACAAATCGGGAAGCAGCATCGGAAAAGCGTTCACGACG
ACGCTCAAAGGCGCTCAGCGACTTGCCGCGCTCGGTGATACGGCGTGGGATTTTGGATCCGTC
GGCGGGGTTTTCACGTCGGTAGGGAAGGCTGTTCATCAAGTATTCGGCGGCGCGTTTCGTTCG
CTTTTTGGCGGAATGTCGTGGATTACTCAAGGATTACTCGGCGCGCTTCTGTTGTGGATGGGA
ATTAACGCACGAGATCGATCGATCGCGCTCACATTTCTCGCTGTCGGAGGAGTTCTGCTCTTCC
TCTCCGTGAACGTGCACGCTGACACTGGGTGTGCCATAGACATCAGCCGGCAAGAGCTGAGA
TGTGGAAGTGGAGTGTTCATACACAATGATGTGGAGGCTTGGATGGACCGGTACAAGTATTAC
CCTGAAACGCCACAAGGCCTAGCCAAGATCATTCAGAAAGCTCATAAGGAAGGAGTGTGCGG
TCTACGATCAGTTTCCAGACTGGAGCATCAAATGTGGGAAGCAGTGAAGGACGAGCTGAACA
CTCTTTTGAAGGAGAATGGTGTGGACCTTAGTGTCGTGGTTGAGAAACAGGAGGGAATGTAC
AAGTCAGCACCTAAACGCCTCACCGCCACCACGGAAAAATTGGAAATTGGCTGGAAGGCCTG
GGGAAAGAGTATTTTATTTGCACCAGAACTCGCCAACAACACCTTTGTGGTTGATGGTCCGGA
GACCAAGGAATGTCCGACTCAGAATCGCGCTTGGAATAGCTTAGAAGTGGAGGATTTTGGATT
TGGTCTCACCAGCACTCGGATGTTCCTGAAGGTCAGAGAGAGCAACACAACTGAATGTGACT
CGAAGATCATTGGAACGGCTGTCAAGAACAACTTGGCGATCCACAGTGACCTGTCCTATTGG
ATTGAAAGCAGGCTCAATGATACGTGGAAGCTTGAAAGGGCAGTTCTGGGTGAAGTCAAATC
ATGTACGTGGCCTGAGACGCATACCTTGTGGGGCGATGGAATCCTTGAGAGTGACTTGATAAT
ACCAGTCACACTGGCGGGACCACGAAGCAATCACAATCGGAGACCTGGGTACAAGACACAA
AACCAGGGCCCATGGGACGAAGGCCGGGTAGAGATTGACTTCGATTACTGCCCAGGAACTAC
GGTCACCCTGAGTGAGAGCTGCGGACACCGTGGACCTGCCACTCGCACCACCACAGAGAGC
GGAAAGTTGATAACAGATTGGTGCTGCAGGAGCTGCACCTTACCACCACTGCGCTACCAAAC
TGACAGCGGCTGTTGGTATGGTATGGAGATCAGACCACAGAGACATGATGAAAAGACCCTCG
TGCAGTCACAAGTGAATGCTTATAATGCTGATATGATTGACCCTTTTCAGTTGGGCCTTCTGGT
CGTGTTCTTGGCCACCCAGGAGGTCCTTCGC

>wt-NY99_**Fragment-II**
TATAATGCTGATATGATTGACCCTTTTCAGTTGGGCCTTCTGGTCGTGTTCTTGGCCACCCAGG
AGGTCCTTCGCAAGAGGTGGACAGCCAAGATCAGCATGCCAGCTATACTGATTGCTCTGCTAG
TCCTGGTGTTTGGGGGCATTACTTACACTGATGTGTTACGCTATGTCATCTTGGTGGGGGCAGC
TTTCGCAGAATCTAATTCGGGAGGAGACGTGGTACACTTGGCGCTCATGGCGACCTTCAAGAT
ACAACCAGTGTTTATGGTGGCATCGTTTCTCAAAGCGAGATGGACCAACCAGGAGAACATTTT
GTTGATGTTGGCGGCTGTTTTCTTTCAAATGGCTTATCACGATGCCCGCCAAATTCTGCTCTGG
GAGATCCCTGATGTGTTGAATTCACTGGCGGTAGCTTGGATGATACTGAGAGCCATAACATTCA
CAACGACATCAAACGTGGTTGTTCCGCTGCTAGCCCTGCTAACACCCGGGCTGAGATGCTTGA
ATCTGGATGTGTACAGGATACTGCTGTTGATGGTCGGAATAGGCAGCTTGATCAGGGAGAAGA
GGAGTGCAGCCGCAAAAAAGAAAGGAGCAAGTCTGCTATGCTTGGCTCTAGCCTCAACAGG
ACTTTTCAACCCCATGATCCTTGCTGCTGGACTGATTGCATGTGATCCCAACCGTAAACGCGG
ATGGCCCGCAACTGAAGTGATGACAGCTGTCGGCCTAATGTTTGCCATCGTCGGAGGGCTGGC
AGAGCTTGACATTGACTCCATGGCCATTCCAATGACTATCGCGGGGCTCATGTTTGCTGCTTTC
GTGATTTCTGGGAAATCAACAGATATGTGGATTGAGAGAACGGCGGACATTTCCTGGGAAAGT
GATGCAGAAATTACAGGCTCGAGCGAAAGAGTTGATGTGCGGCTTGATGATGATGGAAACTT
CCAGCTCATGAATGATCCAGGAGCACCTTGGAAGATATGGATGCTCAGAATGGTCTGTCTCGC

File S2 ISA fragments

GATTAGTGCGTACACCCCCTGGGCAATCTTGCCCTCAGTAGTTGGATTTTGGATAACTCTCCAA
TACACAAAGAGAGGAGGCGTGTTGTGGGACACTCCCTCACCAAAGGAGTACAAAAAGGGGG
ACACGACCACCGGCGTCTACAGGATCATGACTCGTGGGCTGCTCGGCAGTTATCAAGCAGGA
GCGGGCGTGATGGTTGAAGGTGTTTTCCACACCCTTTGGCATACAACAAAAGGAGCCGCTTT
GATGAGCGGAGAGGGCCGCCTGGACCCATACTGGGGCAGTGTCAAGGAGGATCGACTTTGTT
ACGGAGGACCCTGGAAATTGCAGCACAAGTGGAACGGGCAGGATGAGGTGCAGATGATTGT
GGTGGAACCTGGCAAGAACGTTAAGAACGTCCAGACGAAACCAGGGGTGTTCAAAACACCT
GAAGGAGAAATCGGGGCCGTGACTTTGGACTTCCCCACTGGAACATCAGGCTCACCAATAGT
GGACAAAAACGGTGATGTGATTGGGCTTTATGGCAATGGAGTCATAATGCCCAACGGCTCATA
CATAAGCGCGATAGTGCAGGGTGAAAGGATGGATGAGCCAATCCCAGCCGGATTCGAACCTG
AGATGCTGAGGAAAAAACAGATCACTGTACTGGATCTCCATCCCGGCGCCGGTAAAACAAGG
AGGATTCTGCCACAGATCATCAAAGAGGCCATAAACAGAAGACTGAGAACAGCCGTGCTAGC
GCCAACCAGGGTTGTGGCTGCTGAGATGGCTGAAGCACTGAGAGGACTGCCCATCCGGTACC
AGACATCCGCAGTGCCCAGAGAACATAATGGAAATGAGATTGTTGATGTCATGTGTCATGCTA
CCCTCACCCACAGGCTGATGTCTCCTCACAGGGTGCCGAACTACAACCTGTTCGTGATGGATG
AGGCTCATTTCACCGACCCAGCTAGCATTGCAGCAAGAGGTTACATTTCCACAAAGGTCGAG
CTAGGGGAGGCGGCGGCAATATTCATGACAGCCACCCCACCAGGCACTTCAGATCCATTCCCA
GAGTCCAATTCACCAATTTCCGACTTACAGACTGAGATCCCGGATCGAGCTTGGAACTCTGGA
TACGAATGGATCACAGAATACACCGGGAAGACGGTTTGGTTTGTGCCTAGTGTCAAGATGGG
GAATGAGATTGCCCTTTGCCTACAACGTGCTGGAAAGAAAGTAGTCCAATTGAACAGAAAGT
CGTACGAGACGGAGTACCCAAAATGTAAGAACGATGATTGGGACTTTGTTATCACAACAGAC
ATATCTGAAATGGGGGCTAACTTCAAGGCGAGCAGGGTGATTGACAGCCGGAAGAGTGTGAA
ACCAACCATCATAACAGAAGGAGAAGGGAGAGTGATCCTGGGAGAACCATCTGCAGTGACA
GCAGCTAGTGCCGCCCAGAGACGTGGACGTATCGGTAGAAATCCGTCGCAAGTTGGTGATGA
GTACTGTTATGGGGGGCACACGAATGAAGACGACTCGAACTTCGCCCATTGGACTGAGGCAC
GAATCATGCTGGACAACATCAACATGCCAAACGGACTGATCGCTCAATTCTACCAACCAGAGC
GTGAGAAGGTATATACCATGGATGGGGAATACCGGCTCAGAGGAGAAGAGAGAAAAAACTTT
CTGGAACTGTTGAGGACTGCAGATCTGCCAGTTTGGCTGGCTTACAAGGTTGCAGCGGCTGG
AGTGTCATACCACGACCGGAGGTGGTGCTTTGATGGTCCTAGGACAAACACAATTTTAGAAG
ACAACAACGAAGTGGAAGTCATCACGAAGCTTGGTGAAAGGAAGATTCTGAGGCCGCGCTG
GATTGATGCCAGGGTGTACTCGGATCACCAGGCACTAAAGGCGTTCAAGGACTTCGCCTCGG
GAAAACGTTCTCAGATAGGGCTCATTGAGGTTCTGGGAAAGATGCCTGAGCACTTCATGGGG
AAGACATGGGAAGCACTTGACACCATGTACGTTGTGGCCACTGCAGAGAAAGGAGGAAGAG
CTCACAGAATGGCCCTGGAGGAACTGCCAGATGCTCTTCAGACAATTGCCTTGATTGCCTTAT
TGAGTGTGATGACCATGGGAGTATTCTTCCTCCTCATGCAGCGGAAGGGCATTGGAAAGATAG
GTTTGGGAGGCGCTGTCTTGGGAGTCGCGACCTTTTTCTGTTGGATGGCTGAAGTTCCAGGA
ACGAAGATCGCCGGAATGTTGCTGCTCTCCCTTCTCTTGATGATTGTGCTAATTCCTGAGCCAG
AGAAGCAACGTTCGCAGACAGACAACCAGCTAGCCGTGTTCCTGATTTGTGTCATGACCCTT
GTGAGCGCAGTGGCAGCCAACGAGATGGGTTGGCTAGATAAGACCAAGAGTGACATAAGCA
GTTTGTTTGGGCAAAGAATTGAGGTCAAGGAGAATTTCAGCATGGGAGAGTTTCTTCTGGAC
TTGAGGCCGGCAACAGCCTGGTCACTGTACGCTGTGACAACAGCGGTCCTCACTCCACTGCT
AAAGCATTTGATCACGTCAGATTACATCAACACCTCATTGACCTCAATAAACGTTCAGGCAAG
TGCACTATTCACACTCGCGCGAGGCTTCCCCTTCGTCGATGTTGGAGTGTCGGCTCTCCTGCT
AGCAGCCGGATGCTGGGGACAAGTCACCCTCACCGTTACGGTAACAGCGGCAACACTCCTTT
TTTGCCACTATGCCTACATGGTTCCCGGTTGGCAAGCTGAGGCAATGCGCTCAGCCCAGCGGC
GGACAGCGGCCGGAATCATGAAGAACGCTGTAGTGGATGGCATCGTGGCCACGGACGTCCCA
GAATTAGAGCGCACCACACCCATCATGCAGAAGAAAGTTGGACAGATCATGCTGATCTTGGTG

TCTCTAGCTGCAGTAGTAGTGAACCCGTCTGTGAAGACAGTACGAGAAGCCGGAATTTTGATC
ACGGCCGCAGCGGTGACGCTTTGGGAGAATGGAGCAAGCTCTGTTTGGAACGCAACAACTG
CCATCGGACTCTGCCACATCATGCGTGGGGGTTGGTTGTCATGTCTATCCATAACATGGACACT
CATAAAGAACATGGAAAAACCAGGACTAAAAAGAGGTGGGGCAAAAGGACGCACCTTGGGA
GAGGTTTGGAAAGAAAGACTCAACCAGATGACAAAAGAAGAGTTCACT

>wt-NY99_**Fragment-III**
GGTGGGGCAAAAGGACGCACCTTGGGAGAGGTTTGGAAAGAAAGACTCAACCAGATGACAA
AAGAAGAGTTCACTAGGTACCGCAAAGAGGCCATCATCGAAGTCGATCGCTCAGCGGCAAAA
CACGCCAGGAAAGAAGGCAATGTCACTGGAGGGCATCCAGTCTCTAGGGGCACAGCAAAAC
TGAGATGGCTGGTCGAACGGAGGTTTCTCGAACCGGTCGGAAAAGTGATTGACCTTGGATGT
GGAAGAGGCGGTTGGTGTTACTATATGGCAACCCAAAAAAGAGTCCAAGAAGTCAGAGGGTA
CACAAAGGGCGGTCCCGGACATGAAGAGCCCCAACTAGTGCAAAGTTATGGATGGAACATTG
TCACCATGAAGAGTGGAGTGGATGTGTTCTACAGACCTTCTGAGTGTTGTGACACCCTCCTTT
GTGACATCGGAGAGTCCTCGTCAAGTGCTGAGGTTGAAGAGCATAGGACGATTCGGGTCCTT
GAAATGGTTGAGGACTGGCTGCACCGAGGGCCAAGGGAATTTTGCGTGAAGGTGCTCTGTCC
CTACATGCCGAAAGTCATAGAGAAGATGGAGCTGCTCCAACGCCGGTATGGGGGGGGACTGG
TCAGAAACCCACTCTCACGGAATTCCACGCACGAGATGTATTGGGTGAGTCGAGCTTCAGGC
AATGTGGTACATTCAGTGAATATGACCAGCCAGGTGCTCCTAGGAAGAATGGAAAAAAGGAC
CTGGAAGGGACCCCAATACGAGGAAGATGTAAACTTGGGAAGTGGAACCAGGGCGGTGGGA
AAACCCCTGCTCAACTCAGACACCAGTAAAATCAAGAACAGGATTGAACGACTCAGGCGTGA
GTACAGTTCGACGTGGCACCACGATGAGAACCACCCATATAGAACCTGGAACTATCACGGCA
GTTATGATGTGAAGCCCACAGGCTCCGCCAGTTCGCTGGTCAATGGAGTGGTCAGGCTCCTCT
CAAAACCATGGGACACCATCACGAATGTTACCACCATGGCCATGACTGACACTACTCCCTTCG
GGCAGCAGCGAGTGTTCAAAGAGAAGGTGGACACGAAAGCTCCTGAACCGCCAGAAGGAG
TGAAGTACGTGCTCAACGAGACCACCAACTGGTTGTGGGCGTTTTTGGCCAGAGAAAAACGT
CCCAGAATGTGCTCTCGAGAGGAATTCATAAGAAAGGTCAACAGCAATGCAGCTTTGGGTGC
CATGTTTGAAGAGCAGAATCAATGGAGGAGCGCCAGAGAAGCAGTTGAAGATCCAAAATTTT
GGGAGATGGTGGATGAGGAGCGCGAGGCACATCTGCGGGGGGAATGTCACACTTGCATTTAC
AACATGATGGGAAAGAGAGAGAAAAAACCCGGAGAGTTCGGAAAGGCCAAGGGAAGCAGA
GCCATTTGGTTCATGTGGCTCGGAGCTCGCTTTCTGGAGTTCGAGGCTCTGGGTTTTCTCAAT
GAAGACCACTGGCTTGGAAGAAAGAACTCAGGAGGAGGTGTCGAGGGCTTGGGCCTCCAAA
AACTGGGTTACATCCTGCGTGAAGTTGGCACCCGGCCTGGGGGCAAGATCTATGCTGATGACA
CAGCTGGCTGGGACACCCGCATCACGAGAGCTGACTTGGAAAATGAAGCTAAGGTGCTTGAG
CTGCTTGATGGGGAACATCGGCGTCTTGCCAGGGCCATCATTGAGCTCACCTATCGTCACAAA
GTTGTGAAAGTGATGCGCCCGGCTGCTGATGGAAGAACCGTCATGGATGTTATCTCCAGAGAA
GATCAGAGGGGGAGTGGACAAGTTGTCACCTACGCCCTAAACACTTTCACCAACCTGGCCGT
CCAGCTGGTGAGGATGATGGAAGGGGAAGGAGTGATTGGCCCAGATGATGTGGAGAAACTC
ACAAAAGGGAAAGGACCCAAAGTCAGGACCTGGCTGTTTGAGAATGGGGAAGAAAGACTCA
GCCGCATGGCTGTCAGTGGAGATGACTGTGTGGTAAAGCCCCTGGACGATCGCTTTGCCACCT
CGCTCCACTTCCTCAATGCTATGTCAAAGGTTCGCAAAGACATCCAAGAGTGGAAACCGTCA
ACTGGATGGTATGATTGGCAGCAGGTTCCATTTTGCTCAAACCATTTCACTGAATTGATCATGA
AAGATGGAAGAACACTGGTGGTTCCATGCCGAGGACAGGATGAATTGGTAGGCAGAGCTCGC
ATATCTCCAGGGGCCGGATGGAACGTCCGCGACACTGCTTGTCTGGCTAAGTCTTATGCCCAG
ATGTGGCTGCTTCTGTACTTCCACAGAAGAGACCTGCGGCTCATGGCCAACGCCATTTGCTCC
GCTGTCCCTGTGAATTGGGTCCCTACCGGAAGAACCACGTGGTCCATCCATGCAGGAGGAGA
GTGGATGACAACAGAGGACATGTTGGAGGTCTGGAACCGTGTTTGGATAGAGGAGAATGAAT

File S2 ISA fragments

GGATGGAAGACAAAACCCCAGTGGAGAAATGGAGTGACGTCCCATATTCAGGAAAACGAGA
GGACATCTGGTGTGGCAGCCTGATTGGCACAAGAGCCCGAGCCACGTGGGCAGAAAACATCC
AGGTGGCTATCAACCAAGTCAGAGCAATCATCGGAGATGAGAAGTATGTGGACTACATGAGTT
CACTAAAGAGATATGAAGACACAACTTTGGTTGAGGACACAGTACTGTAGATATTTAATCAAT
TGTAAATAGACAATATAAGTATGCATAAAAGTGTAGTTTTATAGTAGTATTTAGTGGTGTTAGTG
TAAATAGTTAAGAAAATTTTGAGGAGAAAGTCAGGCCGGGAAGTTCCCGCCACCGGAAGTTG
AGTAGACGGTGCTGCCTGCGACTCAACCCCAGGAGGACTGGGTGAACAAAGCCGCGAAGTG
ATCCATGTAAGCCCTCAGAACCGTCTCGGAAGGAGGACCCCACATGTTGTAACTTCAAAGCC
CAATGTCAGACCACGCTACGGCGTGCTACTCTGCGGAGAGTGCAGTCTGCGATAGTGCCCCA
GGAGGACTGGGTTAACAAAGGCAAACCAACGCCCCACGCGGCCCTAGCCCCGGTAATGGTGT
TAACCAGGGCGAAAGGACTAGAGGTTAGAGGAGACCCCGCGGTTTAAAGTGCACGGCCCAG
CCTGGCTGAAGCTGTAGGTCAGGGGAAGGACTAGAGGTTAGTGGAGACCCCGTGCCACAAA
ACACCACAACAAAACAGCATATTGACACCTGGGATAGACTAGGAGATCTTCTGCTCTGCACAA
CCAGCCACACGGCACAGTGCGCCGACAATGGTGGCTGGTGGTGCGAGAACACAGGATCT**GG**
CCGGCATGGTCCCAGCCTCCTCGCTGGCGCCGGCTGGGCAACATTCCGAGGGGACCGT
CCCCTCGGTAATGGCGAATGGGACTCGCGACAGACATGATAAGATACATTGATGAGTTT
GGACAAACCACAACTAGAATGCAGTGAAAAAAATGCTTTATTTGTGAAATTAAGCGCTG
GCATTGACCCTGAG

File S2 ISA fragments

E/NS1+CG

Highlighted in red – pCMV promoter sequence.
Highlighted in green – HDR/SV40pA sequence.

>NY99-CpG-E-NS1_**Fragment-I**
CACCCAACTGATCTTCAGCATCTTCAATATTGGCCATTAGCCATATTATTCATTGGTTATATAGCA
TAAATCAATATTGGCTATTGGCCATTGCATACGTTGTATCTATATCATAATATGTACATTTATATTG
GCTCATGTCCAATATGACCGCCATGTTGGCATTGATTATTGACTAGTTATTAATAGTAATCAATTA
CGGGGTCATTAGTTCATAGCCCATATATGGAGTTCCGCGTTACATAACTTACGGTAAATGGCCC
GCCTGGCTGACCGCCCAACGACCCCCGCCCATTGACGTCAATAATGACGTATGTTCCCATAGT
AACGCCAATAGGGACTTTCCATTGACGTCAATGGGTGGAGTATTTACGGTAAACTGCCCACTT
GGCAGTACATCAAGTGTATCATATGCCAAGTCCGCCCCCTATTGACGTCAATGACGGTAAATGG
CCCGCCTGGCATTATGCCCAGTACATGACCTTACGGGACTTTCCTACTTGGCAGTACATCTACG
TATTAGTCATCGCTATTACCATGGTGATGCGGTTTTGGCAGTACACCAATGGGCGTGGATAGCG
GTTTGACTCACGGGGATTTCCAAGTCTCCACCCCATTGACGTCAATGGGAGTTTGTTTTGGCA
CCAAAATCAACGGGACTTTCCAAAATGTCGTAATAACCCCGCCCCGTTGACGCAAATGGGCG
GTAGGCGTGTACGGTGGGAGGTCTATATAAGCAGAGCTCGTTTAGTGAACCGAGTAGTTCGCC
TGTGTGAGCTGACAAACTTAGTAGTGTTTGTGAGGATTAACAACAATTAACACAGTGCGAGCT
GTTTCTTAGCACGAAGATCTCGATGTCTAAGAAACCAGGAGGGCCCGGCAAGAGCCGGGCTG
TCAATATGCTAAAACGCGGAATGCCCCGCGTGTTGTCCTTGATTGGACTGAAGAGGGCTATGT
TGAGCCTGATCGACGGCAAGGGGCCAATACGATTTGTGTTGGCTCTCTTGGCGTTCTTCAGGT
TCACAGCAATTGCTCCGACCCGAGCAGTGCTGGATCGATGGAGAGGTGTGAACAAACAAACA
GCGATGAAACACCTTCTGAGTTTTAAGAAGGAACTAGGGACCTTGACCAGTGCTATCAATCGG
CGGAGCTCAAAACAAAAGAAAAGAGGAGGAAAGACCGGAATTGCAGTCATGATTGGCCTGA
TCGCCAGCGTAGGAGCAGTTACCCTCTCTAACTTCCAAGGGAAGGTGATGATGACGGTAAATG
CTACTGACGTCACAGATGTCATCACGATTCCAACAGCTGCTGGAAAGAACCTATGCATTGTCA
GAGCAATGGATGTGGGATACATGTGCGATGATACTATCACTTATGAATGCCCAGTACTGTCGGC
TGGTAATGATCCAGAAGACATCGACTGTTGGTGCACAAAGTCAGCAGTCTACGTCAGGTATGG
AAGATGCACCAAGACACGCCACTCAAGACGCAGTCGGAGGTCACTGACAGTGCAGACACAC
GGAGAAAGCACTCTAGCGAACAAGAAGGGGGCTTGGATGGACAGCACCAAGGCCACAAGGT
ATTTGGTAAAAACAGAATCATGGATCTTGAGGAACCCTGGATATGCCCTGGTGGCAGCCGTCA
TTGGTTGGATGCTTGGGAGCAACACCATGCAGAGAGTTGTGTTTGTCGTGCTATTGCTTTTGG
TGGCCCCAGCTTACAGCTTCAACTGCCTTGGAATGAGCAACAGAGACTTTTTGGAAGGCGTG
TCCGGAGCAACGTGGGTCGATTTGGTTCTCGAAGGCGACAGTTGTGTAACGATAATGTCGAA
AGACAAACCAACGATCGACGTCAAGATGATGAACATGGAAGCGGCGAACCTCGCCGAAGTA
CGAAGTTATTGTTATTTGGCGACCGTAAGTGATCTCTCAACCAAAGCGGCGTGTCCGACAATG
GGCGAAGCGCACAACGACAAACGTGCGGATCCCGCATTTGTGTGCCGACAAGGCGTCGTCG
ACCGCGGATGGGGAAACGGATGTGGACTGTTCGGAAAAGGAAGCATCGACACGTGCGCAAA
ATTTGCATGCTCGACGAAAGCGATCGGACGAACGATTTTGAAAGAAAACATTAAATACGAAG
TTGCGATTTTTGTTCACGGACCGACAACAGTAGAATCTCACGGAAATTATTCGACGCAAGTCG
GCGCGACACAAGCCGGTCGACTGAGCATCACGCCCGCGGCGCCGTCGTATACGCTAAAACTC
GGCGAATACGGCGAAGTGACGGTTGATTGCGAACCTCGTTCCGGCATCGACACGAACGCGTA
CTACGTTATGACGGTCGGAACGAAGACTTTTTAGTTCATCGCGAATGGTTCATGGATCTTAAT
CTACCTTGGAGCAGCGCGGGAAGCACGGTTTGGCGTAATCGCGAAACGTTGATGGAATTTGA
AGAACCTCACGCGACAAAACAGTCCGTTATCGCGTTGGGTTCACAAGAAGGCGCGCTACATC
AAGCGTTGGCCGGCGCGATTCCTGTCGAATTTTCGAGCAATACAGTTAAGTTGACGTCCGGTC

File S2 ISA fragments

ATTTGAAATGTCGCGTGAAAATGGAAAAATTGCAATTGAAAGGAACGACATACGGTGTGTGTT
CGAAAGCGTTCAAATTTCTCGGAACGCCCGCGGATACCGGTCACGGAACTGTTGTTTTAGAAT
TGCAATACACTGGAACGGACGGACCTTGTAAAGTTCCGATCTCATCGGTCGCGTCATTGAACG
ATCTGACACCGGTCGGACGATTAGTGACTGTGAACCCGTTTGTTTCGGTCGCGACGGCAAATG
CAAAAGTTCTGATCGAATTGGAACCGCCGTTTGGCGACTCGTACATCGTTGTTGGACGCGGCG
AACAACAAATTAATCATCATTGGCACAAATCGGGAAGCAGCATCGGAAAAGCGTTCACGACG
ACGCTCAAAGGCGCTCAGCGACTTGCCGCGCTCGGTGATACGGCGTGGGATTTTGGATCCGTC
GGCGGGGTTTTCACGTCGGTAGGGAAGGCTGTTCATCAAGTATTCGGCGGCGCGTTTCGTTCG
CTTTTTGGCGGAATGTCGTGGATTACTCAAGGATTACTCGGCGCGCTTCTGTTGTGGATGGGA
ATTAACGCACGAGATCGATCGATCGCGCTCACATTTCTCGCTGTCGGAGGAGTTCTGCTCTTCC
TCTCCGTGAACGTGCACGCTGACACTGGGTGTGCCATAGACATCAGCCGGCAAGAGCTACGA
TGCGGAAGTGGCGTGTTTATTCACAACGACGTCGAAGCGTGGATGGATCGGTACAAGTATTAT
CCGGAAACGCCGCAAGGTCTTGCGAAAATTATACAGAAAGCGCACAAAGAAGGCGTTTGCG
GTCTACGATCGGTTTCTCGACTCGAACATCAAATGTGGGAAGCAGTGAAGGACGAGCTAAAC
ACGCTTTTAAAAGAAAACGGTGTCGATCTCAGCGTCGTCGTTGAAAAACAAGAGGGAATGTA
CAAGTCGGCGCCGAAACGACTCACCGCAACGACGGAAAAATTGGAAATTGGATGGAAGGCG
TGGGGAAAAAGCATTTTATTCGCGCCTGAACTCGCGAACAATACGTTCGTCGTCGACGGTCCT
GAAACGAAAGAATGTCCAACGCAAAATCGCGCGTGGAACAGTTTAGAAGTCGAGGATTTCGG
ATTCGGTCTCACGAGCACGCGGATGTTTCTTAAGGTCCGAGAGAGCAACACGACGGAATGCG
ACTCGAAAATAATTGGAACGGCGGTTAAAAACAATTTGGCGATACACAGCGATCTTTCTTATT
GGATCGAAAGTCGGCTGAACGATACTTGGAAACTCGAACGCGCCGTTCTCGGCGAAGTAAAA
TCTTGCACGTGGCCGGAGACGCATACGTTGTGGGGTGACGGAATTCTCGAAAGCGATTTGATA
ATTCCCGTGACCCTCGCGGGACCGCGAAGCAATCACAATCGTCGACCGGGGTATAAAACGCA
AAATCAGGGTCCATGGGACGAAGGACGCGTCGAGATTGATTTCGATTATTGTCCCGGAACAAC
GGTGACACTGAGTGAGAGTTGTGGACATCGCGGACCCGCGACGCGAACGACGACGGAGAGT
GGAAAGTTGATTACGGATTGGTGCTGTCGTAGTTGCACCTTGCCTCCGCTTCGTTACCAAACC
GACAGCGGATGTTGGTACGGGATGGAAATACGACCGCAACGACACGATGAAAAGACCCTCGT
GCAGTCACAAGTGAATGCTTATAATGCTGATATGATTGACCCTTTTCAGTTGGGCCTTCTGGTC
GTGTTCTTGGCCACCCAGGAGGTCCTTCGC

>wt-NY99_**Fragment-II**
TATAATGCTGATATGATTGACCCTTTTCAGTTGGGCCTTCTGGTCGTGTTCTTGGCCACCCAGG
AGGTCCTTCGCAAGAGGTGGACAGCCAAGATCAGCATGCCAGCTATACTGATTGCTCTGCTAG
TCCTGGTGTTTGGGGGCATTACTTACACTGATGTGTTACGCTATGTCATCTTGGTGGGGGCAGC
TTTCGCAGAATCTAATTCGGGAGGAGACGTGGTACACTTGGCGCTCATGGCGACCTTCAAGAT
ACAACCAGTGTTTATGGTGGCATCGTTTCTCAAAGCGAGATGGACCAACCAGGAGAACATTTT
GTTGATGTTGGCGGCTGTTTTCTTTCAAATGGCTTATCACGATGCCCGCCAAATTCTGCTCTGG
GAGATCCCTGATGTGTTGAATTCACTGGCGGTAGCTTGGATGATACTGAGAGCCATAACATTCA
CAACGACATCAAACGTGGTTGTTCCGCTGCTAGCCCTGCTAACACCCGGGCTGAGATGCTTGA
ATCTGGATGTGTACAGGATACTGCTGTTGATGGTCGGAATAGGCAGCTTGATCAGGGAGAAGA
GGAGTGCAGCCGCAAAAAAGAAAGGAGCAAGTCTGCTATGCTTGGCTCTAGCCTCAACAGG
ACTTTTCAACCCCATGATCCTTGCTGCTGGACTGATTGCATGTGATCCCAACCGTAAACGCGG
ATGGCCCGCAACTGAAGTGATGACAGCTGTCGGCCTAATGTTTGCCATCGTCGGAGGGCTGGC
AGAGCTTGACATTGACTCCATGGCCATTCCAATGACTATCGCGGGGCTCATGTTTGCTGCTTTC
GTGATTTCTGGGAAATCAACAGATATGTGGATTGAGAGAACGGCGGACATTTCCTGGGAAAGT
GATGCAGAAATTACAGGCTCGAGCGAAAGAGTTGATGTGCGGCTTGATGATGATGGAAACTT
CCAGCTCATGAATGATCCAGGAGCACCTTGGAAGATATGGATGCTCAGAATGGTCTGTCTCGC

File S2 ISA fragments

GATTAGTGCGTACACCCCCTGGGCAATCTTGCCCTCAGTAGTTGGATTTTGGATAACTCTCCAA
TACACAAAGAGAGGAGGCGTGTTGTGGGACACTCCCTCACCAAAGGAGTACAAAAAGGGGG
ACACGACCACCGGCGTCTACAGGATCATGACTCGTGGGCTGCTCGGCAGTTATCAAGCAGGA
GCGGGCGTGATGGTTGAAGGTGTTTTCCACACCCTTTGGCATACAACAAAAGGAGCCGCTTT
GATGAGCGGAGAGGGCCGCCTGGACCCATACTGGGGCAGTGTCAAGGAGGATCGACTTTGTT
ACGGAGGACCCTGGAAATTGCAGCACAAGTGGAACGGGCAGGATGAGGTGCAGATGATTGT
GGTGGAACCTGGCAAGAACGTTAAGAACGTCCAGACGAAACCAGGGGTGTTCAAAACACCT
GAAGGAGAAATCGGGGCCGTGACTTTGGACTTCCCCACTGGAACATCAGGCTCACCAATAGT
GGACAAAAACGGTGATGTGATTGGGCTTTATGGCAATGGAGTCATAATGCCCAACGGCTCATA
CATAAGCGCGATAGTGCAGGGTGAAAGGATGGATGAGCCAATCCCAGCCGGATTCGAACCTG
AGATGCTGAGGAAAAAACAGATCACTGTACTGGATCTCCATCCCGGCGCCGGTAAAACAAGG
AGGATTCTGCCACAGATCATCAAAGAGGCCATAAACAGAAGACTGAGAACAGCCGTGCTAGC
GCCAACCAGGGTTGTGGCTGCTGAGATGGCTGAAGCACTGAGAGGACTGCCCATCCGGTACC
AGACATCCGCAGTGCCCAGAGAACATAATGGAAATGAGATTGTTGATGTCATGTGTCATGCTA
CCCTCACCCACAGGCTGATGTCTCCTCACAGGGTGCCGAACTACAACCTGTTCGTGATGGATG
AGGCTCATTTCACCGACCCAGCTAGCATTGCAGCAAGAGGTTACATTTCCACAAAGGTCGAG
CTAGGGGAGGCGGCGGCAATATTCATGACAGCCACCCCACCAGGCACTTCAGATCCATTCCCA
GAGTCCAATTCACCAATTTCCGACTTACAGACTGAGATCCCGGATCGAGCTTGGAACTCTGGA
TACGAATGGATCACAGAATACACCGGGAAGACGGTTTGGTTTGTGCCTAGTGTCAAGATGGG
GAATGAGATTGCCCTTTGCCTACAACGTGCTGGAAAGAAAGTAGTCCAATTGAACAGAAAGT
CGTACGAGACGGAGTACCCAAAATGTAAGAACGATGATTGGGACTTTGTTATCACAACAGAC
ATATCTGAAATGGGGGCTAACTTCAAGGCGAGCAGGGTGATTGACAGCCGGAAGAGTGTGAA
ACCAACCATCATAACAGAAGGAGAAGGGAGAGTGATCCTGGGAGAACCATCTGCAGTGACA
GCAGCTAGTGCCGCCCAGAGACGTGGACGTATCGGTAGAAATCCGTCGCAAGTTGGTGATGA
GTACTGTTATGGGGGGCACACGAATGAAGACGACTCGAACTTCGCCCATTGGACTGAGGCAC
GAATCATGCTGGACAACATCAACATGCCAAACGGACTGATCGCTCAATTCTACCAACCAGAGC
GTGAGAAGGTATATACCATGGATGGGGAATACCGGCTCAGAGGAGAAGAGAGAAAAAACTTT
CTGGAACTGTTGAGGACTGCAGATCTGCCAGTTTGGCTGGCTTACAAGGTTGCAGCGGCTGG
AGTGTCATACCACGACCGGAGGTGGTGCTTTGATGGTCCTAGGACAAACACAATTTTAGAAG
ACAACAACGAAGTGGAAGTCATCACGAAGCTTGGTGAAAGGAAGATTCTGAGGCCGCGCTG
GATTGATGCCAGGGTGTACTCGGATCACCAGGCACTAAAGGCGTTCAAGGACTTCGCCTCGG
GAAAACGTTCTCAGATAGGGCTCATTGAGGTTCTGGGAAAGATGCCTGAGCACTTCATGGGG
AAGACATGGGAAGCACTTGACACCATGTACGTTGTGGCCACTGCAGAGAAAGGAGGAAGAG
CTCACAGAATGGCCCTGGAGGAACTGCCAGATGCTCTTCAGACAATTGCCTTGATTGCCTTAT
TGAGTGTGATGACCATGGGAGTATTCTTCCTCCTCATGCAGCGGAAGGGCATTGGAAAGATAG
GTTTGGGAGGCGCTGTCTTGGGAGTCGCGACCTTTTTCTGTTGGATGGCTGAAGTTCCAGGA
ACGAAGATCGCCGGAATGTTGCTGCTCTCCCTTCTCTTGATGATTGTGCTAATTCCTGAGCCAG
AGAAGCAACGTTCGCAGACAGACAACCAGCTAGCCGTGTTCCTGATTTGTGTCATGACCCTT
GTGAGCGCAGTGGCAGCCAACGAGATGGGTTGGCTAGATAAGACCAAGAGTGACATAAGCA
GTTTGTTTGGGCAAAGAATTGAGGTCAAGGAGAATTTCAGCATGGGAGAGTTTCTTCTGGAC
TTGAGGCCGGCAACAGCCTGGTCACTGTACGCTGTGACAACAGCGGTCCTCACTCCACTGCT
AAAGCATTTGATCACGTCAGATTACATCAACACCTCATTGACCTCAATAAACGTTCAGGCAAG
TGCACTATTCACACTCGCGCGAGGCTTCCCCTTCGTCGATGTTGGAGTGTCGGCTCTCCTGCT
AGCAGCCGGATGCTGGGGACAAGTCACCCTCACCGTTACGGTAACAGCGGCAACACTCCTTT
TTTGCCACTATGCCTACATGGTTCCCGGTTGGCAAGCTGAGGCAATGCGCTCAGCCCAGCGGC
GGACAGCGGCCGGAATCATGAAGAACGCTGTAGTGGATGGCATCGTGGCCACGGACGTCCCA
GAATTAGAGCGCACCACACCCATCATGCAGAAGAAAGTTGGACAGATCATGCTGATCTTGGTG

TCTCTAGCTGCAGTAGTAGTGAACCCGTCTGTGAAGACAGTACGAGAAGCCGGAATTTTGATC
ACGGCCGCAGCGGTGACGCTTTGGGAGAATGGAGCAAGCTCTGTTTGGAACGCAACAACTG
CCATCGGACTCTGCCACATCATGCGTGGGGGTTGGTTGTCATGTCTATCCATAACATGGACACT
CATAAAGAACATGGAAAAACCAGGACTAAAAAGAGGTGGGGCAAAAGGACGCACCTTGGGA
GAGGTTTGGAAAGAAAGACTCAACCAGATGACAAAAGAAGAGTTCACT

>wt-NY99_**Fragment-III**
GGTGGGGCAAAAGGACGCACCTTGGGAGAGGTTTGGAAAGAAAGACTCAACCAGATGACAA
AAGAAGAGTTCACTAGGTACCGCAAAGAGGCCATCATCGAAGTCGATCGCTCAGCGGCAAAA
CACGCCAGGAAAGAAGGCAATGTCACTGGAGGGCATCCAGTCTCTAGGGGCACAGCAAAAC
TGAGATGGCTGGTCGAACGGAGGTTTCTCGAACCGGTCGGAAAAGTGATTGACCTTGGATGT
GGAAGAGGCGGTTGGTGTTACTATATGGCAACCCAAAAAAGAGTCCAAGAAGTCAGAGGGTA
CACAAAGGGCGGTCCCGGACATGAAGAGCCCCAACTAGTGCAAAGTTATGGATGGAACATTG
TCACCATGAAGAGTGGAGTGGATGTGTTCTACAGACCTTCTGAGTGTTGTGACACCCTCCTTT
GTGACATCGGAGAGTCCTCGTCAAGTGCTGAGGTTGAAGAGCATAGGACGATTCGGGTCCTT
GAAATGGTTGAGGACTGGCTGCACCGAGGGCCAAGGGAATTTTGCGTGAAGGTGCTCTGTCC
CTACATGCCGAAAGTCATAGAGAAGATGGAGCTGCTCCAACGCCGGTATGGGGGGGGACTGG
TCAGAAACCCACTCTCACGGAATTCCACGCACGAGATGTATTGGGTGAGTCGAGCTTCAGGC
AATGTGGTACATTCAGTGAATATGACCAGCCAGGTGCTCCTAGGAAGAATGGAAAAAAGGAC
CTGGAAGGGACCCCAATACGAGGAAGATGTAAACTTGGGAAGTGGAACCAGGGCGGTGGGA
AAACCCCTGCTCAACTCAGACACCAGTAAAATCAAGAACAGGATTGAACGACTCAGGCGTGA
GTACAGTTCGACGTGGCACCACGATGAGAACCACCCATATAGAACCTGGAACTATCACGGCA
GTTATGATGTGAAGCCCACAGGCTCCGCCAGTTCGCTGGTCAATGGAGTGGTCAGGCTCCTCT
CAAAACCATGGGACACCATCACGAATGTTACCACCATGGCCATGACTGACACTACTCCCTTCG
GGCAGCAGCGAGTGTTCAAAGAGAAGGTGGACACGAAAGCTCCTGAACCGCCAGAAGGAG
TGAAGTACGTGCTCAACGAGACCACCAACTGGTTGTGGGCGTTTTTGGCCAGAGAAAAACGT
CCCAGAATGTGCTCTCGAGAGGAATTCATAAGAAAGGTCAACAGCAATGCAGCTTTGGGTGC
CATGTTTGAAGAGCAGAATCAATGGAGGAGCGCCAGAGAAGCAGTTGAAGATCCAAAATTTT
GGGAGATGGTGGATGAGGAGCGCGAGGCACATCTGCGGGGGGAATGTCACACTTGCATTTAC
AACATGATGGGAAAGAGAGAGAAAAAACCCGGAGAGTTCGGAAAGGCCAAGGGAAGCAGA
GCCATTTGGTTCATGTGGCTCGGAGCTCGCTTTCTGGAGTTCGAGGCTCTGGGTTTTCTCAAT
GAAGACCACTGGCTTGGAAGAAAGAACTCAGGAGGAGGTGTCGAGGGCTTGGGCCTCCAAA
AACTGGGTTACATCCTGCGTGAAGTTGGCACCCGGCCTGGGGGCAAGATCTATGCTGATGACA
CAGCTGGCTGGGACACCCGCATCACGAGAGCTGACTTGGAAAATGAAGCTAAGGTGCTTGAG
CTGCTTGATGGGGAACATCGGCGTCTTGCCAGGGCCATCATTGAGCTCACCTATCGTCACAAA
GTTGTGAAAGTGATGCGCCCGGCTGCTGATGGAAGAACCGTCATGGATGTTATCTCCAGAGAA
GATCAGAGGGGGAGTGGACAAGTTGTCACCTACGCCCTAAACACTTTCACCAACCTGGCCGT
CCAGCTGGTGAGGATGATGGAAGGGGAAGGAGTGATTGGCCCAGATGATGTGGAGAAACTC
ACAAAAGGGAAAGGACCCAAAGTCAGGACCTGGCTGTTTGAGAATGGGGAAGAAAGACTCA
GCCGCATGGCTGTCAGTGGAGATGACTGTGTGGTAAAGCCCCTGGACGATCGCTTTGCCACCT
CGCTCCACTTCCTCAATGCTATGTCAAAGGTTCGCAAAGACATCCAAGAGTGGAAACCGTCA
ACTGGATGGTATGATTGGCAGCAGGTTCCATTTTGCTCAAACCATTTCACTGAATTGATCATGA
AAGATGGAAGAACACTGGTGGTTCCATGCCGAGGACAGGATGAATTGGTAGGCAGAGCTCGC
ATATCTCCAGGGGCCGGATGGAACGTCCGCGACACTGCTTGTCTGGCTAAGTCTTATGCCCAG
ATGTGGCTGCTTCTGTACTTCCACAGAAGAGACCTGCGGCTCATGGCCAACGCCATTTGCTCC
GCTGTCCCTGTGAATTGGGTCCCTACCGGAAGAACCACGTGGTCCATCCATGCAGGAGGAGA
GTGGATGACAACAGAGGACATGTTGGAGGTCTGGAACCGTGTTTGGATAGAGGAGAATGAAT

File S2 ISA fragments

GGATGGAAGACAAAACCCCAGTGGAGAAATGGAGTGACGTCCCATATTCAGGAAAACGAGA
GGACATCTGGTGTGGCAGCCTGATTGGCACAAGAGCCCGAGCCACGTGGGCAGAAAACATCC
AGGTGGCTATCAACCAAGTCAGAGCAATCATCGGAGATGAGAAGTATGTGGACTACATGAGTT
CACTAAAGAGATATGAAGACACAACTTTGGTTGAGGACACAGTACTGTAGATATTTAATCAAT
TGTAAATAGACAATATAAGTATGCATAAAAGTGTAGTTTTATAGTAGTATTTAGTGGTGTTAGTG
TAAATAGTTAAGAAAATTTTGAGGAGAAAGTCAGGCCGGGAAGTTCCCGCCACCGGAAGTTG
AGTAGACGGTGCTGCCTGCGACTCAACCCCAGGAGGACTGGGTGAACAAAGCCGCGAAGTG
ATCCATGTAAGCCCTCAGAACCGTCTCGGAAGGAGGACCCCACATGTTGTAACTTCAAAGCC
CAATGTCAGACCACGCTACGGCGTGCTACTCTGCGGAGAGTGCAGTCTGCGATAGTGCCCCA
GGAGGACTGGGTTAACAAAGGCAAACCAACGCCCCACGCGGCCCTAGCCCCGGTAATGGTGT
TAACCAGGGCGAAAGGACTAGAGGTTAGAGGAGACCCCGCGGTTTAAAGTGCACGGCCCAG
CCTGGCTGAAGCTGTAGGTCAGGGGAAGGACTAGAGGTTAGTGGAGACCCCGTGCCACAAA
ACACCACAACAAAACAGCATATTGACACCTGGGATAGACTAGGAGATCTTCTGCTCTGCACAA
CCAGCCACACGGCACAGTGCGCCGACAATGGTGGCTGGTGGTGCGAGAACACAGGATCT**GG
CCGGCATGGTCCCAGCCTCCTCGCTGGCGCCGGCTGGGCAACATTCCGAGGGGACCGT
CCCCTCGGTAATGGCGAATGGGACTCGCGACAGACATGATAAGATACATTGATGAGTTT
GGACAAACCACAACTAGAATGCAGTGAAAAAAATGCTTTATTTGTGAAATTAAGCGCTG
GCATTGACCCTGAG**

File S2 ISA fragments

E/NS1/NS5+CG

Highlighted in red – pCMV promoter sequence.
Highlighted in green – HDR/SV40pA sequence.

>NY99-CpG-E-NS1_**Fragment-I**
CACCCAACTGATCTTCAGCATCTTCAATATTGGCCATTAGCCATATTATTCATTGGTTATATAGCA
TAAATCAATATTGGCTATTGGCCATTGCATACGTTGTATCTATATCATAATATGTACATTTATATTG
GCTCATGTCCAATATGACCGCCATGTTGGCATTGATTATTGACTAGTTATTAATAGTAATCAATTA
CGGGGTCATTAGTTCATAGCCCATATATGGAGTTCCGCGTTACATAACTTACGGTAAATGGCCC
GCCTGGCTGACCGCCCAACGACCCCCGCCCATTGACGTCAATAATGACGTATGTTCCCATAGT
AACGCCAATAGGGACTTTCCATTGACGTCAATGGGTGGAGTATTTACGGTAAACTGCCCACTT
GGCAGTACATCAAGTGTATCATATGCCAAGTCCGCCCCCTATTGACGTCAATGACGGTAAATGG
CCCGCCTGGCATTATGCCCAGTACATGACCTTACGGGACTTTCCTACTTGGCAGTACATCTACG
TATTAGTCATCGCTATTACCATGGTGATGCGGTTTTGGCAGTACACCAATGGGCGTGGATAGCG
GTTTGACTCACGGGGATTTCCAAGTCTCCACCCCATTGACGTCAATGGGAGTTTGTTTTGGCA
CCAAAATCAACGGGACTTTCCAAAATGTCGTAATAACCCCGCCCCGTTGACGCAAATGGGCG
GTAGGCGTGTACGGTGGGAGGTCTATATAAGCAGAGCTCGTTTAGTGAACCGAGTAGTTCGCC
TGTGTGAGCTGACAAACTTAGTAGTGTTTGTGAGGATTAACAACAATTAACACAGTGCGAGCT
GTTTCTTAGCACGAAGATCTCGATGTCTAAGAAACCAGGAGGGCCCGGCAAGAGCCGGGCTG
TCAATATGCTAAAACGCGGAATGCCCCGCGTGTTGTCCTTGATTGGACTGAAGAGGGCTATGT
TGAGCCTGATCGACGGCAAGGGGCCAATACGATTTGTGTTGGCTCTCTTGGCGTTCTTCAGGT
TCACAGCAATTGCTCCGACCCGAGCAGTGCTGGATCGATGGAGAGGTGTGAACAAACAAACA
GCGATGAAACACCTTCTGAGTTTTAAGAAGGAACTAGGGACCTTGACCAGTGCTATCAATCGG
CGGAGCTCAAAACAAAAGAAAAGAGGAGGAAAGACCGGAATTGCAGTCATGATTGGCCTGA
TCGCCAGCGTAGGAGCAGTTACCCTCTCTAACTTCCAAGGGAAGGTGATGATGACGGTAAATG
CTACTGACGTCACAGATGTCATCACGATTCCAACAGCTGCTGGAAAGAACCTATGCATTGTCA
GAGCAATGGATGTGGGATACATGTGCGATGATACTATCACTTATGAATGCCCAGTACTGTCGGC
TGGTAATGATCCAGAAGACATCGACTGTTGGTGCACAAAGTCAGCAGTCTACGTCAGGTATGG
AAGATGCACCAAGACACGCCACTCAAGACGCAGTCGGAGGTCACTGACAGTGCAGACACAC
GGAGAAAGCACTCTAGCGAACAAGAAGGGGGCTTGGATGGACAGCACCAAGGCCACAAGGT
ATTTGGTAAAAACAGAATCATGGATCTTGAGGAACCCTGGATATGCCCTGGTGGCAGCCGTCA
TTGGTTGGATGCTTGGGAGCAACACCATGCAGAGAGTTGTGTTTGTCGTGCTATTGCTTTTGG
TGGCCCCAGCTTACAGCTTCAACTGCCTTGGAATGAGCAACAGAGACTTTTTGGAAGGCGTG
TCCGGAGCAACGTGGGTCGATTTGGTTCTCGAAGGCGACAGTTGTGTAACGATAATGTCGAA
AGACAAACCAACGATCGACGTCAAGATGATGAACATGGAAGCGGCGAACCTCGCCGAAGTA
CGAAGTTATTGTTATTTGGCGACCGTAAGTGATCTCTCAACCAAAGCGGCGTGTCCGACAATG
GGCGAAGCGCACAACGACAAACGTGCGGATCCCGCATTTGTGTGCCGACAAGGCGTCGTCG
ACCGCGGATGGGGAAACGGATGTGGACTGTTCGGAAAAGGAAGCATCGACACGTGCGCAAA
ATTTGCATGCTCGACGAAAGCGATCGGACGAACGATTTTGAAAGAAAACATTAAATACGAAG
TTGCGATTTTTGTTCACGGACCGACAACAGTAGAATCTCACGGAAATTATTCGACGCAAGTCG
GCGCGACACAAGCCGGTCGACTGAGCATCACGCCCGCGGCGCCGTCGTATACGCTAAAACTC
GGCGAATACGGCGAAGTGACGGTTGATTGCGAACCTCGTTCCGGCATCGACACGAACGCGTA
CTACGTTATGACGGTCGGAACGAAGACTTTTTTAGTTCATCGCGAATGGTTCATGGATCTTAAT
CTACCTTGGAGCAGCGCGGGAAGCACGGTTTGGCGTAATCGCGAAACGTTGATGGAATTTGA
AGAACCTCACGCGACAAAACAGTCCGTTATCGCGTTGGGTTCACAAGAAGGCGCGCTACATC
AAGCGTTGGCCGGCGCGATTCCTGTCGAATTTTCGAGCAATACAGTTAAGTTGACGTCCGGTC

File S2 ISA fragments

ATTTGAAATGTCGCGTGAAAATGGAAAAATTGCAATTGAAAGGAACGACATACGGTGTGTGTT
CGAAAGCGTTCAAATTTCTCGGAACGCCCGCGGATACCGGTCACGGAACTGTTGTTTTAGAAT
TGCAATACACTGGAACGGACGGACCTTGTAAAGTTCCGATCTCATCGGTCGCGTCATTGAACG
ATCTGACACCGGTCGGACGATTAGTGACTGTGAACCCGTTTGTTTCGGTCGCGACGGCAAATG
CAAAAGTTCTGATCGAATTGGAACCGCCGTTTGGCGACTCGTACATCGTTGTTGGACGCGGCG
AACAACAAATTAATCATCATTGGCACAAATCGGGAAGCAGCATCGGAAAAGCGTTCACGACG
ACGCTCAAAGGCGCTCAGCGACTTGCCGCGCTCGGTGATACGGCGTGGGATTTTGGATCCGTC
GGCGGGGTTTTCACGTCGGTAGGGAAGGCTGTTCATCAAGTATTCGGCGGCGCGTTTCGTTCG
CTTTTTGGCGGAATGTCGTGGATTACTCAAGGATTACTCGGCGCGCTTCTGTTGTGGATGGGA
ATTAACGCACGAGATCGATCGATCGCGCTCACATTTCTCGCTGTCGGAGGAGTTCTGCTCTTCC
TCTCCGTGAACGTGCACGCTGACACTGGGTGTGCCATAGACATCAGCCGGCAAGAGCTACGA
TGCGGAAGTGGCGTGTTTATTCACAACGACGTCGAAGCGTGGATGGATCGGTACAAGTATTAT
CCGGAAACGCCGCAAGGTCTTGCGAAAATTATACAGAAAGCGCACAAAGAAGGCGTTTGCG
GTCTACGATCGGTTTCTCGACTCGAACATCAAATGTGGGAAGCAGTGAAGGACGAGCTAAAC
ACGCTTTTAAAAGAAAACGGTGTCGATCTCAGCGTCGTCGTTGAAAAACAAGAGGGAATGTA
CAAGTCGGCGCCGAAACGACTCACCGCAACGACGGAAAAATTGGAAATTGGATGGAAGGCG
TGGGGAAAAAGCATTTTATTCGCGCCTGAACTCGCGAACAATACGTTCGTCGTCGACGGTCCT
GAAACGAAAGAATGTCCAACGCAAAATCGCGCGTGGAACAGTTTAGAAGTCGAGGATTTCGG
ATTCGGTCTCACGAGCACGCGGATGTTTCTTAAGGTCCGAGAGAGCAACACGACGGAATGCG
ACTCGAAAATAATTGGAACGGCGGTTAAAAACAATTTGGCGATACACAGCGATCTTTCTTATT
GGATCGAAAGTCGGCTGAACGATACTTGGAAACTCGAACGCGCCGTTCTCGGCGAAGTAAAA
TCTTGCACGTGGCCGGAGACGCATACGTTGTGGGGTGACGGAATTCTCGAAAGCGATTTGATA
ATTCCCGTGACCCTCGCGGGACCGCGAAGCAATCACAATCGTCGACCGGGGTATAAAACGCA
AAATCAGGGTCCATGGGACGAAGGACGCGTCGAGATTGATTTCGATTATTGTCCCGGAACAAC
GGTGACACTGAGTGAGAGTTGTGGACATCGCGGACCCGCGACGCGAACGACGACGGAGAGT
GGAAAGTTGATTACGGATTGGTGCTGTCGTAGTTGCACCTTGCCTCCGCTTCGTTACCAAACC
GACAGCGGATGTTGGTACGGGATGGAAATACGACCGCAACGACACGATGAAAAGACCCTCGT
GCAGTCACAAGTGAATGCTTATAATGCTGATATGATTGACCCTTTTCAGTTGGGCCTTCTGGTC
GTGTTCTTGGCCACCCAGGAGGTCCTTCGC

>wt-NY99_**Fragment-II**
TATAATGCTGATATGATTGACCCTTTTCAGTTGGGCCTTCTGGTCGTGTTCTTGGCCACCCAGG
AGGTCCTTCGCAAGAGGTGGACAGCCAAGATCAGCATGCCAGCTATACTGATTGCTCTGCTAG
TCCTGGTGTTTGGGGGCATTACTTACACTGATGTGTTACGCTATGTCATCTTGGTGGGGGCAGC
TTTCGCAGAATCTAATTCGGGAGGAGACGTGGTACACTTGGCGCTCATGGCGACCTTCAAGAT
ACAACCAGTGTTTATGGTGGCATCGTTTCTCAAAGCGAGATGGACCAACCAGGAGAACATTTT
GTTGATGTTGGCGGCTGTTTTCTTTCAAATGGCTTATCACGATGCCCGCCAAATTCTGCTCTGG
GAGATCCCTGATGTGTTGAATTCACTGGCGGTAGCTTGGATGATACTGAGAGCCATAACATTCA
CAACGACATCAAACGTGGTTGTTCCGCTGCTAGCCCTGCTAACACCCGGGCTGAGATGCTTGA
ATCTGGATGTGTACAGGATACTGCTGTTGATGGTCGGAATAGGCAGCTTGATCAGGGAGAAGA
GGAGTGCAGCCGCAAAAAAGAAAGGAGCAAGTCTGCTATGCTTGGCTCTAGCCTCAACAGG
ACTTTTCAACCCCATGATCCTTGCTGCTGGACTGATTGCATGTGATCCCAACCGTAAACGCGG
ATGGCCCGCAACTGAAGTGATGACAGCTGTCGGCCTAATGTTTGCCATCGTCGGAGGGCTGGC
AGAGCTTGACATTGACTCCATGGCCATTCCAATGACTATCGCGGGGCTCATGTTTGCTGCTTTC
GTGATTTCTGGGAAATCAACAGATATGTGGATTGAGAGAACGGCGGACATTTCCTGGGAAAGT
GATGCAGAAATTACAGGCTCGAGCGAAAGAGTTGATGTGCGGCTTGATGATGATGGAAACTT
CCAGCTCATGAATGATCCAGGAGCACCTTGGAAGATATGGATGCTCAGAATGGTCTGTCTCGC

File S2 ISA fragments

GATTAGTGCGTACACCCCCTGGGCAATCTTGCCCTCAGTAGTTGGATTTTGGATAACTCTCCAA
TACACAAAGAGAGGAGGCGTGTTGTGGGACACTCCCTCACCAAAGGAGTACAAAAAGGGGG
ACACGACCACCGGCGTCTACAGGATCATGACTCGTGGGCTGCTCGGCAGTTATCAAGCAGGA
GCGGGCGTGATGGTTGAAGGTGTTTTCCACACCCTTTGGCATACAACAAAAGGAGCCGCTTT
GATGAGCGGAGAGGGCCGCCTGGACCCATACTGGGGCAGTGTCAAGGAGGATCGACTTTGTT
ACGGAGGACCCTGGAAATTGCAGCACAAGTGGAACGGGCAGGATGAGGTGCAGATGATTGT
GGTGGAACCTGGCAAGAACGTTAAGAACGTCCAGACGAAACCAGGGGTGTTCAAAACACCT
GAAGGAGAAATCGGGGCCGTGACTTTGGACTTCCCCACTGGAACATCAGGCTCACCAATAGT
GGACAAAAACGGTGATGTGATTGGGCTTTATGGCAATGGAGTCATAATGCCCAACGGCTCATA
CATAAGCGCGATAGTGCAGGGTGAAAGGATGGATGAGCCAATCCCAGCCGGATTCGAACCTG
AGATGCTGAGGAAAAAACAGATCACTGTACTGGATCTCCATCCCGGCGCCGGTAAAACAAGG
AGGATTCTGCCACAGATCATCAAAGAGGCCATAAACAGAAGACTGAGAACAGCCGTGCTAGC
GCCAACCAGGGTTGTGGCTGCTGAGATGGCTGAAGCACTGAGAGGACTGCCCATCCGGTACC
AGACATCCGCAGTGCCCAGAGAACATAATGGAAATGAGATTGTTGATGTCATGTGTCATGCTA
CCCTCACCCACAGGCTGATGTCTCCTCACAGGGTGCCGAACTACAACCTGTTCGTGATGGATG
AGGCTCATTTCACCGACCCAGCTAGCATTGCAGCAAGAGGTTACATTTCCACAAAGGTCGAG
CTAGGGGAGGCGGCGGCAATATTCATGACAGCCACCCCACCAGGCACTTCAGATCCATTCCCA
GAGTCCAATTCACCAATTTCCGACTTACAGACTGAGATCCCGGATCGAGCTTGGAACTCTGGA
TACGAATGGATCACAGAATACACCGGGAAGACGGTTTGGTTTGTGCCTAGTGTCAAGATGGG
GAATGAGATTGCCCTTTGCCTACAACGTGCTGGAAAGAAAGTAGTCCAATTGAACAGAAAGT
CGTACGAGACGGAGTACCCAAAATGTAAGAACGATGATTGGGACTTTGTTATCACAACAGAC
ATATCTGAAATGGGGGCTAACTTCAAGGCGAGCAGGGTGATTGACAGCCGGAAGAGTGTGAA
ACCAACCATCATAACAGAAGGAGAAGGGAGAGTGATCCTGGGAGAACCATCTGCAGTGACA
GCAGCTAGTGCCGCCCAGAGACGTGGACGTATCGGTAGAAATCCGTCGCAAGTTGGTGATGA
GTACTGTTATGGGGGGCACACGAATGAAGACGACTCGAACTTCGCCCATTGGACTGAGGCAC
GAATCATGCTGGACAACATCAACATGCCAAACGGACTGATCGCTCAATTCTACCAACCAGAGC
GTGAGAAGGTATATACCATGGATGGGGAATACCGGCTCAGAGGAGAAGAGAGAAAAAACTTT
CTGGAACTGTTGAGGACTGCAGATCTGCCAGTTTGGCTGGCTTACAAGGTTGCAGCGGCTGG
AGTGTCATACCACGACCGGAGGTGGTGCTTTGATGGTCCTAGGACAAACACAATTTTAGAAG
ACAACAACGAAGTGGAAGTCATCACGAAGCTTGGTGAAAGGAAGATTCTGAGGCCGCGCTG
GATTGATGCCAGGGTGTACTCGGATCACCAGGCACTAAAGGCGTTCAAGGACTTCGCCTCGG
GAAAACGTTCTCAGATAGGGCTCATTGAGGTTCTGGGAAAGATGCCTGAGCACTTCATGGGG
AAGACATGGGAAGCACTTGACACCATGTACGTTGTGGCCACTGCAGAGAAAGGAGGAAGAG
CTCACAGAATGGCCCTGGAGGAACTGCCAGATGCTCTTCAGACAATTGCCTTGATTGCCTTAT
TGAGTGTGATGACCATGGGAGTATTCTTCCTCCTCATGCAGCGGAAGGGCATTGGAAAGATAG
GTTTGGGAGGCGCTGTCTTGGGAGTCGCGACCTTTTTCTGTTGGATGGCTGAAGTTCCAGGA
ACGAAGATCGCCGGAATGTTGCTGCTCTCCCTTCTCTTGATGATTGTGCTAATTCCTGAGCCAG
AGAAGCAACGTTCGCAGACAGACAACCAGCTAGCCGTGTTCCTGATTTGTGTCATGACCCTT
GTGAGCGCAGTGGCAGCCAACGAGATGGGTTGGCTAGATAAGACCAAGAGTGACATAAGCA
GTTTGTTTGGGCAAAGAATTGAGGTCAAGGAGAATTTCAGCATGGGAGAGTTTCTTCTGGAC
TTGAGGCCGGCAACAGCCTGGTCACTGTACGCTGTGACAACAGCGGTCCTCACTCCACTGCT
AAAGCATTTGATCACGTCAGATTACATCAACACCTCATTGACCTCAATAAACGTTCAGGCAAG
TGCACTATTCACACTCGCGCGAGGCTTCCCCTTCGTCGATGTTGGAGTGTCGGCTCTCCTGCT
AGCAGCCGGATGCTGGGGACAAGTCACCCTCACCGTTACGGTAACAGCGGCAACACTCCTTT
TTTGCCACTATGCCTACATGGTTCCCGGTTGGCAAGCTGAGGCAATGCGCTCAGCCCAGCGGC
GGACAGCGGCCGGAATCATGAAGAACGCTGTAGTGGATGGCATCGTGGCCACGGACGTCCCA
GAATTAGAGCGCACCACACCCATCATGCAGAAGAAAGTTGGACAGATCATGCTGATCTTGGTG

File S2 ISA fragments

TCTCTAGCTGCAGTAGTAGTGAACCCGTCTGTGAAGACAGTACGAGAAGCCGGAATTTTGATC
ACGGCCGCAGCGGTGACGCTTTGGGAGAATGGAGCAAGCTCTGTTTGGAACGCAACAACTG
CCATCGGACTCTGCCACATCATGCGTGGGGGTTGGTTGTCATGTCTATCCATAACATGGACACT
CATAAAGAACATGGAAAAACCAGGACTAAAAAGAGGTGGGGCAAAAGGACGCACCTTGGGA
GAGGTTTGGAAAGAAAGACTCAACCAGATGACAAAAGAAGAGTTCACT

>NY99-CpG-NS5_**Fragment-III**
GGTGGGGCAAAAGGACGCACCTTGGGAGAGGTTTGGAAAGAAAGACTCAACCAGATGACAA
AAGAAGAGTTCACTAGGTACCGCAAAGAAGCGATCATAGAAGTCGATCGATCGGCAGCGAAA
CACGCGCGAAAAGAAGGAAACGTAACGGGCGGTCATCCCGTTTCACGCGGGACGGCGAAAC
TTCGATGGCTTGTAGAACGGCGGTTTCTCGAACCGGTCGGAAAAGTGATAGATCTAGGATGCG
GACGCGGAGGTTGGTGTTATTACATGGCGACACAAAAACGCGTTCAAGAAGTCCGTGGGTAC
ACGAAAGGAGGTCCGGGACACGAAGAACCGCAACTCGTTCAAAGTTACGGATGGAACATCG
TCACCATGAAGAGCGGCGTCGACGTTTTTTATCGACCTTCGGAATGTTGCGACACACTTCTAT
GTGACATTGGCGAGTCTTCATCAAGTGCGGAAGTCGAAGAACATCGCACAATTCGCGTTCTAG
AAATGGTCGAAGACTGGCTACATCGCGGACCGCGCGAATTTTGTGTGAAAGTGCTCTGTCCGT
ACATGCCGAAAGTTATCGAAAAGATGGAACTGCTTCAACGTCGTTATGGCGGAGGACTCGTG
CGAAATCCGCTGTCTCGGAATTCGACGCACGAAATGTATTGGGTGAGTCGAGCGTCGGGTAAT
GTCGTCCATTCGGTAAACATGACGAGCCAAGTTCTCCTCGGACGAATGGAAAAACGAACGTG
GAAGGGACCGCAATACGAAGAAGATGTCAATTTGGGAAGCGGAACCCGAGCGGTCGGAAAA
CCGCTTCTGAATTCCGACACGAGCAAAATCAAAAACCGAATCGAACGACTCCGACGCGAATA
CAGTTCGACATGGCATCACGATGAGAATCATCCTTATCGAACCTGGAATTATCACGGGAGTTAC
GACGTGAAGCCGACGGGATCGGCCAGTTCGCTCGTGAACGGAGTGGTTCGACTTCTGTCAAA
ACCGTGGGACACGATAACGAACGTAACGACAATGGCCATGACGGACACGACTCCGTTCGGTC
AGCAACGCGTTTTCAAAGAGAAAGTTGACACGAAAGCGCCGGAACCGCCGGAAGGCGTGAA
GTACGTGCTTAACGAAACGACCAATTGGTTGTGGGCGTTTTTGGCACGCGAAAAACGACCTC
GAATGTGTTCGCGAGAAGAATTCATTCGAAAAGTCAACAGCAACGCGGCATTGGGCGCGATG
TTCGAAGAGCAAAATCAATGGCGGAGCGCGCGAGAAGCGGTTGAAGATCCCAAATTTTGGGA
AATGGTGGACGAGGAGCGAGAAGCGCATCTGCGCGGCGAATGTCACACGTGCATTTACAACA
TGATGGGAAAGCGCGAGAAAAAACCAGGCGAATTTGGAAAAGCAAAAGGAAGCCGCGCGAT
TTGGTTCATGTGGCTCGGCGCTCGATTTCTCGAATTTGAGGCGCTCGGTTTTCTCAACGAAGA
TCATTGGCTAGGACGAAAAAATTCCGGAGGCGGCGTCGAAGGATTGGGTCTGCAAAAACTCG
GTTACATTCTTCGTGAAGTTGGAACGCGGCCAGGCGGAAAGATCTACGCGGACGACACGGCA
GGATGGGACACGCGAATTACGCGAGCGGATTTGGAAAACGAAGCGAAAGTACTCGAGCTGCT
TGATGGCGAACATCGTCGTCTCGCGCGAGCCATCATCGAGCTGACGTATCGTCACAAAGTCGT
CAAAGTGATGCGGCCGGCGGCGGACGGACGAACGGTGATGGACGTCATCTCGCGAGAAGAT
CAGCGTGGGAGTGGACAAGTCGTTACATACGCGCTCAACACGTTCACCAATCTCGCCGTGCA
ACTAGTTCGAATGATGGAAGGAGAAGGCGTGATCGGACCGGATGACGTCGAAAAACTTACAA
AAGGCAAAGGACCGAAAGTACGGACGTGGCTGTTCGAAAATGGAGAAGAACGACTGAGTCG
AATGGCGGTTAGTGGCGACGATTGCGTCGTTAAACCGCTCGACGATCGATTCGCGACCTCGCT
TCATTTCCTTAACGCGATGTCGAAGGTTCGAAAAGACATTCAAGAATGGAAACCCTCGACGG
GATGGTATGATTGGCAACAAGTTCCGTTTTGTTCGAATCATTTCACCGAATTGATAATGAAAGA
CGGACGAACGCTTGTTGTTCCTTGTCGAGGACAAGATGAATTGGTCGGGCGCGCACGAATTT
CGCCGGGCGCAGGATGGAATGTTCGCGACACGGCGTGTCTCGCAAAATCGTACGCGCAGATG
TGGCTGCTTCTGTATTTTCATCGACGCGATCTTCGACTCATGGCGAACGCGATTTGTTCGGCAG
TTCCGGTGAATTGGGTTCCAACGGGACGAACAACGTGGTCAATCCACGCGGGCGGTGAGTGG
ATGACAACGGAAGACATGTTGGAAGTCTGGAACCGCGTTTGGATAGAAGAGAATGAATGGAT

File S2 ISA fragments

GGAAGACAAAACGCCGGTCGAGAAATGGAGCGATGTTCCGTATTCGGGAAAACGCGAGGAC
ATTTGGTGCGGGAGTCTGATCGGAACGCGAGCGCGCGCCACGTGGGCGGAAAACATTCAAGT
CGCAATCAATCAAGTCCGTGCGATCATCGGAGATGAGAAGTATGTGGACTACATGAGTTCACT
AAAGAGATATGAAGACACAACTTTGGTTGAGGACACAGTACTGTAGATATTTAATCAATTGTA
AATAGACAATATAAGTATGCATAAAAGTGTAGTTTTATAGTAGTATTTAGTGGTGTTAGTGTAAA
TAGTTAAGAAAATTTTGAGGAGAAAGTCAGGCCGGGAAGTTCCCGCCACCGGAAGTTGAGTA
GACGGTGCTGCCTGCGACTCAACCCCAGGAGGACTGGGTGAACAAAGCCGCGAAGTGATCC
ATGTAAGCCCTCAGAACCGTCTCGGAAGGAGGACCCCACATGTTGTAACTTCAAAGCCCAAT
GTCAGACCACGCTACGGCGTGCTACTCTGCGGAGAGTGCAGTCTGCGATAGTGCCCCAGGAG
GACTGGGTTAACAAAGGCAAACCAACGCCCCACGCGGCCCTAGCCCCGGTAATGGTGTTAAC
CAGGGCGAAAGGACTAGAGGTTAGAGGAGACCCCGCGGTTTAAAGTGCACGGCCCAGCCTG
GCTGAAGCTGTAGGTCAGGGGAAGGACTAGAGGTTAGTGGAGACCCCGTGCCACAAAACAC
CACAACAAAACAGCATATTGACACCTGGGATAGACTAGGAGATCTTCTGCTCTGCACAACCAG
CCACACGGCACAGTGCGCCGACAATGGTGGCTGGTGGTGCGAGAACACAGGATCTGGCCGG
CATGGTCCCAGCCTCCTCGCTGGCGCCGGCTGGGCAACATTCCGAGGGGACCGTCCCCTCGG
TAATGGCGAATGGGACTCGCGACAGACATGATAAGATACATTGATGAGTTTGGACAAACCACA
ACTAGAATGCAGTGAAAAAAATGCTTTATTTGTGAAATTAAGCGCTGGCATTGACCCTGAG

File S2 ISA fragments

E-MAX

Highlighted in red – pCMV promoter sequence.
Highlighted in green – HDR/SV40pA sequence.

>NY99-E_CpG+UpA_**Fragment-I**
CACCCAACTGATCTTCAGCATCTTCAATATTGGCCATTAGCCATATTATTCATTGGTTATATAGCA
TAAATCAATATTGGCTATTGGCCATTGCATACGTTGTATCTATATCATAATATGTACATTTATATTG
GCTCATGTCCAATATGACCGCCATGTTGGCATTGATTATTGACTAGTTATTAATAGTAATCAATTA
CGGGGTCATTAGTTCATAGCCCATATATGGAGTTCCGCGTTACATAACTTACGGTAAATGGCCC
GCCTGGCTGACCGCCCAACGACCCCCGCCCATTGACGTCAATAATGACGTATGTTCCCATAGT
AACGCCAATAGGGACTTTCCATTGACGTCAATGGGTGGAGTATTTACGGTAAACTGCCCACTT
GGCAGTACATCAAGTGTATCATATGCCAAGTCCGCCCCCTATTGACGTCAATGACGGTAAATGG
CCCGCCTGGCATTATGCCCAGTACATGACCTTACGGGACTTTCCTACTTGGCAGTACATCTACG
TATTAGTCATCGCTATTACCATGGTGATGCGGTTTTGGCAGTACACCAATGGGCGTGGATAGCG
GTTTGACTCACGGGGATTTCCAAGTCTCCACCCCATTGACGTCAATGGGAGTTTGTTTTGGCA
CCAAAATCAACGGGACTTTCCAAAATGTCGTAATAACCCCGCCCCGTTGACGCAAATGGGCG
GTAGGCGTGTACGGTGGGAGGTCTATATAAGCAGAGCTCGTTTAGTGAACCGAGTAGTTCGCC
TGTGTGAGCTGACAAACTTAGTAGTGTTTGTGAGGATTAACAACAATTAACACAGTGCGAGCT
GTTTCTTAGCACGAAGATCTCGATGTCTAAGAAACCAGGAGGGCCCGGCAAGAGCCGGGCTG
TCAATATGCTAAAACGCGGAATGCCCCGCGTGTTGTCCTTGATTGGACTGAAGAGGGCTATGT
TGAGCCTGATCGACGGCAAGGGGCCAATACGATTTGTGTTGGCTCTCTTGGCGTTCTTCAGGT
TCACAGCAATTGCTCCGACCCGAGCAGTGCTGGATCGATGGAGAGGTGTGAACAAACAAACA
GCGATGAAACACCTTCTGAGTTTTAAGAAGGAACTAGGGACCTTGACCAGTGCTATCAATCGG
CGGAGCTCAAAACAAAAGAAAAGAGGAGGAAAGACCGGAATTGCAGTCATGATTGGCCTGA
TCGCCAGCGTAGGAGCAGTTACCCTCTCTAACTTCCAAGGGAAGGTGATGATGACGGTAAATG
CTACTGACGTCACAGATGTCATCACGATTCCAACAGCTGCTGGAAAGAACCTATGCATTGTCA
GAGCAATGGATGTGGGATACATGTGCGATGATACTATCACTTATGAATGCCCAGTACTGTCGGC
TGGTAATGATCCAGAAGACATCGACTGTTGGTGCACAAAGTCAGCAGTCTACGTCAGGTATGG
AAGATGCACCAAGACACGCCACTCAAGACGCAGTCGGAGGTCACTGACAGTGCAGACACAC
GGAGAAAGCACTCTAGCGAACAAGAAGGGGGCTTGGATGGACAGCACCAAGGCCACAAGGT
ATTTGGTAAAAACAGAATCATGGATCTTGAGGAACCCTGGATATGCCCTGGTGGCAGCCGTCA
TTGGTTGGATGCTTGGGAGCAACACCATGCAGAGAGTTGTGTTTGTCGTGCTATTGCTTTTGG
TGGCCCCAGCTTACAGCTTCAACTGCCTTGGAATGAGCAACAGAGACTTTTTGGAAGGCGTT
TCCGGCGCGACGTGGGTCGATTTAGTTCTCGAAGGGGATAGTTGCGTTACGATAATGTCGAAA
GACAAACCGACGATCGACGTTAAAATGATGAATATGGAGGCCGCGAATCTAGCGGAAGTTCG
CAGTTATTGTTATTTAGCAACCGTAAGCGATCTTTCGACGAAAGCCGCGTGTCCGACGATGGG
CGAAGCGCACAACGATAAACGGGCGGATCCCGCGTTTGTTTGTCGACAAGGCGTCGTCGATC
GCGGTTGGGGAAATGGTTGTGGACTATTCGGCAAAGGAAGTATCGATACGTGCGCGAAATTTG
CGTGTTCTACTAAAGCGATAGGACGAACGATATTAAAAGAAAATATCAAATACGAAGTCGCGA
TTTTTGTCCACGGACCGACGACGGTCGAATCGCACGGAAATTATTCGACGCAAGTCGGCGCG
ACGCAGGCCGGTCGACTAAGTATAACGCCCGCGGCGCCGTCGTACACGCTAAAACTGGGCGA
ATATGGCGAGGTTACGGTCGACTGCGAACCGCGTTCGGGTATCGATACGAACGCGTATTATGTT
ATGACGGTCGGAACGAAAACGTTTTAGTACATCGGGAATGGTTCATGGATCTAAACCTACCG
TGGAGCAGTGCCGGAAGTACCGTTTGGCGTAACCGGGAAACGTTAATGGAATTCGAAGAACC
GCACGCGACTAAACAGTCCGTAATCGCGTTAGGATCGCAAGAAGGCGCGCTTCATCAAGCGT
TAGCCGGCGCGATTCCGGTCGAATTTTCGAGTAATACCGTCAAATTAACATCCGGTCATTTGAA

File S2 ISA fragments

ATGTCGGGTCAAAATGGAAAAATTACAATTAAAAGGAACGACTTACGGCGTATGTTCGAAAG
CGTTCAAGTTTCTCGGTACACCCGCCGATACCGGTCACGGAACCGTCGTTTTGGAATTACAAT
ATACCGGAACCGATGGACCGTGTAAAGTTCCGATATCGTCCGTGGCGTCGTTGAACGATCTAA
CACCCGTCGGCCGATTAGTCACCGTTAATCCGTTCGTTTCCGTCGCGACGGCGAACGCGAAAG
TACTTATAGAATTGGAACCGCCGTTTGGCGATTCGTATATCGTCGTCGGCCGCGGGGAACAAC
AAATAAATCACCATTGGCATAAGTCCGGAAGTAGTATCGGAAAAGCGTTTACGACGACGCTAA
AAGGCGCGCAACGACTAGCCGCGCTCGGCGATACCGCGTGGGATTTCGGATCCGTCGGGGGC
GTTTTTACGTCCGTCGGTAAAGCCGTACATCAAGTATTCGGCGGGGCGTTTCGCTCGCTTTTCG
GCGGAATGTCGTGGATAACGCAAGGATTACTGGGCGCGCTACTTTTATGGATGGGAATAAACG
CGCGGGATCGTTCGATCGCGCTAACGTTTCTCGCCGTCGGCGGAGTTCTGCTCTTCCTCTCCG
TGAACGTGCACGCTGACACTGGGTGTGCCATAGACATCAGCCGGCAAGAGCTGAGATGTGGA
AGTGGAGTGTTCATACACAATGATGTGGAGGCTTGGATGGACCGGTACAAGTATTACCCTGAA
ACGCCACAAGGCCTAGCCAAGATCATTCAGAAAGCTCATAAGGAAGGAGTGTGCGGTCTACG
ATCAGTTTCCAGACTGGAGCATCAAATGTGGGAAGCAGTGAAGGACGAGCTGAACACTCTTT
TGAAGGAGAATGGTGTGGACCTTAGTGTCGTGGTTGAGAAACAGGAGGGAATGTACAAGTCA
GCACCTAAACGCCTCACCGCCACCACGGAAAAATTGGAAATTGGCTGGAAGGCCTGGGGAA
AGAGTATTTTATTTGCACCAGAACTCGCCAACAACACCTTTGTGGTTGATGGTCCGGAGACCA
AGGAATGTCCGACTCAGAATCGCGCTTGGAATAGCTTAGAAGTGGAGGATTTTGGATTTGGTC
TCACCAGCACTCGGATGTTCCTGAAGGTCAGAGAGAGCAACACAACTGAATGTGACTCGAAG
ATCATTGGAACGGCTGTCAAGAACAACTTGGCGATCCACAGTGACCTGTCCTATTGGATTGAA
AGCAGGCTCAATGATACGTGGAAGCTTGAAAGGGCAGTTCTGGGTGAAGTCAAATCATGTAC
GTGGCCTGAGACGCATACCTTGTGGGGCGATGGAATCCTTGAGAGTGACTTGATAATACCAGT
CACACTGGCGGGACCACGAAGCAATCACAATCGGAGACCTGGGTACAAGACACAAAACCAG
GGCCCATGGGACGAAGGCCGGGTAGAGATTGACTTCGATTACTGCCCAGGAACTACGGTCAC
CCTGAGTGAGAGCTGCGGACACCGTGGACCTGCCACTCGCACCACCACAGAGAGCGGAAAG
TTGATAACAGATTGGTGCTGCAGGAGCTGCACCTTACCACCACTGCGCTACCAAACTGACAGC
GGCTGTTGGTATGGTATGGAGATCAGACCACAGAGACATGATGAAAAGACCCTCGTGCAGTC
ACAAGTGAATGCTTATAATGCTGATATGATTGACCCTTTTCAGTTGGGCCTTCTGGTCGTGTTC
TTGGCCACCCAGGAGGTCCTTCGC

>wt-NY99_**Fragment-II**
TATAATGCTGATATGATTGACCCTTTTCAGTTGGGCCTTCTGGTCGTGTTCTTGGCCACCCAGG
AGGTCCTTCGCAAGAGGTGGACAGCCAAGATCAGCATGCCAGCTATACTGATTGCTCTGCTAG
TCCTGGTGTTTGGGGGCATTACTTACACTGATGTGTTACGCTATGTCATCTTGGTGGGGGCAGC
TTTCGCAGAATCTAATTCGGGAGGAGACGTGGTACACTTGGCGCTCATGGCGACCTTCAAGAT
ACAACCAGTGTTTATGGTGGCATCGTTTCTCAAAGCGAGATGGACCAACCAGGAGAACATTTT
GTTGATGTTGGCGGCTGTTTTCTTTCAAATGGCTTATCACGATGCCCGCCAAATTCTGCTCTGG
GAGATCCCTGATGTGTTGAATTCACTGGCGGTAGCTTGGATGATACTGAGAGCCATAACATTCA
CAACGACATCAAACGTGGTTGTTCCGCTGCTAGCCCTGCTAACACCCGGGCTGAGATGCTTGA
ATCTGGATGTGTACAGGATACTGCTGTTGATGGTCGGAATAGGCAGCTTGATCAGGGAGAAGA
GGAGTGCAGCCGCAAAAAAGAAAGGAGCAAGTCTGCTATGCTTGGCTCTAGCCTCAACAGG
ACTTTTCAACCCCATGATCCTTGCTGCTGGACTGATTGCATGTGATCCCAACCGTAAACGCGG
ATGGCCCGCAACTGAAGTGATGACAGCTGTCGGCCTAATGTTTGCCATCGTCGGAGGGCTGGC
AGAGCTTGACATTGACTCCATGGCCATTCCAATGACTATCGCGGGGCTCATGTTTGCTGCTTTC
GTGATTTCTGGGAAATCAACAGATATGTGGATTGAGAGAACGGCGGACATTTCCTGGGAAAGT
GATGCAGAAATTACAGGCTCGAGCGAAAGAGTTGATGTGCGGCTTGATGATGATGGAAACTT
CCAGCTCATGAATGATCCAGGAGCACCTTGGAAGATATGGATGCTCAGAATGGTCTGTCTCGC

File S2 ISA fragments

GATTAGTGCGTACACCCCCTGGGCAATCTTGCCCTCAGTAGTTGGATTTTGGATAACTCTCCAA
TACACAAAGAGAGGAGGCGTGTTGTGGGACACTCCCTCACCAAAGGAGTACAAAAAGGGGG
ACACGACCACCGGCGTCTACAGGATCATGACTCGTGGGCTGCTCGGCAGTTATCAAGCAGGA
GCGGGCGTGATGGTTGAAGGTGTTTTCCACACCCTTTGGCATACAACAAAAGGAGCCGCTTT
GATGAGCGGAGAGGGCCGCCTGGACCCATACTGGGGCAGTGTCAAGGAGGATCGACTTTGTT
ACGGAGGACCCTGGAAATTGCAGCACAAGTGGAACGGGCAGGATGAGGTGCAGATGATTGT
GGTGGAACCTGGCAAGAACGTTAAGAACGTCCAGACGAAACCAGGGGTGTTCAAAACACCT
GAAGGAGAAATCGGGGCCGTGACTTTGGACTTCCCCACTGGAACATCAGGCTCACCAATAGT
GGACAAAAACGGTGATGTGATTGGGCTTTATGGCAATGGAGTCATAATGCCCAACGGCTCATA
CATAAGCGCGATAGTGCAGGGTGAAAGGATGGATGAGCCAATCCCAGCCGGATTCGAACCTG
AGATGCTGAGGAAAAAACAGATCACTGTACTGGATCTCCATCCCGGCGCCGGTAAAACAAGG
AGGATTCTGCCACAGATCATCAAAGAGGCCATAAACAGAAGACTGAGAACAGCCGTGCTAGC
GCCAACCAGGGTTGTGGCTGCTGAGATGGCTGAAGCACTGAGAGGACTGCCCATCCGGTACC
AGACATCCGCAGTGCCCAGAGAACATAATGGAAATGAGATTGTTGATGTCATGTGTCATGCTA
CCCTCACCCACAGGCTGATGTCTCCTCACAGGGTGCCGAACTACAACCTGTTCGTGATGGATG
AGGCTCATTTCACCGACCCAGCTAGCATTGCAGCAAGAGGTTACATTTCCACAAAGGTCGAG
CTAGGGGAGGCGGCGGCAATATTCATGACAGCCACCCCACCAGGCACTTCAGATCCATTCCCA
GAGTCCAATTCACCAATTTCCGACTTACAGACTGAGATCCCGGATCGAGCTTGGAACTCTGGA
TACGAATGGATCACAGAATACACCGGGAAGACGGTTTGGTTTGTGCCTAGTGTCAAGATGGG
GAATGAGATTGCCCTTTGCCTACAACGTGCTGGAAAGAAAGTAGTCCAATTGAACAGAAAGT
CGTACGAGACGGAGTACCCAAAATGTAAGAACGATGATTGGGACTTTGTTATCACAACAGAC
ATATCTGAAATGGGGGCTAACTTCAAGGCGAGCAGGGTGATTGACAGCCGGAAGAGTGTGAA
ACCAACCATCATAACAGAAGGAGAAGGGAGAGTGATCCTGGGAGAACCATCTGCAGTGACA
GCAGCTAGTGCCGCCCAGAGACGTGGACGTATCGGTAGAAATCCGTCGCAAGTTGGTGATGA
GTACTGTTATGGGGGGCACACGAATGAAGACGACTCGAACTTCGCCCATTGGACTGAGGCAC
GAATCATGCTGGACAACATCAACATGCCAAACGGACTGATCGCTCAATTCTACCAACCAGAGC
GTGAGAAGGTATATACCATGGATGGGGAATACCGGCTCAGAGGAGAAGAGAGAAAAAACTTT
CTGGAACTGTTGAGGACTGCAGATCTGCCAGTTTGGCTGGCTTACAAGGTTGCAGCGGCTGG
AGTGTCATACCACGACCGGAGGTGGTGCTTTGATGGTCCTAGGACAAACACAATTTTAGAAG
ACAACAACGAAGTGGAAGTCATCACGAAGCTTGGTGAAAGGAAGATTCTGAGGCCGCGCTG
GATTGATGCCAGGGTGTACTCGGATCACCAGGCACTAAAGGCGTTCAAGGACTTCGCCTCGG
GAAAACGTTCTCAGATAGGGCTCATTGAGGTTCTGGGAAAGATGCCTGAGCACTTCATGGGG
AAGACATGGGAAGCACTTGACACCATGTACGTTGTGGCCACTGCAGAGAAAGGAGGAAGAG
CTCACAGAATGGCCCTGGAGGAACTGCCAGATGCTCTTCAGACAATTGCCTTGATTGCCTTAT
TGAGTGTGATGACCATGGGAGTATTCTTCCTCCTCATGCAGCGGAAGGGCATTGGAAAGATAG
GTTTGGGAGGCGCTGTCTTGGGAGTCGCGACCTTTTTCTGTTGGATGGCTGAAGTTCCAGGA
ACGAAGATCGCCGGAATGTTGCTGCTCTCCCTTCTCTTGATGATTGTGCTAATTCCTGAGCCAG
AGAAGCAACGTTCGCAGACAGACAACCAGCTAGCCGTGTTCCTGATTTGTGTCATGACCCTT
GTGAGCGCAGTGGCAGCCAACGAGATGGGTTGGCTAGATAAGACCAAGAGTGACATAAGCA
GTTTGTTTGGGCAAAGAATTGAGGTCAAGGAGAATTTCAGCATGGGAGAGTTTCTTCTGGAC
TTGAGGCCGGCAACAGCCTGGTCACTGTACGCTGTGACAACAGCGGTCCTCACTCCACTGCT
AAAGCATTTGATCACGTCAGATTACATCAACACCTCATTGACCTCAATAAACGTTCAGGCAAG
TGCACTATTCACACTCGCGCGAGGCTTCCCCTTCGTCGATGTTGGAGTGTCGGCTCTCCTGCT
AGCAGCCGGATGCTGGGGACAAGTCACCCTCACCGTTACGGTAACAGCGGCAACACTCCTTT
TTTGCCACTATGCCTACATGGTTCCCGGTTGGCAAGCTGAGGCAATGCGCTCAGCCCAGCGGC
GGACAGCGGCCGGAATCATGAAGAACGCTGTAGTGGATGGCATCGTGGCCACGGACGTCCCA
GAATTAGAGCGCACCACACCCATCATGCAGAAGAAAGTTGGACAGATCATGCTGATCTTGGTG

TCTCTAGCTGCAGTAGTAGTGAACCCGTCTGTGAAGACAGTACGAGAAGCCGGAATTTTGATC
ACGGCCGCAGCGGTGACGCTTTGGGAGAATGGAGCAAGCTCTGTTTGGAACGCAACAACTG
CCATCGGACTCTGCCACATCATGCGTGGGGGTTGGTTGTCATGTCTATCCATAACATGGACACT
CATAAAGAACATGGAAAAACCAGGACTAAAAAGAGGTGGGGCAAAAGGACGCACCTTGGGA
GAGGTTTGGAAAGAAAGACTCAACCAGATGACAAAAGAAGAGTTCACT

>wt-NY99_**Fragment-III**
GGTGGGGCAAAAGGACGCACCTTGGGAGAGGTTTGGAAAGAAAGACTCAACCAGATGACAA
AAGAAGAGTTCACTAGGTACCGCAAAGAGGCCATCATCGAAGTCGATCGCTCAGCGGCAAAA
CACGCCAGGAAAGAAGGCAATGTCACTGGAGGGCATCCAGTCTCTAGGGGCACAGCAAAAC
TGAGATGGCTGGTCGAACGGAGGTTTCTCGAACCGGTCGGAAAAGTGATTGACCTTGGATGT
GGAAGAGGCGGTTGGTGTTACTATATGGCAACCCAAAAAAGAGTCCAAGAAGTCAGAGGGTA
CACAAAGGGCGGTCCCGGACATGAAGAGCCCCAACTAGTGCAAAGTTATGGATGGAACATTG
TCACCATGAAGAGTGGAGTGGATGTGTTCTACAGACCTTCTGAGTGTTGTGACACCCTCCTTT
GTGACATCGGAGAGTCCTCGTCAAGTGCTGAGGTTGAAGAGCATAGGACGATTCGGGTCCTT
GAAATGGTTGAGGACTGGCTGCACCGAGGGCCAAGGGAATTTTGCGTGAAGGTGCTCTGTCC
CTACATGCCGAAAGTCATAGAGAAGATGGAGCTGCTCCAACGCCGGTATGGGGGGGACTGG
TCAGAAACCCACTCTCACGGAATTCCACGCACGAGATGTATTGGGTGAGTCGAGCTTCAGGC
AATGTGGTACATTCAGTGAATATGACCAGCCAGGTGCTCCTAGGAAGAATGGAAAAAAGGAC
CTGGAAGGGACCCCAATACGAGGAAGATGTAAACTTGGGAAGTGGAACCAGGGCGGTGGGA
AAACCCCTGCTCAACTCAGACACCAGTAAAATCAAGAACAGGATTGAACGACTCAGGCGTGA
GTACAGTTCGACGTGGCACCACGATGAGAACCACCCATATAGAACCTGGAACTATCACGGCA
GTTATGATGTGAAGCCCACAGGCTCCGCCAGTTCGCTGGTCAATGGAGTGGTCAGGCTCCTCT
CAAAACCATGGGACACCATCACGAATGTTACCACCATGGCCATGACTGACACTACTCCCTTCG
GGCAGCAGCGAGTGTTCAAAGAGAAGGTGGACACGAAAGCTCCTGAACCGCCAGAAGGAG
TGAAGTACGTGCTCAACGAGACCACCAACTGGTTGTGGGCGTTTTTGGCCAGAGAAAAACGT
CCCAGAATGTGCTCTCGAGAGGAATTCATAAGAAAGGTCAACAGCAATGCAGCTTTGGGTGC
CATGTTTGAAGAGCAGAATCAATGGAGGAGCGCCAGAGAAGCAGTTGAAGATCCAAAATTTT
GGGAGATGGTGGATGAGGAGCGCGAGGCACATCTGCGGGGGGAATGTCACACTTGCATTTAC
AACATGATGGGAAAGAGAGAGAAAAAACCCGGAGAGTTCGGAAAGGCCAAGGGAAGCAGA
GCCATTTGGTTCATGTGGCTCGGAGCTCGCTTTCTGGAGTTCGAGGCTCTGGGTTTTCTCAAT
GAAGACCACTGGCTTGGAAGAAAGAACTCAGGAGGAGGTGTCGAGGGCTTGGGCCTCCAAA
AACTGGGTTACATCCTGCGTGAAGTTGGCACCCGGCCTGGGGGCAAGATCTATGCTGATGACA
CAGCTGGCTGGGACACCCGCATCACGAGAGCTGACTTGGAAAATGAAGCTAAGGTGCTTGAG
CTGCTTGATGGGGAACATCGGCGTCTTGCCAGGGCCATCATTGAGCTCACCTATCGTCACAAA
GTTGTGAAAGTGATGCGCCCGGCTGCTGATGGAAGAACCGTCATGGATGTTATCTCCAGAGAA
GATCAGAGGGGGAGTGGACAAGTTGTCACCTACGCCCTAAACACTTTCACCAACCTGGCCGT
CCAGCTGGTGAGGATGATGGAAGGGGAAGGAGTGATTGGCCCAGATGATGTGGAGAAACTC
ACAAAAGGGAAAGGACCCAAAGTCAGGACCTGGCTGTTTGAGAATGGGGAAGAAAGACTCA
GCCGCATGGCTGTCAGTGGAGATGACTGTGTGGTAAAGCCCCTGGACGATCGCTTTGCCACCT
CGCTCCACTTCCTCAATGCTATGTCAAAGGTTCGCAAAGACATCCAAGAGTGGAAACCGTCA
ACTGGATGGTATGATTGGCAGCAGGTTCCATTTTGCTCAAACCATTTCACTGAATTGATCATGA
AAGATGGAAGAACACTGGTGGTTCCATGCCGAGGACAGGATGAATTGGTAGGCAGAGCTCGC
ATATCTCCAGGGGCCGGATGGAACGTCCGCGACACTGCTTGTCTGGCTAAGTCTTATGCCCAG
ATGTGGCTGCTTCTGTACTTCCACAGAAGAGACCTGCGGCTCATGGCCAACGCCATTTGCTCC
GCTGTCCCTGTGAATTGGGTCCCTACCGGAAGAACCACGTGGTCCATCCATGCAGGAGGAGA
GTGGATGACAACAGAGGACATGTTGGAGGTCTGGAACCGTGTTTGGATAGAGGAGAATGAAT

File S2 ISA fragments

GGATGGAAGACAAAACCCCAGTGGAGAAATGGAGTGACGTCCCATATTCAGGAAAACGAGA
GGACATCTGGTGTGGCAGCCTGATTGGCACAAGAGCCCGAGCCACGTGGGCAGAAAACATCC
AGGTGGCTATCAACCAAGTCAGAGCAATCATCGGAGATGAGAAGTATGTGGACTACATGAGTT
CACTAAAGAGATATGAAGACACAACTTTGGTTGAGGACACAGTACTGTAGATATTTAATCAAT
TGTAAATAGACAATATAAGTATGCATAAAAGTGTAGTTTTATAGTAGTATTTAGTGGTGTTAGTG
TAAATAGTTAAGAAAATTTTGAGGAGAAAGTCAGGCCGGGAAGTTCCCGCCACCGGAAGTTG
AGTAGACGGTGCTGCCTGCGACTCAACCCCAGGAGGACTGGGTGAACAAAGCCGCGAAGTG
ATCCATGTAAGCCCTCAGAACCGTCTCGGAAGGAGGACCCCACATGTTGTAACTTCAAAGCC
CAATGTCAGACCACGCTACGGCGTGCTACTCTGCGGAGAGTGCAGTCTGCGATAGTGCCCCA
GGAGGACTGGGTTAACAAAGGCAAACCAACGCCCCACGCGGCCCTAGCCCCGGTAATGGTGT
TAACCAGGGCGAAAGGACTAGAGGTTAGAGGAGACCCCGCGGTTTAAAGTGCACGGCCCAG
CCTGGCTGAAGCTGTAGGTCAGGGGAAGGACTAGAGGTTAGTGGAGACCCCGTGCCACAAA
ACACCACAACAAAACAGCATATTGACACCTGGGATAGACTAGGAGATCTTCTGCTCTGCACAA
CCAGCCACACGGCACAGTGCGCCGACAATGGTGGCTGGTGGTGCGAGAACACAGGATCT**GG
CCGGCATGGTCCCAGCCTCCTCGCTGGCGCCGGCTGGGCAACATTCCGAGGGGACCGT
CCCCTCGGTAATGGCGAATGGGACTCGCGACAGACATGATAAGATACATTGATGAGTTT
GGACAAACCACAACTAGAATGCAGTGAAAAAAATGCTTTATTTGTGAAATTAAGCGCTG
GCATTGACCCTGAG**

File S2 ISA fragments

E+UA

Highlighted in red – pCMV promoter sequence.
Highlighted in green – HDR/SV40pA sequence.

In brown are 22 nt adapters applied during synthesis of DNA without using bacterial plasmids and bacteria.

>NY99-E+UA_**Fragment-IA**
*caatccgccctcactacaaccg*CACCCAACTGATCTTCAGCATCTTCAATATTGGCCATTAGCCATATTATT
CATTGGTTATATAGCATAAATCAATATTGGCTATTGGCCATTGCATACGTTGTATCTATATCATAAT
ATGTACATTTATATTGGCTCATGTCCAATATGACCGCCATGTTGGCATTGATTATTGACTAGTTAT
TAATAGTAATCAATTACGGGGTCATTAGTTCATAGCCCATATATGGAGTTCCGCGTTACATAACT
TACGGTAAATGGCCCGCCTGGCTGACCGCCCAACGACCCCCGCCCATTGACGTCAATAATGAC
GTATGTTCCCATAGTAACGCCAATAGGGACTTTCCATTGACGTCAATGGGTGGAGTATTTACGG
TAAACTGCCCACTTGGCAGTACATCAAGTGTATCATATGCCAAGTCCGCCCCCTATTGACGTCA
ATGACGGTAAATGGCCCGCCTGGCATTATGCCCAGTACATGACCTTACGGGACTTTCCTACTTG
GCAGTACATCTACGTATTAGTCATCGCTATTACCATGGTGATGCGGTTTTGGCAGTACACCAAT
GGGCGTGGATAGCGGTTTGACTCACGGGGATTTCCAAGTCTCCACCCCATTGACGTCAATGGG
AGTTTGTTTTGGCACCAAAATCAACGGGACTTTCCAAAATGTCGTAATAACCCCGCCCCGTTG
ACGCAAATGGGCGGTAGGCGTGTACGGTGGGAGGTCTATATAAGCAGAGCTCGTTTAGTGAA
CCGAGTAGTTCGCCTGTGTGAGCTGACAAACTTAGTAGTGTTTGTGAGGATTAACAACAATTA
ACACAGTGCGAGCTGTTTCTTAGCACGAAGATCTCGATGTCTAAGAAACCAGGAGGGCCCGG
CAAGAGCCGGGCTGTCAATATGCTAAAACGCGGAATGCCCCGCGTGTTGTCCTTGATTGGACT
GAAGAGGGCTATGTTGAGCCTGATCGACGGCAAGGGGCCAATACGATTTGTGTTGGCTCTCTT
GGCGTTCTTCAGGTTCACAGCAATTGCTCCGACCCGAGCAGTGCTGGATCGATGGAGAGGTG
TGAACAAACAAACAGCGATGAAACACCTTCTGAGTTTTAAGAAGGAACTAGGGACCTTGACC
AGTGCTATCAATCGGCGGAGCTCAAAACAAAAGAAAAGAGGAGGAAAGACCGGAATTGCAG
TCATGATTGGCCTGATCGCCAGCGTAGGAGCAGTTACCCTCTCTAACTTCCAAGGGAAGGTGA
TGATGACGGTAAATGCTACTGACGTCACAGATGTCATCACGATTCCAACAGCTGCTGGAAAGA
ACCTATGCATTGTCAGAGCAATGGATGTGGGATACATGTGCGATGATACTATCACTTATGAATG
CCCAGTACTGTCGGCTGGTAATGATCCAGAAGACATCGACTGTTGGTGCACAAAGTCAGCAG
TCTACGTCAGGTATGGAAGATGCACCAAGACACGCCACTCAAGACGCAGTCGGAGGTCACTG
ACAGTGCAGACACACGGA*ctactctggcgtcgatgaggga*

>NY99-E+UA_**Fragment-IB**
*caatccgccctcactacaaccg*AGGTATGGAAGATGCACCAAGACACGCCACTCAAGACGCAGTCGGAG
GTCACTGACAGTGCAGACACACGGAGAAAGCACTCTAGCGAACAAGAAGGGGGCTTGGATG
GACAGCACCAAGGCCACAAGGTATTTGGTAAAAACAGAATCATGGATCTTGAGGAACCCTGG
ATATGCCCTGGTGGCAGCCGTCATTGGTTGGATGCTTGGGAGCAACACCATGCAGAGAGTTGT
GTTTGTCGTGCTATTGCTTTTGGTGGCCCCAGCTTACAGCTTCAACTGCCTTGGAATGAGCAA
CAGAGACTTCTTGGAGGGAGTATCTGGAGCAACGTGGGTAGACTTAGTGCTAGAAGGCGACA
GCTGTGTGACTATCATGTCTAAGGACAAGCCTACCATCGATGTTAAGATGATGAACATGGAGG
CGGCCAACCTGGCAGAGGTCCGTAGCTATTGCTACTTGGCGACCGTCAGTGATCTCTCCACCA
AAGCCGCGTGCCCGACTATGGGAGAAGCGCACAATGATAAACGTGCGGACCCAGCTTTCGTA
TGCAGACAGGGAGTAGTAGACAGGGGCTGGGGCAACGGCTGCGGGCTATTCGGCAAAGGAA
GCATAGATACGTGTGCCAAGTTTGCCTGCTCTACCAAGGCAATAGGAAGAACCATATTGAAAG

File S2 ISA fragments

AGAACATCAAGTATGAGGTGGCTATTTTGTACATGGGCCTACTACTGTAGAGTCTCACGGCA
ACTACTCCACCCAGGTAGGAGCTACTCAGGCAGGTAGACTCAGTATCACTCCTGCGGCTCCCT
CATATACCCTAAAGCTTGGGGAATATGGAGAGGTTACGGTGGACTGTGAGCCCCGGTCAGGGA
TAGACACTAATGCATACTACGTGATGACTGTTGGTACAAAGACGTTCTTGGTCCATAGGGAGT
GGTTCATGGACCTCAACCTACCCTGGAGCAGTGCGGGAAGTACTGTGTGGAGGAATAGAGAG
ACTTTAATGGAGTTTGAGGAGCCACACGCCACTAAGCAGTCTGTTATAGCATTAGGCTCACAA
GAGGGCGCCCTGCATCAAGCTTTAGCGGGGGCCATTCCTGTGGAATTCTCCAGCAACACTGTC
AAGTTGACGTCCGGTCATTTGAAGTGTAGAGTAAAGATGGAAAAATTGCAGTTGAAGGGCAC
CACCTATGGAGTCTGTTCAAAGGCTTTTAAGTTTCTTGGGACTCCCGCGGACACAGGTCACGG
CACTGTGGTGTTAGAATTACAGTACACTGGCACGGATGGACCTTGCAAAGTTCCTATCTCTTC
GGTGGCTTCATTGAATGACCTAACACCGGTAGGTAGATTGGTAACTGTAAACCCCTTTGTCTCC
GTGGCCACGGCCAACGCTAAGGTACTCATTGAATTGGAGCCACCCTTCGGGGACTCCTACATA
GTAGTAGGCAGAGGGGAACAACAGATCA***ctactctggcgtcgatgaggga***

>NY99-E+UA_**Fragment-IC**
caatccgccctcactacaaccgGGTACTCATTGAATTGGAGCCACCCTTCGGGGACTCCTACATAGTAGTA
GGCAGAGGGGAACAACAGATCAATCACCATTGGCACAAGTCGGGTAGCAGCATAGGCAAAG
CCTTTACCACCACCCTAAAAGGAGCGCAGAGACTAGCAGCTCTAGGAGACACAGCTTGGGAC
TTTGGGTCGGTTGGAGGGGTATTTACGTCAGTAGGGAAGGCTGTCCACCAAGTATTTGGAGGA
GCCTTCAGATCACTGTTCGGAGGCATGTCCTGGATAACCCAAGGCTTGCTGGGGGCTCTCCTC
TTGTGGATGGGCATAAATGCTCGGGATAGGTCCATAGCCCTCACTTTCCTGGCAGTTGGAGGA
GTTCTGCTCTTCCTCTCCGTGAACGTGCACGCTGACACTGGGTGTGCCATAGACATCAGCCGG
CAAGAGCTGAGATGTGGAAGTGGAGTGTTCATACACAATGATGTGGAGGCTTGGATGGACCG
GTACAAGTATTACCCTGAAACGCCACAAGGCCTAGCCAAGATCATTCAGAAAGCTCATAAGGA
AGGAGTGTGCGGTCTACGATCAGTTTCCAGACTGGAGCATCAAATGTGGGAAGCAGTGAAGG
ACGAGCTGAACACTCTTTTGAAGGAGAATGGTGTGGACCTTAGTGTCGTGGTTGAGAAACAG
GAGGGAATGTACAAGTCAGCACCTAAACGCCTCACCGCCACCACGGAAAAATTGGAAATTGG
CTGGAAGGCCTGGGGAAAGAGTATTTTATTTGCACCAGAACTCGCCAACAACACCTTTGTGG
TTGATGGTCCGGAGACCAAGGAATGTCCGACTCAGAATCGCGCTTGGAATAGCTTAGAAGTG
GAGGATTTTGGATTTGGTCTCACCAGCACTCGGATGTTCCTGAAGGTCAGAGAGAGCAACAC
AACTGAATGTGACTCGAAGATCATTGGAACGGCTGTCAAGAACAACTTGGCGATCCACAGTG
ACCTGTCCTATTGGATTGAAAGCAGGCTCAATGATACGTGGAAGCTTGAAAGGGCAGTTCTGG
GTGAAGTCAAATCATGTACGTGGCCTGAGACGCATACCTTGTGGGGCGATGGAATCCTTGAGA
GTGACTTGATAATACCAGTCACACTGGCGGGACCACGAAGCAATCACAATCGGAGACCTGGG
TACAAGACACAAAACCAGGGCCCATGGGACGAAGGCCGGGTAGAGATTGACTTCGATTACTG
CCCAGGAACTACGGTCACCCTGAGTGAGAGCTGCGGACACCGTGGACCTGCCACTCGCACCA
CCACAGAGAGCGGAAAGTTGATAACAGATTGGTGCTGCAGGAGCTGCACCTTACCACCACTG
CGCTACCAAACTGACAGCGGCTGTTGGTATGGTATGGAGATCAGACCACAGAGACATGATGA
AAAGACCCTCGTGCAGTCACAAGTGAATGCTTATAATGCTGATATGATTGACCCTTTTCAGTTG
GGCCTTCTGGTCGTGTTCTTGGCCACCCAGGAGGTCCTTCGC***ctactctggcgtcgatgaggga***

>wt-NY99_**Fragment-II**
TATAATGCTGATATGATTGACCCTTTTCAGTTGGGCCTTCTGGTCGTGTTCTTGGCCACCCAGG
AGGTCCTTCGCAAGAGGTGGACAGCCAAGATCAGCATGCCAGCTATACTGATTGCTCTGCTAG
TCCTGGTGTTTGGGGGCATTACTTACACTGATGTGTTACGCTATGTCATCTTGGTGGGGGCAGC
TTTCGCAGAATCTAATTCGGGAGGAGACGTGGTACACTTGGCGCTCATGGCGACCTTCAAGAT
ACAACCAGTGTTTATGGTGGCATCGTTTCTCAAAGCGAGATGGACCAACCAGGAGAACATTTT

File S2 ISA fragments

GTTGATGTTGGCGGCTGTTTTCTTTCAAATGGCTTATCACGATGCCCGCCAAATTCTGCTCTGG
GAGATCCCTGATGTGTTGAATTCACTGGCGGTAGCTTGGATGATACTGAGAGCCATAACATTCA
CAACGACATCAAACGTGGTTGTTCCGCTGCTAGCCCTGCTAACACCCGGGCTGAGATGCTTGA
ATCTGGATGTGTACAGGATACTGCTGTTGATGGTCGGAATAGGCAGCTTGATCAGGGAGAAGA
GGAGTGCAGCCGCAAAAAAGAAAGGAGCAAGTCTGCTATGCTTGGCTCTAGCCTCAACAGG
ACTTTTCAACCCCATGATCCTTGCTGCTGGACTGATTGCATGTGATCCCAACCGTAAACGCGG
ATGGCCCGCAACTGAAGTGATGACAGCTGTCGGCCTAATGTTTGCCATCGTCGGAGGGCTGGC
AGAGCTTGACATTGACTCCATGGCCATTCCAATGACTATCGCGGGGCTCATGTTTGCTGCTTTC
GTGATTTCTGGGAAATCAACAGATATGTGGATTGAGAGAACGGCGGACATTTCCTGGGAAAGT
GATGCAGAAATTACAGGCTCGAGCGAAAGAGTTGATGTGCGGCTTGATGATGATGGAAACTT
CCAGCTCATGAATGATCCAGGAGCACCTTGGAAGATATGGATGCTCAGAATGGTCTGTCTCGC
GATTAGTGCGTACACCCCCTGGGCAATCTTGCCCTCAGTAGTTGGATTTTGGATAACTCTCCAA
TACACAAAGAGAGGAGGCGTGTTGTGGGACACTCCCTCACCAAAGGAGTACAAAAAGGGGG
ACACGACCACCGGCGTCTACAGGATCATGACTCGTGGGCTGCTCGGCAGTTATCAAGCAGGA
GCGGGCGTGATGGTTGAAGGTGTTTTCCACACCCTTTGGCATACAACAAAAGGAGCCGCTTT
GATGAGCGGAGAGGGCCGCCTGGACCCATACTGGGGCAGTGTCAAGGAGGATCGACTTTGTT
ACGGAGGACCCTGGAAATTGCAGCACAAGTGGAACGGGCAGGATGAGGTGCAGATGATTGT
GGTGGAACCTGGCAAGAACGTTAAGAACGTCCAGACGAAACCAGGGGTGTTCAAAACACCT
GAAGGAGAAATCGGGGCCGTGACTTTGGACTTCCCCACTGGAACATCAGGCTCACCAATAGT
GGACAAAAACGGTGATGTGATTGGGCTTTATGGCAATGGAGTCATAATGCCCAACGGCTCATA
CATAAGCGCGATAGTGCAGGGTGAAAGGATGGATGAGCCAATCCCAGCCGGATTCGAACCTG
AGATGCTGAGGAAAAAACAGATCACTGTACTGGATCTCCATCCCGGCGCCGGTAAAACAAGG
AGGATTCTGCCACAGATCATCAAAGAGGCCATAAACAGAAGACTGAGAACAGCCGTGCTAGC
GCCAACCAGGGTTGTGGCTGCTGAGATGGCTGAAGCACTGAGAGGACTGCCCATCCGGTACC
AGACATCCGCAGTGCCCAGAGAACATAATGGAAATGAGATTGTTGATGTCATGTGTCATGCTA
CCCTCACCCACAGGCTGATGTCTCCTCACAGGGTGCCGAACTACAACCTGTTCGTGATGGATG
AGGCTCATTTCACCGACCCAGCTAGCATTGCAGCAAGAGGTTACATTTCCACAAAGGTCGAG
CTAGGGGAGGCGGCGGCAATATTCATGACAGCCACCCCACCAGGCACTTCAGATCCATTCCCA
GAGTCCAATTCACCAATTTCCGACTTACAGACTGAGATCCCGGATCGAGCTTGGAACTCTGGA
TACGAATGGATCACAGAATACACCGGGAAGACGGTTTGGTTTGTGCCTAGTGTCAAGATGGG
GAATGAGATTGCCCTTTGCCTACAACGTGCTGGAAAGAAAGTAGTCCAATTGAACAGAAAGT
CGTACGAGACGGAGTACCCAAAATGTAAGAACGATGATTGGGACTTTGTTATCACAACAGAC
ATATCTGAAATGGGGGCTAACTTCAAGGCGAGCAGGGTGATTGACAGCCGGAAGAGTGTGAA
ACCAACCATCATAACAGAAGGAGAAGGGAGAGTGATCCTGGGAGAACCATCTGCAGTGACA
GCAGCTAGTGCCGCCCAGAGACGTGGACGTATCGGTAGAAATCCGTCGCAAGTTGGTGATGA
GTACTGTTATGGGGGGCACACGAATGAAGACGACTCGAACTTCGCCCATTGGACTGAGGCAC
GAATCATGCTGGACAACATCAACATGCCAAACGGACTGATCGCTCAATTCTACCAACCAGAGC
GTGAGAAGGTATATACCATGGATGGGGAATACCGGCTCAGAGGAGAAGAGAGAAAAAACTTT
CTGGAACTGTTGAGGACTGCAGATCTGCCAGTTTGGCTGGCTTACAAGGTTGCAGCGGCTGG
AGTGTCATACCACGACCGGAGGTGGTGCTTTGATGGTCCTAGGACAAACACAATTTTAGAAG
ACAACAACGAAGTGGAAGTCATCACGAAGCTTGGTGAAAGGAAGATTCTGAGGCCGCGCTG
GATTGATGCCAGGGTGTACTCGGATCACCAGGCACTAAAGGCGTTCAAGGACTTCGCCTCGG
GAAAACGTTCTCAGATAGGGCTCATTGAGGTTCTGGGAAAGATGCCTGAGCACTTCATGGGG
AAGACATGGGAAGCACTTGACACCATGTACGTTGTGGCCACTGCAGAGAAAGGAGGAAGAG
CTCACAGAATGGCCCTGGAGGAACTGCCAGATGCTCTTCAGACAATTGCCTTGATTGCCTTAT
TGAGTGTGATGACCATGGGAGTATTCTTCCTCCTCATGCAGCGGAAGGGCATTGGAAAGATAG
GTTTGGGAGGCGCTGTCTTGGGAGTCGCGACCTTTTTCTGTTGGATGGCTGAAGTTCCAGGA

ACGAAGATCGCCGGAATGTTGCTGCTCTCCCTTCTCTTGATGATTGTGCTAATTCCTGAGCCAG
AGAAGCAACGTTCGCAGACAGACAACCAGCTAGCCGTGTTCCTGATTTGTGTCATGACCCTT
GTGAGCGCAGTGGCAGCCAACGAGATGGGTTGGCTAGATAAGACCAAGAGTGACATAAGCA
GTTTGTTTGGGCAAAGAATTGAGGTCAAGGAGAATTTCAGCATGGGAGAGTTTCTTCTGGAC
TTGAGGCCGGCAACAGCCTGGTCACTGTACGCTGTGACAACAGCGGTCCTCACTCCACTGCT
AAAGCATTTGATCACGTCAGATTACATCAACACCTCATTGACCTCAATAAACGTTCAGGCAAG
TGCACTATTCACACTCGCGCGAGGCTTCCCCTTCGTCGATGTTGGAGTGTCGGCTCTCCTGCT
AGCAGCCGGATGCTGGGGACAAGTCACCCTCACCGTTACGGTAACAGCGGCAACACTCCTTT
TTTGCCACTATGCCTACATGGTTCCCGGTTGGCAAGCTGAGGCAATGCGCTCAGCCCAGCGGC
GGACAGCGGCCGGAATCATGAAGAACGCTGTAGTGGATGGCATCGTGGCCACGGACGTCCCA
GAATTAGAGCGCACCACACCCATCATGCAGAAGAAAGTTGGACAGATCATGCTGATCTTGGTG
TCTCTAGCTGCAGTAGTAGTGAACCCGTCTGTGAAGACAGTACGAGAAGCCGGAATTTTGATC
ACGGCCGCAGCGGTGACGCTTTGGGAGAATGGAGCAAGCTCTGTTTGGAACGCAACAACTG
CCATCGGACTCTGCCACATCATGCGTGGGGGTTGGTTGTCATGTCTATCCATAACATGGACACT
CATAAAGAACATGGAAAAACCAGGACTAAAAAGAGGTGGGGCAAAAGGACGCACCTTGGGA
GAGGTTTGGAAAGAAAGACTCAACCAGATGACAAAAGAAGAGTTCACT

>wt-NY99_**Fragment-III**
GGTGGGGCAAAAGGACGCACCTTGGGAGAGGTTTGGAAAGAAAGACTCAACCAGATGACAA
AAGAAGAGTTCACTAGGTACCGCAAAGAGGCCATCATCGAAGTCGATCGCTCAGCGGCAAAA
CACGCCAGGAAAGAAGGCAATGTCACTGGAGGGCATCCAGTCTCTAGGGGCACAGCAAAAC
TGAGATGGCTGGTCGAACGGAGGTTTCTCGAACCGGTCGGAAAAGTGATTGACCTTGGATGT
GGAAGAGGCGGTTGGTGTTACTATATGGCAACCCAAAAAAGAGTCCAAGAAGTCAGAGGGTA
CACAAAGGGCGGTCCCGGACATGAAGAGCCCCAACTAGTGCAAAGTTATGGATGGAACATTG
TCACCATGAAGAGTGGAGTGGATGTGTTCTACAGACCTTCTGAGTGTTGTGACACCCTCCTTT
GTGACATCGGAGAGTCCTCGTCAAGTGCTGAGGTTGAAGAGCATAGGACGATTCGGGTCCTT
GAAATGGTTGAGGACTGGCTGCACCGAGGGCCAAGGGAATTTTGCGTGAAGGTGCTCTGTCC
CTACATGCCGAAAGTCATAGAGAAGATGGAGCTGCTCCAACGCCGGTATGGGGGGGGACTGG
TCAGAAACCCACTCTCACGGAATTCCACGCACGAGATGTATTGGGTGAGTCGAGCTTCAGGC
AATGTGGTACATTCAGTGAATATGACCAGCCAGGTGCTCCTAGGAAGAATGGAAAAAAGGAC
CTGGAAGGGACCCCAATACGAGGAAGATGTAAACTTGGGAAGTGGAACCAGGGCGGTGGGA
AAACCCCTGCTCAACTCAGACACCAGTAAAATCAAGAACAGGATTGAACGACTCAGGCGTGA
GTACAGTTCGACGTGGCACCACGATGAGAACCACCCATATAGAACCTGGAACTATCACGGCA
GTTATGATGTGAAGCCCACAGGCTCCGCCAGTTCGCTGGTCAATGGAGTGGTCAGGCTCCTCT
CAAAACCATGGGACACCATCACGAATGTTACCACCATGGCCATGACTGACACTACTCCCTTCG
GGCAGCAGCGAGTGTTCAAAGAGAAGGTGGACACGAAAGCTCCTGAACCGCCAGAAGGAG
TGAAGTACGTGCTCAACGAGACCACCAACTGGTTGTGGGCGTTTTTGGCCAGAGAAAAACGT
CCCAGAATGTGCTCTCGAGAGGAATTCATAAGAAAGGTCAACAGCAATGCAGCTTTGGGTGC
CATGTTTGAAGAGCAGAATCAATGGAGGAGCGCCAGAGAAGCAGTTGAAGATCCAAAATTTT
GGGAGATGGTGGATGAGGAGCGCGAGGCACATCTGCGGGGGGAATGTCACACTTGCATTTAC
AACATGATGGGAAAGAGAGAGAAAAAACCCGGAGAGTTCGGAAAGGCCAAGGGAAGCAGA
GCCATTTGGTTCATGTGGCTCGGAGCTCGCTTTCTGGAGTTCGAGGCTCTGGGTTTTCTCAAT
GAAGACCACTGGCTTGGAAGAAAGAACTCAGGAGGAGGTGTCGAGGGCTTGGGCCTCCAAA
AACTGGGTTACATCCTGCGTGAAGTTGGCACCCGGCCTGGGGGCAAGATCTATGCTGATGACA
CAGCTGGCTGGGACACCCGCATCACGAGAGCTGACTTGGAAAATGAAGCTAAGGTGCTTGAG
CTGCTTGATGGGGAACATCGGCGTCTTGCCAGGGCCATCATTGAGCTCACCTATCGTCACAAA
GTTGTGAAAGTGATGCGCCCGGCTGCTGATGGAAGAACCGTCATGGATGTTATCTCCAGAGAA

File S2 ISA fragments

GATCAGAGGGGGAGTGGACAAGTTGTCACCTACGCCCTAAACACTTTCACCAACCTGGCCGT
CCAGCTGGTGAGGATGATGGAAGGGGAAGGAGTGATTGGCCCAGATGATGTGGAGAAACTC
ACAAAAGGGAAAGGACCCAAAGTCAGGACCTGGCTGTTTGAGAATGGGGAAGAAAGACTCA
GCCGCATGGCTGTCAGTGGAGATGACTGTGTGGTAAAGCCCCTGGACGATCGCTTTGCCACCT
CGCTCCACTTCCTCAATGCTATGTCAAAGGTTCGCAAAGACATCCAAGAGTGGAAACCGTCA
ACTGGATGGTATGATTGGCAGCAGGTTCCATTTTGCTCAAACCATTTCACTGAATTGATCATGA
AAGATGGAAGAACACTGGTGGTTCCATGCCGAGGACAGGATGAATTGGTAGGCAGAGCTCGC
ATATCTCCAGGGGCCGGATGGAACGTCCGCGACACTGCTTGTCTGGCTAAGTCTTATGCCCAG
ATGTGGCTGCTTCTGTACTTCCACAGAAGAGACCTGCGGCTCATGGCCAACGCCATTTGCTCC
GCTGTCCCTGTGAATTGGGTCCCTACCGGAAGAACCACGTGGTCCATCCATGCAGGAGGAGA
GTGGATGACAACAGAGGACATGTTGGAGGTCTGGAACCGTGTTTGGATAGAGGAGAATGAAT
GGATGGAAGACAAAACCCCAGTGGAGAAATGGAGTGACGTCCCATATTCAGGAAAACGAGA
GGACATCTGGTGTGGCAGCCTGATTGGCACAAGAGCCCGAGCCACGTGGGCAGAAAACATCC
AGGTGGCTATCAACCAAGTCAGAGCAATCATCGGAGATGAGAAGTATGTGGACTACATGAGTT
CACTAAAGAGATATGAAGACACAACTTTGGTTGAGGACACAGTACTGTAGATATTTAATCAAT
TGTAAATAGACAATATAAGTATGCATAAAAGTGTAGTTTTATAGTAGTATTTAGTGGTGTTAGTG
TAAATAGTTAAGAAAATTTTGAGGAGAAAGTCAGGCCGGGAAGTTCCCGCCACCGGAAGTTG
AGTAGACGGTGCTGCCTGCGACTCAACCCCAGGAGGACTGGGTGAACAAAGCCGCGAAGTG
ATCCATGTAAGCCCTCAGAACCGTCTCGGAAGGAGGACCCCACATGTTGTAACTTCAAAGCC
CAATGTCAGACCACGCTACGGCGTGCTACTCTGCGGAGAGTGCAGTCTGCGATAGTGCCCCA
GGAGGACTGGGTTAACAAAGGCAAACCAACGCCCCACGCGGCCCTAGCCCCGGTAATGGTGT
TAACCAGGGCGAAAGGACTAGAGGTTAGAGGAGACCCCGCGGTTTAAAGTGCACGGCCCAG
CCTGGCTGAAGCTGTAGGTCAGGGGAAGGACTAGAGGTTAGTGGAGACCCCGTGCCACAAA
ACACCACAACAAAACAGCATATTGACACCTGGGATAGACTAGGAGATCTTCTGCTCTGCACAA
CCAGCCACACGGCACAGTGCGCCGACAATGGTGGCTGGTGGTGCGAGAACACAGGATCT**GG
CCGGCATGGTCCCAGCCTCCTCGCTGGCGCCGGCTGGGCAACATTCCGAGGGGACCGT
CCCCTCGGTAATGGCGAATGGGACTCGCGACAGACATGATAAGATACATTGATGAGTTT
GGACAAACCACAACTAGAATGCAGTGAAAAAAATGCTTTATTTGTGAAATTAAGCGCTG
GCATTGACCCTGAG**

File S2 ISA fragments

E-MAX/NS5+CG

Highlighted in red – pCMV promoter sequence.
Highlighted in green – HDR/SV40pA sequence.

>NY99-E_CpG+UpA_**Fragment-I**
CACCCAACTGATCTTCAGCATCTTCAATATTGGCCATTAGCCATATTATTCATTGGTTATATAGCA
TAAATCAATATTGGCTATTGGCCATTGCATACGTTGTATCTATATCATAATATGTACATTTATATTG
GCTCATGTCCAATATGACCGCCATGTTGGCATTGATTATTGACTAGTTATTAATAGTAATCAATTA
CGGGGTCATTAGTTCATAGCCCATATATGGAGTTCCGCGTTACATAACTTACGGTAAATGGCCC
GCCTGGCTGACCGCCCAACGACCCCCGCCCATTGACGTCAATAATGACGTATGTTCCCATAGT
AACGCCAATAGGGACTTTCCATTGACGTCAATGGGTGGAGTATTTACGGTAAACTGCCCACTT
GGCAGTACATCAAGTGTATCATATGCCAAGTCCGCCCCCTATTGACGTCAATGACGGTAAATGG
CCCGCCTGGCATTATGCCCAGTACATGACCTTACGGGACTTTCCTACTTGGCAGTACATCTACG
TATTAGTCATCGCTATTACCATGGTGATGCGGTTTTGGCAGTACACCAATGGGCGTGGATAGCG
GTTTGACTCACGGGGATTTCCAAGTCTCCACCCCATTGACGTCAATGGGAGTTTGTTTTGGCA
CCAAAATCAACGGGACTTTCCAAAATGTCGTAATAACCCCGCCCCGTTGACGCAAATGGGCG
GTAGGCGTGTACGGTGGGAGGTCTATATAAGCAGAGCTCGTTTAGTGAACCGAGTAGTTCGCC
TGTGTGAGCTGACAAACTTAGTAGTGTTTGTGAGGATTAACAACAATTAACACAGTGCGAGCT
GTTTCTTAGCACGAAGATCTCGATGTCTAAGAAACCAGGAGGGCCCGGCAAGAGCCGGGCTG
TCAATATGCTAAAACGCGGAATGCCCCGCGTGTTGTCCTTGATTGGACTGAAGAGGGCTATGT
TGAGCCTGATCGACGGCAAGGGGCCAATACGATTTGTGTTGGCTCTCTTGGCGTTCTTCAGGT
TCACAGCAATTGCTCCGACCCGAGCAGTGCTGGATCGATGGAGAGGTGTGAACAAACAAACA
GCGATGAAACACCTTCTGAGTTTTAAGAAGGAACTAGGGACCTTGACCAGTGCTATCAATCGG
CGGAGCTCAAAACAAAAGAAAAGAGGAGGAAAGACCGGAATTGCAGTCATGATTGGCCTGA
TCGCCAGCGTAGGAGCAGTTACCCTCTCTAACTTCCAAGGGAAGGTGATGATGACGGTAAATG
CTACTGACGTCACAGATGTCATCACGATTCCAACAGCTGCTGGAAAGAACCTATGCATTGTCA
GAGCAATGGATGTGGGATACATGTGCGATGATACTATCACTTATGAATGCCCAGTACTGTCGGC
TGGTAATGATCCAGAAGACATCGACTGTTGGTGCACAAAGTCAGCAGTCTACGTCAGGTATGG
AAGATGCACCAAGACACGCCACTCAAGACGCAGTCGGAGGTCACTGACAGTGCAGACACAC
GGAGAAAGCACTCTAGCGAACAAGAAGGGGGCTTGGATGGACAGCACCAAGGCCACAAGGT
ATTTGGTAAAAACAGAATCATGGATCTTGAGGAACCCTGGATATGCCCTGGTGGCAGCCGTCA
TTGGTTGGATGCTTGGGAGCAACACCATGCAGAGAGTTGTGTTTGTCGTGCTATTGCTTTTGG
TGGCCCCAGCTTACAGCTTCAACTGCCTTGGAATGAGCAACAGAGACTTTTTGGAAGGCGTT
TCCGGCGCGACGTGGGTCGATTTAGTTCTCGAAGGGGATAGTTGCGTTACGATAATGTCGAAA
GACAAACCGACGATCGACGTTAAAATGATGAATATGGAGGCCGCGAATCTAGCGGAAGTTCG
CAGTTATTGTTATTTAGCAACCGTAAGCGATCTTTCGACGAAAGCCGCGTGTCCGACGATGGG
CGAAGCGCACAACGATAAACGGGCGGATCCCGCGTTTGTTTGTCGACAAGGCGTCGTCGATC
GCGGTTGGGGAAATGGTTGTGGACTATTCGGCAAAGGAAGTATCGATACGTGCGCGAAATTTG
CGTGTTCTACTAAAGCGATAGGACGAACGATATTAAAAGAAAATATCAAATACGAAGTCGCGA
TTTTTGTCCACGGACCGACGACGGTCGAATCGCACGGAAATTATTCGACGCAAGTCGGCGCG
ACGCAGGCCGGTCGACTAAGTATAACGCCCGCGGCGCCGTCGTACACGCTAAAACTGGGCGA
ATATGGCGAGGTTACGGTCGACTGCGAACCGCGTTCGGGTATCGATACGAACGCGTATTATGTT
ATGACGGTCGGAACGAAAACGTTTTTAGTACATCGGGAATGGTTCATGGATCTAAACCTACCG
TGGAGCAGTGCCGGAAGTACCGTTTGGCGTAACCGGGAAACGTTAATGGAATTCGAAGAACC
GCACGCGACTAAACAGTCCGTAATCGCGTTAGGATCGCAAGAAGGCGCGCTTCATCAAGCGT
TAGCCGGCGCGATTCCGGTCGAATTTTCGAGTAATACCGTCAAATTAACATCCGGTCATTTGAA

File S2 ISA fragments

ATGTCGGGTCAAAATGGAAAAATTACAATTAAAAGGAACGACTTACGGCGTATGTTCGAAAG
CGTTCAAGTTTCTCGGTACACCCGCCGATACCGGTCACGGAACCGTCGTTTTGGAATTACAAT
ATACCGGAACCGATGGACCGTGTAAAGTTCCGATATCGTCCGTGGCGTCGTTGAACGATCTAA
CACCCGTCGGCCGATTAGTCACCGTTAATCCGTTCGTTTCCGTCGCGACGGCGAACGCGAAAG
TACTTATAGAATTGGAACCGCCGTTTGGCGATTCGTATATCGTCGTCGGCCGCGGGGAACAAC
AAATAAATCACCATTGGCATAAGTCCGGAAGTAGTATCGGAAAAGCGTTTACGACGACGCTAA
AAGGCGCGCAACGACTAGCCGCGCTCGGCGATACCGCGTGGGATTTCGGATCCGTCGGGGGC
GTTTTTACGTCCGTCGGTAAAGCCGTACATCAAGTATTCGGCGGGGCGTTTCGCTCGCTTTTCG
GCGGAATGTCGTGGATAACGCAAGGATTACTGGGCGCGCTACTTTTATGGATGGGAATAAACG
CGCGGGATCGTTCGATCGCGCTAACGTTTCTCGCCGTCGGCGGAGTTCTGCTCTTCCTCTCCG
TGAACGTGCACGCTGACACTGGGTGTGCCATAGACATCAGCCGGCAAGAGCTGAGATGTGGA
AGTGGAGTGTTCATACACAATGATGTGGAGGCTTGGATGGACCGGTACAAGTATTACCCTGAA
ACGCCACAAGGCCTAGCCAAGATCATTCAGAAAGCTCATAAGGAAGGAGTGTGCGGTCTACG
ATCAGTTTCCAGACTGGAGCATCAAATGTGGGAAGCAGTGAAGGACGAGCTGAACACTCTTT
TGAAGGAGAATGGTGTGGACCTTAGTGTCGTGGTTGAGAAACAGGAGGGAATGTACAAGTCA
GCACCTAAACGCCTCACCGCCACCACGGAAAAATTGGAAATTGGCTGGAAGGCCTGGGGAA
AGAGTATTTTATTTGCACCAGAACTCGCCAACAACACCTTTGTGGTTGATGGTCCGGAGACCA
AGGAATGTCCGACTCAGAATCGCGCTTGGAATAGCTTAGAAGTGGAGGATTTTGGATTTGGTC
TCACCAGCACTCGGATGTTCCTGAAGGTCAGAGAGAGCAACACAACTGAATGTGACTCGAAG
ATCATTGGAACGGCTGTCAAGAACAACTTGGCGATCCACAGTGACCTGTCCTATTGGATTGAA
AGCAGGCTCAATGATACGTGGAAGCTTGAAAGGGCAGTTCTGGGTGAAGTCAAATCATGTAC
GTGGCCTGAGACGCATACCTTGTGGGGCGATGGAATCCTTGAGAGTGACTTGATAATACCAGT
CACACTGGCGGGACCACGAAGCAATCACAATCGGAGACCTGGGTACAAGACACAAAACCAG
GGCCCATGGGACGAAGGCCGGGTAGAGATTGACTTCGATTACTGCCCAGGAACTACGGTCAC
CCTGAGTGAGAGCTGCGGACACCGTGGACCTGCCACTCGCACCACCACAGAGAGCGGAAAG
TTGATAACAGATTGGTGCTGCAGGAGCTGCACCTTACCACCACTGCGCTACCAAACTGACAGC
GGCTGTTGGTATGGTATGGAGATCAGACCACAGAGACATGATGAAAAGACCCTCGTGCAGTC
ACAAGTGAATGCTTATAATGCTGATATGATTGACCCTTTTCAGTTGGGCCTTCTGGTCGTGTTC
TTGGCCACCCAGGAGGTCCTTCGC

>wt-NY99_**Fragment-II**
TATAATGCTGATATGATTGACCCTTTTCAGTTGGGCCTTCTGGTCGTGTTCTTGGCCACCCAGG
AGGTCCTTCGCAAGAGGTGGACAGCCAAGATCAGCATGCCAGCTATACTGATTGCTCTGCTAG
TCCTGGTGTTTGGGGGCATTACTTACACTGATGTGTTACGCTATGTCATCTTGGTGGGGGCAGC
TTTCGCAGAATCTAATTCGGGAGGAGACGTGGTACACTTGGCGCTCATGGCGACCTTCAAGAT
ACAACCAGTGTTTATGGTGGCATCGTTTCTCAAAGCGAGATGGACCAACCAGGAGAACATTTT
GTTGATGTTGGCGGCTGTTTTCTTTCAAATGGCTTATCACGATGCCCGCCAAATTCTGCTCTGG
GAGATCCCTGATGTGTTGAATTCACTGGCGGTAGCTTGGATGATACTGAGAGCCATAACATTCA
CAACGACATCAAACGTGGTTGTTCCGCTGCTAGCCCTGCTAACACCCGGGCTGAGATGCTTGA
ATCTGGATGTGTACAGGATACTGCTGTTGATGGTCGGAATAGGCAGCTTGATCAGGGAGAAGA
GGAGTGCAGCCGCAAAAAAGAAAGGAGCAAGTCTGCTATGCTTGGCTCTAGCCTCAACAGG
ACTTTTCAACCCCATGATCCTTGCTGCTGGACTGATTGCATGTGATCCCAACCGTAAACGCGG
ATGGCCCGCAACTGAAGTGATGACAGCTGTCGGCCTAATGTTTGCCATCGTCGGAGGGCTGGC
AGAGCTTGACATTGACTCCATGGCCATTCCAATGACTATCGCGGGGCTCATGTTTGCTGCTTTC
GTGATTTCTGGGAAATCAACAGATATGTGGATTGAGAGAACGGCGGACATTTCCTGGGAAAGT
GATGCAGAAATTACAGGCTCGAGCGAAAGAGTTGATGTGCGGCTTGATGATGATGGAAACTT
CCAGCTCATGAATGATCCAGGAGCACCTTGGAAGATATGGATGCTCAGAATGGTCTGTCTCGC

File S2 ISA fragments

GATTAGTGCGTACACCCCCTGGGCAATCTTGCCCTCAGTAGTTGGATTTTGGATAACTCTCCAA
TACACAAAGAGAGGAGGCGTGTTGTGGGACACTCCCTCACCAAAGGAGTACAAAAAGGGGG
ACACGACCACCGGCGTCTACAGGATCATGACTCGTGGGCTGCTCGGCAGTTATCAAGCAGGA
GCGGGCGTGATGGTTGAAGGTGTTTTCCACACCCTTTGGCATACAACAAAAGGAGCCGCTTT
GATGAGCGGAGAGGGCCGCCTGGACCCATACTGGGGCAGTGTCAAGGAGGATCGACTTTGTT
ACGGAGGACCCTGGAAATTGCAGCACAAGTGGAACGGGCAGGATGAGGTGCAGATGATTGT
GGTGGAACCTGGCAAGAACGTTAAGAACGTCCAGACGAAACCAGGGGTGTTCAAAACACCT
GAAGGAGAAATCGGGGCCGTGACTTTGGACTTCCCCACTGGAACATCAGGCTCACCAATAGT
GGACAAAAACGGTGATGTGATTGGGCTTTATGGCAATGGAGTCATAATGCCCAACGGCTCATA
CATAAGCGCGATAGTGCAGGGTGAAAGGATGGATGAGCCAATCCCAGCCGGATTCGAACCTG
AGATGCTGAGGAAAAAACAGATCACTGTACTGGATCTCCATCCCGGCGCCGGTAAAACAAGG
AGGATTCTGCCACAGATCATCAAAGAGGCCATAAACAGAAGACTGAGAACAGCCGTGCTAGC
GCCAACCAGGGTTGTGGCTGCTGAGATGGCTGAAGCACTGAGAGGACTGCCCATCCGGTACC
AGACATCCGCAGTGCCCAGAGAACATAATGGAAATGAGATTGTTGATGTCATGTGTCATGCTA
CCCTCACCCACAGGCTGATGTCTCCTCACAGGGTGCCGAACTACAACCTGTTCGTGATGGATG
AGGCTCATTTCACCGACCCAGCTAGCATTGCAGCAAGAGGTTACATTTCCACAAAGGTCGAG
CTAGGGGAGGCGGCGGCAATATTCATGACAGCCACCCCACCAGGCACTTCAGATCCATTCCCA
GAGTCCAATTCACCAATTTCCGACTTACAGACTGAGATCCCGGATCGAGCTTGGAACTCTGGA
TACGAATGGATCACAGAATACACCGGGAAGACGGTTTGGTTTGTGCCTAGTGTCAAGATGGG
GAATGAGATTGCCCTTTGCCTACAACGTGCTGGAAAGAAAGTAGTCCAATTGAACAGAAAGT
CGTACGAGACGGAGTACCCAAAATGTAAGAACGATGATTGGGACTTTGTTATCACAACAGAC
ATATCTGAAATGGGGGCTAACTTCAAGGCGAGCAGGGTGATTGACAGCCGGAAGAGTGTGAA
ACCAACCATCATAACAGAAGGAGAAGGGAGAGTGATCCTGGGAGAACCATCTGCAGTGACA
GCAGCTAGTGCCGCCCAGAGACGTGGACGTATCGGTAGAAATCCGTCGCAAGTTGGTGATGA
GTACTGTTATGGGGGCACACGAATGAAGACGACTCGAACTTCGCCCATTGGACTGAGGCAC
GAATCATGCTGGACAACATCAACATGCCAAACGGACTGATCGCTCAATTCTACCAACCAGAGC
GTGAGAAGGTATATACCATGGATGGGGAATACCGGCTCAGAGGAGAAGAGAGAAAAAACTTT
CTGGAACTGTTGAGGACTGCAGATCTGCCAGTTTGGCTGGCTTACAAGGTTGCAGCGGCTGG
AGTGTCATACCACGACCGGAGGTGGTGCTTTGATGGTCCTAGGACAAACACAATTTTAGAAG
ACAACAACGAAGTGGAAGTCATCACGAAGCTTGGTGAAAGGAAGATTCTGAGGCCGCGCTG
GATTGATGCCAGGGTGTACTCGGATCACCAGGCACTAAAGGCGTTCAAGGACTTCGCCTCGG
GAAAACGTTCTCAGATAGGGCTCATTGAGGTTCTGGGAAAGATGCCTGAGCACTTCATGGGG
AAGACATGGGAAGCACTTGACACCATGTACGTTGTGGCCACTGCAGAGAAAGGAGGAAGAG
CTCACAGAATGGCCCTGGAGGAACTGCCAGATGCTCTTCAGACAATTGCCTTGATTGCCTTAT
TGAGTGTGATGACCATGGGAGTATTCTTCCTCCTCATGCAGCGGAAGGGCATTGGAAAGATAG
GTTTGGGAGGCGCTGTCTTGGGAGTCGCGACCTTTTTCTGTTGGATGGCTGAAGTTCCAGGA
ACGAAGATCGCCGGAATGTTGCTGCTCTCCCTTCTCTTGATGATTGTGCTAATTCCTGAGCCAG
AGAAGCAACGTTCGCAGACAGACAACCAGCTAGCCGTGTTCCTGATTTGTGTCATGACCCTT
GTGAGCGCAGTGGCAGCCAACGAGATGGGTTGGCTAGATAAGACCAAGAGTGACATAAGCA
GTTTGTTTGGGCAAAGAATTGAGGTCAAGGAGAATTTCAGCATGGGAGAGTTTCTTCTGGAC
TTGAGGCCGGCAACAGCCTGGTCACTGTACGCTGTGACAACAGCGGTCCTCACTCCACTGCT
AAAGCATTTGATCACGTCAGATTACATCAACACCTCATTGACCTCAATAAACGTTCAGGCAAG
TGCACTATTCACACTCGCGCGAGGCTTCCCCTTCGTCGATGTTGGAGTGTCGGCTCTCCTGCT
AGCAGCCGGATGCTGGGGACAAGTCACCCTCACCGTTACGGTAACAGCGGCAACACTCCTTT
TTTGCCACTATGCCTACATGGTTCCCGGTTGGCAAGCTGAGGCAATGCGCTCAGCCCAGCGGC
GGACAGCGGCCGGAATCATGAAGAACGCTGTAGTGGATGGCATCGTGGCCACGGACGTCCCA
GAATTAGAGCGCACCACACCCATCATGCAGAAGAAAGTTGGACAGATCATGCTGATCTTGGTG

File S2 ISA fragments

TCTCTAGCTGCAGTAGTAGTGAACCCGTCTGTGAAGACAGTACGAGAAGCCGGAATTTTGATC
ACGGCCGCAGCGGTGACGCTTTGGGAGAATGGAGCAAGCTCTGTTTGGAACGCAACAACTG
CCATCGGACTCTGCCACATCATGCGTGGGGGTTGGTTGTCATGTCTATCCATAACATGGACACT
CATAAAGAACATGGAAAAACCAGGACTAAAAAGAGGTGGGGCAAAAGGACGCACCTTGGGA
GAGGTTTGGAAAGAAAGACTCAACCAGATGACAAAAGAAGAGTTCACT

>NY99-CpG-NS5_**Fragment-III**
GGTGGGGCAAAAGGACGCACCTTGGGAGAGGTTTGGAAAGAAAGACTCAACCAGATGACAA
AAGAAGAGTTCACTAGGTACCGCAAAGAAGCGATCATAGAAGTCGATCGATCGGCAGCGAAA
CACGCGCGAAAAGAAGGAAACGTAACGGGCGGTCATCCCGTTTCACGCGGGACGGCGAAAC
TTCGATGGCTTGTAGAACGGCGGTTTCTCGAACCGGTCGGAAAAGTGATAGATCTAGGATGCG
GACGCGGAGGTTGGTGTTATTACATGGCGACACAAAAACGCGTTCAAGAAGTCCGTGGGTAC
ACGAAAGGAGGTCCGGGACACGAAGAACCGCAACTCGTTCAAAGTTACGGATGGAACATCG
TCACCATGAAGAGCGGCGTCGACGTTTTTTATCGACCTTCGGAATGTTGCGACACACTTCTAT
GTGACATTGGCGAGTCTTCATCAAGTGCGGAAGTCGAAGAACATCGCACAATTCGCGTTCTAG
AAATGGTCGAAGACTGGCTACATCGCGGACCGCGCGAATTTTGTGTGAAAGTGCTCTGTCCGT
ACATGCCGAAAGTTATCGAAAAGATGGAACTGCTTCAACGTCGTTATGGCGGAGGACTCGTG
CGAAATCCGCTGTCTCGGAATTCGACGCACGAAATGTATTGGGTGAGTCGAGCGTCGGGTAAT
GTCGTCCATTCGGTAAACATGACGAGCCAAGTTCTCCTCGGACGAATGGAAAAACGAACGTG
GAAGGGACCGCAATACGAAGAAGATGTCAATTTGGGAAGCGGAACCCGAGCGGTCGGAAAA
CCGCTTCTGAATTCCGACACGAGCAAAATCAAAAACCGAATCGAACGACTCCGACGCGAATA
CAGTTCGACATGGCATCACGATGAGAATCATCCTTATCGAACCTGGAATTATCACGGGAGTTAC
GACGTGAAGCCGACGGGATCGGCCAGTTCGCTCGTGAACGGAGTGGTTCGACTTCTGTCAAA
ACCGTGGGACACGATAACGAACGTAACGACAATGGCCATGACGGACACGACTCCGTTCGGTC
AGCAACGCGTTTTCAAAGAGAAAGTTGACACGAAAGCGCCGGAACCGCCGGAAGGCGTGAA
GTACGTGCTTAACGAAACGACCAATTGGTTGTGGGCGTTTTTGGCACGCGAAAAACGACCTC
GAATGTGTTCGCGAGAAGAATTCATTCGAAAAGTCAACAGCAACGCGGCATTGGGCGCGATG
TTCGAAGAGCAAAATCAATGGCGGAGCGCGCGAGAAGCGGTTGAAGATCCCAAATTTTGGGA
AATGGTGGACGAGGAGCGAGAAGCGCATCTGCGCGGCGAATGTCACACGTGCATTTACAACA
TGATGGGAAAGCGCGAGAAAAAACCAGGCGAATTTGGAAAAGCAAAAGGAAGCCGCGCGAT
TTGGTTCATGTGGCTCGGCGCTCGATTTCTCGAATTTGAGGCGCTCGGTTTTCTCAACGAAGA
TCATTGGCTAGGACGAAAAAATTCCGGAGGCGGCGTCGAAGGATTGGGTCTGCAAAAACTCG
GTTACATTCTTCGTGAAGTTGGAACGCGGCCAGGCGGAAAGATCTACGCGGACGACACGGCA
GGATGGGACACGCGAATTACGCGAGCGGATTTGGAAAACGAAGCGAAAGTACTCGAGCTGCT
TGATGGCGAACATCGTCGTCTCGCGCGAGCCATCATCGAGCTGACGTATCGTCACAAAGTCGT
CAAAGTGATGCGGCCGGCGGCGGACGGACGAACGGTGATGGACGTCATCTCGCGAGAAGAT
CAGCGTGGGAGTGGACAAGTCGTTACATACGCGCTCAACACGTTCACCAATCTCGCCGTGCA
ACTAGTTCGAATGATGGAAGGAGAAGGCGTGATCGGACCGGATGACGTCGAAAAACTTACAA
AAGGCAAAGGACCGAAAGTACGGACGTGGCTGTTCGAAAATGGAGAAGAACGACTGAGTCG
AATGGCGGTTAGTGGCGACGATTGCGTCGTTAAACCGCTCGACGATCGATTCGCGACCTCGCT
TCATTTCCTTAACGCGATGTCGAAGGTTCGAAAAGACATTCAAGAATGGAAACCCTCGACGG
GATGGTATGATTGGCAACAAGTTCCGTTTTGTTCGAATCATTTCACCGAATTGATAATGAAAGA
CGGACGAACGCTTGTTGTTCCTTGTCGAGGACAAGATGAATTGGTCGGGCGCGCACGAATTT
CGCCGGGCGCAGGATGGAATGTTCGCGACACGGCGTGTCTCGCAAAATCGTACGCGCAGATG
TGGCTGCTTCTGTATTTTCATCGACGCGATCTTCGACTCATGGCGAACGCGATTTGTTCGGCAG
TTCCGGTGAATTGGGTTCCAACGGGACGAACAACGTGGTCAATCCACGCGGGCGGTGAGTGG
ATGACAACGGAAGACATGTTGGAAGTCTGGAACCGCGTTTGGATAGAAGAGAATGAATGGAT

File S2 ISA fragments

GGAAGACAAAACGCCGGTCGAGAAATGGAGCGATGTTCCGTATTCGGGAAAACGCGAGGAC
ATTTGGTGCGGGAGTCTGATCGGAACGCGAGCGCGCGCCACGTGGGCGGAAAACATTCAAGT
CGCAATCAATCAAGTCCGTGCGATCATCGGAGATGAGAAGTATGTGGACTACATGAGTTCACT
AAAGAGATATGAAGACACAACTTTGGTTGAGGACACAGTACTGTAGATATTTAATCAATTGTA
AATAGACAATATAAGTATGCATAAAAGTGTAGTTTTATAGTAGTATTTAGTGGTGTTAGTGTAAA
TAGTTAAGAAAATTTTGAGGAGAAAGTCAGGCCGGGAAGTTCCCGCCACCGGAAGTTGAGTA
GACGGTGCTGCCTGCGACTCAACCCCAGGAGGACTGGGTGAACAAAGCCGCGAAGTGATCC
ATGTAAGCCCTCAGAACCGTCTCGGAAGGAGGACCCCACATGTTGTAACTTCAAAGCCCAAT
GTCAGACCACGCTACGGCGTGCTACTCTGCGGAGAGTGCAGTCTGCGATAGTGCCCCAGGAG
GACTGGGTTAACAAAGGCAAACCAACGCCCCACGCGGCCCTAGCCCCGGTAATGGTGTTAAC
CAGGGCGAAAGGACTAGAGGTTAGAGGAGACCCCGCGGTTTAAAGTGCACGGCCCAGCCTG
GCTGAAGCTGTAGGTCAGGGGAAGGACTAGAGGTTAGTGGAGACCCCGTGCCACAAAACAC
CACAACAAAACAGCATATTGACACCTGGGATAGACTAGGAGATCTTCTGCTCTGCACAACCAG
CCACACGGCACAGTGCGCCGACAATGGTGGCTGGTGGTGCGAGAACACAGGATCTGGCCGG
CATGGTCCCAGCCTCCTCGCTGGCGCCGGCTGGGCAACATTCCGAGGGGACCGTCCCCTCGG
TAATGGCGAATGGGACTCGCGACAGACATGATAAGATACATTGATGAGTTTGGACAAACCACA
ACTAGAATGCAGTGAAAAAAATGCTTTATTTGTGAAATTAAGCGCTGGCATTGACCCTGAG

File S2 ISA fragments

<u>E-MAX/NS5-MAX</u>

Highlighted in red – pCMV promoter sequence.
Highlighted in green – HDR/SV40pA sequence.

<u>In brown are 22 nt adapters applied during synthesis of DNA without using bacterial plasmids and bacteria.</u>

>NY99-E_CpG+UpA_**Fragment-I**
CACCCAACTGATCTTCAGCATCTTCAATATTGGCCATTAGCCATATTATTCATTGGTTATATAGCA
TAAATCAATATTGGCTATTGGCCATTGCATACGTTGTATCTATATCATAATATGTACATTTATATTG
GCTCATGTCCAATATGACCGCCATGTTGGCATTGATTATTGACTAGTTATTAATAGTAATCAATTA
CGGGGTCATTAGTTCATAGCCCATATATGGAGTTCCGCGTTACATAACTTACGGTAAATGGCCC
GCCTGGCTGACCGCCCAACGACCCCCGCCCATTGACGTCAATAATGACGTATGTTCCCATAGT
AACGCCAATAGGGACTTTCCATTGACGTCAATGGGTGGAGTATTTACGGTAAACTGCCCACTT
GGCAGTACATCAAGTGTATCATATGCCAAGTCCGCCCCCTATTGACGTCAATGACGGTAAATGG
CCCGCCTGGCATTATGCCCAGTACATGACCTTACGGGACTTTCCTACTTGGCAGTACATCTACG
TATTAGTCATCGCTATTACCATGGTGATGCGGTTTTGGCAGTACACCAATGGGCGTGGATAGCG
GTTTGACTCACGGGGATTTCCAAGTCTCCACCCCATTGACGTCAATGGGAGTTTGTTTTGGCA
CCAAAATCAACGGGACTTTCCAAAATGTCGTAATAACCCCGCCCCGTTGACGCAAATGGGCG
GTAGGCGTGTACGGTGGGAGGTCTATATAAGCAGAGCTCGTTTAGTGAACCGAGTAGTTCGCC
TGTGTGAGCTGACAAACTTAGTAGTGTTTGTGAGGATTAACAACAATTAACACAGTGCGAGCT
GTTTCTTAGCACGAAGATCTCGATGTCTAAGAAACCAGGAGGGCCCGGCAAGAGCCGGGCTG
TCAATATGCTAAAACGCGGAATGCCCCGCGTGTTGTCCTTGATTGGACTGAAGAGGGCTATGT
TGAGCCTGATCGACGGCAAGGGGCCAATACGATTTGTGTTGGCTCTCTTGGCGTTCTTCAGGT
TCACAGCAATTGCTCCGACCCGAGCAGTGCTGGATCGATGGAGAGGTGTGAACAAACAAACA
GCGATGAAACACCTTCTGAGTTTTAAGAAGGAACTAGGGACCTTGACCAGTGCTATCAATCGG
CGGAGCTCAAAACAAAAGAAAAGAGGAGGAAAGACCGGAATTGCAGTCATGATTGGCCTGA
TCGCCAGCGTAGGAGCAGTTACCCTCTCTAACTTCCAAGGGAAGGTGATGATGACGGTAAATG
CTACTGACGTCACAGATGTCATCACGATTCCAACAGCTGCTGGAAAGAACCTATGCATTGTCA
GAGCAATGGATGTGGGATACATGTGCGATGATACTATCACTTATGAATGCCCAGTACTGTCGGC
TGGTAATGATCCAGAAGACATCGACTGTTGGTGCACAAAGTCAGCAGTCTACGTCAGGTATGG
AAGATGCACCAAGACACGCCACTCAAGACGCAGTCGGAGGTCACTGACAGTGCAGACACAC
GGAGAAAGCACTCTAGCGAACAAGAAGGGGGCTTGGATGGACAGCACCAAGGCCACAAGGT
ATTTGGTAAAAACAGAATCATGGATCTTGAGGAACCCTGGATATGCCCTGGTGGCAGCCGTCA
TTGGTTGGATGCTTGGGAGCAACACCATGCAGAGAGTTGTGTTTGTCGTGCTATTGCTTTTGG
TGGCCCCAGCTTACAGCTTCAACTGCCTTGGAATGAGCAACAGAGACTTTTTGGAAGGCGTT
TCCGGCGCGACGTGGGTCGATTTAGTTCTCGAAGGGGATAGTTGCGTTACGATAATGTCGAAA
GACAAACCGACGATCGACGTTAAAATGATGAATATGGAGGCCGCGAATCTAGCGGAAGTTCG
CAGTTATTGTTATTTAGCAACCGTAAGCGATCTTTCGACGAAAGCCGCGTGTCCGACGATGGG
CGAAGCGCACAACGATAAACGGGCGGATCCCGCGTTTGTTTGTCGACAAGGCGTCGTCGATC
GCGGTTGGGGAAATGGTTGTGGACTATTCGGCAAAGGAAGTATCGATACGTGCGCGAAATTTG
CGTGTTCTACTAAAGCGATAGGACGAACGATATTAAAAGAAAATATCAAATACGAAGTCGCGA
TTTTTGTCCACGGACCGACGACGGTCGAATCGCACGGAAATTATTCGACGCAAGTCGGCGCG
ACGCAGGCCGGTCGACTAAGTATAACGCCCGCGGCGCCGTCGTACACGCTAAAACTGGGCGA
ATATGGCGAGGTTACGGTCGACTGCGAACCGCGTTCGGGTATCGATACGAACGCGTATTATGTT
ATGACGGTCGGAACGAAAACGTTTTAGTACATCGGGAATGGTTCATGGATCTAAACCTACCG

File S2 ISA fragments

TGGAGCAGTGCCGGAAGTACCGTTTGGCGTAACCGGGAAACGTTAATGGAATTCGAAGAACC
GCACGCGACTAAACAGTCCGTAATCGCGTTAGGATCGCAAGAAGGCGCGCTTCATCAAGCGT
TAGCCGGCGCGATTCCGGTCGAATTTTCGAGTAATACCGTCAAATTAACATCCGGTCATTTGAA
ATGTCGGGTCAAAATGGAAAAATTACAATTAAAAGGAACGACTTACGGCGTATGTTCGAAAG
CGTTCAAGTTTCTCGGTACACCCGCCGATACCGGTCACGGAACCGTCGTTTTGGAATTACAAT
ATACCGGAACCGATGGACCGTGTAAAGTTCCGATATCGTCCGTGGCGTCGTTGAACGATCTAA
CACCCGTCGGCCGATTAGTCACCGTTAATCCGTTCGTTTCCGTCGCGACGGCGAACGCGAAAG
TACTTATAGAATTGGAACCGCCGTTTGGCGATTCGTATATCGTCGTCGGCCGCGGGGAACAAC
AAATAAATCACCATTGGCATAAGTCCGGAAGTAGTATCGGAAAAGCGTTTACGACGACGCTAA
AAGGCGCGCAACGACTAGCCGCGCTCGGCGATACCGCGTGGGATTTCGGATCCGTCGGGGGC
GTTTTTACGTCCGTCGGTAAAGCCGTACATCAAGTATTCGGCGGGGCGTTTCGCTCGCTTTTCG
GCGGAATGTCGTGGATAACGCAAGGATTACTGGGCGCGCTACTTTTATGGATGGGAATAAACG
CGCGGGATCGTTCGATCGCGCTAACGTTTCTCGCCGTCGGCGGAGTTCTGCTCTTCCTCTCCG
TGAACGTGCACGCTGACACTGGGTGTGCCATAGACATCAGCCGGCAAGAGCTGAGATGTGGA
AGTGGAGTGTTCATACACAATGATGTGGAGGCTTGGATGGACCGGTACAAGTATTACCCTGAA
ACGCCACAAGGCCTAGCCAAGATCATTCAGAAAGCTCATAAGGAAGGAGTGTGCGGTCTACG
ATCAGTTTCCAGACTGGAGCATCAAATGTGGGAAGCAGTGAAGGACGAGCTGAACACTCTTT
TGAAGGAGAATGGTGTGGACCTTAGTGTCGTGGTTGAGAAACAGGAGGGAATGTACAAGTCA
GCACCTAAACGCCTCACCGCCACCACGGAAAAATTGGAAATTGGCTGGAAGGCCTGGGGAA
AGAGTATTTTATTTGCACCAGAACTCGCCAACAACACCTTTGTGGTTGATGGTCCGGAGACCA
AGGAATGTCCGACTCAGAATCGCGCTTGGAATAGCTTAGAAGTGGAGGATTTTGGATTTGGTC
TCACCAGCACTCGGATGTTCCTGAAGGTCAGAGAGAGCAACACAACTGAATGTGACTCGAAG
ATCATTGGAACGGCTGTCAAGAACAACTTGGCGATCCACAGTGACCTGTCCTATTGGATTGAA
AGCAGGCTCAATGATACGTGGAAGCTTGAAAGGGCAGTTCTGGGTGAAGTCAAATCATGTAC
GTGGCCTGAGACGCATACCTTGTGGGGCGATGGAATCCTTGAGAGTGACTTGATAATACCAGT
CACACTGGCGGGACCACGAAGCAATCACAATCGGAGACCTGGGTACAAGACACAAAACCAG
GGCCCATGGGACGAAGGCCGGGTAGAGATTGACTTCGATTACTGCCCAGGAACTACGGTCAC
CCTGAGTGAGAGCTGCGGACACCGTGGACCTGCCACTCGCACCACCACAGAGAGCGGAAAG
TTGATAACAGATTGGTGCTGCAGGAGCTGCACCTTACCACCACTGCGCTACCAAACTGACAGC
GGCTGTTGGTATGGTATGGAGATCAGACCACAGAGACATGATGAAAAGACCCTCGTGCAGTC
ACAAGTGAATGCTTATAATGCTGATATGATTGACCCTTTTCAGTTGGGCCTTCTGGTCGTGTTC
TTGGCCACCCAGGAGGTCCTTCGC

>wt-NY99_**Fragment-II**
TATAATGCTGATATGATTGACCCTTTTCAGTTGGGCCTTCTGGTCGTGTTCTTGGCCACCCAGG
AGGTCCTTCGCAAGAGGTGGACAGCCAAGATCAGCATGCCAGCTATACTGATTGCTCTGCTAG
TCCTGGTGTTTGGGGGCATTACTTACACTGATGTGTTACGCTATGTCATCTTGGTGGGGGCAGC
TTTCGCAGAATCTAATTCGGGAGGAGACGTGGTACACTTGGCGCTCATGGCGACCTTCAAGAT
ACAACCAGTGTTTATGGTGGCATCGTTTCTCAAAGCGAGATGGACCAACCAGGAGAACATTTT
GTTGATGTTGGCGGCTGTTTTCTTTCAAATGGCTTATCACGATGCCCGCCAAATTCTGCTCTGG
GAGATCCCTGATGTGTTGAATTCACTGGCGGTAGCTTGGATGATACTGAGAGCCATAACATTCA
CAACGACATCAAACGTGGTTGTTCCGCTGCTAGCCCTGCTAACACCCGGGCTGAGATGCTTGA
ATCTGGATGTGTACAGGATACTGCTGTTGATGGTCGGAATAGGCAGCTTGATCAGGGAGAAGA
GGAGTGCAGCCGCAAAAAAGAAAGGAGCAAGTCTGCTATGCTTGGCTCTAGCCTCAACAGG
ACTTTTCAACCCCATGATCCTTGCTGCTGGACTGATTGCATGTGATCCCAACCGTAAACGCGG
ATGGCCCGCAACTGAAGTGATGACAGCTGTCGGCCTAATGTTTGCCATCGTCGGAGGGCTGGC
AGAGCTTGACATTGACTCCATGGCCATTCCAATGACTATCGCGGGGCTCATGTTTGCTGCTTTC

File S2 ISA fragments

GTGATTTCTGGGAAATCAACAGATATGTGGATTGAGAGAACGGCGGACATTTCCTGGGAAAGT
GATGCAGAAATTACAGGCTCGAGCGAAAGAGTTGATGTGCGGCTTGATGATGATGGAAACTT
CCAGCTCATGAATGATCCAGGAGCACCTTGGAAGATATGGATGCTCAGAATGGTCTGTCTCGC
GATTAGTGCGTACACCCCCTGGGCAATCTTGCCCTCAGTAGTTGGATTTTGGATAACTCTCCAA
TACACAAAGAGAGGAGGCGTGTTGTGGGACACTCCCTCACCAAAGGAGTACAAAAAGGGGG
ACACGACCACCGGCGTCTACAGGATCATGACTCGTGGGCTGCTCGGCAGTTATCAAGCAGGA
GCGGGCGTGATGGTTGAAGGTGTTTTCCACACCCTTTGGCATACAACAAAAGGAGCCGCTTT
GATGAGCGGAGAGGGCCGCCTGGACCCATACTGGGGCAGTGTCAAGGAGGATCGACTTTGTT
ACGGAGGACCCTGGAAATTGCAGCACAAGTGGAACGGGCAGGATGAGGTGCAGATGATTGT
GGTGGAACCTGGCAAGAACGTTAAGAACGTCCAGACGAAACCAGGGGTGTTCAAAACACCT
GAAGGAGAAATCGGGGCCGTGACTTTGGACTTCCCCACTGGAACATCAGGCTCACCAATAGT
GGACAAAAACGGTGATGTGATTGGGCTTTATGGCAATGGAGTCATAATGCCCAACGGCTCATA
CATAAGCGCGATAGTGCAGGGTGAAAGGATGGATGAGCCAATCCCAGCCGGATTCGAACCTG
AGATGCTGAGGAAAAAACAGATCACTGTACTGGATCTCCATCCCGGCGCCGGTAAAACAAGG
AGGATTCTGCCACAGATCATCAAAGAGGCCATAAACAGAAGACTGAGAACAGCCGTGCTAGC
GCCAACCAGGGTTGTGGCTGCTGAGATGGCTGAAGCACTGAGAGGACTGCCCATCCGGTACC
AGACATCCGCAGTGCCCAGAGAACATAATGGAAATGAGATTGTTGATGTCATGTGTCATGCTA
CCCTCACCCACAGGCTGATGTCTCCTCACAGGGTGCCGAACTACAACCTGTTCGTGATGGATG
AGGCTCATTTCACCGACCCAGCTAGCATTGCAGCAAGAGGTTACATTTCCACAAAGGTCGAG
CTAGGGGAGGCGGCGGCAATATTCATGACAGCCACCCCACCAGGCACTTCAGATCCATTCCCA
GAGTCCAATTCACCAATTTCCGACTTACAGACTGAGATCCCGGATCGAGCTTGGAACTCTGGA
TACGAATGGATCACAGAATACACCGGGAAGACGGTTTGGTTTGTGCCTAGTGTCAAGATGGG
GAATGAGATTGCCCTTTGCCTACAACGTGCTGGAAAGAAAGTAGTCCAATTGAACAGAAAGT
CGTACGAGACGGAGTACCCAAAATGTAAGAACGATGATTGGGACTTTGTTATCACAACAGAC
ATATCTGAAATGGGGGCTAACTTCAAGGCGAGCAGGGTGATTGACAGCCGGAAGAGTGTGAA
ACCAACCATCATAACAGAAGGAGAAGGGAGAGTGATCCTGGGAGAACCATCTGCAGTGACA
GCAGCTAGTGCCGCCCAGAGACGTGGACGTATCGGTAGAAATCCGTCGCAAGTTGGTGATGA
GTACTGTTATGGGGGGCACACGAATGAAGACGACTCGAACTTCGCCCATTGGACTGAGGCAC
GAATCATGCTGGACAACATCAACATGCCAAACGGACTGATCGCTCAATTCTACCAACCAGAGC
GTGAGAAGGTATATACCATGGATGGGGAATACCGGCTCAGAGGAGAAGAGAGAAAAAACTTT
CTGGAACTGTTGAGGACTGCAGATCTGCCAGTTTGGCTGGCTTACAAGGTTGCAGCGGCTGG
AGTGTCATACCACGACCGGAGGTGGTGCTTTGATGGTCCTAGGACAAACACAATTTTAGAAG
ACAACAACGAAGTGGAAGTCATCACGAAGCTTGGTGAAAGGAAGATTCTGAGGCCGCGCTG
GATTGATGCCAGGGTGTACTCGGATCACCAGGCACTAAAGGCGTTCAAGGACTTCGCCTCGG
GAAAACGTTCTCAGATAGGGCTCATTGAGGTTCTGGGAAAGATGCCTGAGCACTTCATGGGG
AAGACATGGGAAGCACTTGACACCATGTACGTTGTGGCCACTGCAGAGAAAGGAGGAAGAG
CTCACAGAATGGCCCTGGAGGAACTGCCAGATGCTCTTCAGACAATTGCCTTGATTGCCTTAT
TGAGTGTGATGACCATGGGAGTATTCTTCCTCCTCATGCAGCGGAAGGGCATTGGAAAGATAG
GTTTGGGAGGCGCTGTCTTGGGAGTCGCGACCTTTTTCTGTTGGATGGCTGAAGTTCCAGGA
ACGAAGATCGCCGGAATGTTGCTGCTCTCCCTTCTCTTGATGATTGTGCTAATTCCTGAGCCAG
AGAAGCAACGTTCGCAGACAGACAACCAGCTAGCCGTGTTCCTGATTTGTGTCATGACCCTT
GTGAGCGCAGTGGCAGCCAACGAGATGGGTTGGCTAGATAAGACCAAGAGTGACATAAGCA
GTTTGTTTGGGCAAAGAATTGAGGTCAAGGAGAATTTCAGCATGGGAGAGTTTCTTCTGGAC
TTGAGGCCGGCAACAGCCTGGTCACTGTACGCTGTGACAACAGCGGTCCTCACTCCACTGCT
AAAGCATTTGATCACGTCAGATTACATCAACACCTCATTGACCTCAATAAACGTTCAGGCAAG
TGCACTATTCACACTCGCGCGAGGCTTCCCCTTCGTCGATGTTGGAGTGTCGGCTCTCCTGCT
AGCAGCCGGATGCTGGGGACAAGTCACCCTCACCGTTACGGTAACAGCGGCAACACTCCTTT

File S2 ISA fragments

TTTGCCACTATGCCTACATGGTTCCCGGTTGGCAAGCTGAGGCAATGCGCTCAGCCCAGCGGC
GGACAGCGGCCGGAATCATGAAGAACGCTGTAGTGGATGGCATCGTGGCCACGGACGTCCCA
GAATTAGAGCGCACCACACCCATCATGCAGAAGAAAGTTGGACAGATCATGCTGATCTTGGTG
TCTCTAGCTGCAGTAGTAGTGAACCCGTCTGTGAAGACAGTACGAGAAGCCGGAATTTTGATC
ACGGCCGCAGCGGTGACGCTTTGGGAGAATGGAGCAAGCTCTGTTTGGAACGCAACAACTG
CCATCGGACTCTGCCACATCATGCGTGGGGGTTGGTTGTCATGTCTATCCATAACATGGACACT
CATAAAGAACATGGAAAAACCAGGACTAAAAAGAGGTGGGGCAAAAGGACGCACCTTGGGA
GAGGTTTGGAAAGAAAGACTCAACCAGATGACAAAAGAAGAGTTCACT

>NY99-CpG-NS5-MAX_**Fragment-III-A**
caatccgccctcactacaaccgGGTGGGGCAAAAGGACGCACCTTGGGAGAGGTTTGGAAAGAAAGAC
TCAACCAGATGACAAAAGAAGAGTTCACTAGGTACCGTAAAGAAGCGATAATCGAAGTCGAT
CGATCGGCGGCGAAACACGCGCGAAAAGAAGGTAACGTTACGGGCGGTCATCCCGTATCGCG
CGGTACGGCGAAACTTCGATGGCTCGTCGAACGGCGGTTTCTCGAACCGGTCGGAAAAGTGA
TCGATCTCGGATGCGGACGCGGCGGTTGGTGTTATTATATGGCGACACAAAAACGCGTACAAG
AAGTACGTGGGTATACTAAAGGCGGTCCGGGACACGAAGAACCGCAACTCGTACAAAGTTAC
GGATGGAATATCGTTACTATGAAGAGCGGCGTCGACGTTTTTTATCGACCCTCGGAATGTTGCG
ATACGCTACTTTGCGATATCGGCGAGTCGTCCTCAAGCGCGGAAGTCGAAGAACATCGTACGA
TTCGCGTACTCGAAATGGTCGAAGACTGGCTGCATCGCGGTCCGCGCGAATTTTGCGTGAAA
GTGCTATGTCCGTACATGCCGAAAGTAATCGAAAAGATGGAACTGCTACAACGTCGTTACGGC
GGCGGACTAGTACGAAATCCGCTATCCCGGAATTCGACGCACGAGATGTATTGGGTGAGTCGC
GCGTCGGGTAACGTCGTACATTCGGTTAATATGACGAGCCAAGTTCTACTCGGACGAATGGAA
AAACGTACGTGGAAGGGACCGCAATACGAAGAAGACGTAAATTTAGGAAGCGGAACCCGCG
CGGTCGGAAAACCGCTTCTAAATTCCGACACGAGTAAAATAAAAAACCGAATCGAACGACTC
CGACGCGAATATAGTTCGACCTGGCATCACGACGAGAATCATCCGTATCGAACCTGGAATTATC
ACGGTAGTTACGACGTGAAGCCGACGGGATCGGCCAGTTCGCTCGTAAACGGCGTAGTTCGG
CTTCTATCTAAACCGTGGGACACGATTACGAACGTTACGACGATGGCCATGACGGACACGACT
CCGTTCGGTCAGCAACGCGTTTTTAAAGAGAAAGTCGATACGAAAGCGCCGGAACCGCCGGA
AGGCGTGAAGTACGTGCTAAACGAAACGACCAATTGGTTATGGGCGTTTTTAGCACGCGAAA
AACGTCCTC***ctactctggcgtcgatgaggga***

>NY99-CpG-NS5-MAX_**Fragment-III-B**
caatccgccctcactacaaccgCCGGAAGGCGTGAAGTACGTGCTAAACGAAACGACCAATTGGTTATGG
GCGTTTTTAGCACGCGAAAAACGTCCTCGAATGTGTTCGCGCGAAGAATTTATACGAAAAGTA
AATAGTAACGCGGCGTTAGGCGCGATGTTCGAAGAGCAAAATCAATGGCGGAGCGCGCGCGA
AGCGGTCGAAGATCCCAAATTTTGGGAGATGGTAGACGAGGAGCGCGAAGCGCATCTGCGCG
GCGAATGTCATACGTGCATTTATAATATGATGGGAAAGCGCGAGAAAAAACCCGGCGAATTCG
GAAAAGCAAAAGGAAGCCGCGCGATTTGGTTTATGTGGCTCGGCGCTCGATTTCTCGAATTCG
AGGCGCTCGGTTTTCTAAACGAAGATCATTGGCTCGGACGAAAAAATTCCGGCGGCGGCGTC
GAGGGATTGGGTCTACAAAAACTCGGTTATATTCTTCGCGAAGTCGGAACGCGGCCGGGCGG
AAAGATATACGCGGACGACACGGCGGGATGGGATACGCGAATAACGCGCGCGGATTTGGAAA
ACGAAGCGAAAGTGCTCGAGCTGCTTGACGGCGAACATCGTCGTCTCGCGCGAGCCATAATC
GAGCTAACGTATCGTCATAAAGTCGTTAAAGTGATGCGGCCGGCGGCGGACGGACGAACGGT
AATGGACGTTATATCGCGAGAAGATCAGCGTGGTAGCGGACAAGTCGTAACATACGCGCTAAA
TACGTTTACCAATCTAGCCGTACAACTCGTTCGAATGATGGAAGGCGAAGGCGTTATCGGTCC
GGACGACGTCGAGAAACTAACGAAAGGTAAAGGACCGAAAGTCCGGACGTGGCTGTTCGAG
AATGGCGAAGAACGACTAAGTCGAATGGCGGTAAGCGGCGACGATTGCGTCGTAAAACCGCT
CGACGATCGATTCGCGACCTCGCTTCATTTCCTAAACGCGATGTCGAAGGTTCGAAAAGATAT
ACAAGAATGGAAACCCTCGACGGGATGGTACGATTGGCAACAGGTTCCGTTTTGCTCGAATC
ATTTTACCGAATTAATTATGAAAGACGGACGAACGCTAGTTGTTCCTTGTCGCGGACAAGACG

File S2 ISA fragments

AATTAGTCGGGCGCGCGCGAATATCGCCGGGCGCCGGATGGAATGTACGCGACACGGCGTGT
CTCGCGAAATCGTACGCGCAGATGTGGCTGCTTCTATATTTTCATCGACGCGATCTTCGACTAA
TGGCGAACGCGATTTGTTCGGC***ctactctggcgtcgatgaggga***

>NY99-CpG-NS5-MAX_**Fragment-III-C**
caatccgccctcactacaaccgCGTACGCGCAGATGTGGCTGCTTCTATATTTTCATCGACGCGATCTTCGA
CTAATGGCGAACGCGATTTGTTCGGCGGTACCGGTAAATTGGGTACCAACGGGACGAACGAC
GTGGTCGATACACGCGGGCGGCGAGTGGATGACAACGGAAGATATGTTGGAAGTATGGAACC
GCGTTTGGATCGAAGAGAACGAATGGATGGAAGATAAAACGCCGGTCGAGAAATGGAGCGAT
GTTCCGTATTCGGGAAAACGCGAGGATATATGGTGCGGTAGTCTGATCGGAACGCGCGCGCGC
GCCACGTGGGCGGAAAATATACAAGTCGCGATAAACCAAGTACGCGCGATAATCGGAGATGA
GAAGTATGTGGACTACATGAGTTCACTAAAGAGATATGAAGACACAACTTTGGTTGAGGACA
CAGTACTGTAGATATTTAATCAATTGTAAATAGACAATATAAGTATGCATAAAAGTGTAGTTTTA
TAGTAGTATTTAGTGGTGTTAGTGTAAATAGTTAAGAAAATTTTGAGGAGAAAGTCAGGCCGG
GAAGTTCCCGCCACCGGAAGTTGAGTAGACGGTGCTGCCTGCGACTCAACCCCAGGAGGACT
GGGTGAACAAAGCCGCGAAGTGATCCATGTAAGCCCTCAGAACCGTCTCGGAAGGAGGACC
CCACATGTTGTAACTTCAAAGCCCAATGTCAGACCACGCTACGGCGTGCTACTCTGCGGAGAG
TGCAGTCTGCGATAGTGCCCCAGGAGGACTGGGTTAACAAAGGCAAACCAACGCCCCACGC
GGCCCTAGCCCCGGTAATGGTGTTAACCAGGGCGAAAGGACTAGAGGTTAGAGGAGACCCCG
CGGTTTAAAGTGCACGGCCCAGCCTGGCTGAAGCTGTAGGTCAGGGGAAGGACTAGAGGTTA
GTGGAGACCCCGTGCCACAAAACACCACAACAAAACAGCATATTGACACCTGGGATAGACTA
GGAGATCTTCTGCTCTGCACAACCAGCCACACGGCACAGTGCGCCGACAATGGTGGCTGGTG
GTGCGAGAACACAGGATCTGGCCGGCATGGTCCCAGCCTCCTCGCTGGCGCCGGCTGGGCAA
CATTCCGAGGGGACCGTCCCCTCGGTAATGGCGAATGGGACTCGCGACAGACATGATAAGATA
CATTGATGAGTTTGGACAAACCACAACTAGAATGCAGTGAAAAAAATGCTTTATTTGTGAAAT
TAAGCGCTGGCATTGACCCTGAG***ctactctggcgtcgatgaggga***

File S2 ISA fragments

WNV-WT+FVR

Highlighted in red – pCMV promoter sequence.
Highlighted in green – HDR/SV40pA sequence.

In brown are 22 nt adapters applied during synthesis of DNA without using bacterial plasmids and bacteria.

>wt-NY99-E-FVR_**Fragment-I-A**
*caatccgccctcactacaaccg*CACCCAACTGATCTTCAGCATCTTCAATATTGGCCATTAGCCATATTATT
CATTGGTTATATAGCATAAATCAATATTGGCTATTGGCCATTGCATACGTTGTATCTATATCATAAT
ATGTACATTTATATTGGCTCATGTCCAATATGACCGCCATGTTGGCATTGATTATTGACTAGTTAT
TAATAGTAATCAATTACGGGGTCATTAGTTCATAGCCCATATATGGAGTTCCGCGTTACATAACT
TACGGTAAATGGCCCGCCTGGCTGACCGCCCAACGACCCCCGCCCATTGACGTCAATAATGAC
GTATGTTCCCATAGTAACGCCAATAGGGACTTTCCATTGACGTCAATGGGTGGAGTATTTACGG
TAAACTGCCCACTTGGCAGTACATCAAGTGTATCATATGCCAAGTCCGCCCCCTATTGACGTCA
ATGACGGTAAATGGCCCGCCTGGCATTATGCCCAGTACATGACCTTACGGGACTTTCCTACTTG
GCAGTACATCTACGTATTAGTCATCGCTATTACCATGGTGATGCGGTTTTGGCAGTACACCAAT
GGGCGTGGATAGCGGTTTGACTCACGGGGATTTCCAAGTCTCCACCCCATTGACGTCAATGGG
AGTTTGTTTTGGCACCAAAATCAACGGGACTTTCCAAAATGTCGTAATAACCCCGCCCCGTTG
ACGCAAATGGGCGGTAGGCGTGTACGGTGGGAGGTCTATATAAGCAGAGCTCGTTTAGTGAA
CCGAGTAGTTCGCCTGTGTGAGCTGACAAACTTAGTAGTGTTTGTGAGGATTAACAACAATTA
ACACAGTGCGAGCTGTTTCTTAGCACGAAGATCTCGATGTCTAAGAAACCAGGAGGGCCCGG
CAAGAGCCGGGCTGTCAATATGCTAAAACGCGGAATGCCCCGCGTGTTGTCCTTGATTGGACT
GAAGAGGGCTATGTTGAGCCTGATCGACGGCAAGGGGCCAATACGATTTGTGTTGGCTCTCTT
GGCGTTCTTCAGGTTCACAGCAATTGCTCCGACCCGAGCAGTGCTGGATCGATGGAGAGGTG
TGAACAAACAAACAGCGATGAAACACCTTCTGAGTTTTAAGAAGGAACTAGGGACCTTGACC
AGTGCTATCAATCGGCGGAGCTCAAAACAAAAGAAAAGAGGAGGAAAGACCGGAATTGCAG
TCATGATTGGCCTGATCGCCAGCGTAGGAGCAGTTACCCTCTCTAACTTCCAAGGGAAGGTGA
TGATGACGGTAAATGCTACTGACGTCACAGATGTCATCACGATTCCAACAGCTGCTGGAAAGA
ACCTATGCATTGTCAGAGCAATGGATGTGGGATACATGTGCGATGATACTATCACTTATGAATG
CCCAGTACTGTCGGCTGGTAATGATCCAGAAGACATCGACTGTTGGTGCACAAAGTCAGCAG
TCTACGTCAGGTATGGAAGATGCACCAAGACACGCCACTCAAGACGCAGTCGGAGGTCACTG
ACAGTGCAGACACACGG*ctactctggcgtcgatgaggga*

>wt-NY99-E-FVR_**Fragment-I-B**
*caatccgccctcactacaaccg*GGTATGGAAGATGCACCAAGACACGCCACTCAAGACGCAGTCGGAGG
TCACTGACAGTGCAGACACACGGAGAAAGCACTCTAGCGAACAAGAAGGGGGCTTGGATGG
ACAGCACCAAGGCCACAAGGTATTTGGTAAAAACAGAATCATGGATCTTGAGGAACCCTGGA
TATGCCCTGGTGGCAGCCGTCATTGGTTGGATGCTTGGGAGCAACACCATGCAGAGAGTTGTG
TTTGTCGTGCTATTGCTTTTGGTGGCCCCAGCTTACAGCTTCAACTGCCTTGGAATGAGCAAC
AGAGACTTCTTGGAAGGAGTGTCTGGAGCAACATGGGTGGATTTGGTTCTCGAAGGCGACAG
CTGCGTGACTATCATGTCTAAGGACAAGCCTACCATCGATGTGAAGATGATGAATATGGAGGC
GGCCAACCTGGCAGAGGTCCGCAGTTATTGCTATTTGGCTACCGTCAGCGATCTCTCCACCAA
AGCTGCGTGCCCGACCATGGGAGAAGCTCACAATGACAAACGTGCTGACCCAGCTTTTGTGT
GCAGACAAGGAGTGGTGGACAGGGGCTGGGGCAACGGCTGCGGATTTTTTGGCAAAGGAAG
CATTGACACATGCGCCAAATTTGCCTGCTCTACCAAGGCAATAGGAAGAACCATCTTGAAAGA
GAATATCAAGTACGAAGTGGCCATTTTTGTCCATGGACCAACTACTGTGGAGTCGCACGGAAA
CTACTCCACACAGGTTGGAGCCACTCAGGCAGGGAGACTCAGCATCACTCCTGCGGCGCCTT

File S2 ISA fragments

CATACACACTAAAGCTTGGAGAATATGGAGAGGTGACAGTGGACTGTGAACCACGGTCAGGG
ATTGACACCAATGCATACTACGTGATGACTGTTGGAACAAAGACGTTCTTGGTCCATCGTGAG
TGGTTCATGGACCTCAACCTCCCTTGGAGCAGTGCTGGAAGTACTGTGTGGAGGAACAGAGA
GACGTTAATGGAGTTTGAGGAACCACACGCCACGAAGCAGTCTGTGATAGCATTGGGCTCAC
AAGAGGGAGCTCTGCATCAAGCTTTGGCTGGAGCCATTCCTGTGGAATTTTCAAGCAACACT
GTCAAGTTGACGTCGGGTCATTTGAAGTGTAGAGTGAAGATGGAAAAATTGCAGTTGAAGGG
AACAACCTATGGCGTCTGTTCAAAGGCTTTCAAGTTTCTTGGGACTCCCGTGGACACAGGTCA
CGGCACTGTGGTGTTGGAATTGCAGTACACTGGCACGGATGGACCTTGCAAAGTTCCTATCTC
GTCAGTGGCTTCATTGAACGACCTAACGCCAGTGGGCAGATTGGTCACTGTCAACCCTTTTGT
TTCAGTGGCCACGGCCAACGCTAAGGTCCTGATTGAATTGGAACCACCCTTTGGAGACTCATA
CATAGTGGTGGGCAGAGGAGAACAACAGATCAATCACCATTGGCACAAGTCTGGAAGCAGCA
TTGGCAAAGCCTTTACAACCACCCTCAAAGGAGCGCAGAG*ctactctggcgtcgatgaggga*

>wt-NY99-E-FVR_**Fragment-I-C**
*caatccgccctcactacaaccg*ATCACCATTGGCACAAGTCTGGAAGCAGCATTGGCAAAGCCTTTACAA
CCACCCTCAAAGGAGCGCAGAGACTAGCCGCTCTAGGAGACACAGCTTGGGACTTTGGATCA
GTTGGAGGGGTGTTCACCTCAGTTGGGCGGGCTGTCCATCAAGTGTTCGGAGGAGCATTCCG
CTCACTGTTCGGAGGCATGTCCTGGATAACGCAAGGATTGCTGGGGGCTCTCCTGTTGTGGAT
GGGCATCAATGCTCGTGATAGGTCCATAGCTCTCACGTTTCTCGCAGTTGGAGGAGTTCTGCT
CTTCCTCTCCGTGAACGTGCACGCTGACACTGGGTGTGCCATAGACATCAGCCGGCAAGAGC
TGAGATGTGGAAGTGGAGTGTTCATACACAATGATGTGGAGGCTTGGATGGACCGGTACAAG
TATTACCCTGAAACGCCACAAGGCCTAGCCAAGATCATTCAGAAAGCTCATAAGGAAGGAGT
GTGCGGTCTACGATCAGTTTCCAGACTGGAGCATCAAATGTGGGAAGCAGTGAAGGACGAGC
TGAACACTCTTTTGAAGGAGAATGGTGTGGACCTTAGTGTCGTGGTTGAGAAACAGGAGGGA
ATGTACAAGTCAGCACCTAAACGCCTCACCGCCACCACGGAAAAATTGGAAATTGGCTGGAA
GGCCTGGGGAAAGAGTATTTTATTTGCACCAGAACTCGCCAACAACACCTTTGTGGTTGATGG
TCCGGAGACCAAGGAATGTCCGACTCAGAATCGCGCTTGGAATAGCTTAGAAGTGGAGGATT
TTGGATTTGGTCTCACCAGCACTCGGATGTTCCTGAAGGTCAGAGAGAGCAACACAACTGAA
TGTGACTCGAAGATCATTGGAACGGCTGTCAAGAACAACTTGGCGATCCACAGTGACCTGTC
CTATTGGATTGAAAGCAGGCTCAATGATACGTGGAAGCTTGAAAGGGCAGTTCTGGGTGAAG
TCAAATCATGTACGTGGCCTGAGACGCATACCTTGTGGGGCGATGGAATCCTTGAGAGTGACT
TGATAATACCAGTCACACTGGCGGGACCACGAAGCAATCACAATCGGAGACCTGGGTACAAG
ACACAAAACCAGGGCCCATGGGACGAAGGCCGGGTAGAGATTGACTTCGATTACTGCCCAGG
AACTACGGTCACCCTGAGTGAGAGCTGCGGACACCGTGGACCTGCCACTCGCACCACCACAG
AGAGCGGAAAGTTGATAACAGATTGGTGCTGCAGGAGCTGCACCTTACCACCACTGCGCTAC
CAAACTGACAGCGGCTGTTGGTATGGTATGGAGATCAGACCACAGAGACATGATGAAAAGAC
CCTCGTGCAGTCACAAGTGAATGCTTATAATGCTGATATGATTGACCCTTTTCAGTTGGGCCTT
CTGGTCGTGTTCTTGGCCACCCAGGAGGTCCTTCGC*ctactctggcgtcgatgaggga*

>wt-NY99_**Fragment-II**
TATAATGCTGATATGATTGACCCTTTTCAGTTGGGCCTTCTGGTCGTGTTCTTGGCCACCCAGG
AGGTCCTTCGCAAGAGGTGGACAGCCAAGATCAGCATGCCAGCTATACTGATTGCTCTGCTAG
TCCTGGTGTTTGGGGGCATTACTTACACTGATGTGTTACGCTATGTCATCTTGGTGGGGGCAGC
TTTCGCAGAATCTAATTCGGGAGGAGACGTGGTACACTTGGCGCTCATGGCGACCTTCAAGAT
ACAACCAGTGTTTATGGTGGCATCGTTTCTCAAAGCGAGATGGACCAACCAGGAGAACATTTT
GTTGATGTTGGCGGCTGTTTTCTTTCAAATGGCTTATCACGATGCCCGCCAAATTCTGCTCTGG
GAGATCCCTGATGTGTTGAATTCACTGGCGGTAGCTTGGATGATACTGAGAGCCATAACATTCA

File S2 ISA fragments

CAACGACATCAAACGTGGTTGTTCCGCTGCTAGCCCTGCTAACACCCGGGCTGAGATGCTTGA
ATCTGGATGTGTACAGGATACTGCTGTTGATGGTCGGAATAGGCAGCTTGATCAGGGAGAAGA
GGAGTGCAGCCGCAAAAAAGAAAGGAGCAAGTCTGCTATGCTTGGCTCTAGCCTCAACAGG
ACTTTTCAACCCCATGATCCTTGCTGCTGGACTGATTGCATGTGATCCCAACCGTAAACGCGG
ATGGCCCGCAACTGAAGTGATGACAGCTGTCGGCCTAATGTTTGCCATCGTCGGAGGGCTGGC
AGAGCTTGACATTGACTCCATGGCCATTCCAATGACTATCGCGGGGCTCATGTTTGCTGCTTTC
GTGATTTCTGGGAAATCAACAGATATGTGGATTGAGAGAACGGCGGACATTTCCTGGGAAAGT
GATGCAGAAATTACAGGCTCGAGCGAAAGAGTTGATGTGCGGCTTGATGATGATGGAAACTT
CCAGCTCATGAATGATCCAGGAGCACCTTGGAAGATATGGATGCTCAGAATGGTCTGTCTCGC
GATTAGTGCGTACACCCCCTGGGCAATCTTGCCCTCAGTAGTTGGATTTTGGATAACTCTCCAA
TACACAAAGAGAGGAGGCGTGTTGTGGGACACTCCCTCACCAAAGGAGTACAAAAAGGGGG
ACACGACCACCGGCGTCTACAGGATCATGACTCGTGGGCTGCTCGGCAGTTATCAAGCAGGA
GCGGGCGTGATGGTTGAAGGTGTTTTCCACACCCTTTGGCATACAACAAAAGGAGCCGCTTT
GATGAGCGGAGAGGGCCGCCTGGACCCATACTGGGGCAGTGTCAAGGAGGATCGACTTTGTT
ACGGAGGACCCTGGAAATTGCAGCACAAGTGGAACGGGCAGGATGAGGTGCAGATGATTGT
GGTGGAACCTGGCAAGAACGTTAAGAACGTCCAGACGAAACCAGGGGTGTTCAAAACACCT
GAAGGAGAAATCGGGGCCGTGACTTTGGACTTCCCCACTGGAACATCAGGCTCACCAATAGT
GGACAAAAACGGTGATGTGATTGGGCTTTATGGCAATGGAGTCATAATGCCCAACGGCTCATA
CATAAGCGCGATAGTGCAGGGTGAAAGGATGGATGAGCCAATCCCAGCCGGATTCGAACCTG
AGATGCTGAGGAAAAAACAGATCACTGTACTGGATCTCCATCCCGGCGCCGGTAAAACAAGG
AGGATTCTGCCACAGATCATCAAAGAGGCCATAAACAGAAGACTGAGAACAGCCGTGCTAGC
GCCAACCAGGGTTGTGGCTGCTGAGATGGCTGAAGCACTGAGAGGACTGCCCATCCGGTACC
AGACATCCGCAGTGCCCAGAGAACATAATGGAAATGAGATTGTTGATGTCATGTGTCATGCTA
CCCTCACCCACAGGCTGATGTCTCCTCACAGGGTGCCGAACTACAACCTGTTCGTGATGGATG
AGGCTCATTTCACCGACCCAGCTAGCATTGCAGCAAGAGGTTACATTTCCACAAAGGTCGAG
CTAGGGGAGGCGGCGGCAATATTCATGACAGCCACCCCACCAGGCACTTCAGATCCATTCCCA
GAGTCCAATTCACCAATTTCCGACTTACAGACTGAGATCCCGGATCGAGCTTGGAACTCTGGA
TACGAATGGATCACAGAATACACCGGGAAGACGGTTTGGTTTGTGCCTAGTGTCAAGATGGG
GAATGAGATTGCCCTTTGCCTACAACGTGCTGGAAAGAAAGTAGTCCAATTGAACAGAAAGT
CGTACGAGACGGAGTACCCAAAATGTAAGAACGATGATTGGGACTTTGTTATCACAACAGAC
ATATCTGAAATGGGGGCTAACTTCAAGGCGAGCAGGGTGATTGACAGCCGGAAGAGTGTGAA
ACCAACCATCATAACAGAAGGAGAAGGGAGAGTGATCCTGGGAGAACCATCTGCAGTGACA
GCAGCTAGTGCCGCCCAGAGACGTGGACGTATCGGTAGAAATCCGTCGCAAGTTGGTGATGA
GTACTGTTATGGGGGGCACACGAATGAAGACGACTCGAACTTCGCCCATTGGACTGAGGCAC
GAATCATGCTGGACAACATCAACATGCCAAACGGACTGATCGCTCAATTCTACCAACCAGAGC
GTGAGAAGGTATATACCATGGATGGGGAATACCGGCTCAGAGGAGAAGAGAGAAAAAACTTT
CTGGAACTGTTGAGGACTGCAGATCTGCCAGTTTGGCTGGCTTACAAGGTTGCAGCGGCTGG
AGTGTCATACCACGACCGGAGGTGGTGCTTTGATGGTCCTAGGACAAACACAATTTTAGAAG
ACAACAACGAAGTGGAAGTCATCACGAAGCTTGGTGAAAGGAAGATTCTGAGGCCGCGCTG
GATTGATGCCAGGGTGTACTCGGATCACCAGGCACTAAAGGCGTTCAAGGACTTCGCCTCGG
GAAAACGTTCTCAGATAGGGCTCATTGAGGTTCTGGGAAAGATGCCTGAGCACTTCATGGGG
AAGACATGGGAAGCACTTGACACCATGTACGTTGTGGCCACTGCAGAGAAAGGAGGAAGAG
CTCACAGAATGGCCCTGGAGGAACTGCCAGATGCTCTTCAGACAATTGCCTTGATTGCCTTAT
TGAGTGTGATGACCATGGGAGTATTCTTCCTCCTCATGCAGCGGAAGGGCATTGGAAAGATAG
GTTTGGGAGGCGCTGTCTTGGGAGTCGCGACCTTTTTCTGTTGGATGGCTGAAGTTCCAGGA
ACGAAGATCGCCGGAATGTTGCTGCTCTCCCTTCTCTTGATGATTGTGCTAATTCCTGAGCCAG
AGAAGCAACGTTCGCAGACAGACAACCAGCTAGCCGTGTTCCTGATTTGTGTCATGACCCTT

File S2 ISA fragments

GTGAGCGCAGTGGCAGCCAACGAGATGGGTTGGCTAGATAAGACCAAGAGTGACATAAGCA
GTTTGTTTGGGCAAAGAATTGAGGTCAAGGAGAATTTCAGCATGGGAGAGTTTCTTCTGGAC
TTGAGGCCGGCAACAGCCTGGTCACTGTACGCTGTGACAACAGCGGTCCTCACTCCACTGCT
AAAGCATTTGATCACGTCAGATTACATCAACACCTCATTGACCTCAATAAACGTTCAGGCAAG
TGCACTATTCACACTCGCGCGAGGCTTCCCCTTCGTCGATGTTGGAGTGTCGGCTCTCCTGCT
AGCAGCCGGATGCTGGGGACAAGTCACCCTCACCGTTACGGTAACAGCGGCAACACTCCTTT
TTTGCCACTATGCCTACATGGTTCCCGGTTGGCAAGCTGAGGCAATGCGCTCAGCCCAGCGGC
GGACAGCGGCCGGAATCATGAAGAACGCTGTAGTGGATGGCATCGTGGCCACGGACGTCCCA
GAATTAGAGCGCACCACACCCATCATGCAGAAGAAAGTTGGACAGATCATGCTGATCTTGGTG
TCTCTAGCTGCAGTAGTAGTGAACCCGTCTGTGAAGACAGTACGAGAAGCCGGAATTTTGATC
ACGGCCGCAGCGGTGACGCTTTGGGAGAATGGAGCAAGCTCTGTTTGGAACGCAACAACTG
CCATCGGACTCTGCCACATCATGCGTGGGGGTTGGTTGTCATGTCTATCCATAACATGGACACT
CATAAAGAACATGGAAAAACCAGGACTAAAAAGAGGTGGGGCAAAAGGACGCACCTTGGGA
GAGGTTTGGAAAGAAAGACTCAACCAGATGACAAAAGAAGAGTTCACT

>wt-NY99_**Fragment-III**
GGTGGGGCAAAAGGACGCACCTTGGGAGAGGTTTGGAAAGAAAGACTCAACCAGATGACAA
AAGAAGAGTTCACTAGGTACCGCAAAGAGGCCATCATCGAAGTCGATCGCTCAGCGGCAAAA
CACGCCAGGAAAGAAGGCAATGTCACTGGAGGGCATCCAGTCTCTAGGGGCACAGCAAAAC
TGAGATGGCTGGTCGAACGGAGGTTTCTCGAACCGGTCGGAAAAGTGATTGACCTTGGATGT
GGAAGAGGCGGTTGGTGTTACTATATGGCAACCCAAAAAAGAGTCCAAGAAGTCAGAGGGTA
CACAAAGGGCGGTCCCGGACATGAAGAGCCCCAACTAGTGCAAAGTTATGGATGGAACATTG
TCACCATGAAGAGTGGAGTGGATGTGTTCTACAGACCTTCTGAGTGTTGTGACACCCTCCTTT
GTGACATCGGAGAGTCCTCGTCAAGTGCTGAGGTTGAAGAGCATAGGACGATTCGGGTCCTT
GAAATGGTTGAGGACTGGCTGCACCGAGGGCCAAGGGAATTTTGCGTGAAGGTGCTCTGTCC
CTACATGCCGAAAGTCATAGAGAAGATGGAGCTGCTCCAACGCCGGTATGGGGGGGGACTGG
TCAGAAACCCACTCTCACGGAATTCCACGCACGAGATGTATTGGGTGAGTCGAGCTTCAGGC
AATGTGGTACATTCAGTGAATATGACCAGCCAGGTGCTCCTAGGAAGAATGGAAAAAAGGAC
CTGGAAGGGACCCCAATACGAGGAAGATGTAAACTTGGGAAGTGGAACCAGGGCGGTGGGA
AAACCCCTGCTCAACTCAGACACCAGTAAAATCAAGAACAGGATTGAACGACTCAGGCGTGA
GTACAGTTCGACGTGGCACCACGATGAGAACCACCCATATAGAACCTGGAACTATCACGGCA
GTTATGATGTGAAGCCCACAGGCTCCGCCAGTTCGCTGGTCAATGGAGTGGTCAGGCTCCTCT
CAAAACCATGGGACACCATCACGAATGTTACCACCATGGCCATGACTGACACTACTCCCTTCG
GGCAGCAGCGAGTGTTCAAAGAGAAGGTGGACACGAAAGCTCCTGAACCGCCAGAAGGAG
TGAAGTACGTGCTCAACGAGACCACCAACTGGTTGTGGGCGTTTTTGGCCAGAGAAAAACGT
CCCAGAATGTGCTCTCGAGAGGAATTCATAAGAAAGGTCAACAGCAATGCAGCTTTGGGTGC
CATGTTTGAAGAGCAGAATCAATGGAGGAGCGCCAGAGAAGCAGTTGAAGATCCAAAATTTT
GGGAGATGGTGGATGAGGAGCGCGAGGCACATCTGCGGGGGGAATGTCACACTTGCATTTAC
AACATGATGGGAAAGAGAGAGAAAAAACCCGGAGAGTTCGGAAAGGCCAAGGGAAGCAGA
GCCATTTGGTTCATGTGGCTCGGAGCTCGCTTTCTGGAGTTCGAGGCTCTGGGTTTTCTCAAT
GAAGACCACTGGCTTGGAAGAAAGAACTCAGGAGGAGGTGTCGAGGGCTTGGGCCTCCAAA
AACTGGGTTACATCCTGCGTGAAGTTGGCACCCGGCCTGGGGGCAAGATCTATGCTGATGACA
CAGCTGGCTGGGACACCCGCATCACGAGAGCTGACTTGGAAAATGAAGCTAAGGTGCTTGAG
CTGCTTGATGGGGAACATCGGCGTCTTGCCAGGGCCATCATTGAGCTCACCTATCGTCACAAA
GTTGTGAAAGTGATGCGCCCGGCTGCTGATGGAAGAACCGTCATGGATGTTATCTCCAGAGAA
GATCAGAGGGGGAGTGGACAAGTTGTCACCTACGCCCTAAACACTTTCACCAACCTGGCCGT
CCAGCTGGTGAGGATGATGGAAGGGGAAGGAGTGATTGGCCCAGATGATGTGGAGAAACTC
ACAAAAGGGAAAGGACCCAAAGTCAGGACCTGGCTGTTTGAGAATGGGGAAGAAAGACTCA
GCCGCATGGCTGTCAGTGGAGATGACTGTGTGGTAAAGCCCCTGGACGATCGCTTTGCCACCT

File S2 ISA fragments

CGCTCCACTTCCTCAATGCTATGTCAAAGGTTCGCAAAGACATCCAAGAGTGGAAACCGTCA
ACTGGATGGTATGATTGGCAGCAGGTTCCATTTTGCTCAAACCATTTCACTGAATTGATCATGA
AAGATGGAAGAACACTGGTGGTTCCATGCCGAGGACAGGATGAATTGGTAGGCAGAGCTCGC
ATATCTCCAGGGGCCGGATGGAACGTCCGCGACACTGCTTGTCTGGCTAAGTCTTATGCCCAG
ATGTGGCTGCTTCTGTACTTCCACAGAAGAGACCTGCGGCTCATGGCCAACGCCATTTGCTCC
GCTGTCCCTGTGAATTGGGTCCCTACCGGAAGAACCACGTGGTCCATCCATGCAGGAGGAGA
GTGGATGACAACAGAGGACATGTTGGAGGTCTGGAACCGTGTTTGGATAGAGGAGAATGAAT
GGATGGAAGACAAAACCCCAGTGGAGAAATGGAGTGACGTCCCATATTCAGGAAAACGAGA
GGACATCTGGTGTGGCAGCCTGATTGGCACAAGAGCCCGAGCCACGTGGGCAGAAAACATCC
AGGTGGCTATCAACCAAGTCAGAGCAATCATCGGAGATGAGAAGTATGTGGACTACATGAGTT
CACTAAAGAGATATGAAGACACAACTTTGGTTGAGGACACAGTACTGTAGATATTTAATCAAT
TGTAAATAGACAATATAAGTATGCATAAAAGTGTAGTTTTATAGTAGTATTTAGTGGTGTTAGTG
TAAATAGTTAAGAAAATTTTGAGGAGAAAGTCAGGCCGGGAAGTTCCCGCCACCGGAAGTTG
AGTAGACGGTGCTGCCTGCGACTCAACCCCAGGAGGACTGGGTGAACAAAGCCGCGAAGTG
ATCCATGTAAGCCCTCAGAACCGTCTCGGAAGGAGGACCCCACATGTTGTAACTTCAAAGCC
CAATGTCAGACCACGCTACGGCGTGCTACTCTGCGGAGAGTGCAGTCTGCGATAGTGCCCCA
GGAGGACTGGGTTAACAAAGGCAAACCAACGCCCCACGCGGCCCTAGCCCCGGTAATGGTGT
TAACCAGGGCGAAAGGACTAGAGGTTAGAGGAGACCCCGCGGTTTAAAGTGCACGGCCCAG
CCTGGCTGAAGCTGTAGGTCAGGGGAAGGACTAGAGGTTAGTGGAGACCCCGTGCCACAAA
ACACCACAACAAAACAGCATATTGACACCTGGGATAGACTAGGAGATCTTCTGCTCTGCACAA
CCAGCCACACGGCACAGTGCGCCGACAATGGTGGCTGGTGGTGCGAGAACACAGGATCT**GG
CCGGCATGGTCCCAGCCTCCTCGCTGGCGCCGGCTGGGCAACATTCCGAGGGGACCGT
CCCCTCGGTAATGGCGAATGGGACTCGCGACAGACATGATAAGATACATTGATGAGTTT
GGACAAACCACAACTAGAATGCAGTGAAAAAAATGCTTTATTTGTGAAATTAAGCGCTG
GCATTGACCCTGAG**

File S2 ISA fragments

WNV-WT+FR

Highlighted in red – pCMV promoter sequence.
Highlighted in green – HDR/SV40pA sequence.

In brown are 22 nt adapters applied during synthesis of DNA without using bacterial plasmids and bacteria.

>wt-NY99-E-FVR_**Fragment-I-A**
*caatccgccctcactacaaccg*CACCCAACTGATCTTCAGCATCTTCAATATTGGCCATTAGCCATATTATT
CATTGGTTATATAGCATAAATCAATATTGGCTATTGGCCATTGCATACGTTGTATCTATATCATAAT
ATGTACATTTATATTGGCTCATGTCCAATATGACCGCCATGTTGGCATTGATTATTGACTAGTTAT
TAATAGTAATCAATTACGGGGTCATTAGTTCATAGCCCATATATGGAGTTCCGCGTTACATAACT
TACGGTAAATGGCCCGCCTGGCTGACCGCCCAACGACCCCCGCCCATTGACGTCAATAATGAC
GTATGTTCCCATAGTAACGCCAATAGGGACTTTCCATTGACGTCAATGGGTGGAGTATTTACGG
TAAACTGCCCACTTGGCAGTACATCAAGTGTATCATATGCCAAGTCCGCCCCCTATTGACGTCA
ATGACGGTAAATGGCCCGCCTGGCATTATGCCCAGTACATGACCTTACGGGACTTTCCTACTTG
GCAGTACATCTACGTATTAGTCATCGCTATTACCATGGTGATGCGGTTTTGGCAGTACACCAAT
GGGCGTGGATAGCGGTTTGACTCACGGGGATTTCCAAGTCTCCACCCCATTGACGTCAATGGG
AGTTTGTTTTGGCACCAAAATCAACGGGACTTTCCAAAATGTCGTAATAACCCCGCCCCGTTG
ACGCAAATGGGCGGTAGGCGTGTACGGTGGGAGGTCTATATAAGCAGAGCTCGTTTAGTGAA
CCGAGTAGTTCGCCTGTGTGAGCTGACAAACTTAGTAGTGTTTGTGAGGATTAACAACAATTA
ACACAGTGCGAGCTGTTTCTTAGCACGAAGATCTCGATGTCTAAGAAACCAGGAGGGCCCGG
CAAGAGCCGGGCTGTCAATATGCTAAAACGCGGAATGCCCCGCGTGTTGTCCTTGATTGGACT
GAAGAGGGCTATGTTGAGCCTGATCGACGGCAAGGGGCCAATACGATTTGTGTTGGCTCTCTT
GGCGTTCTTCAGGTTCACAGCAATTGCTCCGACCCGAGCAGTGCTGGATCGATGGAGAGGTG
TGAACAAACAAACAGCGATGAAACACCTTCTGAGTTTTAAGAAGGAACTAGGGACCTTGACC
AGTGCTATCAATCGGCGGAGCTCAAAACAAAAGAAAAGAGGAGGAAAGACCGGAATTGCAG
TCATGATTGGCCTGATCGCCAGCGTAGGAGCAGTTACCCTCTCTAACTTCCAAGGGAAGGTGA
TGATGACGGTAAATGCTACTGACGTCACAGATGTCATCACGATTCCAACAGCTGCTGGAAAGA
ACCTATGCATTGTCAGAGCAATGGATGTGGGATACATGTGCGATGATACTATCACTTATGAATG
CCCAGTACTGTCGGCTGGTAATGATCCAGAAGACATCGACTGTTGGTGCACAAAGTCAGCAG
TCTACGTCAGGTATGGAAGATGCACCAAGACACGCCACTCAAGACGCAGTCGGAGGTCACTG
ACAGTGCAGACACACGG*ctactctggcgtcgatgaggga*

>wt-NY99-E-FR_**Fragment-I-B**
*caatccgccctcactacaaccg*GGTATGGAAGATGCACCAAGACACGCCACTCAAGACGCAGTCGGAGG
TCACTGACAGTGCAGACACACGGAGAAAGCACTCTAGCGAACAAGAAGGGGGCTTGGATGG
ACAGCACCAAGGCCACAAGGTATTTGGTAAAAACAGAATCATGGATCTTGAGGAACCCTGGA
TATGCCCTGGTGGCAGCCGTCATTGGTTGGATGCTTGGGAGCAACACCATGCAGAGAGTTGTG
TTTGTCGTGCTATTGCTTTTGGTGGCCCCAGCTTACAGCTTCAACTGCCTTGGAATGAGCAAC
AGAGACTTCTTGGAAGGAGTGTCTGGAGCAACATGGGTGGATTTGGTTCTCGAAGGCGACAG
CTGCGTGACTATCATGTCTAAGGACAAGCCTACCATCGATGTGAAGATGATGAATATGGAGGC
GGCCAACCTGGCAGAGGTCCGCAGTTATTGCTATTTGGCTACCGTCAGCGATCTCTCCACCAA
AGCTGCGTGCCCGACCATGGGAGAAGCTCACAATGACAAACGTGCTGACCCAGCTTTTGTGT
GCAGACAAGGAGTGGTGGACAGGGGCTGGGGCAACGGCTGCGGATTTTTGGCAAAGGAAG
CATTGACACATGCGCCAAATTTGCCTGCTCTACCAAGGCAATAGGAAGAACCATCTTGAAAGA
GAATATCAAGTACGAAGTGGCCATTTTTGTCCATGGACCAACTACTGTGGAGTCGCACGGAAA
CTACTCCACACAGGTTGGAGCCACTCAGGCAGGGAGACTCAGCATCACTCCTGCGGCGCCTT

File S2 ISA fragments

CATACACACTAAAGCTTGGAGAATATGGAGAGGTGACAGTGGACTGTGAACCACGGTCAGGG
ATTGACACCAATGCATACTACGTGATGACTGTTGGAACAAAGACGTTCTTGGTCCATCGTGAG
TGGTTCATGGACCTCAACCTCCCTTGGAGCAGTGCTGGAAGTACTGTGTGGAGGAACAGAGA
GACGTTAATGGAGTTTGAGGAACCACACGCCACGAAGCAGTCTGTGATAGCATTGGGCTCAC
AAGAGGGAGCTCTGCATCAAGCTTTGGCTGGAGCCATTCCTGTGGAATTTTCAAGCAACACT
GTCAAGTTGACGTCGGGTCATTTGAAGTGTAGAGTGAAGATGGAAAAATTGCAGTTGAAGGG
AACAACCTATGGCGTCTGTTCAAAGGCTTTCAAGTTTCTTGGGACTCCCGCAGACACAGGTC
ACGGCACTGTGGTGTTGGAATTGCAGTACACTGGCACGGATGGACCTTGCAAAGTTCCTATCT
CGTCAGTGGCTTCATTGAACGACCTAACGCCAGTGGGCAGATTGGTCACTGTCAACCCTTTTG
TTTCAGTGGCCACGGCCAACGCTAAGGTCCTGATTGAATTGGAACCACCCTTTGGAGACTCAT
ACATAGTGGTGGGCAGAGGAGAACAACAGATCAATCACCATTGGCACAAGTCTGGAAGCAGC
ATTGGCAAAGCCTTTACAACCACCCTCAAAGGAGCGCAGAG***ctactctggcgtcgatgaggga***

>wt-NY99-E-FVR_**Fragment-I-C**
caatccgccctcactacaaccgATCACCATTGGCACAAGTCTGGAAGCAGCATTGGCAAAGCCTTTACAA
CCACCCTCAAAGGAGCGCAGAGACTAGCCGCTCTAGGAGACACAGCTTGGGACTTTGGATCA
GTTGGAGGGGTGTTCACCTCAGTTGGGCGGGCTGTCCATCAAGTGTTCGGAGGAGCATTCCG
CTCACTGTTCGGAGGCATGTCCTGGATAACGCAAGGATTGCTGGGGGCTCTCCTGTTGTGGAT
GGGCATCAATGCTCGTGATAGGTCCATAGCTCTCACGTTTCTCGCAGTTGGAGGAGTTCTGCT
CTTCCTCTCCGTGAACGTGCACGCTGACACTGGGTGTGCCATAGACATCAGCCGGCAAGAGC
TGAGATGTGGAAGTGGAGTGTTCATACACAATGATGTGGAGGCTTGGATGGACCGGTACAAG
TATTACCCTGAAACGCCACAAGGCCTAGCCAAGATCATTCAGAAAGCTCATAAGGAAGGAGT
GTGCGGTCTACGATCAGTTTCCAGACTGGAGCATCAAATGTGGGAAGCAGTGAAGGACGAGC
TGAACACTCTTTTGAAGGAGAATGGTGTGGACCTTAGTGTCGTGGTTGAGAAACAGGAGGGA
ATGTACAAGTCAGCACCTAAACGCCTCACCGCCACCACGGAAAAATTGGAAATTGGCTGGAA
GGCCTGGGGAAAGAGTATTTTATTTGCACCAGAACTCGCCAACAACACCTTTGTGGTTGATGG
TCCGGAGACCAAGGAATGTCCGACTCAGAATCGCGCTTGGAATAGCTTAGAAGTGGAGGATT
TTGGATTTGGTCTCACCAGCACTCGGATGTTCCTGAAGGTCAGAGAGAGCAACACAACTGAA
TGTGACTCGAAGATCATTGGAACGGCTGTCAAGAACAACTTGGCGATCCACAGTGACCTGTC
CTATTGGATTGAAAGCAGGCTCAATGATACGTGGAAGCTTGAAAGGGCAGTTCTGGGTGAAG
TCAAATCATGTACGTGGCCTGAGACGCATACCTTGTGGGGCGATGGAATCCTTGAGAGTGACT
TGATAATACCAGTCACACTGGCGGGACCACGAAGCAATCACAATCGGAGACCTGGGTACAAG
ACACAAAACCAGGGCCCATGGGACGAAGGCCGGGTAGAGATTGACTTCGATTACTGCCCAGG
AACTACGGTCACCCTGAGTGAGAGCTGCGGACACCGTGGACCTGCCACTCGCACCACCACAG
AGAGCGGAAAGTTGATAACAGATTGGTGCTGCAGGAGCTGCACCTTACCACCACTGCGCTAC
CAAACTGACAGCGGCTGTTGGTATGGTATGGAGATCAGACCACAGAGACATGATGAAAAGAC
CCTCGTGCAGTCACAAGTGAATGCTTATAATGCTGATATGATTGACCCTTTTCAGTTGGGCCTT
CTGGTCGTGTTCTTGGCCACCCAGGAGGTCCTTCGC***ctactctggcgtcgatgaggga***

>wt-NY99_**Fragment-II**
TATAATGCTGATATGATTGACCCTTTTCAGTTGGGCCTTCTGGTCGTGTTCTTGGCCACCCAGG
AGGTCCTTCGCAAGAGGTGGACAGCCAAGATCAGCATGCCAGCTATACTGATTGCTCTGCTAG
TCCTGGTGTTTGGGGGCATTACTTACACTGATGTGTTACGCTATGTCATCTTGGTGGGGGCAGC
TTTCGCAGAATCTAATTCGGGAGGAGACGTGGTACACTTGGCGCTCATGGCGACCTTCAAGAT
ACAACCAGTGTTTATGGTGGCATCGTTTCTCAAAGCGAGATGGACCAACCAGGAGAACATTTT
GTTGATGTTGGCGGCTGTTTTCTTTCAAATGGCTTATCACGATGCCCGCCAAATTCTGCTCTGG
GAGATCCCTGATGTGTTGAATTCACTGGCGGTAGCTTGGATGATACTGAGAGCCATAACATTCA

File S2 ISA fragments

CAACGACATCAAACGTGGTTGTTCCGCTGCTAGCCCTGCTAACACCCGGGCTGAGATGCTTGA
ATCTGGATGTGTACAGGATACTGCTGTTGATGGTCGGAATAGGCAGCTTGATCAGGGAGAAGA
GGAGTGCAGCCGCAAAAAAGAAAGGAGCAAGTCTGCTATGCTTGGCTCTAGCCTCAACAGG
ACTTTTCAACCCCATGATCCTTGCTGCTGGACTGATTGCATGTGATCCCAACCGTAAACGCGG
ATGGCCCGCAACTGAAGTGATGACAGCTGTCGGCCTAATGTTTGCCATCGTCGGAGGGCTGGC
AGAGCTTGACATTGACTCCATGGCCATTCCAATGACTATCGCGGGGCTCATGTTTGCTGCTTTC
GTGATTTCTGGGAAATCAACAGATATGTGGATTGAGAGAACGGCGGACATTTCCTGGGAAAGT
GATGCAGAAATTACAGGCTCGAGCGAAAGAGTTGATGTGCGGCTTGATGATGATGGAAACTT
CCAGCTCATGAATGATCCAGGAGCACCTTGGAAGATATGGATGCTCAGAATGGTCTGTCTCGC
GATTAGTGCGTACACCCCCTGGGCAATCTTGCCCTCAGTAGTTGGATTTTGGATAACTCTCCAA
TACACAAAGAGAGGAGGCGTGTTGTGGGACACTCCCTCACCAAAGGAGTACAAAAAGGGGG
ACACGACCACCGGCGTCTACAGGATCATGACTCGTGGGCTGCTCGGCAGTTATCAAGCAGGA
GCGGGCGTGATGGTTGAAGGTGTTTTCCACACCCTTTGGCATACAACAAAAGGAGCCGCTTT
GATGAGCGGAGAGGGCCGCCTGGACCCATACTGGGGCAGTGTCAAGGAGGATCGACTTTGTT
ACGGAGGACCCTGGAAATTGCAGCACAAGTGGAACGGGCAGGATGAGGTGCAGATGATTGT
GGTGGAACCTGGCAAGAACGTTAAGAACGTCCAGACGAAACCAGGGGTGTTCAAAACACCT
GAAGGAGAAATCGGGGCCGTGACTTTGGACTTCCCCACTGGAACATCAGGCTCACCAATAGT
GGACAAAAACGGTGATGTGATTGGGCTTTATGGCAATGGAGTCATAATGCCCAACGGCTCATA
CATAAGCGCGATAGTGCAGGGTGAAAGGATGGATGAGCCAATCCCAGCCGGATTCGAACCTG
AGATGCTGAGGAAAAAACAGATCACTGTACTGGATCTCCATCCCGGCGCCGGTAAAACAAGG
AGGATTCTGCCACAGATCATCAAAGAGGCCATAAACAGAAGACTGAGAACAGCCGTGCTAGC
GCCAACCAGGGTTGTGGCTGCTGAGATGGCTGAAGCACTGAGAGGACTGCCCATCCGGTACC
AGACATCCGCAGTGCCCAGAGAACATAATGGAAATGAGATTGTTGATGTCATGTGTCATGCTA
CCCTCACCCACAGGCTGATGTCTCCTCACAGGGTGCCGAACTACAACCTGTTCGTGATGGATG
AGGCTCATTTCACCGACCCAGCTAGCATTGCAGCAAGAGGTTACATTTCCACAAAGGTCGAG
CTAGGGGAGGCGGCGGCAATATTCATGACAGCCACCCCACCAGGCACTTCAGATCCATTCCCA
GAGTCCAATTCACCAATTTCCGACTTACAGACTGAGATCCCGGATCGAGCTTGGAACTCTGGA
TACGAATGGATCACAGAATACACCGGGAAGACGGTTTGGTTTGTGCCTAGTGTCAAGATGGG
GAATGAGATTGCCCTTTGCCTACAACGTGCTGGAAAGAAAGTAGTCCAATTGAACAGAAAGT
CGTACGAGACGGAGTACCCAAAATGTAAGAACGATGATTGGGACTTTGTTATCACAACAGAC
ATATCTGAAATGGGGGCTAACTTCAAGGCGAGCAGGGTGATTGACAGCCGGAAGAGTGTGAA
ACCAACCATCATAACAGAAGGAGAAGGGAGAGTGATCCTGGGAGAACCATCTGCAGTGACA
GCAGCTAGTGCCGCCCAGAGACGTGGACGTATCGGTAGAAATCCGTCGCAAGTTGGTGATGA
GTACTGTTATGGGGGGCACACGAATGAAGACGACTCGAACTTCGCCCATTGGACTGAGGCAC
GAATCATGCTGGACAACATCAACATGCCAAACGGACTGATCGCTCAATTCTACCAACCAGAGC
GTGAGAAGGTATATACCATGGATGGGGAATACCGGCTCAGAGGAGAAGAGAGAAAAAACTTT
CTGGAACTGTTGAGGACTGCAGATCTGCCAGTTTGGCTGGCTTACAAGGTTGCAGCGGCTGG
AGTGTCATACCACGACCGGAGGTGGTGCTTTGATGGTCCTAGGACAAACACAATTTTAGAAG
ACAACAACGAAGTGGAAGTCATCACGAAGCTTGGTGAAAGGAAGATTCTGAGGCCGCGCTG
GATTGATGCCAGGGTGTACTCGGATCACCAGGCACTAAAGGCGTTCAAGGACTTCGCCTCGG
GAAAACGTTCTCAGATAGGGCTCATTGAGGTTCTGGGAAAGATGCCTGAGCACTTCATGGGG
AAGACATGGGAAGCACTTGACACCATGTACGTTGTGGCCACTGCAGAGAAAGGAGGAAGAG
CTCACAGAATGGCCCTGGAGGAACTGCCAGATGCTCTTCAGACAATTGCCTTGATTGCCTTAT
TGAGTGTGATGACCATGGGAGTATTCTTCCTCCTCATGCAGCGGAAGGGCATTGGAAAGATAG
GTTTGGGAGGCGCTGTCTTGGGAGTCGCGACCTTTTTCTGTTGGATGGCTGAAGTTCCAGGA
ACGAAGATCGCCGGAATGTTGCTGCTCTCCCTTCTCTTGATGATTGTGCTAATTCCTGAGCCAG
AGAAGCAACGTTCGCAGACAGACAACCAGCTAGCCGTGTTCCTGATTTGTGTCATGACCCTT

GTGAGCGCAGTGGCAGCCAACGAGATGGGTTGGCTAGATAAGACCAAGAGTGACATAAGCA
GTTTGTTTGGGCAAAGAATTGAGGTCAAGGAGAATTTCAGCATGGGAGAGTTTCTTCTGGAC
TTGAGGCCGGCAACAGCCTGGTCACTGTACGCTGTGACAACAGCGGTCCTCACTCCACTGCT
AAAGCATTTGATCACGTCAGATTACATCAACACCTCATTGACCTCAATAAACGTTCAGGCAAG
TGCACTATTCACACTCGCGCGAGGCTTCCCCTTCGTCGATGTTGGAGTGTCGGCTCTCCTGCT
AGCAGCCGGATGCTGGGGACAAGTCACCCTCACCGTTACGGTAACAGCGGCAACACTCCTTT
TTTGCCACTATGCCTACATGGTTCCCGGTTGGCAAGCTGAGGCAATGCGCTCAGCCCAGCGGC
GGACAGCGGCCGGAATCATGAAGAACGCTGTAGTGGATGGCATCGTGGCCACGGACGTCCCA
GAATTAGAGCGCACCACACCCATCATGCAGAAGAAAGTTGGACAGATCATGCTGATCTTGGTG
TCTCTAGCTGCAGTAGTAGTGAACCCGTCTGTGAAGACAGTACGAGAAGCCGGAATTTTGATC
ACGGCCGCAGCGGTGACGCTTTGGGAGAATGGAGCAAGCTCTGTTTGGAACGCAACAACTG
CCATCGGACTCTGCCACATCATGCGTGGGGGTTGGTTGTCATGTCTATCCATAACATGGACACT
CATAAAGAACATGGAAAAACCAGGACTAAAAAGAGGTGGGGCAAAAGGACGCACCTTGGGA
GAGGTTTGGAAAGAAAGACTCAACCAGATGACAAAAGAAGAGTTCACT

>wt-NY99_**Fragment-III**
GGTGGGGCAAAAGGACGCACCTTGGGAGAGGTTTGGAAAGAAAGACTCAACCAGATGACAA
AAGAAGAGTTCACTAGGTACCGCAAAGAGGCCATCATCGAAGTCGATCGCTCAGCGGCAAAA
CACGCCAGGAAAGAAGGCAATGTCACTGGAGGGCATCCAGTCTCTAGGGGCACAGCAAAAC
TGAGATGGCTGGTCGAACGGAGGTTTCTCGAACCGGTCGGAAAAGTGATTGACCTTGGATGT
GGAAGAGGCGGTTGGTGTTACTATATGGCAACCCAAAAAAGAGTCCAAGAAGTCAGAGGGTA
CACAAAGGGCGGTCCCGGACATGAAGAGCCCCAACTAGTGCAAAGTTATGGATGGAACATTG
TCACCATGAAGAGTGGAGTGGATGTGTTCTACAGACCTTCTGAGTGTTGTGACACCCTCCTTT
GTGACATCGGAGAGTCCTCGTCAAGTGCTGAGGTTGAAGAGCATAGGACGATTCGGGTCCTT
GAAATGGTTGAGGACTGGCTGCACCGAGGGCCAAGGGAATTTTGCGTGAAGGTGCTCTGTCC
CTACATGCCGAAAGTCATAGAGAAGATGGAGCTGCTCCAACGCCGGTATGGGGGGGGACTGG
TCAGAAACCCACTCTCACGGAATTCCACGCACGAGATGTATTGGGTGAGTCGAGCTTCAGGC
AATGTGGTACATTCAGTGAATATGACCAGCCAGGTGCTCCTAGGAAGAATGGAAAAAAGGAC
CTGGAAGGGACCCCAATACGAGGAAGATGTAAACTTGGGAAGTGGAACCAGGGCGGTGGGA
AAACCCCTGCTCAACTCAGACACCAGTAAAATCAAGAACAGGATTGAACGACTCAGGCGTGA
GTACAGTTCGACGTGGCACCACGATGAGAACCACCCATATAGAACCTGGAACTATCACGGCA
GTTATGATGTGAAGCCCACAGGCTCCGCCAGTTCGCTGGTCAATGGAGTGGTCAGGCTCCTCT
CAAAACCATGGGACACCATCACGAATGTTACCACCATGGCCATGACTGACACTACTCCCTTCG
GGCAGCAGCGAGTGTTCAAAGAGAAGGTGGACACGAAAGCTCCTGAACCGCCAGAAGGAG
TGAAGTACGTGCTCAACGAGACCACCAACTGGTTGTGGGCGTTTTTGGCCAGAGAAAAACGT
CCCAGAATGTGCTCTCGAGAGGAATTCATAAGAAAGGTCAACAGCAATGCAGCTTTGGGTGC
CATGTTTGAAGAGCAGAATCAATGGAGGAGCGCCAGAGAAGCAGTTGAAGATCCAAAATTTT
GGGAGATGGTGGATGAGGAGCGCGAGGCACATCTGCGGGGGGAATGTCACACTTGCATTTAC
AACATGATGGGAAAGAGAGAGAAAAAACCCGGAGAGTTCGGAAAGGCCAAGGGAAGCAGA
GCCATTTGGTTCATGTGGCTCGGAGCTCGCTTTCTGGAGTTCGAGGCTCTGGGTTTTCTCAAT
GAAGACCACTGGCTTGGAAGAAAGAACTCAGGAGGAGGTGTCGAGGGCTTGGGCCTCCAAA
AACTGGGTTACATCCTGCGTGAAGTTGGCACCCGGCCTGGGGGCAAGATCTATGCTGATGACA
CAGCTGGCTGGGACACCCGCATCACGAGAGCTGACTTGGAAAATGAAGCTAAGGTGCTTGAG
CTGCTTGATGGGGAACATCGGCGTCTTGCCAGGGCCATCATTGAGCTCACCTATCGTCACAAA
GTTGTGAAAGTGATGCGCCCGGCTGCTGATGGAAGAACCGTCATGGATGTTATCTCCAGAGAA
GATCAGAGGGGGAGTGGACAAGTTGTCACCTACGCCCTAAACACTTTCACCAACCTGGCCGT
CCAGCTGGTGAGGATGATGGAAGGGGAAGGAGTGATTGGCCCAGATGATGTGGAGAAACTC
ACAAAAGGGAAAGGACCCAAAGTCAGGACCTGGCTGTTTGAGAATGGGGAAGAAAGACTCA
GCCGCATGGCTGTCAGTGGAGATGACTGTGTGGTAAAGCCCCTGGACGATCGCTTTGCCACCT

File S2 ISA fragments

CGCTCCACTTCCTCAATGCTATGTCAAAGGTTCGCAAAGACATCCAAGAGTGGAAACCGTCA
ACTGGATGGTATGATTGGCAGCAGGTTCCATTTTGCTCAAACCATTTCACTGAATTGATCATGA
AAGATGGAAGAACACTGGTGGTTCCATGCCGAGGACAGGATGAATTGGTAGGCAGAGCTCGC
ATATCTCCAGGGGCCGGATGGAACGTCCGCGACACTGCTTGTCTGGCTAAGTCTTATGCCCAG
ATGTGGCTGCTTCTGTACTTCCACAGAAGAGACCTGCGGCTCATGGCCAACGCCATTTGCTCC
GCTGTCCCTGTGAATTGGGTCCCTACCGGAAGAACCACGTGGTCCATCCATGCAGGAGGAGA
GTGGATGACAACAGAGGACATGTTGGAGGTCTGGAACCGTGTTTGGATAGAGGAGAATGAAT
GGATGGAAGACAAAACCCCAGTGGAGAAATGGAGTGACGTCCCATATTCAGGAAAACGAGA
GGACATCTGGTGTGGCAGCCTGATTGGCACAAGAGCCCGAGCCACGTGGGCAGAAAACATCC
AGGTGGCTATCAACCAAGTCAGAGCAATCATCGGAGATGAGAAGTATGTGGACTACATGAGTT
CACTAAAGAGATATGAAGACACAACTTTGGTTGAGGACACAGTACTGTAGATATTTAATCAAT
TGTAAATAGACAATATAAGTATGCATAAAAGTGTAGTTTTATAGTAGTATTTAGTGGTGTTAGTG
TAAATAGTTAAGAAAATTTTGAGGAGAAAGTCAGGCCGGGAAGTTCCCGCCACCGGAAGTTG
AGTAGACGGTGCTGCCTGCGACTCAACCCCAGGAGGACTGGGTGAACAAAGCCGCGAAGTG
ATCCATGTAAGCCCTCAGAACCGTCTCGGAAGGAGGACCCCACATGTTGTAACTTCAAAGCC
CAATGTCAGACCACGCTACGGCGTGCTACTCTGCGGAGAGTGCAGTCTGCGATAGTGCCCCA
GGAGGACTGGGTTAACAAAGGCAAACCAACGCCCCACGCGGCCCTAGCCCCGGTAATGGTGT
TAACCAGGGCGAAAGGACTAGAGGTTAGAGGAGACCCCGCGGTTTAAAGTGCACGGCCCAG
CCTGGCTGAAGCTGTAGGTCAGGGGAAGGACTAGAGGTTAGTGGAGACCCCGTGCCACAAA
ACACCACAACAAAACAGCATATTGACACCTGGGATAGACTAGGAGATCTTCTGCTCTGCACAA
CCAGCCACACGGCACAGTGCGCCGACAATGGTGGCTGGTGGTGCGAGAACACAGGATCT**GG
CCGGCATGGTCCCAGCCTCCTCGCTGGCGCCGGCTGGGCAACATTCCGAGGGGACCGT
CCCCTCGGTAATGGCGAATGGGACTCGCGACAGACATGATAAGATACATTGATGAGTTT
GGACAAACCACAACTAGAATGCAGTGAAAAAAATGCTTTATTTGTGAAATTAAGCGCTG
GCATTGACCCTGAG**

File S2 ISA fragments

E-MAX+FVR

Highlighted in red – pCMV promoter sequence.
Highlighted in green – HDR/SV40pA sequence.

>wt-NY99-E-MAX-FVR_**Fragment-I**
CACCCAACTGATCTTCAGCATCTTCAATATTGGCCATTAGCCATATTATTCATTGGTTATATAGCA
TAAATCAATATTGGCTATTGGCCATTGCATACGTTGTATCTATATCATAATATGTACATTTATATTG
GCTCATGTCCAATATGACCGCCATGTTGGCATTGATTATTGACTAGTTATTAATAGTAATCAATTA
CGGGGTCATTAGTTCATAGCCCATATATGGAGTTCCGCGTTACATAACTTACGGTAAATGGCCC
GCCTGGCTGACCGCCCAACGACCCCCGCCCATTGACGTCAATAATGACGTATGTTCCCATAGT
AACGCCAATAGGGACTTTCCATTGACGTCAATGGGTGGAGTATTTACGGTAAACTGCCCACTT
GGCAGTACATCAAGTGTATCATATGCCAAGTCCGCCCCCTATTGACGTCAATGACGGTAAATGG
CCCGCCTGGCATTATGCCCAGTACATGACCTTACGGGACTTTCCTACTTGGCAGTACATCTACG
TATTAGTCATCGCTATTACCATGGTGATGCGGTTTTGGCAGTACACCAATGGGCGTGGATAGCG
GTTTGACTCACGGGGATTTCCAAGTCTCCACCCCATTGACGTCAATGGGAGTTTGTTTTGGCA
CCAAAATCAACGGGACTTTCCAAAATGTCGTAATAACCCCGCCCCGTTGACGCAAATGGGCG
GTAGGCGTGTACGGTGGGAGGTCTATATAAGCAGAGCTCGTTTAGTGAACCGAGTAGTTCGCC
TGTGTGAGCTGACAAACTTAGTAGTGTTTGTGAGGATTAACAACAATTAACACAGTGCGAGCT
GTTTCTTAGCACGAAGATCTCGATGTCTAAGAAACCAGGAGGGCCCGGCAAGAGCCGGGCTG
TCAATATGCTAAAACGCGGAATGCCCCGCGTGTTGTCCTTGATTGGACTGAAGAGGGCTATGT
TGAGCCTGATCGACGGCAAGGGGCCAATACGATTTGTGTTGGCTCTCTTGGCGTTCTTCAGGT
TCACAGCAATTGCTCCGACCCGAGCAGTGCTGGATCGATGGAGAGGTGTGAACAAACAAACA
GCGATGAAACACCTTCTGAGTTTTAAGAAGGAACTAGGGACCTTGACCAGTGCTATCAATCGG
CGGAGCTCAAAACAAAAGAAAAGAGGAGGAAAGACCGGAATTGCAGTCATGATTGGCCTGA
TCGCCAGCGTAGGAGCAGTTACCCTCTCTAACTTCCAAGGGAAGGTGATGATGACGGTAAATG
CTACTGACGTCACAGATGTCATCACGATTCCAACAGCTGCTGGAAAGAACCTATGCATTGTCA
GAGCAATGGATGTGGGATACATGTGCGATGATACTATCACTTATGAATGCCCAGTACTGTCGGC
TGGTAATGATCCAGAAGACATCGACTGTTGGTGCACAAAGTCAGCAGTCTACGTCAGGTATGG
AAGATGCACCAAGACACGCCACTCAAGACGCAGTCGGAGGTCACTGACAGTGCAGACACAC
GGAGAAAGCACTCTAGCGAACAAGAAGGGGGCTTGGATGGACAGCACCAAGGCCACAAGGT
ATTTGGTAAAAACAGAATCATGGATCTTGAGGAACCCTGGATATGCCCTGGTGGCAGCCGTCA
TTGGTTGGATGCTTGGGAGCAACACCATGCAGAGAGTTGTGTTTGTCGTGCTATTGCTTTTGG
TGGCCCCAGCTTACAGCTTCAACTGCCTTGGAATGAGCAACAGAGACTTTTTGGAAGGCGTT
TCCGGCGCGACGTGGGTCGATTTAGTTCTCGAAGGGGATAGTTGCGTTACGATAATGTCGAAA
GACAAACCGACGATCGACGTTAAAATGATGAATATGGAGGCCGCGAATCTAGCGGAAGTTCG
CAGTTATTGTTATTTAGCAACCGTAAGCGATCTTTCGACGAAAGCCGCGTGTCCGACGATGGG
CGAAGCGCACAACGATAAACGGGCGGATCCCGCGTTTGTTTGTCGACAAGGCGTCGTCGATC
GCGGTTGGGGAAATGGTTGTGGATTTTTCGGCAAAGGAAGTATCGATACGTGCGCGAAATTTG
CGTGTTCTACTAAAGCGATAGGACGAACGATATTAAAAGAAAATATCAAATACGAAGTCGCGA
TTTTTGTCCACGGACCGACGACGGTCGAATCGCACGGAAATTATTCGACGCAAGTCGGCGCG
ACGCAGGCCGGTCGACTAAGTATAACGCCCGCGGCGCCGTCGTACACGCTAAAACTGGGCGA
ATATGGCGAGGTTACGGTCGACTGCGAACCGCGTTCGGGTATCGATACGAACGCGTATTATGTT
ATGACGGTCGGAACGAAAACGTTTTTAGTACATCGGGAATGGTTCATGGATCTAAACCTACCG
TGGAGCAGTGCCGGAAGTACCGTTTGGCGTAACCGGGAAACGTTAATGGAATTCGAAGAACC
GCACGCGACTAAACAGTCCGTAATCGCGTTAGGATCGCAAGAAGGCGCGCTTCATCAAGCGT
TAGCCGGCGCGATTCCGGTCGAATTTTCGAGTAATACCGTCAAATTAACATCCGGTCATTTGAA
ATGTCGGGTCAAAATGGAAAAATTACAATTAAAAGGAACGACTTACGGCGTATGTTCGAAAG
CGTTCAAGTTTCTCGGTACACCCGTGGATACCGGTCACGGAACCGTCGTTTTGGAATTACAAT
ATACCGGAACCGATGGACCGTGTAAAGTTCCGATATCGTCCGTGGCGTCGTTGAACGATCTAA
CACCCGTCGGCCGATTAGTCACCGTTAATCCGTTCGTTTCCGTCGCGACGGCGAACGCGAAAG

File S2 ISA fragments

TACTTATAGAATTGGAACCGCCGTTTGGCGATTCGTATATCGTCGTCGGCCGCGGGGAACAAC
AAATAAATCACCATTGGCATAAGTCCGGAAGTAGTATCGGAAAAGCGTTTACGACGACGCTAA
AAGGCGCGCAACGACTAGCCGCGCTCGGCGATACCGCGTGGGATTTCGGATCCGTCGGGGGC
GTTTTTACGTCCGTCGGTCGAGCCGTACATCAAGTATTCGGCGGGGCGTTTCGCTCGCTTTTCG
GCGGAATGTCGTGGATAACGCAAGGATTACTGGGCGCGCTACTTTTATGGATGGGAATAAACG
CGCGGGATCGTTCGATCGCGCTAACGTTTCTCGCCGTCGGCGGAGTTCTGCTCTTCCTCTCCG
TGAACGTGCACGCTGACACTGGGTGTGCCATAGACATCAGCCGGCAAGAGCTGAGATGTGGA
AGTGGAGTGTTCATACACAATGATGTGGAGGCTTGGATGGACCGGTACAAGTATTACCCTGAA
ACGCCACAAGGCCTAGCCAAGATCATTCAGAAAGCTCATAAGGAAGGAGTGTGCGGTCTACG
ATCAGTTTCCAGACTGGAGCATCAAATGTGGGAAGCAGTGAAGGACGAGCTGAACACTCTTT
TGAAGGAGAATGGTGTGGACCTTAGTGTCGTGGTTGAGAAACAGGAGGGAATGTACAAGTCA
GCACCTAAACGCCTCACCGCCACCACGGAAAAATTGGAAATTGGCTGGAAGGCCTGGGGAA
AGAGTATTTTATTTGCACCAGAACTCGCCAACAACACCTTTGTGGTTGATGGTCCGGAGACCA
AGGAATGTCCGACTCAGAATCGCGCTTGGAATAGCTTAGAAGTGGAGGATTTTGGATTTGGTC
TCACCAGCACTCGGATGTTCCTGAAGGTCAGAGAGAGCAACACAACTGAATGTGACTCGAAG
ATCATTGGAACGGCTGTCAAGAACAACTTGGCGATCCACAGTGACCTGTCCTATTGGATTGAA
AGCAGGCTCAATGATACGTGGAAGCTTGAAAGGGCAGTTCTGGGTGAAGTCAAATCATGTAC
GTGGCCTGAGACGCATACCTTGTGGGGCGATGGAATCCTTGAGAGTGACTTGATAATACCAGT
CACACTGGCGGGACCACGAAGCAATCACAATCGGAGACCTGGGTACAAGACACAAAACCAG
GGCCCATGGGACGAAGGCCGGGTAGAGATTGACTTCGATTACTGCCCAGGAACTACGGTCAC
CCTGAGTGAGAGCTGCGGACACCGTGGACCTGCCACTCGCACCACCACAGAGAGCGGAAAG
TTGATAACAGATTGGTGCTGCAGGAGCTGCACCTTACCACCACTGCGCTACCAAACTGACAGC
GGCTGTTGGTATGGTATGGAGATCAGACCACAGAGACATGATGAAAAGACCCTCGTGCAGTC
ACAAGTGAATGCTTATAATGCTGATATGATTGACCCTTTTCAGTTGGGCCTTCTGGTCGTGTTC
TTGGCCACCCAGGAGGTCCTTCGC

>wt-NY99_**Fragment-II**
TATAATGCTGATATGATTGACCCTTTTCAGTTGGGCCTTCTGGTCGTGTTCTTGGCCACCCAGG
AGGTCCTTCGCAAGAGGTGGACAGCCAAGATCAGCATGCCAGCTATACTGATTGCTCTGCTAG
TCCTGGTGTTTGGGGGCATTACTTACACTGATGTGTTACGCTATGTCATCTTGGTGGGGGCAGC
TTTCGCAGAATCTAATTCGGGAGGAGACGTGGTACACTTGGCGCTCATGGCGACCTTCAAGAT
ACAACCAGTGTTTATGGTGGCATCGTTTCTCAAAGCGAGATGGACCAACCAGGAGAACATTTT
GTTGATGTTGGCGGCTGTTTTCTTTCAAATGGCTTATCACGATGCCCGCCAAATTCTGCTCTGG
GAGATCCCTGATGTGTTGAATTCACTGGCGGTAGCTTGGATGATACTGAGAGCCATAACATTCA
CAACGACATCAAACGTGGTTGTTCCGCTGCTAGCCCTGCTAACACCCGGGCTGAGATGCTTGA
ATCTGGATGTGTACAGGATACTGCTGTTGATGGTCGGAATAGGCAGCTTGATCAGGGAGAAGA
GGAGTGCAGCCGCAAAAAAGAAAGGAGCAAGTCTGCTATGCTTGGCTCTAGCCTCAACAGG
ACTTTTCAACCCCATGATCCTTGCTGCTGGACTGATTGCATGTGATCCCAACCGTAAACGCGG
ATGGCCCGCAACTGAAGTGATGACAGCTGTCGGCCTAATGTTTGCCATCGTCGGAGGGCTGGC
AGAGCTTGACATTGACTCCATGGCCATTCCAATGACTATCGCGGGGCTCATGTTTGCTGCTTTC
GTGATTTCTGGGAAATCAACAGATATGTGGATTGAGAGAACGGCGGACATTTCCTGGGAAAGT
GATGCAGAAATTACAGGCTCGAGCGAAAGAGTTGATGTGCGGCTTGATGATGATGGAAACTT
CCAGCTCATGAATGATCCAGGAGCACCTTGGAAGATATGGATGCTCAGAATGGTCTGTCTCGC
GATTAGTGCGTACACCCCCTGGGCAATCTTGCCCTCAGTAGTTGGATTTTGGATAACTCTCCAA
TACACAAAGAGAGGAGGCGTGTTGTGGGACACTCCCTCACCAAAGGAGTACAAAAAGGGGG
ACACGACCACCGGCGTCTACAGGATCATGACTCGTGGGCTGCTCGGCAGTTATCAAGCAGGA
GCGGGCGTGATGGTTGAAGGTGTTTTCCACACCCTTTGGCATACAACAAAAGGAGCCGCTTT
GATGAGCGGAGAGGGCCGCCTGGACCCATACTGGGGCAGTGTCAAGGAGGATCGACTTTGTT
ACGGAGGACCCTGGAAATTGCAGCACAAGTGGAACGGGCAGGATGAGGTGCAGATGATTGT

File S2 ISA fragments

GGTGGAACCTGGCAAGAACGTTAAGAACGTCCAGACGAAACCAGGGGTGTTCAAAACACCT
GAAGGAGAAATCGGGGCCGTGACTTTGGACTTCCCCACTGGAACATCAGGCTCACCAATAGT
GGACAAAAACGGTGATGTGATTGGGCTTTATGGCAATGGAGTCATAATGCCCAACGGCTCATA
CATAAGCGCGATAGTGCAGGGTGAAAGGATGGATGAGCCAATCCCAGCCGGATTCGAACCTG
AGATGCTGAGGAAAAAACAGATCACTGTACTGGATCTCCATCCCGGCGCCGGTAAAACAAGG
AGGATTCTGCCACAGATCATCAAAGAGGCCATAAACAGAAGACTGAGAACAGCCGTGCTAGC
GCCAACCAGGGTTGTGGCTGCTGAGATGGCTGAAGCACTGAGAGGACTGCCCATCCGGTACC
AGACATCCGCAGTGCCCAGAGAACATAATGGAAATGAGATTGTTGATGTCATGTGTCATGCTA
CCCTCACCCACAGGCTGATGTCTCCTCACAGGGTGCCGAACTACAACCTGTTCGTGATGGATG
AGGCTCATTTCACCGACCCAGCTAGCATTGCAGCAAGAGGTTACATTTCCACAAAGGTCGAG
CTAGGGGAGGCGGCGGCAATATTCATGACAGCCACCCCACCAGGCACTTCAGATCCATTCCCA
GAGTCCAATTCACCAATTTCCGACTTACAGACTGAGATCCCGGATCGAGCTTGGAACTCTGGA
TACGAATGGATCACAGAATACACCGGGAAGACGGTTTGGTTTGTGCCTAGTGTCAAGATGGG
GAATGAGATTGCCCTTTGCCTACAACGTGCTGGAAAGAAAGTAGTCCAATTGAACAGAAAGT
CGTACGAGACGGAGTACCCAAAATGTAAGAACGATGATTGGGACTTTGTTATCACAACAGAC
ATATCTGAAATGGGGGCTAACTTCAAGGCGAGCAGGGTGATTGACAGCCGGAAGAGTGTGAA
ACCAACCATCATAACAGAAGGAGAAGGGAGAGTGATCCTGGGAGAACCATCTGCAGTGACA
GCAGCTAGTGCCGCCCAGAGACGTGGACGTATCGGTAGAAATCCGTCGCAAGTTGGTGATGA
GTACTGTTATGGGGGGCACACGAATGAAGACGACTCGAACTTCGCCCATTGGACTGAGGCAC
GAATCATGCTGGACAACATCAACATGCCAAACGGACTGATCGCTCAATTCTACCAACCAGAGC
GTGAGAAGGTATATACCATGGATGGGGAATACCGGCTCAGAGGAGAAGAGAGAAAAAACTTT
CTGGAACTGTTGAGGACTGCAGATCTGCCAGTTTGGCTGGCTTACAAGGTTGCAGCGGCTGG
AGTGTCATACCACGACCGGAGGTGGTGCTTTGATGGTCCTAGGACAAACACAATTTTAGAAG
ACAACAACGAAGTGGAAGTCATCACGAAGCTTGGTGAAAGGAAGATTCTGAGGCCGCGCTG
GATTGATGCCAGGGTGTACTCGGATCACCAGGCACTAAAGGCGTTCAAGGACTTCGCCTCGG
GAAAACGTTCTCAGATAGGGCTCATTGAGGTTCTGGGAAAGATGCCTGAGCACTTCATGGGG
AAGACATGGGAAGCACTTGACACCATGTACGTTGTGGCCACTGCAGAGAAAGGAGGAAGAG
CTCACAGAATGGCCCTGGAGGAACTGCCAGATGCTCTTCAGACAATTGCCTTGATTGCCTTAT
TGAGTGTGATGACCATGGGAGTATTCTTCCTCCTCATGCAGCGGAAGGGCATTGGAAAGATAG
GTTTGGGAGGCGCTGTCTTGGGAGTCGCGACCTTTTTCTGTTGGATGGCTGAAGTTCCAGGA
ACGAAGATCGCCGGAATGTTGCTGCTCTCCCTTCTCTTGATGATTGTGCTAATTCCTGAGCCAG
AGAAGCAACGTTCGCAGACAGACAACCAGCTAGCCGTGTTCCTGATTTGTGTCATGACCCTT
GTGAGCGCAGTGGCAGCCAACGAGATGGGTTGGCTAGATAAGACCAAGAGTGACATAAGCA
GTTTGTTTGGGCAAAGAATTGAGGTCAAGGAGAATTTCAGCATGGGAGAGTTTCTTCTGGAC
TTGAGGCCGGCAACAGCCTGGTCACTGTACGCTGTGACAACAGCGGTCCTCACTCCACTGCT
AAAGCATTTGATCACGTCAGATTACATCAACACCTCATTGACCTCAATAAACGTTCAGGCAAG
TGCACTATTCACACTCGCGCGAGGCTTCCCCTTCGTCGATGTTGGAGTGTCGGCTCTCCTGCT
AGCAGCCGGATGCTGGGGACAAGTCACCCTCACCGTTACGGTAACAGCGGCAACACTCCTTT
TTTGCCACTATGCCTACATGGTTCCCGGTTGGCAAGCTGAGGCAATGCGCTCAGCCCAGCGGC
GGACAGCGGCCGGAATCATGAAGAACGCTGTAGTGGATGGCATCGTGGCCACGGACGTCCCA
GAATTAGAGCGCACCACACCCATCATGCAGAAGAAAGTTGGACAGATCATGCTGATCTTGGTG
TCTCTAGCTGCAGTAGTAGTGAACCCGTCTGTGAAGACAGTACGAGAAGCCGGAATTTTGATC
ACGGCCGCAGCGGTGACGCTTTGGGAGAATGGAGCAAGCTCTGTTTGGAACGCAACAACTG
CCATCGGACTCTGCCACATCATGCGTGGGGGTTGGTTGTCATGTCTATCCATAACATGGACACT
CATAAAGAACATGGAAAAACCAGGACTAAAAAGAGGTGGGGCAAAAGGACGCACCTTGGGA
GAGGTTTGGAAAGAAAGACTCAACCAGATGACAAAAGAAGAGTTCACT

File S2 ISA fragments

>wt-NY99_**Fragment-III**
GGTGGGGCAAAAGGACGCACCTTGGGAGAGGTTTGGAAAGAAAGACTCAACCAGATGACAA
AAGAAGAGTTCACTAGGTACCGCAAAGAGGCCATCATCGAAGTCGATCGCTCAGCGGCAAAA
CACGCCAGGAAAGAAGGCAATGTCACTGGAGGGCATCCAGTCTCTAGGGGCACAGCAAAAC
TGAGATGGCTGGTCGAACGGAGGTTTCTCGAACCGGTCGGAAAAGTGATTGACCTTGGATGT
GGAAGAGGCGGTTGGTGTTACTATATGGCAACCCAAAAAAGAGTCCAAGAAGTCAGAGGGTA
CACAAAGGGCGGTCCCGGACATGAAGAGCCCCAACTAGTGCAAAGTTATGGATGGAACATTG
TCACCATGAAGAGTGGAGTGGATGTGTTCTACAGACCTTCTGAGTGTTGTGACACCCTCCTTT
GTGACATCGGAGAGTCCTCGTCAAGTGCTGAGGTTGAAGAGCATAGGACGATTCGGGTCCTT
GAAATGGTTGAGGACTGGCTGCACCGAGGGCCAAGGGAATTTTGCGTGAAGGTGCTCTGTCC
CTACATGCCGAAAGTCATAGAGAAGATGGAGCTGCTCCAACGCCGGTATGGGGGGGACTGG
TCAGAAACCCACTCTCACGGAATTCCACGCACGAGATGTATTGGGTGAGTCGAGCTTCAGGC
AATGTGGTACATTCAGTGAATATGACCAGCCAGGTGCTCCTAGGAAGAATGGAAAAAAGGAC
CTGGAAGGGACCCCAATACGAGGAAGATGTAAACTTGGGAAGTGGAACCAGGGCGGTGGGA
AAACCCCTGCTCAACTCAGACACCAGTAAAATCAAGAACAGGATTGAACGACTCAGGCGTGA
GTACAGTTCGACGTGGCACCACGATGAGAACCACCCATATAGAACCTGGAACTATCACGGCA
GTTATGATGTGAAGCCCACAGGCTCCGCCAGTTCGCTGGTCAATGGAGTGGTCAGGCTCCTCT
CAAAACCATGGGACACCATCACGAATGTTACCACCATGGCCATGACTGACACTACTCCCTTCG
GGCAGCAGCGAGTGTTCAAAGAGAAGGTGGACACGAAAGCTCCTGAACCGCCAGAAGGAG
TGAAGTACGTGCTCAACGAGACCACCAACTGGTTGTGGGCGTTTTTGGCCAGAGAAAAACGT
CCCAGAATGTGCTCTCGAGAGGAATTCATAAGAAAGGTCAACAGCAATGCAGCTTTGGGTGC
CATGTTTGAAGAGCAGAATCAATGGAGGAGCGCCAGAGAAGCAGTTGAAGATCCAAAATTTT
GGGAGATGGTGGATGAGGAGCGCGAGGCACATCTGCGGGGGGAATGTCACACTTGCATTTAC
AACATGATGGGAAAGAGAGAGAAAAAACCCGGAGAGTTCGGAAAGGCCAAGGGAAGCAGA
GCCATTTGGTTCATGTGGCTCGGAGCTCGCTTTCTGGAGTTCGAGGCTCTGGGTTTTCTCAAT
GAAGACCACTGGCTTGGAAGAAAGAACTCAGGAGGAGGTGTCGAGGGCTTGGGCCTCCAAA
AACTGGGTTACATCCTGCGTGAAGTTGGCACCCGGCCTGGGGGCAAGATCTATGCTGATGACA
CAGCTGGCTGGGACACCCGCATCACGAGAGCTGACTTGGAAAATGAAGCTAAGGTGCTTGAG
CTGCTTGATGGGGAACATCGGCGTCTTGCCAGGGCCATCATTGAGCTCACCTATCGTCACAAA
GTTGTGAAAGTGATGCGCCCGGCTGCTGATGGAAGAACCGTCATGGATGTTATCTCCAGAGAA
GATCAGAGGGGGAGTGGACAAGTTGTCACCTACGCCCTAAACACTTTCACCAACCTGGCCGT
CCAGCTGGTGAGGATGATGGAAGGGGAAGGAGTGATTGGCCCAGATGATGTGGAGAAACTC
ACAAAAGGGAAAGGACCCAAAGTCAGGACCTGGCTGTTTGAGAATGGGGAAGAAAGACTCA
GCCGCATGGCTGTCAGTGGAGATGACTGTGTGGTAAAGCCCCTGGACGATCGCTTTGCCACCT
CGCTCCACTTCCTCAATGCTATGTCAAAGGTTCGCAAAGACATCCAAGAGTGGAAACCGTCA
ACTGGATGGTATGATTGGCAGCAGGTTCCATTTTGCTCAAACCATTTCACTGAATTGATCATGA
AAGATGGAAGAACACTGGTGGTTCCATGCCGAGGACAGGATGAATTGGTAGGCAGAGCTCGC
ATATCTCCAGGGGCCGGATGGAACGTCCGCGACACTGCTTGTCTGGCTAAGTCTTATGCCCAG
ATGTGGCTGCTTCTGTACTTCCACAGAAGAGACCTGCGGCTCATGGCCAACGCCATTTGCTCC
GCTGTCCCTGTGAATTGGGTCCCTACCGGAAGAACCACGTGGTCCATCCATGCAGGAGGAGA
GTGGATGACAACAGAGGACATGTTGGAGGTCTGGAACCGTGTTTGGATAGAGGAGAATGAAT
GGATGGAAGACAAAACCCCAGTGGAGAAATGGAGTGACGTCCCATATTCAGGAAAACGAGA
GGACATCTGGTGTGGCAGCCTGATTGGCACAAGAGCCCGAGCCACGTGGGCAGAAAACATCC
AGGTGGCTATCAACCAAGTCAGAGCAATCATCGGAGATGAGAAGTATGTGGACTACATGAGTT
CACTAAAGAGATATGAAGACACAACTTTGGTTGAGGACACAGTACTGTAGATATTTAATCAAT
TGTAAATAGACAATATAAGTATGCATAAAAGTGTAGTTTTATAGTAGTATTTAGTGGTGTTAGTG
TAAATAGTTAAGAAAATTTTGAGGAGAAAGTCAGGCCGGGAAGTTCCCGCCACCGGAAGTTG
AGTAGACGGTGCTGCCTGCGACTCAACCCCAGGAGGACTGGGTGAACAAAGCCGCGAAGTG
ATCCATGTAAGCCCTCAGAACCGTCTCGGAAGGAGGACCCCACATGTTGTAACTTCAAAGCC
CAATGTCAGACCACGCTACGGCGTGCTACTCTGCGGAGAGTGCAGTCTGCGATAGTGCCCCA
GGAGGACTGGGTTAACAAAGGCAAACCAACGCCCCACGCGGCCCTAGCCCCGGTAATGGTGT

File S2 ISA fragments

TAACCAGGGCGAAAGGACTAGAGGTTAGAGGAGACCCCGCGGTTTAAAGTGCACGGCCCAG
CCTGGCTGAAGCTGTAGGTCAGGGGAAGGACTAGAGGTTAGTGGAGACCCCGTGCCACAAA
ACACCACAACAAAACAGCATATTGACACCTGGGATAGACTAGGAGATCTTCTGCTCTGCACAA
CCAGCCACACGGCACAGTGCGCCGACAATGGTGGCTGGTGGTGCGAGAACACAGGATCT**GG
CCGGCATGGTCCCAGCCTCCTCGCTGGCGCCGGCTGGGCAACATTCCGAGGGGACCGT
CCCCTCGGTAATGGCGAATGGGACTCGCGACAGACATGATAAGATACATTGATGAGTTT
GGACAAACCACAACTAGAATGCAGTGAAAAAAATGCTTTATTTGTGAAATTAAGCGCTG
GCATTGACCCTGAG**

File S2 ISA fragments

E-MAX+FR

Highlighted in red – pCMV promoter sequence.
Highlighted in green – HDR/SV40pA sequence.

>wt-NY99-E-MAX-FR_**Fragment-I**
CACCCAACTGATCTTCAGCATCTTCAATATTGGCCATTAGCCATATTATTCATTGGTTATATAGCA
TAAATCAATATTGGCTATTGGCCATTGCATACGTTGTATCTATATCATAATATGTACATTTATATTG
GCTCATGTCCAATATGACCGCCATGTTGGCATTGATTATTGACTAGTTATTAATAGTAATCAATTA
CGGGGTCATTAGTTCATAGCCCATATATGGAGTTCCGCGTTACATAACTTACGGTAAATGGCCC
GCCTGGCTGACCGCCCAACGACCCCCGCCCATTGACGTCAATAATGACGTATGTTCCCATAGT
AACGCCAATAGGGACTTTCCATTGACGTCAATGGGTGGAGTATTTACGGTAAACTGCCCACTT
GGCAGTACATCAAGTGTATCATATGCCAAGTCCGCCCCCTATTGACGTCAATGACGGTAAATGG
CCCGCCTGGCATTATGCCCAGTACATGACCTTACGGGACTTTCCTACTTGGCAGTACATCTACG
TATTAGTCATCGCTATTACCATGGTGATGCGGTTTTGGCAGTACACCAATGGGCGTGGATAGCG
GTTTGACTCACGGGGATTTCCAAGTCTCCACCCCATTGACGTCAATGGGAGTTTGTTTTGGCA
CCAAAATCAACGGGACTTTCCAAAATGTCGTAATAACCCCGCCCCGTTGACGCAAATGGGCG
GTAGGCGTGTACGGTGGGAGGTCTATATAAGCAGAGCTCGTTTAGTGAACCGAGTAGTTCGCC
TGTGTGAGCTGACAAACTTAGTAGTGTTTGTGAGGATTAACAACAATTAACACAGTGCGAGCT
GTTTCTTAGCACGAAGATCTCGATGTCTAAGAAACCAGGAGGGCCCGGCAAGAGCCGGGCTG
TCAATATGCTAAAACGCGGAATGCCCCGCGTGTTGTCCTTGATTGGACTGAAGAGGGCTATGT
TGAGCCTGATCGACGGCAAGGGGCCAATACGATTTGTGTTGGCTCTCTTGGCGTTCTTCAGGT
TCACAGCAATTGCTCCGACCCGAGCAGTGCTGGATCGATGGAGAGGTGTGAACAAACAAACA
GCGATGAAACACCTTCTGAGTTTTAAGAAGGAACTAGGGACCTTGACCAGTGCTATCAATCGG
CGGAGCTCAAAACAAAAGAAAAGAGGAGGAAAGACCGGAATTGCAGTCATGATTGGCCTGA
TCGCCAGCGTAGGAGCAGTTACCCTCTCTAACTTCCAAGGGAAGGTGATGATGACGGTAAATG
CTACTGACGTCACAGATGTCATCACGATTCCAACAGCTGCTGGAAAGAACCTATGCATTGTCA
GAGCAATGGATGTGGGATACATGTGCGATGATACTATCACTTATGAATGCCCAGTACTGTCGGC
TGGTAATGATCCAGAAGACATCGACTGTTGGTGCACAAAGTCAGCAGTCTACGTCAGGTATGG
AAGATGCACCAAGACACGCCACTCAAGACGCAGTCGGAGGTCACTGACAGTGCAGACACAC
GGAGAAAGCACTCTAGCGAACAAGAAGGGGGCTTGGATGGACAGCACCAAGGCCACAAGGT
ATTTGGTAAAAACAGAATCATGGATCTTGAGGAACCCTGGATATGCCCTGGTGGCAGCCGTCA
TTGGTTGGATGCTTGGGAGCAACACCATGCAGAGAGTTGTGTTTGTCGTGCTATTGCTTTTGG
TGGCCCCAGCTTACAGCTTCAACTGCCTTGGAATGAGCAACAGAGACTTTTTGGAAGGCGTT
TCCGGCGCGACGTGGGTCGATTTAGTTCTCGAAGGGGATAGTTGCGTTACGATAATGTCGAAA
GACAAACCGACGATCGACGTTAAAATGATGAATATGGAGGCCGCGAATCTAGCGGAAGTTCG
CAGTTATTGTTATTTAGCAACCGTAAGCGATCTTTCGACGAAAGCCGCGTGTCCGACGATGGG
CGAAGCGCACAACGATAAACGGGCGGATCCCGCGTTTGTTTGTCGACAAGGCGTCGTCGATC
GCGGTTGGGGAAATGGTTGTGGATTTTTCGGCAAAGGAAGTATCGATACGTGCGCGAAATTTG
CGTGTTCTACTAAAGCGATAGGACGAACGATATTAAAAGAAAATATCAAATACGAAGTCGCGA
TTTTTGTCCACGGACCGACGACGGTCGAATCGCACGGAAATTATTCGACGCAAGTCGGCGCG
ACGCAGGCCGGTCGACTAAGTATAACGCCCGCGGCGCCGTCGTACACGCTAAAACTGGGCGA
ATATGGCGAGGTTACGGTCGACTGCGAACCGCGTTCGGGTATCGATACGAACGCGTATTATGTT
ATGACGGTCGGAACGAAAACGTTTTAGTACATCGGGAATGGTTCATGGATCTAAACCTACCG
TGGAGCAGTGCCGGAAGTACCGTTTGGCGTAACCGGGAAACGTTAATGGAATTCGAAGAACC
GCACGCGACTAAACAGTCCGTAATCGCGTTAGGATCGCAAGAAGGCGCGCTTCATCAAGCGT
TAGCCGGCGCGATTCCGGTCGAATTTTCGAGTAATACCGTCAAATTAACATCCGGTCATTTGAA
ATGTCGGGTCAAAATGGAAAAATTACAATTAAAAGGAACGACTTACGGCGTATGTTCGAAAG
CGTTCAAGTTTCTCGGTACACCCGCCGATACCGGTCACGGAACCGTCGTTTTGGAATTACAAT
ATACCGGAACCGATGGACCGTGTAAAGTTCCGATATCGTCCGTGGCGTCGTTGAACGATCTAA
CACCCGTCGGCCGATTAGTCACCGTTAATCCGTTCGTTTCCGTCGCGACGGCGAACGCGAAAG

File S2 ISA fragments

TACTTATAGAATTGGAACCGCCGTTTGGCGATTCGTATATCGTCGTCGGCCGCGGGGAACAAC
AAATAAATCACCATTGGCATAAGTCCGGAAGTAGTATCGGAAAAGCGTTTACGACGACGCTAA
AAGGCGCGCAACGACTAGCCGCGCTCGGCGATACCGCGTGGGATTTCGGATCCGTCGGGGGC
GTTTTTACGTCCGTCGGTCGAGCCGTACATCAAGTATTCGGCGGGGCGTTTCGCTCGCTTTTCG
GCGGAATGTCGTGGATAACGCAAGGATTACTGGGCGCGCTACTTTTATGGATGGGAATAAACG
CGCGGGATCGTTCGATCGCGCTAACGTTTCTCGCCGTCGGCGGAGTTCTGCTCTTCCTCTCCG
TGAACGTGCACGCTGACACTGGGTGTGCCATAGACATCAGCCGGCAAGAGCTGAGATGTGGA
AGTGGAGTGTTCATACACAATGATGTGGAGGCTTGGATGGACCGGTACAAGTATTACCCTGAA
ACGCCACAAGGCCTAGCCAAGATCATTCAGAAAGCTCATAAGGAAGGAGTGTGCGGTCTACG
ATCAGTTTCCAGACTGGAGCATCAAATGTGGGAAGCAGTGAAGGACGAGCTGAACACTCTTT
TGAAGGAGAATGGTGTGGACCTTAGTGTCGTGGTTGAGAAACAGGAGGGAATGTACAAGTCA
GCACCTAAACGCCTCACCGCCACCACGGAAAAATTGGAAATTGGCTGGAAGGCCTGGGGAA
AGAGTATTTTATTTGCACCAGAACTCGCCAACAACACCTTTGTGGTTGATGGTCCGGAGACCA
AGGAATGTCCGACTCAGAATCGCGCTTGGAATAGCTTAGAAGTGGAGGATTTTGGATTTGGTC
TCACCAGCACTCGGATGTTCCTGAAGGTCAGAGAGAGCAACACAACTGAATGTGACTCGAAG
ATCATTGGAACGGCTGTCAAGAACAACTTGGCGATCCACAGTGACCTGTCCTATTGGATTGAA
AGCAGGCTCAATGATACGTGGAAGCTTGAAAGGGCAGTTCTGGGTGAAGTCAAATCATGTAC
GTGGCCTGAGACGCATACCTTGTGGGGCGATGGAATCCTTGAGAGTGACTTGATAATACCAGT
CACACTGGCGGGACCACGAAGCAATCACAATCGGAGACCTGGGTACAAGACACAAAACCAG
GGCCCATGGGACGAAGGCCGGGTAGAGATTGACTTCGATTACTGCCCAGGAACTACGGTCAC
CCTGAGTGAGAGCTGCGGACACCGTGGACCTGCCACTCGCACCACCACAGAGAGCGGAAAG
TTGATAACAGATTGGTGCTGCAGGAGCTGCACCTTACCACCACTGCGCTACCAAACTGACAGC
GGCTGTTGGTATGGTATGGAGATCAGACCACAGAGACATGATGAAAAGACCCTCGTGCAGTC
ACAAGTGAATGCTTATAATGCTGATATGATTGACCCTTTTCAGTTGGGCCTTCTGGTCGTGTTC
TTGGCCACCCAGGAGGTCCTTCGC

>wt-NY99_**Fragment-II**
TATAATGCTGATATGATTGACCCTTTTCAGTTGGGCCTTCTGGTCGTGTTCTTGGCCACCCAGG
AGGTCCTTCGCAAGAGGTGGACAGCCAAGATCAGCATGCCAGCTATACTGATTGCTCTGCTAG
TCCTGGTGTTTGGGGGCATTACTTACACTGATGTGTTACGCTATGTCATCTTGGTGGGGGCAGC
TTTCGCAGAATCTAATTCGGGAGGAGACGTGGTACACTTGGCGCTCATGGCGACCTTCAAGAT
ACAACCAGTGTTTATGGTGGCATCGTTTCTCAAAGCGAGATGGACCAACCAGGAGAACATTTT
GTTGATGTTGGCGGCTGTTTTCTTTCAAATGGCTTATCACGATGCCCGCCAAATTCTGCTCTGG
GAGATCCCTGATGTGTTGAATTCACTGGCGGTAGCTTGGATGATACTGAGAGCCATAACATTCA
CAACGACATCAAACGTGGTTGTTCCGCTGCTAGCCCTGCTAACACCCGGGCTGAGATGCTTGA
ATCTGGATGTGTACAGGATACTGCTGTTGATGGTCGGAATAGGCAGCTTGATCAGGGAGAAGA
GGAGTGCAGCCGCAAAAAAGAAAGGAGCAAGTCTGCTATGCTTGGCTCTAGCCTCAACAGG
ACTTTTCAACCCCATGATCCTTGCTGCTGGACTGATTGCATGTGATCCCAACCGTAAACGCGG
ATGGCCCGCAACTGAAGTGATGACAGCTGTCGGCCTAATGTTTGCCATCGTCGGAGGGCTGGC
AGAGCTTGACATTGACTCCATGGCCATTCCAATGACTATCGCGGGGCTCATGTTTGCTGCTTTC
GTGATTTCTGGGAAATCAACAGATATGTGGATTGAGAGAACGGCGGACATTTCCTGGGAAAGT
GATGCAGAAATTACAGGCTCGAGCGAAAGAGTTGATGTGCGGCTTGATGATGATGGAAACTT
CCAGCTCATGAATGATCCAGGAGCACCTTGGAAGATATGGATGCTCAGAATGGTCTGTCTCGC
GATTAGTGCGTACACCCCCTGGGCAATCTTGCCCTCAGTAGTTGGATTTTGGATAACTCTCCAA
TACACAAAGAGAGGAGGCGTGTTGTGGGACACTCCCTCACCAAAGGAGTACAAAAAGGGGG
ACACGACCACCGGCGTCTACAGGATCATGACTCGTGGGCTGCTCGGCAGTTATCAAGCAGGA
GCGGGCGTGATGGTTGAAGGTGTTTTCCACACCCTTTGGCATACAACAAAAGGAGCCGCTTT
GATGAGCGGAGAGGGCCGCCTGGACCCATACTGGGGCAGTGTCAAGGAGGATCGACTTTGTT
ACGGAGGACCCTGGAAATTGCAGCACAAGTGGAACGGGCAGGATGAGGTGCAGATGATTGT

File S2 ISA fragments

GGTGGAACCTGGCAAGAACGTTAAGAACGTCCAGACGAAACCAGGGGTGTTCAAAACACCT
GAAGGAGAAATCGGGGCCGTGACTTTGGACTTCCCCACTGGAACATCAGGCTCACCAATAGT
GGACAAAAACGGTGATGTGATTGGGCTTTATGGCAATGGAGTCATAATGCCCAACGGCTCATA
CATAAGCGCGATAGTGCAGGGTGAAAGGATGGATGAGCCAATCCCAGCCGGATTCGAACCTG
AGATGCTGAGGAAAAAACAGATCACTGTACTGGATCTCCATCCCGGCGCCGGTAAAACAAGG
AGGATTCTGCCACAGATCATCAAAGAGGCCATAAACAGAAGACTGAGAACAGCCGTGCTAGC
GCCAACCAGGGTTGTGGCTGCTGAGATGGCTGAAGCACTGAGAGGACTGCCCATCCGGTACC
AGACATCCGCAGTGCCCAGAGAACATAATGGAAATGAGATTGTTGATGTCATGTGTCATGCTA
CCCTCACCCACAGGCTGATGTCTCCTCACAGGGTGCCGAACTACAACCTGTTCGTGATGGATG
AGGCTCATTTCACCGACCCAGCTAGCATTGCAGCAAGAGGTTACATTTCCACAAAGGTCGAG
CTAGGGGAGGCGGCGGCAATATTCATGACAGCCACCCCACCAGGCACTTCAGATCCATTCCCA
GAGTCCAATTCACCAATTTCCGACTTACAGACTGAGATCCCGGATCGAGCTTGGAACTCTGGA
TACGAATGGATCACAGAATACACCGGGAAGACGGTTTGGTTTGTGCCTAGTGTCAAGATGGG
GAATGAGATTGCCCTTTGCCTACAACGTGCTGGAAAGAAAGTAGTCCAATTGAACAGAAAGT
CGTACGAGACGGAGTACCCAAAATGTAAGAACGATGATTGGGACTTTGTTATCACAACAGAC
ATATCTGAAATGGGGGCTAACTTCAAGGCGAGCAGGGTGATTGACAGCCGGAAGAGTGTGAA
ACCAACCATCATAACAGAAGGAGAAGGGAGAGTGATCCTGGGAGAACCATCTGCAGTGACA
GCAGCTAGTGCCGCCCAGAGACGTGGACGTATCGGTAGAAATCCGTCGCAAGTTGGTGATGA
GTACTGTTATGGGGGGCACACGAATGAAGACGACTCGAACTTCGCCCATTGGACTGAGGCAC
GAATCATGCTGGACAACATCAACATGCCAAACGGACTGATCGCTCAATTCTACCAACCAGAGC
GTGAGAAGGTATATACCATGGATGGGGAATACCGGCTCAGAGGAGAAGAGAGAAAAAACTTT
CTGGAACTGTTGAGGACTGCAGATCTGCCAGTTTGGCTGGCTTACAAGGTTGCAGCGGCTGG
AGTGTCATACCACGACCGGAGGTGGTGCTTTGATGGTCCTAGGACAAACACAATTTTAGAAG
ACAACAACGAAGTGGAAGTCATCACGAAGCTTGGTGAAAGGAAGATTCTGAGGCCGCGCTG
GATTGATGCCAGGGTGTACTCGGATCACCAGGCACTAAAGGCGTTCAAGGACTTCGCCTCGG
GAAAACGTTCTCAGATAGGGCTCATTGAGGTTCTGGGAAAGATGCCTGAGCACTTCATGGGG
AAGACATGGGAAGCACTTGACACCATGTACGTTGTGGCCACTGCAGAGAAAGGAGGAAGAG
CTCACAGAATGGCCCTGGAGGAACTGCCAGATGCTCTTCAGACAATTGCCTTGATTGCCTTAT
TGAGTGTGATGACCATGGGAGTATTCTTCCTCCTCATGCAGCGGAAGGGCATTGGAAAGATAG
GTTTGGGAGGCGCTGTCTTGGGAGTCGCGACCTTTTTCTGTTGGATGGCTGAAGTTCCAGGA
ACGAAGATCGCCGGAATGTTGCTGCTCTCCCTTCTCTTGATGATTGTGCTAATTCCTGAGCCAG
AGAAGCAACGTTCGCAGACAGACAACCAGCTAGCCGTGTTCCTGATTTGTGTCATGACCCTT
GTGAGCGCAGTGGCAGCCAACGAGATGGGTTGGCTAGATAAGACCAAGAGTGACATAAGCA
GTTTGTTTGGGCAAAGAATTGAGGTCAAGGAGAATTTCAGCATGGGAGAGTTTCTTCTGGAC
TTGAGGCCGGCAACAGCCTGGTCACTGTACGCTGTGACAACAGCGGTCCTCACTCCACTGCT
AAAGCATTTGATCACGTCAGATTACATCAACACCTCATTGACCTCAATAAACGTTCAGGCAAG
TGCACTATTCACACTCGCGCGAGGCTTCCCCTTCGTCGATGTTGGAGTGTCGGCTCTCCTGCT
AGCAGCCGGATGCTGGGGACAAGTCACCCTCACCGTTACGGTAACAGCGGCAACACTCCTTT
TTTGCCACTATGCCTACATGGTTCCCGGTTGGCAAGCTGAGGCAATGCGCTCAGCCCAGCGGC
GGACAGCGGCCGGAATCATGAAGAACGCTGTAGTGGATGGCATCGTGGCCACGGACGTCCCA
GAATTAGAGCGCACCACACCCATCATGCAGAAGAAAGTTGGACAGATCATGCTGATCTTGGTG
TCTCTAGCTGCAGTAGTAGTGAACCCGTCTGTGAAGACAGTACGAGAAGCCGGAATTTTGATC
ACGGCCGCAGCGGTGACGCTTTGGGAGAATGGAGCAAGCTCTGTTTGGAACGCAACAACTG
CCATCGGACTCTGCCACATCATGCGTGGGGGTTGGTTGTCATGTCTATCCATAACATGGACACT
CATAAAGAACATGGAAAAACCAGGACTAAAAAGAGGTGGGGCAAAAGGACGCACCTTGGGA
GAGGTTTGGAAAGAAAGACTCAACCAGATGACAAAAGAAGAGTTCACT

File S2 ISA fragments

>wt-NY99_**Fragment-III**
GGTGGGGCAAAAGGACGCACCTTGGGAGAGGTTTGGAAAGAAAGACTCAACCAGATGACAA
AAGAAGAGTTCACTAGGTACCGCAAAGAGGCCATCATCGAAGTCGATCGCTCAGCGGCAAAA
CACGCCAGGAAAGAAGGCAATGTCACTGGAGGGCATCCAGTCTCTAGGGGCACAGCAAAAC
TGAGATGGCTGGTCGAACGGAGGTTTCTCGAACCGGTCGGAAAAGTGATTGACCTTGGATGT
GGAAGAGGCGGTTGGTGTTACTATATGGCAACCCAAAAAAGAGTCCAAGAAGTCAGAGGGTA
CACAAAGGGCGGTCCCGGACATGAAGAGCCCCAACTAGTGCAAAGTTATGGATGGAACATTG
TCACCATGAAGAGTGGAGTGGATGTGTTCTACAGACCTTCTGAGTGTTGTGACACCCTCCTTT
GTGACATCGGAGAGTCCTCGTCAAGTGCTGAGGTTGAAGAGCATAGGACGATTCGGGTCCTT
GAAATGGTTGAGGACTGGCTGCACCGAGGGCCAAGGGAATTTTGCGTGAAGGTGCTCTGTCC
CTACATGCCGAAAGTCATAGAGAAGATGGAGCTGCTCCAACGCCGGTATGGGGGGGACTGG
TCAGAAACCCACTCTCACGGAATTCCACGCACGAGATGTATTGGGTGAGTCGAGCTTCAGGC
AATGTGGTACATTCAGTGAATATGACCAGCCAGGTGCTCCTAGGAAGAATGGAAAAAAGGAC
CTGGAAGGGACCCCAATACGAGGAAGATGTAAACTTGGGAAGTGGAACCAGGGCGGTGGGA
AAACCCCTGCTCAACTCAGACACCAGTAAAATCAAGAACAGGATTGAACGACTCAGGCGTGA
GTACAGTTCGACGTGGCACCACGATGAGAACCACCCATATAGAACCTGGAACTATCACGGCA
GTTATGATGTGAAGCCCACAGGCTCCGCCAGTTCGCTGGTCAATGGAGTGGTCAGGCTCCTCT
CAAAACCATGGGACACCATCACGAATGTTACCACCATGGCCATGACTGACACTACTCCCTTCG
GGCAGCAGCGAGTGTTCAAAGAGAAGGTGGACACGAAAGCTCCTGAACCGCCAGAAGGAG
TGAAGTACGTGCTCAACGAGACCACCAACTGGTTGTGGGCGTTTTTGGCCAGAGAAAAACGT
CCCAGAATGTGCTCTCGAGAGGAATTCATAAGAAAGGTCAACAGCAATGCAGCTTTGGGTGC
CATGTTTGAAGAGCAGAATCAATGGAGGAGCGCCAGAGAAGCAGTTGAAGATCCAAAATTTT
GGGAGATGGTGGATGAGGAGCGCGAGGCACATCTGCGGGGGGAATGTCACACTTGCATTTAC
AACATGATGGGAAAGAGAGAGAAAAAACCCGGAGAGTTCGGAAAGGCCAAGGGAAGCAGA
GCCATTTGGTTCATGTGGCTCGGAGCTCGCTTTCTGGAGTTCGAGGCTCTGGGTTTTCTCAAT
GAAGACCACTGGCTTGGAAGAAAGAACTCAGGAGGAGGTGTCGAGGGCTTGGGCCTCCAAA
AACTGGGTTACATCCTGCGTGAAGTTGGCACCCGGCCTGGGGGCAAGATCTATGCTGATGACA
CAGCTGGCTGGGACACCCGCATCACGAGAGCTGACTTGGAAAATGAAGCTAAGGTGCTTGAG
CTGCTTGATGGGGAACATCGGCGTCTTGCCAGGGCCATCATTGAGCTCACCTATCGTCACAAA
GTTGTGAAAGTGATGCGCCCGGCTGCTGATGGAAGAACCGTCATGGATGTTATCTCCAGAGAA
GATCAGAGGGGGAGTGGACAAGTTGTCACCTACGCCCTAAACACTTTCACCAACCTGGCCGT
CCAGCTGGTGAGGATGATGGAAGGGGAAGGAGTGATTGGCCCAGATGATGTGGAGAAACTC
ACAAAAGGGAAAGGACCCAAAGTCAGGACCTGGCTGTTTGAGAATGGGGAAGAAAGACTCA
GCCGCATGGCTGTCAGTGGAGATGACTGTGTGGTAAAGCCCCTGGACGATCGCTTTGCCACCT
CGCTCCACTTCCTCAATGCTATGTCAAAGGTTCGCAAAGACATCCAAGAGTGGAAACCGTCA
ACTGGATGGTATGATTGGCAGCAGGTTCCATTTTGCTCAAACCATTTCACTGAATTGATCATGA
AAGATGGAAGAACACTGGTGGTTCCATGCCGAGGACAGGATGAATTGGTAGGCAGAGCTCGC
ATATCTCCAGGGGCCGGATGGAACGTCCGCGACACTGCTTGTCTGGCTAAGTCTTATGCCCAG
ATGTGGCTGCTTCTGTACTTCCACAGAAGAGACCTGCGGCTCATGGCCAACGCCATTTGCTCC
GCTGTCCCTGTGAATTGGGTCCCTACCGGAAGAACCACGTGGTCCATCCATGCAGGAGGAGA
GTGGATGACAACAGAGGACATGTTGGAGGTCTGGAACCGTGTTTGGATAGAGGAGAATGAAT
GGATGGAAGACAAAACCCCAGTGGAGAAATGGAGTGACGTCCCATATTCAGGAAAACGAGA
GGACATCTGGTGTGGCAGCCTGATTGGCACAAGAGCCCGAGCCACGTGGGCAGAAAACATCC
AGGTGGCTATCAACCAAGTCAGAGCAATCATCGGAGATGAGAAGTATGTGGACTACATGAGTT
CACTAAAGAGATATGAAGACACAACTTTGGTTGAGGACACAGTACTGTAGATATTTAATCAAT
TGTAAATAGACAATATAAGTATGCATAAAAGTGTAGTTTTATAGTAGTATTTAGTGGTGTTAGTG
TAAATAGTTAAGAAAATTTTGAGGAGAAAGTCAGGCCGGGAAGTTCCCGCCACCGGAAGTTG
AGTAGACGGTGCTGCCTGCGACTCAACCCCAGGAGGACTGGGTGAACAAAGCCGCGAAGTG
ATCCATGTAAGCCCTCAGAACCGTCTCGGAAGGAGGACCCCACATGTTGTAACTTCAAAGCC
CAATGTCAGACCACGCTACGGCGTGCTACTCTGCGGAGAGTGCAGTCTGCGATAGTGCCCCA
GGAGGACTGGGTTAACAAAGGCAAACCAACGCCCCACGCGGCCCTAGCCCCGGTAATGGTGT

File S2 ISA fragments

TAACCAGGGCGAAAGGACTAGAGGTTAGAGGAGACCCCGCGGTTTAAAGTGCACGGCCCAG
CCTGGCTGAAGCTGTAGGTCAGGGGAAGGACTAGAGGTTAGTGGAGACCCCGTGCCACAAA
ACACCACAACAAAACAGCATATTGACACCTGGGATAGACTAGGAGATCTTCTGCTCTGCACAA
CCAGCCACACGGCACAGTGCGCCGACAATGGTGGCTGGTGGTGCGAGAACACAGGATCT**GG**
CCGGCATGGTCCCAGCCTCCTCGCTGGCGCCGGCTGGGCAACATTCCGAGGGGACCGT
CCCCTCGGTAATGGCGAATGGGACTCGCGACAGACATGATAAGATACATTGATGAGTTT
GGACAAACCACAACTAGAATGCAGTGAAAAAAATGCTTTATTTGTGAAATTAAGCGCTG
GCATTGACCCTGAG
