# Supplementary material for: West Nile virus vaccine candidates attenuated by dinucleotide enrichment are immunogenic and protective against lethal infection
Source: PLoS Pathog. 2025 Oct 3;21(10):e1013560. doi: 10.1371/journal.ppat.1013560 (PMC12513643; doi:10.1371/journal.ppat.1013560)
Supplement: S3 File — (PDF) [file ppat.1013560.s006.pdf]

File S3 WNV RNA copy numbers, infectious titers, and viral RNA to infectious titer ratio

WNV variant RNA copy numbers and infectious titers (Figure 4 in the manuscript):

**WNV variants**	**Log$_{10}$ WNV RNA copies/ mouse**	**VERO-ZAP-WT Log$_{10}$ TCID$_{50}$ titers/ mouse (RNA/infectious titer ratio*)**	**VERO-ZAP-KO Log$_{10}$ TCID$_{50}$ titers/ mouse (RNA/infectious titer ratio*)**	**Injection route**
WNV-WT	**7**	5 (100)	5.4 (39.8)	IP
E/NS1-Per	**7**	4.4 (398)	5 (100)	IP
E/NS1/NS5-Per	**7**	5.8 (15.8)	5.9 (12.6)	IP
E+CG	**7**	4.6 (251)	5 (100)	IP
E/NS1+CG	**7**	4.4 (398)	5 (100)	IP
E/NS1/NS5+CG	**7**	3.8 (1,585)	6.3 (5)	IP
E-MAX	**7**	4.4 (398)	5.8 (16)	IP
E+UA	**7**	5.4 (39.8)	5.5 (31.6)	IP
E-MAX/NS5+CG	**7**	2.7 (19,952)	4.5 (316)	IP
E-MAX/NS5-MAX	**7**	<lod (173,768,335)	4.3 (501)	IP
**WNV-WT (Challenge)**	**10**	**8 (100)**	**8.4 (39.8)**	**IP Challenge**

***Values in brackets indicate the viral RNA copy-to-infectious titer ratio (Fig 1D, E) for the corresponding WNV variant stocks listed in the first column.**

WNV variant RNA copy numbers and infectious titers (Figure 9 in the manuscript):

**WNV variants**	**Log$_{10}$ WNV RNA copies/ mouse**	**VERO-ZAP-WT Log$_{10}$ TCID$_{50}$ titers/ mouse (RNA/infectious titer ratio*)**	**VERO-ZAP-KO Log$_{10}$ TCID$_{50}$ titers/ mouse (RNA/infectious titer ratio*)**	**Injection route (Figures)**
WNV-WT	**5**	3 (100)	3.4 (39.8)	footpad
	**7**	5 (100)	5.4 (39.8)	footpad
E-MAX	**5**	2.4 (398)	3.8 (16)	footpad
	**7**	4.4 (398)	5.8 (16)	footpad
	**8**	5.4 (398)	6.8 (16)	footpad
**WNV-WT (Challenge)**	**10**	**8 (100)**	**8.4 (39.8)**	**IP Challenge**

***Values in brackets indicate the viral RNA copy-to-infectious titer ratio (Fig 1D, E) for the corresponding WNV variant stocks listed in the first column.**

WNV variant RNA copy numbers and infectious titers (Figure 10 in the manuscript):

**WNV variants**	**Log$_{10}$ WNV RNA copies/ mouse**	**VERO-ZAP-WT Log$_{10}$ TCID$_{50}$ titers/ mouse (RNA/infectious titer ratio*)**	**VERO-ZAP-KO Log$_{10}$ TCID$_{50}$ titers/ mouse (RNA/infectious titer ratio*)**	**Injection route (Figures)**
WNV-WT+FR	**8**	7 (10)	7 (10)	footpad
E-MAX+FR	**8**	6 (100)	7 (10)	footpad
**WNV-WT (Challenge)**	**10**	**8 (100)**	**8.4 (39.8)**	**IP Challenge**

***Values in brackets indicate the viral RNA copy-to-infectious titer ratio (Fig 1D, E) for the corresponding WNV variant stocks listed in the first column.**
