# Supplementary material for: West Nile virus vaccine candidates attenuated by dinucleotide enrichment are immunogenic and protective against lethal infection
Source: PLoS Pathog. 2025 Oct 3;21(10):e1013560. doi: 10.1371/journal.ppat.1013560 (PMC12513643; doi:10.1371/journal.ppat.1013560)

File S4 Raw Western blot images of ZAP, DDX50, RIG I in Huh7 and Vero cells following infection with wild-type and dinucleotide-enriched WNV variants.

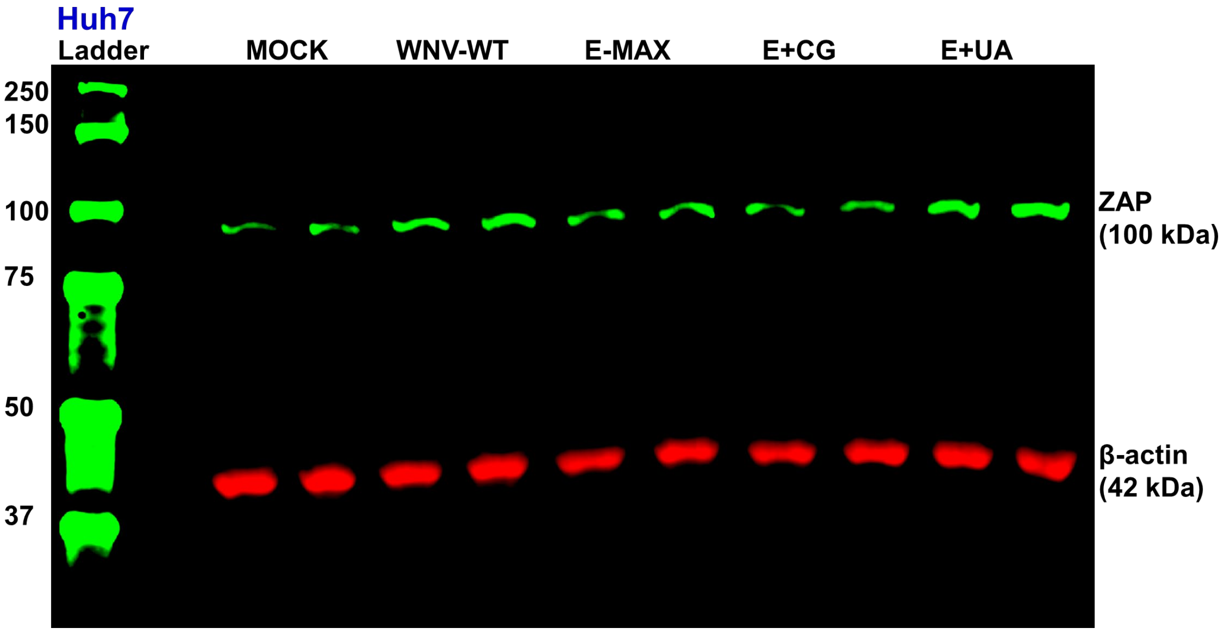

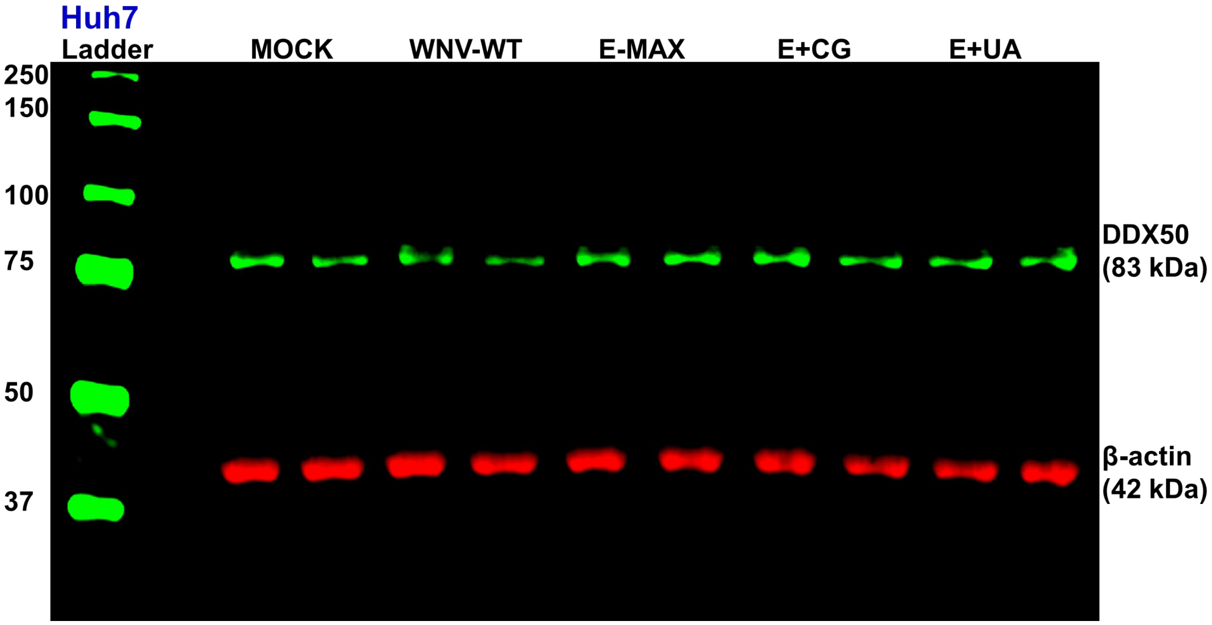

File S4 Raw Western blot images of ZAP, DDX50, RIG I in Huh7 and Vero cells following infection with wild-type and dinucleotide-enriched WNV variants.

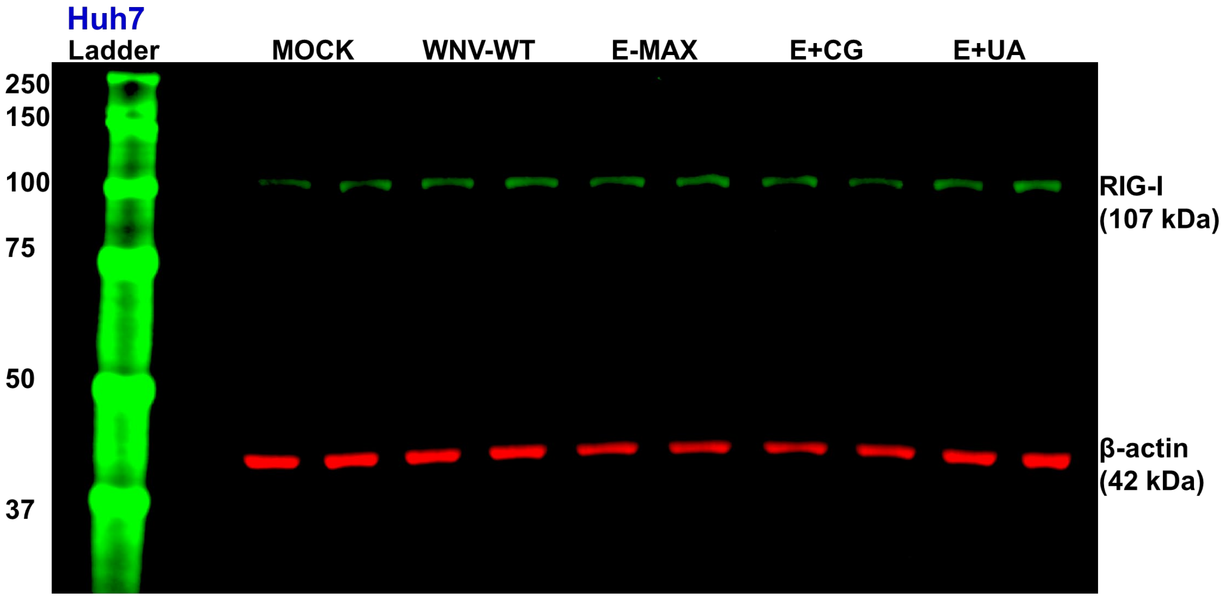

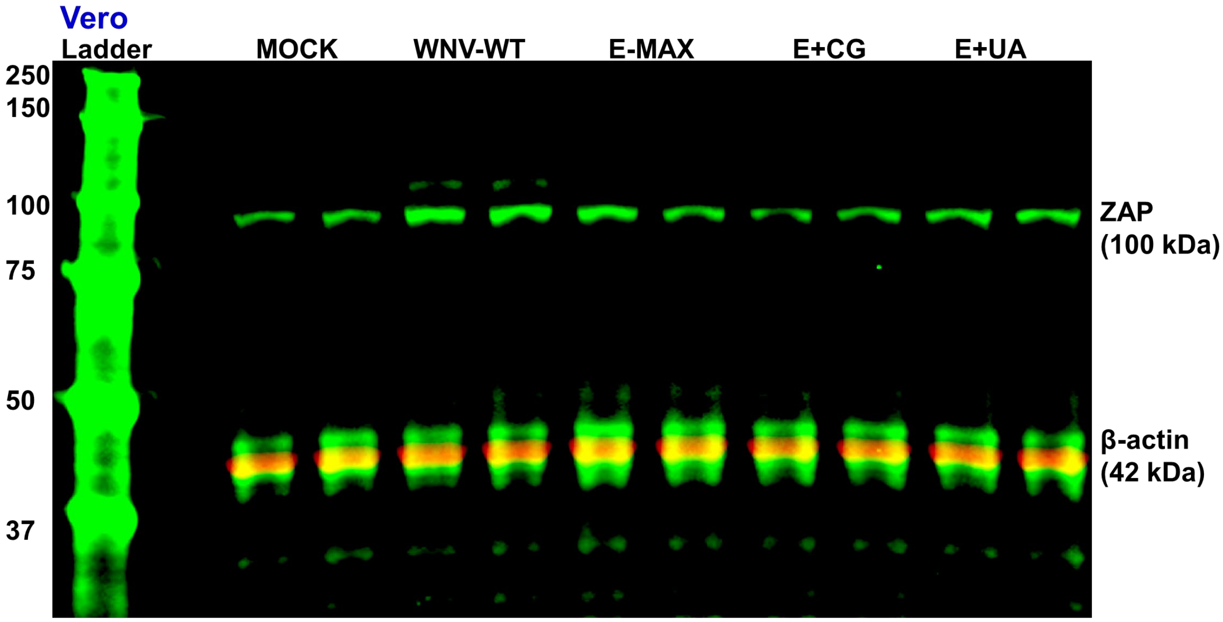

File S4 Raw Western blot images of ZAP, DDX50, RIG I in Huh7 and Vero cells following infection with wild-type and dinucleotide-enriched WNV variants.

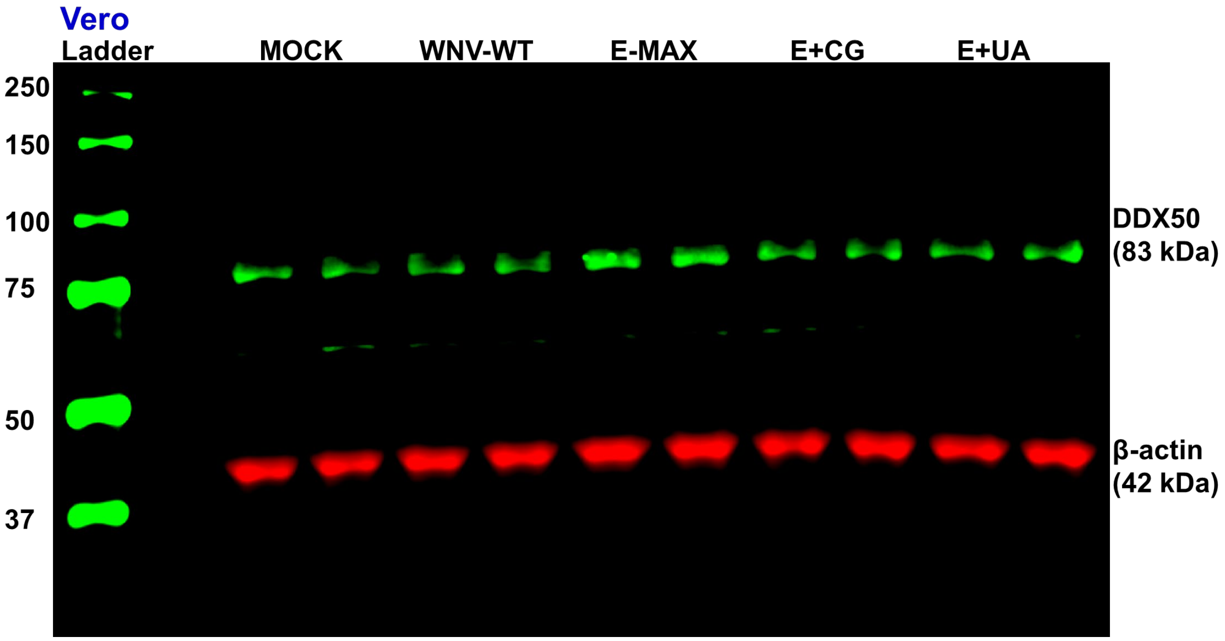

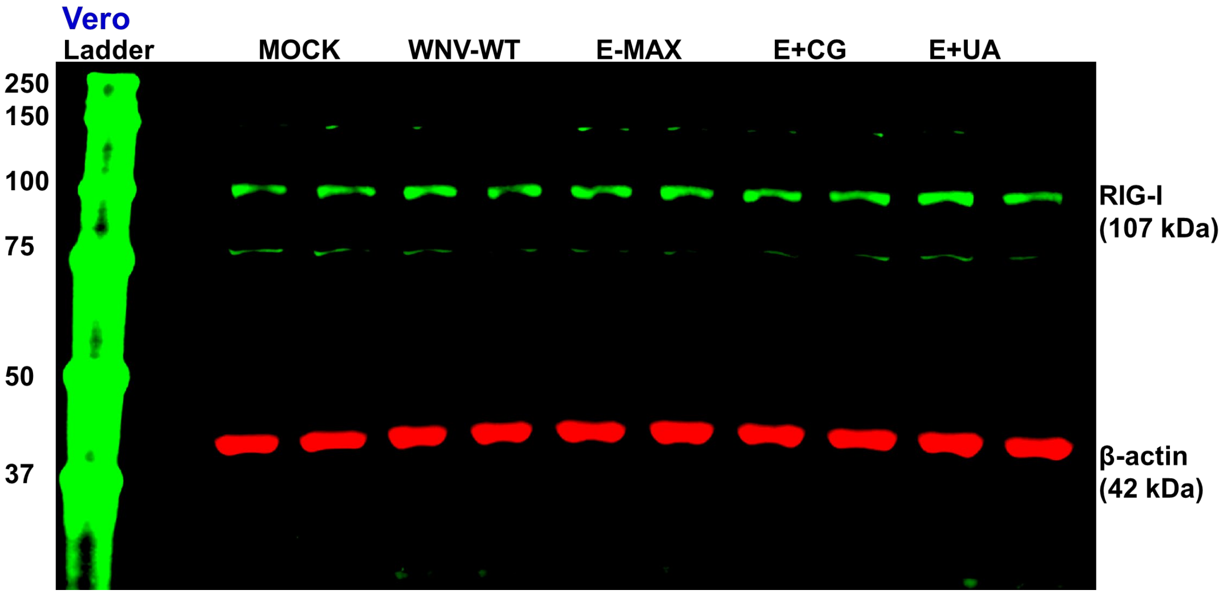

Supplement: S4 File — Raw Western blot images of ZAP, DDX50, RIG-I in Huh7 and Vero cells following infection with wild-type and dinucleotide-enriched WNV variants. Human HuH-7 and monkey Vero cells were inoculated with 1,000 RNA genome copies/cell of WNV-WT, E-MAX, E+ CG, E+ UA, or MOCK. Cells were washed and lysed at 6 h post-inoculation for Western blot. Green bands indicate target proteins of interest: ZAP (100 kDa; multiple bands may represent the four isoforms described for human ZAP, which are still not experimentally characterized in monkeys), DDX50 (83 kDa), and RIG-I (107 kDa). Red bands represent β-actin internal loading control (42 kDa). Western blot was performed in biological duplicate for each experimental condition. (PDF) [file ppat.1013560.s007.pdf]
