# Supplementary material for: West Nile virus vaccine candidates attenuated by dinucleotide enrichment are immunogenic and protective against lethal infection
Source: PLoS Pathog. 2025 Oct 3;21(10):e1013560. doi: 10.1371/journal.ppat.1013560 (PMC12513643; doi:10.1371/journal.ppat.1013560)

File. S5 West Nile virus NGS coverage

Data for Fig 2 in manuscript

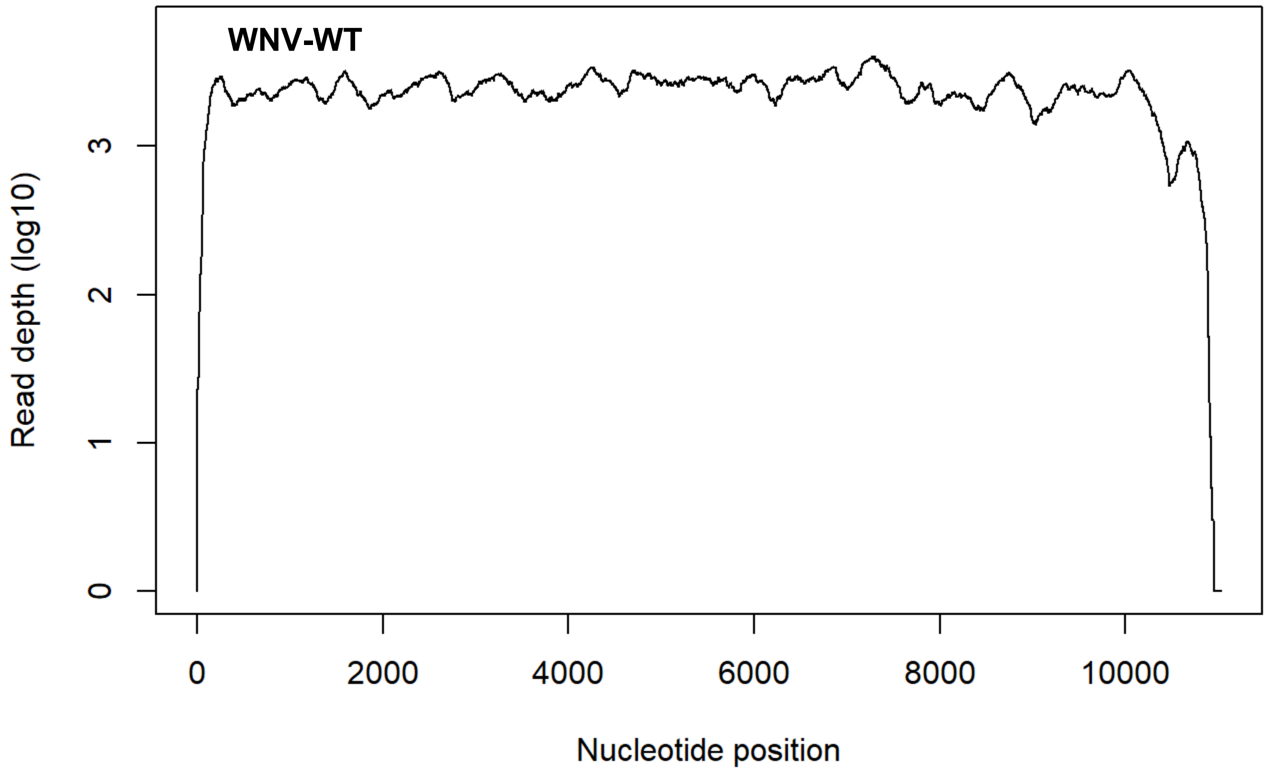

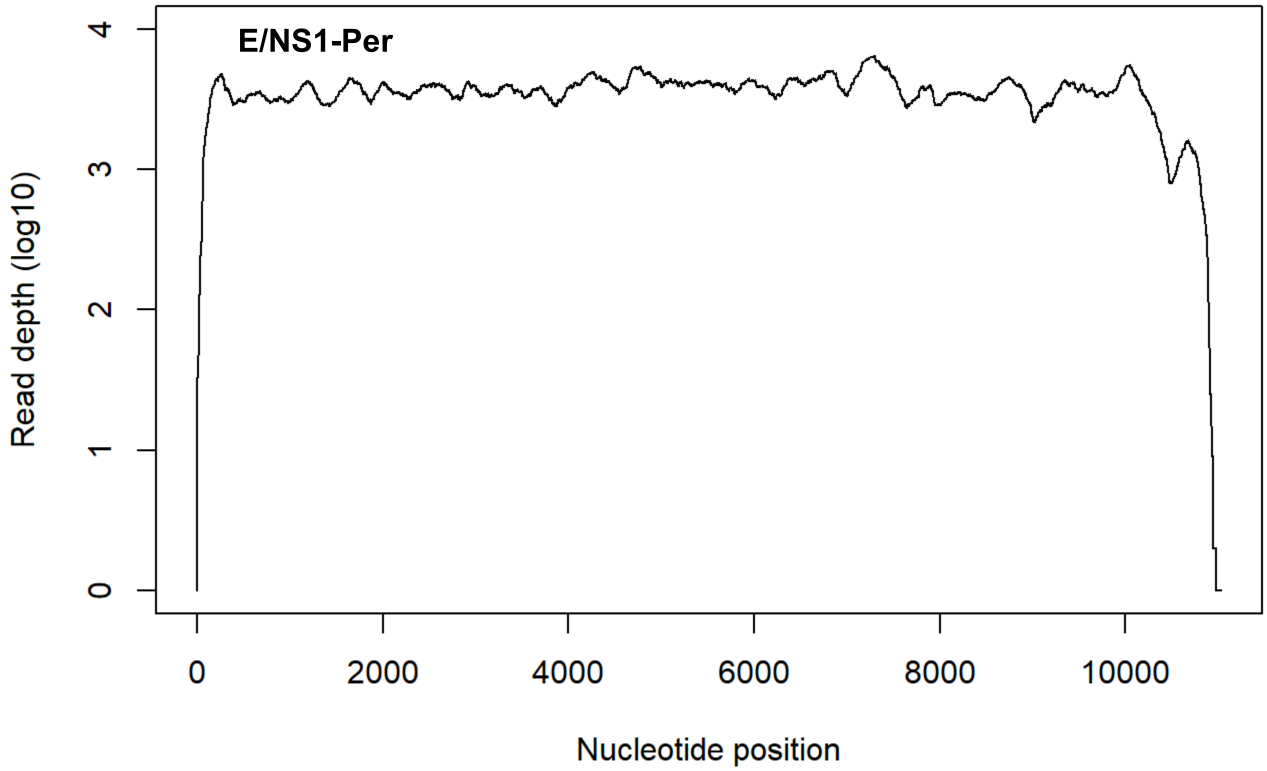
E/NS1-Per
Read depth (log10)
0
1
2
3
4
0
2000
4000
6000
8000
10000
Nucleotide position

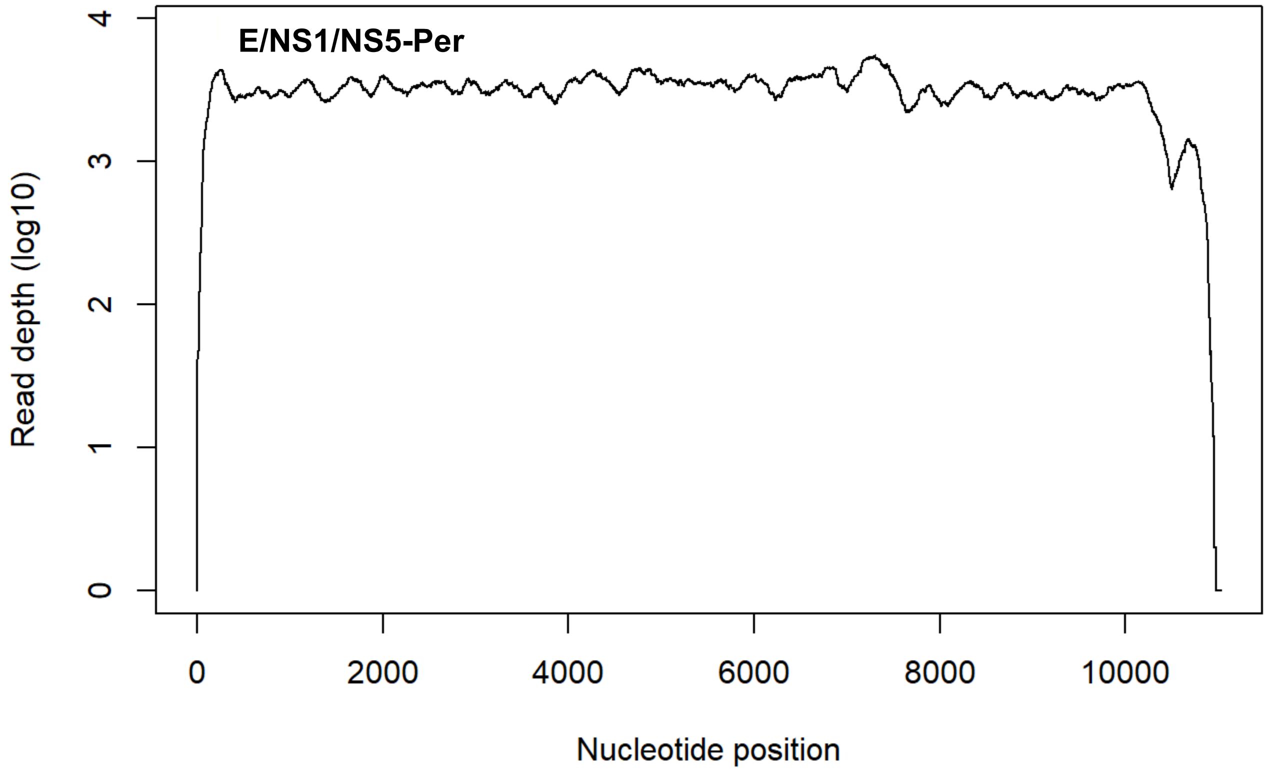
E/NS1/NS5-Per
Read depth (log10)
0
1
2
3
4
0
2000
4000
6000
8000
10000
Nucleotide position

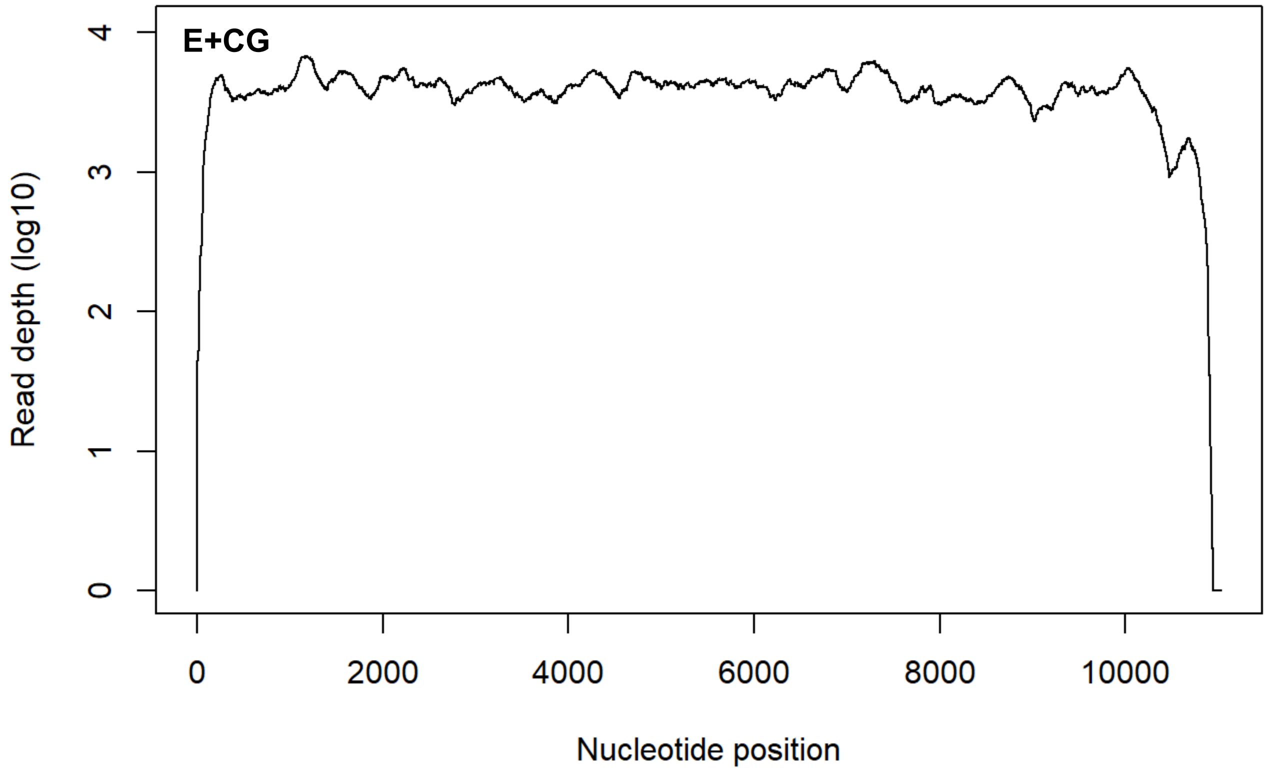
E+CG
Read depth (log10)
0
1
2
3
4
0
2000
4000
6000
8000
10000
Nucleotide position

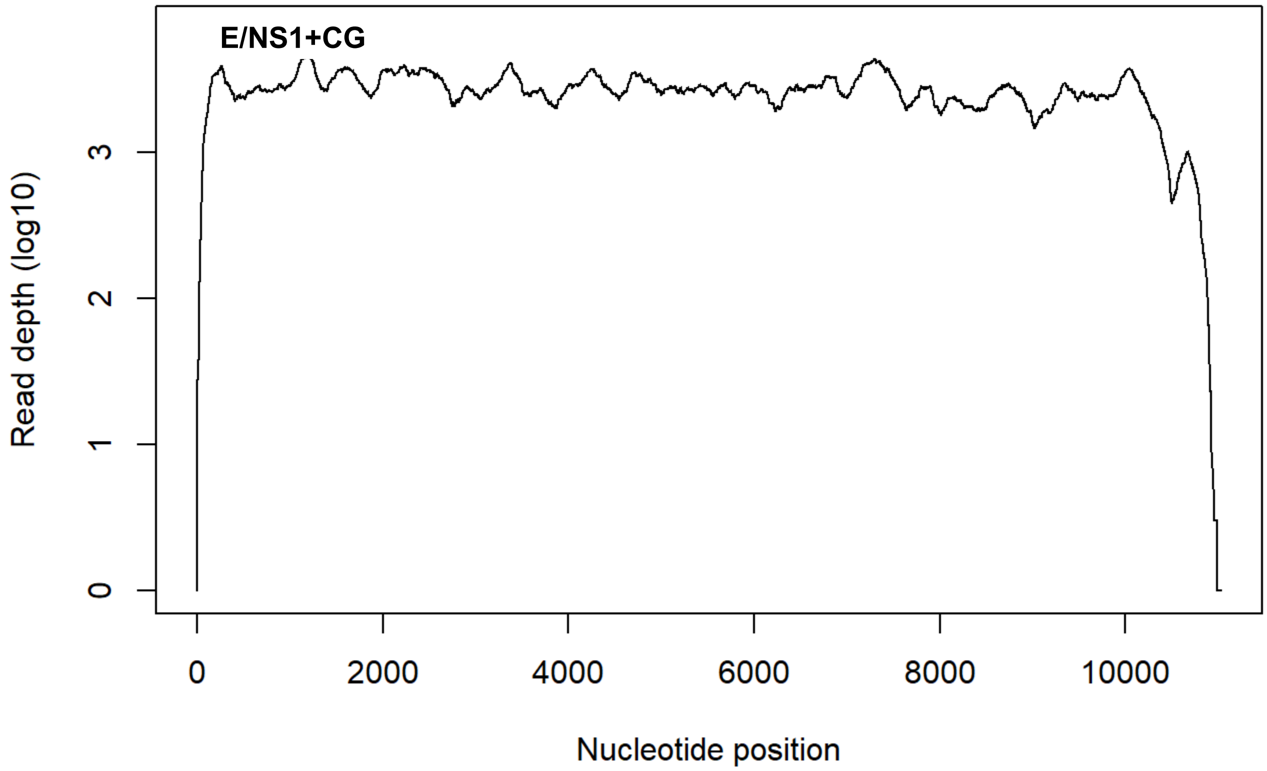
E/NS1+CG
Read depth (log10)
0
1
2
3
0
2000
4000
6000
8000
10000
Nucleotide position

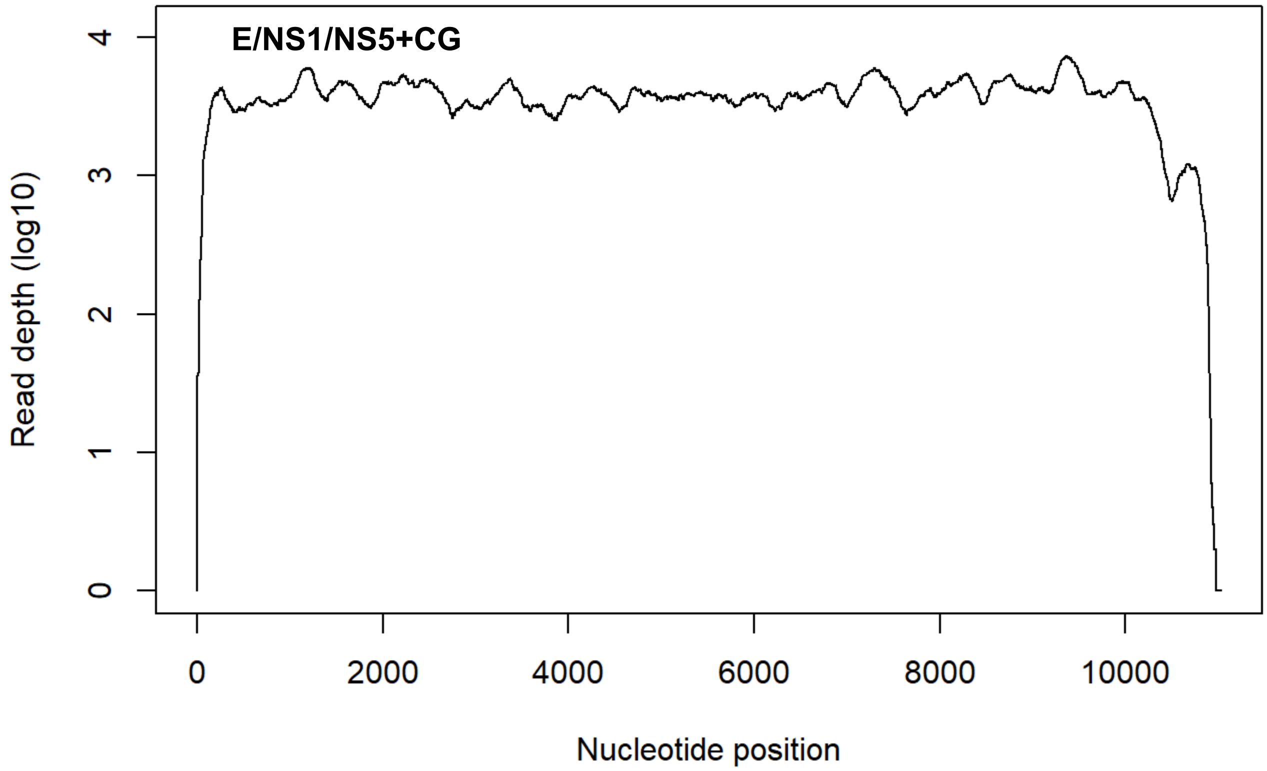
E/NS1/NS5+CG
Read depth (log10)
0
1
2
3
4
0
2000
4000
6000
8000
10000
Nucleotide position

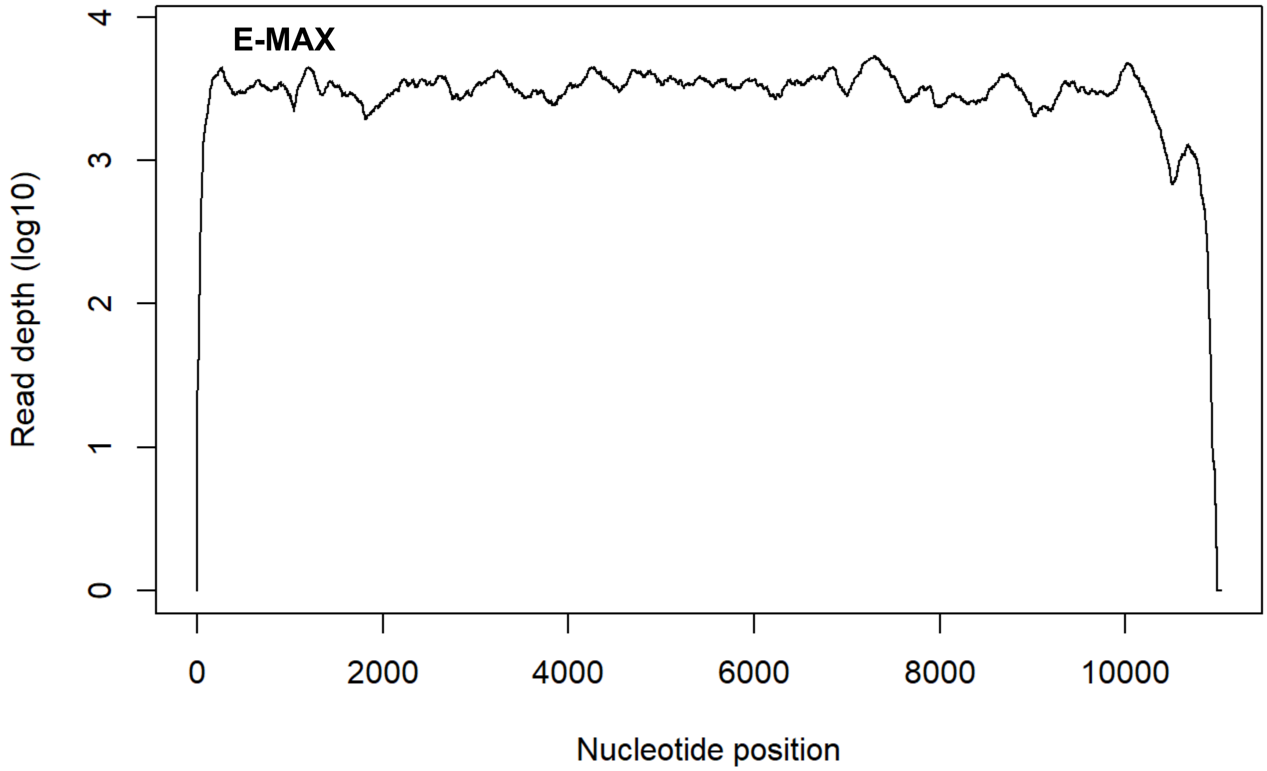

E-MAX
Read depth (log10)
0
1
2
3
4
0
2000
4000
6000
8000
10000
Nucleotide position

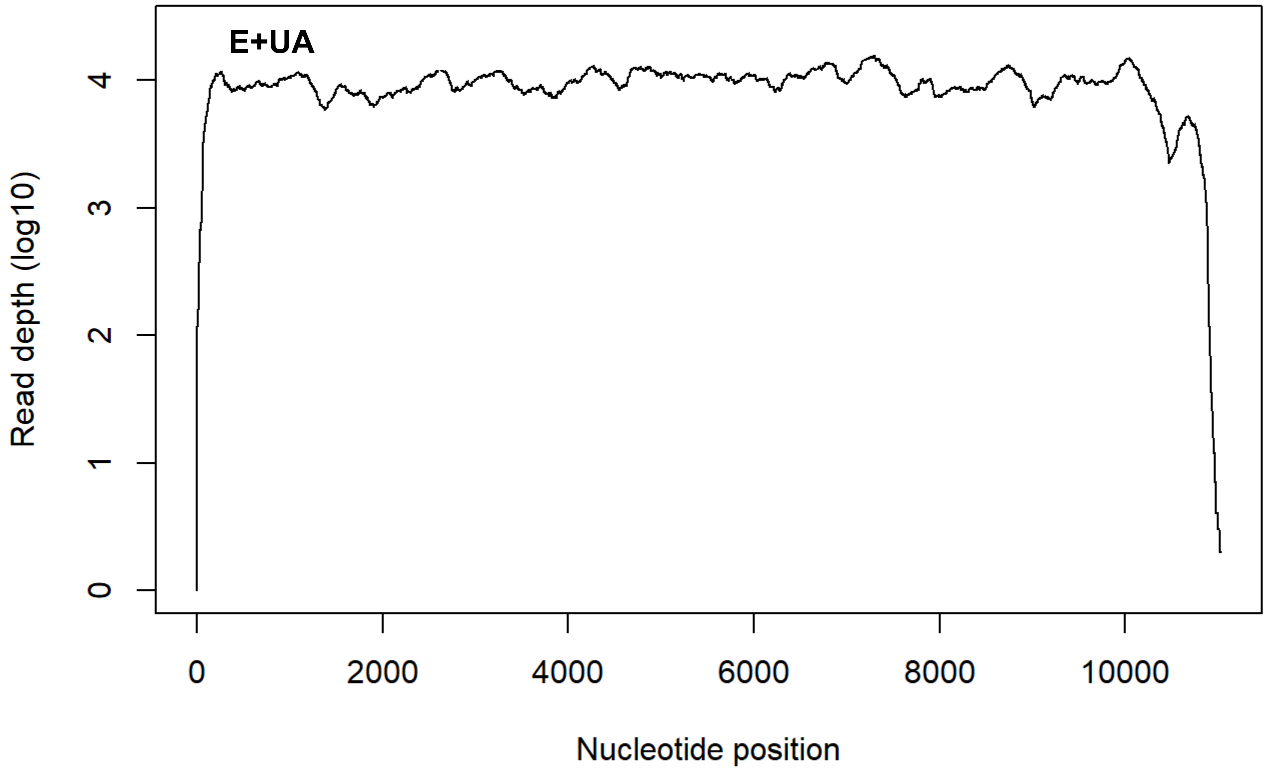

E+UA
Read depth (log10)
0
1
2
3
4
0
2000
4000
6000
8000
10000
Nucleotide position

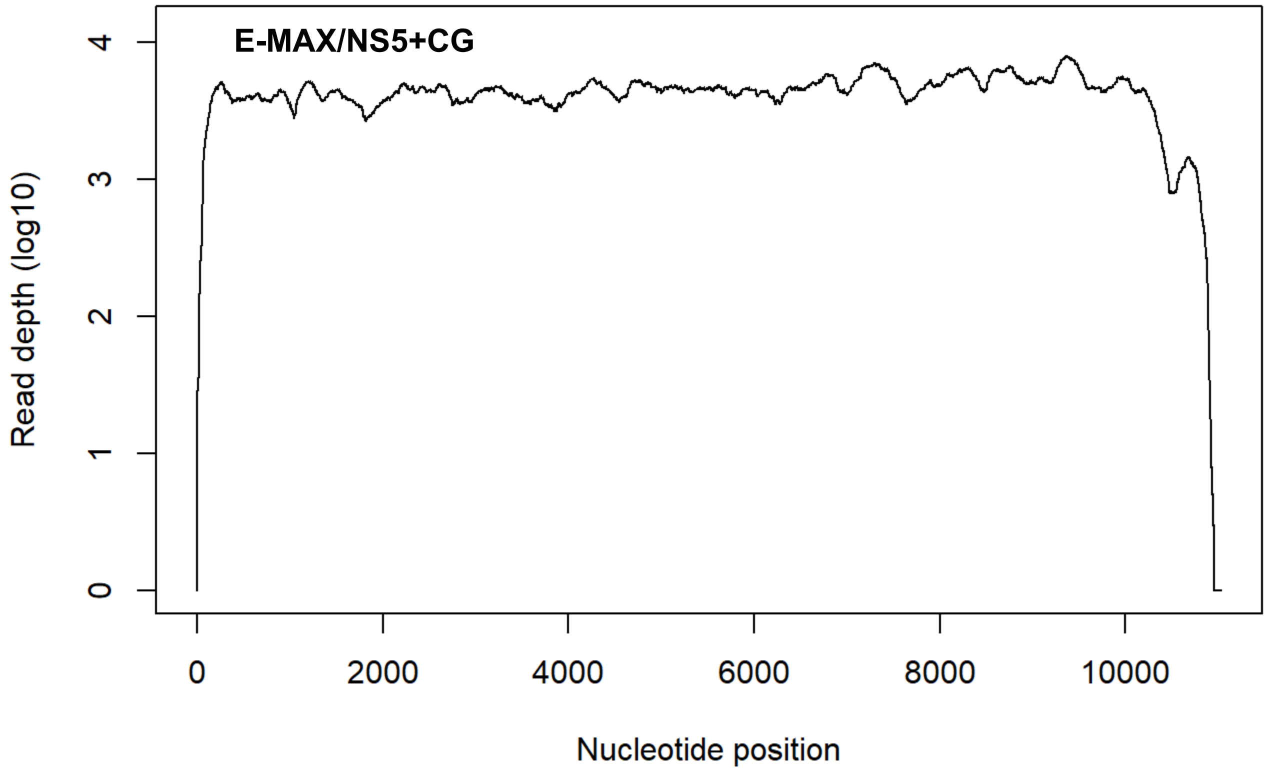
E-MAX/NS5+CG
Read depth (log10)
0
1
2
3
4
0
2000
4000
6000
8000
10000
Nucleotide position

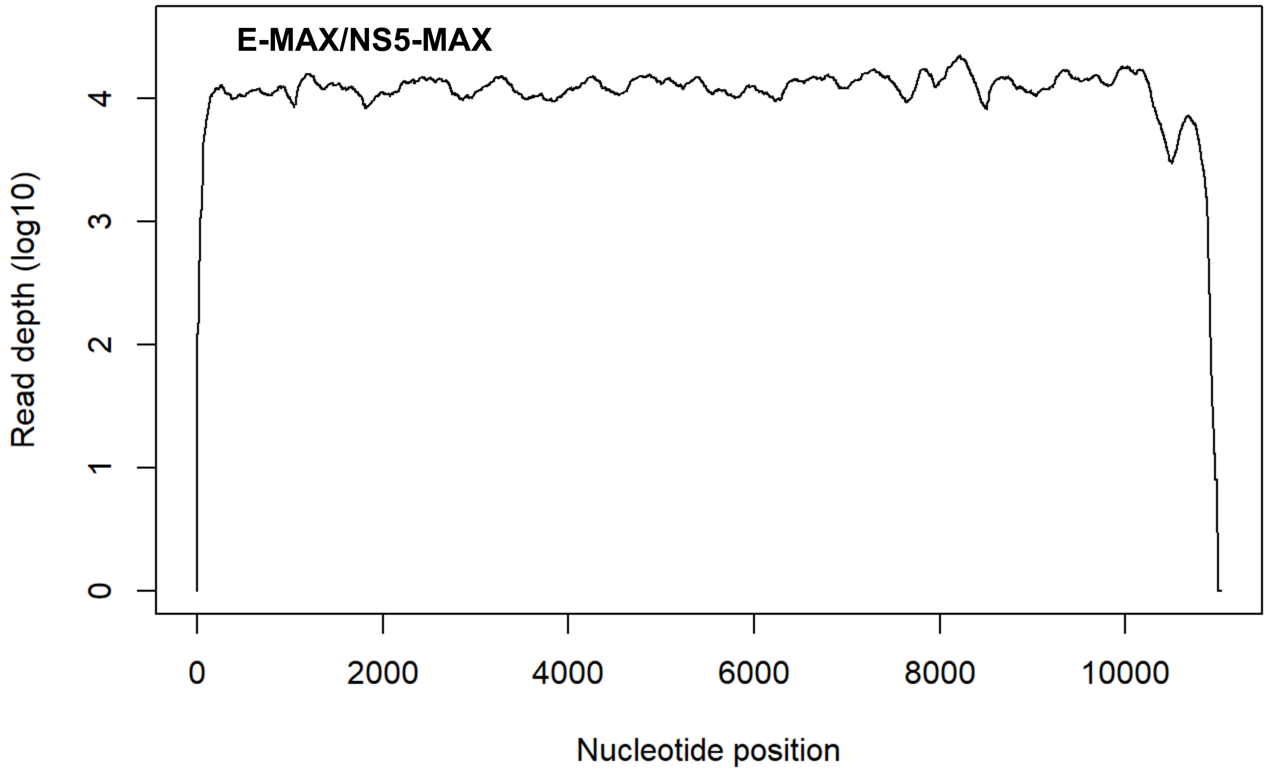
E-MAX/NS5-MAX
Read depth (log10)
0
1
2
3
4
0
2000
4000
6000
8000
10000
Nucleotide position

Data for Fig 10 in manuscript

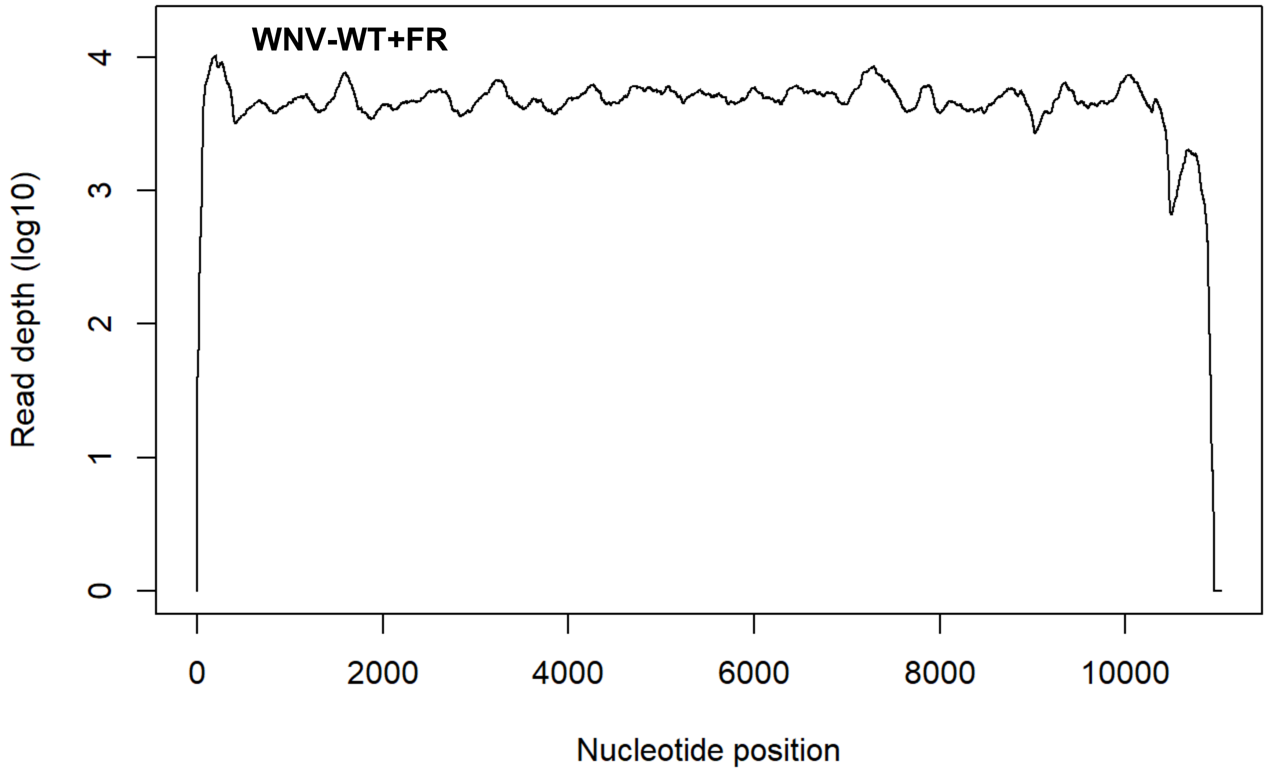

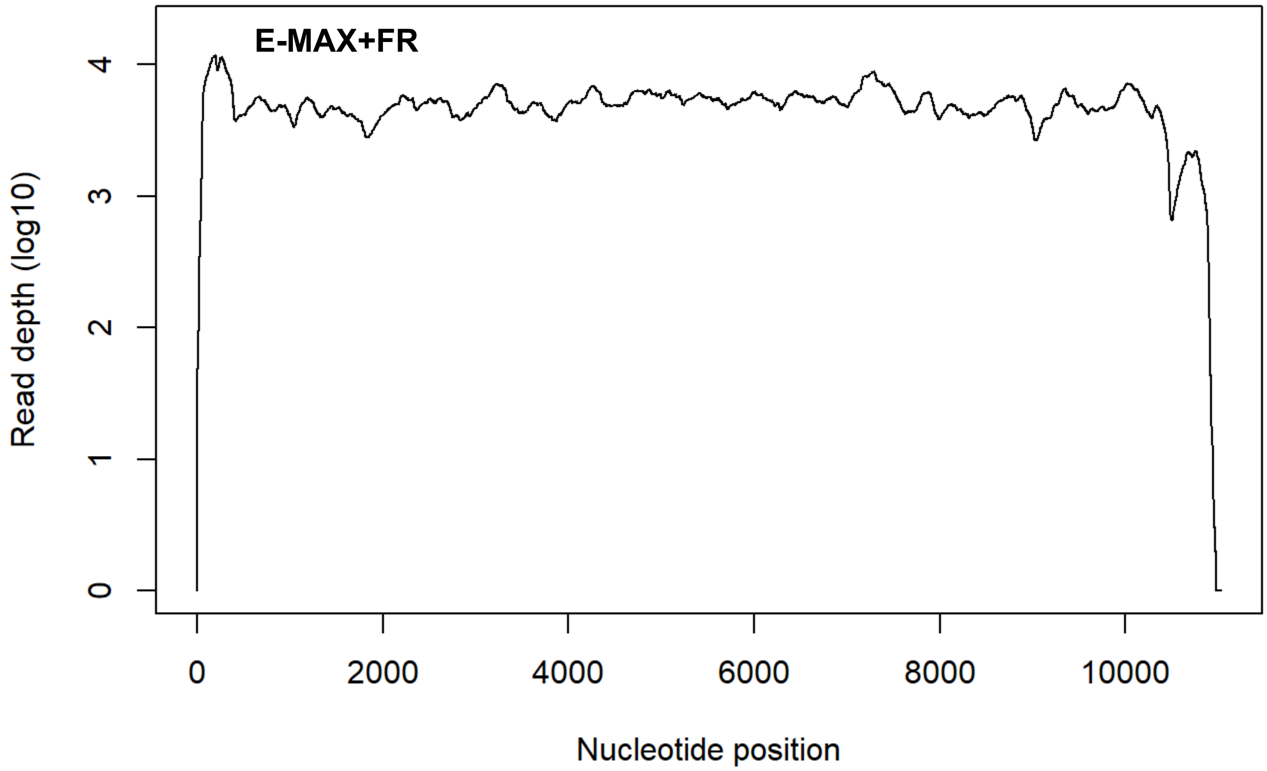
E-MAX+FR
Read depth (log10)
0
1
2
3
4
0
2000
4000
6000
8000
10000
Nucleotide position

Data for Fig 5 in manuscript

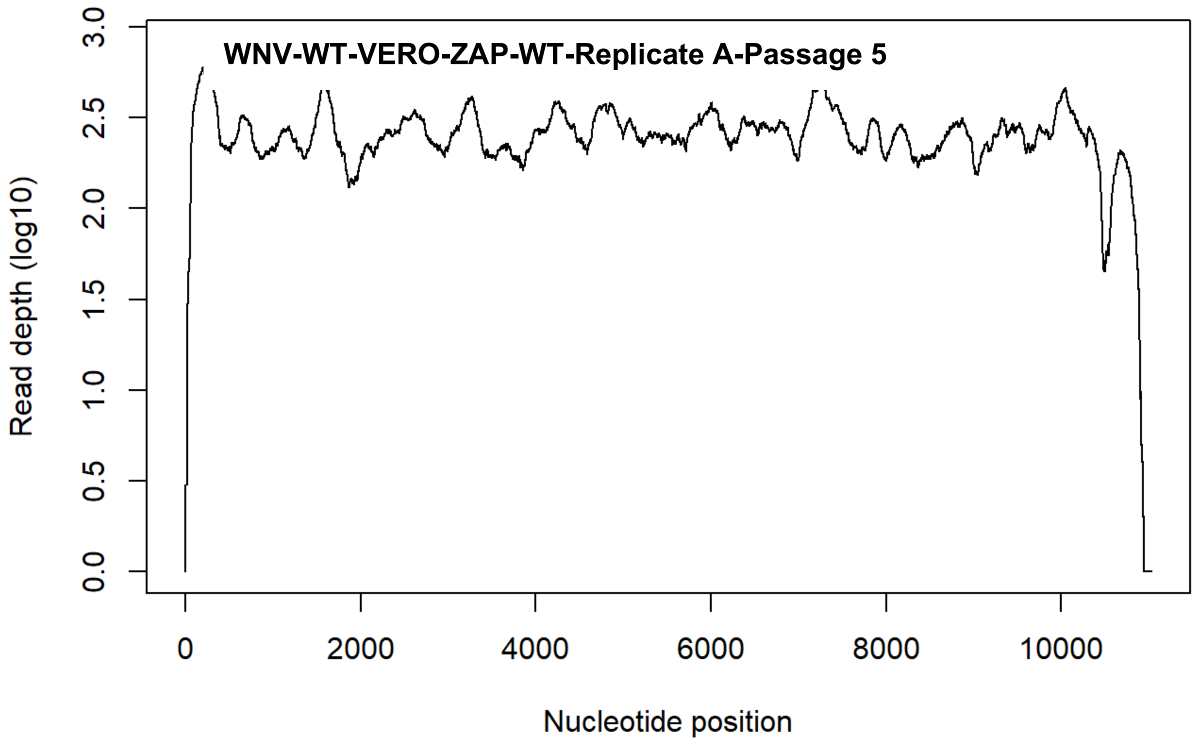

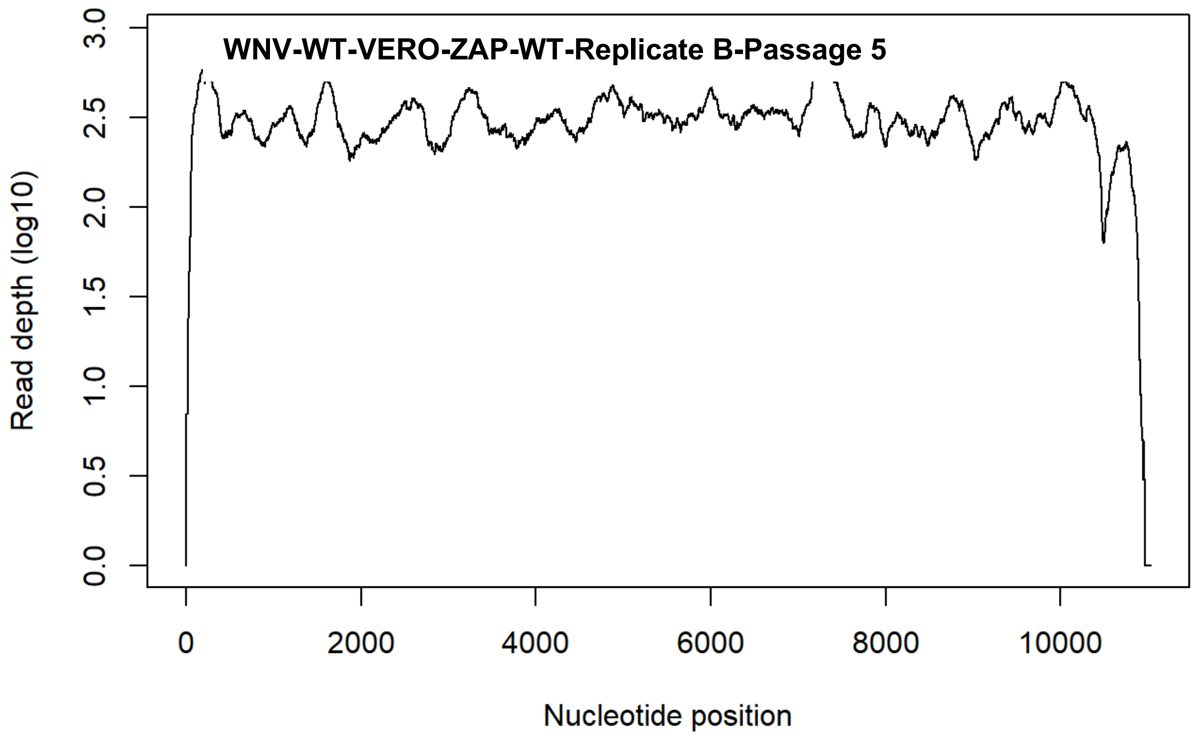

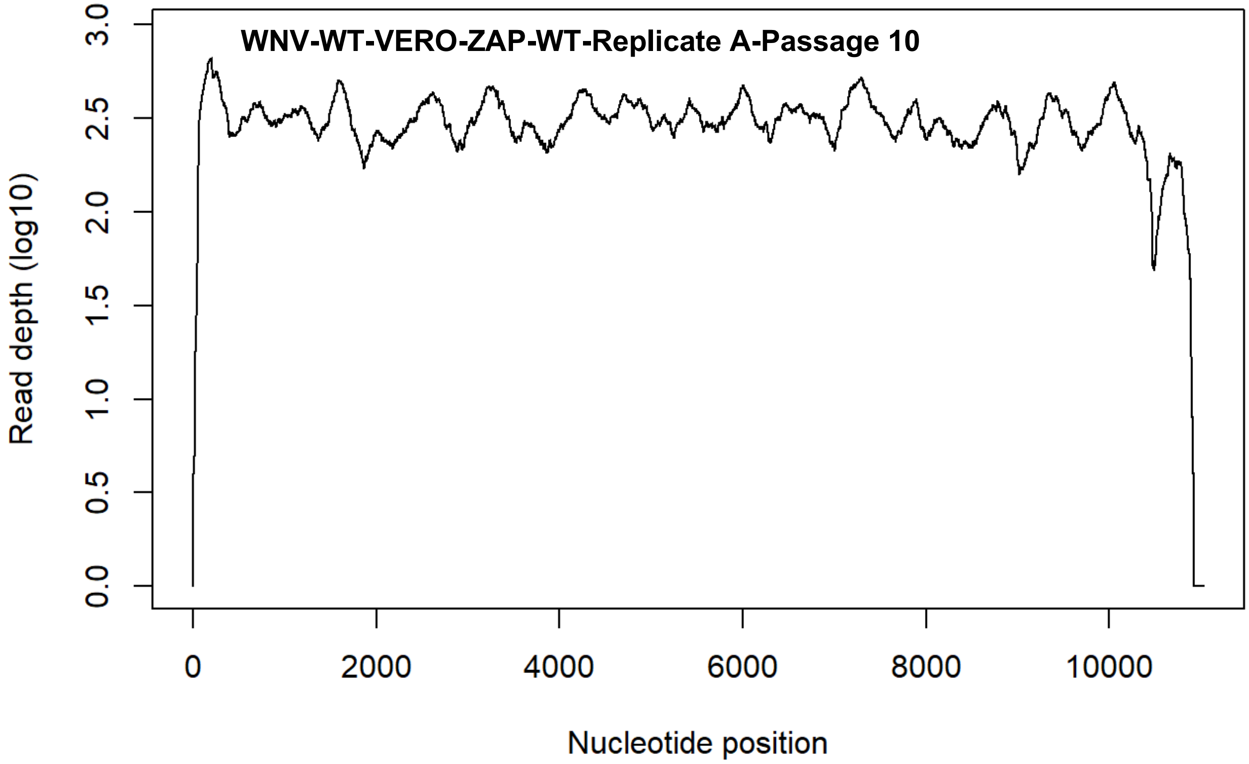
WNV-WT-VERO-ZAP-WT-Replicate A-Passage 10
Read depth (log10)
0.0
0.5
1.0
1.5
2.0
2.5
3.0
0
2000
4000
6000
8000
10000
Nucleotide position

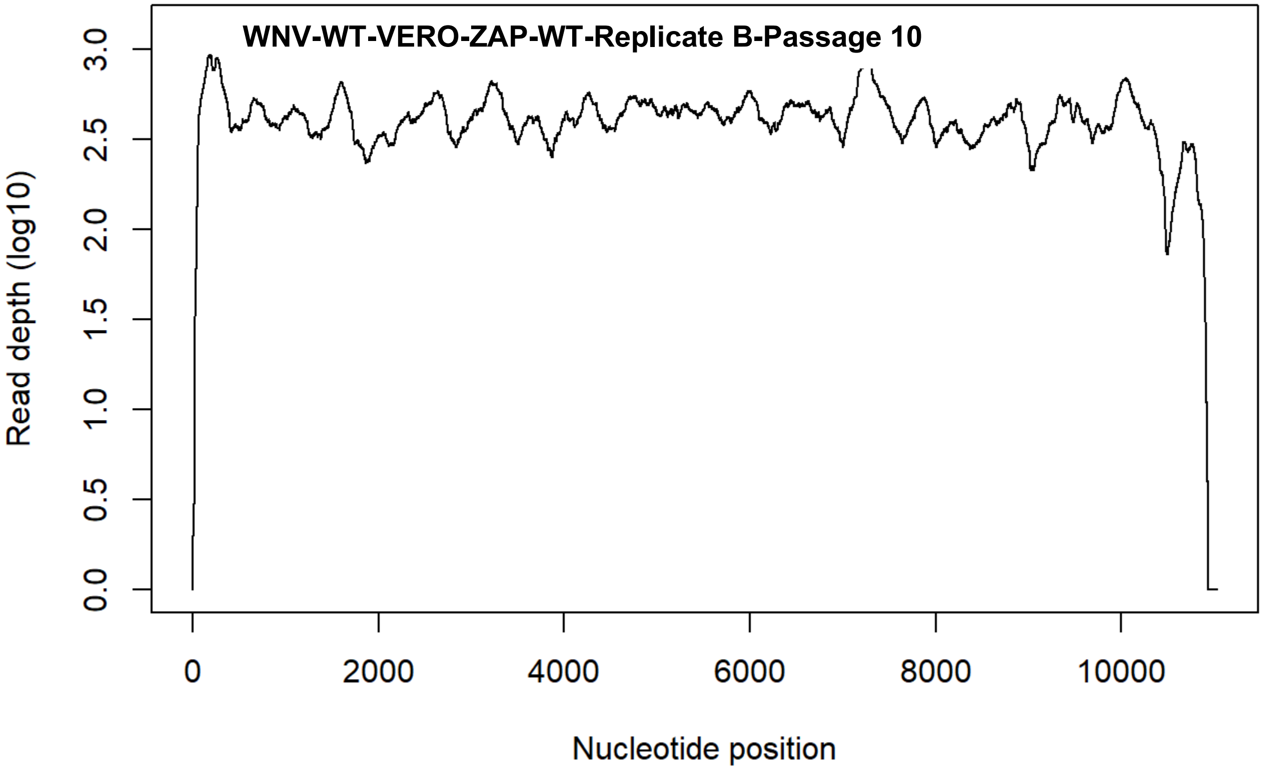
WNV-WT-VERO-ZAP-WT-Replicate B-Passage 10
Read depth (log10)
0.0
0.5
1.0
1.5
2.0
2.5
3.0
0
2000
4000
6000
8000
10000
Nucleotide position

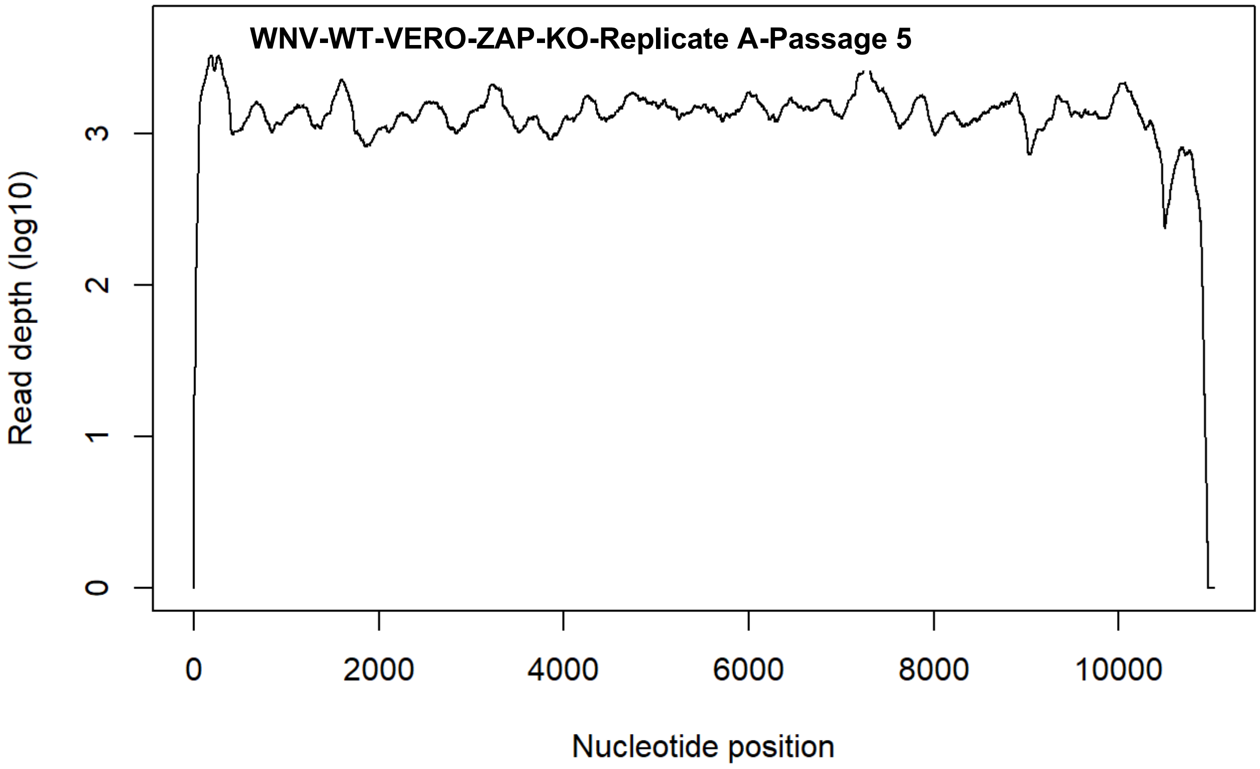

WNV-WT-VERO-ZAP-KO-Replicate A-Passage 5
Read depth (log10)
0
1
2
3
0
2000
4000
6000
8000
10000
Nucleotide position

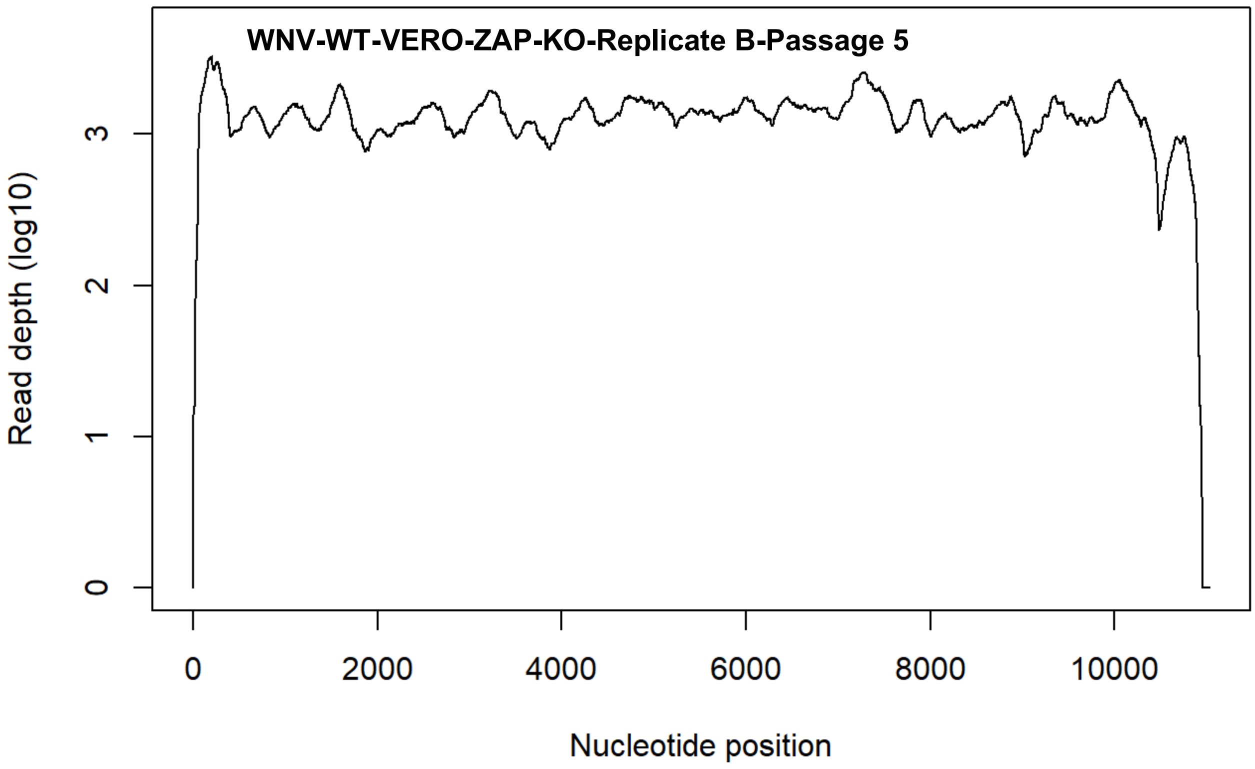

WNV-WT-VERO-ZAP-KO-Replicate B-Passage 5
Read depth (log10)
0
1
2
3
0
2000
4000
6000
8000
10000
Nucleotide position

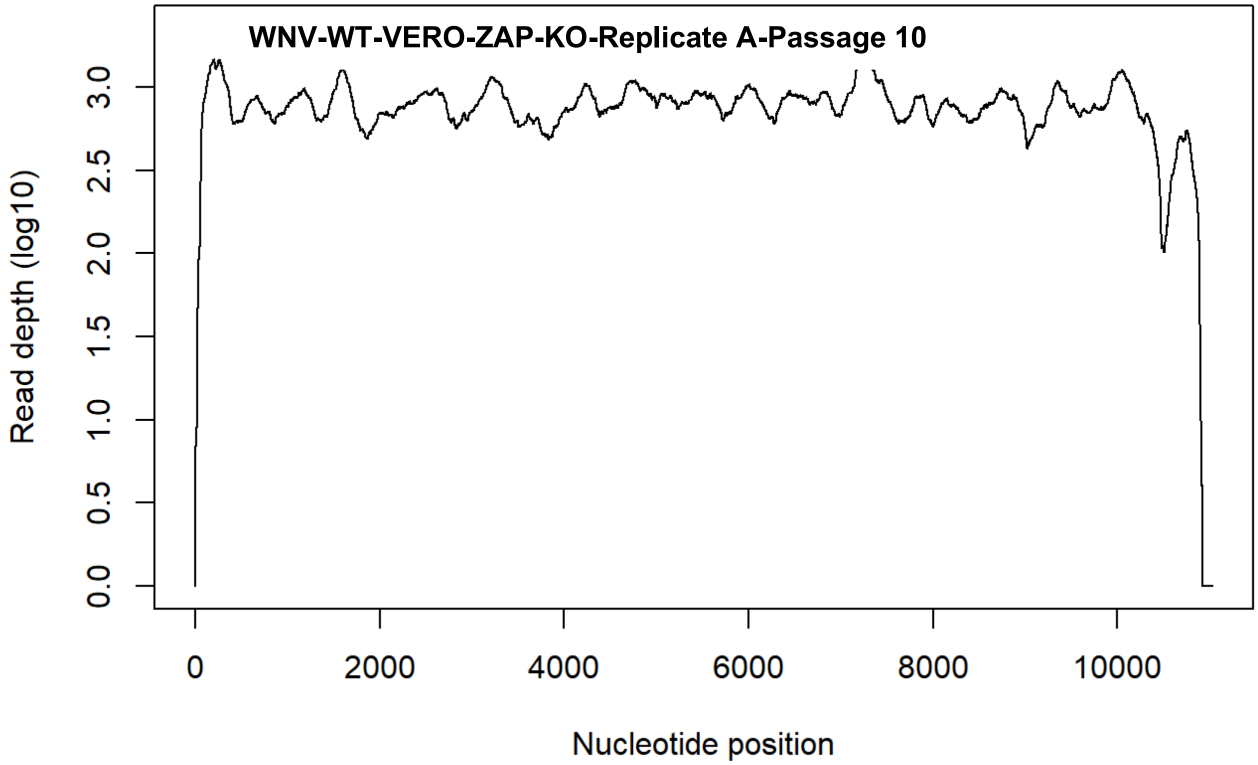
WNV-WT-VERO-ZAP-KO-Replicate A-Passage 10
Read depth (log10)
0.0
0.5
1.0
1.5
2.0
2.5
3.0
0
2000
4000
6000
8000
10000
Nucleotide position

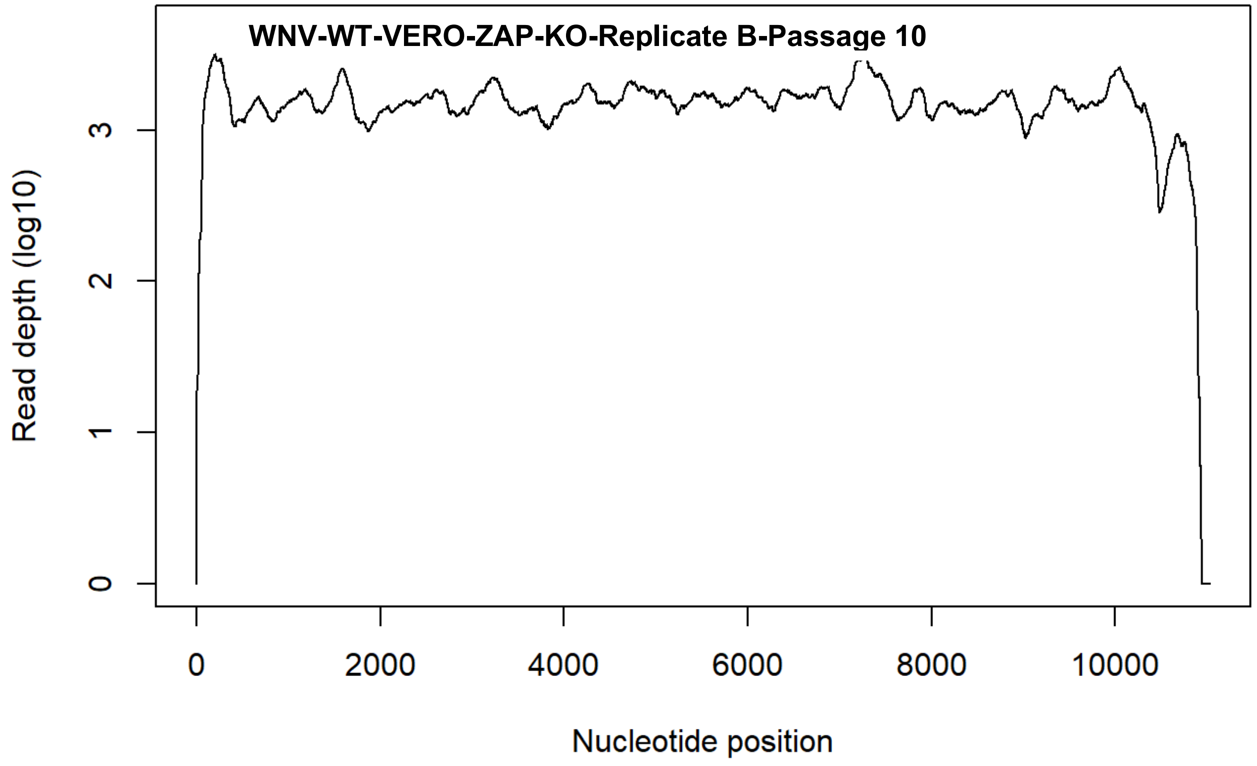
WNV-WT-VERO-ZAP-KO-Replicate B-Passage 10
Read depth (log10)
0
1
2
3
0
2000
4000
6000
8000
10000
Nucleotide position

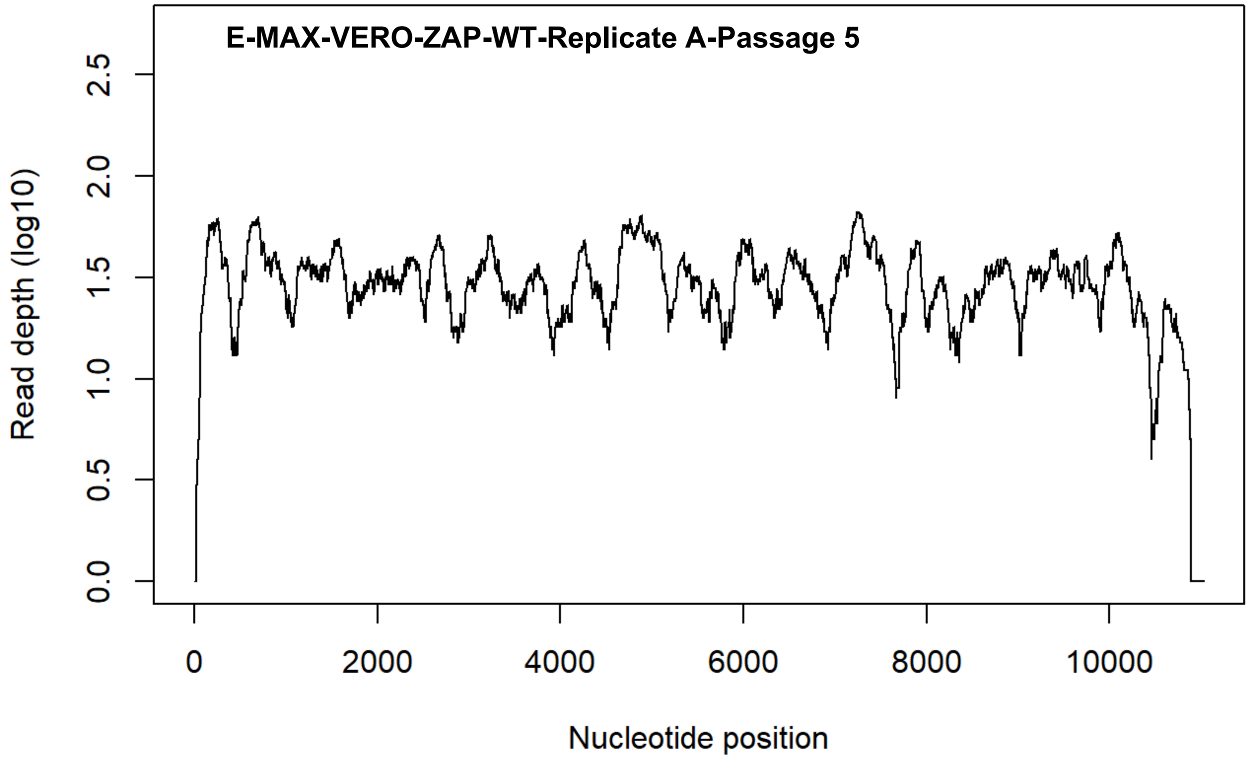
E-MAX-VERO-ZAP-WT-Replicate A-Passage 5
Read depth (log10)
0.0
0.5
1.0
1.5
2.0
2.5
0
2000
4000
6000
8000
10000
Nucleotide position

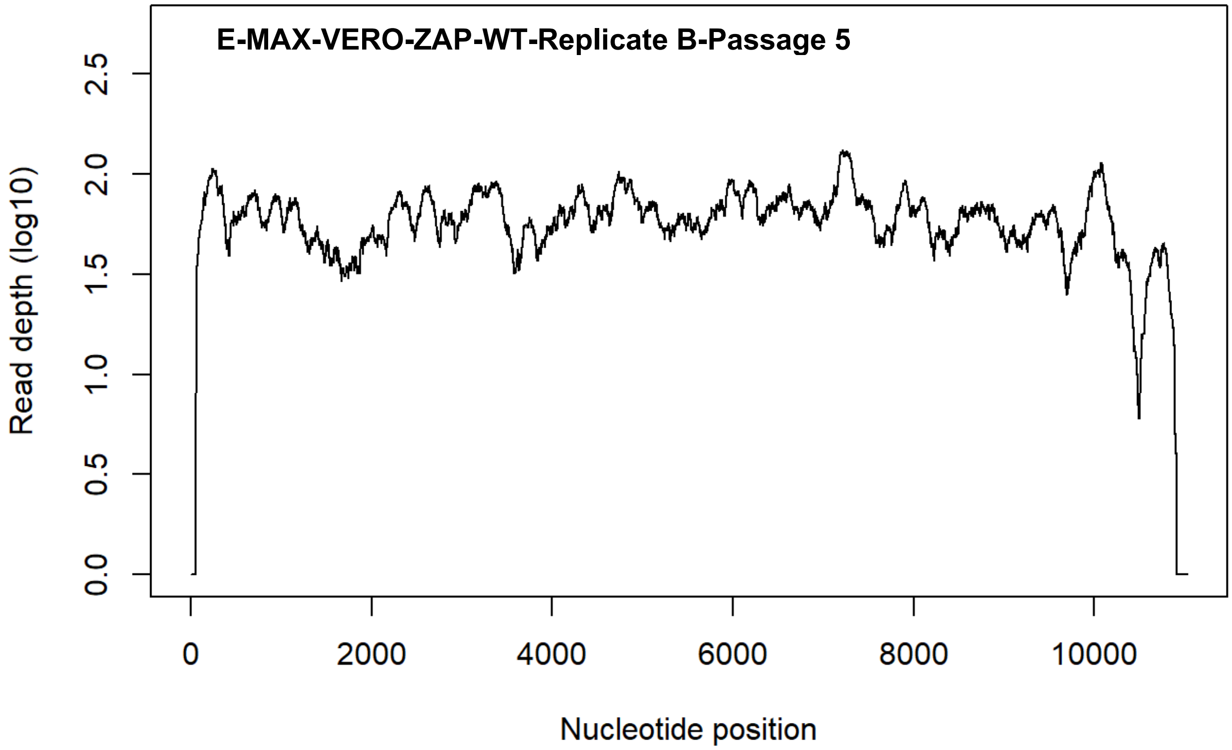
E-MAX-VERO-ZAP-WT-Replicate B-Passage 5
Read depth (log10)
0.0
0.5
1.0
1.5
2.0
2.5
0
2000
4000
6000
8000
10000
Nucleotide position

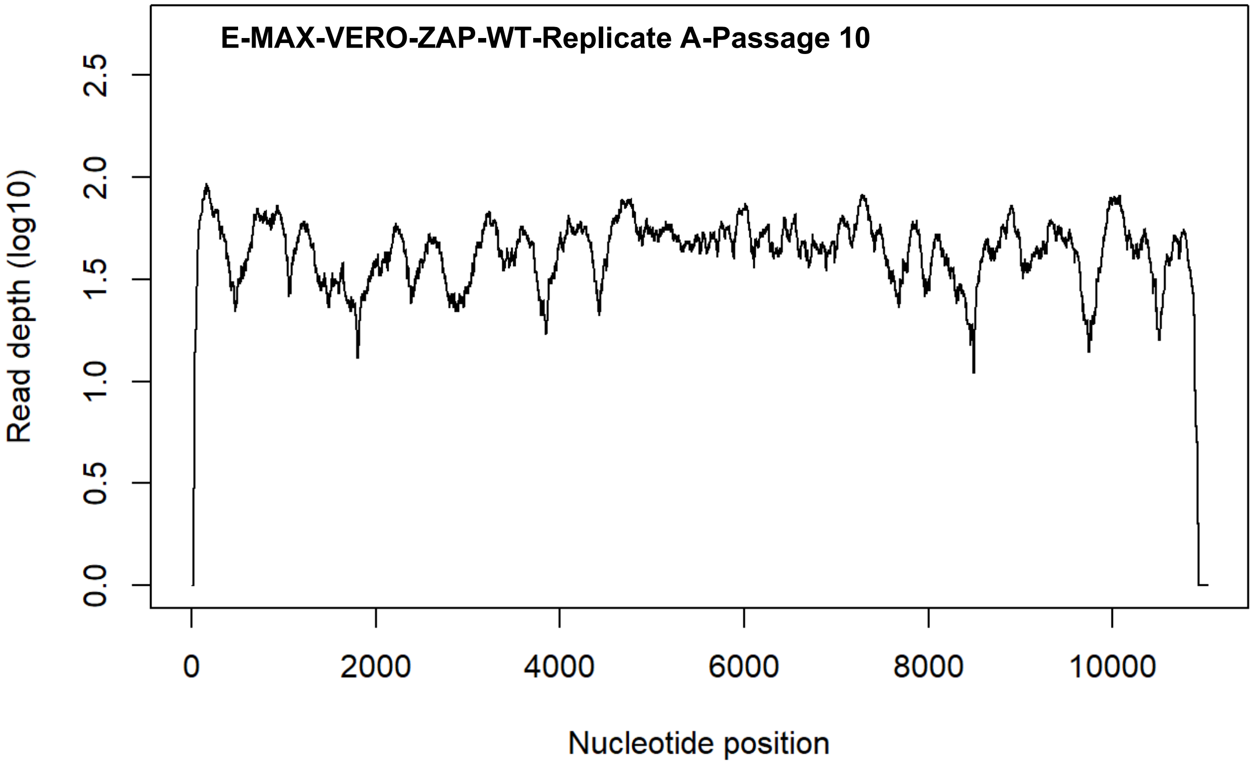
E-MAX-VERO-ZAP-WT-Replicate A-Passage 10
Read depth (log10)
0.0
0.5
1.0
1.5
2.0
2.5
0
2000
4000
6000
8000
10000
Nucleotide position

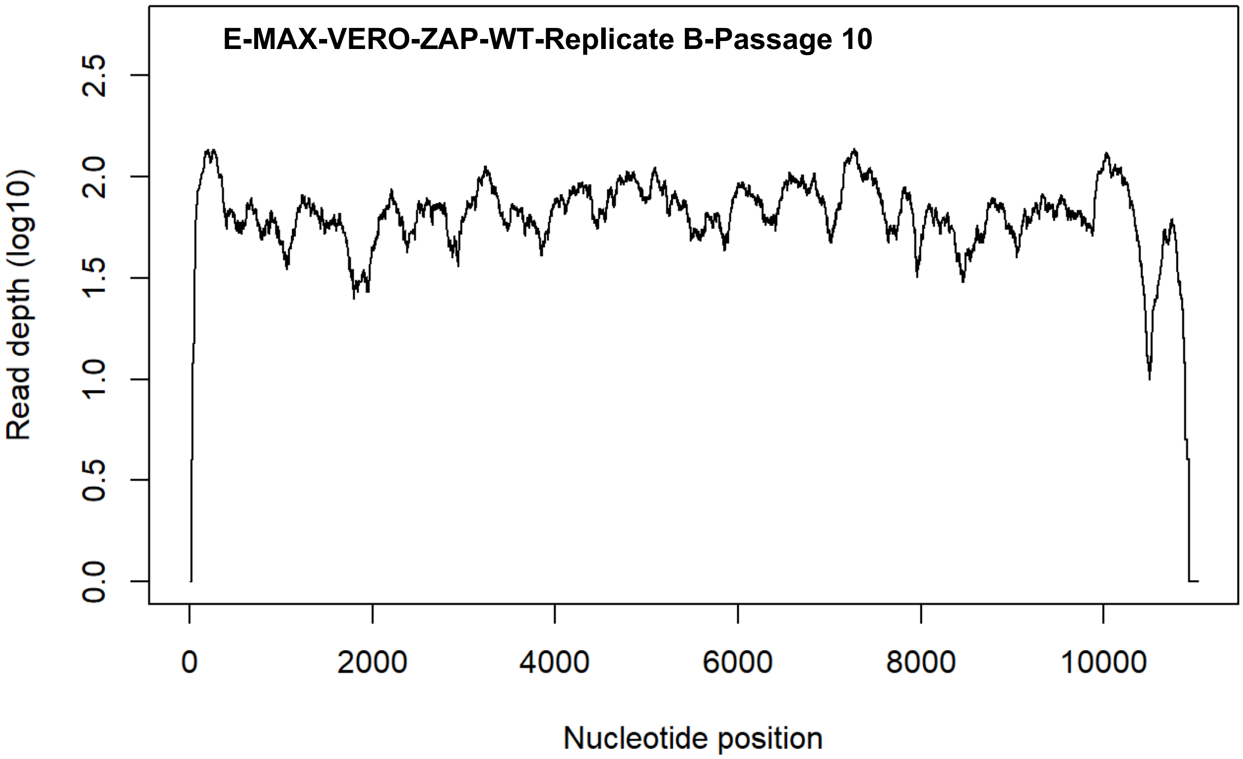
E-MAX-VERO-ZAP-WT-Replicate B-Passage 10
Read depth (log10)
0.0
0.5
1.0
1.5
2.0
2.5
0
2000
4000
6000
8000
10000
Nucleotide position

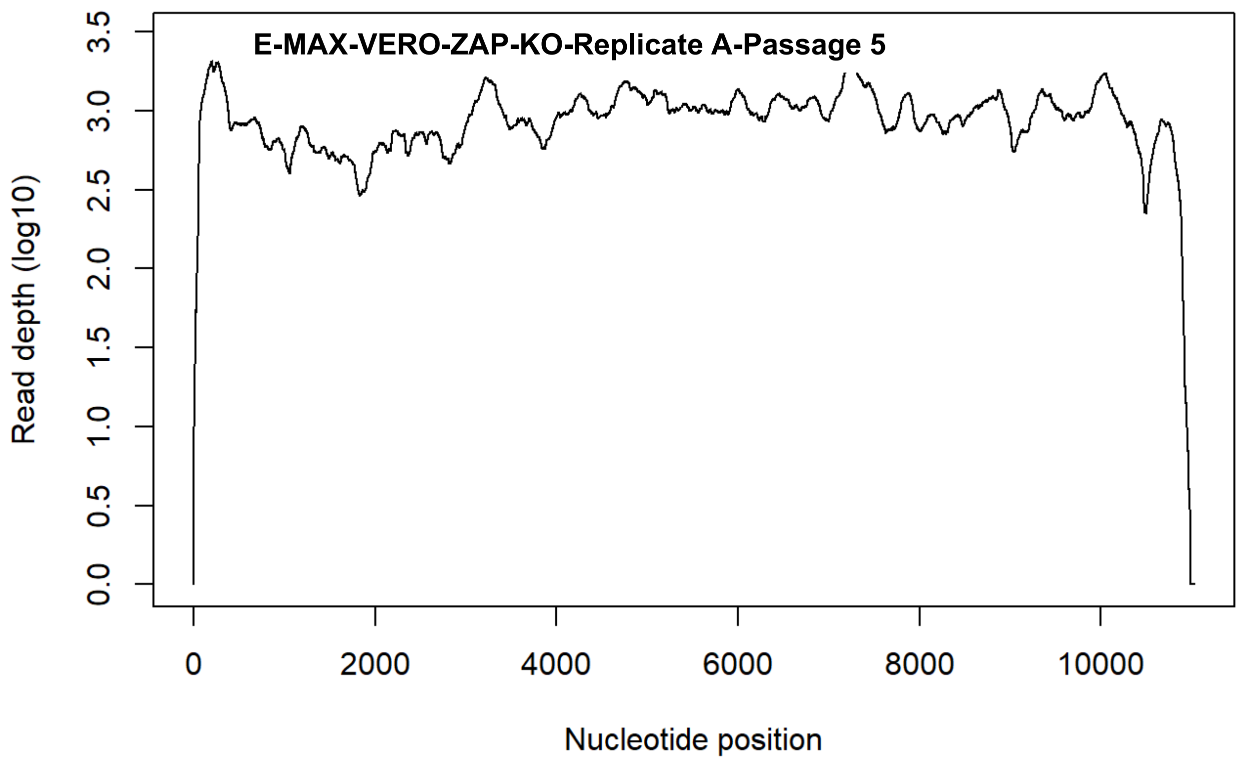
E-MAX-VERO-ZAP-KO-Replicate A-Passage 5
Read depth (log10)
0.0
0.5
1.0
1.5
2.0
2.5
3.0
3.5
0
2000
4000
6000
8000
10000
Nucleotide position

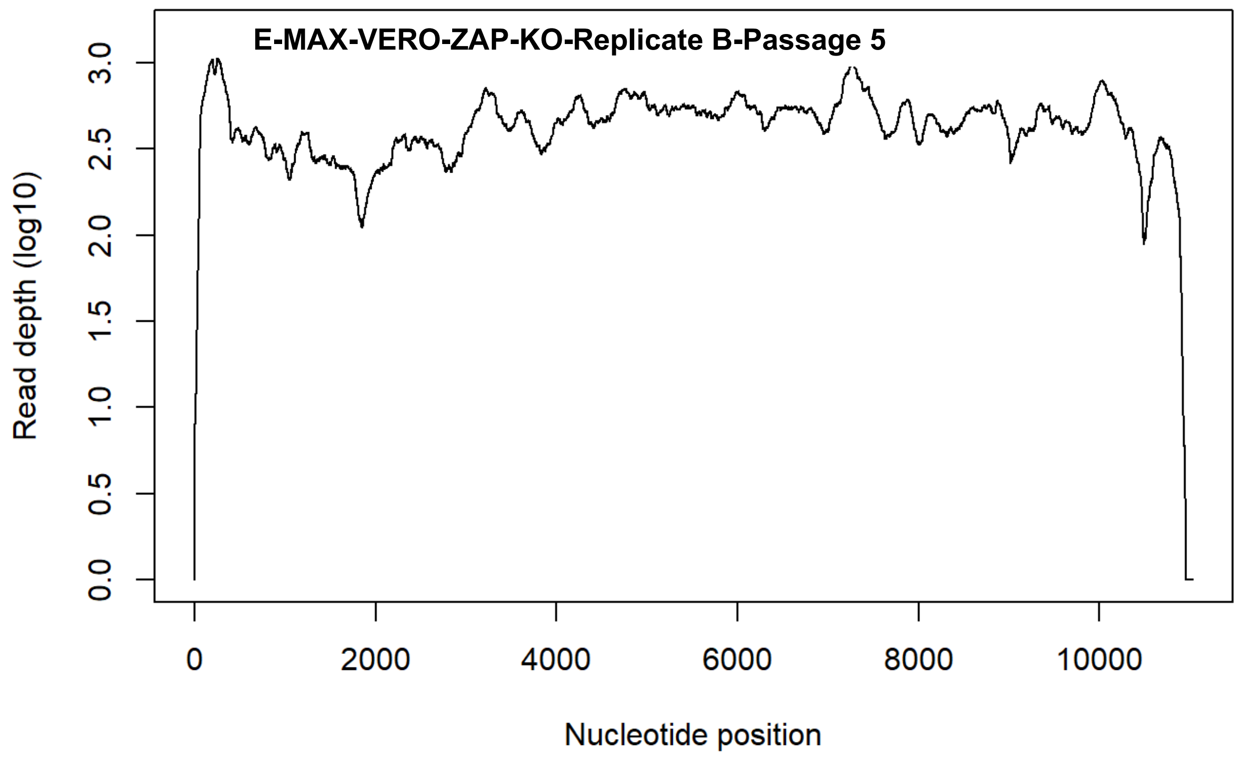
E-MAX-VERO-ZAP-KO-Replicate B-Passage 5
Read depth (log10)
0.0
0.5
1.0
1.5
2.0
2.5
3.0
0
2000
4000
6000
8000
10000
Nucleotide position

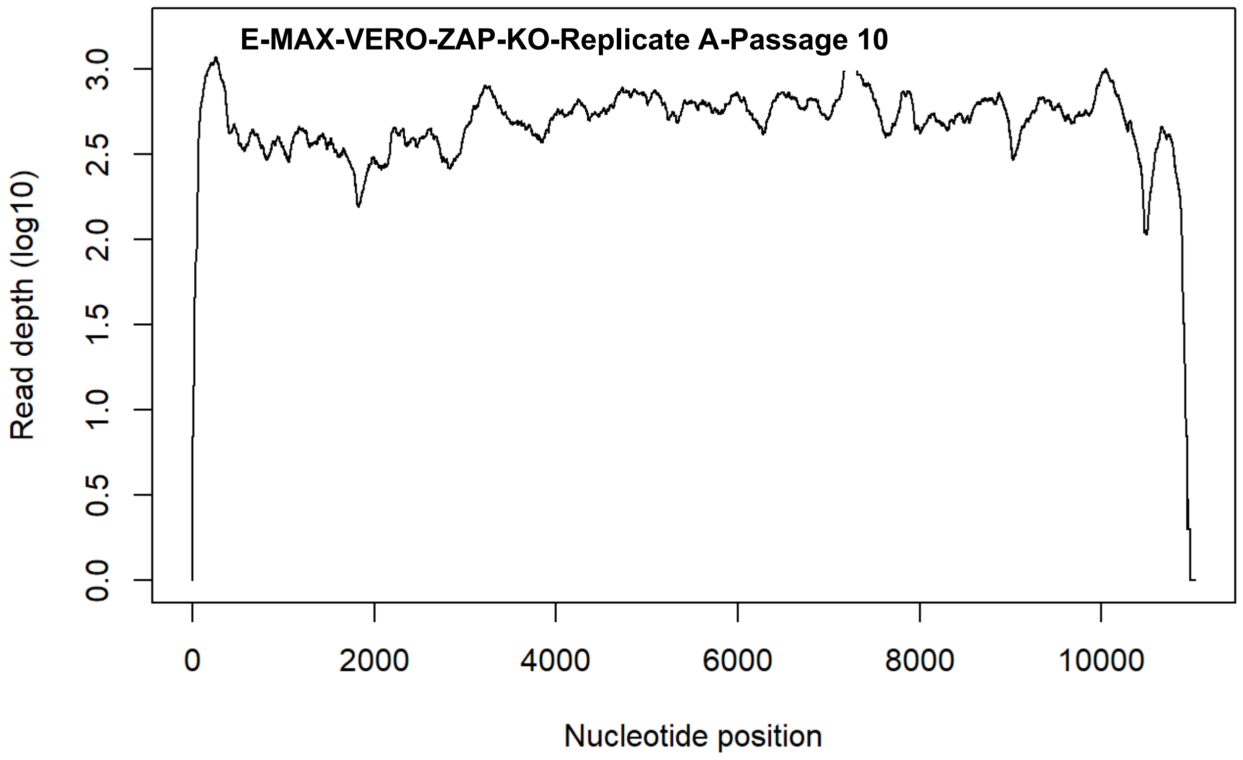
E-MAX-VERO-ZAP-KO-Replicate A-Passage 10
Read depth (log10)
0.0
0.5
1.0
1.5
2.0
2.5
3.0
0
2000
4000
6000
8000
10000
Nucleotide position

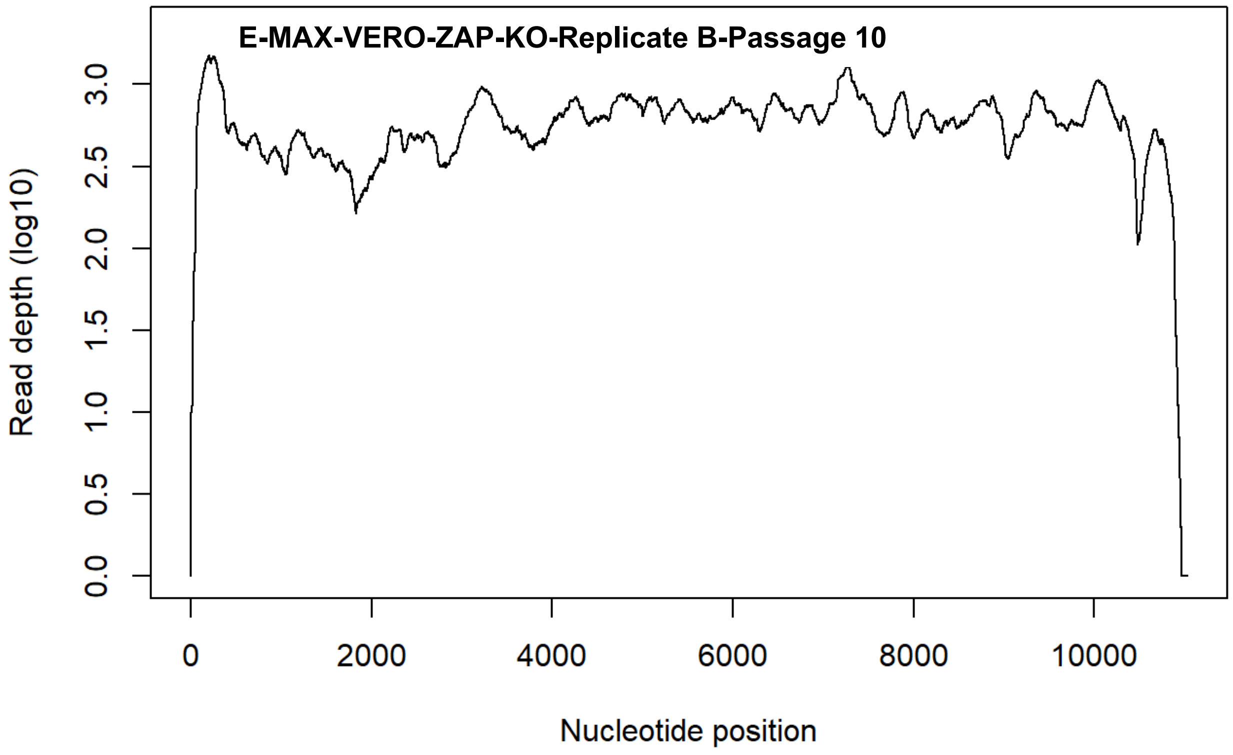
E-MAX-VERO-ZAP-KO-Replicate B-Passage 10
Read depth (log10)
0.0
0.5
1.0
1.5
2.0
2.5
3.0
0
2000
4000
6000
8000
10000
Nucleotide position

Data for Fig 4N in manuscript

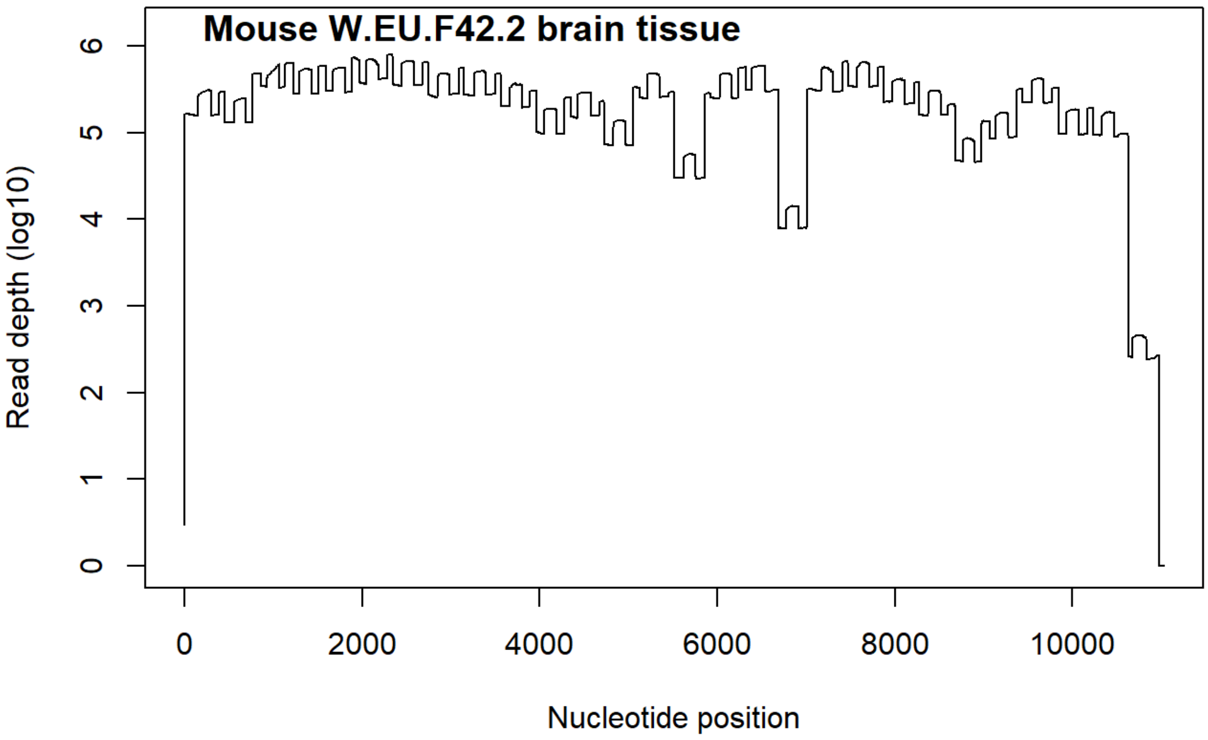

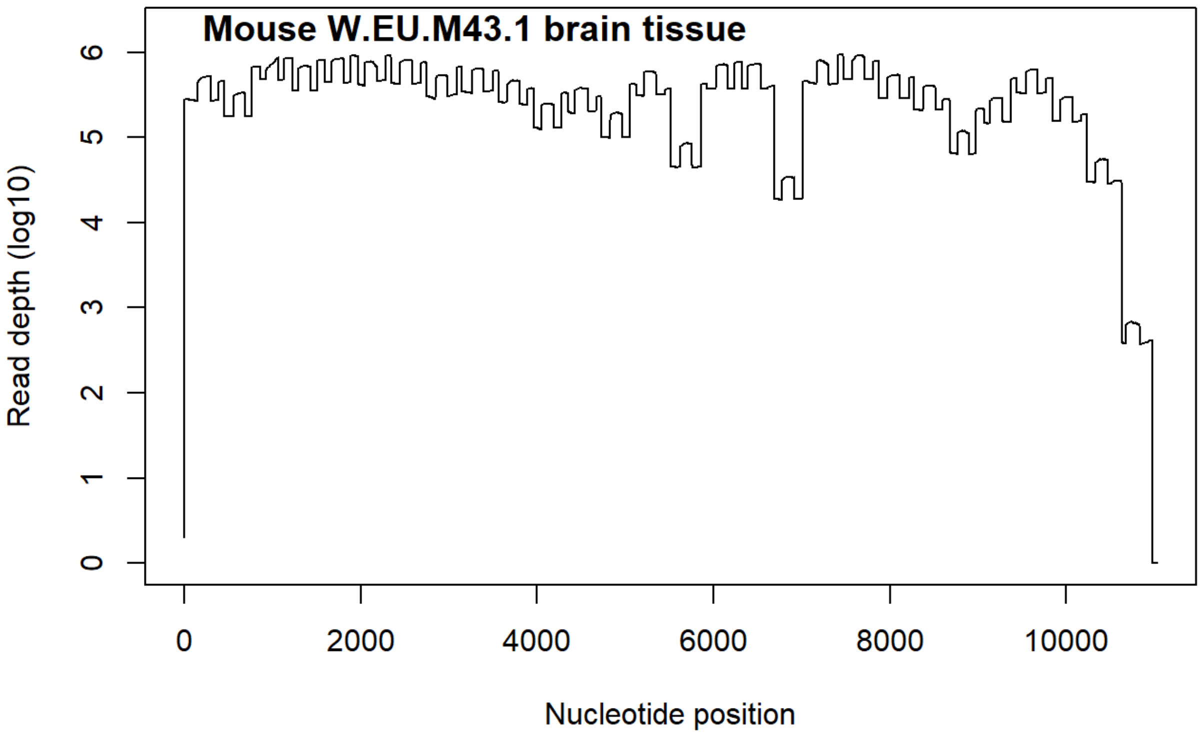

Data for Fig 9M in manuscript

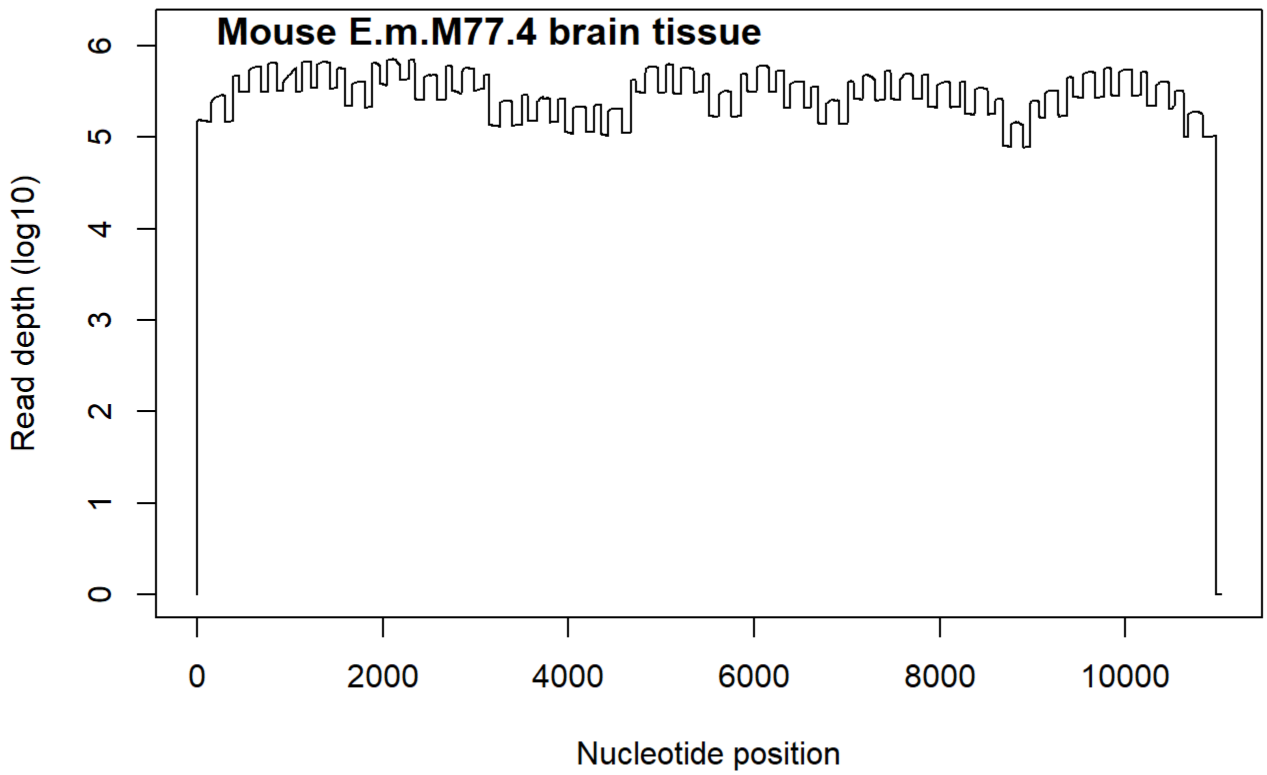

Supplement: S5 File — (PDF) [file ppat.1013560.s008.pdf]
