# Supplementary material for: West Nile virus vaccine candidates attenuated by dinucleotide enrichment are immunogenic and protective against lethal infection
Source: PLoS Pathog. 2025 Oct 3;21(10):e1013560. doi: 10.1371/journal.ppat.1013560 (PMC12513643; doi:10.1371/journal.ppat.1013560)

File S6 Shannon entropy

Shannon entropy (corresponds to SNV data in Fig 2, Fig 10 (FR variants), Table S3)

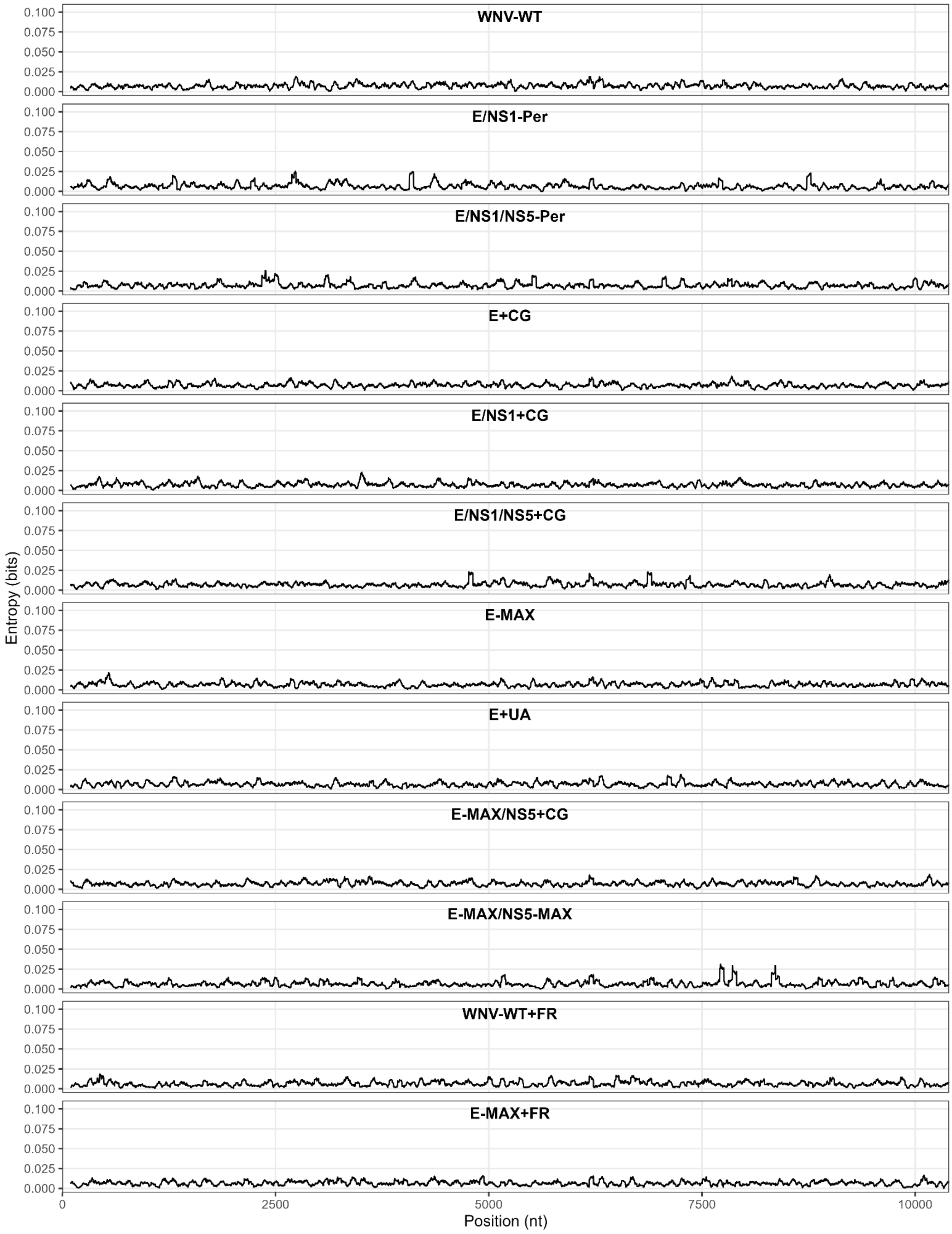

File S6 Shannon entropy

Shannon entropy (corresponds to SNV data in Fig 5, Table S3)

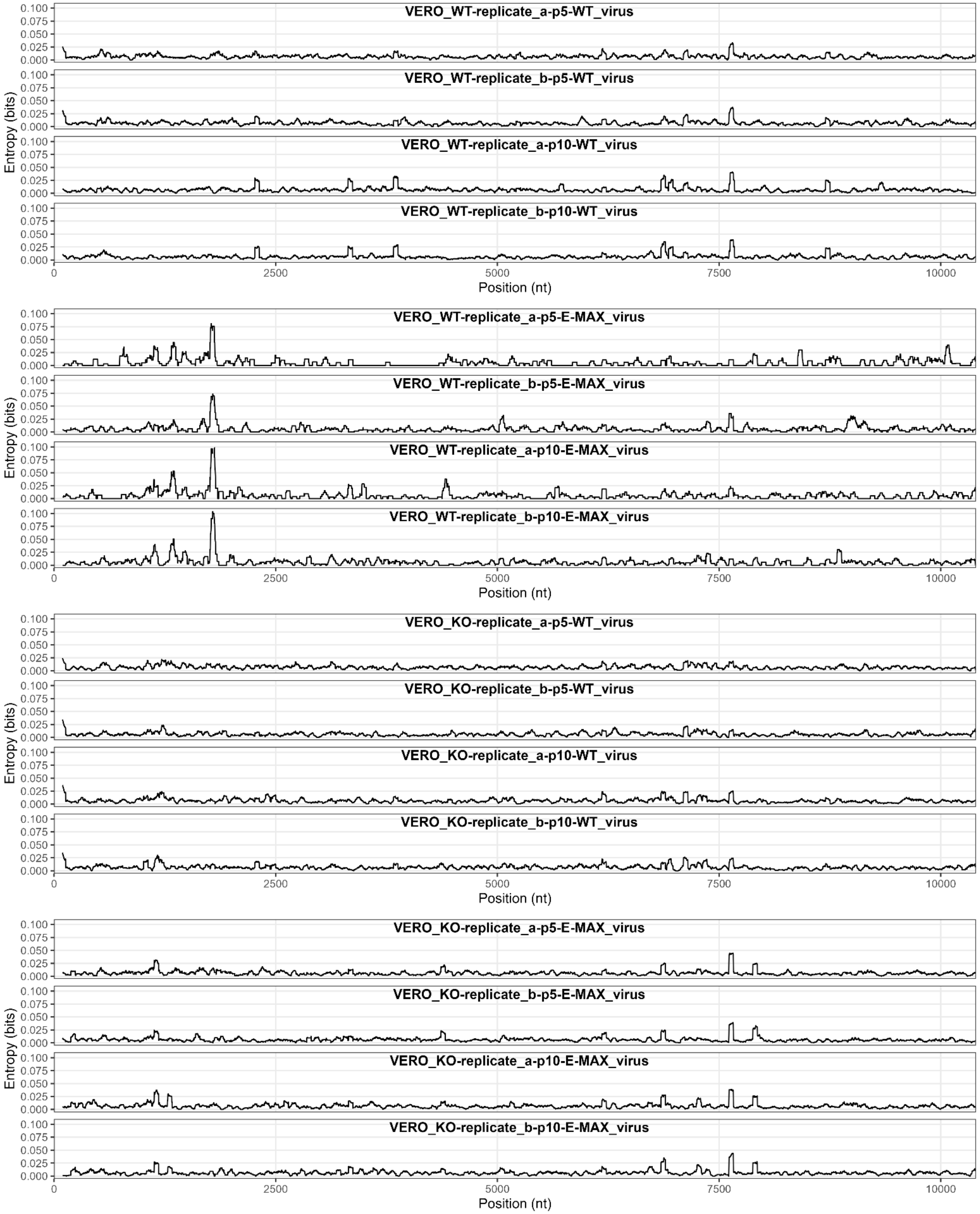

a and b: represent passage replicates.

File S6 Shannon entropy

Shannon entropy (corresponds to SNV data in Fig 4N, Table S3)

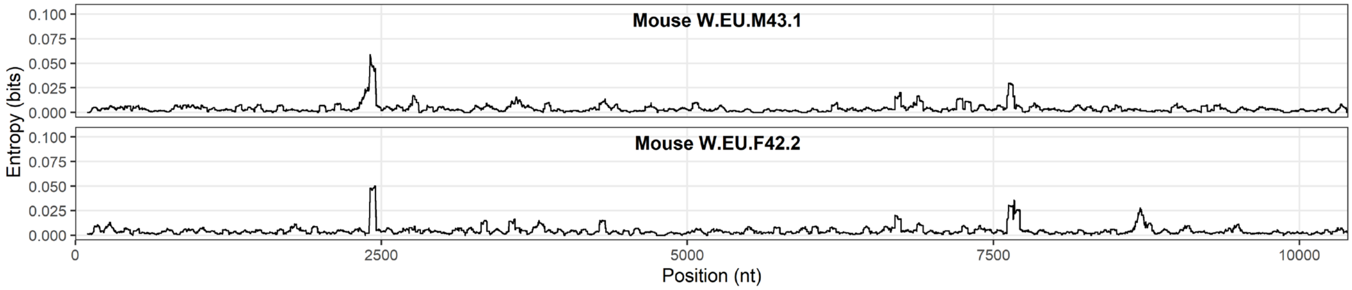

Shannon entropy (corresponds to SNV data in Fig 9M, Table S3)

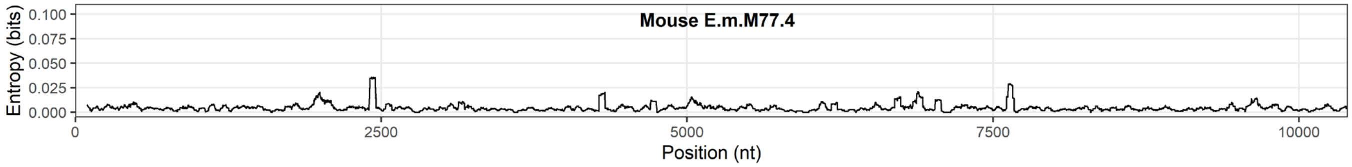

Supplement: S6 File — (PDF) [file ppat.1013560.s009.pdf]
