# Supplementary material for: West Nile virus vaccine candidates attenuated by dinucleotide enrichment are immunogenic and protective against lethal infection
Source: PLoS Pathog. 2025 Oct 3;21(10):e1013560. doi: 10.1371/journal.ppat.1013560 (PMC12513643; doi:10.1371/journal.ppat.1013560)
Supplement: S1 Table — (PDF) [file ppat.1013560.s010.pdf]

Table S1 CpG and UpA composition in WNV variants

WNV RNA region	WNV variant	CpG #	CpG ratio*	CpG O/E	UpA #	UpA ratio*	UpA O/E	GC3	ENc ratio	CPB
**E**	WNV-WT	49	1.00	0.50	36	1.00	0.41	0.56	0.84	0.038
	E/NS1-Per	48	0.98	0.49	36	1.00	0.41	0.56	0.87	0.031
	E/NS1/NS5-Per	48	0.98	0.49	36	1.00	0.41	0.56	0.87	0.031
	E+CG	179	3.65	1.83	39	1.08	0.44	0.53	0.75	-0.203
	E/NS1+CG	179	3.65	1.83	39	1.08	0.44	0.53	0.75	-0.203
	E/NS1/NS5+CG	179	3.65	1.83	39	1.08	0.44	0.53	0.75	-0.203
	E-MAX	208	4.25	2.12	86	2.39	0.97	0.53	0.73	-0.284
	E+UA	49	1.00	0.50	86	2.39	0.97	0.56	0.91	0.03
	E-MAX/NS5+CG	208	4.25	2.12	86	2.39	0.97	0.53	0.73	-0.284
	E-MAX/NS5-MAX	208	4.25	2.12	86	2.39	0.97	0.53	0.73	-0.284
**NS1**	WNV-WT	37	1	0.56	30	1	0.49	0.56	0.93	0.012
	E/NS1-Per	37	1	0.56	30	1	0.49	0.56	0.95	0.038
	E/NS1/NS5-Per	37	1	0.56	30	1	0.49	0.56	0.95	0.038
	E+CG	37	1	0.56	30	1	0.49	0.56	0.93	0.012
	E/NS1+CG	117	3.16	1.75	32	1.07	0.52	0.53	0.79	-0.208
	E/NS1/NS5+CG	117	3.16	1.75	32	1.07	0.52	0.53	0.79	-0.208
	E-MAX	37	1	0.56	30	1	0.49	0.56	0.93	0.012
	E+UA	37	1	0.56	30	1	0.49	0.56	0.93	0.012
	E-MAX/NS5+CG	37	1	0.56	30	1	0.49	0.56	0.93	0.012
	E-MAX/NS5-MAX	37	1	0.56	30	1	0.49	0.56	0.93	0.012
**NS5**	WNV-WT	93	1	0.53	55	1	0.36	0.57	0.89	0.022
	E/NS1-Per	93	1	0.53	55	1	0.36	0.57	0.89	0.022
	E/NS1/NS5-Per	93	1	0.53	55	1	0.36	0.57	0.90	0.031
	E+CG	93	1	0.53	55	1	0.36	0.57	0.89	0.022
	E/NS1+CG	93	1	0.53	55	1	0.36	0.57	0.89	0.022
	E/NS1/NS5+CG	306	3.29	1.73	56	1.02	0.37	0.52	0.74	-0.173
	E-MAX	93	1	0.53	55	1	0.36	0.57	0.89	0.022
	E+UA	93	1	0.53	55	1	0.36	0.57	0.89	0.022
	E-MAX/NS5+CG	306	3.29	1.73	56	1.02	0.37	0.52	0.74	-0.173
	E-MAX/NS5-MAX	369	3.97	2.09	144	2.62	0.94	0.52	0.73	-0.27

***CpG or UpA ratio:** The number of corresponding dinucleotides in the permuted or enriched WNV variant/the number in the WNV-WT. **CpG** or **UpA O/E:** Ratio of observed dinucleotide frequencies to estimated. **GC3:** Guanine (G) and cytosine (C) composition at the third codon position. **Enc ratio**: Effective number of codons ratio. **CPB:** Codon pair bias.
