# Supplementary material for: West Nile virus vaccine candidates attenuated by dinucleotide enrichment are immunogenic and protective against lethal infection
Source: PLoS Pathog. 2025 Oct 3;21(10):e1013560. doi: 10.1371/journal.ppat.1013560 (PMC12513643; doi:10.1371/journal.ppat.1013560)
Supplement: S5 Table — (PDF) [file ppat.1013560.s014.pdf]

Table S5 ISA fragments and primers

Synthetic clonal genes—DNA fragments are synthesized, inserted into the bacterial plasmids, amplified in bacteria, extracted, and plasmids with inserted fragments are delivered as lyophilized DNA with known concentration.

Synthesis of DNA—DNA fragments, flanked with 22 nt adapters, are rapidly (2-4 days) synthesized without using bacterial plasmids and bacteria.

For sequences of each ISA fragment, see **File S2**.

Virus	ISA fragment ID	Plasmids	Fragment origin	Primers
**WNV-WT**				
	wt-NY99_**Fragment-I**	pCC1	synthetic clonal genes	WNV-wt-NY99-I-Forward: CACCCAACTGATCTTCAGCATCT<br><br>WNV-wt-NY99-I-Reverse: GCGAAGGACCTCCTGGGTGGC
	wt-NY99_**Fragment-II**	pET-28a(+)	synthetic clonal genes	WNV-wt-NY99-II-Forward: TATAATGCTGATATGATTGACC<br><br>WNV-wt-NY99-II-Reverse: AGTGAACTCTTCTTTTGTCATC
	wt-NY99_**Fragment-III**	pET-28a(+)	synthetic clonal genes	WNV-wt-NY99-III-Forward: GGTGGGGCAAAAGGACGCACCT<br><br>WNV-wt-NY99-III-Reverse: CTCAGGGTCAATGCCAGCGCTT
**E/NS1-Per**				
	NY99-Pr-E-NS1_**Fragment-I-A**<br><br>NY99-Pr-E-NS1_**Fragment-I-B**<br><br>NY99-Pr-E-NS1_**Fragment-I-C**<br><br>NY99-Pr-E-**NS1_Fragment-I-D**	NA<br><br>NA<br><br>NA<br><br>NA	*synthesis of DNA*<br><br>*synthesis of DNA*<br><br>*synthesis of DNA*<br><br>*synthesis of DNA*	Fr-NY99-Per-FI-A: CACCCAACTGATCTTCAGCATCT<br>Rv-NY99-Per-FI-A: ATCATGACTGCAATTCCGGTC<br><br>Fr-NY99-Per-FI-B: TGACCAGTGCTATCAATCGGC<br>Rv-NY99-Per-FI-B: TATGATGGAGCTGCTGGTGTG<br><br>Fr-NY99-Per-FI-C: CGGCAACTACTCCACCCAG<br>Rv-NY99-Per-FI-C: CGTTTCTGGGTAATACTTGTACCGAT<br><br>Fr-NY99-Per-FI-D: TGTGGAAGTGGGGTGTTCATAC<br>Rv-NY99-Per-FI-D: GCGAAGGACCTCCTGGGT
	wt-NY99_**Fragment-II**	pET-28a(+)	synthetic clonal genes	WNV-wt-NY99-II-Forward: TATAATGCTGATATGATTGACC<br><br>WNV-wt-NY99-II-Reverse: AGTGAACTCTTCTTTTGTCATC
	wt-NY99_**Fragment-III**	pET-28a(+)	synthetic clonal genes	WNV-wt-NY99-III-Forward: GGTGGGGCAAAAGGACGCACCT<br><br>WNV-wt-NY99-III-Reverse: CTCAGGGTCAATGCCAGCGCTT
**E/NS1/NS5-Per**				
	NY99-Pr-E-NS1_**Fragment-I-A**	NA	*synthesis of DNA*	Fr-NY99-Per-FI-A: CACCCAACTGATCTTCAGCATCT

	NY99-Pr-E-NS1_**Fragment-I-B**<br><br>NY99-Pr-E-NS1_**Fragment-I-C**<br><br>NY99-Pr-E-**NS1_Fragment-I-D**	NA<br><br>NA<br><br>NA	*synthesis of DNA*<br><br>*synthesis of DNA*<br><br>*synthesis of DNA*	Rv-NY99-Per-FI-A:<br>ATCATGACTGCAATTCCGGTC<br><br>Fr-NY99-Per-FI-B:<br>TGACCAGTGCTATCAATCGGC<br>Rv-NY99-Per-FI-B:<br>TATGATGGAGCTGCTGGTGTG<br><br>Fr-NY99-Per-FI-C:<br>CGGCAACTACTCCACCCAG<br>Rv-NY99-Per-FI-C:<br>CGTTTCTGGGTAATACTTGTACCGAT<br><br>Fr-NY99-Per-FI-D:<br>TGTGGAAGTGGGGTGTTCATAC<br>Rv-NY99-Per-FI-D:<br>GCGAAGGACCTCCTGGGT
	wt-NY99_**Fragment-II**	pET-28a(+)	synthetic clonal genes	WNV-wt-NY99-II-Forward:<br>TATAATGCTGATATGATTGACC<br><br>WNV-wt-NY99-II-Reverse:<br>AGTGAACTCTTCTTTTGTCATC
	NY99-Permuted-NS5_**Fragment-III**	pET-28a(+)	synthetic clonal genes	WNV-wt-NY99-III-Forward:<br>GGTGGGGCAAAAGGACGCACCT<br><br>WNV-wt-NY99-III-Reverse:<br>CTCAGGGTCAATGCCAGCGCTT
**E+CG**				
	NY99-CpG-E_**Fragment-I**	pET-28a(+)	synthetic clonal genes	WNV-wt-NY99-I-Forward:<br>CACCCAACTGATCTTCAGCATCT<br><br>WNV-wt-NY99-I-Reverse:<br>GCGAAGGACCTCCTGGGTGGC
	wt-NY99_**Fragment-II**	pET-28a(+)	synthetic clonal genes	WNV-wt-NY99-II-Forward:<br>TATAATGCTGATATGATTGACC<br><br>WNV-wt-NY99-II-Reverse:<br>AGTGAACTCTTCTTTTGTCATC
	wt-NY99_**Fragment-III**	pET-28a(+)	synthetic clonal genes	WNV-wt-NY99-III-Forward:<br>GGTGGGGCAAAAGGACGCACCT<br><br>WNV-wt-NY99-III-Reverse:<br>CTCAGGGTCAATGCCAGCGCTT
**E/NS1+CG**				
	NY99-CpG-E-NS1_**Fragment-I**	pET-28a(+)	synthetic clonal genes	WNV-wt-NY99-I-Forward:<br>CACCCAACTGATCTTCAGCATCT<br><br>WNV-wt-NY99-I-Reverse:<br>GCGAAGGACCTCCTGGGTGGC
	wt-NY99_**Fragment-II**	pET-28a(+)	synthetic clonal genes	WNV-wt-NY99-II-Forward:<br>TATAATGCTGATATGATTGACC<br><br>WNV-wt-NY99-II-Reverse:<br>AGTGAACTCTTCTTTTGTCATC
	wt-NY99_**Fragment-III**	pET-28a(+)	synthetic clonal genes	WNV-wt-NY99-III-Forward:<br>GGTGGGGCAAAAGGACGCACCT<br><br>WNV-wt-NY99-III-Reverse:<br>CTCAGGGTCAATGCCAGCGCTT
**E/NS1/NS5+CG**				
	NY99-CpG-E-NS1_**Fragment-I**	pET-28a(+)	synthetic clonal genes	WNV-wt-NY99-I-Forward:<br>CACCCAACTGATCTTCAGCATCT

				WNV-wt-NY99-I-Reverse: GCGAAGGACCTCCTGGGTGGC
	wt-NY99_**Fragment-II**	pET-28a(+)	synthetic clonal genes	WNV-wt-NY99-II-Forward: TATAATGCTGATATGATTGACC<br><br>WNV-wt-NY99-II-Reverse: AGTGAACTCTTCTTTTGTCATC
	NY99-CpG-NS5_**Fragment-III**	pET-28a(+)	synthetic clonal genes	WNV-wt-NY99-III-Forward: GGTGGGGCAAAAGGACGCACCT<br><br>WNV-wt-NY99-III-Reverse: CTCAGGGTCAATGCCAGCGCTT
**E-MAX**				
	NY99-E_CpG+UpA_**Fragment-I**	pET-28a(+)	synthetic clonal genes	WNV-wt-NY99-I-Forward: CACCCAACTGATCTTCAGCATCT<br><br>WNV-wt-NY99-I-Reverse: GCGAAGGACCTCCTGGGTGGC
	wt-NY99_**Fragment-II**	pET-28a(+)	synthetic clonal genes	WNV-wt-NY99-II-Forward: TATAATGCTGATATGATTGACC<br><br>WNV-wt-NY99-II-Reverse: AGTGAACTCTTCTTTTGTCATC
	wt-NY99_**Fragment-III**	pET-28a(+)	synthetic clonal genes	WNV-wt-NY99-III-Forward: GGTGGGGCAAAAGGACGCACCT<br><br>WNV-wt-NY99-III-Reverse: CTCAGGGTCAATGCCAGCGCTT
**E+UA**				
	NY99-E+UA_**Fragment-IA**<br><br>NY99-E+UA_**Fragment-IB**<br><br>NY99-E+UA_**Fragment-IC**	NA<br><br>NA<br><br>NA	*synthesis of DNA*<br><br>*synthesis of DNA*<br><br>*synthesis of DNA*	NY99-E+UA-I-A-Forward: CACCCAACTGATCTTCAGCATCT<br>NY99-E+UA-I-A-Reverse: TCCGTGTGTCTGCACTGTCAG<br><br>NY99-E+UA-I-B- Forward: AGGTATGGAAGATGCACCAAGAC<br>NY99-E+UA-I-B- Reverse: TGATCTGTTGTTCCCCTCTGCC<br><br>NY99-E+UA-I-C-Forward: GGTACTCATTGAATTGGAGCCAC<br>NY99-E+UA-I-C-Reverse: GCGAAGGACCTCCTGGGTGGC
	wt-NY99_**Fragment-II**	pET-28a(+)	synthetic clonal genes	WNV-wt-NY99-II-Forward: TATAATGCTGATATGATTGACC<br><br>WNV-wt-NY99-II-Reverse: AGTGAACTCTTCTTTTGTCATC
	wt-NY99_**Fragment-III**	pET-28a(+)	synthetic clonal genes	WNV-wt-NY99-III-Forward: GGTGGGGCAAAAGGACGCACCT<br><br>WNV-wt-NY99-III-Reverse: CTCAGGGTCAATGCCAGCGCTT
**E-MAX/NS5+CG**				
	NY99-E_CpG+UpA_**Fragment-I**	pET-28a(+)	synthetic clonal genes	WNV-wt-NY99-I-Forward: CACCCAACTGATCTTCAGCATCT<br><br>WNV-wt-NY99-I-Reverse: GCGAAGGACCTCCTGGGTGGC

	wt-NY99_**Fragment-II**	pET-28a(+)	synthetic clonal genes	WNV-wt-NY99-II-Forward: TATAATGCTGATATGATTGACC<br><br>WNV-wt-NY99-II-Reverse: AGTGAACTCTTCTTTTGTCATC
	NY99-CpG-NS5_**Fragment-III**	pET-28a(+)	synthetic clonal genes	WNV-wt-NY99-III-Forward: GGTGGGGCAAAAGGACGCACCT<br><br>WNV-wt-NY99-III-Reverse: CTCAGGGTCAATGCCAGCGCTT
**E-MAX/NS5-MAX**				
	NY99-E_CpG+UpA_**Fragment-I**	pET-28a(+)	synthetic clonal genes	WNV-wt-NY99-I-Forward: CACCCAACTGATCTTCAGCATCT<br><br>WNV-wt-NY99-I-Reverse: GCGAAGGACCTCCTGGGTGGC
	wt-NY99_**Fragment-II**	pET-28a(+)	synthetic clonal genes	WNV-wt-NY99-II-Forward: TATAATGCTGATATGATTGACC<br><br>WNV-wt-NY99-II-Reverse: AGTGAACTCTTCTTTTGTCATC
	NY99-CpG-NS5-MAX_**Fragment-III-A**<br><br>NY99-CpG-NS5-MAX_**Fragment-III-B**<br><br>NY99-CpG-NS5-MAX_**Fragment-III-C**	NA<br><br>NA<br><br>NA	*synthesis of DNA*<br><br>*synthesis of DNA*<br><br>*synthesis of DNA*	NY99-CpG-NS5_MAX-III-A-Forward: GGTGGGGCAAAAGGACGCACCT<br>NY99-CpG-NS5_MAX-III-A-Reverse: GAGGACGTTTTTCGCGTGCTA<br><br>NY99-CpG-NS5_MAX-III-B-Forward: CCGGAAGGCGTGAAGTACGTG<br>NY99-CpG-NS5_MAX-III-B-Reverse: GCCGAACAAATCGCGTTCGC<br><br>NY99-CpG-NS5_MAX-III-C-Forward: CGTACGCGCAGATGTGGCT<br>NY99-CpG-NS5_MAX-III-C-Reverse: CTCAGGGTCAATGCCAGCGCTT
**WNV-WT+FVR**				
	wt-NY99-E-FVR_**Fragment-I-A**<br><br>wt-NY99-E-FVR_**Fragment-I-B**<br><br>wt-NY99-E-FVR_**Fragment-I-C**	NA<br><br>NA<br><br>NA	*synthesis of DNA*<br><br>*synthesis of DNA*<br><br>*synthesis of DNA*	NY99-IA-WT-FVR-Forward: CACCCAACTGATCTTCAGCATCT<br>NY99-IA-WT-FVR-Reverse: CCGTGTGTCTGCACTGTCAGTG<br><br>NY99-IB-WT-FVR-Forward: GGTATGGAAGATGCACCAAGACAC<br>NY99-IB-WT-FVR-Reverse: CTCTGCGCTCCTTTGAGGGTG<br><br>NY99-IC-WT-FVR-Forward: ATCACCATTGGCACAAGTCTGG<br>NY99-IC-WT-FVR-Reverse: GCGAAGGACCTCCTGGGTGGC
	wt-NY99_**Fragment-II**	pET-28a(+)	synthetic clonal genes	WNV-wt-NY99-II-Forward: TATAATGCTGATATGATTGACC<br><br>WNV-wt-NY99-II-Reverse: AGTGAACTCTTCTTTTGTCATC
	wt-NY99_**Fragment-III**	pET-28a(+)	synthetic clonal genes	WNV-wt-NY99-III-Forward: GGTGGGGCAAAAGGACGCACCT<br><br>WNV-wt-NY99-III-Reverse: CTCAGGGTCAATGCCAGCGCTT
**WNV-WT+FR**				

	wt-NY99-E-FVR_**Fragment-I-A**	NA	*synthesis of DNA*	NY99-IA-WT-FVR-Forward: CACCCAACTGATCTTCAGCATCT<br>NY99-IA-WT-FVR-Reverse: CCGTGTGTCTGCACTGTCAGTG
	wt-NY99-E-FR_**Fragment-I-B**	NA	*synthesis of DNA*	NY99-IB-WT-FVR-Forward: GGTATGGAAGATGCACCAAGACAC<br>NY99-IB-WT-FVR-Reverse: CTCTGCGCTCCTTTGAGGGTG
	wt-NY99-E-FVR_**Fragment-I-C**	NA	*synthesis of DNA*	NY99-IC-WT-FVR-Forward: ATCACCATTGGCACAAGTCTGG<br>NY99-IC-WT-FVR-Reverse: GCGAAGGACCTCCTGGGTGGC
	wt-NY99_**Fragment-II**	pET-28a(+)	synthetic clonal genes	WNV-wt-NY99-II-Forward: TATAATGCTGATATGATTGACC<br>WNV-wt-NY99-II-Reverse: AGTGAACTCTTCTTTTGTCATC
	wt-NY99_**Fragment-III**	pET-28a(+)	synthetic clonal genes	WNV-wt-NY99-III-Forward: GGTGGGGCAAAAGGACGCACCT<br>WNV-wt-NY99-III-Reverse: CTCAGGGTCAATGCCAGCGCTT
**E-MAX+FVR**				
	wt-NY99-E-MAX-FVR_**Fragment-I**	pET-28a(+)	synthetic clonal genes	WNV-wt-NY99-I-Forward: CACCCAACTGATCTTCAGCATCT<br>WNV-wt-NY99-I-Reverse: GCGAAGGACCTCCTGGGTGGC
	wt-NY99_**Fragment-II**	pET-28a(+)	synthetic clonal genes	WNV-wt-NY99-II-Forward: TATAATGCTGATATGATTGACC<br>WNV-wt-NY99-II-Reverse: AGTGAACTCTTCTTTTGTCATC
	wt-NY99_**Fragment-III**	pET-28a(+)	synthetic clonal genes	WNV-wt-NY99-III-Forward: GGTGGGGCAAAAGGACGCACCT<br>WNV-wt-NY99-III-Reverse: CTCAGGGTCAATGCCAGCGCTT
**E-MAX+FR**				
	wt-NY99-E-MAX-FR_**Fragment-I**	pET-28a(+)	synthetic clonal genes	WNV-wt-NY99-I-Forward: CACCCAACTGATCTTCAGCATCT<br>WNV-wt-NY99-I-Reverse: GCGAAGGACCTCCTGGGTGGC
	wt-NY99_**Fragment-II**	pET-28a(+)	synthetic clonal genes	WNV-wt-NY99-II-Forward: TATAATGCTGATATGATTGACC<br>WNV-wt-NY99-II-Reverse: AGTGAACTCTTCTTTTGTCATC
	wt-NY99_**Fragment-III**	pET-28a(+)	synthetic clonal genes	WNV-wt-NY99-III-Forward: GGTGGGGCAAAAGGACGCACCT<br>WNV-wt-NY99-III-Reverse: CTCAGGGTCAATGCCAGCGCTT
