# Supplementary material for: West Nile virus vaccine candidates attenuated by dinucleotide enrichment are immunogenic and protective against lethal infection
Source: PLoS Pathog. 2025 Oct 3;21(10):e1013560. doi: 10.1371/journal.ppat.1013560 (PMC12513643; doi:10.1371/journal.ppat.1013560)
Supplement: S6 Table — (PDF) [file ppat.1013560.s015.pdf]

Table S6 Primers for PrimalSeq and Sanger sequencing

Primal Scheme primers

Primer name	Sequence	Pool
E-MAX-FR_1_LEFT_1	AGTTCGCCTGTGTGAGCTGA	1
E-MAX-FR_1_RIGHT_1	GCGATCAGGCCAATCATGACTG	1
E-MAX-FR_2_LEFT_1	GCTCAAAACAAAAGAAAAGAGGAGGAAA	2
E-MAX-FR_2_RIGHT_1	GGTTCCTCAAGATCCATGATTCTGTTTT	2
E-MAX-FR_3_LEFT_1	AGCACTCTAGCGAACAAGAAGGG	1
E-MAX-FR_3_RIGHT_1	GGATCCGCCCGTTTATCGTTGT	1
E-MAX-FR_4_LEFT_1	GCAGTTATTGTTATTTAGCAACCGTAAGC	2
E-MAX-FR_4_RIGHT_1	CGTTTTCGTTCCGACCGTCAT	2
E-MAX-FR_5_LEFT_1	AATATGGCGAGGTTACGGTCGA	1
E-MAX-FR_5_RIGHT_1	ACGGTCCATCGGTTCCGGTATA	1
E-MAX-FR_6_LEFT_1	AAGCGTTCAAGTTTCTCGGTACAC	2
E-MAX-FR_6_RIGHT_1	TCCACGACATTCCGCCGAAA	2
E-MAX-FR_7_LEFT_1	CGAGCCGTACATCAAGTATTCGG	1
E-MAX-FR_7_RIGHT_1	CTGTTTCTCAACCACGACACTAAGG	1
E-MAX-FR_8_LEFT_1	CGAGCTGAACACTCTTTTGAAGGAG	2
E-MAX-FR_8_RIGHT_1	TCTCAGGCCACGTACATGATTTGA	2
E-MAX-FR_9_LEFT_1	GTGGAAGCTTGAAAGGGCAGTT	1
E-MAX-FR_9_RIGHT_1	GCCCAACTGAAAAGGGTCAATCATAT	1
E-MAX-FR_10_LEFT_1	CCCTCGTGCAGTCACAAGTGAA	2
E-MAX-FR_10_RIGHT_1	GTTATGGCTCTCAGTATCATCCAAGC	2
E-MAX-FR_11_LEFT_1	GCCAAATTCTGCTCTGGGAGATC	1
E-MAX-FR_11_RIGHT_1	AAATCACGAAAGCAGCAAACATGAG	1
E-MAX-FR_12_LEFT_1	GGCAGAGCTTGACATTGACTCC	2
E-MAX-FR_12_RIGHT_1	GCTCCTGCTTGATAACTGCCGA	2
E-MAX-FR_13_LEFT_1	CGGCGTCTACAGGATCATGACT	1
E-MAX-FR_13_RIGHT_1	GGCTCATCCATCCTTTCACCCT	1
E-MAX-FR_14_LEFT_1	GATTGGGCTTTATGGCAATGGAGT	2
E-MAX-FR_14_RIGHT_1	TGTAACCTCTTGCTGCAATGCTAG	2
E-MAX-FR_15_LEFT_1	GTTCGTGATGGATGAGGCTCATTT	1
E-MAX-FR_15_RIGHT_1	CTGTTATGATGGTTGGTTTCACACTCT	1
E-MAX-FR_16_LEFT_1	TTCAAGGCGAGCAGGGTGAT	2
E-MAX-FR_16_RIGHT_1	TTGTGTTTGTCCTAGGACCATCAAAG	2
E-MAX-FR_17_LEFT_1	CAGTTTGGCTGGCTTACAAGGTT	1
E-MAX-FR_17_RIGHT_1	CCGCTGCATGAGGAGGAAGAAT	1
E-MAX-FR_18_LEFT_1	ATGCTCTTCAGACAATTGCCTTGAT	2
E-MAX-FR_18_RIGHT_1	TGAGGACCGCTGTTGTCACA	2
E-MAX-FR_19_LEFT_1	GTTTCTTCTGGACTTGAGGCCG	1
E-MAX-FR_19_RIGHT_1	TGCAGCTAGAGACACCAAGATCA	1
E-MAX-FR_20_LEFT_1	CCCATCATGCAGAAGAAAGTTGGA	2
E-MAX-FR_20_RIGHT_1	CCAGCCATCTCAGTTTTGCTGTG	2

E-MAX-FR_21_LEFT_1	AGGAAAGAAGGCAATGTCACTGGA	1
E-MAX-FR_21_RIGHT_1	GAGCAGCTCCATCTTCTCTATGACT	1
E-MAX-FR_22_LEFT_1	TTTTGCGTGAAGGTGCTCTGTC	2
E-MAX-FR_22_RIGHT_1	GTTTTGAGAGGAGCCTGACCACT	2
E-MAX-FR_23_LEFT_1	TCACGGCAGTTATGATGTGAAGC	1
E-MAX-FR_23_RIGHT_1	TTTTTCTCTCTCTTTCCCATCATGTTGT	1
E-MAX-FR_24_LEFT_1	TTTGGGAGATGGTGGATGAGGAG	2
E-MAX-FR_24_RIGHT_1	CGGGCGCATCACTTTCACAA	2
E-MAX-FR_25_LEFT_1	TCTTGCCAGGGCCATCATTGA	1
E-MAX-FR_25_RIGHT_1	CAATTCAGTGAAATGGTTTGAGCAAAATG	1
E-MAX-FR_26_LEFT_1	AGAGTGGAAACCGTCAACTGGA	2
E-MAX-FR_26_RIGHT_1	ACCAGATGTCCTCTCGTTTTCCTG	2
E-MAX-FR_27_LEFT_1	TGGAAGACAAAACCCCAGTGGA	1
E-MAX-FR_27_RIGHT_1	GAGACGGTTCTGAGGGCTTACA	1
E-MAX-FR_28_LEFT_1	CGGAAGTTGAGTAGACGGTGCT	2
E-MAX-FR_28_RIGHT_1	GCTGGTTGTGCAGAGCAGAAGA	2

Sanger sequencing

WNV variant	Mutation	Amplicon size	Primers
**WNV-WT+FVR**	F mutation	491 bp	NY99WT-E-Fm-Forward:<br>CGACAGCTGCGTGACTATCATG<br><br>NY99WT-E-Fm-Reverse:<br>CACAGTCCACTGTCACCTCTCC
	V mutation	485 bp	NY99WT-E-Vm-Forward:<br>CTGTGTGGAGGAACAGAGAGAC<br><br>NY99WT-E-Vm-Reverse:<br>CTGTTGTTCTCCTCTGCCCACC
	R mutation	486 bp	NY99WT-E-Rm-Forward:<br>GTTCCTATCTCGTCAGTGGCTTC<br><br>NY99WT-E-Rm-Reverse:<br>CACGTTCACGGAGAGGAAGAGC
**E-MAX+FVR**	F mutation	491 bp	NY99EMax-E-Fm-Forward:<br>CGAAGGGGATAGTTGCGTTACG<br><br>NY99EMax-E-Fm-Reverse:<br>CGACCGTAACCTCGCCATATTCG
	V mutation	486 bp	NY99EMax-E-Vm-Forward:<br>GGAATTCGAAGAACCGCACGC<br><br>NY99EMax-E-Vm-Reverse:<br>CTTCCGGACTTATGCCAATGGTG
	R mutation	486 bp	NY99EMax-E-Rm-Forward:<br>GTTCCGATATCGTCCGTGGCG<br><br>NY99EMax-E-Rm-Reverse:<br>CACGTTCACGGAGAGGAAGAGC
**WNV-WT+FR**	F mutation	491 bp	NY99WT-E-Fm-Forward:<br>CGACAGCTGCGTGACTATCATG<br><br>NY99WT-E-Fm-Reverse:<br>CACAGTCCACTGTCACCTCTCC
	R mutation	486 bp	NY99WT-E-Rm-Forward:<br>GTTCCTATCTCGTCAGTGGCTTC<br><br>NY99WT-E-Rm-Reverse:<br>CACGTTCACGGAGAGGAAGAGC
**E-MAX+FR**	F mutation	491 bp	NY99EMax-E-Fm-Forward:<br>CGAAGGGGATAGTTGCGTTACG<br><br>NY99EMax-E-Fm-Reverse:<br>CGACCGTAACCTCGCCATATTCG
	R mutation	486 bp	NY99EMax-E-Rm-Forward:<br>GTTCCGATATCGTCCGTGGCG<br><br>NY99EMax-E-Rm-Reverse:<br>CACGTTCACGGAGAGGAAGAGC
